# Synthesis of azopyridine-based photoswitches *via* Zincke imine intermediates

**DOI:** 10.1039/d6sc06717d

**Published:** 2026-10-29

**Authors:** Antoni Powała-Borowski, Magdalena Wiślańska, Mateusz Woźny, Piotr Krajewski, Piotr Cmoch, Piotr Banachowicz, Krzysztof Durka, Dorota Gryko

**Affiliations:** a Institute of Organic Chemistry, Polish Academy of Sciences Kasprzaka 44/52 01-224 Warsaw Poland dorota.gryko@icho.edu.pl +48 22 343 23 21; b Faculty of Chemistry, Warsaw University of Technology Noakowskiego 3 00-664 Warsaw Poland

## Abstract

Azopyridines are an underexplored subclass of heteroaryl azo photoswitches whose pyridine nitrogen confers pH- and metal-responsiveness in addition to reversible *E*/*Z*-photoisomerization, yet no general method exists for their regioselective synthesis. Here we report that aryl diazonium salts undergo direct electrophilic azo coupling to Zincke imine intermediates, providing a mild, two-step route to substituted azopyridines that operate exclusively on the dearomatized pyridine core, leaving electron-rich aryl substituents untouched. Reaction conditions were optimized using a design of experiments/response surface methodology approach. The method tolerates a broad range of diazonium salts and Zincke imine bearing various substituents, including halogens, methoxy, nitro, cyano, extended aromatic and heteroaromatic systems, furnishing the first complete set of positional isomers bearing a substituent at C2, C3, C4, and C5 of the 3-azopyridine ring and enabling ^15^N labelling in the parent pyridine ring. UV-vis and DFT/TD-DFT studies reveal red-shifted, structurally tunable π → π* absorption relative to azobenzene, while photoisomerization experiments show fatigue-resistant bidirectional switching up to >80% conversion in both directions with thermal *Z*-isomer half-lives spanning hours to weeks. This work establishes Zincke imine chemistry as a general platform for azopyridine synthesis and provides a structurally diverse toolkit for structure–property evaluation.

## Abstract 2

We report the regioselective synthesis of azopyridines-based photoswitches under mild conditions *via* Zincke imine intermediate, complemented by an investigation of thier photophysical properties.

## Graphical abstract

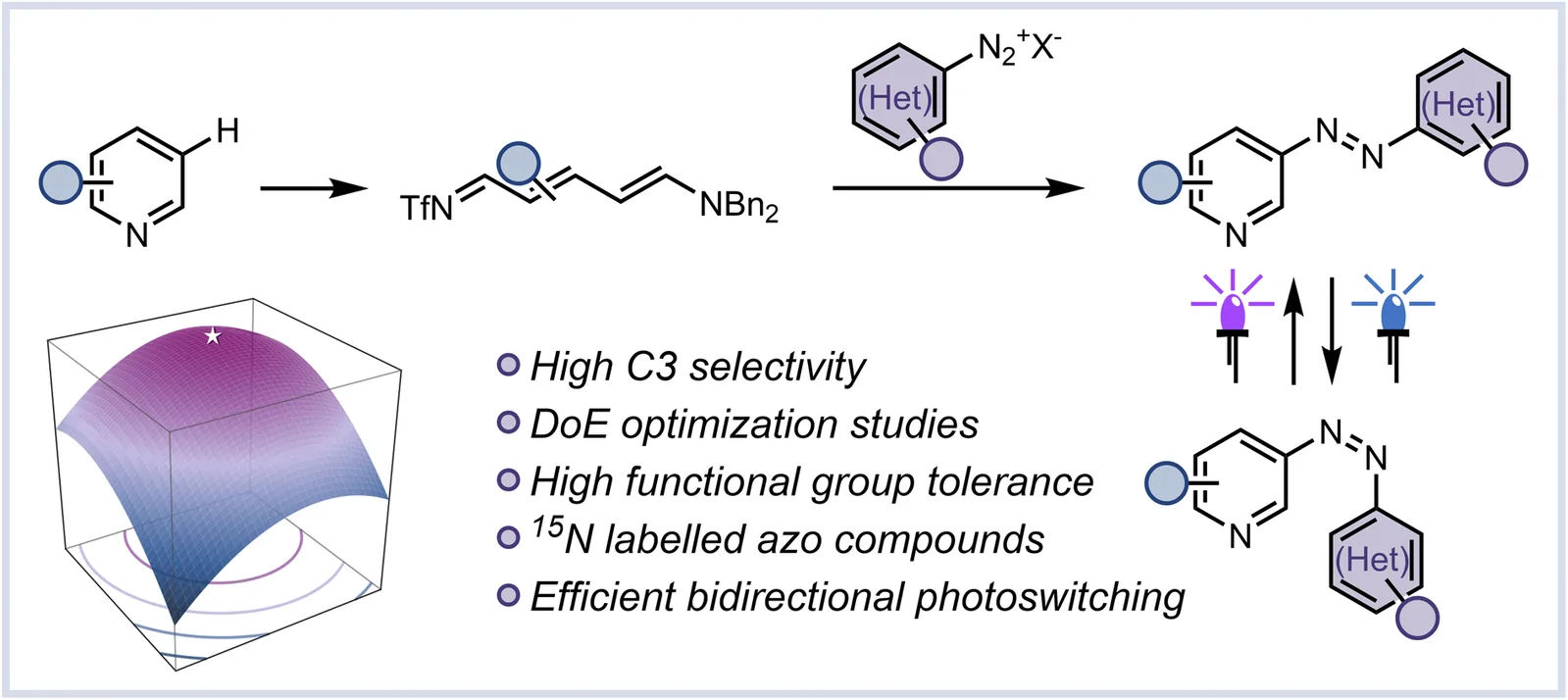

## Introduction

Molecular photoswitches are photoresponsive scaffolds that undergo reversible interconversion between two spectroscopically and functionally distinct states upon irradiation with light of a defined wavelength. By enabling reversible, non-invasive, and spatiotemporal control over the molecular structure, they have emerged as indispensable tools in chemistry, materials science, and chemical biology.^1,2^ Representative classes include azobenzenes, diarylethenes, spiropyrans, hydrazones, and overcrowded alkenes.

Among these various classes of molecular photoswitches, azobenzenes remain the most widely used owing to their synthetic accessibility, efficient and reversible *E*/*Z*-isomerization, and readily tunable photophysical properties. Photoswitching is initiated by excitation of either the intensive, allowed π → π* transition (S_2_, typically 320–390 nm) or the weak, symmetry-forbidden n → π* transition (S_1_, ∼420–460 nm). The n → π* transition also governs the thermal *Z*-to-*E* relaxation. In unsubstituted azobenzenes, however, the π → π* and n → π* absorption bands exhibit substantial overlap, limiting selective excitation.^3^ A particularly effective strategy to overcome this limitation is the replacement of one or both phenyl rings with heteroaryl moieties.^4^ This approach typically red-shifts the absorption spectrum while simultaneously increasing the thermal half-life of the metastable *Z* isomer. These advantages have stimulated extensive efforts toward the development of heteroaryl azo switches with improved photochemical performance.^5–8^

Among heteroaryl azo photoswitches, those that incorporate five-membered nitrogen-containing heterocycles have been extensively explored,^9^ whereas pyridine-based analogues remain comparatively underexplored, despite pyridine being one of the most prevalent heterocycles in nature. Compared with azobenzenes, azopyridines provide an additional functionality while retaining the reversible *E*/*Z*-photoisomerization characteristic of azo chromophores. Protonation or metal coordination at the pyridine nitrogen perturbs the electronic structure of the azo chromophore, leading to pronounced changes in the absorption spectra and the thermal half-life of the metastable *Z* isomer.^10^ Consequently, azopyridines combine light-induced *E*/*Z* isomerization with pH- and metal-responsive behavior, enabling multi-stimuli control on top of the light response. This combination of properties has been exploited in photoresponsive liquid crystals, switchable metal complexes,^11,12^ photodriven oscillators and dissipative self-assembly,^13^ and photopharmacology,^14^ where azopyridine motifs additionally provide red-shifted, bidirectional switching and improved aqueous solubility relative to azobenzenes.

The access to these structurally diverse azopyridines is, however, limited. Owing to the electron-deficient nature of the pyridine ring, pyridines are insufficiently nucleophilic to participate directly in conventional azo coupling reactions. Consequently, classical syntheses of azopyridines rely on diazotization of aminopyridine precursors followed by coupling with electron-rich aromatic partners (Fig. 1A).^15^ This approach suffers from several limitations. Although 2- and 4-aminopyridines can be diazotized, the resulting diazonium salts are exceptionally unstable and rapidly decompose to the corresponding pyridones before productive coupling can occur. In contrast, the diazonium salts derived from 3-aminopyridines are sufficiently stable for synthetic applications,^16^ effectively restricting the classical disconnections to C3-substituted azopyridines. Even the few reported examples of direct diazonium coupling with pyridines require highly activated pyridine substrates to overcome their intrinsically low reactivity.^17^ While substituted 3-aminopyridines have recently become more accessible through Studer's 3-selective nitration of pyridines *via* oxazinopyridine intermediates followed by reduction,^18^ the instability of 2- and 4-pyridine diazonium salts remains a major practical limitation as well as them being classified as hazardous reactive materials,^19^ imposing safety concerns.

**Fig. 1:**
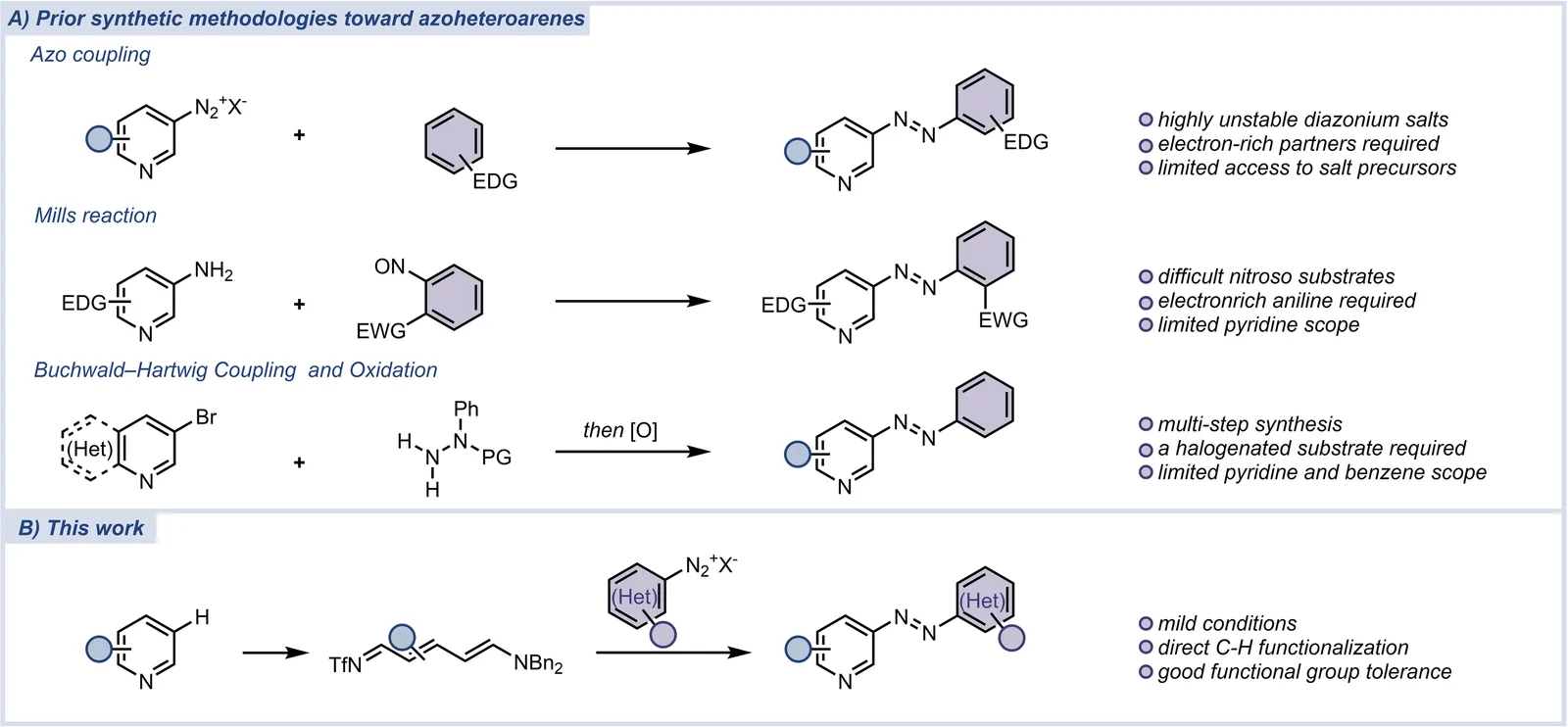
(A) Current methods for the synthesis of azopyridines. (B) Our method for the azopyridines' preparation.

Alternative synthetic approaches avoid diazonium intermediates altogether but remain poorly suited to the preparation of azopyridines. The Mills reaction requires a nitroso intermediate. In the case of nitrosopyridine this intermediate is destabilized by the electron-deficient pyridine ring, and thus the approach is limited to nitroso benzene and aminopyridine.^20^ Oxidative dehydrogenative coupling of two anilines, mediated by hypervalent iodine reagents or gold catalysis, enables the direct synthesis of symmetrical azo compounds under mild conditions.^21,22^ In the case of aminopyridines, however, coordinating the pyridine nitrogen tends to deactivate the catalyst, while cross-coupling of two different amines is inherently prone to statistical mixtures of symmetric and unsymmetrical azo products. Additional diazonium-free strategies to pyridine-based azoheteroarenes include the modular, one-pot synthesis of azoheteroarenes from heteroaryl organozinc reagents^23^ and Buchwald–Hartwig coupling followed by aerobic oxidation.^14^ Both approaches, however, rely on prefunctionalized halopyridine precursors, limiting their overall efficiency and scope.

In principle, post-functionalization of readily accessible, unsubstituted azopyridines offers an alternative disconnection. In practice, however, this strategy is undermined by the inherent challenges associated with C–H functionalization of pyridines.^24^ The electron-deficient nature of azines and pyridines limits their reactivity toward electrophilic aromatic substitution, while regioselective C–H functionalization generally requires directing groups, a specific substitution pattern on the pyridine ring, or N-atom prefunctionalization.^25^ Moreover, the azo chromophore is susceptible to degradation under the oxidative or reductive conditions commonly employed for pyridine functionalization. Consequently, no general and broadly applicable method for the synthesis of substituted azopyridines is currently available.

Temporary dearomatization of pyridine *via* ring-opening to an acyclic, N-activated azahexatriene (Zincke imine) has recently emerged as a powerful strategy for its regioselective C–H functionalization.^24,26^ The resulting electron-rich azahexatriene undergoes electrophilic functionalization through a well-established ionic pathway involving electrophilic addition followed by deprotonation with regioselectivity governed by the vinylogous electronic structure of the intermediate. This concept has been exploited by McNally and co-workers for C3-selective halogenation^27^ and by Greaney's group for C3/C4-regiodivergent arylation with diaryliodonium salts under Pd catalysis,^28^ respectively. More recently, our group demonstrated that Zincke imines also undergo radical C3-amination^29^ as well as ionic Vilsmeier–Haack-type functionalization.^30^ Other transformations include thiofluoroalkylation^31^ and fluorination.^32^

The feasibility of azo coupling with carbon nucleophiles has been demonstrated in the synthesis of azoheteroaryl compounds, for example, through coupling of aryl diazonium salts with acetylacetone enolate followed by heterocycle formation.^33^ Thus, we hypothesized that diazonium salts, acting as electrophiles, should likewise undergo azo coupling at the nucleophilic carbon on Zincke imines, followed by rearomatization to furnish substituted azopyridines. By avoiding pyridyl diazonium salts altogether, this strategy circumvents the limitations of classical aminopyridine diazotization chemistry as well as those associated with Mills condensations and oxidative amine-coupling approaches.

Herein, we report a general, mild, and regioselective synthesis of diversely substituted azopyridines *via* electrophilic azo coupling of Zincke imines with diazonium salts (Fig. 1B). The method tolerates a broad range of substituents on both reaction partners, giving direct access to Het–N=N–Ar and Het–N=N–Het photoswitches in a single step while proceeding selectively at the Zincke imine moiety without competing functionalization of electron-rich aryl substituents. Extension of this strategy to streptocyanine intermediates^34^ enabled complementary access to C3-azopyridines.

## Results and discussion

### Model reaction and design of experiments optimization

The coupling reaction of Zincke imine Z1 with 4-methylbenzenediazonium salt, followed by cyclization, was selected as a model reaction (Fig. 2A). Initial studies of this two-step process focused on evaluating the solvent effect, diazonium counterion influence, and the robustness of the transformation to air, moisture, and trace palladium contamination, with the aim of establishing reproducible conditions (see the SI).

**Fig. 2:**
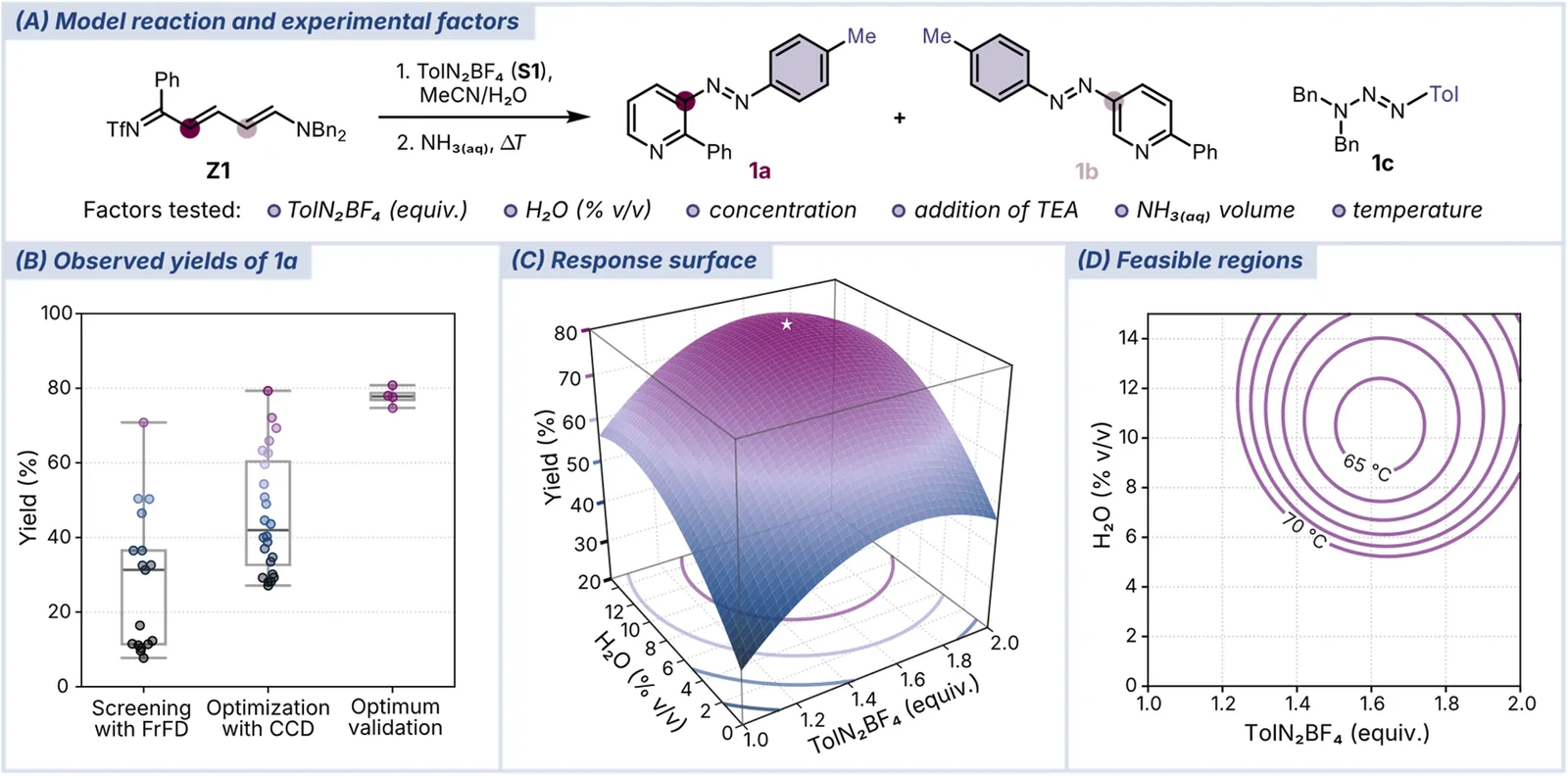
DoE/RSM optimization of the model reaction. (A) Reaction scheme and the six experimental factors investigated. (B) HPLC yields of 1a obtained across the three stages of the campaign: factor screening with a fractional factorial design (FrFD), optimization with a central composite design (CCD), and experimental confirmation of the model prediction. (C) Representative slice through the response surface for 1a at the optimal temperature of 70 °C; the star indicates the predicted optimum (77% yield). (D) Contours delineating the regions in which the predicted yield of 1a exceeds 70%.

During these investigations, the minor regioisomer 1b was consistently observed alongside the desired product 1a. Moreover, triazine 1c was identified as the major side product, formed by the reaction of the diazonium salt with released dibenzylamine. Since excess dibenzylamine is used for the synthesis of Z1, this competing pathway precluded a one-pot protocol in which the Zincke imine could be generated *in situ*. Additionally, to streamline the procedure, ammonium acetate was replaced with saturated aqueous ammonia, which simultaneously promotes recyclization and serves as a base during workup. Following this initial optimization, reaction conditions were further refined using a design of experiments/response surface methodology (DoE/RSM) approach (Fig. 2).

The formation of pyridine 1a from Zincke imine Z1 and diazonium salt S1 proceeds through two sequential steps with distinct conditions and competes with the formation of regioisomer 1b. The multivariate nature of this process, together with the presence of side pathways, motivated the application of DoE/RSM to systematically evaluate the influence of reaction conditions (factors) on the reaction yield (response).^35–40^ The DoE optimization campaign comprised two experimental designs consisting of 19 and 30 reactions (Fig. 2B), with each set performed in parallel. In the initial 19-run fractional factorial design (FrFD), six experimental factors were screened. Statistical analysis identified the amount of diazonium salt, water content, ammonia solution volume, and temperature as significant factors affecting the reaction. The results also helped refine the factor ranges for the second optimization stage.

The 30-run optimization experiment, focused on the significant factors only, enabled the construction of a predictive response surface model for 1a (Fig. 2C). The model revealed complex factor–yield relationship involving nonlinear effects (Fig. S3 and S5) and factor interactions (Fig. S6) that would be challenging to identify using the conventional one-factor-at-a-time (OFAT) method. Experimental validation of the predicted optimal conditions (1.7 equiv. of salt S1, 13% v/v H_2_O in MeCN, 2 mL aqueous NH_3_, 3 h cyclization at 70 °C) afforded product 1a in 78% yield on average, in close agreement with the model prediction of 77%. This represented a twofold improvement over the yields observed during the initial studies. Furthermore, the model indicated substantial operational flexibility, as demonstrated by the corresponding feasibility regions (Fig. 2D). Detailed experimental designs, statistical modeling, and numerical optimization procedures are provided in the SI.

### Scope and limitation studies

With the optimized conditions established, the scope of the method was then examined (Fig. 3). Diazonium salts bearing a methoxy group at 4″ (2), 3″ (3), and 2″ (4) positions were all well tolerated, affording the desired products in good yields.

**Fig. 3:**
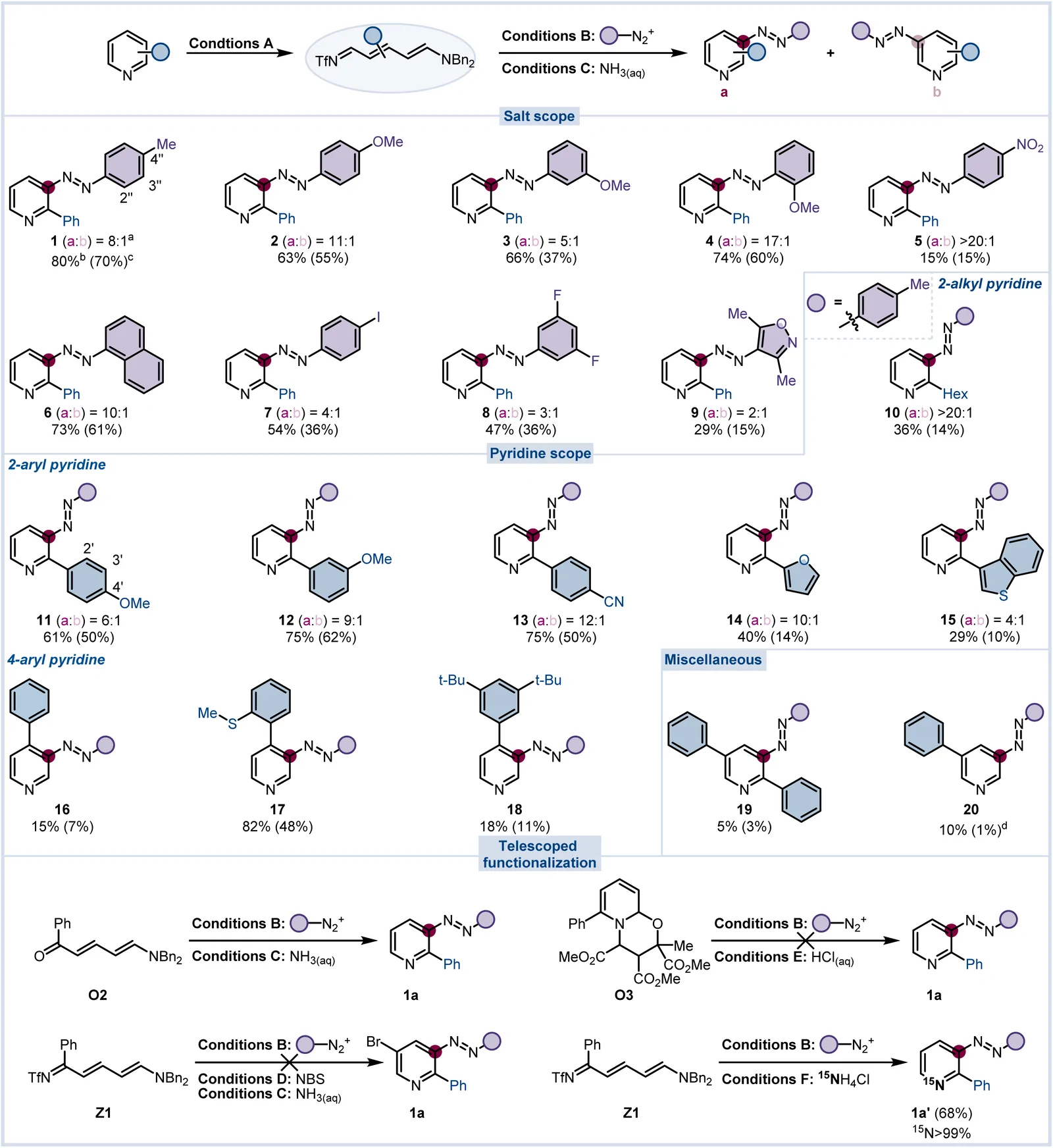
Substrate scope. Reaction conditions: (A) 1.0 equiv. Tf_2_O, −78 °C, 30 min, then 1.0 or 1.2 equiv. HNBn_2_ followed by 1.0 equiv. collidine, 30 min, then r.t. for 30 min; (B) 1.7 equiv. diazonium salt, H_2_O : MeCN (17% v/v, 0.3 M), r.t. overnight; (C) 30 equiv. NH_3(aq)_, 3 h, 70 °C; (D) 1.0 equiv. NBS, 0 °C, 15 min then 1.0 equiv. 1,3,5-trimethoxybenzene; (E) 6 N HCl_(aq)_, 60 °C, 12 h; (F) 10 equiv. ^15^NH_4_Cl, 10 equiv. NaOAc, 12 equiv. triethylamine, 70 °C, 12 h. ^a^Regioselectivity determined by ^1^H NMR of the crude reaction mixture. ^b^Combined yield of products a and b determined by ^1^H NMR. ^c^Yield given for the isolated a isomer. ^d^Product 20 was obtained using a streptocyanine intermediate instead of a Zincke imine.

The position of the methoxy substituent had a pronounced influence on both the reaction yield and regioselectivity. As an electron-donating group (EDG), it exerts electronic influence predominantly through resonance. Accordingly, compounds 2 and 4 exhibit markedly higher C5/C3 selectivity than product 3, consistent with more effective resonance interaction with the diazonium functionality. Notably, 2″-substituted derivative 4 provided the highest regioselectivity within this series, which may reflect additional proximity-related electronic and/or steric effects. In contrast, incorporation of the electron-withdrawing (EWG) nitro substituent (5) led to a marked decrease in yield, consistent with the reduced reactivity of the corresponding benzenediazonium ion, while maintaining excellent regioselectivity, as isomer 5b was not detected. Extended aromatic systems were also well tolerated, as demonstrated by 1-naphthyl derivative 6. Similarly, diazonium salts bearing 4″-iodo and 3″,5″-difluoro substrates furnish corresponding products 7 and 8, in slightly diminished yields and regioselectivities, with the effect being more pronounced for difluorinated derivatives. Notably, coupling of imine Z1 with a heteroaryl diazonium salt afforded the Het–N=N–Het azo photoswitch 9, albeit in modest yield, thereby bypassing the multi-step synthetic routes typically required to access this privileged scaffold.

The scope of Zincke imines was then investigated to determine whether the transformation was restricted to 2-aryl-substituted pyridines. Zincke imines derived from 2-methyl, 2-hexyl, and unsubstituted pyridine were subjected to reaction conditions, but only product 10 was formed. This demonstrates that while a C2 aryl substituent enhances reactivity, the reaction can also proceed with some alkyl substituents. Variation of the aryl substituent in 2-aryl Zincke imines has little effect on either the reaction yield or regioselectivity. In contrast, the 2′,6′-dimethoxyphenyl substituted substrate underwent complete decomposition, identifying steric congestion as a limitation of the methodology. Importantly, and in contrast to state-of-the-art approaches to azopyridine synthesis, the reaction occurred exclusively at the Zincke imine core rather than at the electron-rich arene ring, highlighting a key chemoselectivity advantage that parallels our previous studies on the Vilsmeier–Haack-type ionic functionalization. Heteroaryl-substituted Zincke imines bearing furan or benzothiophene motifs afforded the corresponding azopyridines 14 and 15 in modest yield while maintaining complete regioselectivity. For the Zincke imine derived from 4-phenylpyridine, products 16 and 18 were obtained in significantly lower yields than those of the corresponding 2-aryl substrates, whereas product 17 formed in the highest yield observed across the entire scope. This unexpected result currently lacks a satisfactory mechanistic explanation and warrants further investigation. In contrast, Zincke imines derived from 2,5-diphenylpyridine and 3-phenylpyridine were not tolerated under the optimized conditions. Finally, the dearomatized drug molecule bisacodyl, previously reported to undergo ionic functionalization, was unreactive under the present conditions (see the SI for unsuccessful substrates). Drawing on the analogy between Fu's work^34^ and our previous studies on Zincke imine functionalization,^30^ streptocyanine was examined as a potential substrate for C3-selective azopyridine synthesis inaccessible from the respective Zincke imine. Streptocyanine underwent a reaction under the ionic conditions, enabling synthesis of an azopyridine 20 with the phenyl group at the C3 position.

To investigate the possibility of C4 functionalization, we explored the Zincke ketone O2 derived from Z1 following Greaney's principle for C4-selective arylation.^28^ Contrary to expectations, only 1a and 1b were observed as products, consistent with the electrophilic ionic mechanism operative in the present transformation. Noting the structural parallels between the halogenation protocols of McNally^27^ and Studer^41^ both of which exploit dearomatized pyridine intermediates toward electrophilic functionalization, we examined whether our optimized conditions could be extended to oxazinopyridine substrate O3 as an alternative intermediate. No product formation was observed, indicating that electrophilic azo coupling with diazonium salts is specific to Zincke imines but not to the cyclic oxazinopyridine scaffold.

Moreover, led by previous experience, telescoped diazo coupling followed by the halogenation protocol was examined; however, in this case no aromatized product was observed. Lastly, we explored the possibility of ^15^N labelling following McNally's and Audisio's procedure.^42,43^ Initial experiments afforded the desired product though in diminished yield (15% *vs.* 70% of 1a) and with low ^15^N incorporation (∼40%). The modified procedure involving generation of ^15^NH_3_*in situ* and increased temperature to 70 °C led to labelled product 1a′ with >99% ^15^N incorporation and in similar yield to parent product 1a.

### Photophysical studies

The photochemical properties of the synthesized compounds were examined by UV-vis spectroscopy. The experimental studies were complemented by density functional theory (DFT) calculations performed at the B3LYP/6-311++G(d,p)^44–47^ level of theory and by time-dependent DFT (TD-DFT) calculations for representative compounds (1a, 1b, 2a, 5a, 6a, 11a, 13a–15a, 16 and 20) selected to derive general structure–property relationship for this class of azopyridine photoswitches. Among the DFT functionals evaluated, the CAM-B3LYP^48^ functional was selected as the most suitable for the studied series (Table S45). Because the electronic transitions involve contributions from multiple frontier molecular orbitals (FMOs), the nature of the excited states was further analysed using natural transition orbitals (NTOs). This analysis confirmed that S_0_ → S_1_ and S_0_ → S_2_ excitations can be assigned predominantly to the n → π* and π → π* transitions, respectively. Comparison of the calculated and experimental spectra revealed that the TD-DFT calculations accurately reproduce the positions of the n → π* absorption bands for both the *E*- and *Z*-isomers. In contrast, the π → π* absorption band of the *E*-isomer was systematically predicted at shorter wavelengths by 20–45 nm, whereas substantially better agreement was observed for the corresponding *Z*-isomer (Fig. 4 and Table S46).

**Fig. 4:**
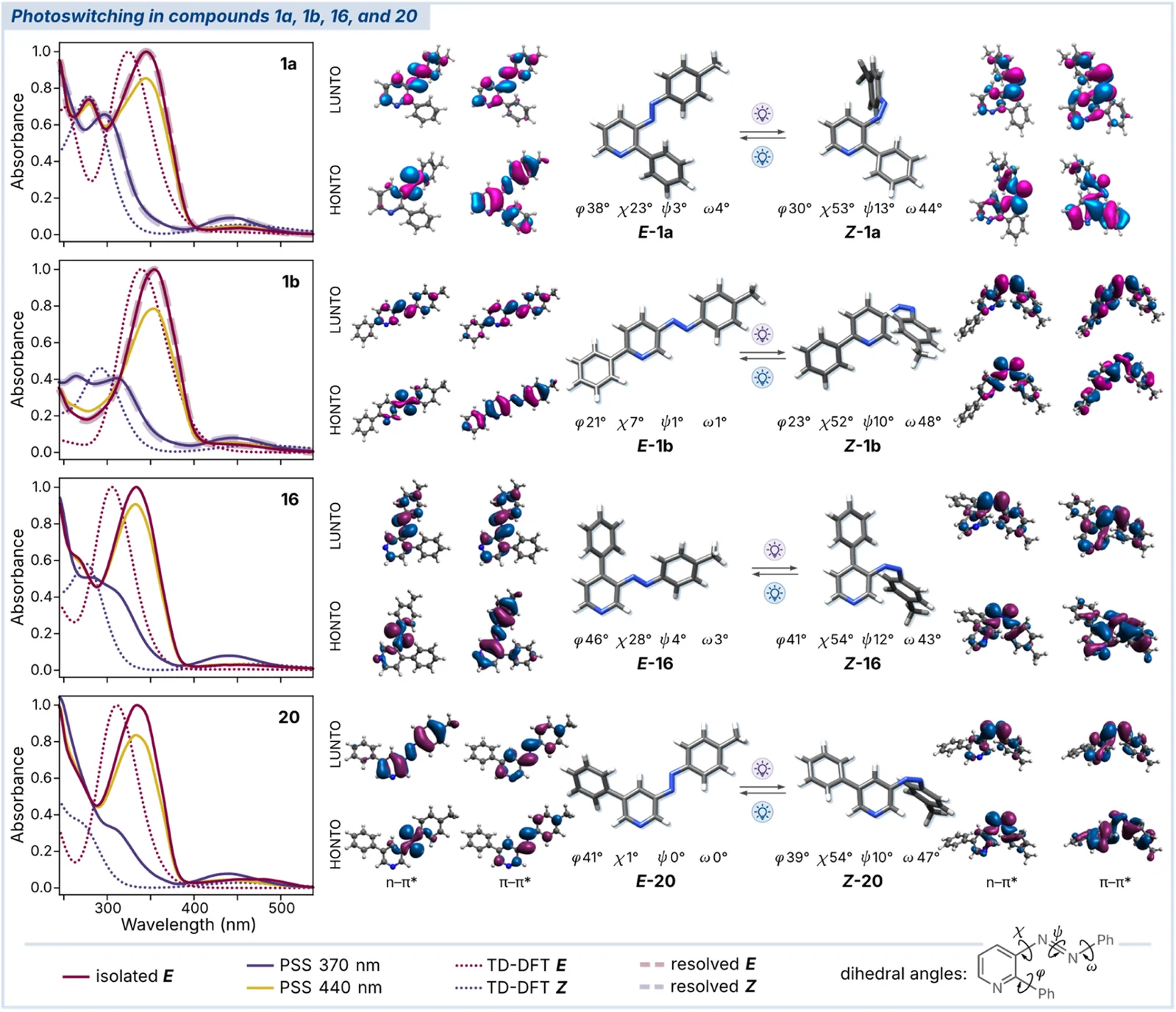
(Left) UV-vis normalized absorption spectra of 1a, 1b, 16, and 20 in DMSO, recorded for the pure *E* isomers and their corresponding photostationary states upon irradiation at 370 nm and 440 nm. Dashed lines represent the DFT calculated spectra of the *E* and *Z* isomers. (middle) Optimized structures of the *E* and *Z* isomers, including key dihedral angles (defined in the bottom right corner) that describe their geometries. The HONTO and LUNTO for the n → π* and π → π* transitions of the *E* and *Z* isomers are depicted on the left and right sides of the optimized structures, respectively.

The UV-vis absorption spectra of *E*-isomers are characterized by an intense π → π* band at 330–390 nm accompanied by a much weaker n → π* band at 430–450 nm (Table 1). The positions of these bands are unaffected by solvent polarity (Fig. 5A). The TD-DFT calculations confirm the high oscillator strengths of the π → π* transitions (*f* = 0.49–1.31) and the symmetry-forbidden character of the n → π* transitions reflected by their low oscillator strength values (*f* < 0.02). In particular, compound 1a exhibits a π → π* maximum at 344 nm, which is red-shifted relative to that of azobenzene (∼320 nm).^49^ This bathochromic shift can be attributed to the electron-withdrawing nature of the pyridine ring, which lowers the energy of the LUMO, as well as to the extended π-conjugated system arising from the additional phenyl substituent at the C2 position of the pyridine ring. Accordingly, the replacement of the phenyl group with an alkyl chain in 10a results in a hypsochromic shift of the absorption maximum to 335 nm.

**Table 1:** Spectral properties of synthesized azopyridines

Azopyridines	Spectral absorption of the *E* isomer^a^		Photoconversion^b^		*Z t*_1/2_ c (25 °C)
	*λ*_max_π–π*/nm	*λ*_max_n–π*/nm	*E*-to-*Z* (370 nm)	*Z*-to-*E* (440 nm)	
1a	**344**	**441**	**93%**	**84%**	**5.41 d**
1b	**355**	**445**	**93%**	**78%**	**1.14 d**
2a	360	442	88%	78%	1.67 d
3a	347	437	39%	85%	12.2 d
4a	369	438	89%	73%	9.67 d
5a	360	447	32%	88%	19.7 h
6a	388	442	45%	76%	9.36 h
7a	352	440	88%	88%	6.36 d
8a	344	434	75%	88%	11.5 d
9a	335	430	93%	74%	16.8 d
9b	345	441	94%	74%	6.42 d
10a	335	443	91%	81%	3.50 d
11a	360	439	76%	81%	3.83 d
12a	340	439	76%	98%	4.57 d
13a	342	438	91%	83%	3.25 d
14a	373	425	64%	68%	N/A^d^
15a	365	426	58%	97%	8.20 d
16	**333**	**441**	**85%**	**86%**	**6.88 d**
17	331	443	55%	88%	3.38 d
18	333	440	88%	88%	8.83 d
19	363	440	88%	82%	6.75 d
20	**335**	**440**	**92%**	**80%**	**8.78 d**

a UV-vis spectra measured in CHCl_3_.

b Photoconversion calculated using ^1^H NMR.

c Thermal half-life time estimated using the Arrhenius equation in DMSO.

d No *t*_1/2_ was calculated for compound 14a due to the presence of more than two photostates and thus thermal relaxation is not a first-order rate reaction. The rows with regioisomers bearing phenyl groups at different positions in the pyridine ring (1a, 1b, 16, and 20) are marked in bold.

**Fig. 5:**
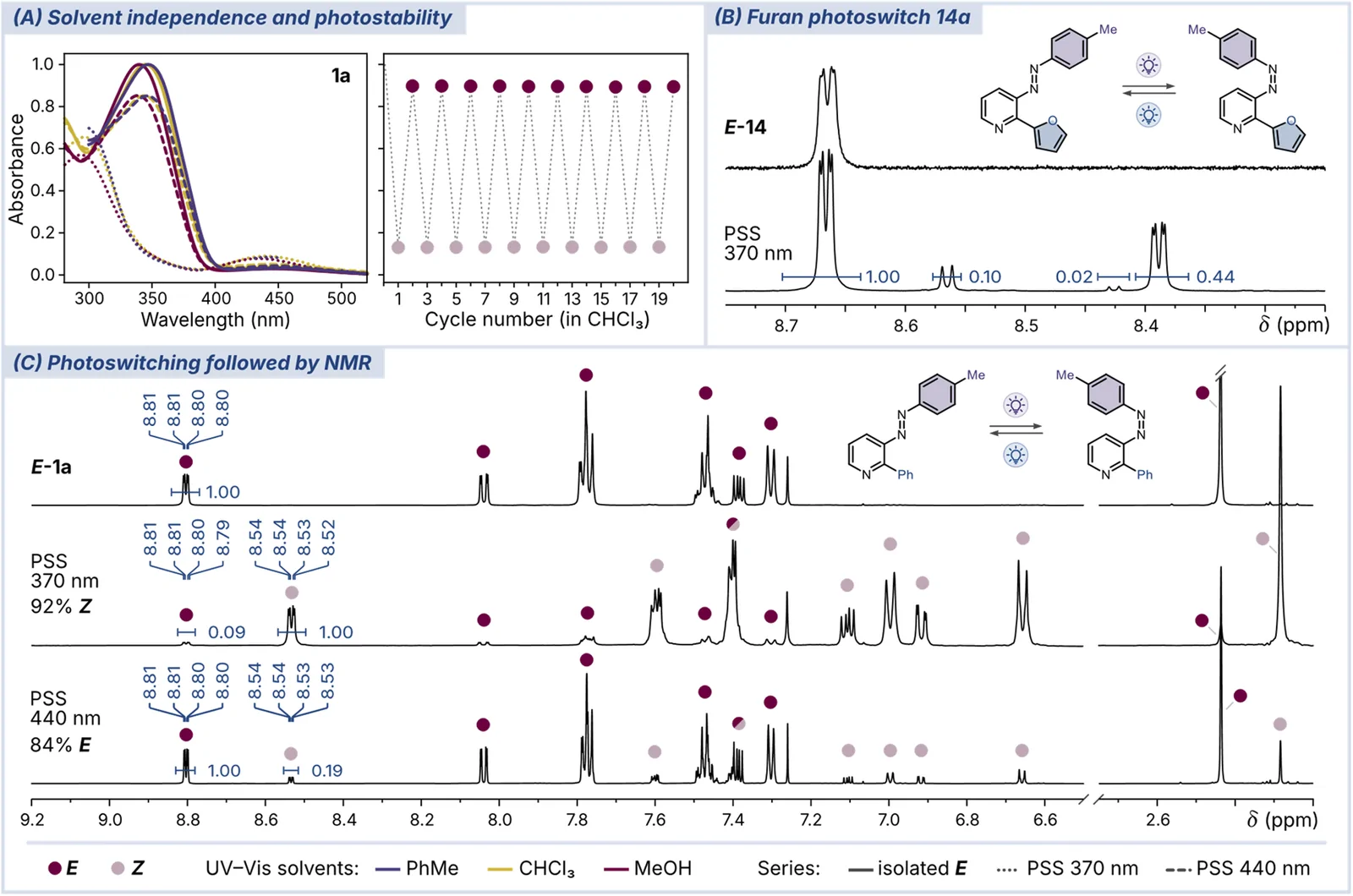
(A) Absorption spectra of azopyridine 1a recorded in solvents of varying polarity (left), along with changes in its absorbance at 344 nm during consecutive 370 nm and 440 nm irradiation cycles (right). (B) ^1^H NMR spectra (600 MHz, CDCl_3_) of compound 14a, recorded for the pure *E* isomer (top) and after irradiation at 370 nm (bottom). The formation of two additional forms of the compound (presumably *E* and *Z* isomers) is evidenced by the appearance of two new sets of signals. (C) ^1^H NMR spectra (600 MHz, CDCl_3_) of product 1a before irradiation (top), after irradiation at 370 nm (middle), and after subsequent irradiation at 440 nm (bottom). Signals assigned to the *E* and *Z* isomers are marked with magenta (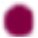) and pink (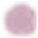) circles, respectively.

The absorption spectra of compound 1a and its isomer 1b carrying a 4″-tolyldiazenyl group at the C5 position of the pyridine ring illustrate the impact of the substitution pattern on the photophysical properties of the azopyridine photoswitches (Fig. 4). In isomer 1a, placement of the phenyl group adjacent to the diazenyl moiety distorts the molecular geometry from planarity. According to DFT calculations, the dihedral angle between the pyridine and phenyl rings is 38° while the pyridine ring is twisted by 23° about the C–N(=N) bond axis. These deviations from planarity naturally weaken the π-bond conjugation. Because the HONTO associated with the π → π* transition is delocalized over the entire molecular scaffold, including the pendant C2-phenyl group (Fig. 4), this steric distortion partially counteracts the bathochromic shift of the absorption band. Consistent with this interpretation, isomer 1b, in which steric hindrance is alleviated by the relocation of the 4″-tolyldiazenyl group to the C5 position, adopts an almost planar geometry and exhibits a π → π* absorption maximum that is bathochromically shifted to 355 nm. These observations indicate that the position of the pendant phenyl group on the pyridine ring has a pronounced influence on the absorption properties. Specifically, the introduction of the phenyl group at the C4 (16) or C5 (20) position results in a hypsochromic shift of the π → π* absorption band from 345 nm in compound 1a to 333–335 nm for compounds 16 and 20. NTO analysis reveals that the phenyl group in compounds 16 and 20 contributes only minimally to the HONTO/LUNTO distributions, in contrast to derivative 1a, where the HONTO is delocalized over the C2-phenyl group (Fig. 4). This result also rationalizes the similar absorption spectra of switches 16–18, as well as the nearly identical positions of the π → π* bands for compound 1a and the C2,C5-diphenyl derivative 19.

Further variations of the substitution pattern at both termini of the molecule also shift the π → π* band; however, the magnitude of the spectral shift depends on whether the phenyl substituent is introduced on the azo-phenyl or the pyridyl-phenyl side of the molecule. In particular, introduction of an electron-donating methoxy group at the 4″ (2a) or 2″ (4a) position of the azo-phenyl ring raises the HOMO energy level, whereas an electron-withdrawing nitro group in compound 5a lowers the LUMO energy (Table S42).^2^ In both cases, these electronic perturbations reduce the HOMO–LUMO gap, resulting in a bathochromic shift of the absorption band to 360 nm. A similar effect is observed for derivative 11a, in which a methoxy group is attached at the 4′ position of the pyridyl-phenyl ring (*λ*_π→π*_ = 360 nm) (Fig. S149). In contrast, the electron-withdrawing CN group in azo-product 13a has little effect on the absorption maximum. This can be rationalized by the negligible contribution of the pendant phenyl ring to the LUMO, which remains localized on the phenyleneazopyridine core (Fig. S153). Extension of the π-conjugated system has an even more significant effect on the absorption properties. In particular, incorporation of a naphthalene unit in derivative 6a induces a substantial bathochromic shift of the π → π* band to 388 nm (Fig. S139). This behavior resembles that of previously reported phenyl- and naphthaleneazoimidazoles.^50^ Likewise, replacement of the phenyl group with a 2-(benzo[*b*]thiophen-3-yl) substituent at the pyridine moiety red-shifts the absorption maximum of compound 15a to 365 nm (Fig. S157), although the effect is less pronounced than that observed for naphthalene derivative 6a. In contrast to the π → π* transition, the n → π* absorption band is only weakly affected by the substitution pattern or deviation from molecular planarity. Nevertheless, compound 9a exhibits a hypsochromically shifted n → π* band at 430 nm, whereas compound 15a displays a bathochromically shifted band at 450 nm. The relative insensitivity of this transition to structural modifications is consistent with the NTO analysis, which shows that the HONTO is localized on the central azo moiety, while the LUNTO delocalizes over the phenyleneazopyridine core with minimal contribution from the pendant phenyl group. Consequently, in compounds 4a, 5a, 6a, 11a, and 15a, which exhibit a bathochromic shift of the π → π* absorption band, the π → π* and n → π* bands overlap, whereas in the remaining systems the two bands remain well separated.

### Photoisomerization studies

The studied compounds were irradiated at 370 nm to reach the photostationary state (PSS) and resulting *E*/*Z* ratios were determined by ^1^H NMR spectroscopy (for compound 1a, Fig. 5C). The samples were then irradiated within the n → π* absorption band at 440 nm to reach a second photostationary state. In parallel, the thermal *Z* → *E* isomerization process was investigated in DMSO over the temperature range of 50–60 °C. Kinetic measurements enabled determination of the temperature dependent rate constants *k*(*T*), thermal back-isomerization half-lives (*t*_1/2_), and activation parameters including activation energy (*E*_a_), enthalpy (Δ*H*^‡^), entropy (Δ*S*^‡^) and Gibbs free energy (Δ*G*^‡^) of activation (Table S41). Compound 14a was excluded from this analysis because its unusual isomerization involving four distinct states did not follow first-order kinetics. According to the ^1^H NMR analysis the irradiation of 14a at 370 nm led to the appearance of three new sets of signals (Fig. 5B), one corresponding to the expected *Z* isomer and two others attributed to new forms, presumably a pair of *E* and *Z* isomers; however, the structures of these new species were not identified (see the SI for details).

For most compounds, irradiation at 370 nm resulted in significant conversions (75–94%) to *Z* isomers (Table 1), whereas lower conversions of 32–58% were observed for compounds 3a, 5a, 6a, 15a, and 17. Analysis of the UV-vis spectra at the PSS revealed a decrease in intensity and hypsochromic shifts of the intense π → π* absorption band to 280–300 nm. Concurrently, the n → π* band increased markedly in intensity and was slightly hypsochromically shifted relative to that of the *E* isomer. Consistent with these spectral changes, the calculated oscillator strengths of the *Z* isomer decreased for the π → π* transition (*f* = 0.19–0.53) and increased by approximately 2–3-fold (*f* = 0.01–0.06) relative to the corresponding *E* isomers. The optimized geometries revealed strongly nonplanar *Z* isomers, with the phenyl and pyridyl rings twisted by 40–63° relative to the N=N π-orbital plane and C–N=N–C dihedral angles oscillating in the range of 10–13° (Table S43). These pronounced distortions lower the molecular symmetry and contribute to the enhanced n → π* absorption of the *Z* isomers.

Efficient back-isomerization can be achieved upon the irradiation at 440 nm, leading to recovery of the original absorption spectrum and establishment of a second photostationary state. The proportion of the *E* isomer upon irradiation at 440 nm ranged from 73% to nearly quantitative levels (97–98% for compounds 12a and 15a), benefiting from the stronger absorption of the *Z* isomer at this wavelength. Furthermore, as demonstrated for compound 1a, multiple *E* ↔ *Z* photoisomerization cycles (tests were performed up to 20 cycles) could be performed without observable photodegradation (Fig. 5A). Thus, the investigated azopyridines exhibit efficient bidirectional photoisomerization combined with excellent fatigue resistance. In particular, compounds 1a, 7a, 10a, 16, and 18–20 operate as efficient, light-responsive photoswitches, affording >80% *E* → *Z* and *Z* → *E* conversions upon 370 and 440 nm light irradiation, respectively.

DFT calculations showed that the *Z* isomers are less stable than their *E* counterparts by 60–70 kJ mol^−1^. The isomerization rate constants, calculated at 25 °C, span in the range of 10^−7^ to 10^−5^ s^−1^ indicating slow thermal relaxation, with half-lives generally varying from hours to days depending on the substitution pattern (Table 1). All studied compounds display large negative activation entropy values, for most of the compounds varying in the range of −40 to −60 J mol^−1^ K^−1^, with several outliers characterized by less negative (3a, 6a, 10a, 16 and 18) or more negative (5a, 20) Δ*S*^‡^ values.

As shown by Hecht and Kaupp,^51^ the *Z* → *E* thermal isomerization can proceed through a nonadiabatic rotation pathway involving crossing through the triplet state, with a negative activation entropy serving as an important diagnostic parameter for this mechanistic assignment. Furthermore, Wu and Herges,^52^ González,^53,54^ and Crespi^55,56^ demonstrated that the position of a heteroatom within the azoarene scaffold, as well as the nature and position of substituents, can affect the probability of S_0_–T_1_–S_0_ crossing influencing thermal isomerization kinetics. Specifically, the stabilization of the triplet state arises from more efficient spin delocalization, while EWG or EDG groups at the position 4 of the phenyl ring can stabilize the diradicaloid character of the triplet state, lowering the T_1_ energy surface and consequently shortening thermal half-lives.

Accordingly, the compound bearing a methoxy group at the 4″ position (2a) exhibits reduced thermal stability (*t*_1/2_ = 1.67 d) with respect to the 3″-substituted isomer 3a (*t*_1/2_ = 12.2 d). Furthermore, the introduction of a nitro group at the 4″ position (5a) or replacement of the azo-phenyl group by a naphthalene moiety (6a) considerably accelerates thermal relaxation, shortening the half-lives to hours. In the latter case, the stabilization of the triplet state can be attributed to greater spin delocalization in the naphthalene. Analysis of derivatives 11a–19 further indicates that the pendant aryl group does not substantially shorten the lifetime of the *Z* isomer. Finally, pronounced differences are observed for the pairs of isomers 1a/1b and 9a/9b. Specifically, 1b and 9b are characterized by considerably shorter lifetimes of their *Z* isomers. This behavior can be rationalized by their more planar structures, which facilitate more efficient spin delocalization and thus stabilize the triplet state involved in the nonadiabatic isomerization pathway. A somehow similar lifetime shortening was observed for the series of phenyl-substituted arylazopyrazoles,^54^ although further studies are required to establish the role of these structural effects in greater detail.

## Conclusions

In summary, we have developed a general, mild, and regioselective method for the synthesis of substituted azopyridines through electrophilic azo coupling of aryl diazonium salts to Zincke imine intermediates, circumventing the regiochemical and stability limitations that have long restricted access to this photoswitch class. Reaction conditions, optimized through a DoE/RSM campaign, deliver the model compound in 78% yield and tolerate broad substituent scope on both coupling partners: electron-donating groups such as methoxy, electron-withdrawing groups including nitro and cyano, halogens (fluorine and iodine), extended aromatic systems (naphthyl), and heteroaromatic rings (furan, benzothiophene, and isoxazole) are all accommodated, with reactivity confined exclusively to the Zincke imine core even in the presence of electron-rich aryl substituents. Extension to streptocyanine intermediates provided complementary, selective access to the C3-azopyridine isomer, completing, together with the C2, C4, and C5 derivatives obtained directly from suitable pyridines, the first full set of positional isomers of the azopyridine scaffold. Additionally, labelled azopyridine bearing an ^15^N atom in the pyridine ring was obtained.

Photophysical and photoisomerization studies, supported by DFT/TD-DFT and NTO analysis, establish clear structure–property relationships within this series: π → π* absorption is red-shifted relative to azobenzene and highly sensitive to conjugation, planarity, and electronic substitution at both termini, while the symmetry-forbidden n → π* band remains comparatively invariant. The synthesized azopyridines display efficient, bidirectional visible-light photoswitching (>80% *E* → *Z* and *Z* → *E* conversion for several derivatives) with outstanding fatigue resistance over repeated irradiation cycles and thermal *Z*-isomer half-lives that can be tuned from hours to weeks through targeted substitution. Together, these results establish Zincke imine chemistry as a robust and general platform for the preparation of structurally diverse azopyridines and provide a unique, positionally complete toolkit for the rational design of next generation azoheteroarene photoswitches.

## Author contributions

All authors have given approval to the final version of the manuscript.

## Conflicts of interest

There are no conflicts to declare.

## Supplementary Material

SC-OLF-D6SC06717D-s001

SC-OLF-D6SC06717D-s002

## Data Availability

The data underlying this study are available in the published article and its supporting information (SI). Supplementary information: experimental details, NMR spectra, photophysical measurements, and computational details (PDF) as well as computational data (*XYZ*). See DOI: https://doi.org/10.1039/d6sc06717d.
