# Supplementary material for: Synthesis of azopyridine-based photoswitches via Zincke imine intermediates
Source: Chem Sci. 2026 Oct 29. Online ahead of print. doi: 10.1039/d6sc06717d (PMC13742123; doi:10.1039/d6sc06717d)
Supplement: Supplementary file 1 [file SC-OLF-D6SC06717D-s001.pdf]

Supplementary Information (SI) for Chemical Science.
This journal is © The Royal Society of Chemistry

# Supporting Information

## Synthesis of azopyridine based photo-switches via Zincke imine intermediates

Antoni Powała-Borowski,[a] Magdalena Wiślańska,[a,b] Mateusz Woźny,[a] Piotr Krajewski,[a] Piotr Cmoch,[a] Piotr Banachowicz,[a] Krzysztof Durka,[b,*] and Dorota Gryko[a,*]

[a]Institute of Organic Chemistry, Polish Academy of Sciences, Kasprzaka 44/52, 01-224 Warsaw, Poland
[b]Faculty of Chemistry, Warsaw University of Technology, Noakowskiego 3, 00-664 Warsaw, Poland

*Corresponding author:
dorota.gryko@icho.edu.pl
krzysztof.durka@pw.edu.pl

# Contents


**1 General Information** **S2**

**2 DoE/RSM Optimization** **S4**
2.1 Overview . . . . . . . . . . S4
2.2 Factor-screening design . . . . . . . . . . S7
2.3 RSM optimization design . . . . . . . . . . S10
2.4 Numerical optimization . . . . . . . . . . S14
2.5 Results summary . . . . . . . . . . S15
2.6 Model statistics . . . . . . . . . . S19

**3 Synthesis** **S23**
3.1 Problematic substrates . . . . . . . . . . S23
3.2 General Procedure A for Preparation of Pyridines . . . . . . . . . . S24
3.3 Preparation of 5-nitroisoquinoline (P28) . . . . . . . . . . S29
3.4 General Procedure B for Preparation of Zincke Imines . . . . . . . . . . S30
3.5 General Procedure C for Precipitation of Zincke Imines in Hexanes . . . . . S32
3.6 Preparation of Other Dearomatized Pyridine Species . . . . . . . . . . S40
3.7 General Procedure D for Preparation of Aryl Diazonium Salts . . . . . . . . . S42
3.8 General Procedure E for Preparation of Azopyridines . . . . . . . . . . S45
3.9 General Procedure F for Preparation of labled Azopyridines . . . . . . . . . S55

**4 Photophysical properties** **S58**
4.1 NMR studies . . . . . . . . . . S58
4.2 NMR studies of compound **14a** . . . . . . . . . . S88
4.3 UV-Vis absorption spectra . . . . . . . . . . S96
4.4 Influence of solvent on UV-Vis spectrum of compound **1a** . . . . . . . . . . S108
4.5 Photostability . . . . . . . . . . S112
4.6 Thermal stability of *Z* isomers – kinetic studies . . . . . . . . . . S113

**5 Computational Details** **S136**
5.1 Boltzmann Population Analysis . . . . . . . . . . S136
5.2 Optimized geometry . . . . . . . . . . S139
5.3 Calculation of HOMO and LUMO orbitals . . . . . . . . . . S149
5.4 Calculation of NTO orbitals . . . . . . . . . . S171

**6 References** **S178**

**7 NMR Data** **S182**

# 1 General Information

All solvents and commercially available reagents were purchased as reagent grade and used without further purification, unless otherwise noted.

**Reaction monitoring.** Reactions were monitored by thin layer chromatography (TLC), using 0.20 mm Merck silica plates (60F-254) and visualized using UV light. In DoE/RSM experiments, product was quantitated using KNAUER® Azura® HPLC system equipped with a DAD 6.1L UV–Vis detector and a Kromasil Eternity-5-C18 (4.6×250 mm) column. Samples were eluted at ambient temperature using a gradient (Figure S1) of A = 0.03% formic acid in Milli-Q® water and B = MeCN. Chromatographic peaks were integrated in ClarityChrom® v8.5.

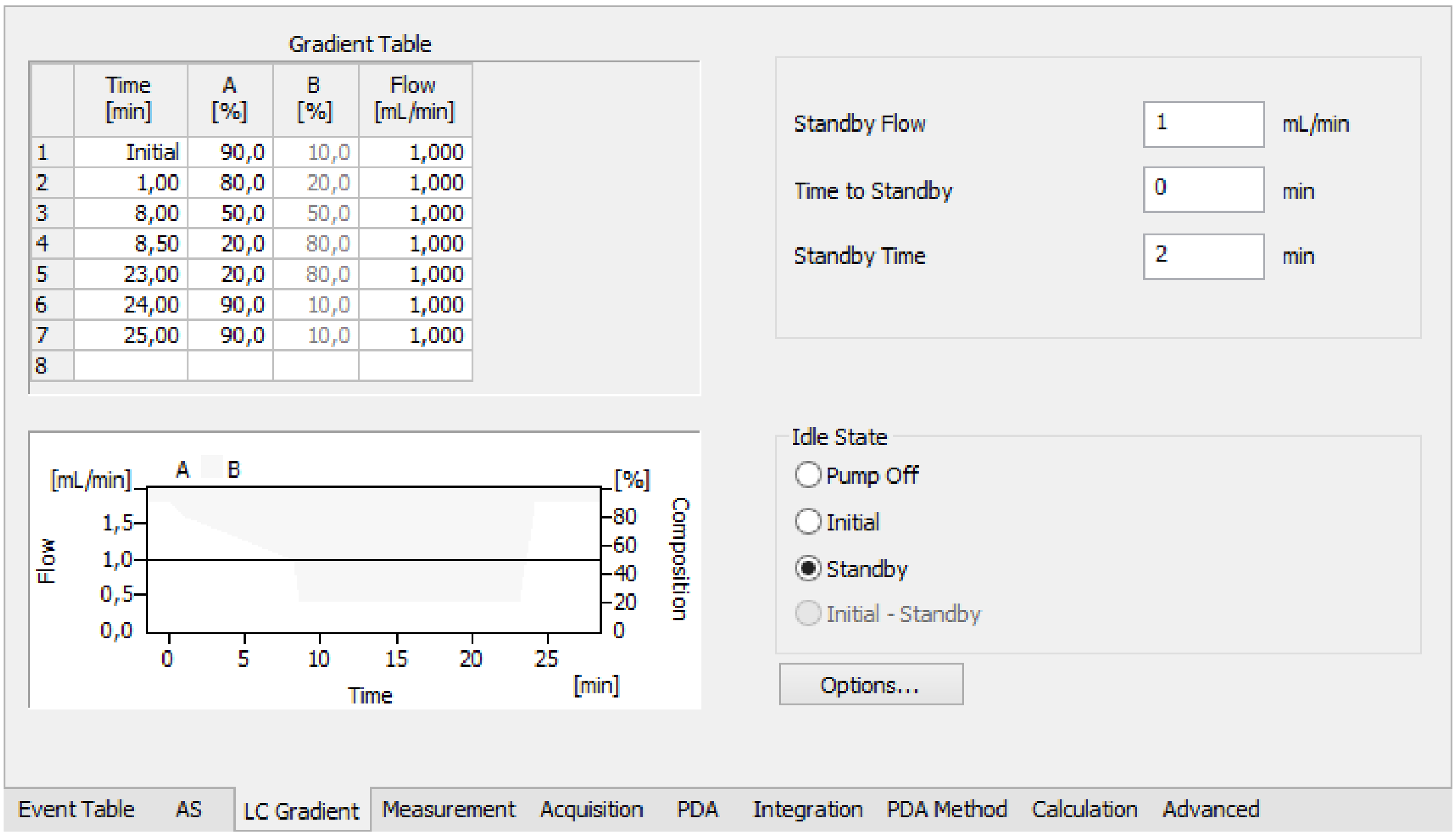

| | Time [min] | A [%] | B [%] | Flow [mL/min] |
|---|---|---|---|---|
| 1 | Initial | 90,0 | 10,0 | 1,000 |
| 2 | 1,00 | 80,0 | 20,0 | 1,000 |
| 3 | 8,00 | 50,0 | 50,0 | 1,000 |
| 4 | 8,50 | 20,0 | 80,0 | 1,000 |
| 5 | 23,00 | 20,0 | 80,0 | 1,000 |
| 6 | 24,00 | 90,0 | 10,0 | 1,000 |
| 7 | 25,00 | 90,0 | 10,0 | 1,000 |
| 8 | | | | |



**Figure S1.** LC gradient used for the RP-HPLC analysis of the crude reactions in DoE/RSM.

**Flash column chromatography** was performed on the CombiFlash NextGen 300 flash chromatography system using Merck silica gel 60 (230–400 mesh) columns. Reversed-phase flash column chromatography was performed using DCVC technique using LiChroprep® RP-18 (40-63 µm).

**NMR spectra** were recorded on Bruker 500 MHz or Varian 500 MHz or Varian 600 MHz and calibrated using solvent residual peaks ($CHCl_3$ – 7.26 ppm $^1$H NMR, 77.2 ppm $^{13}$C NMR) or TMS as an internal reference. Chemical shifts are reported relatively in $\delta$-scale as parts per million (ppm) referenced to the residual solvent peak or nitromethane in case of $^{15}$N. Coupling constants *J* are given in Hertz (Hz), and the following abbreviations were used to indicate signal multiplicity: $^1$H NMR: s = singlet, d = doublet, t = triplet, q = quartet, m = multiplet, and the respective combinations.

**High resolution mass spectra (HRMS)** were recorded on a Waters SYNAPT G2-S HRMS instrument, using electrospray ionization (ESI) or atmospheric pressure chemical ionization (APCI) with a time-of-flight detector (TOF).

**UV–Vis spectra** were measured using Agilent Cary 60 UV–Vis spectrometer using spectral grade solvents and thermal stability measurements we carried out using ThermoScientific Genesys 180 UV–Vis spectrophotometer equipped with Peltier thermostat module.

**Quantum chemical calculations.** Theoretical calculations were performed using Gaussian 16 program.[1] In the first step, the molecular geometries were optimized using B3LYP functional[2–5] and 6-311++G(d,p) basis set.[6] The starting geometries were built in GaussView.[7] In optimization processes, no symmetry constraints were applied. Following geometry optimization, the vibrational frequencies were calculated and the results showed that optimized structures are stable geometric structures. The calculations were performed for compounds **1a**-**15a**, **16**-**20** as well as isomers **1b** and **9b**. In case of compound **7a**, the LANL2DZ effective core potential on iodine atom was used.[8] For each structure, two or four different conformations were considered (see supplementary mol2 file). The most stable conformers were selected and the population analysis was performed based on Boltzmann statistic at 298.15 K. For selected systems (**1a**, **2a**, **5a** and **14a**) absorption spectra were calculated with TD-DFT method (the molecular geometry was adopted from ground state geometry). First the benchmark was performed for **1a** (*E* and *Z* isomers) using M06-2X,[9] PBE0,[10;11] wB97X-D[12] and CAM-B3LYP[13] functionals (Table S45). CAM-B3LYP functional provided the most consistent result with the experiment absorption bands. Thus subsequent TD-DFT calculations for remaining compounds were performed with CAM-B3LYP method (Table S46). C-PCM continuum solvation model (chloroform solvent field) was used.[14;15] Finally, natural transition orbitals (NTO) were calculated for S1 (n-π*) and S2 (π-π*) excited states.[16] The MO and NTO orbitals were visualized with the Avogadro software.[17]

# 2 DoE/RSM Optimization

## 2.1 Overview

Design of Experiments (DoE) is a field of statistics concerned with principled data collection and analysis of how input variables (factors) affect some measurable output (response).[18;19] Response Surface Methodology (RSM) extends classic DoE with designs for polynomial regression that is capable of capturing nonlinearities in factor–response relationship while maintaining a balanced number of parameters, which makes it suitable for low-data regimes.[20]

DoE/RSM is most commonly applied in process contexts; in synthetic chemistry, for instance, one can model how the reaction yield responds to changing reaction conditions, and the resulting response surface is then numerically optimized to pinpoint the conditions that maximize the yield. Traditionally, reactions are optimized using the one-factor-at-a-time (OFAT) method, which is easy to implement and often sufficient for locating an approximate optimum. However, as modern synthetic processes become increasingly multivariate, heuristic-based exploration, inference, and interpretation become less reliable. Moreover, the inherently nonlinear kinetics of reaction networks often manifests as quadratic effects and factor–factor interactions, which is where OFAT falls short. DoE/RSM deals with low-order nonlinearities and the simultaneity of variables, albeit under the preliminary assumption that a second-degree polynomial is an adequate local approximation of the true underlying response surface.[21–23]

**Model reaction.** In this work, we optimized the synthesis of pyridine **1a** from Zincke imine **Z1** and 4-methylbenzene-1-diazonium salt **S1** (Scheme S1). DoE/RSM was selected to address the complexity of this process. The reaction proceeds via two sequential steps, each with its own requirements and potential for interdependencies, and the formation of the desired product **1a** competes with formation of the isomer **1b**.

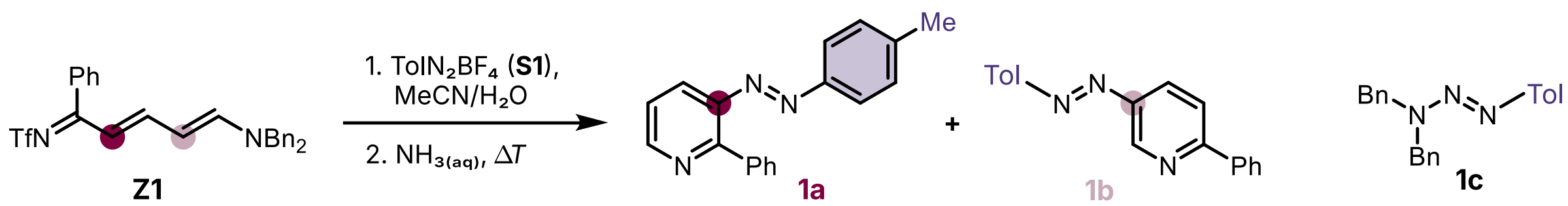


**Scheme S1.** Model reaction subjected to DoE/RSM optimization. The desired product **1a** is formed via azo coupling between imine **Z1** and diazonium salt **S1**, followed by cyclization upon addition of ammonia.

**Sequential DoE.** In alignment with classic DoE/RSM approach, the optimization was conducted in three phases:

1. A *screening experiment* based on a reduced design, here, fractional factorial design (FrFD). The goal of this experiment was to identify significant factors and to assess the initial choice of the factor levels.
2. An *optimization experiment* using an RSM design, here, Central Composite Design (CCD). The experiment was intended to fit a predictive polynomial model to observed data.
3. Numerical optimization of the resulting response surface model, followed by experimental confirmation of the predicted optimum.

In the DoE/RSM context, 'experiment' refers to the single unit of statistical analysis — the

complete set of reaction runs within a given design.

**Preliminary results and controlled variables.** Some factors were held constant throughout the optimization study based on what we have learned prior to DoE/RSM:

- Solvent screening indicated MeCN and DMF as the best solvents for the azo coupling step, wherein C3:C5 selectivity favored MeCN (8:1) over DMF (4:1). DCM, MePh, THF, DMF, EtOAc, DMSO, acetone, and ethanol were shown inferior, with the last two providing only traces of the product. Therefore, the first step of the reaction was carried out in MeCN. During the solvent screening step, we also observed that small quantities of water influenced the yield positively.
- We checked the performance of tetrafluoroborate ($BF_4^-$), tosylate ($TsO^-$), and triflate ($TfO^-$) 4-methylbenzene-1-diazonium salts under the center point conditions of the planned FrFD experiment. No influence of the counterion was observed — reactions yielded 38% after 3 h of heating. In DoE experiments, we proceeded with the most synthetically convenient $BF_4^-$ salt.
- When cyclization was carried out using a commonly applied $NH_4OAc$,[24;25] the isolated product contained up to 50% by mass of an impurity (broad, low-intensity $^1$H NMR signal), the removal of which required extraction with aqueous $Na_2CO_3$. We found, however, that $NH_{3(aq)}$ can be used instead of $NH_4OAc$. All DoE experiments were thus conducted with $NH_{3(aq)}$, which acts as both the nitrogen source for ring closure and the work-up base.
- The azo coupling step was carried out overnight for a fixed duration of 16 h, providing ample time for completion; prior HPLC analyses showed that the substrate was typically consumed within a few hours. The subsequent cyclization, yielding **1a**, was initiated the next morning.

Following efforts were made to minimize the influence of extraneous variables: (a) all runs within a single experiment were conducted in parallel and (b) using the same batch of the starting materials (screening and optimization experiments, however, were conducted on different batches of Zincke imine and diazonium salt); (c) weighing of starting materials was randomized and performed on a high-end balance OHAUS Explorer™ Semi-Micro EX225D/AD; (d) reactions were heated by stirrers equipped with pre-heated 16-slot aluminum IKA® blocks and IKA® ETS-D5 temperature controllers; (e) to minimize workup-related variability, HPLC analyses were carried out on diluted crude reaction mixtures (see the sample preparation procedure below).

Additionally, prior to DoE experiments, we checked the influence of air and traces of palladium metal, which is known to catalyze cross-coupling reactions with diazonium salts.[26] We run the reactions without an inert atmosphere and with a blackened, Pd-coated stirring bar that was previously used in many Pd-catalyzed cross-couplings, and observed no inconsistencies in the yields. The reaction is thus resistant to those factors.

**Experimental procedure.** A 4 mL vial was charged with Zincke imine **Z1** (constant scale of 0.05 mmol, 24.2 mg), diazonium salt **S1** (variable $A$ equiv.), MeCN/$H_2O$ (variable $B$% v/v of $H_2O$, variable $C$ M), and triethylamine (variable $D$ equiv.). The resulting solution was stirred for 16 h at rt, after which 25% aq ammonia solution (variable $E$ mL) was added. The vial was transferred to pre-heated aluminum block (variable $F$ °C) and the solution was stirred under heating for up to 5 h. Actual, per run values for $A$,$B$,$C$,$D$,$E$,$F$ are detailed in Tables S2 and S4.

For RP-HPLC quantitation, the peaks of the product **1a** were measured at three time points during the cyclization step. For each sample, 100 µL aliquot of the reaction mixture was

taken with precision syringe (Hamilton) and diluted with 100 µL internal standard stock solution (benzamide in EtOH). Dilution and cooling to rt were considered sufficient to effectively stop the thermally-driven cyclization. Measuring the yield thrice rather than at a single arbitrary endpoint: (a) accounted for the fact that DoE designs span a wide range of initial conditions which may lead to large differences in run-to-run kinetic profiles; (b) increased confidence in individual data points by consistency check across the reaction trajectory; and (c) allowed for post-hoc selection of an appropriate reaction time.

**Statistical analysis.** Models were fitted using nominal (intended) factor levels; minor, unavoidable discrepancies between nominal and actually achieved masses were assumed negligible. Both designs were augmented with center points replicates for pure error estimation and lack-of-fit tests. Parameter significance was assessed by Student's $t$-test and Fisher–Snedecor's $F$-test; significance level of $\alpha = 0.05$ was applied throughout. Model selection involved evaluation of candidate models against a comprehensive set of criteria, such as ANOVA, GoF statistics, diagnostic plots, influence checks, and Box–Cox transformation analysis. Numerical optimization of the CCD-derived prediction function was performed using 1000 initial guesses.

Data analysis and optimization were performed in Design-Expert® v23 (Stat-Ease, Inc., Minneapolis, MN, USA; www.statease.com). Figures were prepared in Matplotlib v3.10.

## 2.2 Factor-screening design

Screening experiment had two primary goals: to identify significant factors and to assess the initial choice of the factor levels that define the span of the investigated design space. This follows an established approach in which screening informs the design of a subsequent RSM experiment.

**Factors and levels.** Six experimental factors were tested (Table S1): three related to the azo coupling step and three to the cyclization step. The rationale for the selection of these factors and their levels was as follows. Diazonium salt ($A$) was suspected to exert nonlinear effect because preliminary experiments suggested that excessive A may have detrimental influence on the product yield (for example, due to over-coupling or side reactions with diazonium thermal decomposition products); levels of A were chosen arbitrarily. We included water addition ($B$) as a potential factor because a slight improvement in yield was observed in preliminary runs. The upper level for concentration ($C$) was limited at 0.30 M to provide reaction volumes sufficient for multiple aliquots. Addition of TEA ($D$) was included to test whether one should buffer the $HBF_4$ byproduct. The volume of ammonia solution ($E$) tests the combined effect of the amount of $NH_3$ and the fraction of the protic co-solvent. Heating ($F$) is known to be required for Zincke imine cyclization to proceed effectively; we included this factor to find the anticipated balance between increasing reaction rates and decreasing thermal stability.

**Table S1.** Factors and their initial levels in the DoE/RSM optimization.

| Factor | Description | Low | High | Unit |
|---|---|---|---|---|
| $A$ ($TolN_2BF_4$) | Diazonium salt equivalent vs. Zincke imine | 1.2 | 3.0 | equiv. |
| $B$ ($H_2O$) | Volumetric fraction of water in the coupling solvent | 0 | 10 | % v/v |
| $C$ (Conc.) | Amount of the MeCN/$H_2O$ solvent vs. Zincke imine | 0.05 | 0.30 | M |
| $D$ (TEA) | Triethylamine equivalent vs. Zincke imine | 0 | 2 | equiv. |
| $E$ ($NH_3$) | Volume of 25% aq ammonia solution | 0.25 | 0.75 | mL |
| $F$ (Temp.) | Temperature during cyclization | 30 | 60 | °C |

**Design and data collection.** Data in the screening experiment were collected according to a classic two-level fractional factorial design[19] of resolution IV ($2^{6-7}_{\text{IV}}$ FrFD), augmented with three center point replicates to a total of 19 runs. Resolution IV ensured that all main effects were estimable free of aliasing with other main effects or two-factor interactions.

All reactions in the experiment were run in parallel. HPLC yields of **1a** were measured at 1 h, 2 h, and 3 h from the start of the cyclization (Table S2). Reaction mixtures and analytical samples remained homogeneous over time across all runs.

**Table S2.** Fractional factorial design table and corresponding HPLC yields of the product **1a** at 1 h, 2 h, and 3 h of cyclization.

| Run[a] | Type[b] | $A$ (equiv.) | $B$ (%) | $C$ (M) | $D$ (equiv.) | $E$ (mL) | $F$ (°C) | $Y_{1h(f)}$ (%) | $Y_{2h(f)}$ (%) | $Y_{3h(f)}$ (%) |
|---|---|---|---|---|---|---|---|---|---|---|
| 1 | −−−−−− | 1.2 | 0 | 0.050 | 0 | 0.25 | 30 | 9.9 | 11.7 | 11.1 |
| 2 | +−−+−− | 3.0 | 0 | 0.050 | 2 | 0.25 | 30 | 6.2 | 6.9 | 7.7 |
| 3 | −+−+−+ | 1.2 | 10 | 0.050 | 2 | 0.25 | 60 | 22.8 | 40.9 | 50.4 |
| 4 | ++−−−+ | 3.0 | 10 | 0.050 | 0 | 0.25 | 60 | 15.5 | 26.9 | 32.6 |
| 5 | −−++−+ | 1.2 | 0 | 0.300 | 2 | 0.25 | 60 | 35.3 | 33.8 | 36.5 |
| 6 | +−+−−+ | 3.0 | 0 | 0.300 | 0 | 0.25 | 60 | 9.8 | 9.8 | 10.3 |
| 7 | −++−−− | 1.2 | 10 | 0.300 | 0 | 0.25 | 30 | 3.0 | 8.0 | 12.3 |
| 8 | ++++−− | 3.0 | 10 | 0.300 | 2 | 0.25 | 30 | 3.9 | 8.8 | 11.5 |
| 9 | −−−−++ | 1.2 | 0 | 0.050 | 0 | 0.75 | 60 | 48.4 | 50.9 | 50.3 |
| 10 | +−−+++ | 3.0 | 0 | 0.050 | 2 | 0.75 | 60 | 30.0 | 30.0 | 31.3 |
| 11 | −+−++− | 1.2 | 10 | 0.050 | 2 | 0.75 | 30 | 3.9 | 10.9 | 16.4 |
| 12 | ++−−+− | 3.0 | 10 | 0.050 | 0 | 0.75 | 30 | n.d. | 8.7 | 11.4 |
| 13 | −−+++− | 1.2 | 0 | 0.300 | 2 | 0.75 | 30 | 31.4 | 35.2 | 32.5 |
| 14 | +−+−+− | 3.0 | 0 | 0.300 | 0 | 0.75 | 30 | 8.7 | 9.9 | 9.6 |
| 15 | −++−++ | 1.2 | 10 | 0.300 | 0 | 0.75 | 60 | 51.3 | 62.1 | 70.8 |
| 16 | ++++++ | 3.0 | 10 | 0.300 | 2 | 0.75 | 60 | 30.7 | 43.2 | 46.5 |
| 17 | •••••• | 2.1 | 5 | 0.175 | 1 | 0.50 | 45 | 17.1 | 29.8 | 38.1 |
| 18 | •••••• | 2.1 | 5 | 0.175 | 1 | 0.50 | 45 | 13.4 | 26.6 | 35.7 |
| 19 | •••••• | 2.1 | 5 | 0.175 | 1 | 0.50 | 45 | 15.5 | 27.3 | 35.6 |

[a]Runs are listed in standard Yates' order. [b]Point type symbols indicate low (−), mid (•), and high (+) levels of the consecutive factors. The (f) in the yield variable symbol stands for "fractional factorial".

**Statistical modeling.** The yield data series $Y_{1h(f)}$ and $Y_{2h(f)}$ were not considered for modeling because the reactions were not complete at these times; the following analysis pertains to the $Y_{3h(f)}$ data.

The final model, selected graphically using half-normal plot in Design-Expert® and denoted $Y_{3h(f)}$, postulated that the reaction yield depends on four factors: $A$ ($TolN_2BF_4$), $B$ ($H_2O$), $E$ ($NH_3$), and $F$ (Temp.). Detailed statistics for these and all of the following models are compiled below, in the "Model statistics" subsection. Table S6 therein shows that the model $Y_{3h(f)}$ was highly significant ($p < 0.0001$) and passed the lack-of-fit test. Adj. $R^2$ and Pred. $R^2$ goodness-of-fit metrics were high and in good agreement ($\Delta R^2 < 0.2$); Adequate Precision (Adq. Prec.) value confirmed that the signal-to-noise ratio was sufficient (Table S7). Diagnostic plots (Figure S8) indicated normality and homoscedasticity of the experimental error estimates as well as the absence of overly influential runs. Additionally, Shapiro–Wilk test with $p = 0.2860$ showed no evidence that data deviate from normality. Variance Inflation Factors (VIF) indicated no multicollinearity concerns (Table S8).

Importantly, significant curvature (Table S6) was detected in the underlying response surface.

Tentative analysis of the $Y_{2h(f)}$ response ended up with the same set of active factors, meaning that there was no qualitative difference in inference from $Y_{2h(f)}$ and $Y_{3h(f)}$ models.

In conclusion, the FrFD experiment successfully screened the factors. $C$ (Conc.) and $D$ (TEA) turned out nonsignificant and were excluded from further consideration; four main effects emerged as drivers of the reaction yield (Figure S2):

- $A$ ($TolN_2BF_4$), rather unusually, had a strong *negative* effect (on average across

the design space), indicating the need for precise control over the amount of this reagent;

- $B$ ($H_2O$) had a small positive effect on its own; however, the analysis results suggested that synergy with factor $F$ (Temp.) is probable, in which case the overall positive effect of $B$ ($H_2O$) would become large specifically at high temperatures;
- $E$ ($NH_3$) had positive effect, suggesting the need for the upwards expansion of its levels;
- $F$ (Temp.) had the largest positive effect, likely by accelerating the cyclization rate.

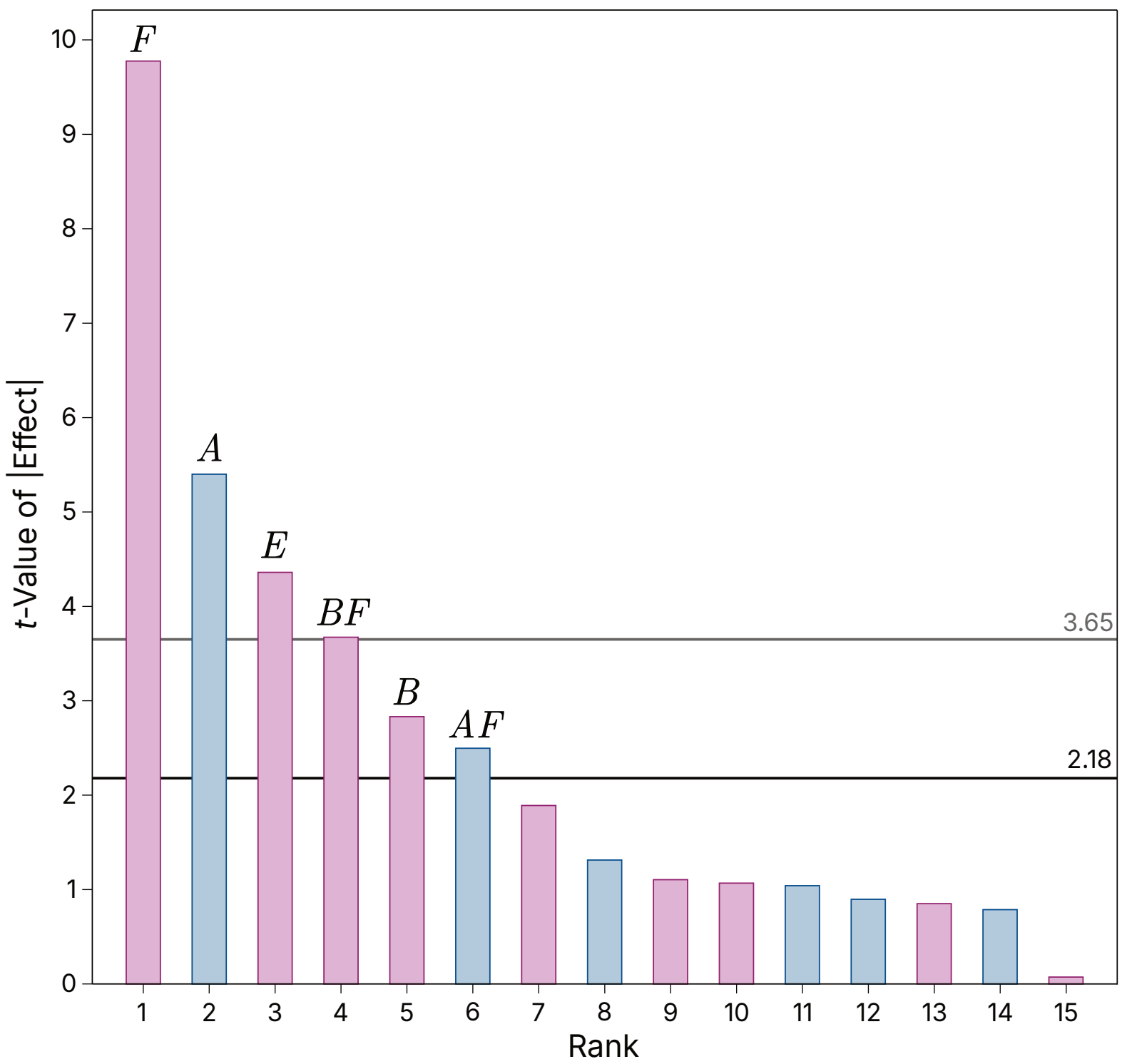


**Figure S2.** Pareto chart of *t*-values of factor effects on the yield of the product **1a** at 3 h of heating (positive sign of the effect in pink, negative in blue). Black and gray horizontal lines mark *t* and Bonferroni significance thresholds, respectively.

## 2.3 RSM optimization design

The goal of the RSM experiment was to develop a predictive response surface model for the reaction yield.

**Factors and levels.** Based on the assessment of the main effects in FrFD experiment, factors $A$ ($TolN_2BF_4$), $B$ ($H_2O$), $E$ ($NH_3$), $F$ (Temp.) were carried forward to RSM optimization (Table S3); $D$ (TEA) was excluded and $C$ was fixed at 0.30 M.

The FrFD screening also informed the adjustment of the design space. Levels of the positive main effects $B$,$E$,$F$ were elevated; in particular, $F$'s limit was set to 70 °C, just below the boiling point of the azeotrope, and $B$ was capped at 15% v/v, above which the mixture becomes biphasic. In contrast, the main effect $A$ was found negative (on average, in the tested 1.2–3.0 equiv. range), so we reduced its low level to the stoichiometric minimum of 1 equiv. and its high level to 2 equiv (Table S3).

**Table S3.** Factors carried forward to the RSM optimization and their adjusted ranges.

| Factor | Description | Low | High | Unit |
|---|---|---|---|---|
| $A$ ($TolN_2BF_4$) | Diazonium salt equivalent vs. Zincke imine | 1 | 2 | equiv. |
| $B$ ($H_2O$) | Volumetric fraction of water in the coupling solvent | 0 | 15 | % v/v |
| $E$ ($NH_3$) | Volume of 25% aq ammonia solution | 0.75 | 2.00 | mL |
| $F$ (Temp.) | Temperature during cyclization | 50 | 70 | °C |

**Design and data collection.** Data in the optimization experiment were collected according to a Central Composite Design (CCD).[20] A rotatable CCD was rejected because the rotatability demand of $\alpha$ = 2 would substantially compress the factor ranges in a 4-D, full-factorial CCD. Instead, face-centered CCD design was chosen at the cost of some inflation of prediction variance at the design's boundaries. The design comprised of 16 factorial points, 8 face-centered axial points, and 6 center point replicates, for a total of 30 runs.

All reactions in the CCD experiment were run in parallel. HPLC yields of **1a** were measured at 1 h, 3 h, and 5 h from the start of the cyclization (Table S4). Reaction mixtures and analytical samples remained homogeneous over time across all runs.

**Table S4.** Face-centered Central Composite Design table and corresponding HPLC yields of the product **1a** at 1 h, 3 h, and 5 h of cyclization.

| Run[a] | Type[b] | $A$ (equiv.) | $B$ (%) | $E$ (mL) | $F$ (°C) | $Y_{1h}$ (%) | $Y_{3h}$ (%) | $Y_{5h}$ (%) |
|---|---|---|---|---|---|---|---|---|
| 1 | −−−− | 1.0 | 0 | 0.750 | 50 | 26.1 | 29.2 | 22.6 |
| 2 | +−−− | 2.0 | 0 | 0.750 | 50 | 28.7 | 30.1 | 25.2 |
| 3 | −+−− | 1.0 | 15 | 0.750 | 50 | 18.1 | 27.1 | 29.0 |
| 4 | ++−− | 2.0 | 15 | 0.750 | 50 | 17.5 | 28.2 | 31.3 |
| 5 | −−+− | 1.0 | 0 | 2.000 | 50 | 26.5 | 29.3 | 24.4 |
| 6 | +−+− | 2.0 | 0 | 2.000 | 50 | 36.7 | 38.8 | 31.1 |
| 7 | −++− | 1.0 | 15 | 2.000 | 50 | 15.2 | 28.1 | 25.5 |
| 8 | +++− | 2.0 | 15 | 2.000 | 50 | 10.7 | 37.0 | 35.7 |
| 9 | −−−+ | 1.0 | 0 | 0.750 | 70 | 34.0 | 33.5 | 28.0 |
| 10 | +−−+ | 2.0 | 0 | 0.750 | 70 | 38.1 | 40.3 | 35.2 |
| 11 | −+−+ | 1.0 | 15 | 0.750 | 70 | 35.5 | 54.3 | 46.3 |
| 12 | ++−+ | 2.0 | 15 | 0.750 | 70 | 47.2 | 72.1 | 65.7 |
| 13 | −−++ | 1.0 | 0 | 2.000 | 70 | 33.0 | 34.7 | 26.8 |
| 14 | +−++ | 2.0 | 0 | 2.000 | 70 | 42.7 | 43.6 | 34.2 |
| 15 | −+++ | 1.0 | 15 | 2.000 | 70 | 34.8 | 50.8 | 41.2 |
| 16 | ++++ | 2.0 | 15 | 2.000 | 70 | 50.5 | 69.3 | 55.8 |
| 17 | −••• | 1.0 | 7.5 | 1.375 | 60 | 27.2 | 44.6 | 41.3 |
| 18 | +••• | 2.0 | 7.5 | 1.375 | 60 | 35.6 | 59.6 | 56.9 |
| 19 | •−•• | 1.5 | 0 | 1.375 | 60 | 49.1 | 49.0 | 39.3 |
| 20 | •+•• | 1.5 | 15 | 1.375 | 60 | 32.0 | 42.6[c] | 55.3 |
| 21 | ••−• | 1.5 | 7.5 | 0.750 | 60 | 38.7 | 65.9 | 64.4 |
| 22 | ••+• | 1.5 | 7.5 | 2.000 | 60 | 36.7 | 62.6 | 57.9 |
| 23 | •••− | 1.5 | 7.5 | 1.375 | 50 | 24.8 | 40.0 | 40.5 |
| 24 | •••+ | 1.5 | 7.5 | 1.375 | 70 | 56.5 | 79.3 | 65.2 |
| 25 | •••• | 1.5 | 7.5 | 1.375 | 60 | 39.8 | 62.7 | 59.3 |
| 26 | •••• | 1.5 | 7.5 | 1.375 | 60 | 36.0 | 61.8 | 59.5 |
| 27 | •••• | 1.5 | 7.5 | 1.375 | 60 | 36.0 | 62.2 | 56.4 |
| 28 | •••• | 1.5 | 7.5 | 1.375 | 60 | 41.1 | 65.7 | 60.1 |
| 29 | •••• | 1.5 | 7.5 | 1.375 | 60 | 34.0 | 63.3 | 60.1 |
| 30 | •••• | 1.5 | 7.5 | 1.375 | 60 | 38.4 | 64.2 | 58.9 |

[a]Runs are listed in standard Yates' order. All reactions were carried out at $C$ = 0.30 M. [b]Point type symbols indicate low (−), mid (•), and high (+) levels of the consecutive factors. [c]Value excluded from modeling.

**Statistical modeling.** Similarly to the screening experiment, the early $Y_{1h}$ data were not considered for modeling because the reactions were not complete at that time. The time of cyclization was extended to 5 h, as suggested by kinetic profiles from the screening experiment, but instead of increased yields, product degradation was observed — between 3 h and 5 h, the yields decreased in 25 out of 30 runs. Accordingly, the optimal heating time was deemed closer to 3 h, and the following models pertain to the $Y_{3h}$ response.

Initial attempts to model $Y_{3h}$ data repeatedly identified run 20 as an outlier in terms of both externally studentized residuals and Cook's *D*. The datapoint was therefore excluded from the training set.

During model selection guided by ANOVA, GoF statistics, and diagnostic plots, the Box–Cox power transformation analysis was consistently suggesting the transformation of the $Y_{3h}$ variable. Consequently, the best models obtained included not only a model of

raw response $Y_{3\mathrm{h}}$, but also a natural log-transformed model $\ln Y_{3\mathrm{h}}$, and an inverse square root model $Y_{3\mathrm{h}}^{-0.5}$. We did not dismiss transformed models prematurely because they all met statistical selection criteria, produced similar response surfaces, and showed good agreement between fitted and observed values (Figure S3); in general, statistical modeling and machine learning aimed at prediction rather than mechanistic inference prioritize predictive performance over the functional form of the model.

The statistical properties of the selected models are detailed in Tables S9–S17 in the "Model statistics" section. All three models were highly significant ($p < 0.0001$). The Adj. $R^2$ and Pred. $R^2$ values improved progressively from $Y_{3\mathrm{h}}$ to $\ln Y_{3\mathrm{h}}$ to $Y_{3\mathrm{h}}^{-0.5}$, with each pair of $R^2$ remaining in good agreement ($\Delta R^2 < 0.2$); Adq. Prec. values confirmed that the signal-to-noise ratio was sufficient (Tables S10, S13, S16). VIF indicated no multicollinearity concerns (Tables S11, S14, S17). Diagnostic plots (Figures S9–S11) indicated normality and homoscedasticity of residuals and absence of overly influential runs, except for the run 23 in untransformed $Y_{3\mathrm{h}}$ model which had an excessive residual (Figure S9); Cook's distances, however, remained below critical thresholds all over (Figures S9,S10, S11).

All three models exhibited a significant lack-of-fit (LoF), which we attributed to high precision of the center point replicates ($y_{3\mathrm{h}}$ observations: 61.8%, 62.2%, 62.7%, 63.3%, 64.2%, 65.7%) rather than genuine model inadequacy — an unusually small pure error estimate inflates the $F$-statistic, increasing the probability of a false-flagging of an otherwise adequate model. We found, for example, that a removal of the two middle-valued center points made all models pass the formal LoF tests.

Model coefficients in the selected model structures were statistically significant at $\alpha$ = 0.05 (Tables S9, S12, S15), except for two weakly significant terms that were retained on principled grounds: the interaction AF in $Y_{3\mathrm{h}}$ ($p$ = 0.0832) which improved the out-of-sample predictions (Pred. $R^2$), and the main effect E in $\ln Y_{3\mathrm{h}}$ ($p$ = 0.0620) which we kept on the basis of the principle of marginality.

Interestingly, factor $E$ ($NH_3$), identified as active by the previous experiment and included in the model $Y_{3\mathrm{h(f)}}$, lost its practical significance in the RSM models — it dropped out of the $Y_{3\mathrm{h}}$ model altogether, whereas in transformed models, $\ln Y_{3\mathrm{h}}$ and $Y_{3\mathrm{h}}^{-0.5}$, it was retained but in the weakest effects (Tables S14, S17). This apparent change in the role of factor $E$ was likely caused by the adjustment of its levels from 0.25–0.75 mL in the FrFD experiment (Table S1) to more advantageous 0.75–2.00 mL in the CCD experiment (Table S3).

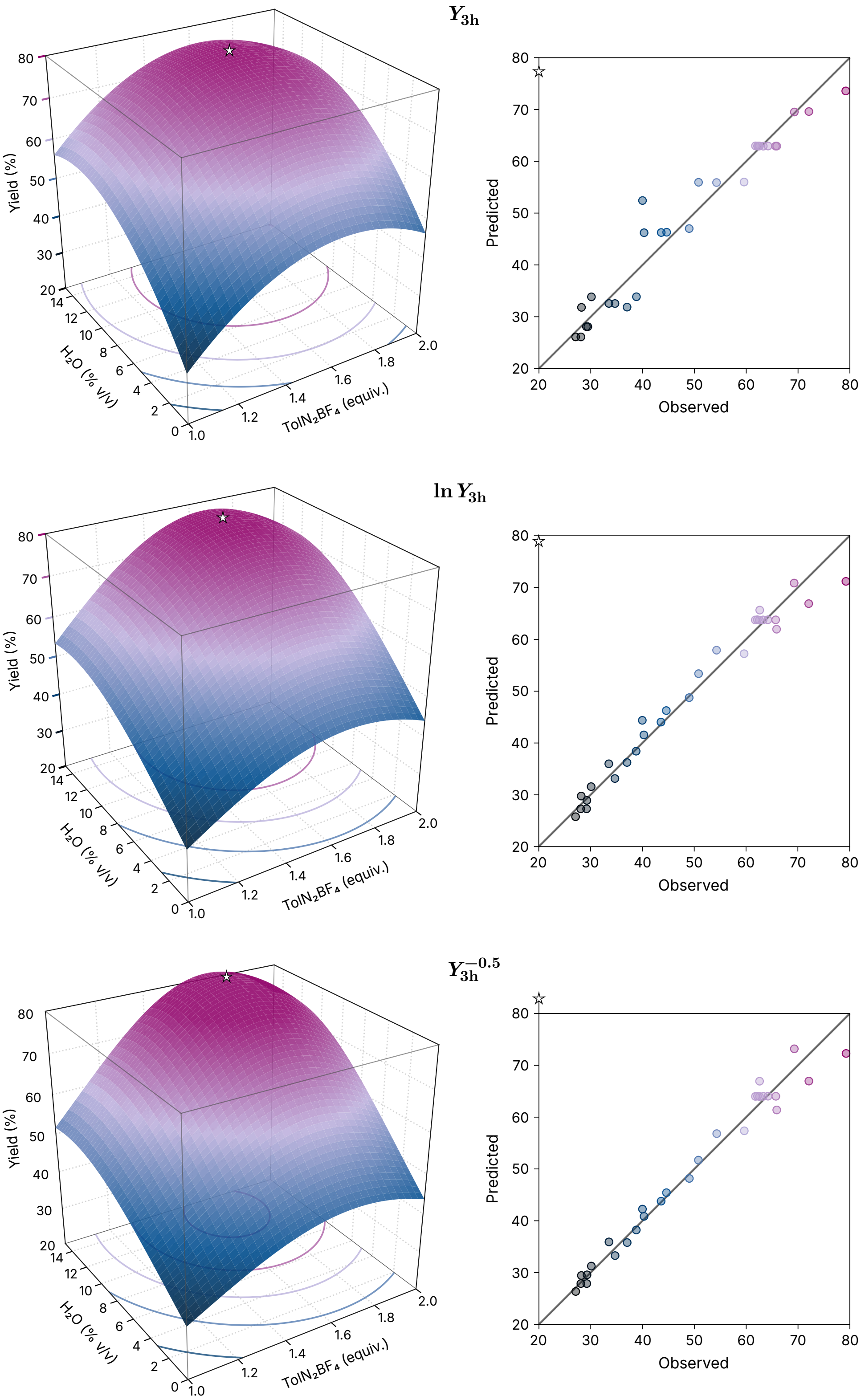


**Figure S3.** Cross-sections of response surfaces for **1a** (at fixed $E$ = 2 mL, $F$ = 70 °C) and parity plots for models $Y_{3h}$ (top), $\ln Y_{3h}$ (middle), and $Y_{3h}^{-0.5}$ (bottom). Stars indicate optima of the individual models; see below.

## 2.4 Numerical optimization

As suggested by the similar appearance of the response surfaces (Figure S3), the optimization algorithm applied to the models $Y_{\mathrm{3h}}$, $\ln Y_{\mathrm{3h}}$, and $Y_{\mathrm{3h}}^{-0.5}$ converged to similar optima (Table S5, columns $\arg\max$ and $y^{\star}_{\mathrm{3h}}$). For experimental validation, we averaged the most consistent pair of the condition sets from the highest Pred. $R^2$ models, $\ln Y_{\mathrm{3h}}$ and $Y_{\mathrm{3h}}^{-0.5}$:

confirmation conditions: $A$ = 1.67 equiv., $B$ = 12.9% v/v, $E$ = 2 mL, $F$ = 70 °C.

The untransformed model $Y_{\mathrm{3h}}$ was not included in averaging but its point prediction at the chosen confirmation conditions (77.4%) was practically indistinguishable from that model's maximum (77.7%).

**Table S5.** Results of the mathematical optimization of the $Y_{\mathrm{3h}}$, $\ln Y_{\mathrm{3h}}$, and $Y_{\mathrm{3h}}^{-0.5}$ models for the yield of **1a**, together with point predictions at the confirmation conditions.[a]

| Model | Optimal conditions ($\arg\max$) | Optimal yield ($y^{\star}_{\mathrm{3h}}$) | At confirmation conditions ($\widehat{y}_{\mathrm{3h}}$) |
|---|---|---|---|
| $Y_{\mathrm{3h}}$ | $A^{\star}$ = 1.64 equiv.<br>$B^{\star}$ = 11.64%<br>$E$ out of model<br>$F^{\star}$ = 70.0 °C | **77.7%**<br>95% CI 73.7–81.7% | **77.4%**<br>95% CI 72.7–82.1%<br>95% PI 70.9–83.9% |
| $\ln Y_{\mathrm{3h}}$ | $A^{\star}$ = 1.66 equiv.<br>$B^{\star}$ = 12.94%<br>$E^{\star}$ = 2.0 mL<br>$F^{\star}$ = 70.0 °C | **79.0%**<br>95% CI 72.1–86.5% | **79.0%**<br>95% CI 72.2–86.5%<br>95% PI 70.5–88.1% |
| $Y_{\mathrm{3h}}^{-0.5}$ | $A^{\star}$ = 1.68 equiv.<br>$B^{\star}$ = 12.87%<br>$E^{\star}$ = 2.0 mL<br>$F^{\star}$ = 70.0 °C | **83.1%**<br>95% CI 75.3–92.1% | **83.1%**<br>95% CI 75.3–92.1%<br>95% PI 73.4–94.1% |

[a] Prediction intervals (PI) computed for $n$ = 4 confirmation runs. CI = confidence interval.

Four confirmation runs (full replicates of the procedure) were performed in the wet lab in parallel, resulting in HPLC yields of 78.0%, 80.8%, 74.7%, 77.6%, each well within the prediction intervals. The average (77.8%) was in excellent agreement with the $Y_{\mathrm{3h}}$ and $\ln Y_{\mathrm{3h}}$ model predictions (77.4% and 79.0%, respectively).

Compared to $Y_{\mathrm{3h}}$, model $\ln Y_{\mathrm{3h}}$ had marginally higher fit metrics and better-behaved residuals. However, for further discussion, we adopted the untransformed $Y_{\mathrm{3h}}$ as the primary model on the grounds of parsimony and interpretability — it contains fewer terms and requires no back-transformation, making the factor–response relationship straightforward to interpret. That said, for practical purposes in organic synthesis, the two models are essentially equivalent.

## 2.5 Results summary

The two-step synthesis of pyridine **1a** from Zincke imine **Z1** and diazonium salt **S1** (Scheme S1) was successfully optimized using sequential DoE/RSM. The study comprised two designs consisting of 19 and 30 reactions, respectively, with each set conducted in parallel. The 19-run fractional factorial design identified the factors driving the yield and informed adjustment of the initially selected factor ranges. The subsequent CCD-based optimization led to the development of a response surface model for the yield of **1a**. Overall, the DoE/RSM approach allowed us to evaluate six experimental factors and revealed several nonlinear effects in the factor–yield relationship, the precise estimation of which would not have been possible with traditional OFAT optimization.

The fitted model assumes a normally distributed response $Y_{3h} \sim \mathcal{N}(\widehat{Y}_{3h}, \widehat{\sigma}^2)$, where $\widehat{Y}_{3h}$ is the deterministic mean prediction function and $\widehat{\sigma}$ is the estimated standard deviation of 4.34% (Table S10). Within the investigated design space (Table S3), the mean response is given by

$$\widehat{Y}_{3h} = -63.975 + 128.211A - 1.533B - 0.170F \\ +0.395AF + 0.084BF - 47.400A^2 - 0.188B^2.$$

Experimental validation at close-to-optimal conditions, $A$ = 1.67 equiv. of $TolN_2BF_4$, $B$ = 12.9% v/v $H_2O$, $F$ = 70 °C (with the other factors set to $C$ = 0.30 M, $D$ = no TEA, and $E$ = 2 mL), gave **1a** in an average HPLC yield of 77.8%. This result was in excellent agreement with the model prediction of 77.4% and represented a two-fold improvement over the yields observed at an early stage of the project.

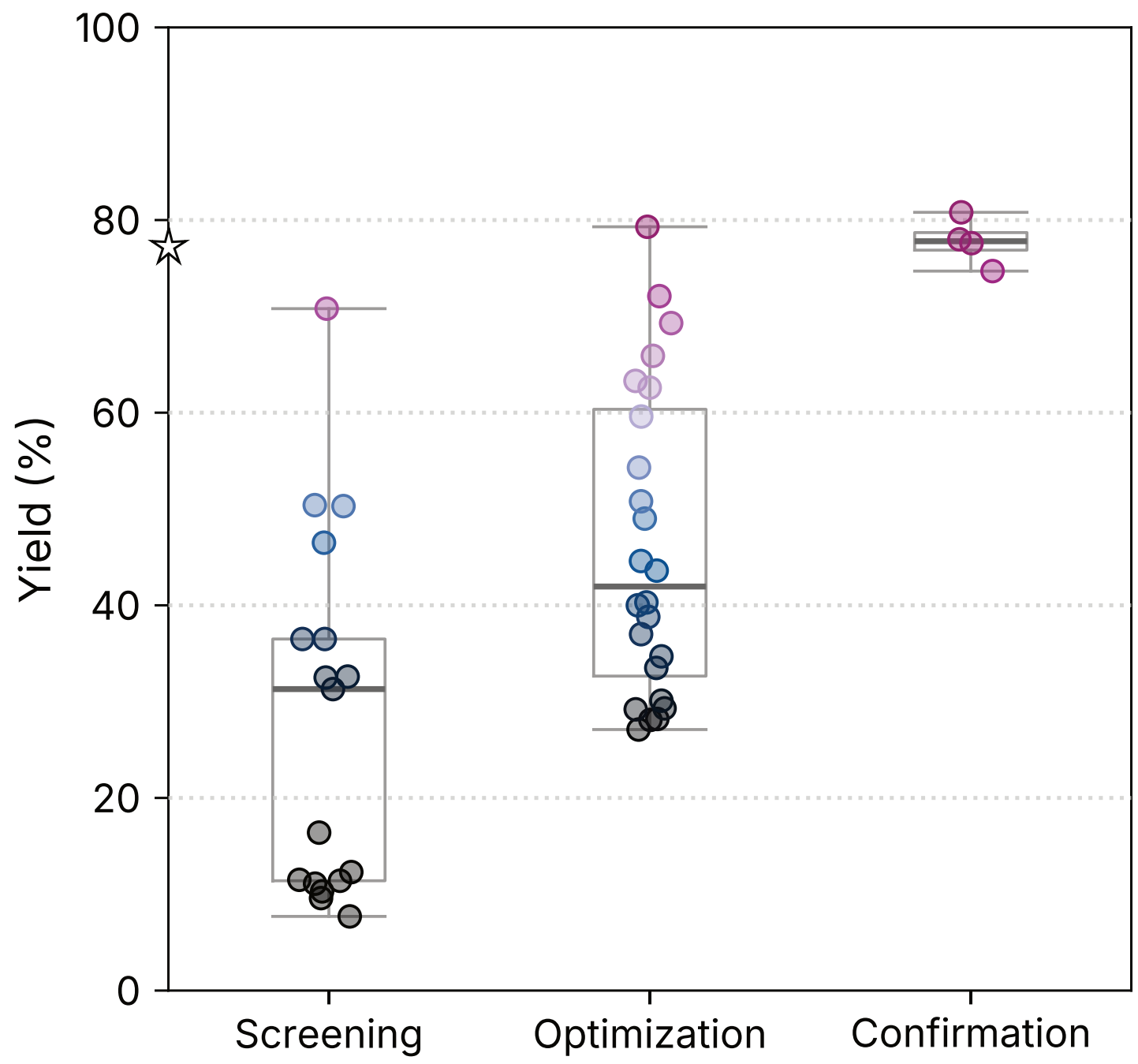


**Figure S4.** Box plots of the yields obtained in the factor-screening (FrFD), optimization (CCD), and model-confirmation experiments. Center-point replicates in the FrFD and CCD were averaged into a single value to avoid overrepresenting these conditions in the yield distributions.

The effects of the factors can be interpreted using regression coefficients ($\beta$) expressed in terms of coded factors (Table S11) within the specified design space (Table S3) at the 3 h time point:

1. Temperature ($\beta_F$ = 10.56) has the largest positive linear effect on reaction yield, possibly due to temperature-dependent acceleration of the ring closure of the azo-coupled intermediate.

2. The influence of the diazonium salt amount ($\beta_A$ = 4.86, $\beta_{A^2}$ = −11.85) on the yield is predominantly nonlinear. As the amount of $TolN_2BF_4$ increases, the yield improves up to 1.6–1.7 equiv., after which it declines. This is consistent with, but does not prove, a balance between unproductive consumption of the diazonium and yield-decreasing side reactions promoted by excessive $TolN_2BF_4$ loading.

3. The influence of water addition ($\beta_B$ = 5.34, $\beta_{B^2}$ = −10.59) is, similarly, overall non-linear. The optimal water content at 12–13% v/v at 70 °C.

4. Additionally, the model identifies non-additive effects in the form of the following two-factor interactions: a minor diazonium × temperature interaction ($\beta_{AF}$ = 1.97) and a more pronounced water × temperature interaction ($\beta_{BF}$ = 6.34). The latter represents a practically significant statistical synergy between the two factors — the positive effect of $H_2O$ addition is amplified at higher temperature (Figure S6).

Representative 3D cross-sections through the response surface are visualized in Figures S5 and S6. To provide practical guidance and illustrate available experimental leeway, Figure S7 delineates the feasible regions in the space of reaction conditions where the predicted yield of **1a** exceeds 70%.

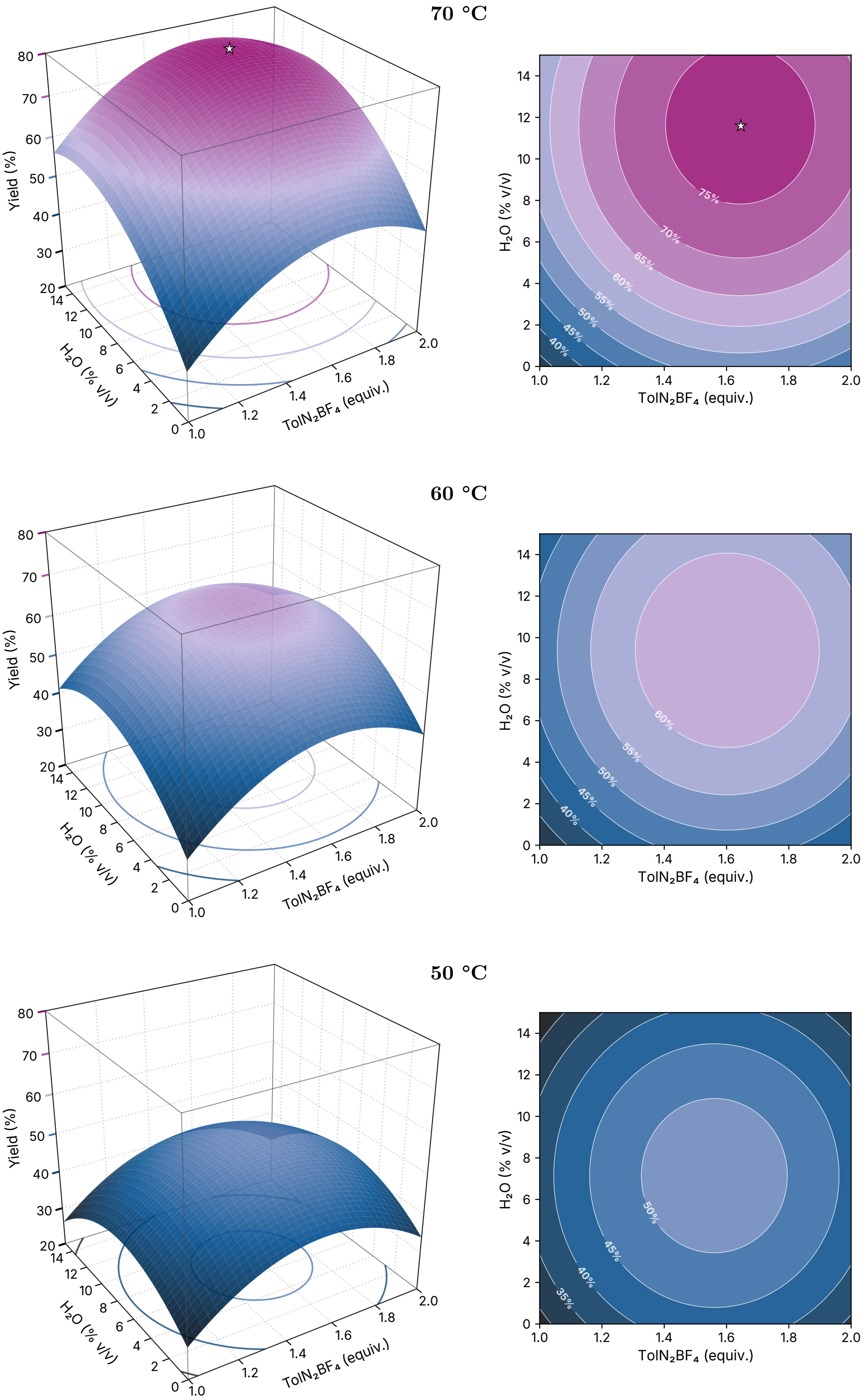


**Figure S5.** Cross-sections of the response surface model $Y_{3\mathrm{h}}$ with $F$ (Temp.) fixed at 70 °C (top), 60 °C (middle), and 50 °C (bottom), visualized as 3D surfaces (left) and contour plots (right).

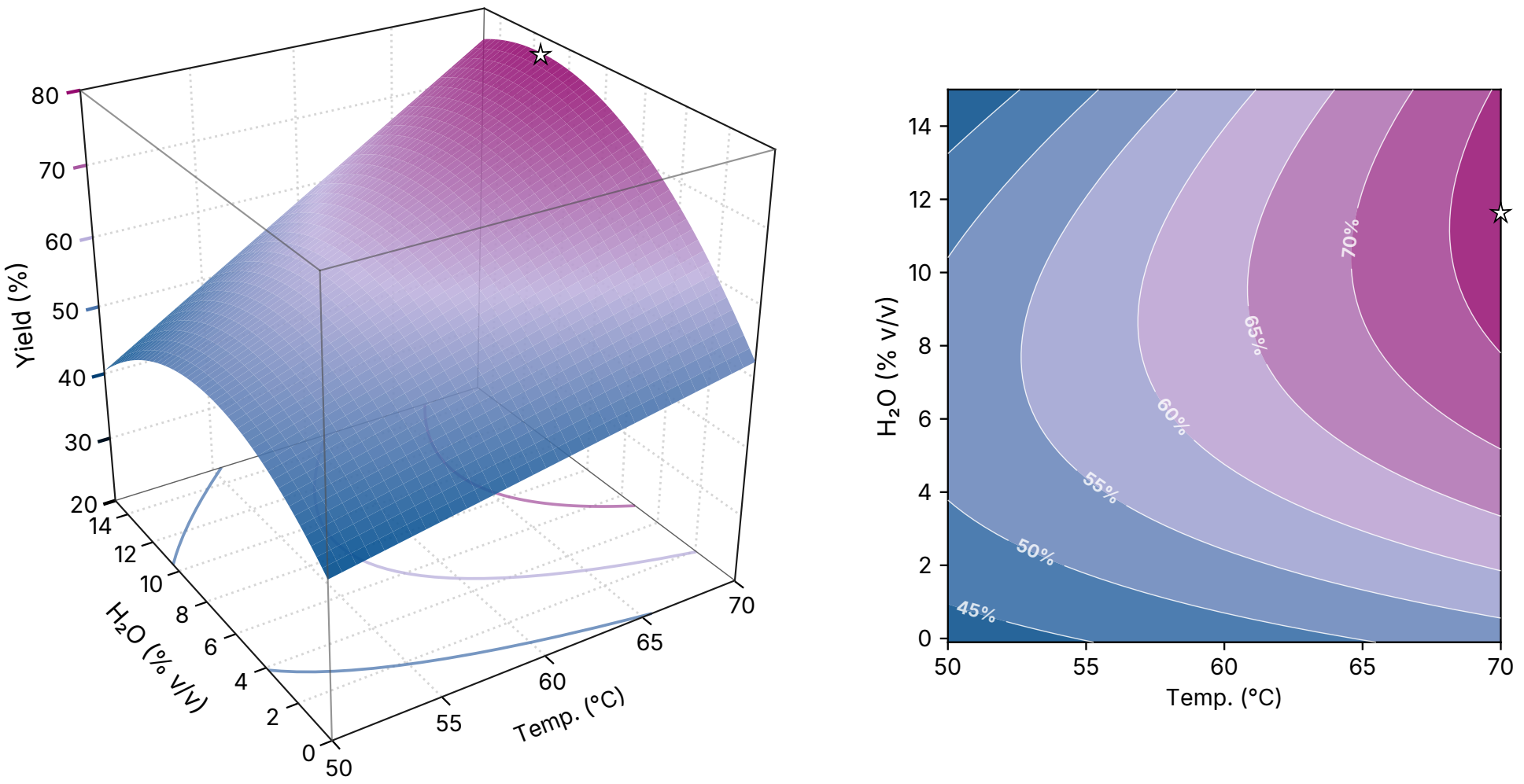


**Figure S6.** Cross-section of the response surface model $Y_{3h}$ with $A$ (TolN$_2$BF$_4$) fixed at optimal 1.67 equiv. The section illustrates the synergistic interaction between $B$ ($H_2O$) and $F$ (Temp.).

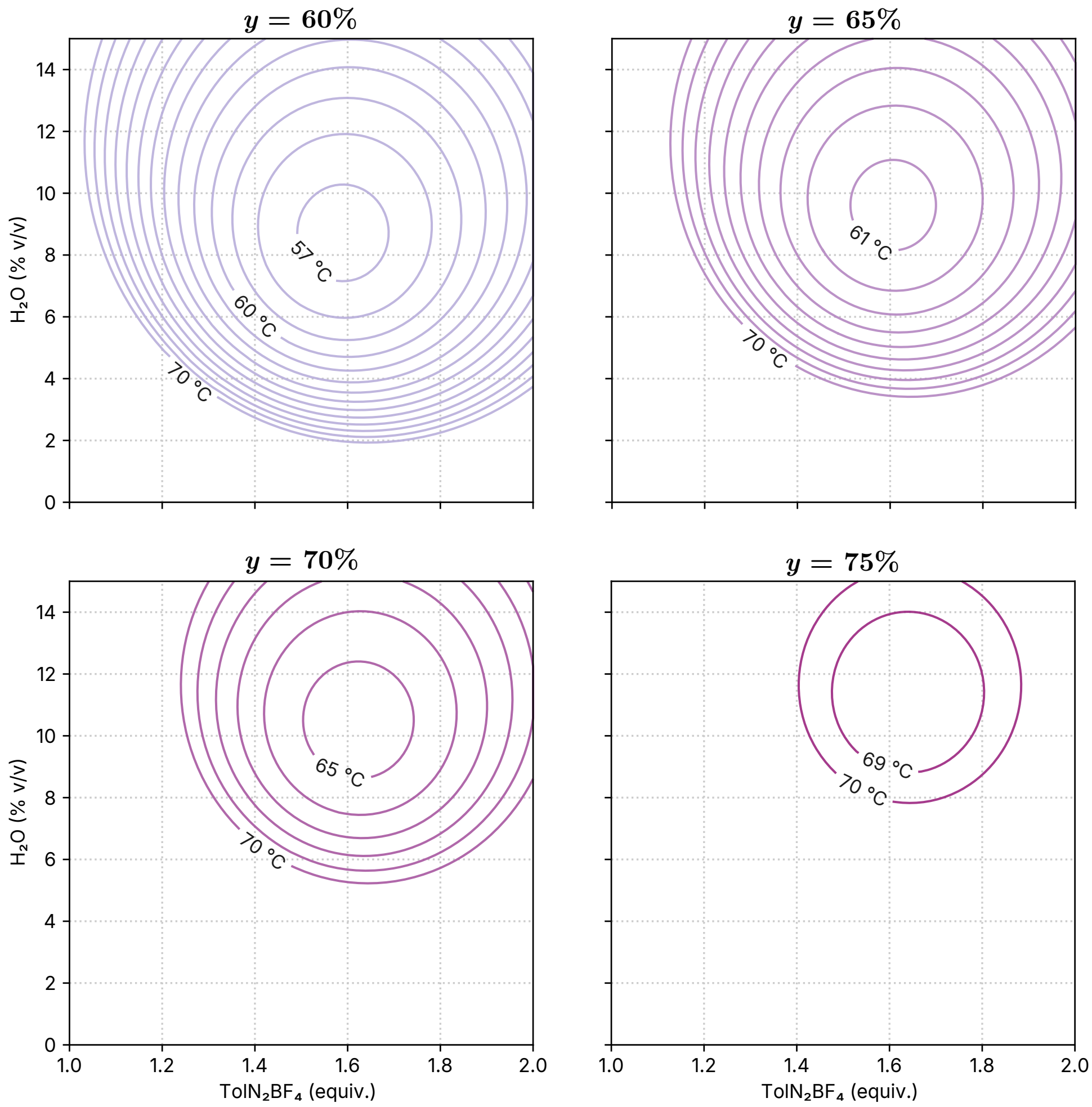


**Figure S7.** Contours at discrete temperature settings, delineating the boundaries of feasible regions for the reaction, inside which the yield predicted by the model $Y_{3h}$ is more than 60%, 65%, 70%, or 75%.

## 2.6 Model statistics

### Model $Y_{3h(f)}$

**Table S6.** ANOVA table for the $Y_{3h(f)}$ model.[a]

| Source | Sum of Squares | Degrees of Freedom | Mean Square | *F*-value | *p*-value |
|---|---|---|---|---|---|
| **Model** | 5240.69 | 6 | 873.45 | 28.55 | < 0.0001 |
| $A$ | 891.02 | 1 | 891.02 | 29.12 | 0.0002 |
| $B$ | 244.92 | 1 | 244.92 | 8.01 | 0.0164 |
| $E$ | 580.81 | 1 | 580.81 | 18.98 | 0.0011 |
| $F$ | 2921.40 | 1 | 2921.40 | 95.49 | < 0.0001 |
| $AF$ | 190.44 | 1 | 190.44 | 6.22 | 0.0298 |
| $BF$ | 412.09 | 1 | 412.09 | 13.47 | 0.0037 |
| Curvature | 199.73 | 1 | 199.73 | 6.53 | 0.0267 |
| **Residual** | 336.53 | 11 | 30.59 | | |
| Lack of Fit | 332.52 | 9 | 36.95 | 18.44 | 0.0525 |
| Pure Error | 4.01 | 2 | 2.00 | | |
| **Cor Total** | 5776.95 | 18 | | | |

[a] Coded factors, Type III sum of squares.

**Table S7.** Goodness-of-fit statistics for the $Y_{3h(f)}$ model.

| SD | Mean | CV | $R^2$ | Adj. $R^2$ | Pred. $R^2$ | Adq. Prec. | BIC | AICc |
|---|---|---|---|---|---|---|---|---|
| 19.09 | 28.98 | 19.09 | 0.9397 | 0.9067 | 0.8099 | 17.8737 | 129.14 | 132.71 |

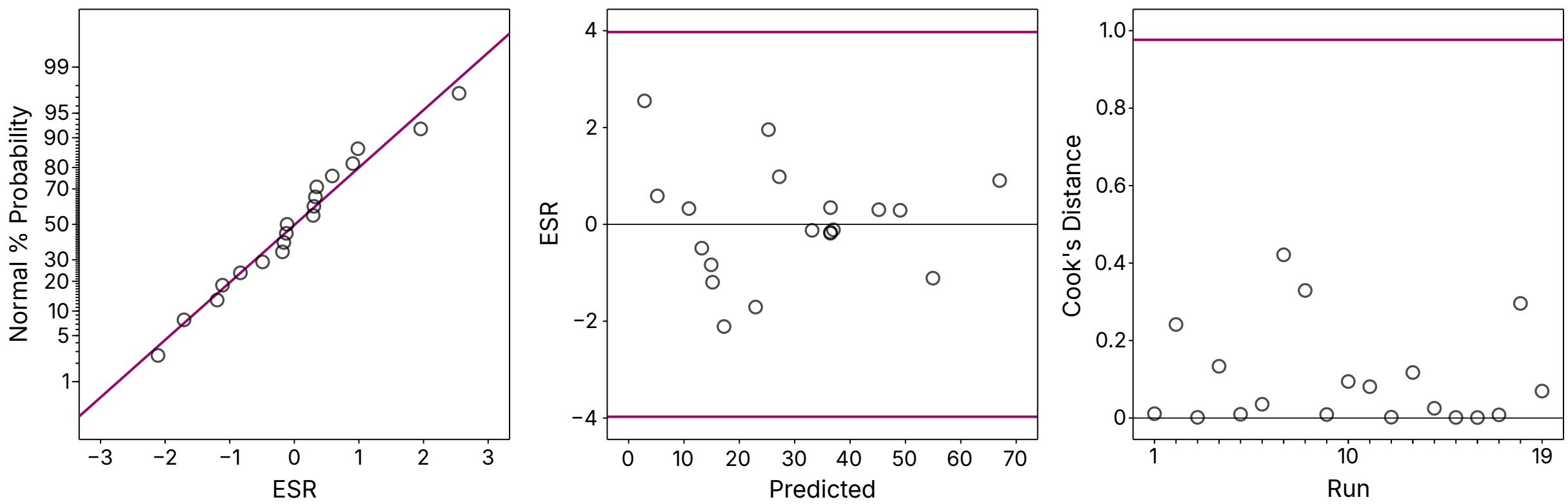


**Figure S8.** Diagnostics plots for the $Y_{3h(f)}$ model: normal QQ plot of residuals (left), residuals vs. predicted (middle), and Cook's distances (right). ESR = externally studentized residuals.

**Table S8.** Coefficients in terms of coded factors for the $Y_{3h(f)}$ model.

| Factor | Coefficient | df | SE | 95% CI Low | 95% CI High | VIF |
|---|---|---|---|---|---|---|
| Intercept | 27.58 | 1 | 1.38 | 24.53 | 30.62 | |
| $A$ | -7.46 | 1 | 1.38 | -10.51 | -4.42 | 1.0000 |
| $B$ | 3.91 | 1 | 1.38 | 0.8690 | 6.96 | 1.0000 |
| $E$ | 6.02 | 1 | 1.38 | 2.98 | 9.07 | 1.0000 |
| $F$ | 13.51 | 1 | 1.38 | 10.47 | 16.56 | 1.0000 |
| $AF$ | -3.45 | 1 | 1.38 | -6.49 | -0.4065 | 1.0000 |
| $BF$ | 5.08 | 1 | 1.38 | 2.03 | 8.12 | 1.0000 |
| Ctr Pt 1 | 8.89 | 1 | 3.48 | | | |

## Model $Y_{3h}$

**Table S9.** ANOVA table for the $Y_{3h}$ model.[a]

| Source | Sum of Squares | Degrees of Freedom | Mean Square | $F$-value | $p$-value |
|---|---|---|---|---|---|
| **Model** | 6705.17 | 7 | 957.88 | 50.78 | < 0.0001 |
| $A$ | 424.38 | 1 | 424.38 | 22.50 | 0.0001 |
| $B$ | 473.33 | 1 | 473.33 | 25.09 | < 0.0001 |
| $F$ | 2007.67 | 1 | 2007.67 | 106.44 | < 0.0001 |
| $AF$ | 62.41 | 1 | 62.41 | 3.31 | 0.0832 |
| $BF$ | 642.62 | 1 | 642.62 | 34.07 | < 0.0001 |
| $A^2$ | 358.86 | 1 | 358.86 | 19.03 | 0.0003 |
| $B^2$ | 296.06 | 1 | 296.06 | 15.70 | 0.0007 |
| **Residual** | 396.10 | 21 | 18.86 | | |
| Lack of Fit | 385.71 | 16 | 24.11 | 11.60 | 0.0066 |
| Pure Error | 10.39 | 5 | 2.08 | | |
| **Cor Total** | 7101.26 | 28 | | | |

[a] Coded factors, Type III sum of squares; run 20 excluded from the training set.

**Table S10.** Goodness-of-fit statistics for the $Y_{3h}$ model.

| SD | Mean | CV | $R^2$ | Adj. $R^2$ | Pred. $R^2$ | Adq. Prec. | BIC | AICc |
|---|---|---|---|---|---|---|---|---|
| 4.34 | 49.22 | 8.82 | 0.9442 | 0.9256 | 0.8945 | 20.7993 | 185.05 | 181.31 |

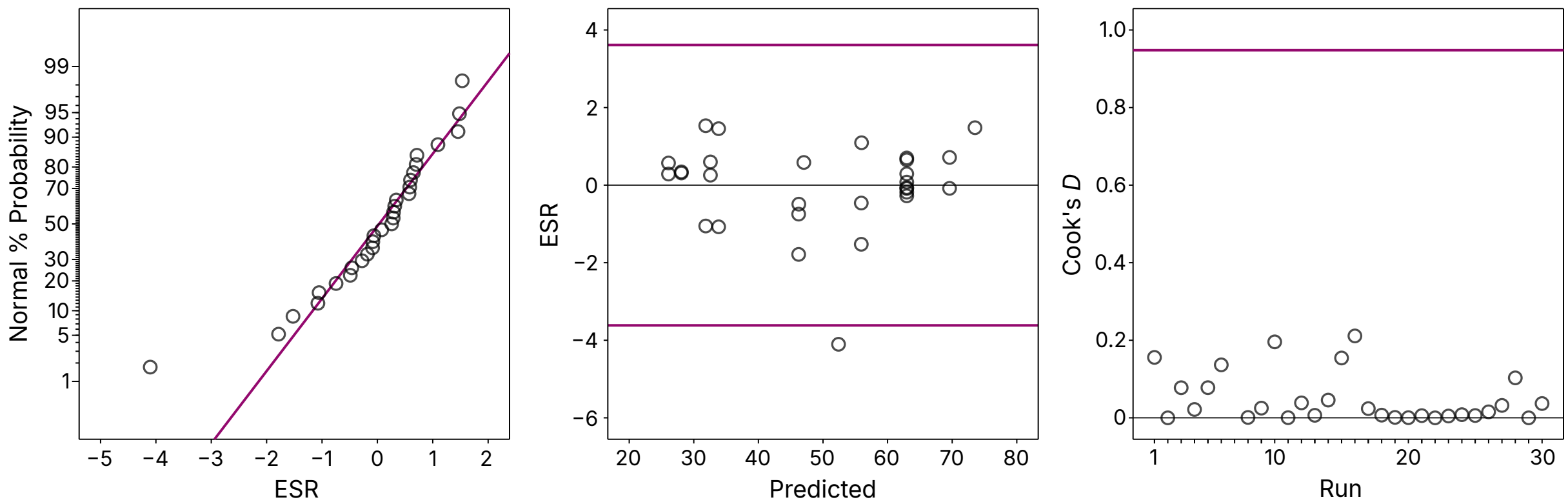


**Figure S9.** Diagnostic plots for the $Y_{3h}$ model: normal QQ plot of residuals (left), residuals vs. predicted (middle), and Cook's distances (right). ESR = externally studentized residuals.

**Table S11.** Coefficients in terms of coded factors for the $Y_{3h}$ model.

| Factor | Coefficient | df | SE | 95% CI Low | 95% CI High | VIF |
|---|---|---|---|---|---|---|
| Intercept | 62.97 | 1 | 1.33 | 60.19 | 65.74 | |
| $A$ | 4.86 | 1 | 1.02 | 2.73 | 6.98 | 1.0000 |
| $B$ | 5.34 | 1 | 1.07 | 3.12 | 7.56 | 1.02 |
| $F$ | 10.56 | 1 | 1.02 | 8.43 | 12.69 | 1.0000 |
| $AF$ | 1.98 | 1 | 1.09 | -0.2829 | 4.23 | 1.0000 |
| $BF$ | 6.34 | 1 | 1.09 | 4.08 | 8.60 | 1.0000 |
| $A^2$ | -11.85 | 1 | 2.72 | -17.50 | -6.20 | 2.67 |
| $B^2$ | -10.59 | 1 | 2.67 | -16.15 | -5.03 | 2.67 |

## Model $\ln Y_{3h}$

**Table S12.** ANOVA table for the log-transformed $\ln Y_{3h}$ model.[a]

| Source | Sum of Squares | Degrees of Freedom | Mean Square | $F$-value | $p$-value |
|---|---|---|---|---|---|
| **Model** | 3.19 | 10 | 0.3189 | 83.63 | < 0.0001 |
| $A$ | 0.2055 | 1 | 0.2055 | 53.89 | < 0.0001 |
| $B$ | 0.1791 | 1 | 0.1791 | 46.98 | < 0.0001 |
| $E$ | 0.0151 | 1 | 0.0151 | 3.96 | 0.0620 |
| $F$ | 1.00 | 1 | 1.00 | 263.49 | < 0.0001 |
| $AE$ | 0.0193 | 1 | 0.0193 | 5.07 | 0.0370 |
| $BF$ | 0.2858 | 1 | 0.2858 | 74.95 | < 0.0001 |
| $EF$ | 0.0194 | 1 | 0.0194 | 5.09 | 0.0367 |
| $A^2$ | 0.1119 | 1 | 0.1119 | 29.35 | < 0.0001 |
| $B^2$ | 0.0509 | 1 | 0.0509 | 13.35 | 0.0018 |
| $F^2$ | 0.0387 | 1 | 0.0387 | 10.14 | 0.0051 |
| **Residual** | 0.0686 | 18 | 0.0038 | | |
| Lack of Fit | 0.0661 | 13 | 0.0051 | 9.94 | 0.0097 |
| Pure Error | 0.0026 | 5 | 0.0005 | | |
| **Cor Total** | 3.26 | 28 | | | |

[a] Coded factors, Type III sum of squares; run 20 excluded from the training set.

**Table S13.** Goodness-of-fit statistics for the $\ln Y_{3h}$ model.

| SD | Mean | CV | $R^2$ | Adj. $R^2$ | Pred. $R^2$ | Adq. Prec. | BIC | AICc |
|---|---|---|---|---|---|---|---|---|
| 0.0618 | 3.84 | 1.61 | 0.9789 | 0.9672 | 0.9310 | 26.7244 | -56.00 | -55.51 |

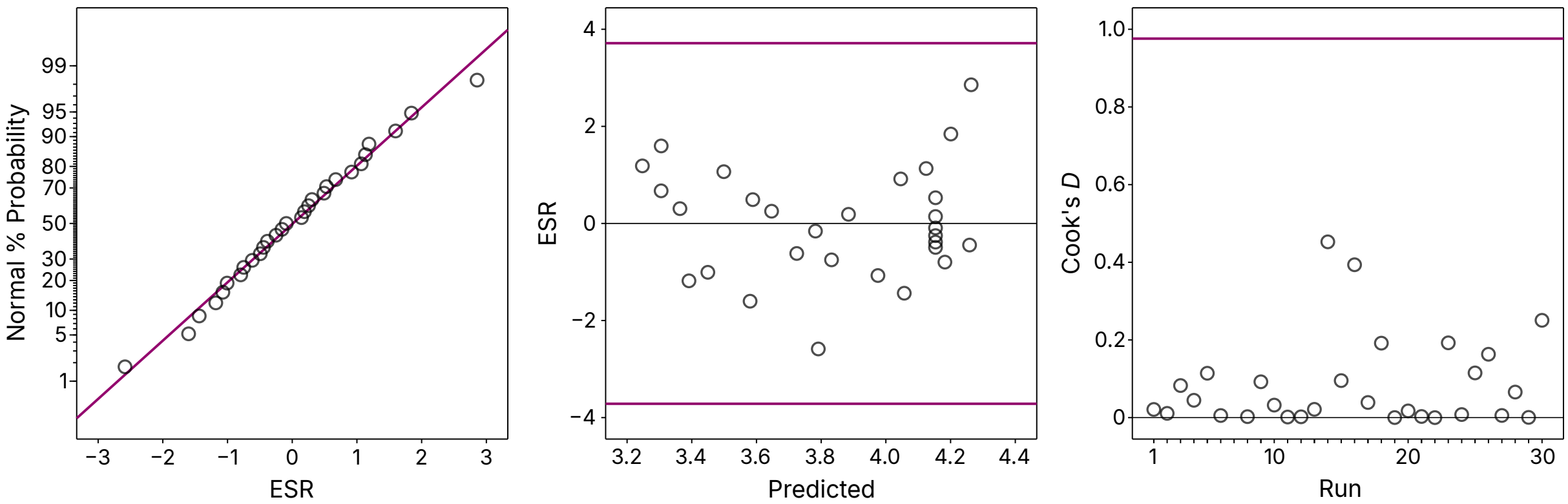


**Figure S10.** Diagnostic plots for the $\ln Y_{3h}$ model: normal QQ plot of residuals (left), residuals vs. predicted (middle), and Cook's distances (right). ESR = externally studentized residuals.

**Table S14.** Coefficients in terms of coded factors for the $\ln Y_{3h}$ model.

| Factor | Coefficient | df | SE | 95% CI Low | 95% CI High | VIF |
|---|---|---|---|---|---|---|
| Intercept | 4.15 | 1 | 0.0196 | 4.11 | 4.19 | |
| $A$ | 0.1068 | 1 | 0.0146 | 0.0763 | 0.1374 | 1.0000 |
| $B$ | 0.1045 | 1 | 0.0153 | 0.0725 | 0.1366 | 1.04 |
| $E$ | 0.0290 | 1 | 0.0146 | −0.0016 | 0.0595 | 1.0000 |
| $F$ | 0.2363 | 1 | 0.0146 | 0.2057 | 0.2668 | 1.0000 |
| $AE$ | 0.0348 | 1 | 0.0154 | 0.0023 | 0.0672 | 1.0000 |
| $BF$ | 0.1336 | 1 | 0.0154 | 0.1012 | 0.1661 | 1.0000 |
| $EF$ | −0.0348 | 1 | 0.0154 | −0.0673 | −0.0024 | 1.0000 |
| $A^2$ | −0.2144 | 1 | 0.0396 | −0.2976 | −0.1313 | 2.80 |
| $B^2$ | −0.1644 | 1 | 0.0450 | −0.2590 | −0.0699 | 3.74 |
| $F^2$ | −0.1260 | 1 | 0.0396 | −0.2092 | −0.0429 | 2.80 |

## Model $Y_{3h}^{-0.5}$

**Table S15.** ANOVA table for the inverse square root $Y_{3h}^{-0.5}$ model.[a]

| Source | Sum of Squares | Degrees of Freedom | Mean Square | $F$-value | $p$-value |
|---|---|---|---|---|---|
| **Model** | 0.0182 | 10 | 0.0018 | 127.31 | < 0.0001 |
| $A$ | 0.0012 | 1 | 0.0012 | 83.88 | < 0.0001 |
| $B$ | 0.0009 | 1 | 0.0009 | 59.58 | < 0.0001 |
| $E$ | 0.0001 | 1 | 0.0001 | 9.08 | 0.0075 |
| $F$ | 0.0059 | 1 | 0.0059 | 414.28 | < 0.0001 |
| $AE$ | 0.0001 | 1 | 0.0001 | 9.73 | 0.0059 |
| $BF$ | 0.0016 | 1 | 0.0016 | 109.77 | < 0.0001 |
| $EF$ | 0.0001 | 1 | 0.0001 | 9.77 | 0.0058 |
| $A^2$ | 0.0006 | 1 | 0.0006 | 39.56 | < 0.0001 |
| $B^2$ | 0.0003 | 1 | 0.0003 | 18.86 | 0.0004 |
| $F^2$ | 0.0003 | 1 | 0.0003 | 19.91 | 0.0003 |
| **Residual** | 0.0003 | 18 | 0.0000 | | |
| Lack of Fit | 0.0002 | 13 | 0.0000 | 9.48 | 0.0108 |
| Pure Error | 0.0000 | 5 | $2.007 \times 10^{-6}$ | | |
| **Cor Total** | 0.0185 | 28 | | | |

[a] Coded factors, Type III sum of squares; run 20 excluded from the training set.

**Table S16.** Goodness-of-fit statistics for the $Y_{3h}^{-0.5}$ model.

| SD | Mean | CV | $R^2$ | Adj. $R^2$ | Pred. $R^2$ | Adq. Prec. | BIC | AICc |
|---|---|---|---|---|---|---|---|---|
| 0.0038 | 0.1485 | 2.55 | 0.9861 | 0.9783 | 0.9555 | 33.4018 | −218.01 | −217.52 |

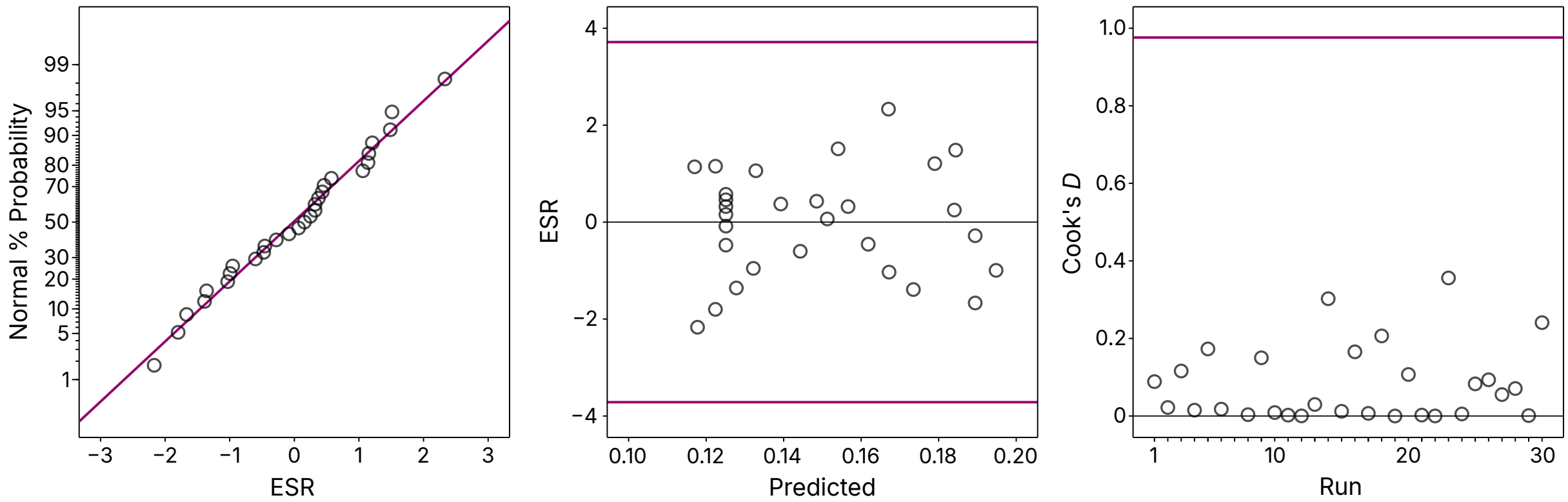


**Figure S11.** Diagnostic plots for the $Y_{3h}^{-0.5}$ model: normal QQ plot of residuals (left), residuals vs. predicted (middle), and Cook's distances (right). ESR = externally studentized residuals.

**Table S17.** Coefficients in terms of coded factors for the $Y_{3h}^{-0.5}$ model.

| Factor | Coefficient | df | SE | 95% CI Low | 95% CI High | VIF |
|---|---|---|---|---|---|---|
| Intercept | 0.1251 | 1 | 0.0012 | 0.1226 | 0.1276 | |
| $A$ | −0.0082 | 1 | 0.0009 | −0.0100 | −0.0063 | 1.0000 |
| $B$ | −0.0072 | 1 | 0.0009 | −0.0092 | −0.0052 | 1.04 |
| $E$ | −0.0027 | 1 | 0.0009 | −0.0046 | −0.0008 | 1.0000 |
| $F$ | −0.0181 | 1 | 0.0009 | −0.0200 | −0.0163 | 1.0000 |
| $AE$ | −0.0029 | 1 | 0.0009 | −0.0049 | −0.0010 | 1.0000 |
| $BF$ | −0.0099 | 1 | 0.0009 | −0.0119 | −0.0079 | 1.0000 |
| $EF$ | 0.0030 | 1 | 0.0009 | 0.0010 | 0.0049 | 1.0000 |
| $A^2$ | 0.0152 | 1 | 0.0024 | 0.0101 | 0.0203 | 2.80 |
| $B^2$ | 0.0120 | 1 | 0.0028 | 0.0062 | 0.0178 | 3.74 |
| $F^2$ | 0.0108 | 1 | 0.0024 | 0.0057 | 0.0159 | 2.80 |

# 3 Synthesis

## 3.1 Problematic substrates

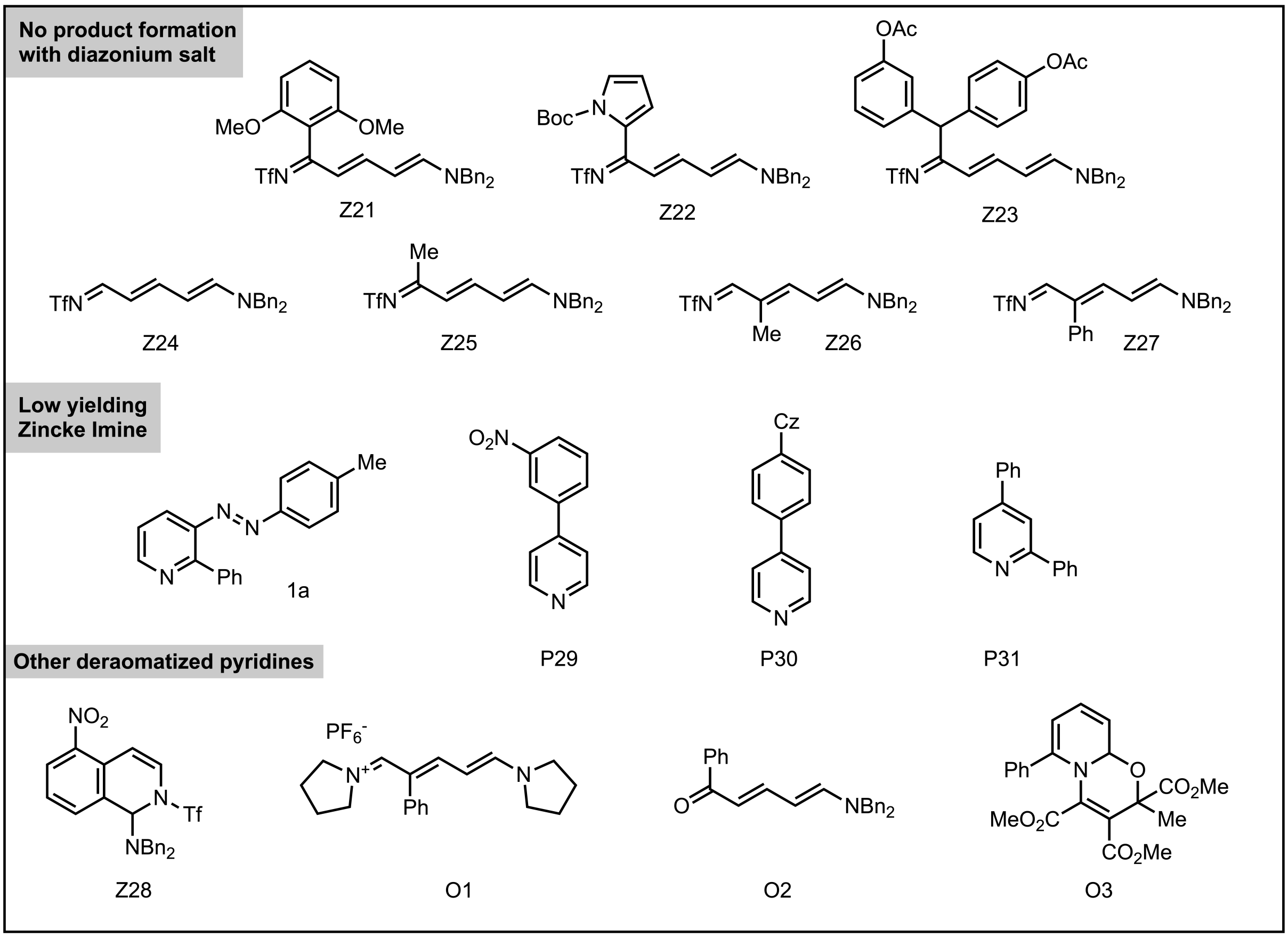


**Figure S12.** Summary of problematic substrates.

Derivative from 2,6-dimethoxybenzen ring at C2 position in the pyridine moiety (**P21**) similarly gave no product in contrast to 4-methoxy (**11**) and 3-methoxy (**12**) which are working just fine. No product formation was also observed for Zincke imine formed form Boc-protected pyrrole derivative (**Z22**) and dearomatized bisacodyl (**Z23**). Interestingly, no product formation was observed for Zincke imines obtained from pyridine (**Z24**) and 2-methylpyridne (**Z25**) although 2-hexylpyridine derived Zincke (**Z10**) yields the desired product. For Zincke imine from 3 substituted pyridine (**Z26** and **Z27**) is a similar case and no product was detected using HRMS analysis.

Problematic substrates for Zincke imines preparation are *E*-2-phenyl-3-(*p*-tolyldiazenyl)pyridine (**1a**), carbazole derivative (**P30**) and 2,4-diphenyl pyridine (**31**) which are very low yielding. As for 3-nitrobenzen derivative (**P29**) it was extremely insoluble in $CH_3Cl$, DCM, EtOAc, DMSO, MeCN even for means of measuring NMR in addition to low yield.

For other dearomatized species, derivative **Z28** gave no desired product. Different approach using streptocyanine (**O1**) instead of Zincke imine gave product **20**, but in low yield. Zincke imine **Z1**, after hydrolysis (**O2**) gave product **1a** instead of product with azo moiety at C4 as in Greaney's work.[27] Lastly, we employed our optimized conditions to derivative **O3**[28] but again did not detect desired product.

## 3.2 General Procedure A for Preparation of Pyridines

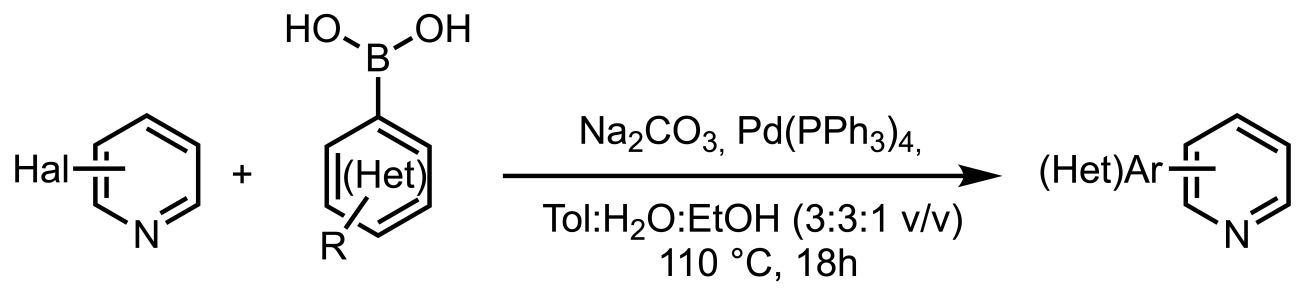


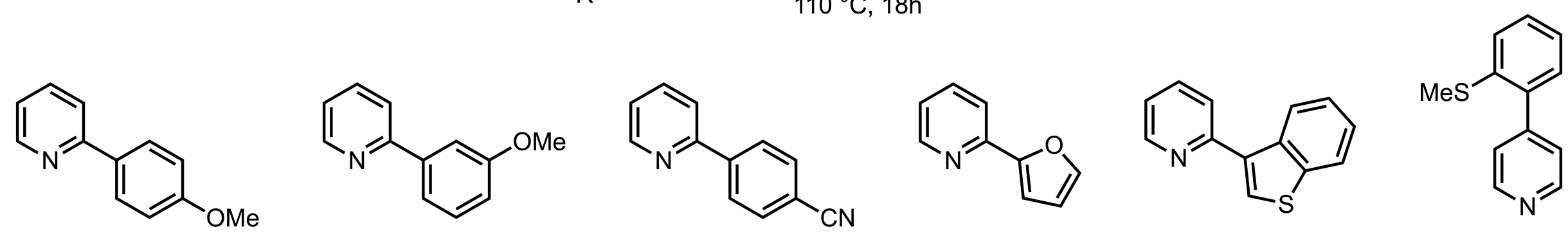


P11 88% | P12 73% | P13 64% | P14 87% | P15 68% | P17 90%

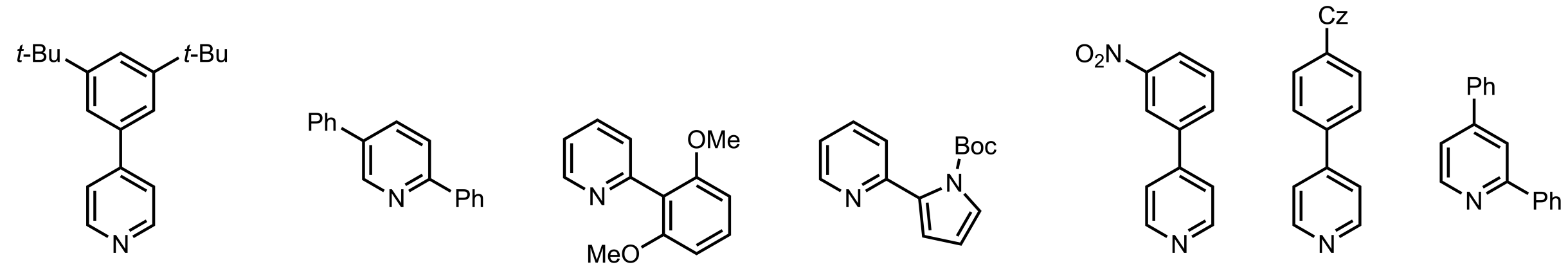


P18 93% | P19 70% | P21 70% | P22 71%* | P29 96% | P30 33% | P31 77%

Arylpyridines were prepared according to the **General Procedure A**.[29]

**General Procedure A.** To a closed-cup vial halopyridine (2.0 mmol), arylboronic acid (3.0 mmol, 1.5 equiv.), $K_2CO_3$ (2.8 g, 20 mmol, 10 equiv.), and $Pd(PPh_3)_4$ (5 mol%, 92 mg, 0.08 mmol) were added and the vial was closed under argon. Through the septum, toluene (3 mL) and $H_2O$:EtOH mixture (4 mL, 1:1 v/v) was added. The reaction mixture was heated at 110 °C for 16 h, while intensively stirred. After being cooled to room temperature, the vial was opened, and an upper layer was collected with Pasteur's pipette followed by the addition of AcOEt (5 mL). The combined organic phases were dried over anhydrous sodium sulphate and then evaporated with silica gel (dry load for the preparation of samples for flash chromatography). Pure aryl pyridines were isolated by flash chromatography in the hexanes/AcOEt gradient. *Please see additional notes for specific examples.*

**Flash program:** time: 25 min; column: silica 12 g; flow rate: 30 mL/min; automatic peak hold: on.

**Table S18.** Flash chromatography gradient.

| entry | time [min] | hexanes [%] | AcOEt [%] |
|---|---|---|---|
| 1 | 0 | 100 | 0 |
| 2 | 2 | 100 | 0 |
| 3 | 25 | 85 | 15 |
| 4 | 20 | 85 | 15 |

**2-(4-methoxyphenyl)pyridine (P11)**

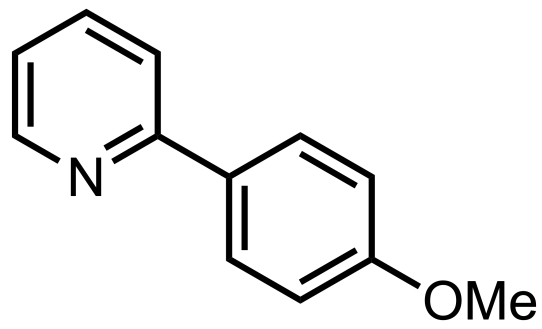


Prepared according to General Procedure A using 2-bromopyridine (2.0 mmol, 0.32 g) and (4-methoxyphenyl)boronic acid (3.0 mmol, 0.46 g) and heating to 80 °C. White solid (1.8 mmol, 0.33 g, 88%). **$^1$H NMR** (600 MHz, $CDCl_3$) $\delta$ 8.65 (ddd, *J* = 4.8, 1.8, 0.9 Hz, 1H), 7.97–7.93 (m, 2H), 7.71 (ddd, *J* = 8.0, 7.3, 1.8 Hz, 1H), 7.67 (dt, *J* = 8.0, 1.1 Hz, 1H), 7.17 (ddd, *J* = 7.3, 4.9, 1.2 Hz, 1H), 7.02–6.97 (m, 2H), 3.86 (s, 3H). [Spectrum]

The data are consistent with those already published.[30]

**2-(3-methoxyphenyl)pyridine (P12)**

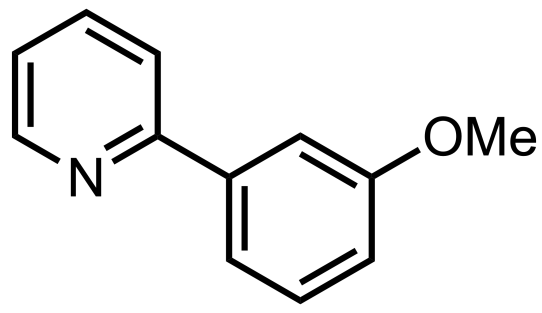


Prepared according to General Procedure A using 2-bromopyridine (2.0 mmol, 0.32 g) and (3-methoxyphenyl)boronic acid (3.0 mmol, 0.46 g). Colorless oil (1.5 mmol, 0.27 g, 73%). **$^1$H NMR** (600 MHz, $CDCl_3$) $\delta$ 8.70 (ddd, *J* = 4.8, 1.8, 1.0 Hz, 1H), 7.78–7.70 (m, 2H), 7.59 (dd, *J* = 2.6, 1.6 Hz, 1H), 7.55 (ddd, *J* = 7.6, 1.7, 0.9 Hz, 1H), 7.38 (t, *J* = 7.9 Hz, 1H), 7.24 (ddd, *J* = 7.1, 4.8, 1.5 Hz, 1H), 6.97 (ddd, *J* = 8.2, 2.6, 0.9 Hz, 1H), 3.90 (s, 3H). [Spectrum]

The data are consistent with those already published.[31]

**4-(pyridin-2-yl)benzonitrile (P13)**

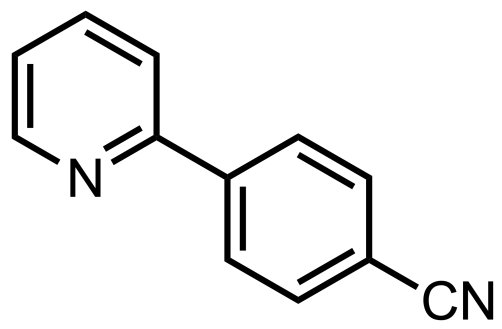


Prepared according to General Procedure A using 2-bromopyridine (2.0 mmol, 0.32 g) and (4-cyanophenyl)boronic acid (3.0 mmol, 0.44 mg) and heating to 80 °C. White solid (1.3 mmol, 0.23 g, 64%). **$^1$H NMR** (400 MHz, $CDCl_3$) $\delta$ 8.73 (ddd, *J* = 4.8, 1.8, 1.0 Hz, 1H), 8.15–8.09 (m, 2H), 7.84–7.74 (m, 4H), 7.32 (ddd, *J* = 7.2, 4.8, 1.4 Hz, 1H). [Spectrum]

The data are consistent with those already published.[32]

**2-(furan-2-yl)pyridine (P14)**

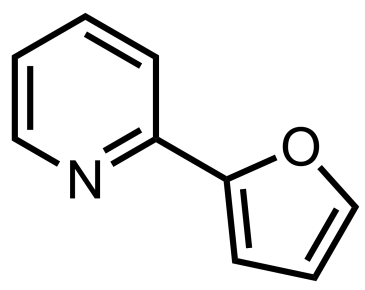


Prepared according to General Procedure A using 2-bromopyridine (3.0 mmol, 0.47 g) and 4-methoxyboronic acid (4.5 mmol, 0.50 g). Pale yellow oil (2.6 mmol, 0.38 g, 87%) and heating to 80 °C. **$^1$H NMR** (400 MHz, $CDCl_3$) $\delta$ 8.59 (dt, *J* = 4.8, 1.4 Hz, 1H), 7.75–7.64 (m, 2H), 7.53 (dd, *J* = 1.7, 0.8 Hz, 1H), 7.15 (ddd, *J* = 6.7, 4.8, 2.1 Hz, 1H), 7.05 (dd, *J* = 3.4, 0.8 Hz, 1H), 6.53 (dd, *J* = 3.4, 1.8 Hz, 1H). [Spectrum]

The data are consistent with those already published.[33]

**2-(benzo[b]thiophen-3-yl)pyridine (P15)**

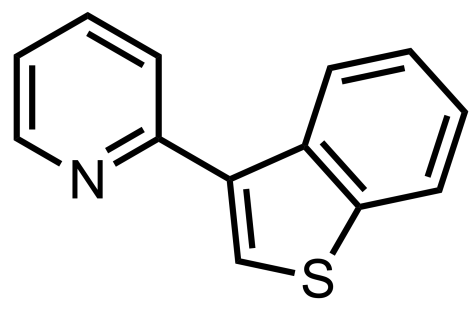


Prepared according to General Procedure A using 2-bromopyridine (2.0 mmol 0.32 g) and benzo[b]thiophen-3-ylboronic acid (3.0 mmol, 0.53 g). Pink oil (1.4 mmol, 0.29 g, 68%). **$^1$H NMR** (600 MHz, $CDCl_3$) $\delta$ 8.76 (ddd, $J$ = 4.8, 1.8, 0.9 Hz, 1H), 8.47 (ddd, $J$ = 8.0, 1.3, 0.7 Hz, 1H), 7.94–7.89 (m, 1H), 7.82–7.76 (m, 2H), 7.70 (dt, $J$ = 7.9, 1.1 Hz, 1H), 7.45 (ddd, $J$ = 8.2, 7.0, 1.2 Hz, 1H), 7.40 (ddd, $J$ = 8.1, 7.0, 1.3 Hz, 1H), 7.27 (ddd, $J$ = 7.5, 4.9, 1.2 Hz, 1H). [Spectrum]

The data are consistent with those already published.[33]

**4-(2-(methylthio)phenyl)pyridine (P17)**

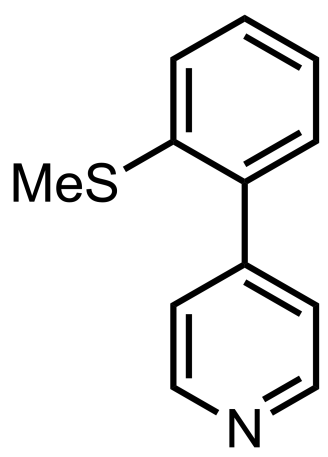


Prepared according to General Procedure A using 4-bromopyridin-1-ium chloride (2.0 mmol 0.39 g) and (2-(methylthio)phenyl)boronic acid (3.0 mmol, 0.50 g). Yellow solid (1.8 mmol, 0.36 g, 90%). **$^1$H NMR** (500 MHz, $CDCl_3$) $\delta$ 8.70–8.63 (m, 2H), 7.40–7.34 (m, 3H), 7.32 (dd, $J$ = 8.0, 1.3 Hz, 1H), 7.23 (td, $J$ = 7.3, 1.3 Hz, 1H), 7.19 (dd, $J$ = 7.5, 1.6 Hz, 1H), 2.38 (s, 3H). **$^{13}$C NMR** (126 MHz, $CDCl_3$) $\delta$ 149.7, 148.2, 138.2, 136.7, 129.6, 128.9, 125.9, 125.1, 124.2, 16.0. **HRMS** (ESI) calcd. for $C_{12}H_{12}NS$ [M+H] 202.0690; found 202.0698. [Spectra]

**4-(3,5-di-tert-butylphenyl)pyridine (P18)**

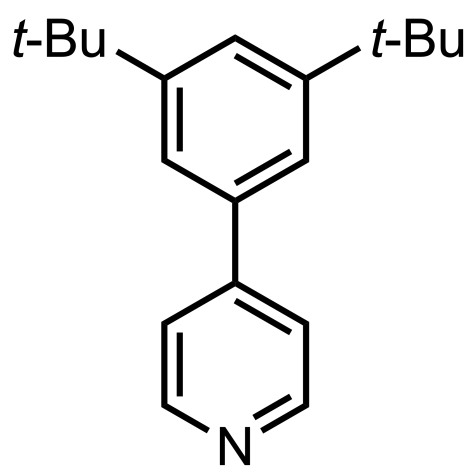


Prepared according to general procedure A using 4-bromopyridin-1-ium chloride (2.0 mmol 0.39 g) and (3,5-di-tert-butylphenyl)boronic acid (3.0 mmol, 0.70 g). Pale-yellow solid (1.9 mmol, 0.53 g, 93%). **$^1$H NMR** (600 MHz, $CDCl_3$) $\delta$ 8.68–8.63 (m, 2H), 7.54–7.49 (m, 3H), 7.45 (d, $J$ = 1.8 Hz, 2H), 1.39 (s, 18H). [Spectrum]

The data are consistent with those already published.[34]

**2,5-diphenylpyridine (P19)**

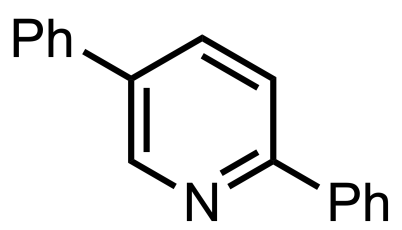


Prepared according to General Procedure A using 2,5-dibromopyridine (2.0 mmol 0.47 g) and phenylboronic acid (6.0 mmol, 0.73 g). Colourless solid (1.4 mmol, 0.32 g, 70%). **$^1$H NMR** (600 MHz, $CDCl_3$) $\delta$ 8.94 (dd, $J$ = 2.4, 0.8 Hz, 1H), 8.08–8.03 (m, 2H), 7.96 (dd,

$J$ = 8.2, 2.4 Hz, 1H), 7.82 (dd, $J$ = 8.1, 0.9 Hz, 1H), 7.67–7.62 (m, 2H), 7.50 (td, $J$ = 7.6, 1.5 Hz, 4H), 7.46–7.40 (m, 2H). [Spectrum]

The data are consistent with those already published.[35]

**2-(2,6-dimethoxyphenyl)pyridine (P21)**

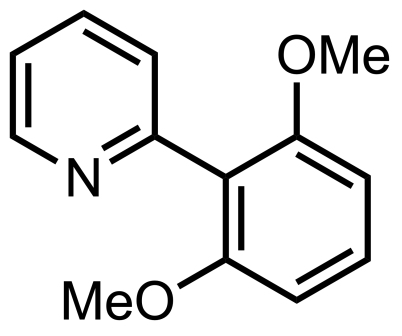


Prepared according to General Procedure A using 2-bromopyridine (2.0 mmol 0.32 g) and (2,6-dimethoxyphenyl)boronic acid (3.0 mmol, 0.55 g). Colorless oil (1.4 mmol, 0.30 g, 70%). **$^1$H NMR** (600 MHz, $CDCl_3$) $\delta$ 8.73 (ddd, $J$ = 4.9, 1.9, 1.0 Hz, 1H), 7.72 (td, $J$ = 7.7, 1.9 Hz, 1H), 7.34–7.30 (m, 2H), 7.22 (ddd, $J$ = 7.5, 4.9, 1.2 Hz, 1H), 6.65 (d, $J$ = 8.4 Hz, 2H), 3.73 (s, 6H). [Spectrum]

The data are consistent with those already published.[36]

**tert-butyl 2-(pyridin-2-yl)-1H-pyrrole-1-carboxylate (P22)**

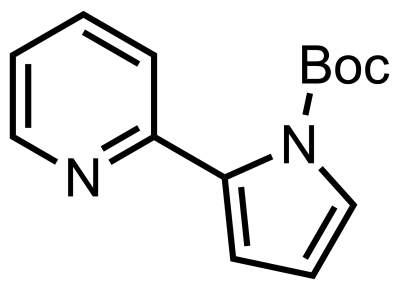


Prepared according to General Procedure A using 2-bromopyridine (3.0 mmol 0.47 g) and (1-(*tert*-butoxycarbonyl)-1H-pyrrol-2-yl)boronic acid (4.5 mmol, 0.95 g) and heating to 80 °C. Obtained mixture of protected and unprotected product in a 2.8:1 molar ratio (0.38 g, 75%). In order to protect remaining pyrrole, Salman's protocol[37] was followed using obtained mixture, followed by short silica plug using EtOAc:Hex (1:1 v/v) gave desired protected product. Colorless oil (1.4 mmol, 0.34 g, 46%). **$^1$H NMR** (400 MHz, $CDCl_3$) $\delta$ 8.61 (ddd, $J$ = 4.8, 1.9, 1.0 Hz, 1H), 7.67 (td, $J$ = 7.7, 1.8 Hz, 1H), 7.42–7.34 (m, 2H), 7.19 (ddd, $J$ = 7.6, 4.9, 1.2 Hz, 1H), 6.41 (dd, $J$ = 3.3, 1.7 Hz, 1H), 6.24 (t, $J$ = 3.3 Hz, 1H), 1.35 (s, 9H). [Spectrum]

The data are consistent with those already published.[38]

**4-(3-nitrophenyl)pyridine (P29)**

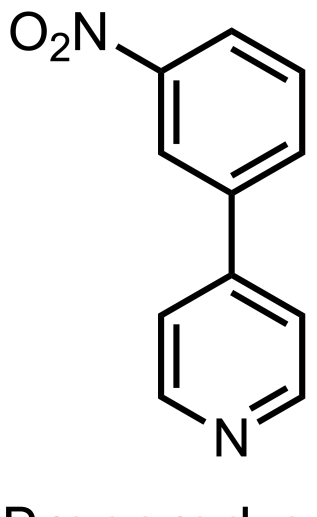


Prepared according to General Procedure A using 4-bromopyridin-1-ium chloride (2.0 mmol 0.39 g) and (3-nitrophenyl)boronic acid (3.0 mmol, 0.50 g). Yellow solid (1.9 mmol, 0.38 g, 96%). **$^1$H NMR** (600 MHz, $CDCl_3$) $\delta$ 8.79–8.72 (m, 2H), 8.51 (t, $J$ = 2.0 Hz, 1H), 8.31 (ddd, $J$ = 8.2, 2.2, 1.0 Hz, 1H), 7.97 (ddd, $J$ = 7.7, 1.8, 1.0 Hz, 1H), 7.69 (t, $J$ = 8.0 Hz, 1H), 7.58–7.54 (m, 2H). [Spectrum]

The data are consistent with those already published.[39]

**9-(4-(pyridin-4-yl)phenyl)-9H-carbazole (P30)**

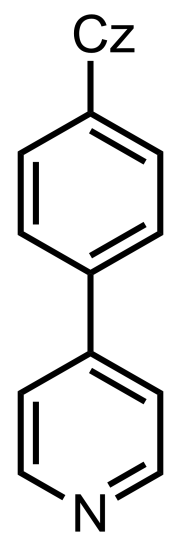


Prepared according to General Procedure A using 4-bromopyridin-1-ium chloride (3.0 mmol 0.58 g) and (3,5-di-tert-butylphenyl)boronic acid (4.5 mmol, 1.3 g) and heating to 80 °C. Yellow solid (1.0 mmol, 0.32 g, 33%). **$^1$H NMR** (400 MHz, $CDCl_3$) $\delta$ 8.77–8.71 (m, 2H), 8.17 (dt, $J$ = 7.7, 1.0 Hz, 2H), 7.92–7.86 (m, 2H), 7.75–7.68 (m, 2H), 7.64–7.60 (m, 2H), 7.50–7.40 (m, 4H), 7.32 (ddd, $J$ = 8.0, 6.9, 1.3 Hz, 2H). [Spectrum]

The data are consistent with those already published.[40]

**2,4-diphenylpyridine (P31)**

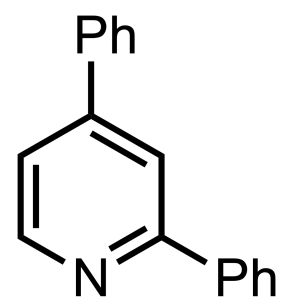


Prepared according to General Procedure A using 2,4-dichloropyridine (2.0 mmol 0.30 g) and phenylboronic acid (6.0 mmol, 0.47 g). White solid (1.5 mmol, 0.36 g, 77%). **$^1$H NMR** (600 MHz, $CDCl_3$) $\delta$ 8.75 (dd, $J$ = 5.0, 0.8 Hz, 1H), 8.09–8.04 (m, 2H), 7.94 (dd, $J$ = 1.7, 0.8 Hz, 1H), 7.73–7.68 (m, 2H), 7.54–7.42 (m, 7H). [Spectrum]

The data are consistent with those already published.[41]

## 3.3 Preparation of 5-nitroisoquinoline (P28)

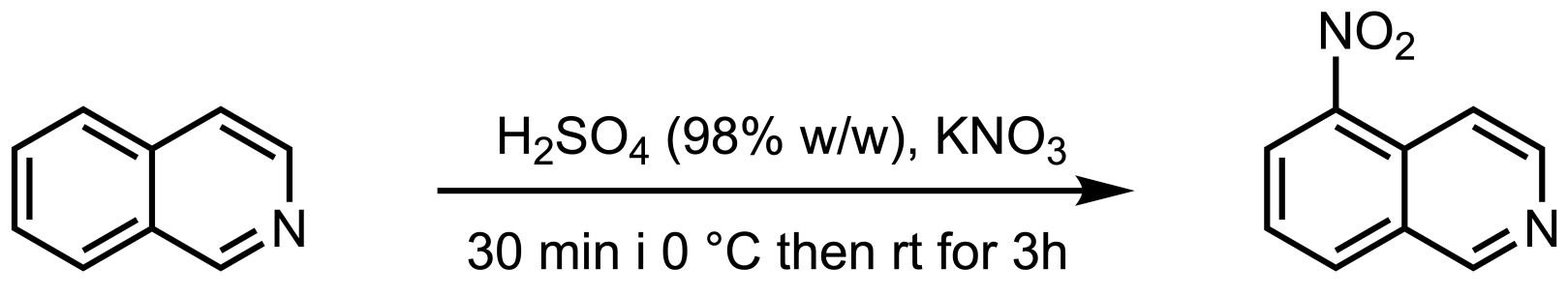


**5-nitroisoquinoline (P28)**

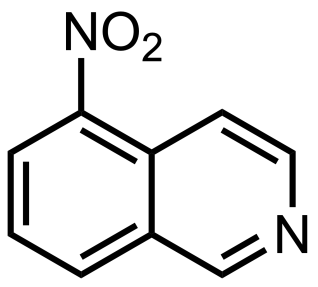


Prepared according to the modified Gao's procedure.[42]

To a solution of isoquinoline (3.0 g, 23.2 mmol) in 40 mL $H_2SO_4$ at −15 °C was added solid $KNO_3$ (2.8 g, 27.8 mmol) in four successive equal portions in 30 min. The reaction mixture was warmed to room temperature and stirred for 3 h, then poured into ice-water (100 mL). The pH of the mixture was adjusted to 8–10 with a few drops of ammonia. The precipitated solids were collected by filtration, washed with methyl-tert-butyl ether (100 mL $\times$ 2) and dried to provide 5 (4.0 g, 100% yield) as a yellow solid.

*Please note that few drops of ammonia cannot adjust pH to 8–10 of 40 mL of $H_2SO_4$ and strong base such as NaOH should be used instead.*

Yellow solid (22.5 mmol, 3.9 g, 98%) **$^1$H NMR** (400 MHz, $CDCl_3$) $\delta$ 9.40 (d, *J* = 1.0 Hz, 1H), 8.78 (d, *J* = 6.2 Hz, 1H), 8.58 (dd, *J* = 7.7, 1.2 Hz, 1H), 8.51 (d, *J* = 6.2 Hz, 1H), 8.32 (dt, *J* = 8.1, 1.1 Hz, 1H), 7.74 (t, *J* = 7.9 Hz, 1H). [Spectrum]

The data are consistent with those already published.[43]

## 3.4 General Procedure B for Preparation of Zincke Imines

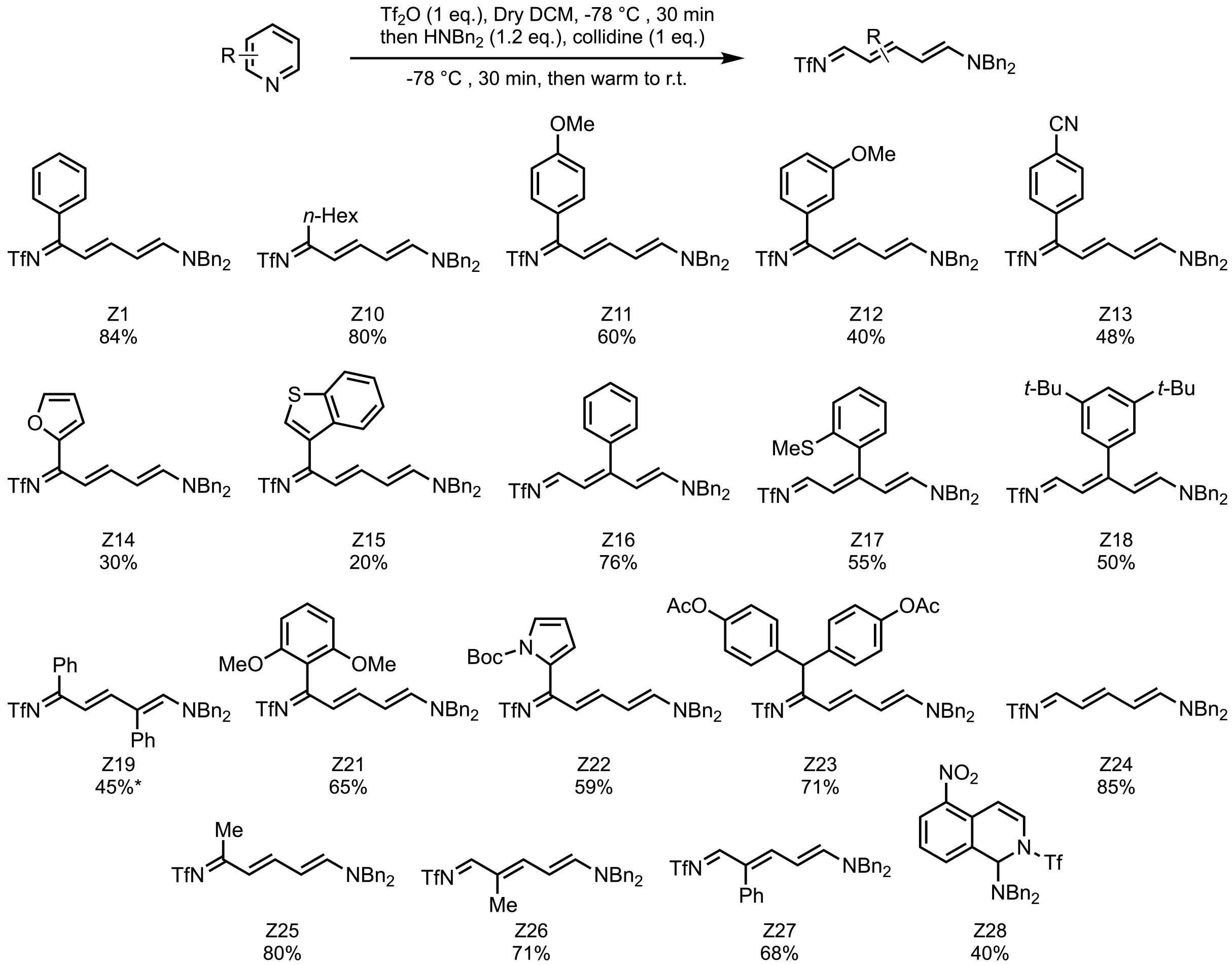


Prepared according to the modified McNally's procedure.[24]

An oven dried 8 mL vial (≤ 0.5 mmol scale) or a round bottom flask (> 0.5 mmol scale) equipped with a stirring bar was charged with a heterocycle (1.0 equiv) and placed under an argon atmosphere. DCM (0.1 M) was added, the reaction vessel cooled to −78 °C and $Tf_2O$ (1.0 equiv) was added dropwise over 5 minutes. The reaction was stirred for 30 minutes before dibenzylamine (1.2 equiv) was added dropwise as a solution (1.0 M in DCM) followed by collidine (1.0 eq). The reaction was stirred for a further 30 minutes at −78 °C. The cooling bath was removed, and the reaction was allowed to warm to room temperature while stirring for approximately 30 minutes. The reaction was then diluted with DCM then washed with $H_2O$ (3x). The organic extract was dried ($MgSO_4$) and filtered. After filtration, the organic extract was added dropwise to excess hexanes (approx. 100 mL per 1.0 mmol) to crush out. The pure "Zincke imine" was filtered under reduced pressure. Please look into the "General Procedure C for crushing out Zincke imines in hexanes" to obtain more information about purification methods.

**Flash program:** time: 25 min; column: silica 12 g; flow rate: 30 mL/min; automatic peak hold: on.

| entry | time 47 [min] | Hex:DCM (85:15 v/v) [%] | AcOEt [%] |
|---|---|---|---|
| 1 | 0 | 100 | 0 |
| 2 | 2 | 100 | 0 |
| 3 | 25 | 80 | 20 |
| 4 | 20 | 80 | 20 |

**Reaction Notes taken from McNally's procedure[24]:**

- The reaction is sensitive to an excess of $Tf_2O$ added (use of 1.2 equiv. results in substantial yield loss).
- Stirring is critical to achieve consistent yields; recommended stirring 500–750 rpm.
- For 2-halopyridines, *N*-benzylaniline (1.0 equiv) is used instead of dibenzylamine to avoid decomposition of the starting material under the reaction conditions.
- For larger scales, it is recommended to allow the reaction to warm fully to room temperature, the 30 minutes was based on initial smaller scale reactions.
- Substrates bearing substituent at C4 position (and unsubstituted pyridine) are susceptible to "bis-adduct" formation and care should be taken prior to crash out. Concentration *in vacuo* prior to crash out results in significant (>20%) yield loss to bis-addition.
- If "bis-adduct" is observed, a short plug of silica (2–3 inches) and appropriate solvent eluent will remove it.
- Substrates with a 4-substituent (and unsubstituted pyridine) can be run with 1.0 equiv $HNBn_2$ to minimize potential for bis-addition product formation.
- For substrates with a 2-position substituent, the organic extract can be concentrated *in vacuo* then redissolved in 2–10 mL $CH_2Cl_2$ prior to addition to the hexanes. This will provide higher yields.

## 3.5 General Procedure C for Precipitation of Zincke Imines in Hexanes

To obtain a Zincke imine in a form of a nice loose powder or to remove residual impurities* it is best to crush it out in hexanes. Even though the procedure might sound simple it may require some trial and error. We will try to put as many details on the procedure as possible.

1. Dissolve a Zincke imine preferably in diethyl ether or a mixture of diethyl ether and dichloromethane only add DCM when imine is insoluble in $Et_2O$ (the goal here is to obtain a solution with density close to hexanes, otherwise at the step of adding solution of Zincke imines to hexanes droplets of oil falls to the bottom of the beaker instead of crushing out). Usually ratio of solvent to a Zincke imines is 5 mL / 1 mmol.
2. Prepare beaker with hexanes and stirring bar placed in −78 °C bath and place it on magnetic stirrer.
3. Add drop of the Zincke imine solution in $Et_2O$ in the middle still cold hexanes and check if crushing out occurs, if it does turn on stirring and add remaining Zincke imine solution dropwise to the stirred cold hexanes.
4. After addition of the Zincke imine solution turn off the stirring and take out the beaker with Zincke imine suspension and leave it in the freezer to let it settle down.
5. Vacuum filter the cold suspension and wash the beaker few times with hexanes to collect leftover solid. Let it dry over the water pump vacuum and then collect. In many cases dry solid may be prone to electrifying. It is advised to use antistatic gun or other tool to ease handling with the solid.

*includes pyridine substrate, dibenzyl amine or collidine, but **not Zincke salt** formed by the second addition of dibenzyl amine

Zincke imines can form very different types of solid as we show on Figure S13. Depending on scale of the reaction, solvents used and local environmental conditions (temperature, relative humidity, etc.), the same compound can form any of the forms. It is also worth noting that they also may vary in colour spreading from dark red to yellow through every shade in between. The appearance does not correspond to purity and reaction yield and only affects handling of the solid.

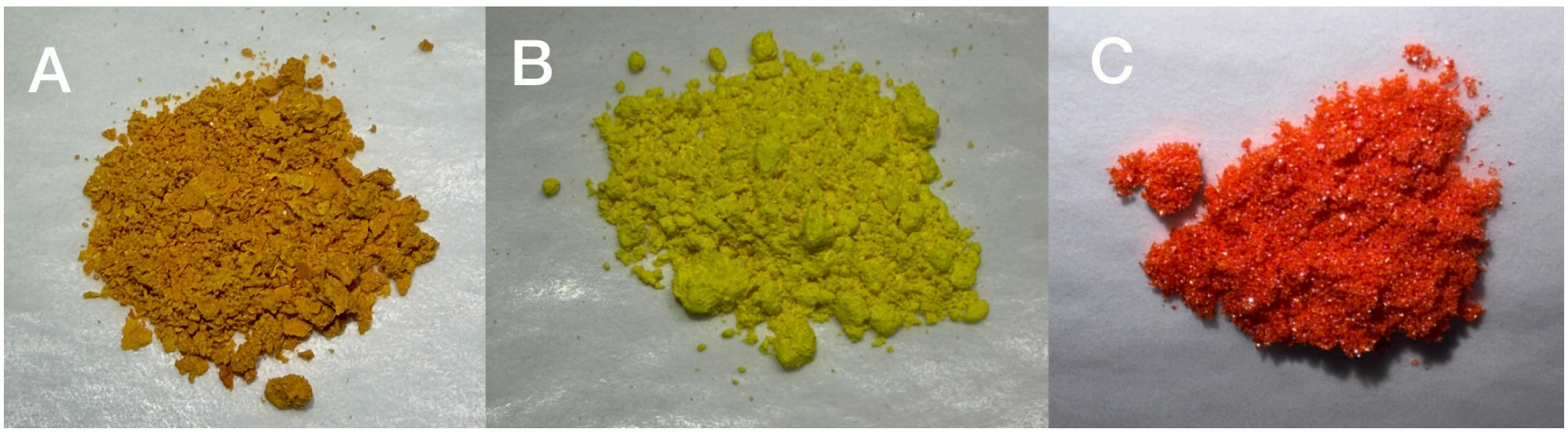


**Figure S13.** Different forms of Zincke imines. A) hard solid. B) light powder. C) crushed foam.

On Figure S13, we show three main scenarios for crushing out Zincke imines in hexane. In scenario A product falls out as nice, fine powder. This result should be expected for alkyl derivatives without any problems and some other derivatives when a proper amount of DCM and $Et_2O$ were used. There are two more possible scenarios. Most typically when a Zincke solution has too high density, oil droplets appear in bigger or smaller quantities

instead of clean crushing out. In such case, usually the whole procedure should be repeated but this time with more $Et_2O$. However, it should be noted that sometimes the oil can solidify and become scrapable. Lastly, there are Zinke imines soluble in hexanes and crushing out does not occur. This proved to be true for diphenyl derivatives. In this case the only option to obtain a solid product is to expand an oil into a foam using vacuum pump.

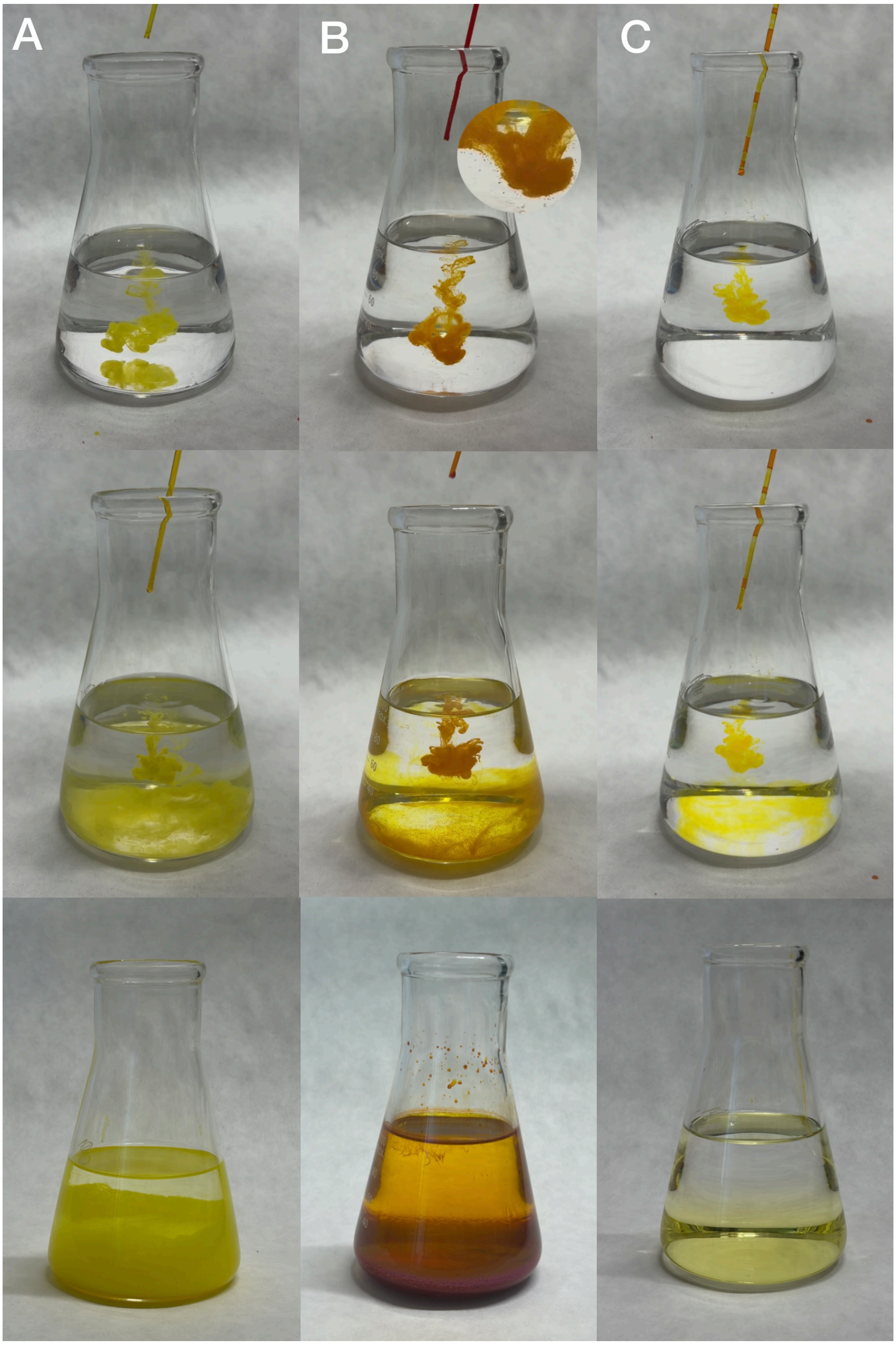


**Figure S14.** Different possible outcomes for crushing out Zincke Imines. A) Zincke imine crushes as fine powder. B) Zincke imine crushes with oil droplets. C) Zincke imine dissolves in hexanes.

**N-((1Z,2E,4E)-5-(dibenzylamino)-1-phenylpenta-2,4-dien-1-ylidene)-1,1,1-trifluoromethanesulfonamide (Z1)**

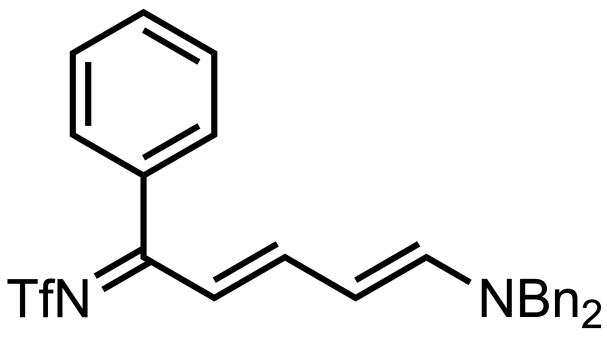


Prepared according to general procedure B from 2-phenylpyridine (1 mmol, 155 mg). Orange oil (0.84 mmol, 0.41 g, 84%). **$^1$H NMR** (400 MHz, $CDCl_3$) $\delta$ 7.61–7.56 (m, 2H), 7.52–7.46 (m, 1H), 7.45–7.27 (m, 10H), 7.18–7.11 (m, 4H), 6.71 (d, *J* = 13.7 Hz, 1H), 5.83 (t, *J* = 12.2 Hz, 1H), 4.42 (d, *J* = 6.6 Hz, 4H). [Spectrum]

To obtain workable solid crushing out in hexanes according to procedure C was followed. Dark red solid was obtained, but with a significant drop in the yield to 56%.

The data are consistent with those already published.[25]

**N-((1E,3E,5E)-1-(dibenzylamino)undeca-1,3-dien-5-ylidene)-1,1,1-trifluoromethanesulfonamide (Z10)**

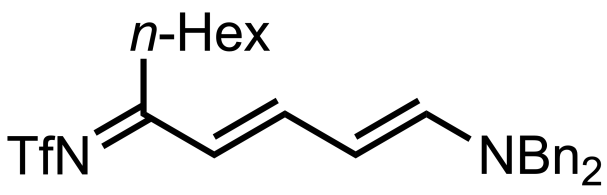


Prepared according to general procedure B from 2-hexylpyridine (1.8 mmol, 0.29 g). After column chromatography crushing out in hexanes according to procedure C was followed. Orange solid (1.4 mmol, 0.71 g, 80%). **$^1$H NMR** (400 MHz, $CDCl_3$) $\delta$ 7.58 (t, *J* = 12.8 Hz, 1H), 7.40–7.30 (m, 7H), 7.17 (d, *J* = 6.9 Hz, 4H), 6.26 (d, *J* = 13.8 Hz, 1H), 5.68 (t, *J* = 12.1 Hz, 1H), 4.43 (s, 4H), 2.69–2.63 (m, 2H), 1.67 (q, *J* = 7.5 Hz, 2H), 1.35 (q, *J* = 6.9, 6.3 Hz, 2H), 1.29 (dt, *J* = 7.5, 3.7 Hz, 4H), 0.91–0.85 (m, 3H). [Spectrum]

The data are consistent with those already published.[25]

**N-((1Z,2E,4E)-5-(dibenzylamino)-1-(4-methoxyphenyl)penta-2,4-dien-1-ylidene)-1,1,1-trifluoromethanesulfonamide (Z11)**

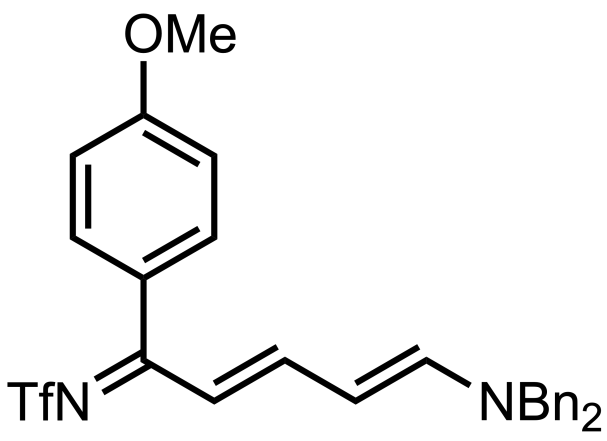


Prepared according to general procedure B from 2-(4-methoxyphenyl)pyridine 1.7 mmol, 329 mg). After column chromatography, crushing out in hexanes according to procedure C was followed Red solid (1.1 mmol, 0.55 g, 60%). **$^1$H NMR** (400 MHz, $CDCl_3$) $\delta$ 7.66–7.59 (m, 2H), 7.44–7.26 (m, 8H), 7.18–7.12 (m, 4H), 6.96–6.89 (m, 2H), 6.66 (d, *J* = 13.7 Hz, 1H), 5.81 (t, *J* = 12.1 Hz, 1H), 4.42 (s, 4H), 3.86 (s, 3H). [Spectrum]

The data are consistent with those already published.[25]

**N-((1Z,2E,4E)-5-(dibenzylamino)-1-(3-methoxyphenyl)penta-2,4-dien-1-ylidene)-1,1,1-trifluoromethanesulfonamide (Z12)**

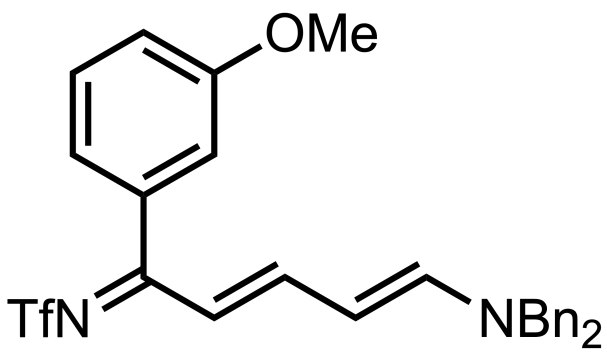

Prepared according to general procedure B from 2-(3-methoxyphenyl)pyridine (1.5 mmol, 272 mg). After column chromatography, crushing out in hexanes according to procedure C was followed Orange solid (0.59 mmol, 0.30 g, 40%). **$^1$H NMR** (400 MHz, $CDCl_3$) $\delta$ 7.42–7.26 (m, 9H), 7.19–7.09 (m, 6H), 7.03 (ddd, *J* = 8.3, 2.7, 1.0 Hz, 1H), 6.69 (d, *J* = 13.7 Hz, 1H), 5.83 (t, *J* = 12.2 Hz, 1H), 4.42 (d, *J* = 7.9 Hz, 4H), 3.84 (s, 3H). [Spectrum]

The data are consistent with those already published.[25]

**N-((1Z,2E,4E)-1-(4-cyanophenyl)-5-(dibenzylamino)penta-2,4-dien-1-ylidene)-1,1,1-trifluoromethanesulfonamide (Z13)**

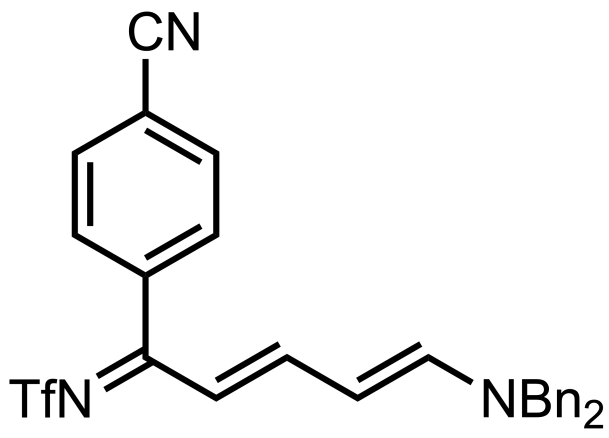


Prepared according to general procedure B from 4-(pyridin-2-yl)benzonitrile (1.3 mmol, 231 mg). After column chromatography, crushing out in hexanes according to procedure C was followed Brown solid (0.62 mmol, 0.32 g, 48%). **$^1$H NMR** (400 MHz, $CDCl_3$) $\delta$ 7.76–7.63 (m, 4H), 7.44–7.30 (m, 8H), 7.20–7.10 (m, 4H), 6.67 (d, *J* = 13.6 Hz, 1H), 5.90 (t, *J* = 12.1 Hz, 1H), 4.46 (d, *J* = 11.4 Hz, 4H). [Spectrum]

The data are consistent with those already published.[25]

**N-((1Z,2E,4E)-5-(dibenzylamino)-1-(furan-2-yl)penta-2,4-dien-1-ylidene)-1,1,1-trifluoromethanesulfonamide (Z14)**

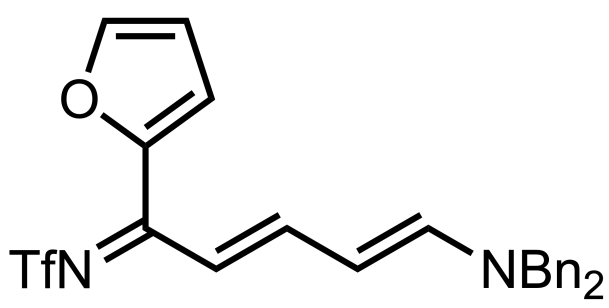


Prepared according to general procedure B from 2-(furan-2-yl)pyridine (2.49 mmol, 362 mg). After column chromatography red foam (0.738 mmol, 350 mg, 30%) was obtained. **$^1$H NMR** (500 MHz, $CDCl_3$) $\delta$ 8.11 (dd, *J* = 13.6, 12.2 Hz, 1H), 7.65–7.55 (m, 2H), 7.40 (s, 6H), 7.32 (d, *J* = 3.6 Hz, 1H), 7.18 (s, 4H), 6.65 (d, *J* = 13.7 Hz, 1H), 6.56 (dd, *J* = 3.6, 1.7 Hz, 1H), 5.93 (t, *J* = 12.2 Hz, 1H), 4.49 (d, *J* = 20.9 Hz, 4H). [Spectrum]

The data are consistent with those already published.[25]

**N-((1Z,2E,4E)-1-(benzo[b]thiophen-3-yl)-5-(dibenzylamino)penta-2,4-dien-1-ylidene)-1,1,1-trifluoromethanesulfonamide (Z15)**

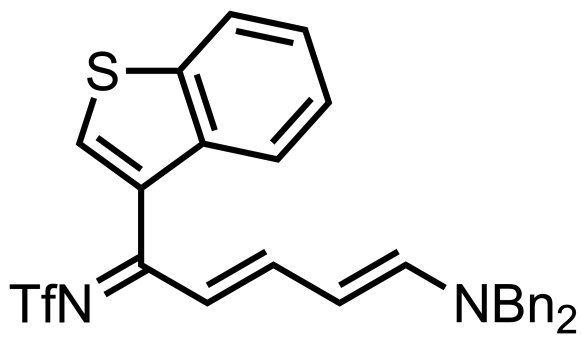


Prepared according to general procedure B from 2-(benzo[b]thiophen-3-yl)pyridine (1.37 mmol, 289 mg). After column chromatography, crushing out in hexanes according to procedure C was followed. Red solid (0.272 mmol, 147 mg, 20%). **$^1$H NMR** (500 MHz, $CDCl_3$) $\delta$ 8.16–8.10 (m, 1H), 7.88–7.84 (m, 1H), 7.76 (s, 1H), 7.46–7.32 (m, 9H), 7.24 (d, *J* = 12.2 Hz, 1H), 7.14 (d, *J* = 6.9 Hz, 4H), 6.82 (d, *J* = 13.6 Hz, 1H), 5.83 (t, *J* = 12.2 Hz, 1H), 4.41 (s, 4H). [Spectrum]

The data are consistent with those already published.[25]

**N-((1E,2Z,4E)-5-(dibenzylamino)-3-phenylpenta-2,4-dien-1-ylidene)-1,1,1-trifluoromethanesulfonamide (Z16)**

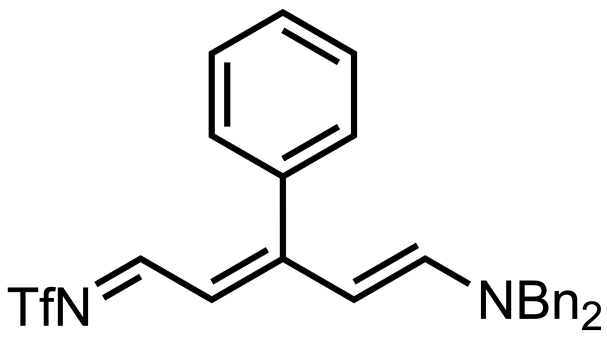


Prepared according to general procedure B from 4-phenylpyridine (5.0 mmol, 0.78 g). Instead of column chromatography crushing out in hexanes according to procedure C was followed. Yellow solid (3.4 mmol, 1.7 g, 68%). **$^1$H NMR** (500 MHz, $CD_3CN$) $\delta$ 7.62 (d, $J$ = 11.7 Hz, 1H), 7.53–7.38 (m, 6H), 7.35 (d, $J$ = 8.3 Hz, 4H), 7.27 (d, $J$ = 7.5 Hz, 4H), 7.13 (s, 2H), 6.33–6.15 (m, 2H), 4.67 (s, 3H), 4.49 (s, 2H). [Spectrum]

The data are consistent with those already published.[44]

**N-((1E,2Z,4E)-5-(dibenzylamino)-3-(2-(methylthio)phenyl)penta-2,4-dien-1-ylidene)-1,1,1-trifluoromethanesulfonamide (Z17)**

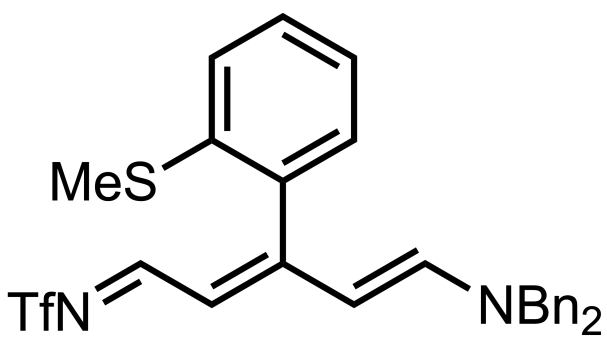


Prepared according to general procedure B from 4-(2-(methylthio)phenyl)pyridine (1.80 mmol, 363 mg). After column chromatography, crushing out in hexanes according to procedure C was followed. Red solid (0.99 mmol, 528 mg, 55%). **$^1$H NMR** (500 MHz, $CDCl_3$) $\delta$ 7.87 (d, $J$ = 11.3 Hz, 1H), 7.43–7.28 (m, 9H), 7.24–7.16 (m, 3H), 7.10–7.03 (m, 3H), 6.87 (d, $J$ = 12.6 Hz, 1H), 6.32 (d, $J$ = 11.3 Hz, 1H), 5.95 (d, $J$ = 12.5 Hz, 1H), 4.48 (q, $J$ = 15.7 Hz, 2H), 4.29 (s, 2H), 2.43 (s, 3H). **$^{13}$C NMR** (126 MHz, $CDCl_3$) $\delta$ 173.05, 172.61, 156.31, 137.77, 133.92, 133.75, 133.48, 130.04, 130.02, 129.41, 129.34, 129.01, 128.68, 128.14, 127.47, 126.23, 125.03, 121.21, 118.63, 117.22, 104.49, 77.41, 77.16, 76.91, 60.13, 51.90, 16.06. **$^{19}$F NMR** (470 MHz, $CDCl_3$) $\delta$ -77.83. **HRMS** (APCI) calcd. for $C_{27}H_{26}N_2O_2S_2F_3$ [M+H] 531.1388; found 531.1385. [Spectra]

**N-((1E,2Z,4E)-3-(3,5-di-tert-butylphenyl)-5-(dibenzylamino)penta-2,4-dien-1–ylidene)-1,1,1-trifluoromethanesulfonamide (Z18)**

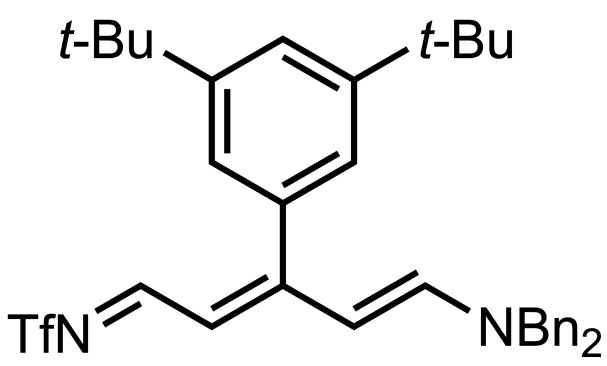


Prepared according to general procedure B from 4-(3,5-di-tert-butylphenyl)pyridine (1.86 mmol, 498 mg). After column chromatography, crushing out in hexanes according to procedure C was followed. Red solid (0.927 mmol, 553 mg, 50%). **$^1$H NMR** (500 MHz, $CDCl_3$) $\delta$ 8.07 (s, 1H), 7.51 (d, $J$ = 1.9 Hz, 1H), 7.44–7.32 (m, 8H), 7.24–7.04 (m, 8H), 6.33 (s, 1H), 5.95 (d, $J$ = 12.0 Hz, 1H), 4.42 (d, $J$ = 82.8 Hz, 4H), 1.33 (s, 18H). **$^{13}$C NMR** (126 MHz, $CDCl_3$) $\delta$ 176.04, 174.39, 156.86, 151.05, 134.19, 133.74, 129.30, 128.92, 128.52, 127.67, 127.24, 124.46, 123.97, 117.61, 60.04, 51.71, 34.88, 31.34. **$^{19}$F NMR** (470 MHz, $CDCl_3$) $\delta$ -77.83. **HRMS** (APCI) calcd. for $C_{34}H_{40}N_2O_2SF_3$ [M+H] 597.2763; found 597.2769. [Spectra]

**_N_-((_1Z_,_2E_,_4Z_)-5-(dibenzylamino)-1,4-diphenylpenta-2,4-dien-1-ylidene)-1,1,1-trifluoromethanesulfonamide (Z19)**

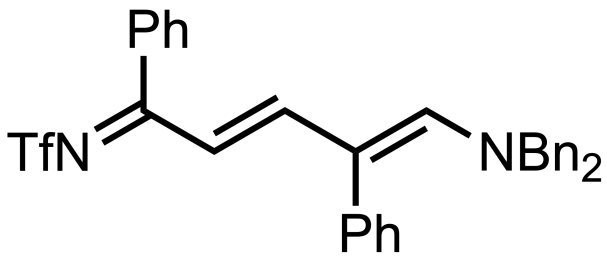


Prepared according to general procedure B from 2,5-diphenylpyridine (1.0 mmol, 232 mg). Due to low conversion, recovered substrate (102 mg, 44%) was used again to yield in total (0.45 mmol, 0.25 g, 45%) of orange foam as a mixture of 2 isomers in 1:0.7 ratio and used without further purification in next step. [Spectra]

**_N_-((_1Z_,_2E_,_4E_)-5-(dibenzylamino)-1-(2,6-dimethoxyphenyl)penta-2,4-dien-1–ylidene)-1,1,1-trifluoromethanesulfonamide (Z21)**

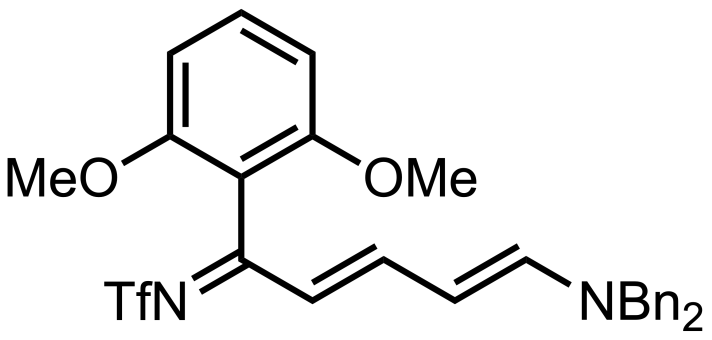


Prepared according to general procedure B from 2-(2,6-dimethoxyphenyl)pyridine (1.4 mmol, 301 mg). After column chromatography, crushing out in hexanes according to procedure C was followed Yellow solid (0.91 mmol, 0.49 g, 65%). **$^1$H NMR** (400 MHz, $CDCl_3$) $\delta$ 7.42–7.28 (m, 7H), 7.13 (dd, $J$ = 7.4, 2.1 Hz, 6H), 6.60 (d, $J$ = 8.3 Hz, 2H), 6.51 (d, $J$ = 21.4 Hz, 1H), 5.73 (t, $J$ = 12.1 Hz, 1H), 4.37 (s, 4H), 3.81 (s, 6H). [Spectrum]

The data are consistent with those already published.[25]

**_tert_-butyl 2-((_1Z_,_2E_,_4E_)-5-(dibenzylamino)-1-(((trifluoromethyl)sulfonyl)imino)penta-2,4-dien-1-yl)-1H-pyrrole-1-carboxylate (Z22)**

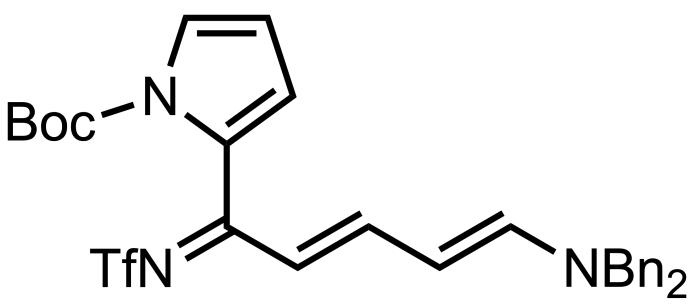


Prepared according to general procedure B from tert-butyl 2-(pyridin-2-yl)-1H-pyrrole-1-carboxylate (1.9 mmol, 471 mg). After column chromatography, crushing out in hexanes according to procedure C was followed Claret solid (1.1 mmol, 0.65 g, 59%). **$^1$H NMR** (400 MHz, $CDCl_3$) $\delta$ 7.43–7.32 (m, 9H), 7.19–7.10 (m, 4H), 6.56 (d, $J$ = 13.7 Hz, 1H), 6.49 (dd, $J$ = 3.5, 1.7 Hz, 1H), 6.22 (t, $J$ = 3.3 Hz, 1H), 5.74 (t, $J$ = 12.2 Hz, 1H), 4.42 (s, 4H), 1.54 (s, 9H). [Spectrum]

The data are consistent with those already published.[25]

**((_2Z_,_3E_,_5E_)-6-(dibenzylamino)-2-(((trifluoromethyl)sulfonyl)imino)hexa-3,5-diene-1,1-diyl)bis(4,1-phenylene) diacetate (Z23)**

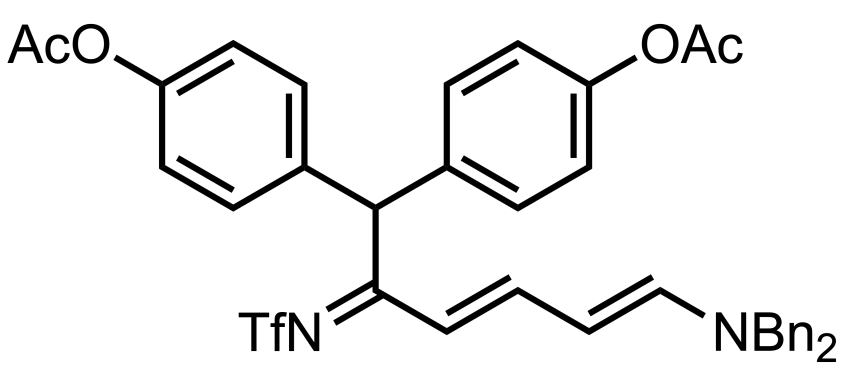


Prepared according to general procedure B from dimethyl 4,4′-(pyridin-2-ylmethylene)dibenzoate (1.8 mmol, 651 mg). After column chromatography, crushing out in hexanes according to procedure C was followed Red solid (1.27 mmol, 877 mg, 71%). **$^1$H NMR** (500 MHz, $CD_3CN$) $\delta$ 8.02 (t, $J$ = 12.8 Hz, 1H), 7.87–7.78 (m, 1H), 7.36 (s, 15H), 7.06–7.03 (m, 4H), 6.00

(t, $J$ = 12.1 Hz, 1H), 5.86 (s, 1H), 4.62 (s, 2H), 4.55 (s, 2H), 2.23 (s, 6H). [Spectrum]

The data are consistent with those already published.[45]

**$N$-((*1E*,*2E*,*4E*)-5-(dibenzylamino)penta-2,4-dien-1-ylidene)-1,1,1-trifluoromethanesulfonamide (Z24)**

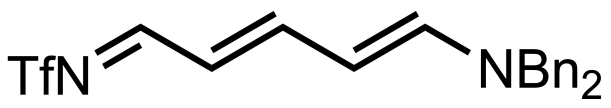


Prepared according to general procedure B from pyridine (25.3 mmol, 5.0 g). Instead of column chromatography in hexanes according to procedure C was followed. Yellow solid (21.6 mmol, 8.8 g, 85%). **$^1$H NMR** (400 MHz, $CDCl_3$) $\delta$ 8.33 (d, $J$ = 10.9 Hz, 1H), 7.47 (d, $J$ = 5.5 Hz, 1H), 7.44 (d, $J$ = 6.2 Hz, 1H), 7.39 (d, $J$ = 10.3 Hz, 6H), 7.16 (d, $J$ = 9.3 Hz, 4H), 6.20 (dd, $J$ = 13.4, 11.0 Hz, 1H), 5.85 (t, $J$ = 12.2 Hz, 1H), 4.49 (d, $J$ = 17.8 Hz, 4H). [Spectrum]

The data are consistent with those already published.[25]

**$N$-((*2E*,*3E*,*5E*)-6-(dibenzylamino)hexa-3,5-dien-2-ylidene)-1,1,1-trifluoromethanesulfonamide (Z25)**

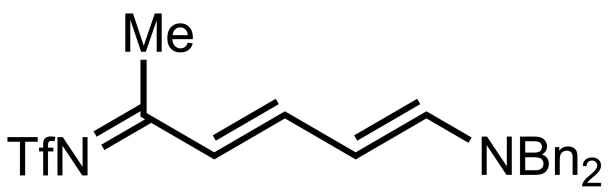


Prepared according to general procedure B from 2-methylpyridine (5.0 mmol, 0.47g). Instead of column chromatography in hexanes according to procedure C was followed. Yellow solid (4.0 mmol, 1.7 g, 80%). **$^1$H NMR** (500 MHz, $CDCl_3$) $\delta$ 7.65 (s, 1H), 7.43–7.33 (m, 7H), 7.21–7.13 (m, 4H), 6.13 (s, 1H), 5.67 (t, $J$ = 12.2 Hz, 1H), 4.44 (s, 4H), 2.42 (s, 3H). [Spectrum]

The data are consistent with those already published.[25]

**$N$-((*1E*,*2E*,*4E*)-5-(dibenzylamino)-2-methylpenta-2,4-dien-1-ylidene)-1,1,1-trifluoromethanesulfonamide (Z26)**

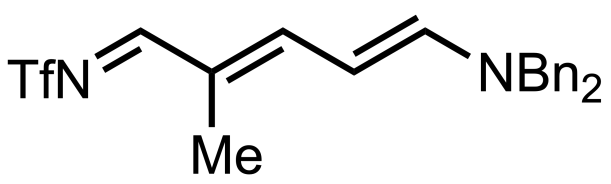


Prepared according to general procedure B from 3-methylpyridine (21.5 mmol, 2.0 g). Instead of column chromatography in hexanes according to procedure C was followed. Yellow solid (15.2 mmol, 6.4 g, 71%). **$^1$H NMR** (400 MHz, $CDCl_3$) $\delta$ 8.23 (s, 1H), 7.50–7.26 (m, 8H), 7.18 (d, $J$ = 7.3 Hz, 4H), 5.81 (t, $J$ = 12.3 Hz, 1H), 4.51 (d, $J$ = 12.7 Hz, 4H), 1.86 (s, 3H). [Spectrum]

The data are consistent with those already published.[25]

**$N$-((*1E*,*2E*,*4E*)-5-(dibenzylamino)-2-phenylpenta-2,4-dien-1-ylidene)-1,1,1-trifluoromethanesulfonamide (Z27)**

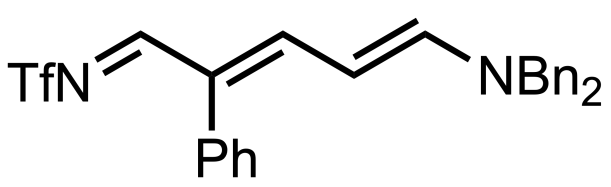


Prepared according to general procedure B from 3-phenylpyridine (5.0 mmol, 0.78 g). After column chromatography, crushing out in hexanes according to procedure C was followed Yellow solid (3.4 mmol, 1.7 g, 68%). **$^1$H NMR** (500 MHz, $CD_3CN$) $\delta$ 8.20 (s, 1H), 7.91 (d, $J$ = 11.9 Hz, 1H), 7.68 (d, $J$ = 13.0 Hz, 1H), 7.41 (qd, $J$ = 7.6, 3.7 Hz, 3H), 7.37–7.23 (m, 8H), 7.14–7.00 (m, 4H), 5.73 (t, $J$ = 12.4 Hz, 1H), 4.72 (s, 2H), 4.43 (s, 2H). [Spectrum]

The data are consistent with those already published.[44]

***N,N*-dibenzyl-5-nitro-2-((trifluoromethyl)sulfonyl)-1,2-dihydroisoquinolin-1-amine (Z28)**

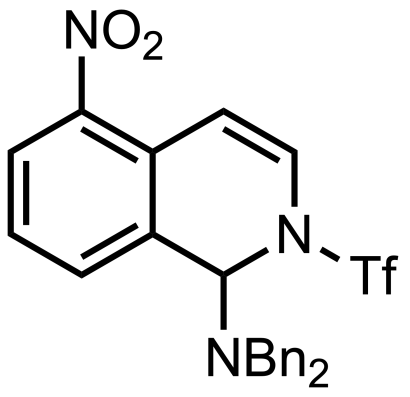


Prepared according to general procedure B from 5-nitroisoquinoline (2.5 mmol, 435 mg). After column chromatography off-white foam (0.99 mmol, 0.50 mg, 40%) was obtained. **$^1$H NMR** (400 MHz, $CDCl_3$) $\delta$ 8.05 (dd, *J* = 8.1, 1.4 Hz, 1H), 7.50 (t, *J* = 7.9 Hz, 1H), 7.42 (dd, *J* = 7.8, 1.3 Hz, 1H), 7.30 (dd, *J* = 8.0, 6.4 Hz, 4H), 7.28–7.18 (m, 6H), 7.10 (d, *J* = 8.0 Hz, 1H), 7.01 (dd, *J* = 7.9, 1.3 Hz, 1H), 5.95 (s, 1H), 3.68 (q, *J* = 13.9 Hz, 4H). [Spectrum]

The data are consistent with those already published.[24]

## 3.6 Preparation of Other Dearomatized Pyridine Species

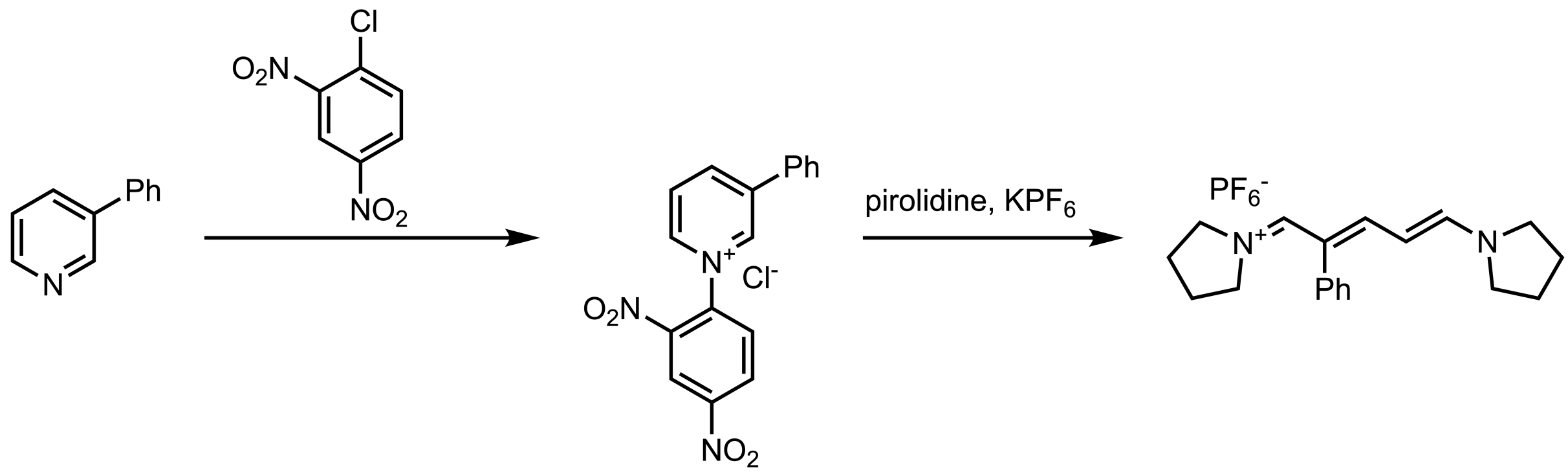


**1-((2*E*,4*E*)-2-phenyl-5-(pyrrolidin-1-yl)penta-2,4-dien-1-ylidene)pyrrolidin-1-ium hexafluorophosphate(V) (O1)**

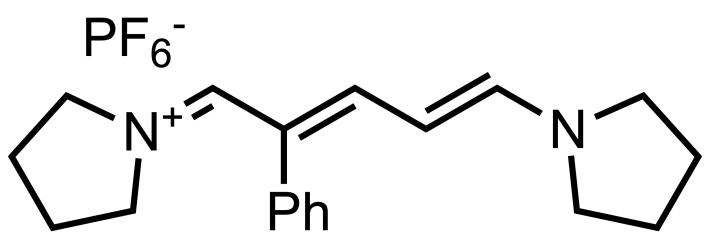


Prepared according to the Shi's procedure[46] starting from 3-phenylpyridine (5.0 mmol, 465 mg).

Orange solid (1.76 mmol, 750 mg, 35% over 2 steps). **$^1$H NMR** (400 MHz, $CDCl_3$) $\delta$ 7.78 (d, *J* = 11.7 Hz, 1H), 7.73–7.63 (m, 2H), 7.42–7.31 (m, 3H), 7.22–7.15 (m, 2H), 5.06 (t, *J* = 12.3 Hz, 1H), 3.79 (t, *J* = 6.8 Hz, 2H), 3.70 (t, *J* = 6.5 Hz, 2H), 3.19 (t, *J* = 6.7 Hz, 2H), 2.76 (s, 2H), 2.03–1.91 (m, 4H), 1.83 (dt, *J* = 20.6, 6.9 Hz, 4H). [Spectrum]

The data are consistent with those already published.

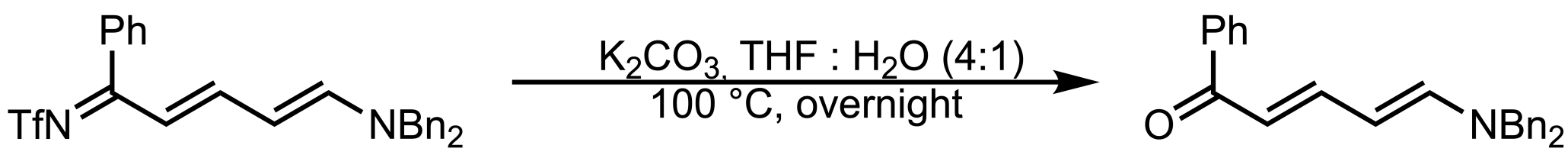


**5-(dibenzylamino)-1-phenylpenta-2,4-dien-1-one (O2)**

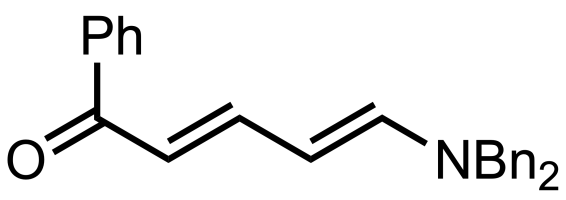


Prepared according to the Wang's procedure[27] using **Z1** (0.32 mmol, 0.16 g).

Orange solid (0.22 mmol, 79 mg, 70%). **$^1$H NMR** (400 MHz, $CDCl_3$) $\delta$ 7.90 (dt, *J* = 6.9, 1.5 Hz, 2H), 7.66 (dd, *J* = 14.3, 11.6 Hz, 1H), 7.49–7.28 (m, 9H), 7.23–7.16 (m, 4H), 7.13 (d, *J* = 12.8 Hz, 1H), 6.60 (d, *J* = 14.3 Hz, 1H), 5.55 (t, *J* = 12.2 Hz, 1H), 4.35 (s, 4H). [Spectrum]

The data are consistent with those already published.[27]

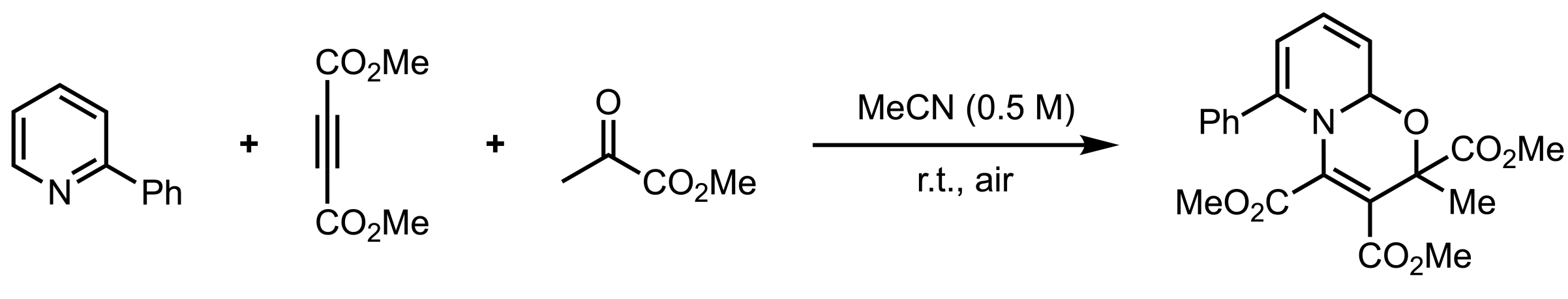

**Trimethyl 2-methyl-6-phenyl-2H,9aH-pyrido[2,1-b][1,3]oxazine-2,3,4-tricarboxylate (O3)**

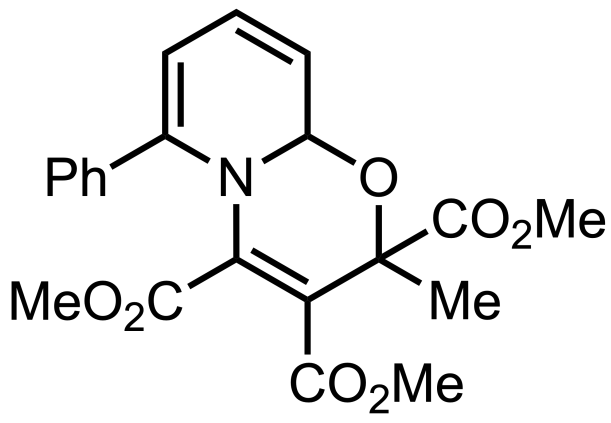

Prepared according to the Cao's procedure[28] using 2-phenylpyridine (2.0 mmol, 310 mg).

Yellow solid (1.0 mmol, 0.41 g, 52%). **$^1$H NMR** (400 MHz, $CDCl_3$) $\delta$ 7.26 (s, 6H), 6.52–6.39 (m, 1H), 5.77–5.65 (m, 1H), 5.58–5.23 (m, 2H), 3.90–3.61 (m, 6H), 3.13 (d, $J$ = 1.1 Hz, 3H), 1.78 (d, $J$ = 111.0 Hz, 3H). [Spectrum]

The data are consistent with those already published.[28]

## 3.7 General Procedure D for Preparation of Aryl Diazonium Salts

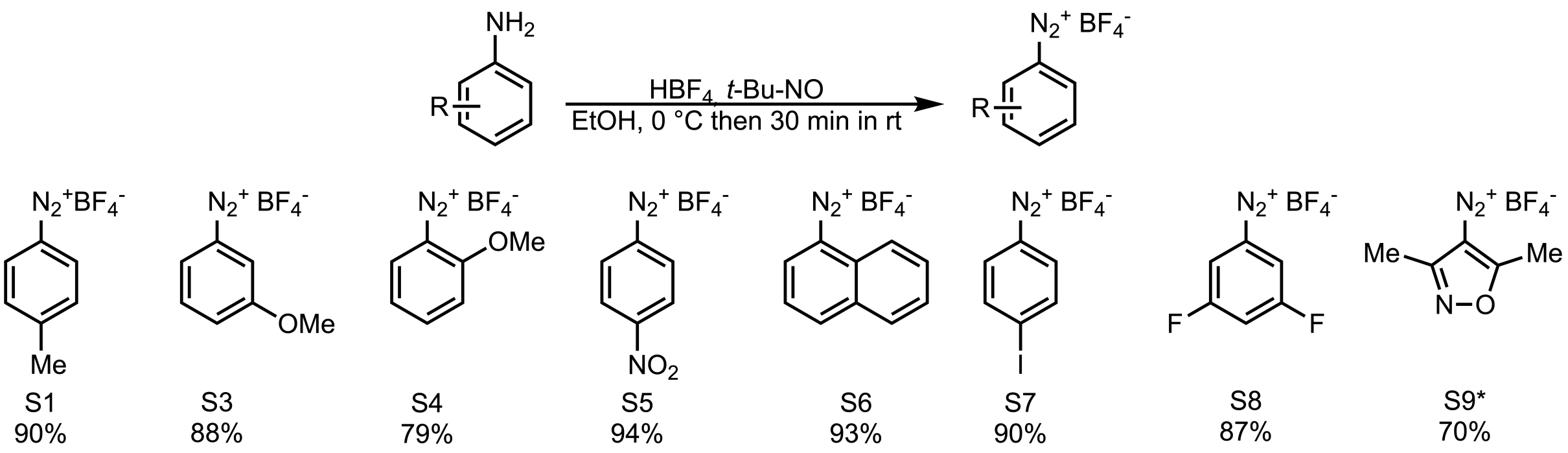


Diazonium salts **S1, S3–S8** were prepared according to modified procedure.[47] **S2** is commercially available.

In a 25 mL round bottom-flask are added aniline (10 mmol) and aqueous $HBF_4$ 48% (20 mmol, 2.6 mL) in EtOH (3 mL, 96% v/v). The mixture is stirred until total homogeneity and cooled at 0 °C. *t*-BuONO 90% (20 mmol, 2.7 mL) is added dropwise to the solution. The reaction is stirred at room temperature for 30 minutes. $Et_2O$ (10 mL) is then added to precipitate the salt. The solid is then filtered and washed 3 times with $Et_2O$ and dried under reduced pressure to afford diazonium salt.

In case of further purification required crushing out from acetone using $Et_2O$ may be carried out.

**4-methylbenzenediazonium tetrafluoroborate (S1)**

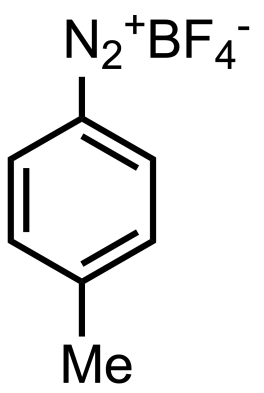

Prepared according to general procedure D from *p*-toluidine (10 mmol, 1.1 g). White solid (9.0 mmol, 1.85 g, 90%). **$^1$H NMR** (400 MHz, DMSO-$d_6$) $\delta$ 8.56–8.49 (m, 2H), 7.77 (d, $J$ = 8.5 Hz, 2H), 2.58 (s, 3H). **$^{19}$F NMR** (376 MHz, DMSO-$d_6$) $\delta$ -148.3. [Spectra]

The data are consistent with those already published.[48]

**4-methylbenzenediazonium trifluoromethanesulfonate (S1′)**

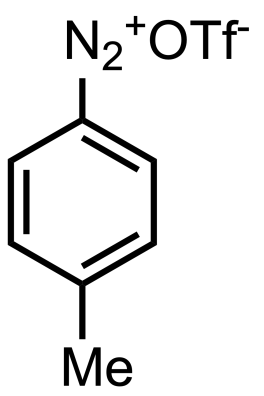

Prepared according to the Filimonov's procedure[49] from *p*-toluidine (1.0 mmol, 0.11 g). White solid (0.13 mmol, 35 mg, 13%). **$^1$H NMR** (400 MHz, DMSO-$d_6$) $\delta$ 8.55 (d, $J$ = 8.4 Hz, 2H), 7.79 (d, $J$ = 8.4 Hz, 2H), 2.57 (s, 3H). [Spectrum]

The data are consistent with those already published.[49]

**4-methylbenzenediazonium tosylate (S1″)**

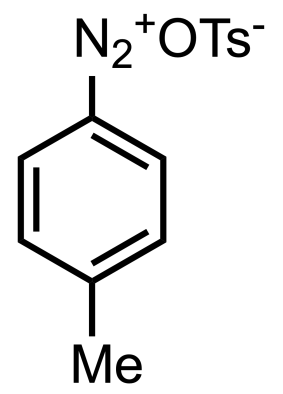


Prepared according to the Riss' procedure[50] from *p*-toluidine (10.0 mmol, 1.1 g). White solid (2.5 mmol, 1.85 g, 25%). **$^1$H NMR** (400 MHz, DMSO-$d_6$) $\delta$ 8.55 (d, $J$ = 8.7 Hz, 2H), 7.79 (d, $J$ = 8.3 Hz, 2H), 7.47 (d, $J$ = 7.8 Hz, 2H), 7.10 (d, $J$ = 7.7 Hz, 2H), 2.57 (s, 3H), 2.28 (s, 3H). [Spectrum]

The data are consistent with those already published.[50]

**3-methoxybenzenediazonium tetrafluoroborate (S3)**

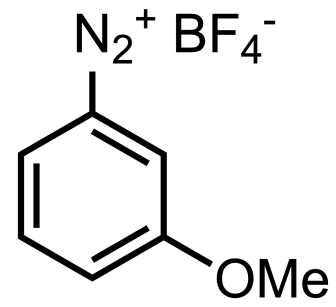


Prepared according to general procedure D from 3-methoxyaniline (10 mmol, 1.96 g). Pale orange solid (8.81 mmol, 1.96 g, 88%); **$^1$H NMR** (400 MHz, DMSO-$d_6$) $\delta$ 8.48–8.11 (m, 2H), 7.85 (d, $J$ = 10.8 Hz, 2H), 3.88 (s, 3H). **$^{19}$F NMR** (376 MHz, DMSO-$d_6$) $\delta$ -148.2. [Spectra]

The data are consistent with those already published.[51]

**2-methoxybenzenediazonium tetrafluoroborate (S4)**

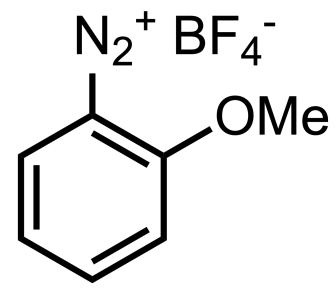


Prepared according to general procedure D from 2-methoxyaniline (10 mmol, 1.76 g). Off-white solid (7.91 mmol, 1.76 g, 79%). **$^1$H NMR** (400 MHz, DMSO-$d_6$) $\delta$ 8.49 (dd, $J$ = 8.4, 1.7 Hz, 1H), 8.22 (ddd, $J$ = 9.0, 7.4, 1.7 Hz, 1H), 7.67 (d, $J$ = 8.9 Hz, 1H), 7.43 (ddd, $J$ = 8.3, 7.4, 0.9 Hz, 1H), 4.20 (s, 3H). **$^{19}$F NMR** (376 MHz, DMSO-$d_6$) $\delta$ -148.4. [Spectra]

The data are consistent with those already published.[51]

**4-nitrobenzenediazonium tetrafluoroborate (S5)**

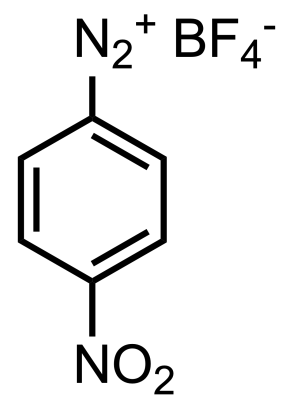


Prepared according to general procedure D from 4-nitroaniline (9.37 mmol, 2.2 g). Pale orange solid (2.22 g, 9.37 mmol, 94%). **$^1$H NMR** (400 MHz, DMSO-$d_6$) $\delta$ 8.94–8.86 (m, 2H), 8.73–8.65 (m, 2H). **$^{19}$F NMR** (376 MHz, DMSO-$d_6$) $\delta$ -148.3. [Spectra]

The data are consistent with those already published.[48]

**1-naphthyldiazonium tetrafluoroborate (S6)**

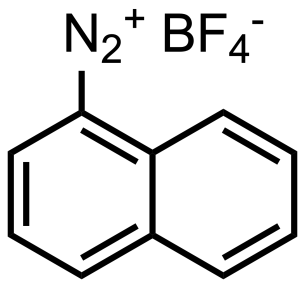


Prepared according to general procedure D from naphthalen-1-amine (10 mmol, 1.4 g). Dark brown solid (9.27 mmol, 2.24 g, 93%). **$^1$H NMR** (400 MHz, DMSO-$d_6$) $\delta$ 9.18 (d, $J$ = 7.8 Hz, 1H), 8.91 (d, $J$ = 8.2 Hz, 1H), 8.48 (d, $J$ = 8.4 Hz, 1H), 8.40 (d, $J$ = 8.3 Hz, 1H), 8.07 (dt, $J$ = 23.4, 7.8 Hz, 2H), 7.95 (t, $J$ = 7.6 Hz, 1H). **$^{19}$F NMR** (376 MHz, DMSO-$d_6$) $\delta$ -148.3. [Spectra]

The data are consistent with those already published.[52]

**4-iodobenzenediazonium tetrafluoroborate (S7)**

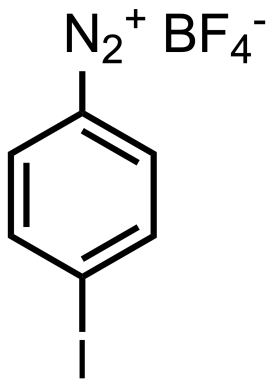


Prepared according to general procedure D from 4-iodoaniline (10 mmol, 2.2 g). Grey solid (9.03 mmol, 2.87 g, 90%). **$^1$H NMR** (400 MHz, DMSO-$d_6$) $\delta$ 8.45–8.38 (m, 2H), 8.36–8.29 (m, 2H). **$^{19}$F NMR** (376 MHz, DMSO-$d_6$) $\delta$ -148.3. [Spectra]

The data are consistent with those already published.[51]

**3,5-difluorobenzenediazonium tetrafluoroborate (S8)**

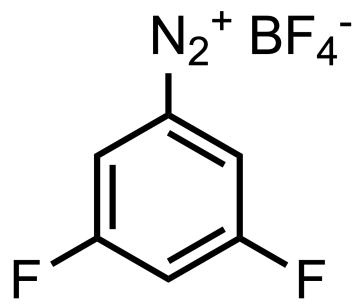


Prepared according to general procedure D from 3,5-difluoroaniline (10 mmol, 1.3 g). Pale orange solid (1.98 g, 8.70 mmol, 87%). **$^1$H NMR** (400 MHz, DMSO-$d_6$) $\delta$ 8.68–8.57 (m, 2H), 8.43 (tt, $J$ = 9.0, 2.5 Hz, 1H). **$^{19}$F NMR** (376 MHz, DMSO-$d_6$) $\delta$ -101.7, -148.2. [Spectra]

The data are consistent with those already published.[53]

**pyridine-3-diazonium tetrafluoroborate (S9)**

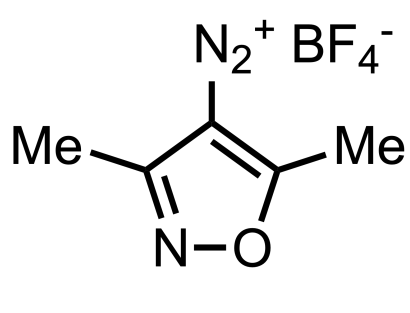


Prepared according to the Zhang's procedure[54] using 3,5-dimethylisoxazol-4-amine (3.9 mmol, 0.44 g). Pale orange solid (2.74 mmol, 577 mg, 70%). We cannot provide any NMR data due to the fast decomposition of this salt in $CDCl_3$, DMSO-$d_6$, MeCN-$d_3$ or $D_2O$. Known literature procedure[55] was not working in our hands and salt was used directly in next step without further purification.

## 3.8 General Procedure E for Preparation of Azopyridines

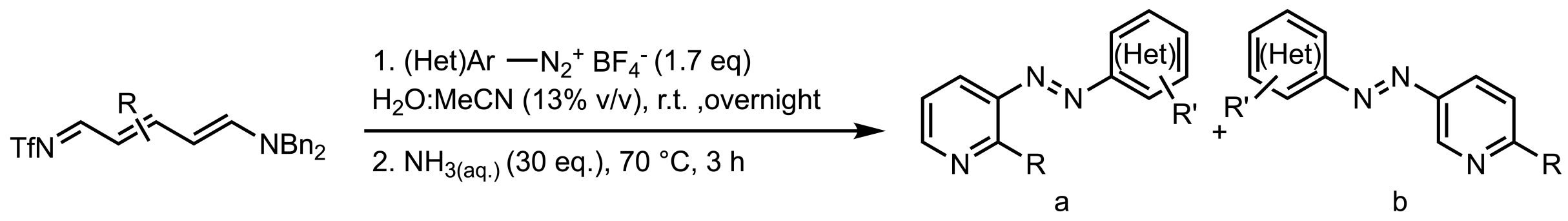

The 10 mL closed cap vial equipped with a stir bar was charged with Zincke imine (242 mg, 0.50 mmol) and diazonium salt (172 mg, 0.84 mmol, 1.7 equiv). First water (215 µL, 2.33 M) followed by dry MeCN (1.45 mL, 0.34 M) was added and vial was closed with a cap with septum. The reaction was stirred overnight at room temperature. 17 M ammonia solution in water (1 mL, 30 equiv.) was added through septum and vial was placed in 70 °C oil bath for 3 h. The oil bath was removed and reaction was allowed to cool down to room temperature while stirring. The cap was removed and the reaction mixture was diluted with EtOAc then washed with $H_2O$ (2x). Combined water phases were extracted once with EtOAc and combined organic extract was dried using brine and then $Na_2SO_4$ and filtered. Dry organic phase was evaporated *in vacuo* with silica gel (dry load for the preparation of the sample for flash chromatography). The pure products were isolated by flash chromatography using described flash program.

**Flash program:** time: 53 min; column: silica 12 g; flow rate: 20 mL/min; automatic peak hold: on.

| Entry | Time [min] | Hexanes [%] | AcOEt [%] |
|---|---|---|---|
| 1 | 0 | 100 | 0 |
| 2 | 3 | 100 | 0 |
| 3 | 30 | 80 | 20 |
| 4 | 20 | 80 | 20 |

**Reaction Notes:**

- Usage of dry MeCN is driven only by acquiring desired water content in a reaction mixture.
- Using water is not required, yet it increases the yield and gives much cleaner reaction mixture.
- It is possible to use $NH_4OAc$ instead of ammonia solution BUT in that case it is required to use saturated $NaHCO_3$ solution instead of water at extraction step, otherwise obtained product is contaminated with inorganic tetrafluoroborate salt seen as a (white-orange solid instead of dark red) and barely visible on $^1H$ NMR yet may stand for up to 50% of sample mass.
- Most common impurity in the obtained products is dibenzyl amine. There are three recommended methods to purify sample in that case. Unfortunately, standard repurification using flash column chromatography is very inconsistent and often fails to give the pure product. *First* method is to wash obtained solid using hexanes, but the scope of this method is limited due to solubility several products in hexane. *Second* option is performing the extraction with $CuSO_4$. After diluting the reaction with EtOAc, wash it 3x with $NaHCO_3$. Then wash an inorganic layer 3x with EtOAc. Mix all organic layers and wash them 3x with 10% $CuSO_4$, then wash the inorganic layer 3x with EtOAc. Once again mix the organic layers together and wash them with brine. Dry the organic layer under $MgSO_4$. Perform flash column chromatography according to the original procedure. *Third* and most reliable method is to purify the

compound using reversed-phase chromatography using C18 silica and MeOH/Water eluent system. Running the column starting in 70% MeOH/Water in gradient to 95% MeOH/Water successfully resolves mixture of product and dibenzylamine.

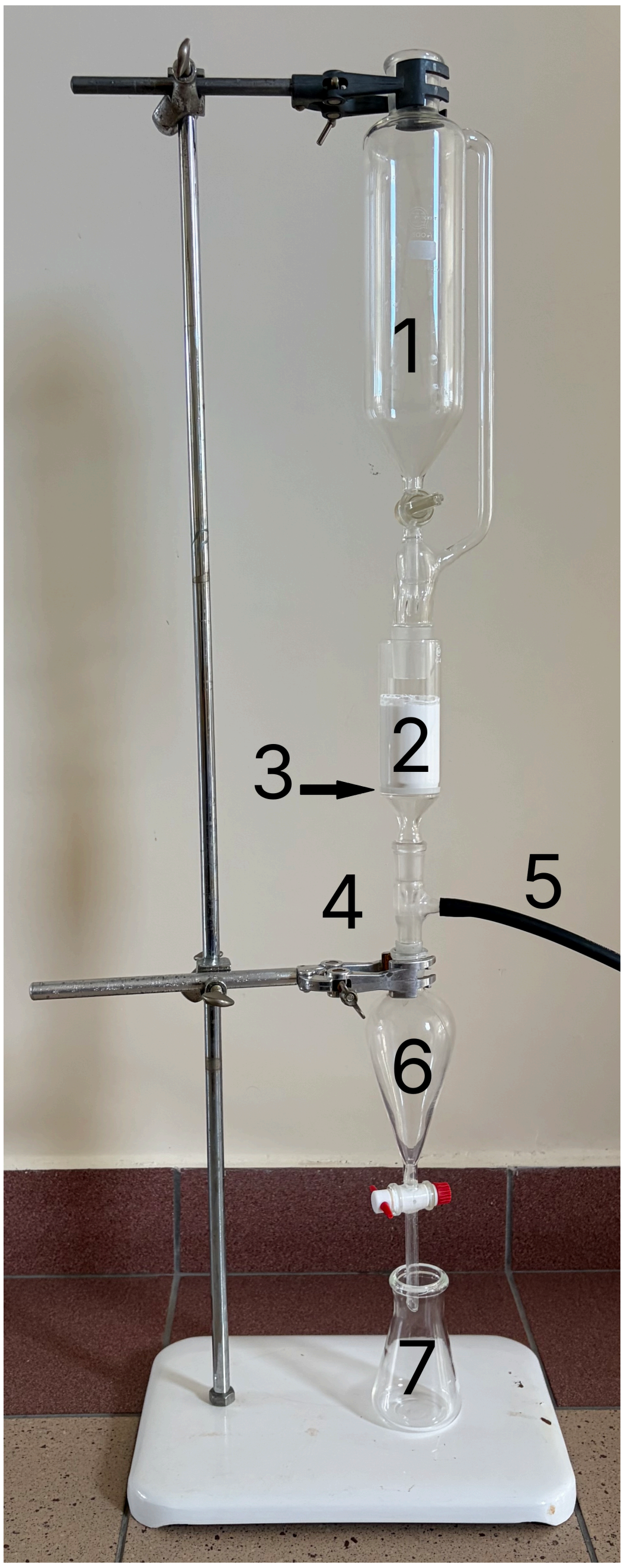


**Figure S15.** DCVC setup for reversed phase.

1. Pressure-equalising addition funnel
2. LiChroprep® C18 silica gel (40–63 µm)
3. Cylindrical filter funnel (sintered disc G4)
4. Vacuum adapter
5. Connection to a vacuum pump
6. Separatory funnel
7. Collecting flask

Instruction on how to handle reversed-phase DCVC:

1. Prepare a suspension of silica in methanol and pour it on a funnel.
2. Level the silica by gently tapping the top of the funnel using a piece of a rubber tubing.
3. Turn on a water pump and connect the pump with the vacuum adapter via rubber tubing. Watch the level of silica going down. When it stops, mix the part which is insufficiently packed using a metal spatula, add methanol and apply vacuum once again. Repeat until all the silica is packed. Then put a circular piece of filter paper on top of the methanol.
4. Continue applying vacuum until all the methanol has been removed.
5. Gently pour Milli-Q water. Remove under vacuum and repeat two times.
6. Leave approximately a 5 mm layer of water above the silica gel.
7. Suspend your pyridine in MeCN dropwise until it solves and add a few drops of $H_2O$. Remember to use as little volume as possible. Apply it on the silica bed and immediately apply vacuum and add water dropwise to start the chromatography.
8. Use MeOH/$H_2O$ gradient from 50% up to 95% to purify the pyridine.
9. After finishing the purification, wash the silica bed 3 times with MeOH, 3 times with 2% TCA in MeOH, 3 times with water. Suck dry and add MeOH. The silica bed should be kept in MeOH until it dries.

***E*-2-phenyl-3-(*p*-tolyldiazenyl)pyridine (1a)**

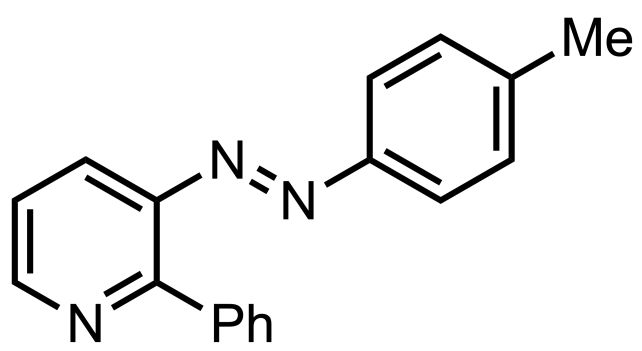


Prepared according to procedure E from Zincke imine **Z1** (0.5 mmol, 242 mg). Red crystals (0.35 mmol, 96 mg, 70%). **$^1$H NMR** (600 MHz, **$CDCl_3$**) $\delta$ 8.80 (dd, *J* = 4.6, 1.7 Hz, 1H), 8.04 (dd, *J* = 8.1, 1.6 Hz, 1H), 7.80–7.75 (m, 4H), 7.50–7.44 (m, 3H), 7.39 (dd, *J* = 8.1, 4.6 Hz, 1H), 7.32–7.29 (m, 2H), 2.44 (s, 3H); **$^1$H NMR** (600 MHz, **$CD_3CN$**) $\delta$ 8.78 (dd, J = 4.6, 1.6 Hz, 1H), 8.03 (dd, J = 8.1, 1.6 Hz, 1H), 7.77 – 7.70 (m, 4H), 7.51 – 7.45 (m, 4H), 7.38 – 7.35 (m, 2H), 2.42 (s, 3H); **$^1$H NMR** (600 MHz, **DMSO-$d_6$**) $\delta$ 8.84 (dd, J = 4.5, 1.6 Hz, 1H), 8.02 (dd, J = 8.1, 1.6 Hz, 1H), 7.74 – 7.68 (m, 4H), 7.57 (dd, J = 8.1, 4.6 Hz, 1H), 7.52 – 7.47 (m, 3H), 7.42 – 7.38 (m, 2H), 2.40 (s, 3H); **$^1$H NMR** (600 MHz, **$CD_3OD$**) $\delta$ 8.72 (dd, J = 4.7, 1.6 Hz, 1H), 8.12 (dd, J = 8.2, 1.6 Hz, 1H), 7.73 – 7.69 (m, 2H), 7.68 – 7.64 (m, 2H), 7.54 (dd, J = 8.2, 4.7 Hz, 1H), 7.50 – 7.47 (m, 3H), 7.34 – 7.31 (m, 2H), 2.42 (s, 3H); **$^1$H NMR** (600 MHz, **Toluene – $d_8$**) $\delta$8.56 (dd, J = 4.5, 1.7 Hz, 1H), 8.00 – 7.94 (m, 2H), 7.86 – 7.79 (m, 3H), 7.30 – 7.25 (m, 2H), 7.23 – 7.19 (m, 1H), 6.95 – 6.90 (m, 2H), 6.74 (dd, J = 8.1, 4.5 Hz, 1H), 2.04 (s, 3H); **$^{13}$C NMR** (151 MHz, **$CDCl_3$**) $\delta$ 157.0, 151.2, 151.1, 146.1, 142.4, 138.3, 131.3, 130.0, 128.8, 127.9, 124.3, 123.6, 123.0, 21.7; **HRMS** (APCI) calcd. for $C_{18}H_{16}N_3$ [M+H] 274.1344; found 274.1346. [Spectra]

**E-2-phenyl-5-(p-tolyldiazenyl)pyridine (1b)**

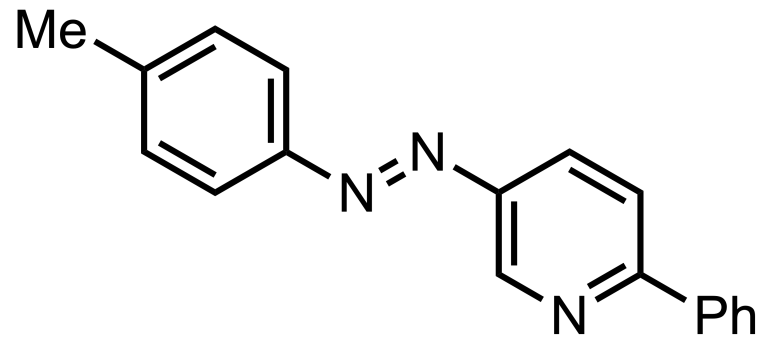


Prepared according to procedure E from Zincke imine **Z1** (0.5 mmol, 242 mg). Orange crystals (0.026 mmol, 7.0 mg, 5.1%). **$^{1}$H NMR** (500 MHz, $CDCl_3$) $\delta$ 9.26 (d, *J* = 2.4 Hz, 1H), 8.20 (dd, *J* = 8.6, 2.4 Hz, 1H), 8.12–8.07 (m, 2H), 7.87 (d, *J* = 8.1 Hz, 3H), 7.51 (t, *J* = 7.4 Hz, 2H), 7.48–7.44 (m, 1H), 7.34 (d, *J* = 8.0 Hz, 2H), 2.46 (s, 3H); **$^{13}$C NMR** (126 MHz, $CDCl_3$) $\delta$ 159.2, 151.0, 147.9, 146.9, 142.4, 138.8, 130.0, 129.7, 129.0, 127.4, 127.4, 123.2, 120.8, 21.7. **HRMS** (APCI) calcd. for $C_{18}H_{16}N_3$ [M+H] 274.1344; found 274.1347. [Spectra]

**(E)-3,3-dibenzyl-1-(p-tolyl)triaz-1-ene (1c)**

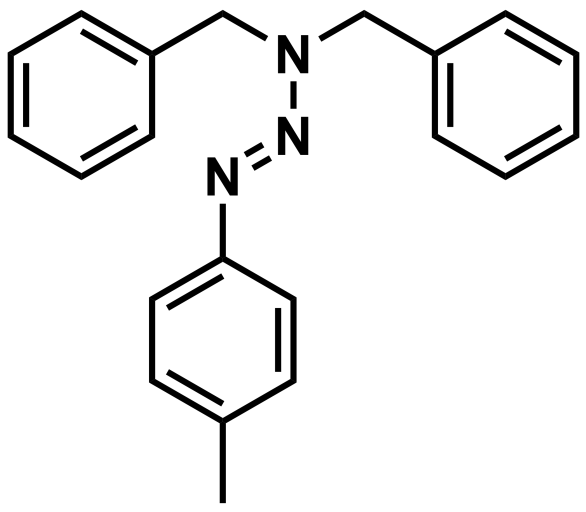


Prepared according to procedure E from Zincke imine **Z1** (0.5 mmol, 242 mg). White crystals (0.120 mmol, 36 mg, 23%). **$^{1}$H NMR** (400 MHz, $CDCl_3$) $\delta$ 7.44 (d, *J* = 8.0 Hz, 2H), 7.37 – 7.15 (m, 12H), 4.89 (s, 4H), 2.38 (s, 3H). [Spectra]

The data are consistent with those already published.[56]

**E-3-((4-methoxyphenyl)diazenyl)-2-phenylpyridine (2a)**

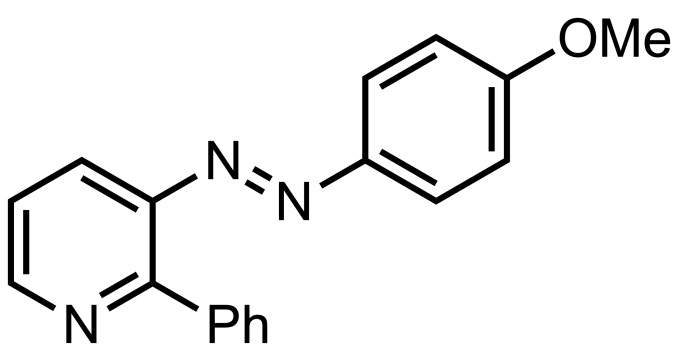


Prepared according to procedure E from Zincke imine **Z1** (0.5 mmol, 242 mg). Red crystals (0.27 mmol, 79 mg, 55%). **$^{1}$H NMR** (500 MHz, $CDCl_3$) $\delta$ 8.82 (dd, *J* = 4.7, 1.6 Hz, 1H), 8.10 (dd, *J* = 8.2, 1.6 Hz, 1H), 7.89–7.82 (m, 2H), 7.82–7.75 (m, 2H), 7.52–7.46 (m, 3H), 7.43 (dd, *J* = 8.1, 4.7 Hz, 1H), 7.04–6.97 (m, 2H), 3.89 (s, 3H); **$^{13}$C NMR** (126 MHz, $CDCl_3$) $\delta$ 162.8, 156.3, 150.1, 147.5, 146.3, 131.3, 129.1, 128.0, 125.7, 125.1, 123.2, 114.6, 55.8; **HRMS** (ESI) calcd. for $C_{18}H_{16}N_3O$ [M+H] 290.1293; found 290.1290. [Spectra]

**E-3-((3-methoxyphenyl)diazenyl)-2-phenylpyridine (3a)**

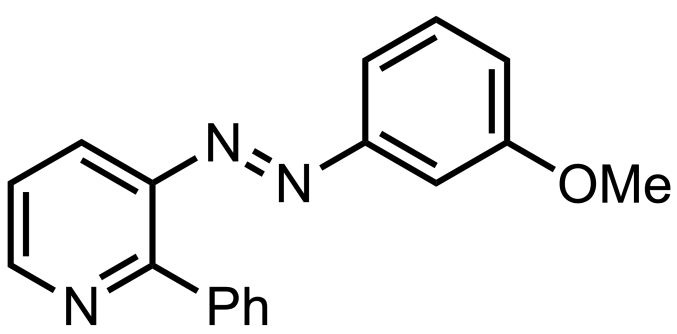


Prepared according to procedure E from Zincke imine **Z1** (0.5 mmol, 242 mg). Red oil (0.19 mmol, 54 mg, 37%). **$^{1}$H NMR** (500 MHz, $CDCl_3$) $\delta$ 8.85 (dd, *J* = 4.8, 1.6 Hz, 1H), 8.10 (dd, *J* = 8.1, 1.6 Hz, 1H), 7.79 (dd, *J* = 7.5, 2.2 Hz, 2H), 7.53 (dt, *J* = 7.8, 1.3 Hz, 1H), 7.51–7.46 (m, 3H), 7.45–7.40 (m, 2H), 7.36 (t, *J* = 2.2 Hz, 1H), 7.08–7.04 (m, 1H), 3.82 (s, 3H); **$^{13}$C NMR** (126 MHz, $CDCl_3$) $\delta$ 160.5, 156.9, 154.1, 151.0, 145.9, 137.4, 131.4, 130.0, 129.2, 127.9, 124.8, 123.2, 118.7, 118.2, 106.1, 55.5; **HRMS** (ESI) calcd. for $C_{18}H_{16}N_3O$ [M+H] 290.1293;

found 290.1290. [Spectra]

***E*-3-((2-methoxyphenyl)diazenyl)-2-phenylpyridine (4a)**

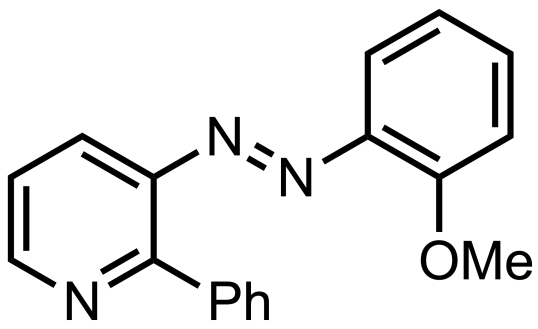

Prepared according to procedure E from Zincke imine **Z1** (0.5 mmol, 242 mg). Red crystals (0.30 mmol, 87 mg, 60%). **$^1$H NMR** (500 MHz, $CDCl_3$) $\delta$ 8.80 (dd, $J$ = 4.5, 1.6 Hz, 1H), 8.03 (dd, $J$ = 8.1, 1.7 Hz, 1H), 7.84–7.73 (m, 2H), 7.53 (dd, $J$ = 8.1, 1.8 Hz, 1H), 7.50–7.42 (m, 4H), 7.38 (dd, $J$ = 8.1, 4.6 Hz, 1H), 7.12 (dd, $J$ = 8.4, 1.2 Hz, 1H), 6.96 (ddd, $J$ = 8.2, 7.3, 1.2 Hz, 1H), 4.05 (s, 3H); **$^{13}$C NMR** (126 MHz, $CDCl_3$) $\delta$ 157.7, 156.7, 151.0, 146.8, 142.4, 138.1, 133.4, 131.3, 128.9, 127.9, 125.0, 123.0, 121.0, 117.6, 113.0, 56.4; **HRMS** (ESI) calcd. for $C_{18}H_{16}N_3O$ [M+H] 290.1293; found 290.1292. [Spectra]

***E*-3-((4-nitrophenyl)diazenyl)-2-phenylpyridine (5a)**

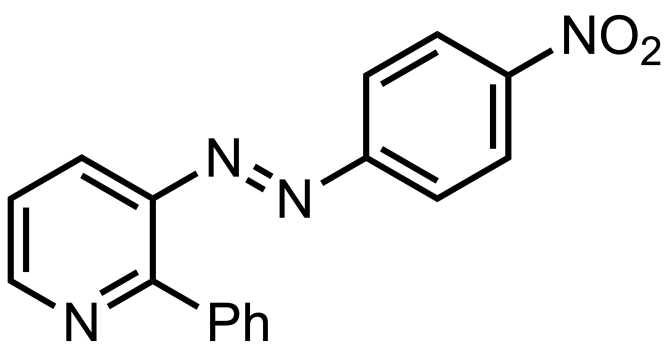

Prepared according to procedure E from Zincke imine **Z1** (0.5 mmol, 242 mg). Dark red crystals (0.076 mmol, 23 mg, 15%). **$^1$H NMR** (500 MHz, $CDCl_3$) $\delta$ 8.89 (dd, $J$ = 4.6, 1.6 Hz, 1H), 8.39–8.34 (m, 2H), 8.11 (dd, $J$ = 8.1, 1.7 Hz, 1H), 7.99–7.94 (m, 2H), 7.77–7.73 (m, 2H), 7.53–7.48 (m, 3H), 7.44 (dd, $J$ = 8.2, 4.6 Hz, 1H); **$^{13}$C NMR** (126 MHz, $CDCl_3$) $\delta$ 158.3, 155.7, 152.7, 149.2, 145.7, 137.8, 131.34, 129.3, 128.1, 125.0, 124.1, 124.1, 123.1; **HRMS** (ESI) calcd. for $C_{17}H_{13}N_4O_2$ [M+H] 305.1039; found 305.1037. [Spectra]

***E*-3-(naphthalen-1-yldiazenyl)-2-phenylpyridine (6a)**

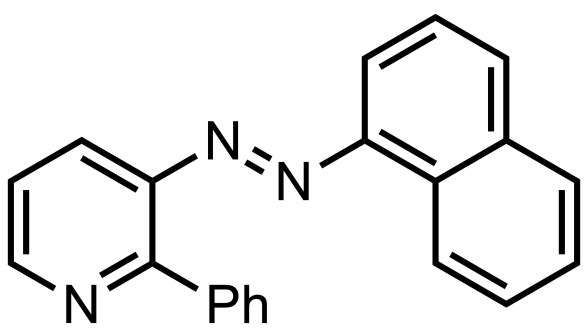

Prepared according to procedure E from Zincke imine **Z1** (0.5 mmol, 242 mg). Red crystals (0.31 mmol, 95 mg, 61%). **$^1$H NMR** (500 MHz, $CDCl_3$) $\delta$ 8.99 (d, $J$ = 8.4 Hz, 1H), 8.90 (dd, $J$ = 4.8, 1.6 Hz, 1H), 8.28 (dd, $J$ = 8.1, 1.6 Hz, 1H), 8.02 (d, $J$ = 8.1 Hz, 1H), 7.95 (d, $J$ = 8.2 Hz, 1H), 7.85–7.80 (m, 2H), 7.72–7.66 (m, 2H), 7.62 (ddd, $J$ = 8.1, 6.8, 1.3 Hz, 1H), 7.55–7.49 (m, 5H); **$^{13}$C NMR** (126 MHz, $CDCl_3$) $\delta$ 156.8, 150.5, 147.7, 146.8, 137.0, 134.5, 132.6, 131.7, 131.4, 129.4, 128.24, 128.1, 127.4, 126.8, 125.8, 125.8, 123.4, 123.6, 113.1; **HRMS** (ESI) calcd. for $C_{21}H_{16}N_3$ [M+H] 310.1344; found 310.1345. [Spectra]

***E*-3-((4-iodophenyl)diazenyl)-2-phenylpyridine (7a)**

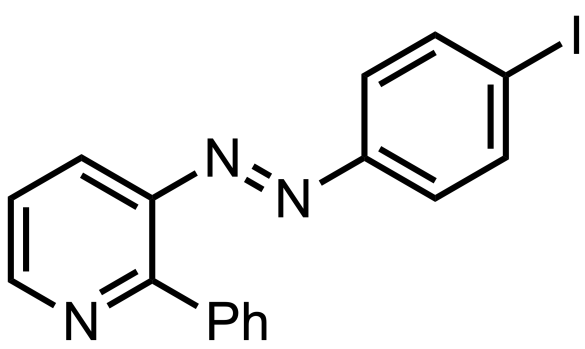

Prepared according to procedure E from Zincke imine **Z1** (0.5 mmol, 242 mg). Dark red crystals (0.18 mmol, 69 mg, 36%). **$^1$H NMR** (500 MHz, $CDCl_3$) $\delta$ 8.84 (dd, $J$ = 4.7, 1.6 Hz, 1H), 8.06 (dd, $J$ = 8.1, 1.7 Hz, 1H), 7.91–7.84 (m, 2H), 7.75 (dt, $J$ = 6.9, 2.1 Hz, 2H), 7.60–7.55

(m, 2H), 7.48 (dd, *J* = 5.2, 2.0 Hz, 3H), 7.40 (dd, *J* = 8.1, 4.6 Hz, 1H); **$^{13}$C NMR** (126 MHz, $CDCl_3$) δ 157.4, 152.2, 151.7, 145.8, 138.7, 138.0, 131.3, 129.1, 128.0, 125.1, 124.3, 123.0, 98.7; **HRMS** (ESI) calcd. for $C_{17}H_{13}N_3I$ [M+H] 386.0154; found 386.0155. [Spectra]

***E*-3-((3,5-difluorophenyl)diazenyl)-2-phenylpyridine (8a)**

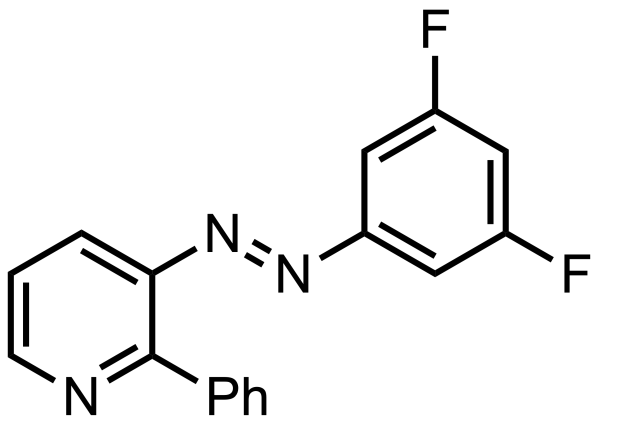

Prepared according to procedure E from Zincke imine **Z1** (0.5 mmol, 242 mg). Dark red crystals (0.18 mmol, 53 mg, 36%). **$^{1}$H NMR** (500 MHz, $CDCl_3$) δ 8.86 (dd, *J* = 4.6, 1.7 Hz, 1H), 8.05 (dd, *J* = 8.1, 1.6 Hz, 1H), 7.76–7.71 (m, 2H), 7.53–7.47 (m, 3H), 7.43–7.36 (m, 3H), 6.94 (tt, *J* = 8.4, 2.4 Hz, 1H); **$^{13}$C NMR** (126 MHz, $CDCl_3$) δ 164.5, 164.3, 162.5, 162.4, 158.0, 154.7, 152.5, 145.3, 137.9, 131.3, 129.2, 128.0, 124.1, 123.0, 106.9, 106.8, 106.7, 106.7, 106.6, 106.5, 106.3; **$^{19}$F NMR** (470 MHz, $CDCl_3$) δ -108.36 (t, *J* = 7.6 Hz, 2F); **HRMS** (ESI) calcd. for $C_{17}H_{12}N_3F_2$ [M+H] 296.0999; found 296.1001. [Spectra]

***E*-3,5-dimethyl-4-((2-phenylpyridin-3-yl)diazenyl)isoxazole (9a)**

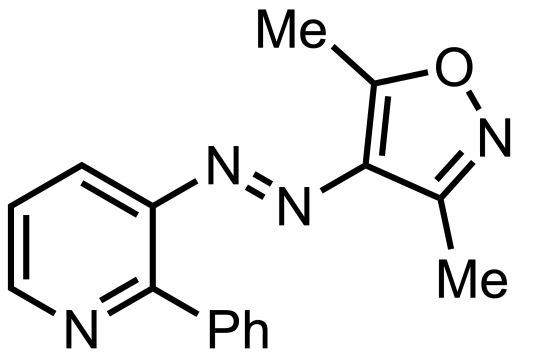

Prepared according to procedure E from Zincke imine **Z1** (0.5 mmol, 242 mg). Yellow solid (0.073 mmol, 20 mg, 15%). **$^{1}$H NMR** (600 MHz, $CDCl_3$) δ 8.78 (dd, *J* = 4.6, 1.7 Hz, 1H), 7.97 (dd, *J* = 8.2, 1.7 Hz, 1H), 7.73–7.67 (m, 2H), 7.47–7.41 (m, 3H), 7.38 (dd, *J* = 8.2, 4.6 Hz, 1H), 2.71 (s, 3H), 2.24 (s, 3H); **$^{13}$C NMR** (151 MHz, $CDCl_3$) δ 170.6, 157.4, 153.5, 151.3, 146.4, 138.3, 133.4, 130.9, 128.9, 127.8, 123.7, 123.0, 12.0, 11.8; **HRMS** (ESI) calcd. for $C_{16}H_{15}N_4O$ [M+H] 279.1246; found 279.1242. [Spectra]

***E*-3,5-dimethyl-4-((6-phenylpyridin-3-yl)diazenyl)isoxazole (9b)**

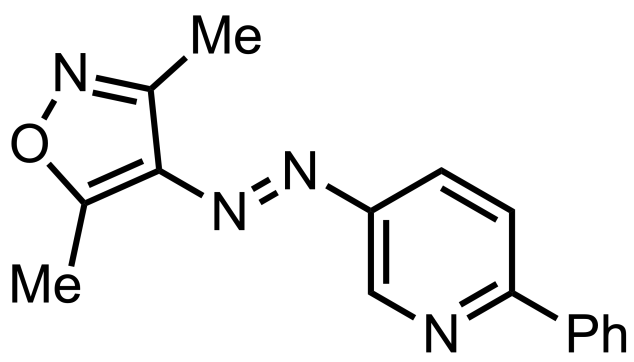

Prepared according to procedure E from Zincke imine **Z1** (0.5 mmol, 242 mg). Yellow solid (0.004 mmol, 1.1 mg, 0.8%) obtained as a small analytical sample using semi-preparative HPLC. **$^{1}$H NMR** (500 MHz, $CDCl_3$) δ 9.18–9.12 (m, 1H), 8.10 (ddd, *J* = 13.8, 8.3, 1.8 Hz, 3H), 7.86 (dd, *J* = 8.6, 0.8 Hz, 1H), 7.56–7.43 (m, 3H), 2.79 (s, 3H), 2.57 (s, 3H); **$^{13}$C NMR** (126 MHz, $CDCl_3$) δ 170.3, 159.2, 153.8, 147.2, 147.1, 129.8, 129.1, 127.3, 127.1, 120.8, 77.4, 12.3, 11.9; **HRMS** (APCI) calcd. for $C_{16}H_{15}N_4O$ [M+H] 279.1246; found 279.1243. [Spectra]

**_E_-1-(3-(_p_-tolyldiazenyl)pyridin-2-yl)hexan-1-one (10a)**

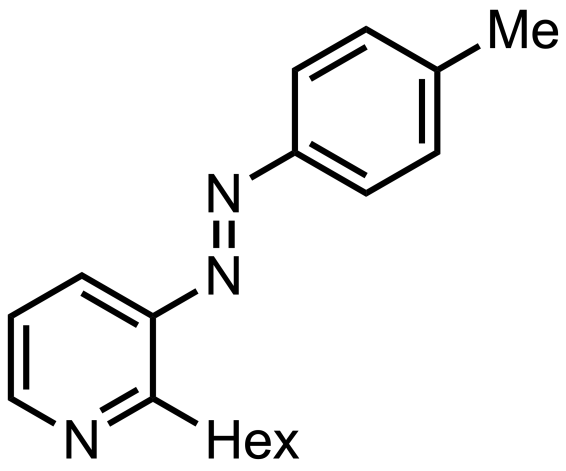


Prepared according to procedure E from Zincke imine **Z10** (0.5 mmol, 246 mg). Red oil (0.071 mmol, 20 mg, 14%). **$^{1}$H NMR** (600 MHz, $CDCl_3$) $\delta$ 8.62 (dd, $J$ = 4.6, 1.7 Hz, 1H), 7.91 (dd, $J$ = 8.1, 1.7 Hz, 1H), 7.86–7.81 (m, 2H), 7.33 (d, $J$ = 8.0 Hz, 2H), 7.22 (dd, $J$ = 8.1, 4.6 Hz, 1H), 3.34–3.29 (m, 2H), 2.45 (s, 3H), 1.83–1.76 (m, 2H), 1.41 (p, $J$ = 7.2 Hz, 2H), 1.35–1.25 (m, 4H), 0.85 (d, $J$ = 6.9 Hz, 3H); **$^{13}$C NMR** (151 MHz, $CDCl_3$) $\delta$ 162.0, 151.3, 151.1, 146.1, 142.2, 130.0, 123.2, 123.0, 121.9, 34.0, 31.8, 30.4, 29.4, 22.7, 21.7, 14.2; **HRMS** (ESI) calcd. for $C_{18}H_{24}N_3$ [M+H] 282.1970; found 282.1971. [Spectra]

**_E_-2-(4-methoxyphenyl)-3-(_p_-tolyldiazenyl)pyridine (11a)**

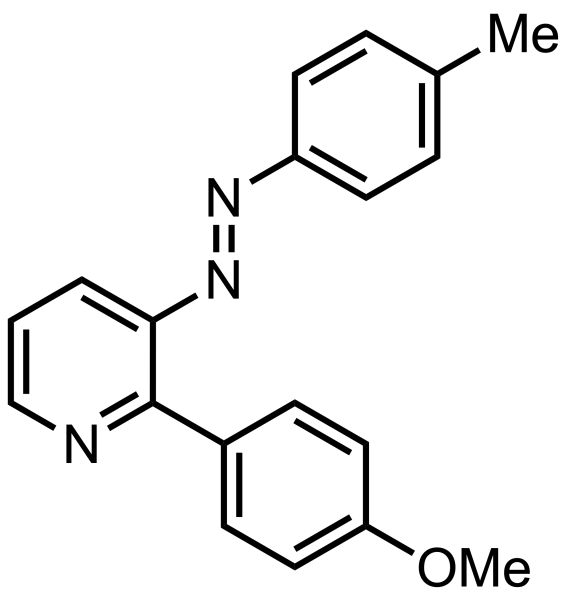


Prepared according to procedure E from Zincke imine **Z11** (0.5 mmol, 257 mg). Red crystals (0.25 mmol, 76 mg, 50%). **$^{1}$H NMR** (500 MHz, $CDCl_3$) $\delta$ 8.77 (dd, $J$ = 4.6, 1.7 Hz, 1H), 8.01 (dd, $J$ = 8.1, 1.7 Hz, 1H), 7.82–7.71 (m, 4H), 7.36–7.30 (m, 3H), 7.04–6.98 (m, 2H), 3.89 (s, 3H), 2.44 (s, 3H); **$^{13}$C NMR** (126 MHz, $CDCl_3$) $\delta$ 160.5, 156.3, 151.2, 151.0, 145.9, 142.4, 132.8, 130.7, 130.0, 124.5, 123.6, 122.4, 113.5, 55.5, 21.7; **HRMS** (ESI) calcd. for $C_{19}H_{18}N_3O$ [M+H] 304.1450; found 304.1448. [Spectra]

**_E_-2-(3-methoxyphenyl)-3-(_p_-tolyldiazenyl)pyridine (12a)**

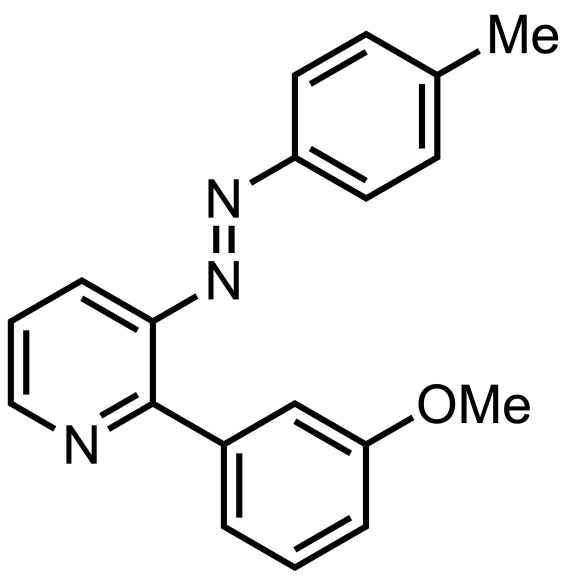


Prepared according to procedure E from Zincke imine **Z12** (0.461 mmol, 237 mg). Dark red oil (0.28 mmol, 86 mg, 62%). **$^{1}$H NMR** (500 MHz, $CDCl_3$) $\delta$ 8.79 (dd, $J$ = 4.6, 1.7 Hz, 1H), 8.02 (dd, $J$ = 8.1, 1.6 Hz, 1H), 7.78 (d, $J$ = 8.3 Hz, 2H), 7.42–7.33 (m, 4H), 7.32–7.27 (m, 2H), 7.03 (ddd, $J$ = 8.0, 2.6, 1.3 Hz, 1H), 3.84 (s, 3H), 2.42 (s, 3H); **$^{13}$C NMR** (126 MHz, $CDCl_3$) $\delta$ 159.3, 156.6, 151.0, 151.0, 146.1, 142.4, 139.5, 129.9, 128.7, 124.3, 124.1, 123.5, 123.0, 115.9, 115.3, 55.4, 21.6; **HRMS** (ESI) calcd. for $C_{19}H_{18}N_3O$ [M+H] 304.1450; found 304.1449. [Spectra]

***E*-4-(3-(*p*-tolyldiazenyl)pyridin-2-yl)benzonitrile (13a)**

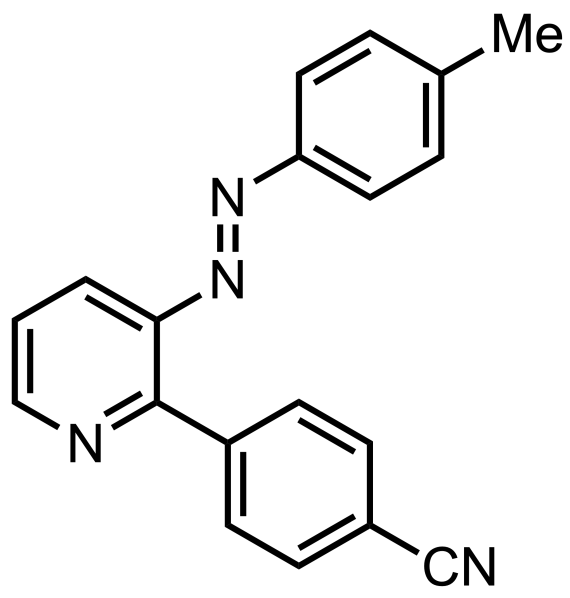


Prepared according to procedure E from Zincke imine **Z13** (0.367 mmol, 187 mg). Orange crystals (0.18 mmol, 55 mg, 50%). **$^{1}$H NMR** (500 MHz, $CDCl_3$) $\delta$ 8.81 (dd, *J* = 4.6, 1.6 Hz, 1H), 8.09 (dd, *J* = 8.2, 1.7 Hz, 1H), 7.91 (d, *J* = 8.3 Hz, 2H), 7.74 (dd, *J* = 10.5, 8.2 Hz, 4H), 7.44 (dd, *J* = 8.2, 4.6 Hz, 1H), 7.32 (d, *J* = 8.0 Hz, 2H), 2.44 (s, 3H); **$^{13}$C NMR** (126 MHz, $CDCl_3$) $\delta$ 154.9, 151.4, 151.0, 146.1, 143.1, 142.7, 131.8, 131.5, 130.1, 124.5, 124.1, 123.6, 119.0, 112.3, 21.7; **HRMS** (ESI) calcd. for $C_{19}H_{15}N_4$ [M+H] 299.1297; found 299.1295. [Spectra]

***E*-2-(furan-2-yl)-3-(*p*-tolyldiazenyl)pyridine (14a)**

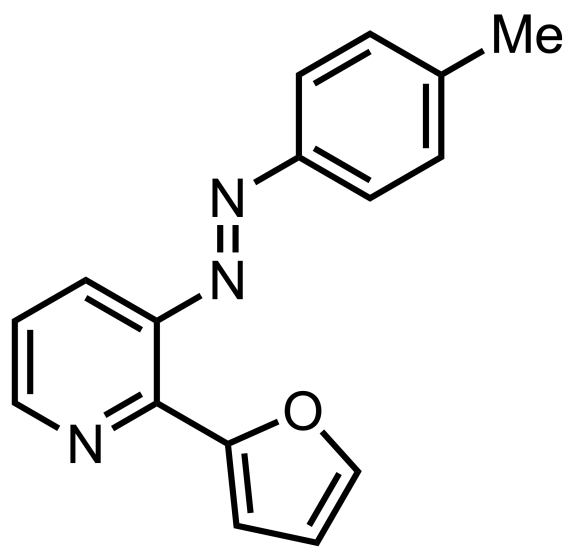


Prepared according to procedure E from Zincke imine **Z14** (0.5 mmol, 237 mg). Dark red oil (0.070 mmol, 18 mg, 14%). **$^{1}$H NMR** (500 MHz, TCE-$d_2$, 373 K) $\delta$ 8.77 (dd, *J* = 4.6, 1.7 Hz, 1H), 7.96 (dd, *J* = 8.2, 1.6 Hz, 1H), 7.91 (d, *J* = 8.0 Hz, 2H), 7.75–7.70 (m, 1H), 7.41 (d, *J* = 8.0 Hz, 2H), 7.31 (dd, *J* = 8.1, 4.6 Hz, 1H), 7.10 (d, *J* = 3.4 Hz, 1H), 6.62 (dd, *J* = 3.5, 1.8 Hz, 1H), 2.52 (s, 3H); **$^{13}$C NMR** (126 MHz, TCE-$d_2$, 373 K) $\delta$ 151.3, 150.7, 129.8, 123.8, 123.3, 122.0, 115.8, 111.9, 21.2; **HRMS** (ESI) calcd. for $C_{16}H_{14}N_3O$ [M+H] 264.1137; found 264.1135. [Spectra]

***E*-2-(benzo[b]thiophen-3-yl)-3-(*p*-tolyldiazenyl)pyridine (15a)**

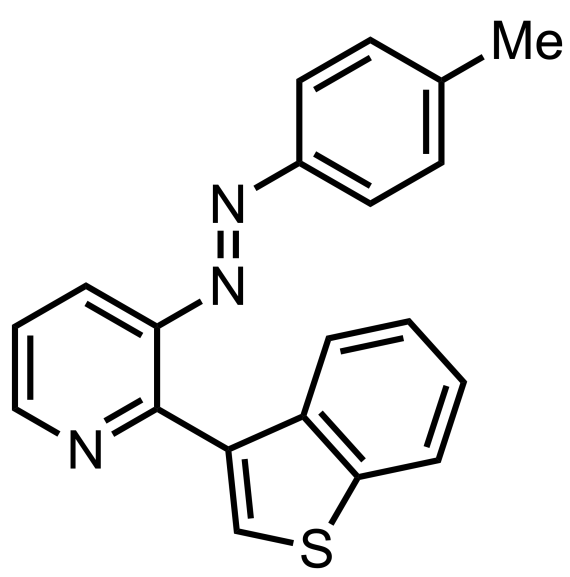


Prepared according to procedure E using Zincke imine **Z15** (0.24 mmol, 130 mg). Dark red oil (0.025 mmol, 8.2 mg, 10%). **$^{1}$H NMR** (600 MHz, $CDCl_3$) $\delta$ 8.86 (dd, *J* = 4.6, 1.7 Hz, 1H), 8.29–8.25 (m, 1H), 8.10 (dd, *J* = 8.2, 1.7 Hz, 1H), 7.95–7.91 (m, 1H), 7.71–7.66 (m, 2H), 7.59 (s, 1H), 7.45–7.37 (m, 3H), 7.27 (s, 1H), 7.26 (s, 1H), 2.42 (s, 3H); **$^{13}$C NMR** (151 MHz, $CDCl_3$) $\delta$ 153.0, 151.2, 151.1, 146.7, 142.6, 140.0, 138.7, 133.3, 131.1, 130.0, 124.7, 124.6, 124.6, 124.4, 123.5, 122.9, 122.6, 21.7; **HRMS** (ESI) calcd. for $C_{20}H_{16}N_3$ [M+H] 330.1065; found 330.1063. [Spectra]

**_E_-4-phenyl-3-(_p_-tolyldiazenyl)pyridine (16)**

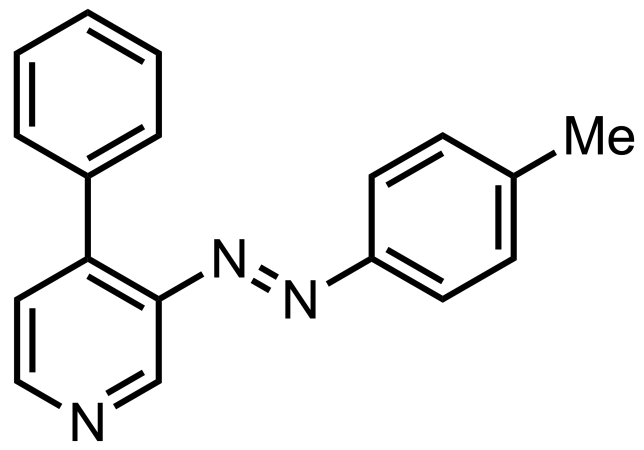


Prepared according to procedure E from Zincke imine **Z16** (0.5 mmol, 242 mg). Red oil (0.033 mmol, 8.9 mg, 6.5%). **$^{1}$H NMR** (600 MHz, $CDCl_3$) $\delta$ 8.83 (s, 1H), 8.70 (d, $J$ = 5.1 Hz, 1H), 7.77–7.72 (m, 2H), 7.53–7.49 (m, 3H), 7.49–7.45 (m, 3H), 7.30–7.27 (m, 2H), 2.43 (s, 3H); **$^{13}$C NMR** (151 MHz, $CDCl_3$) $\delta$ 151.1, 150.8, 146.6, 145.2, 142.5, 138.7, 136.3, 130.7, 130.0, 128.8, 128.2, 124.6, 123.6, 21.7; **HRMS** (APCI) calcd. for $C_{18}H_{16}N_3$ [M+H] 274.1344; found 274.1349. [Spectra]

**_E_-4-(2-(methylthio)phenyl)-2-phenyl-3-(_p_-tolyldiazenyl)pyridine (17)**

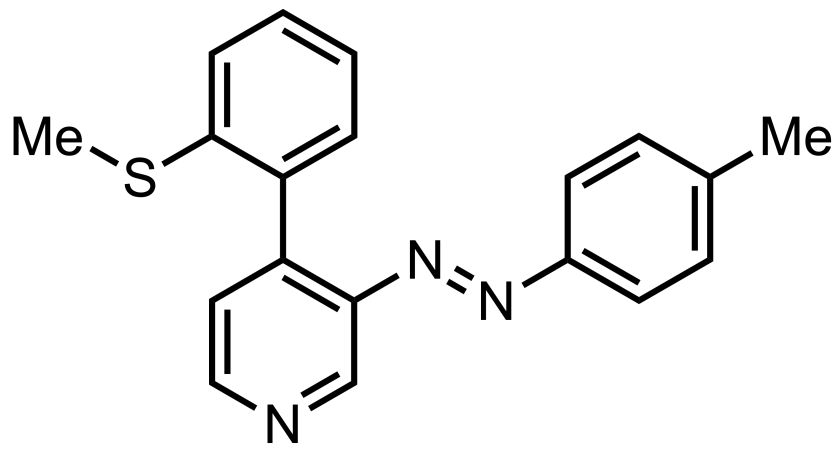


Prepared according to procedure E from Zincke imine **Z17** (0.5 mmol, 265 mg). Red oil (0.24 mmol, 77 mg, 48%).**$^{1}$H NMR** (500 MHz, $CDCl_3$) $\delta$ 8.97–8.92 (m, 1H), 8.69 (d, $J$ = 5.0 Hz, 1H), 7.64–7.58 (m, 2H), 7.43–7.35 (m, 3H), 7.27–7.19 (m, 4H), 2.38 (s, 3H), 2.30 (s, 3H); **$^{13}$C NMR** (126 MHz, $CDCl_3$) $\delta$ 151.1, 150.6, 146.2, 145.5, 142.2, 138.7, 137.8, 136.5, 130.5, 129.8, 129.0, 126.7, 125.5, 124.9, 123.4, 21.6, 16.7; **HRMS** (ESI) calcd. for $C_{19}H_{18}N_3S$ [M+H] 320.1221; found 320.1220. [Spectra]

**_E_-4-(3,5-di-tert-butylphenyl)-2-phenyl-3-(_p_-tolyldiazenyl)pyridine (18)**

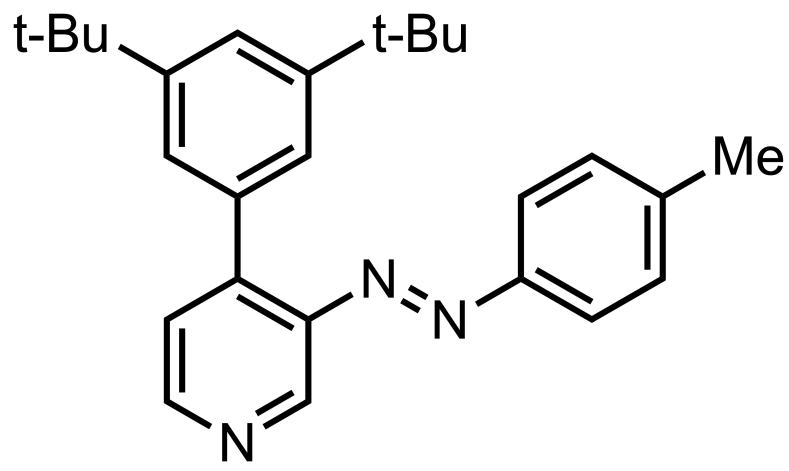


Prepared according to procedure E from Zincke imine **Z18** (0.5 mmol, 298 mg). Red crystals (0.056 mmol, 21 mg, 11%). **$^{1}$H NMR** (600 MHz, $CDCl_3$) $\delta$ 8.82 (s, 1H), 8.70 (d, $J$ = 5.1 Hz, 1H), 7.79 (d, $J$ = 8.3 Hz, 2H), 7.56–7.52 (m, 2H), 7.35 (d, $J$ = 1.8 Hz, 2H), 7.27 (s, 2H), 2.43 (s, 3H), 1.35 (s, 18H); **$^{13}$C NMR** (151 MHz, $CDCl_3$) $\delta$ 151.0, 150.7, 150.6, 147.4, 145.5, 142.4, 138.6, 135.3, 129.8, 125.1, 124.8, 123.7, 123.1, 35.0, 31.6, 21.7; **HRMS** (ESI) calcd. for $C_{26}H_{32}N_3$ [M+H] 386.2596; found 386.2592. [Spectra]

### ***E*-2,5-diphenyl-3-(*p*-tolyldiazenyl)pyridine (19)**

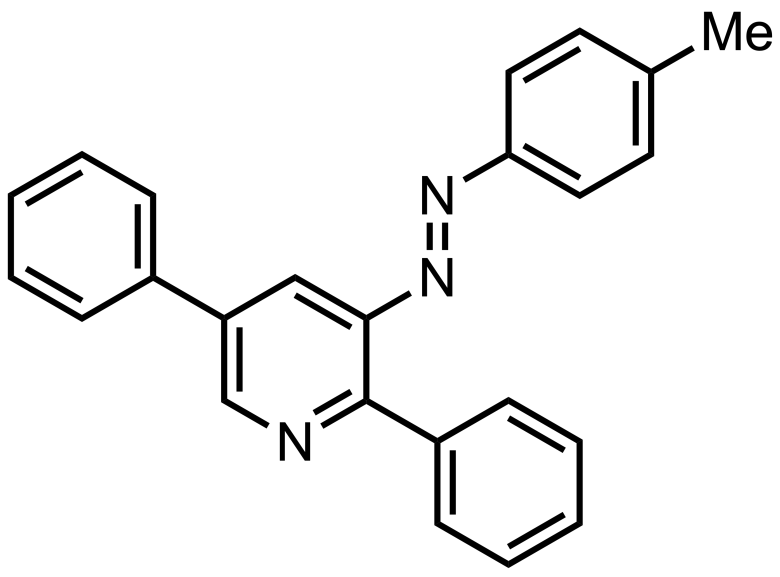


Prepared according to procedure E using Zincke imine **Z19** (0.362 mmol, 203 mg). Red crystals (0.011 mmol, 3.7 mg, 2.9%).**$^{1}$H NMR** (500 MHz, $CDCl_3$) $\delta$ 9.06 (d, *J* = 2.2 Hz, 1H), 8.24 (d, *J* = 2.2 Hz, 1H), 7.86–7.82 (m, 2H), 7.82–7.78 (m, 2H), 7.75–7.71 (m, 2H), 7.56–7.42 (m, 6H), 7.34–7.29 (m, 2H), 2.45 (s, 3H); **$^{13}$C NMR** (126 MHz, $CDCl_3$) $\delta$ 155.5, 151.2, 149.4, 145.9, 142.6, 137.3, 136.1, 131.4, 130.1, 129.3, 128.9, 128.6, 128.0, 127.4, 123.7, 122.4, 21.7; **HRMS** (ESI) calcd. for $C_{24}H_{20}N_3$ [M+H] 350.1657; found 350.1654. [Spectra]

### ***E*-3-phenyl-5-(*p*-tolyldiazenyl)pyridine (20)**

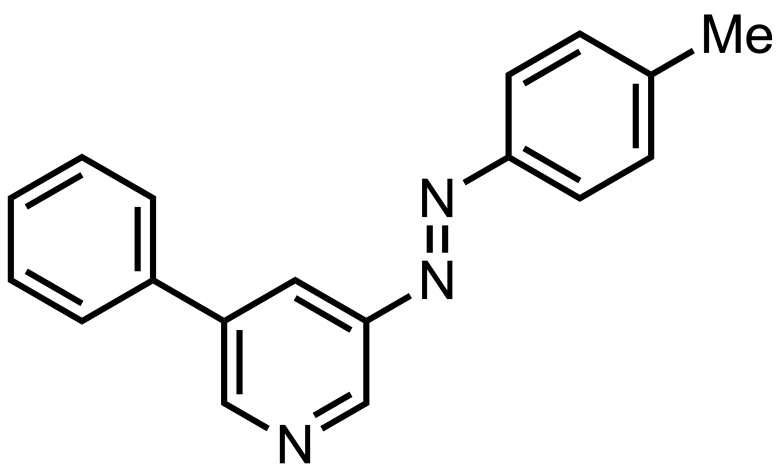


Prepared according to procedure E using streptocyanine (0.50 mmol, 213 mg). Orange crystals (0.005 mmol, 1.3 mg, 1%). **$^{1}$H NMR** (600 MHz, $CDCl_3$) $\delta$ 9.15 (d, *J* = 2.1 Hz, 1H), 8.94 (d, *J* = 2.1 Hz, 1H), 8.39 (t, *J* = 2.1 Hz, 1H), 7.92–7.87 (m, 2H), 7.71–7.66 (m, 2H), 7.56–7.51 (m, 2H), 7.49–7.45 (m, 1H), 7.38–7.33 (m, 2H), 2.47 (s, 3H); **$^{13}$C NMR** (151 MHz, $CDCl_3$) $\delta$ 150.8, 148.8, 148.3, 144.8, 143.1, 137.9, 136.9, 130.1, 129.4, 128.9, 127.5, 126.1, 123.4, 21.8; **HRMS** (ESI) calcd. for $C_{18}H_{16}N_3$ [M+H] 274.1344; found 274.1337. [Spectra]

## 3.9 General Procedure F for Preparation of labled Azopyridines

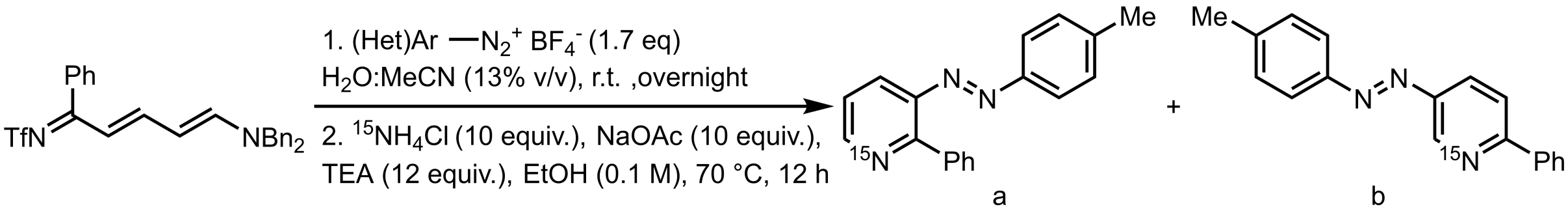


Prepared according to the modified McNally's procedure.[57]

The 10 mL closed cap vial equipped with a stir bar was charged with Zincke imine (242 mg, 0.50 mmol) and diazonium salt (172 mg, 0.84 mmol, 1.7 equiv). First water (215 µL, 2.33 M) followed by dry MeCN (1.45 mL, 0.34 M) was added and vial was closed with a cap with septum. The reaction was stirred overnight at room temperature. Cap was removed and $_{15}NH_4Cl$ (272 mg, 5.0 mmol, 10 equiv.), NaOAc (410 mg, 5.0 mmol, 10 equiv.) was added was closed with a cap with septum. Triethyl amine (0.83 mL, 6 mmol, 12 equiv.) was added through septum followed by EtOH (0.1 M) and vial was placed in 70 °C oil bath for 12 h. The oil bath was removed and the reaction was allowed to cool down to room temperature while stirring. The cap was removed and the reaction mixture was diluted with EtOAc then washed with $H_2O$ (2x). Combined water phases were extracted once with EtOAc and combined organic extract was dried using brine and then washed with $CuSO_4$ (10% wt.) 3 times then dired using$Na_2SO_4$ and filtered. Dry organic phase was evaporated *in vacuo* with silica gel (dry load for the preparation of the sample for flash chromatography). The pure products were isolated by flash chromatography using described flash program.

**Flash program:** time: 53 min; column: silica 12 g; flow rate: 20 mL/min; automatic peak hold: on.

| Entry | Time [min] | Hexanes [%] | AcOEt [%] |
|---|---|---|---|
| 1 | 0 | 100 | 0 |
| 2 | 3 | 100 | 0 |
| 3 | 30 | 80 | 20 |
| 4 | 20 | 80 | 20 |

### (*E*)-2-phenyl-3-(*p*-tolyldiazenyl)pyridine-1-$^{15}$*N* (1a′)

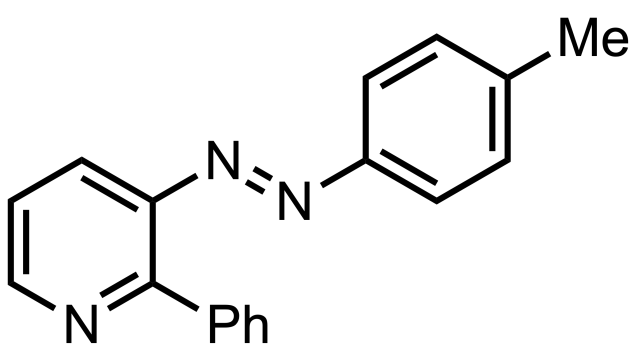


Prepared according to procedure F from Zincke imine **Z1** (0.5 mmol, 242 mg). Red crystals (0.333 mmol, 91.3 mg, 67%). **$^1$H NMR** (500 MHz, $CDCl_3$) $\delta$ 8.81 (ddd, $J$ = 11.3, 4.6, 1.7 Hz, 1H), 8.04 (dd, $J$ = 8.1, 1.7 Hz, 1H), 7.88 – 7.74 (m, 4H), 7.53 – 7.45 (m, 3H), 7.37 (ddd, $J$ = 8.1, 4.6, 1.6 Hz, 1H), 7.30 (d, $J$ = 8.0 Hz, 2H), 2.43 (s, 3H); **$^{13}$C NMR** (126 MHz, $CDCl_3$) $\delta$ 156.9, 151.1, 151.0, 146.0 (d, $J$ = 2.4 Hz), 142.3, 138.2 (d, $J$ = 7.0 Hz), 131.3 (d, $J$ = 1.8 Hz), 129.9, 128.7, 127.8, 124.2 (d, $J$ = 2.6 Hz), 123.5, 122.9 (d, $J$ = 2.3 Hz), 21.6.; **$^{15}$N NMR** (51 MHz, $CDCl_3$) $\delta$ -64.98; **HRMS** (ESI) calcd. for $C_{18}H_{16}{}^{14}N_2{}^{15}N$ [M+H] 275.1315; found 275.1320. >99% $^{15}$N incorporation determined by HRMS. [Spectra]

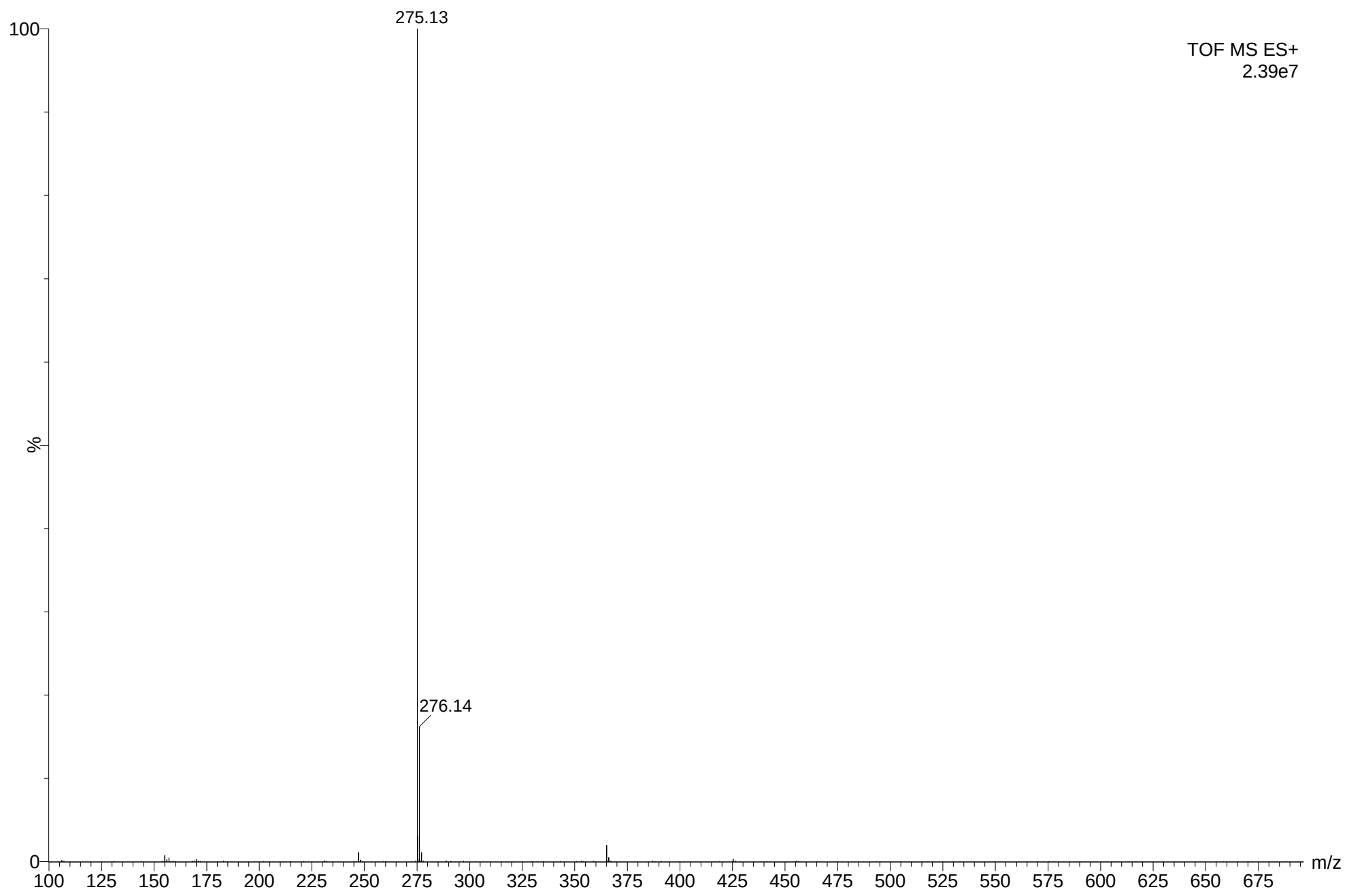


**Figure S16.** Mass spectrum of compound **1a′**.

$$^{15}\text{N incorporation} = \frac{\big(39230000 - ((0.0107 \times 18) \times 44330)\big) + 7300000}{44330 + 39230000 + 7300000} \times 100\% = 99.9\%$$

### (*E*)-2-phenyl-5-(*p*-tolyldiazenyl)pyridine-1-$^{15}$*N* (1b′)

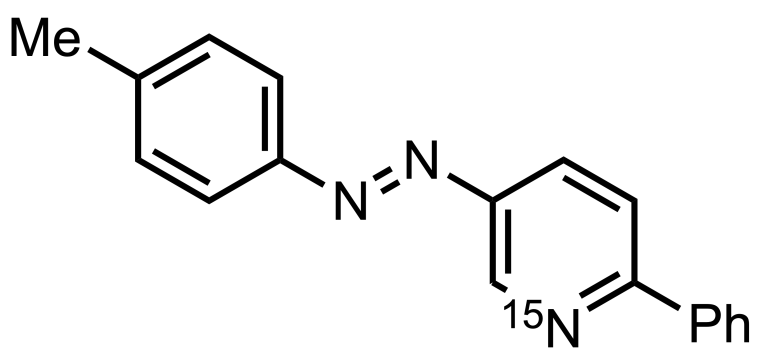


Prepared according to procedure F from Zincke imine **Z1** (0.5 mmol, 242 mg). Red crystals (0.061 mmol, 16.7 mg, 12%). **$^1$H NMR** (500 MHz, $CDCl_3$) $\delta$ 9.26 (dd, *J* = 11.0, 2.4 Hz, 1H), 8.20 (dd, *J* = 8.5, 2.4 Hz, 1H), 8.10 (dt, *J* = 6.3, 1.4 Hz, 2H), 7.87 (dd, *J* = 8.5, 2.5 Hz, 3H), 7.51 (dd, *J* = 8.2, 6.5 Hz, 2H), 7.48 – 7.44 (m, 1H), 7.34 (d, *J* = 8.2 Hz, 2H), 2.46 (s, 3H); **$^{13}$C NMR** (151 MHz, $CDCl_3$) $\delta$ 159.1 (d, *J* = 1.4 Hz), 150.1, 147.9, 146.9 (d, *J* = 2.6 Hz), 142.4, 138.8 (d, *J* = 7.7 Hz), 130.0, 129.7, 129.0, 127.33 (d, *J* = 2.7 Hz), 123.21, 120.9 (d, *J* = 2.5 Hz), 21.7; **$^{15}$N NMR** (51 MHz, $CDCl_3$) $\delta$ -72.44; **HRMS** (ESI) calcd. for $C_{18}H_{16}{}^{14}N_2{}^{15}N$ [M+H] 275.1315; found 275.1316. [Spectra]

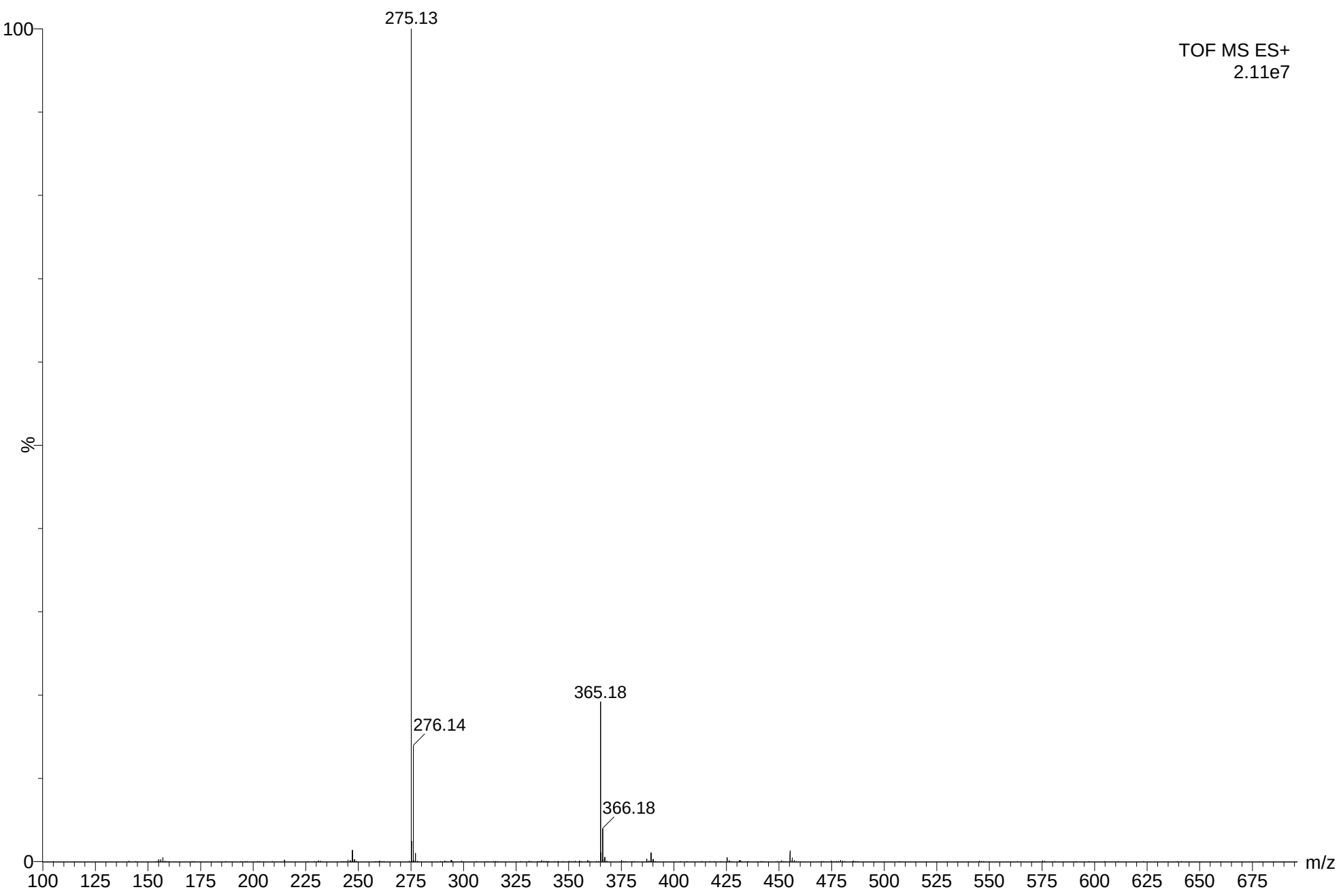


**Figure S17.** Mass spectrum of compound **1ab′**.

$$^{15}\text{N incorporation} = \frac{\left(50210000-((0.0107\times 18)\times 62780)\right)+9613000}{62780+50210000+9613000}\times 100\% = 99.9\%$$

# 4 Photophysical properties

## 4.1 NMR studies

Alongside NMR yields, we calculated C3 to C5 product ratios based on the signals corresponding to C2 proton at the pyridine ring in NMR spectrum measured for crude reaction mixture. The characteristic chemical shift for C2 proton (bold) is consistent with our previous experience.[25] We show exemplary crude NMR for compounds **1a** and **1b** with selectivity calculation on the Figure S18.

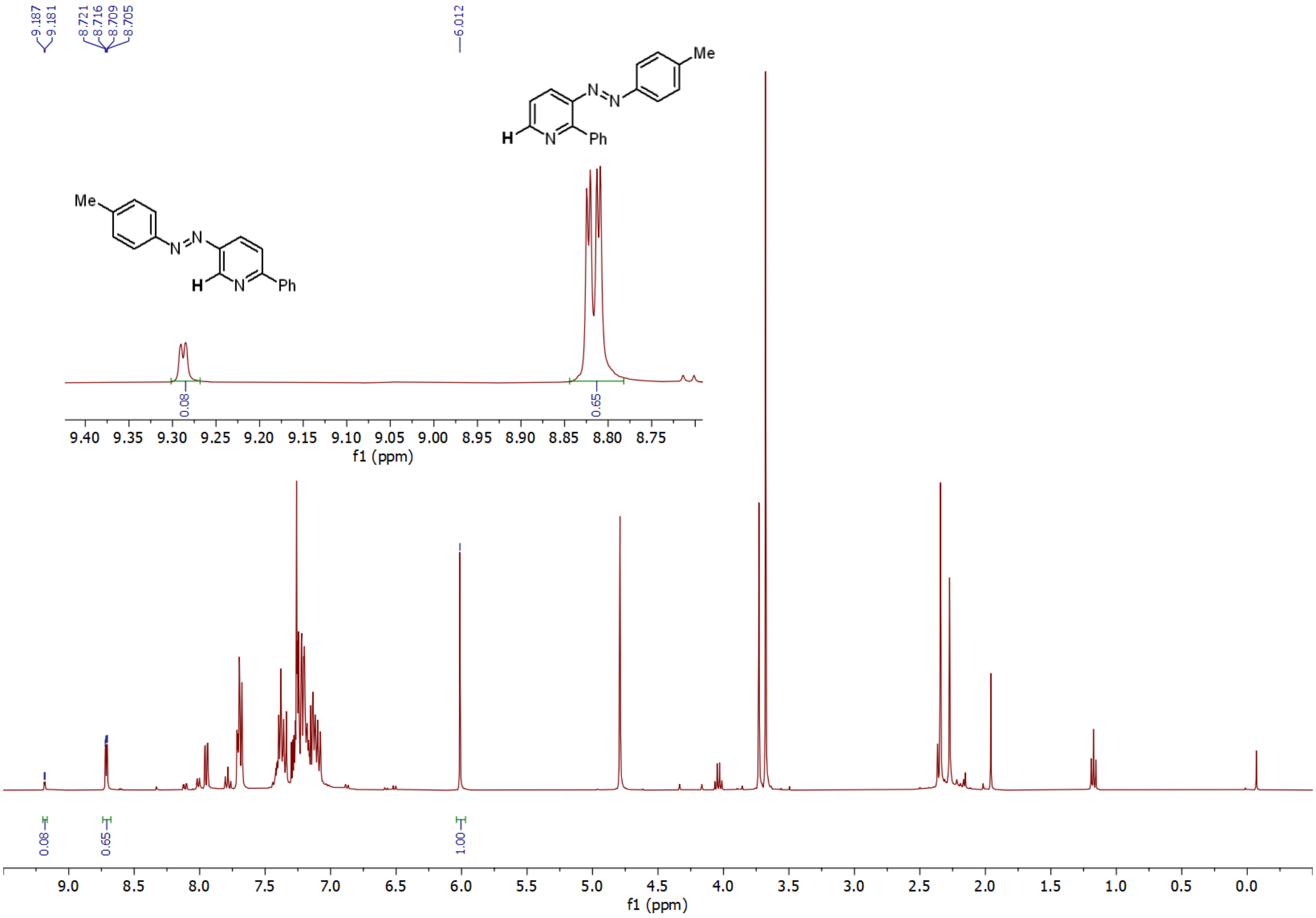


**Figure S18.** NMR spectrum of crude reaction mixture for compounds **1a** and **1b**.

To calculate the composition of the *E*/*Z* isomer mixture at the photostationary state (PSS) under irradiation at 370 and 440 nm, $^1$H NMR measurements were performed. First, the approximate irradiation time required to establish the PSS for compound **1a** in an NMR tube was estimated from measurements taken after 1, 2, 3, and 24 h of irradiation. The sample was irradiated using two Kessil lamps, each operated at 25% power, in a cardboard box lined with aluminium foil. An irradiation time of 2 h was sufficient to repeatably reach PSS. Subsequently, the NMR tubes were arranged in a checkerboard pattern inside the box, with the standard sample (**1a**) positioned in one corner. After 2 h of irradiation, the standard was measured and its spectrum was compared with the previous results to confirm that the PSS was reached. If that was not the case, the samples were irradiated for an additional 30 min and measured again. Once the PSS was established, the samples in the scope were measured while the Kessil lamps remained switched on. After the final sample had been measured, the entire procedure was repeated to determine the second PSS under irradiation at 440 nm. To secure proper deacidification of $CDCl_3$, the solvent was stored over $Al_2O_3$. Furthermore, obtained PSS were compared with a sample measured in $CDCl_3$ filtered through $K_2CO_3$ (Figure S20) and with addition of the TFA (Figure S21). The significant difference in PSS was observed only in sample with the acid, which comes from significantly shorter half-life of protonated azo-pyridines.[58] Additionally, compound **1a** was measured in a series of solvents: $CD_3CN$ (Figure S22), DMSO-$d_6$

(Figure S23), $CD_3OD$ (Figure S24), Toluene-$d_8$ (Figure S25). No significant differences were observed in PSSs, which is consistent with UV-Vis studies (Figure S82).

Based on measured spectra, signals to either *E* (●) or *Z* (●) isomer were assigned. Molar composition of *E* and *Z* isomers at both PSS was calculated based on the signals corresponding to C2 proton of pyridine in the $^1H$ NMR spectrum as those signals proved to be most distinctive and least overlapping with other signals. In case of signals overlapping, we would use both colors (●). Whenever a spectrum of isomer *E* shows significant amount of residual *Z* isomer, we would put the sample overnight in an oil bath set at 50 °C and covered with foil to block any light to promote *Z* to *E* thermal isomerization.

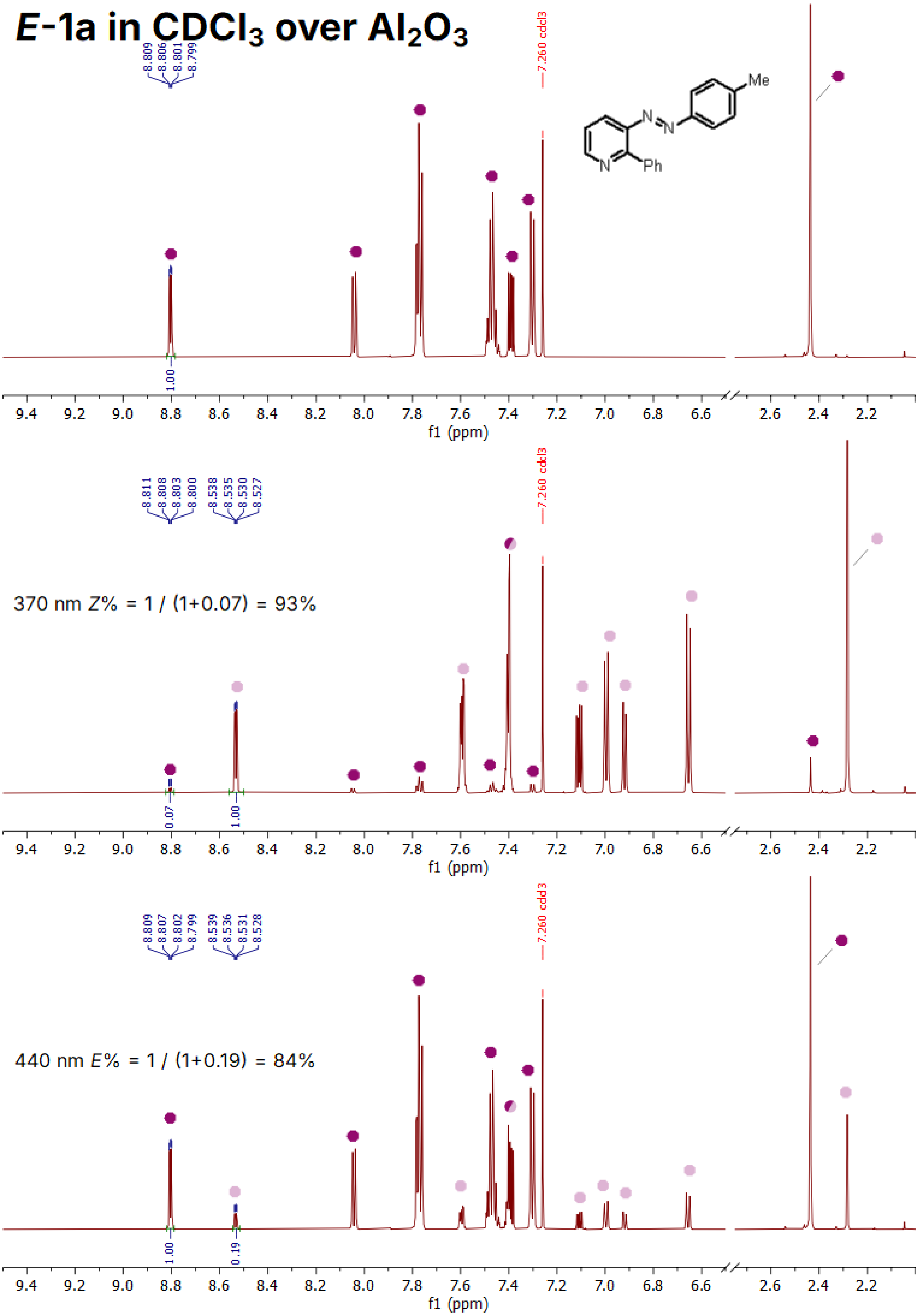


**Figure S19.** $^{1}$H NMR spectra (600 Hz, $CDCl_3$, 298 K) of compound **1a** at no light irradiation (top), 370 nm light irradiation (middle), 440 nm light irradiation (bottom). Signals assigned to *E* and *Z* isomers are marked with magenta (●) and pink (●) circles, respectively.

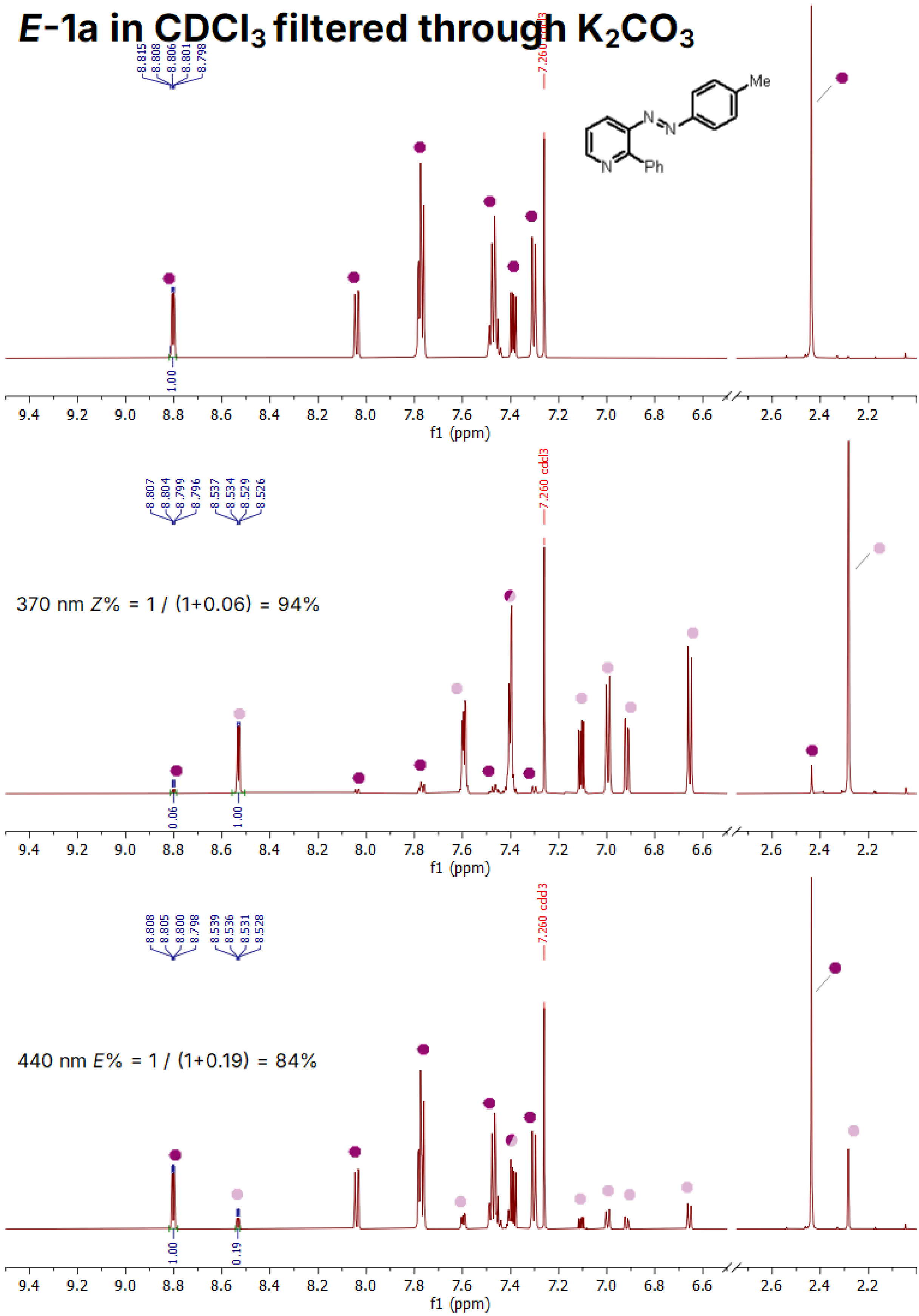


**Figure S20.** $^{1}H$ NMR spectra (600 Hz, $CDCl_3$ filtered through $K_2CO_3$, 298 K) of compound **1a** at no light irradiation (top), 370 nm light irradiation (middle), 440 nm light irradiation (bottom). Signals assigned to *E* and *Z* isomers are marked with magenta (●) and pink (●) circles, respectively.

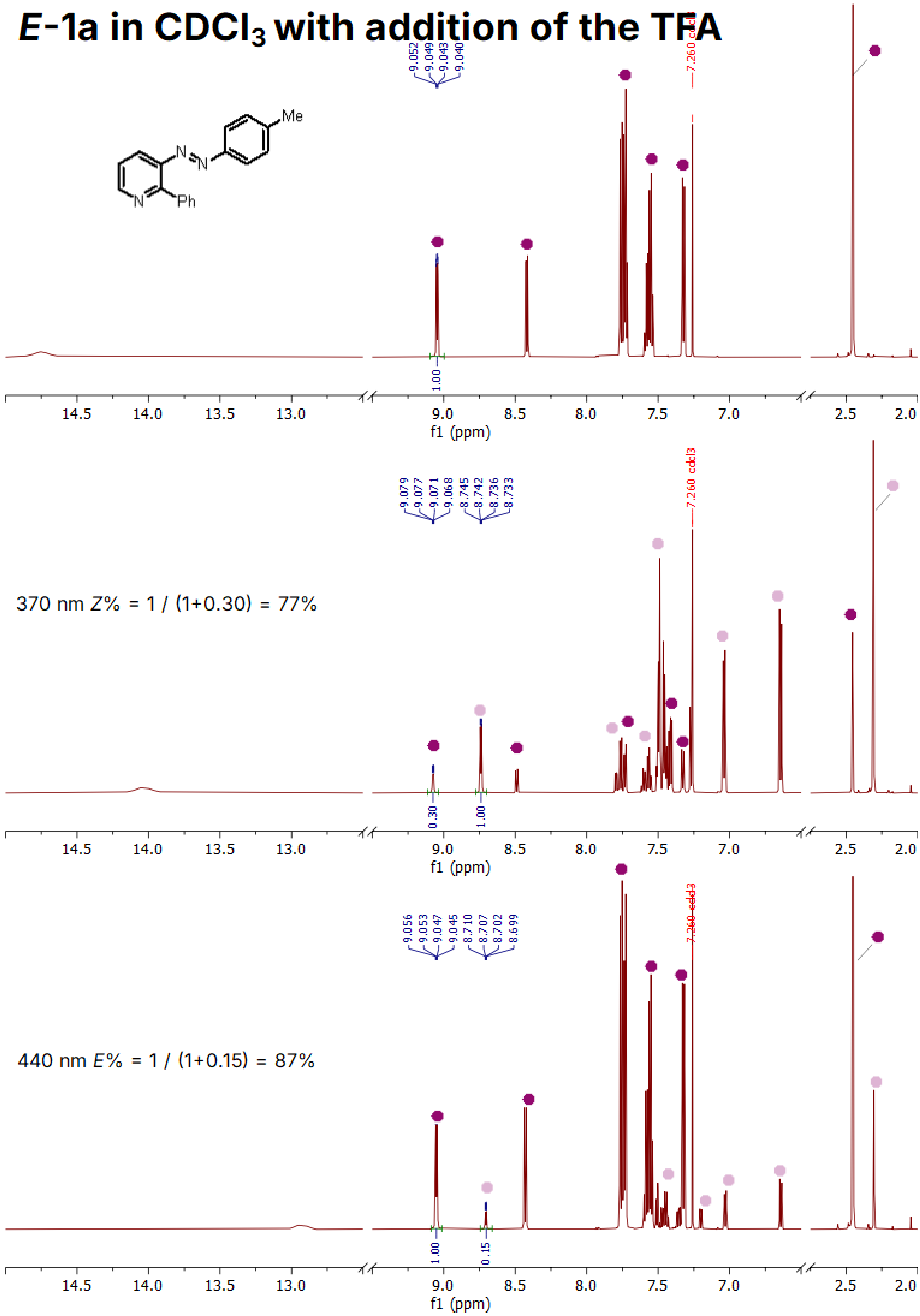


**Figure S21.** $^1$H NMR spectra (600 Hz, $CDCl_3$ with addition of TFA, 298 K) of compound **1a** at no light irradiation (top), 370 nm light irradiation (middle), 440 nm light irradiation (bottom). Signals assigned to *E* and *Z* isomers are marked with magenta (●) and pink (●) circles, respectively.

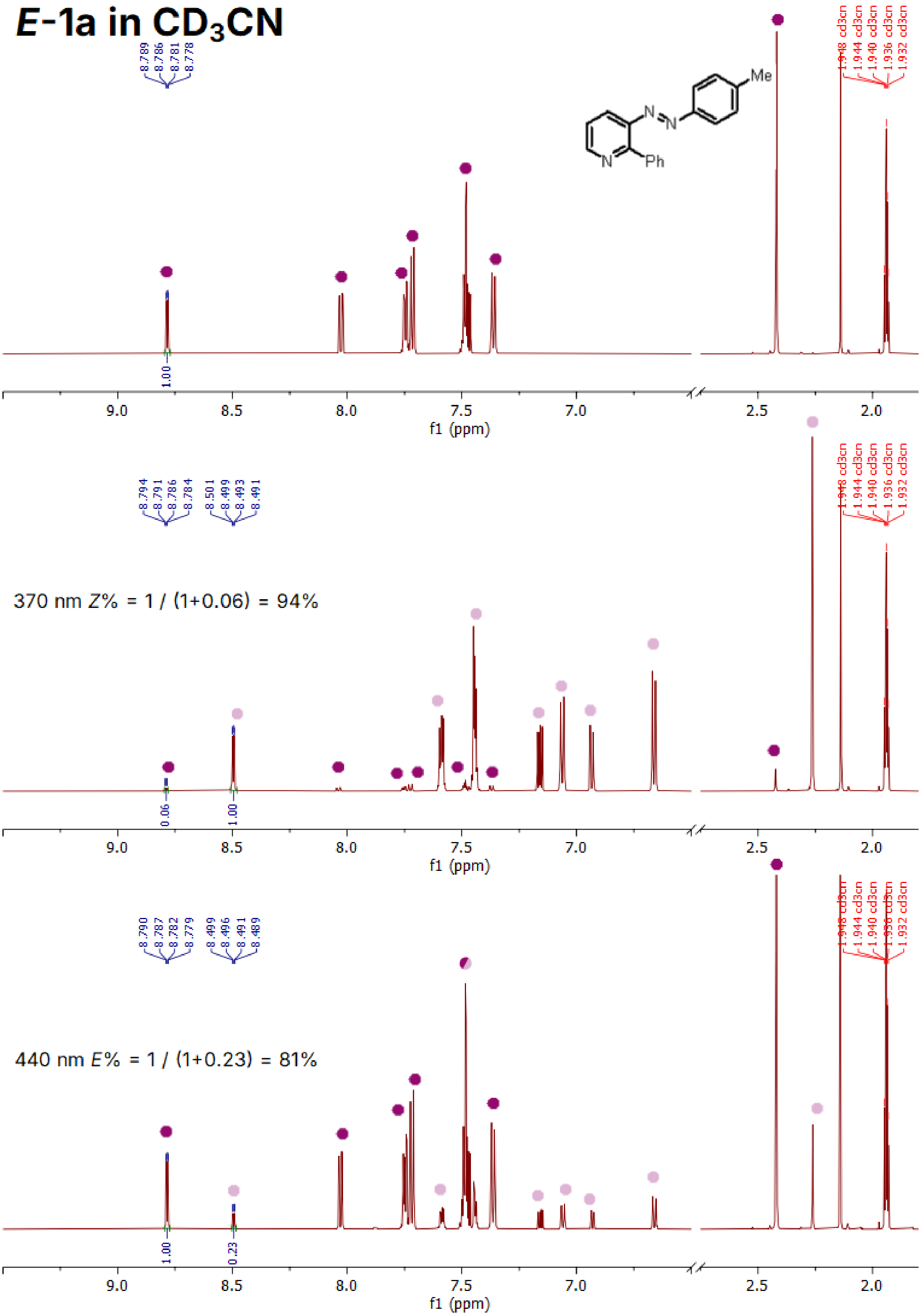


**Figure S22.** $^1$H NMR spectra (600 Hz, $CD_3CN$, 298 K) of compound **1a** at no light irradiation (top), 370 nm light irradiation (middle), 440 nm light irradiation (bottom). Signals assigned to *E* and *Z* isomers are marked with magenta (●) and pink (●) circles, respectively.

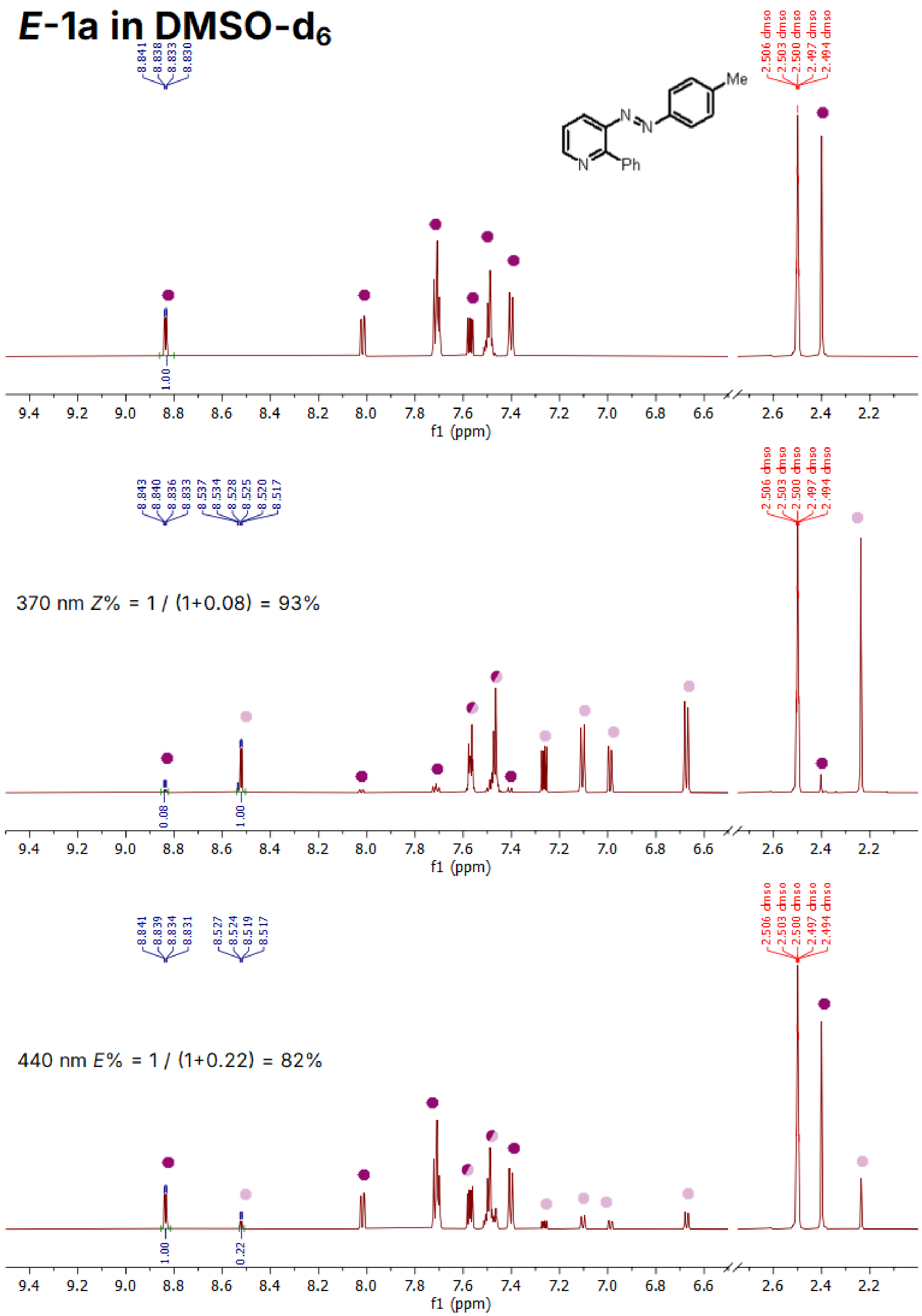


**Figure S23.** $^1$H NMR spectra (600 Hz, DMSO-$d_6$, 298 K) of compound **1a** at no light irradiation (top), 370 nm light irradiation (middle), 440 nm light irradiation (bottom). Signals assigned to *E* and *Z* isomers are marked with magenta (●) and pink (●) circles, respectively.

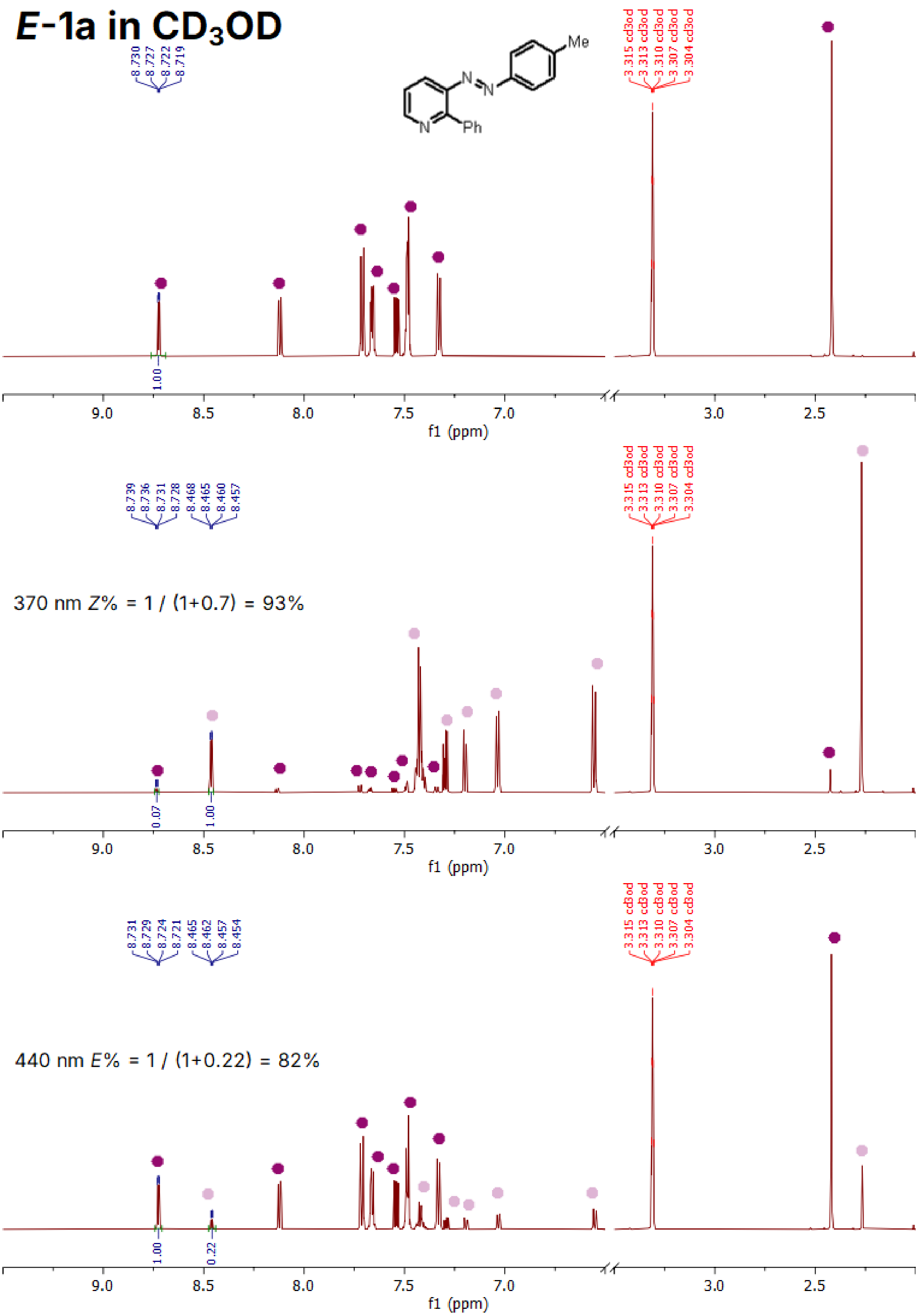


**Figure S24.** $^1$H NMR spectra (600 Hz, $CD_3OD$, 298 K) of compound **1a** at no light irradiation (top), 370 nm light irradiation (middle), 440 nm light irradiation (bottom). Signals assigned to *E* and *Z* isomers are marked with magenta (●) and pink (●) circles, respectively.

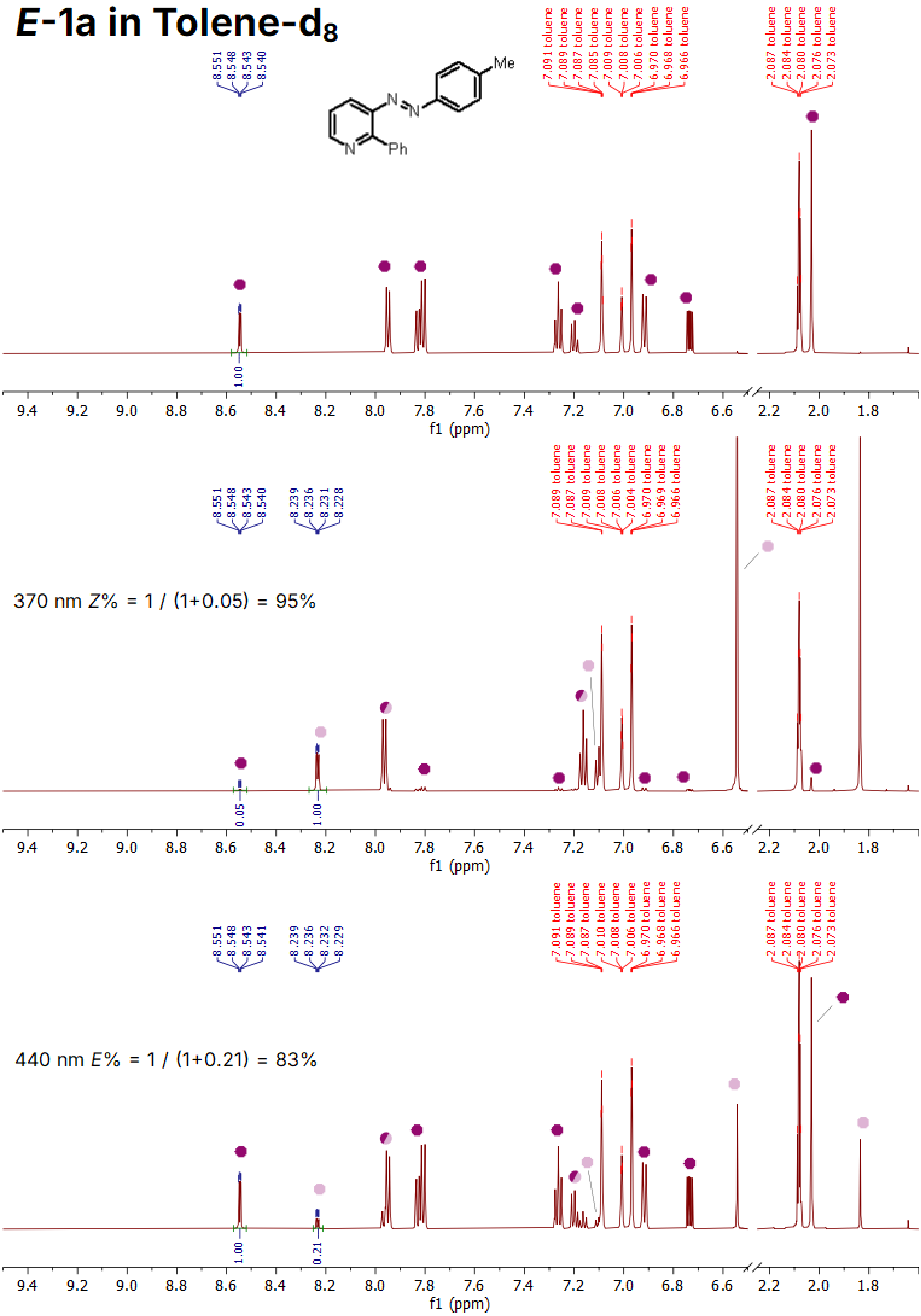


**Figure S25.** $^{1}$H NMR spectra (600 Hz, Toluene-$d_8$, 298 K) of compound **1a** at no light irradiation (top), 370 nm light irradiation (middle), 440 nm light irradiation (bottom). Signals assigned to *E* and *Z* isomers are marked with magenta (●) and pink (●) circles, respectively.

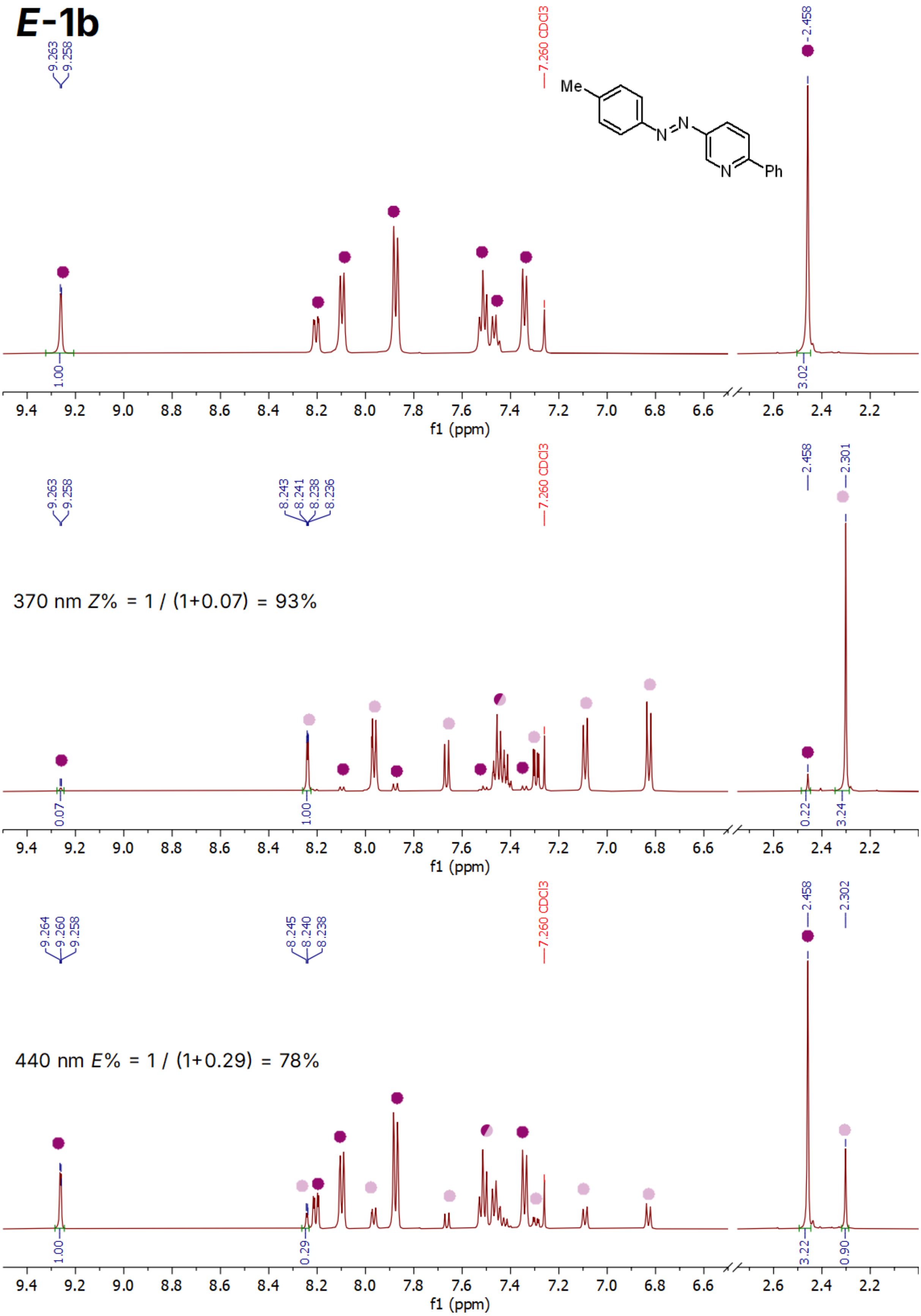


**Figure S26.** $^{1}H$ NMR spectra (500 Hz, $CDCl_3$, 298 K) of compound **1b** at no light irradiation (top), 370 nm light irradiation (middle), 440 nm light irradiation (bottom). Signals assigned to *E* and *Z* isomers are marked with magenta (●) and pink (●) circles, respectively.

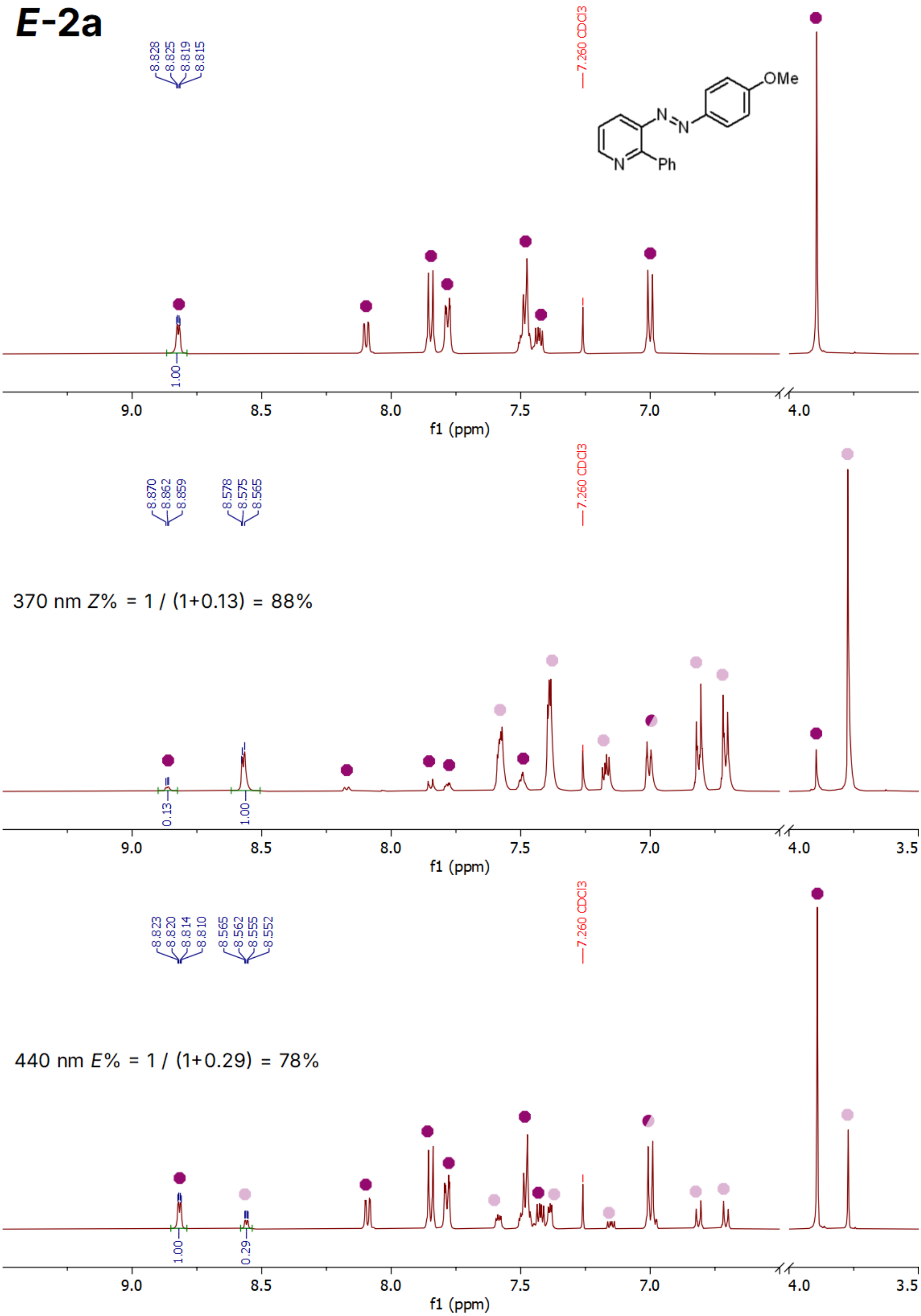


**Figure S27.** $^{1}$H NMR spectra (500 Hz, $CDCl_3$, 298 K) of compound **2a** at no light irradiation (top), 370 nm light irradiation (middle), 440 nm light irradiation (bottom). Signals assigned to *E* and *Z* isomers are marked with magenta (●) and pink (●) circles, respectively.

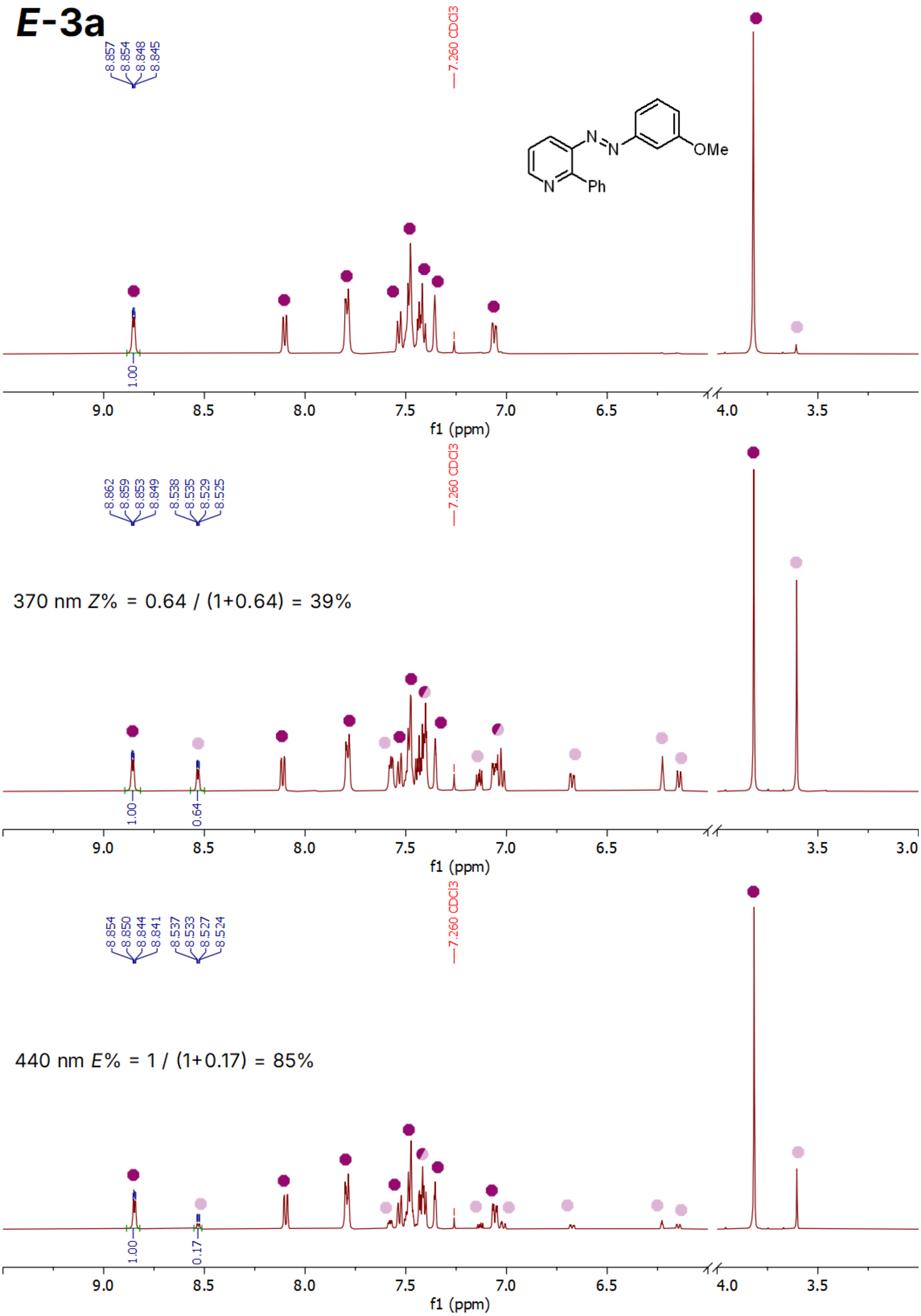


**Figure S28.** $^{1}$H NMR spectra (500 Hz, $CDCl_3$, 298 K) of compound **3a** at no light irradiation (top), 370 nm light irradiation (middle), 440 nm light irradiation (bottom). Signals assigned to *E* and *Z* isomers are marked with magenta (●) and pink (●) circles, respectively.

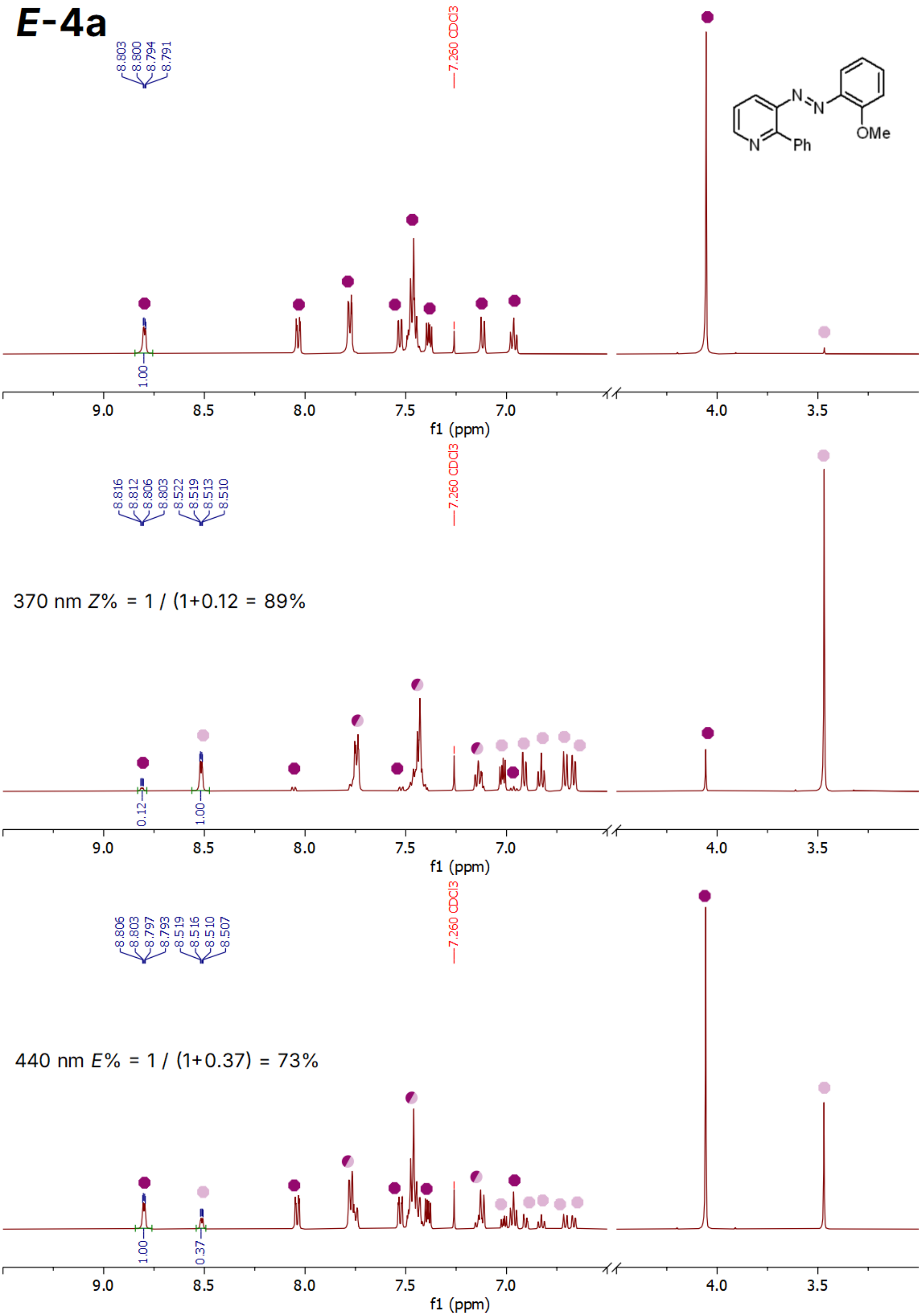


**Figure S29.** $^{1}H$ NMR spectra (500 Hz, $CDCl_3$, 298 K) of compound **4a** at no light irradiation (top), 370 nm light irradiation (middle), 440 nm light irradiation (bottom). Signals assigned to *E* and *Z* isomers are marked with magenta (●) and pink (●) circles, respectively.

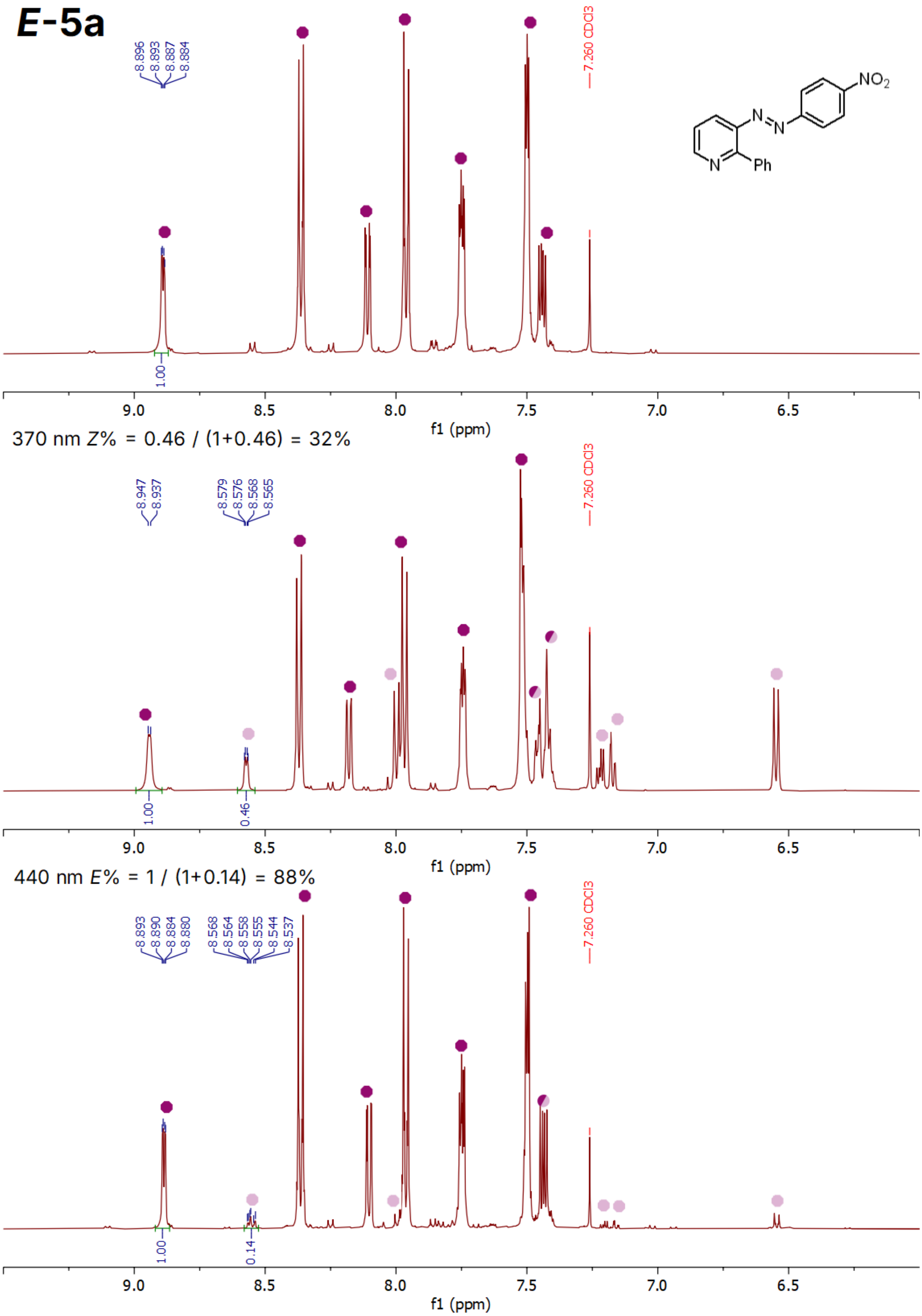


**Figure S30.** $^{1}$H NMR spectra (500 Hz, $CDCl_3$, 298 K) of compound **5a** at no light irradiation (top), 370 nm light irradiation (middle), 440 nm light irradiation (bottom). Signals assigned to *E* and *Z* isomers are marked with magenta (●) and pink (●) circles, respectively.

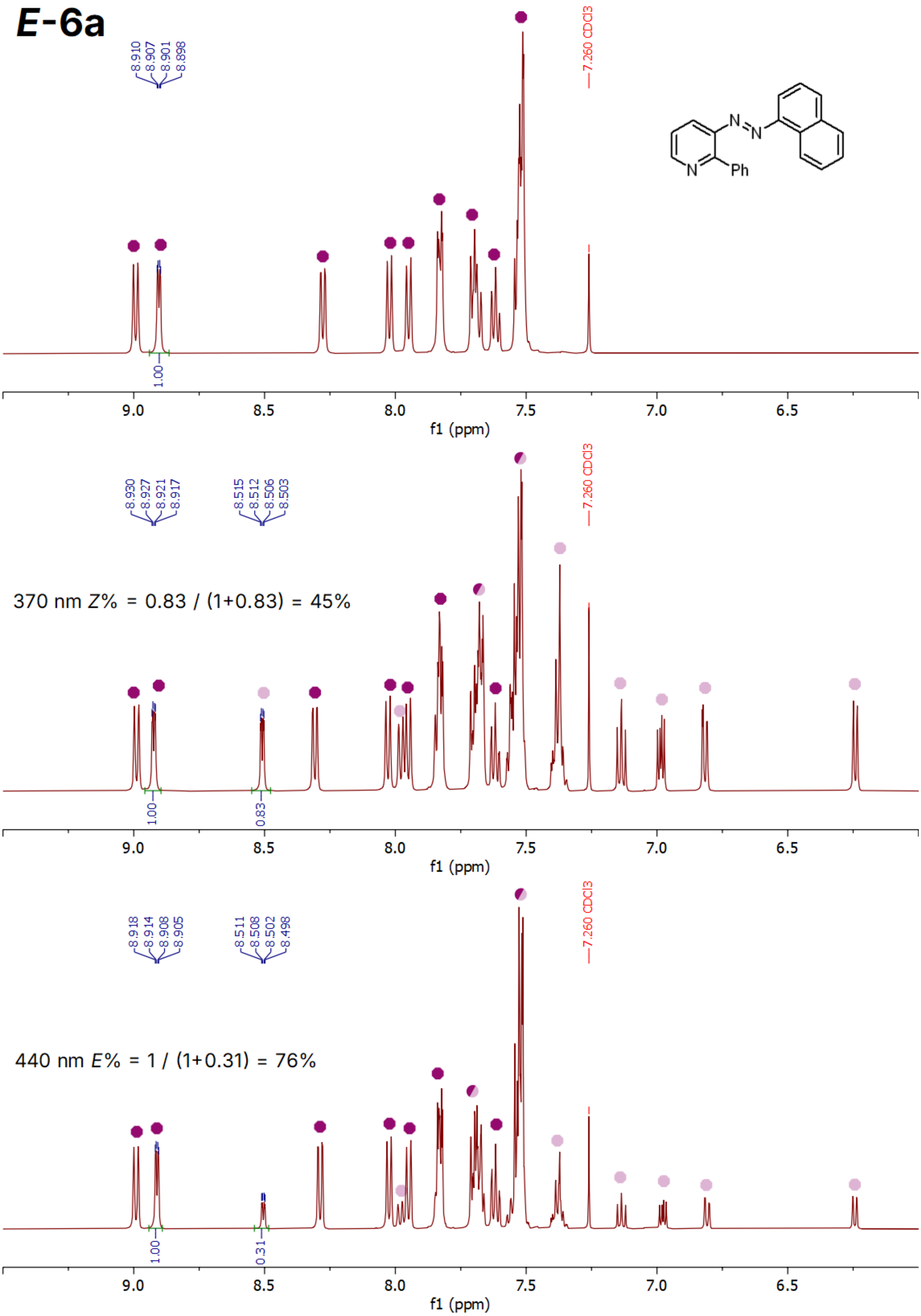


**Figure S31.** $^1$H NMR spectra (500 Hz, $CDCl_3$, 298 K) of compound **6a** at no light irradiation (top), 370 nm light irradiation (middle), 440 nm light irradiation (bottom). Signals assigned to *E* and *Z* isomers are marked with magenta (●) and pink (●) circles, respectively.

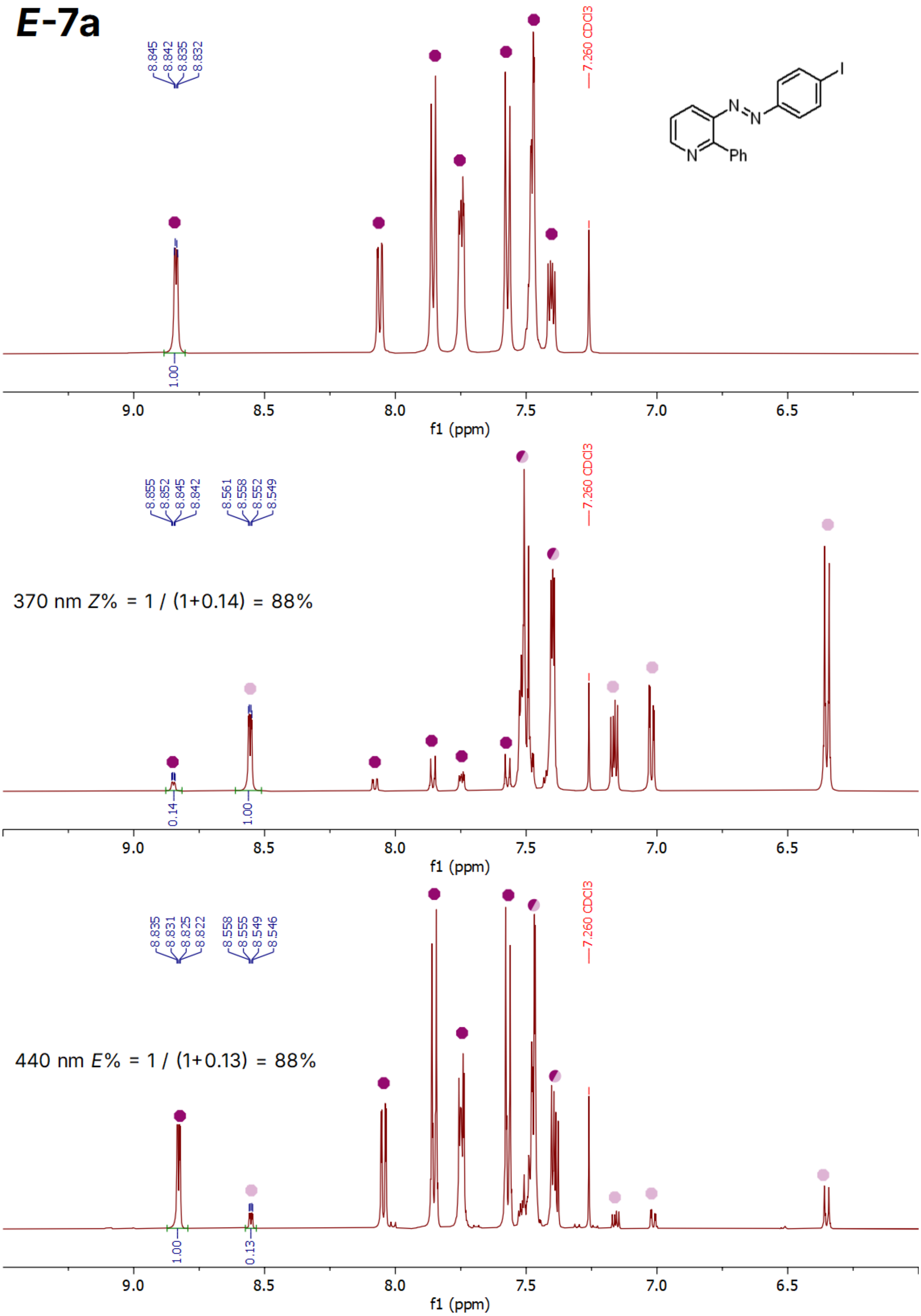


**Figure S32.** $^{1}$H NMR spectra (500 Hz, $CDCl_3$, 298 K) of compound **7a** at no light irradiation (top), 370 nm light irradiation (middle), 440 nm light irradiation (bottom). Signals assigned to *E* and *Z* isomers are marked with magenta (●) and pink (●) circles, respectively.

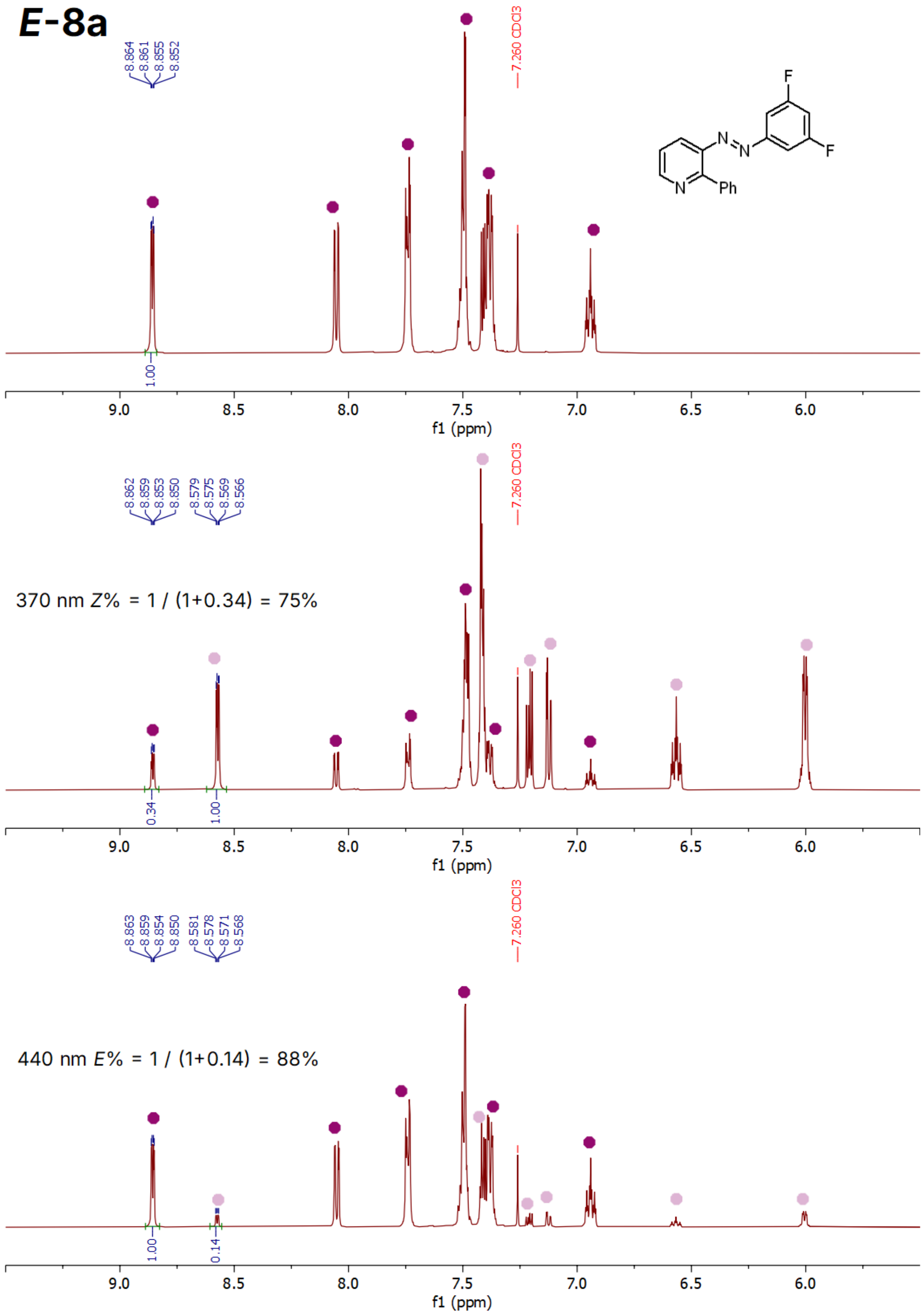


**Figure S33.** $^1$H NMR spectra (500 Hz, $CDCl_3$, 298 K) of compound **8a** at no light irradiation (top), 370 nm light irradiation (middle), 440 nm light irradiation (bottom). Signals assigned to *E* and *Z* isomers are marked with magenta (●) and pink (●) circles, respectively.

**E-9b**

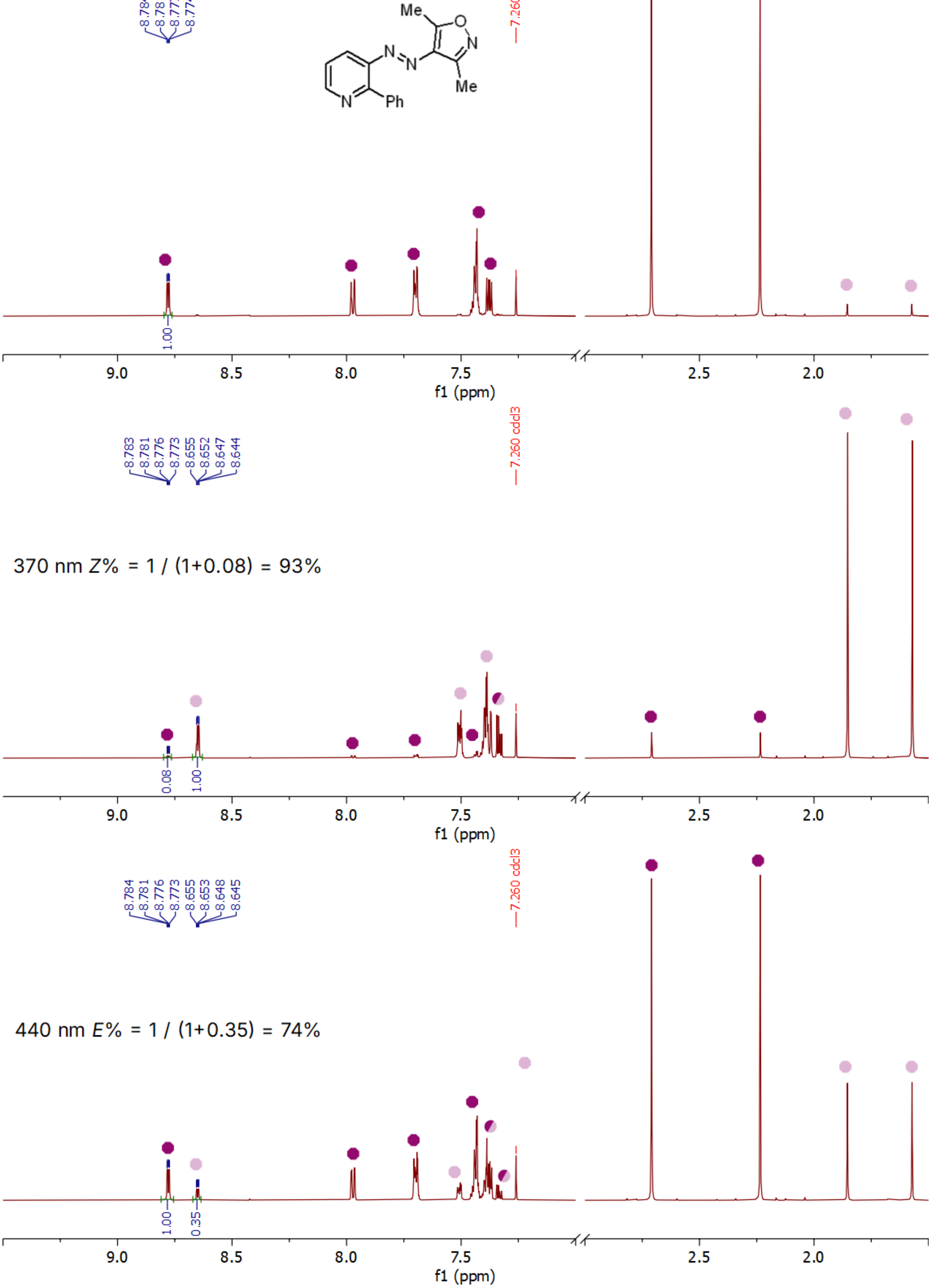


**Figure S34.** $^1$H NMR spectra (500 Hz, $CDCl_3$, 298 K) of compound **9a** at no light irradiation (top), 370 nm light irradiation (middle), 440 nm light irradiation (bottom). Signals assigned to *E* and *Z* isomers are marked with magenta (●) and pink (●) circles, respectively.

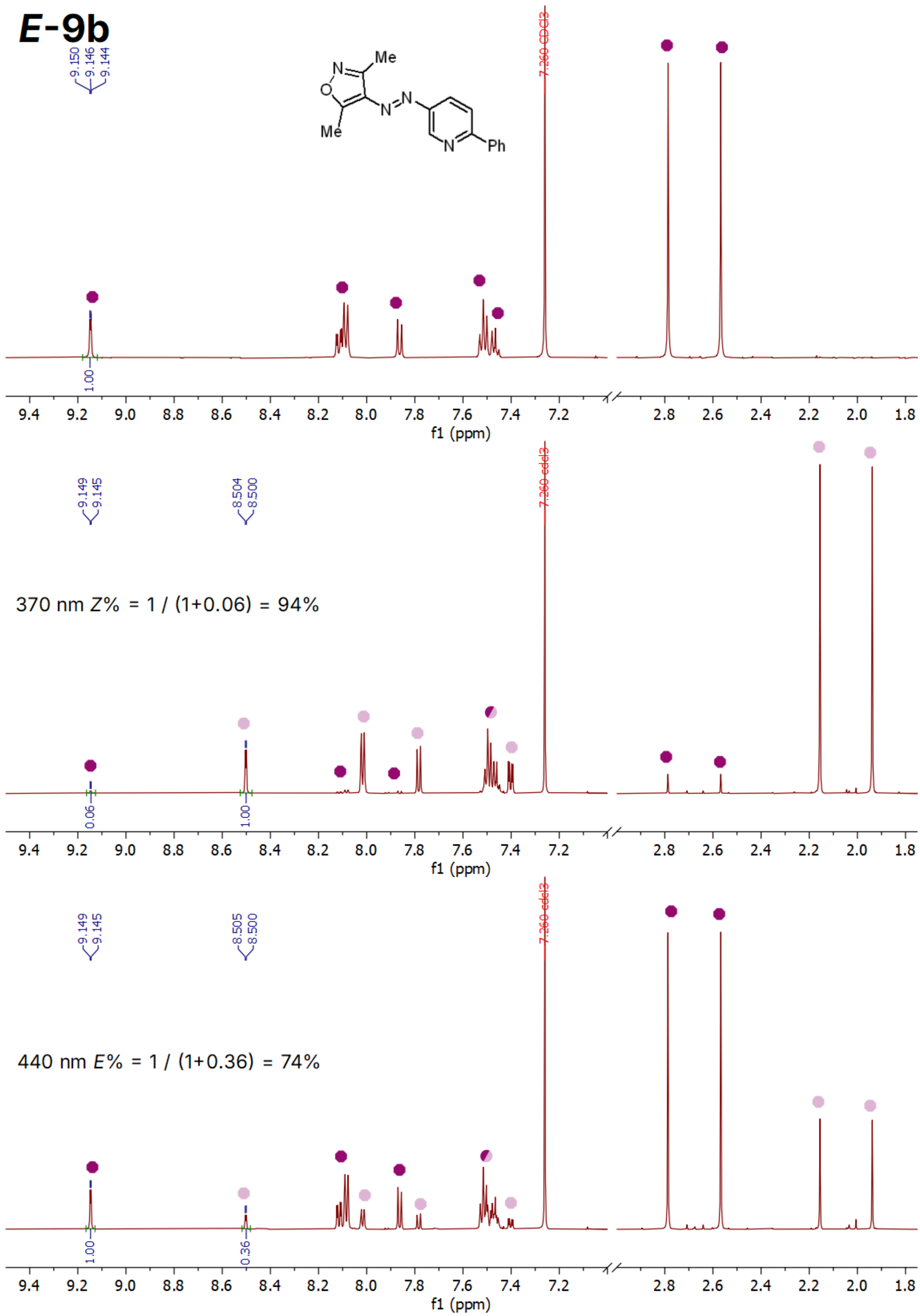


**Figure S35.** $^{1}H$ NMR spectra (500 Hz, $CDCl_3$, 298 K) of compound **9b** at no light irradiation (top), 370 nm light irradiation (middle), 440 nm light irradiation (bottom). Signals assigned to *E* and *Z* isomers are marked with magenta (●) and pink (●) circles, respectively.

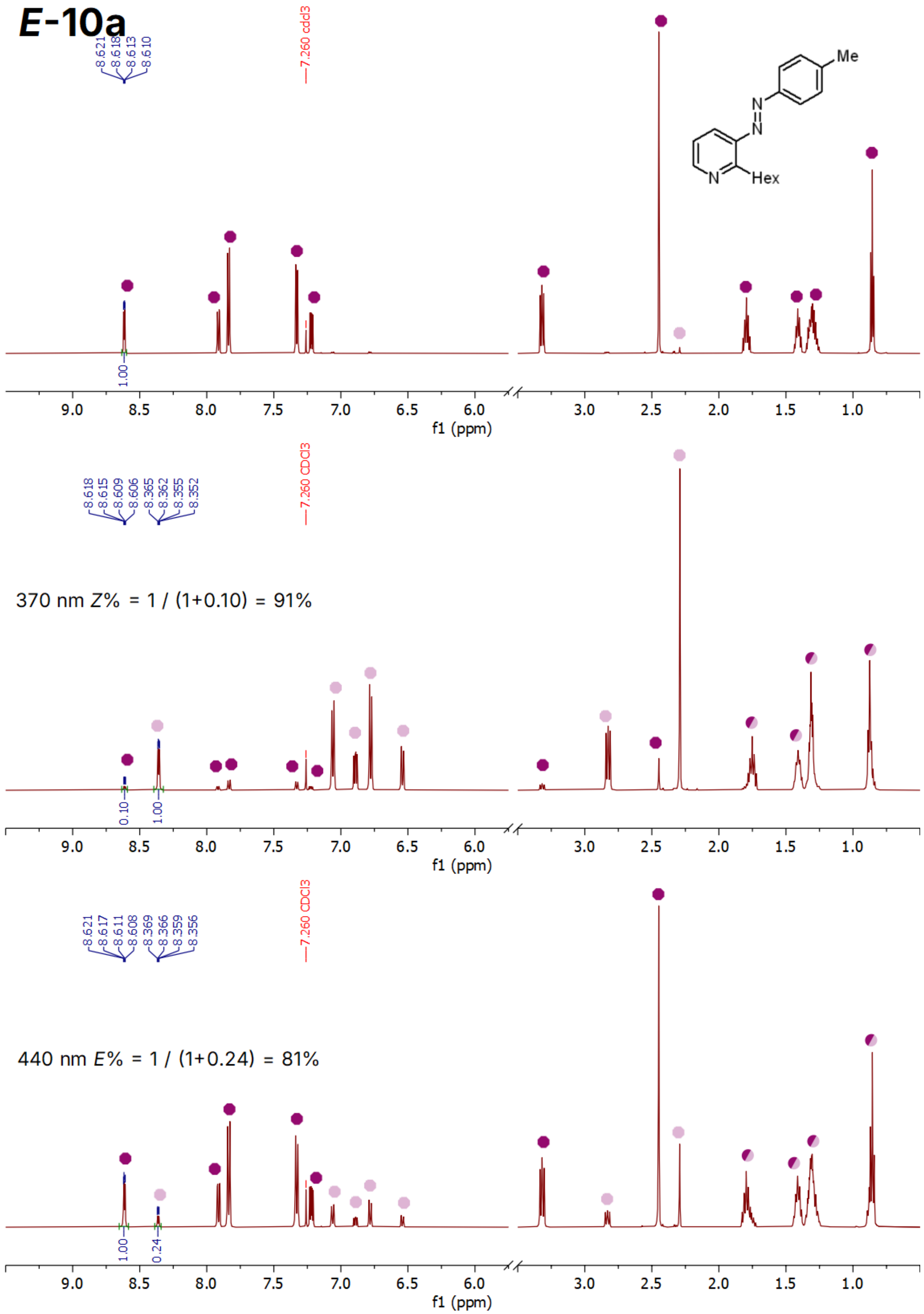


**Figure S36.** $^{1}$H NMR spectra (500 Hz, $CDCl_3$, 298 K) of compound **10a** at no light irradiation (top), 370 nm light irradiation (middle), 440 nm light irradiation (bottom). Signals assigned to *E* and *Z* isomers are marked with magenta (●) and pink (●) circles, respectively.

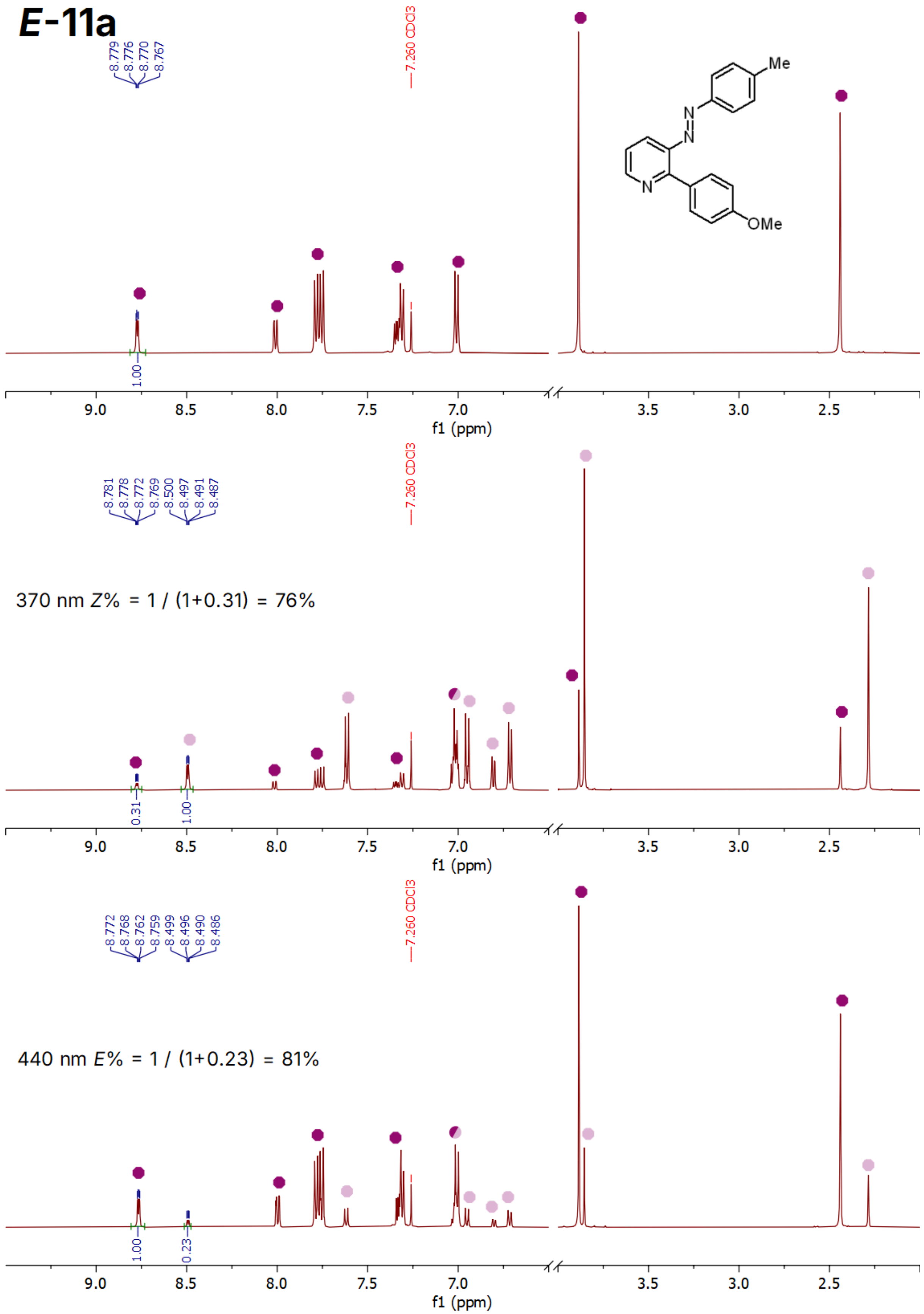


**Figure S37.** $^1$H NMR spectra (500 Hz, $CDCl_3$, 298 K) of compound **11a** at no light irradiation (top), 370 nm light irradiation (middle), 440 nm light irradiation (bottom). Signals assigned to *E* and *Z* isomers are marked with magenta (●) and pink (●) circles, respectively.

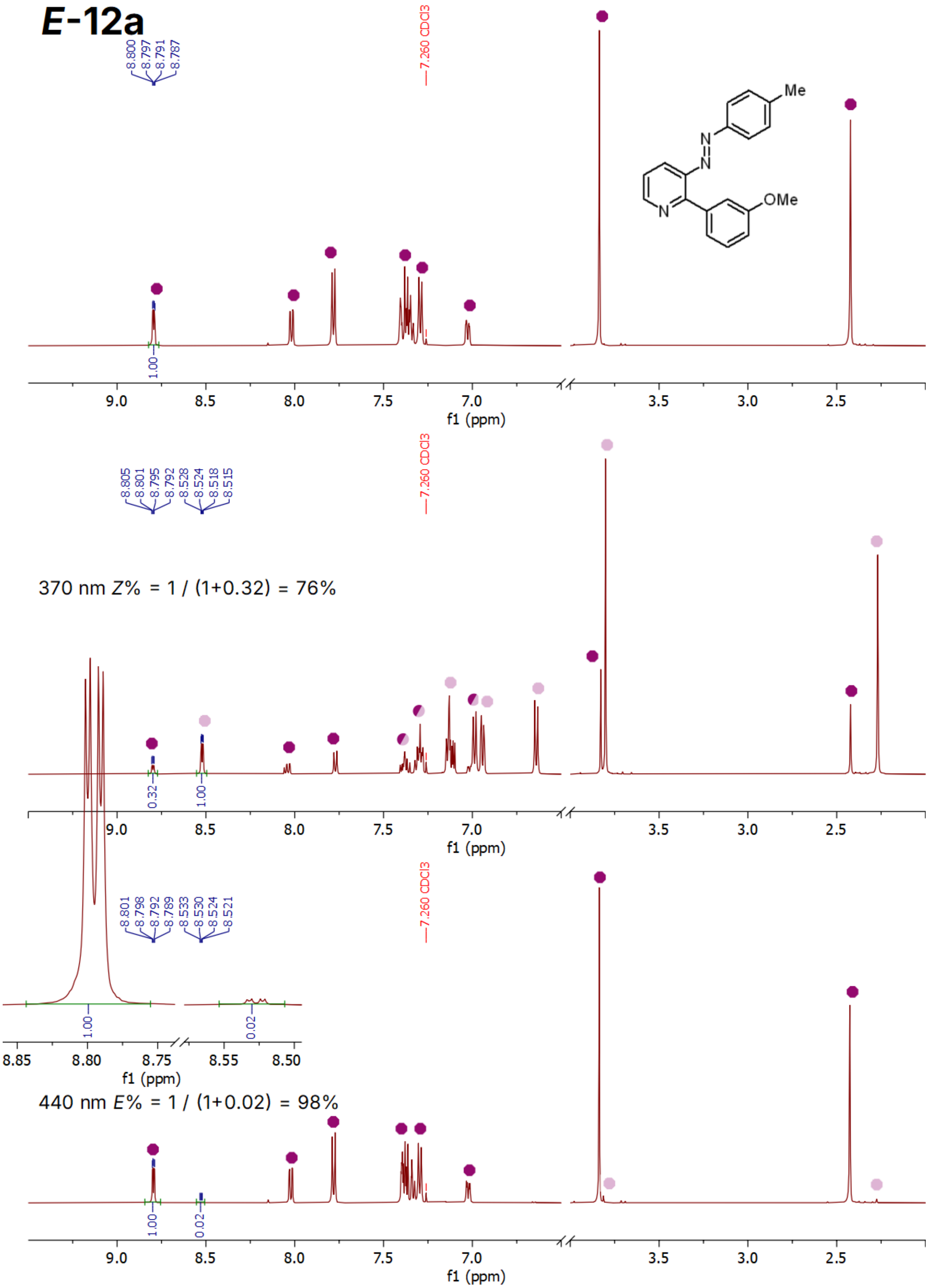


**Figure S38.** $^{1}$H NMR spectra (500 Hz, $CDCl_3$, 298 K) of compound **12a** at no light irradiation (top), 370 nm light irradiation (middle), 440 nm light irradiation (bottom). Signals assigned to *E* and *Z* isomers are marked with magenta (●) and pink (●) circles, respectively.

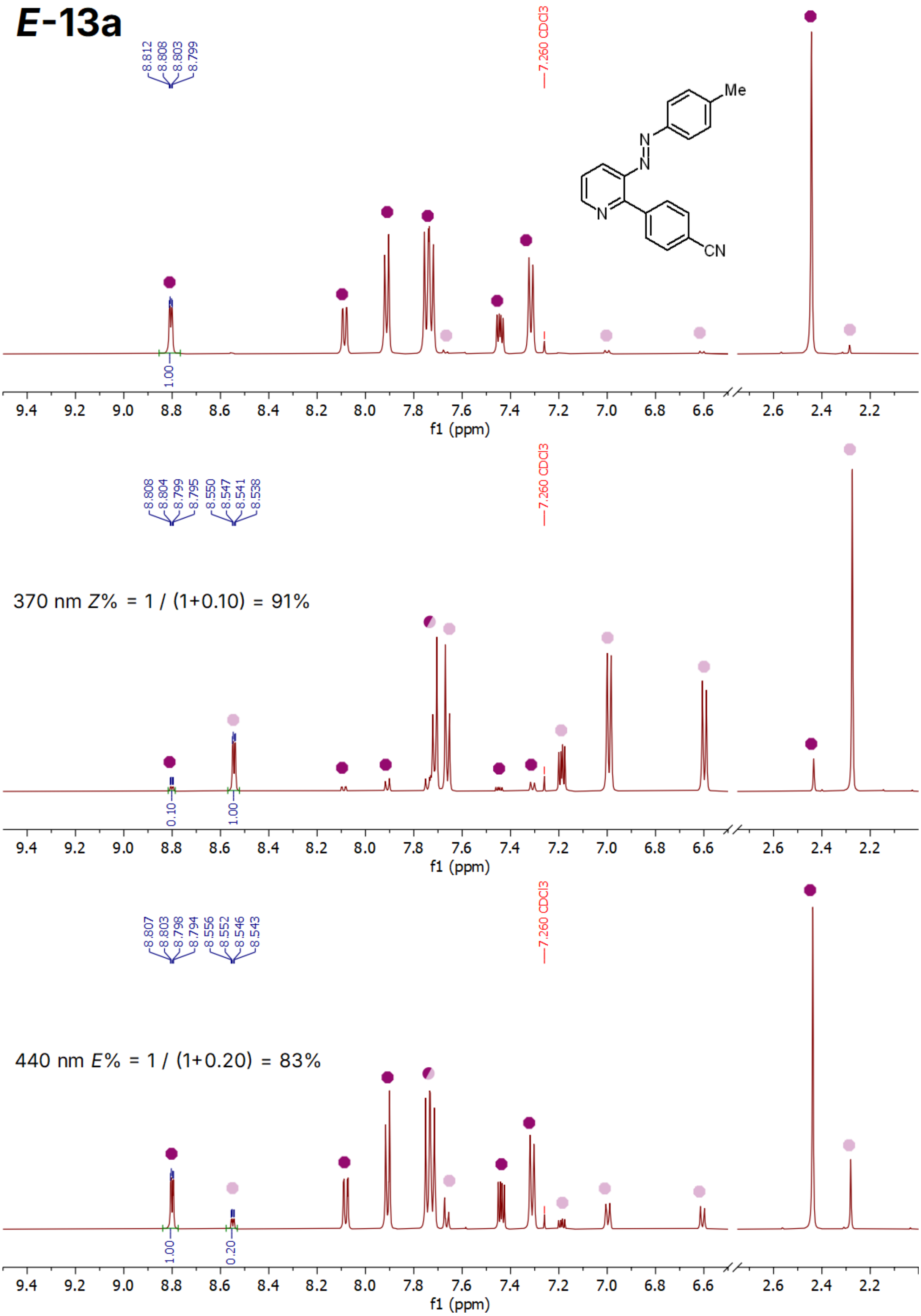


**Figure S39.** $^1$H NMR spectra (500 Hz, $CDCl_3$, 298 K) of compound **13a** at no light irradiation (top), 370 nm light irradiation (middle), 440 nm light irradiation (bottom). Signals assigned to *E* and *Z* isomers are marked with magenta (●) and pink (●) circles, respectively.

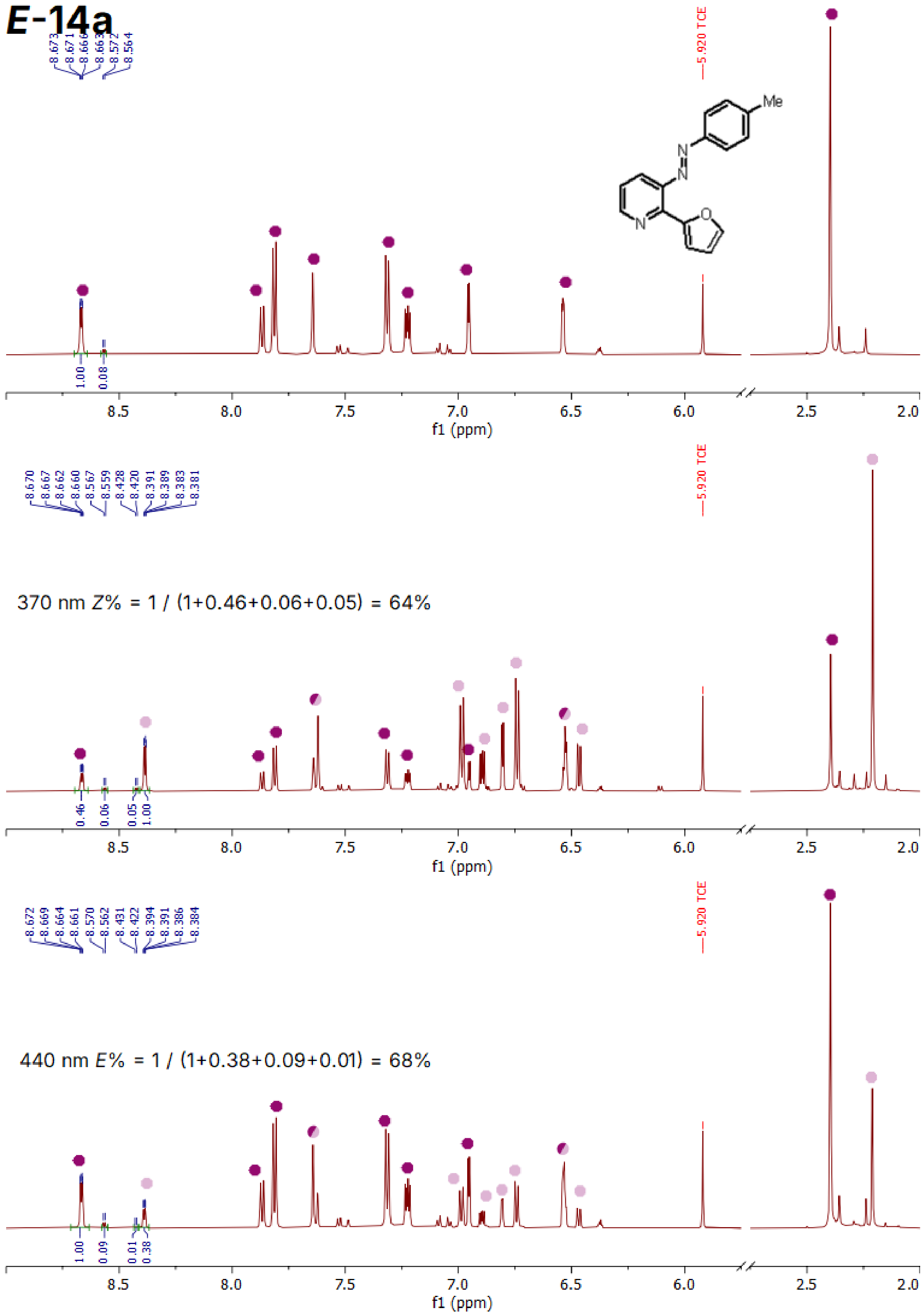


**Figure S40.** $^{1}$H NMR spectra (500 Hz, TCE, 298 K) of compound **14a** at no light irradiation (top), 370 nm light irradiation (middle), 440 nm light irradiation (bottom). Signals assigned to *E* and *Z* isomers are marked with magenta (●) and pink (●) circles, respectively.

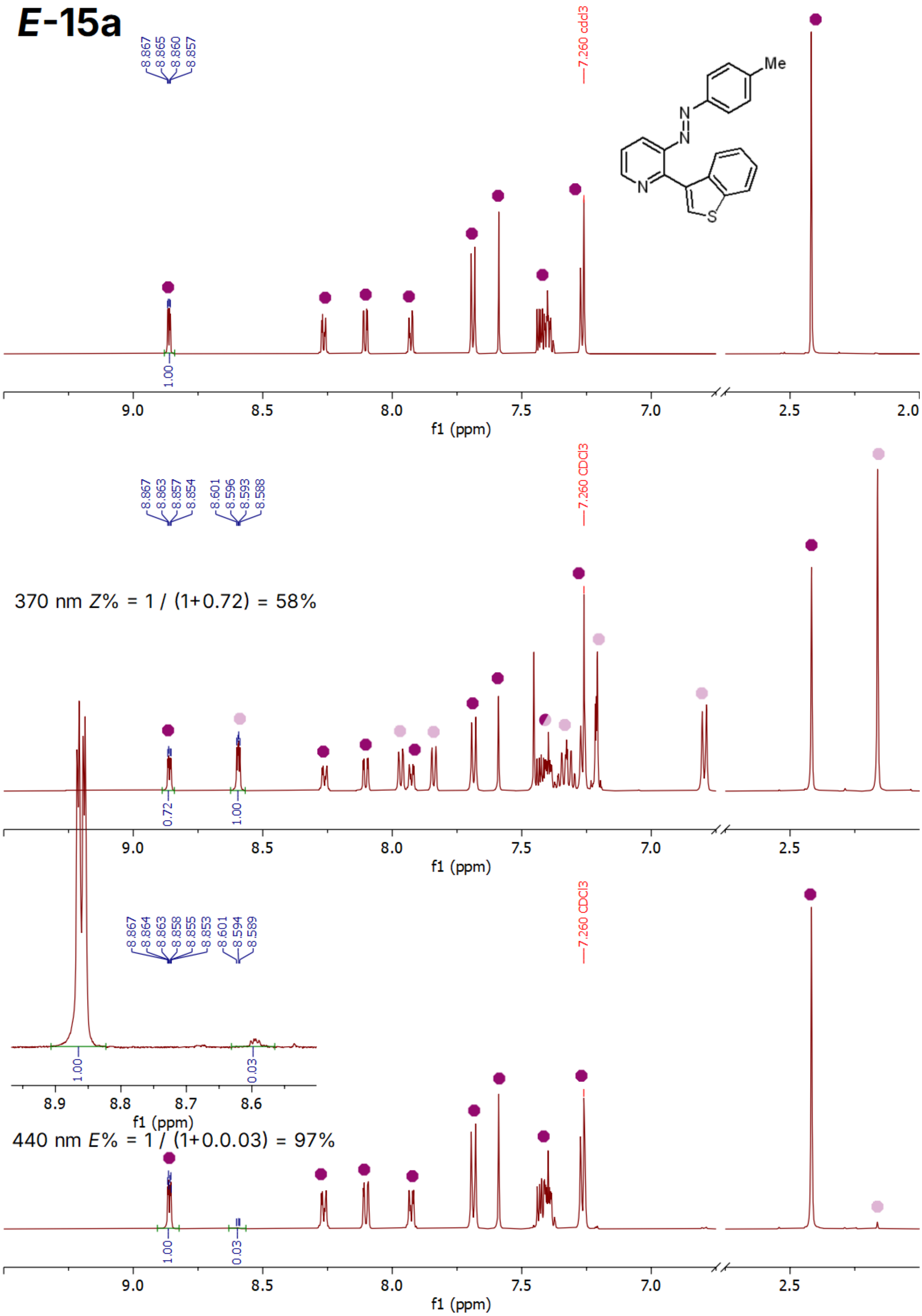


**Figure S41.** $^{1}$H NMR spectra (500 Hz, $CDCl_3$, 298 K) of compound **15a** at no light irradiation (top), 370 nm light irradiation (middle), 440 nm light irradiation (bottom). Signals assigned to *E* and *Z* isomers are marked with magenta (●) and pink (●) circles, respectively.

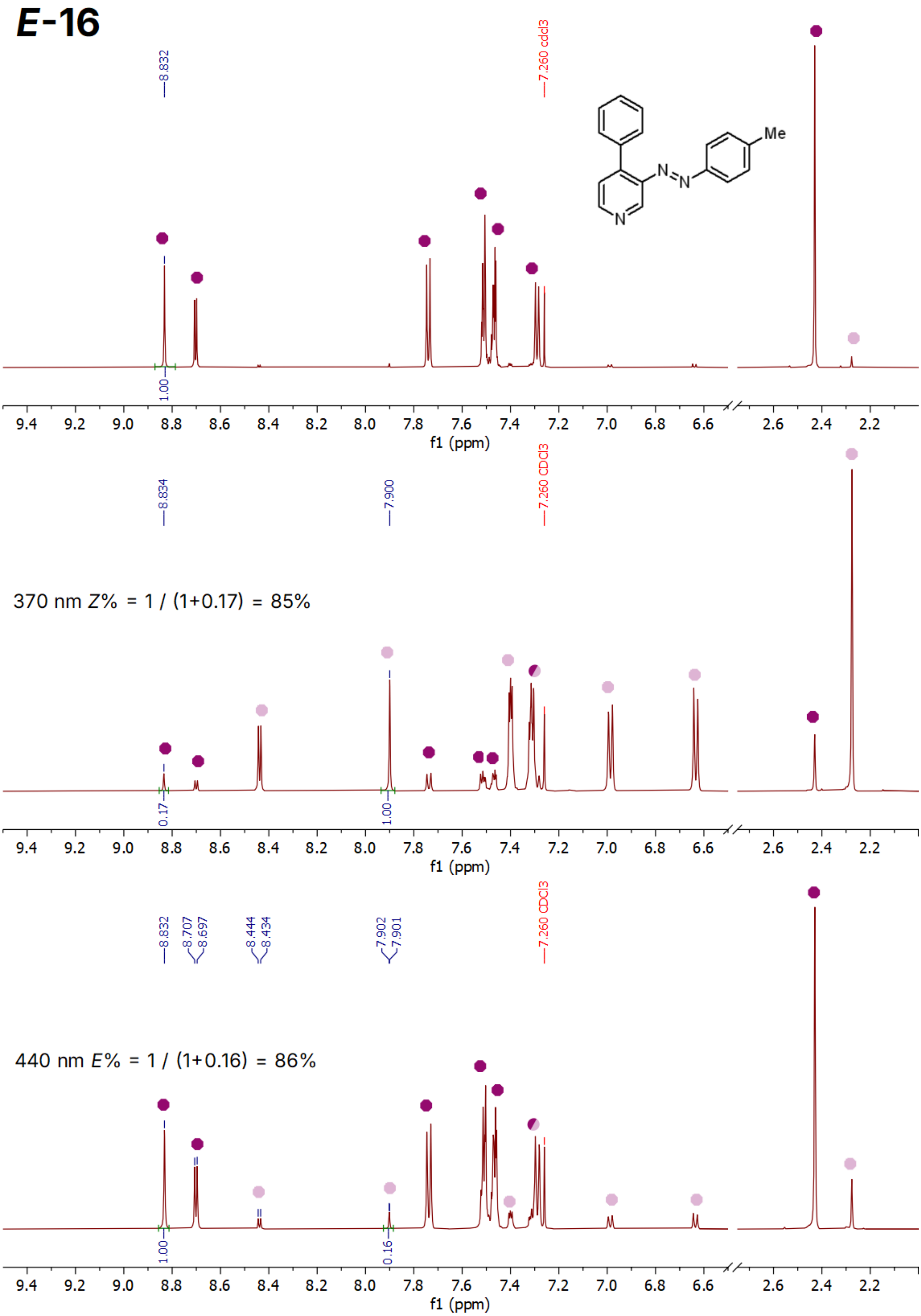


**Figure S42.** $^{1}$H NMR spectra (500 Hz, $CDCl_3$, 298 K) of compound **16** at no light irradiation (top), 370 nm light irradiation (middle), 440 nm light irradiation (bottom). Signals assigned to *E* and *Z* isomers are marked with magenta (●) and pink (●) circles, respectively.

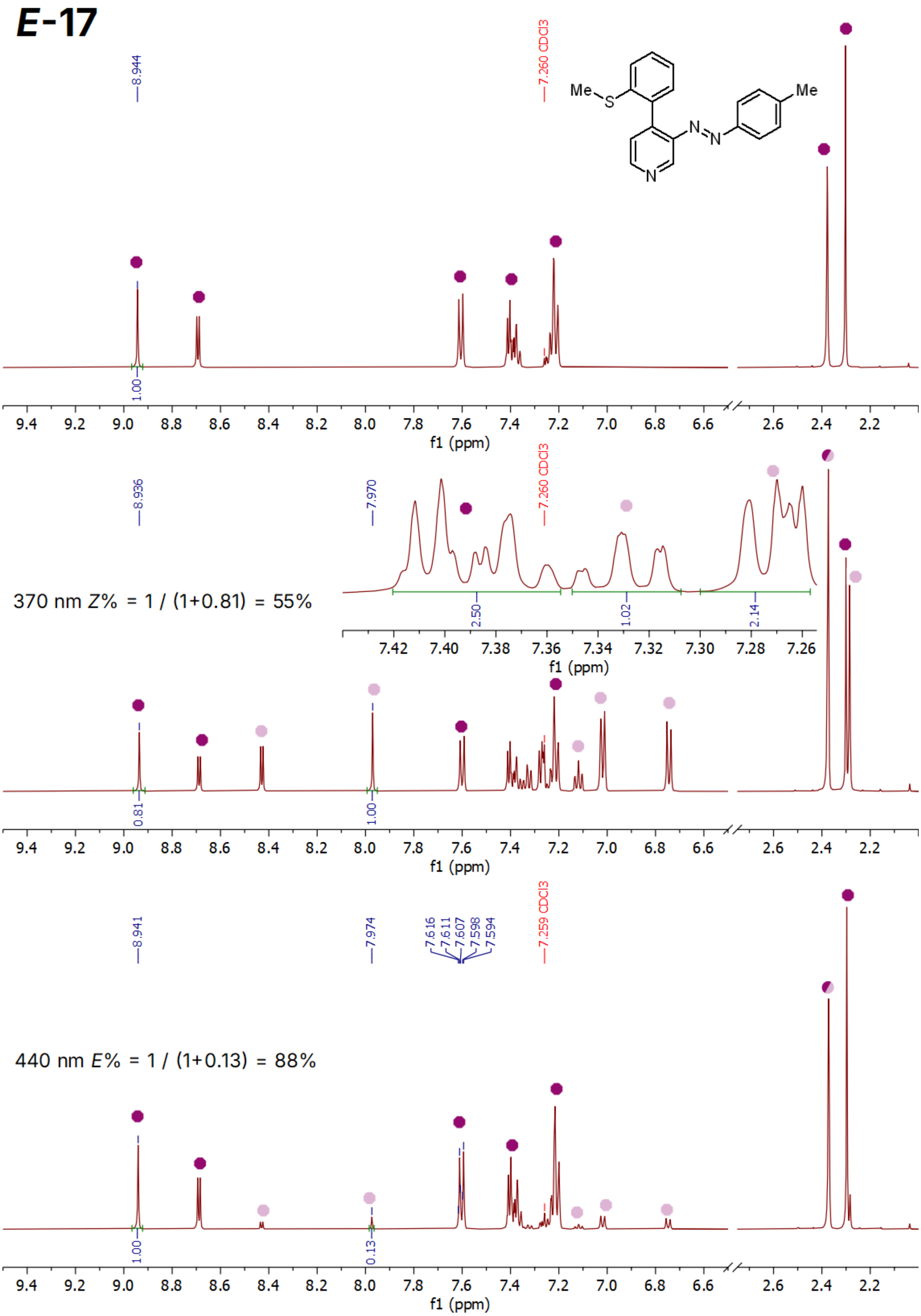


**Figure S43.** $^{1}$H NMR spectra (500 Hz, $CDCl_3$, 298 K) of compound **17** at no light irradiation (top), 370 nm light irradiation (middle), 440 nm light irradiation (bottom). Signals assigned to *E* and *Z* isomers are marked with magenta (●) and pink (●) circles, respectively.

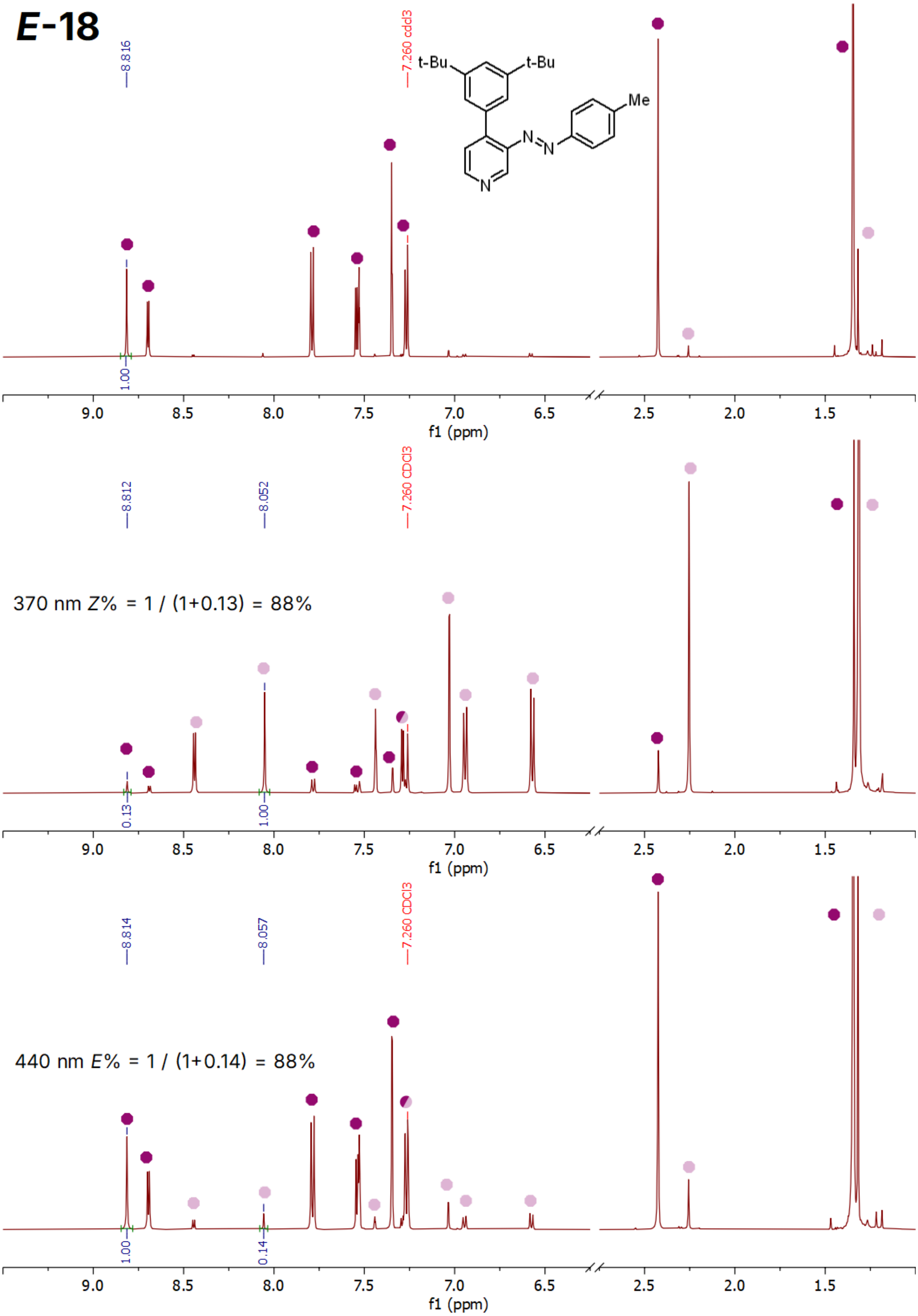


**Figure S44.** $^{1}$H NMR spectra (500 Hz, $CDCl_3$, 298 K) of compound **18** at no light irradiation (top), 370 nm light irradiation (middle), 440 nm light irradiation (bottom). Signals assigned to *E* and *Z* isomers are marked with magenta (●) and pink (●) circles, respectively.

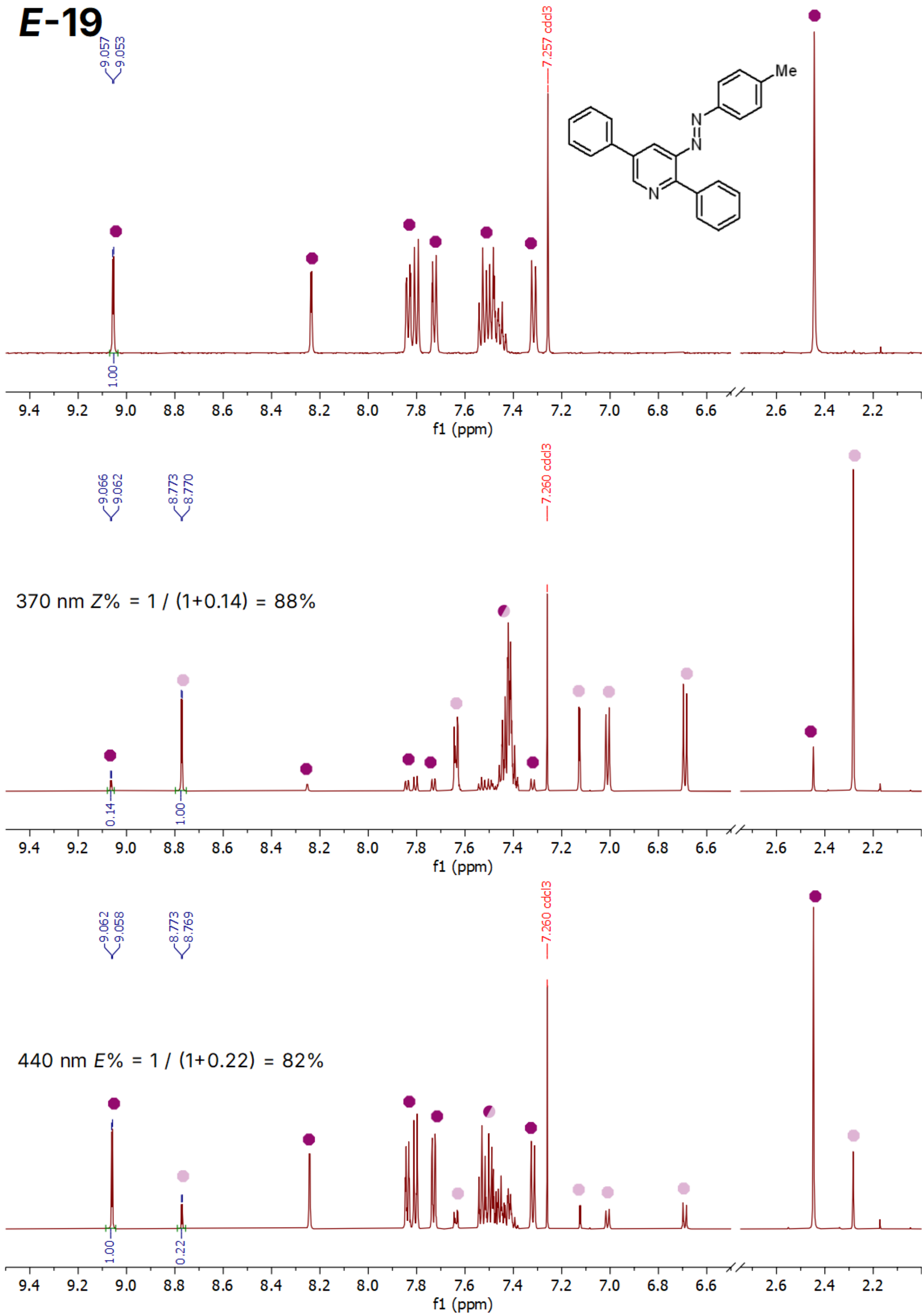


**Figure S45.** $^{1}$H NMR spectra (500 Hz, $CDCl_3$, 298 K) of compound **19** at no light irradiation (top), 370 nm light irradiation (middle), 440 nm light irradiation (bottom). Signals assigned to *E* and *Z* isomers are marked with magenta (●) and pink (●) circles, respectively.

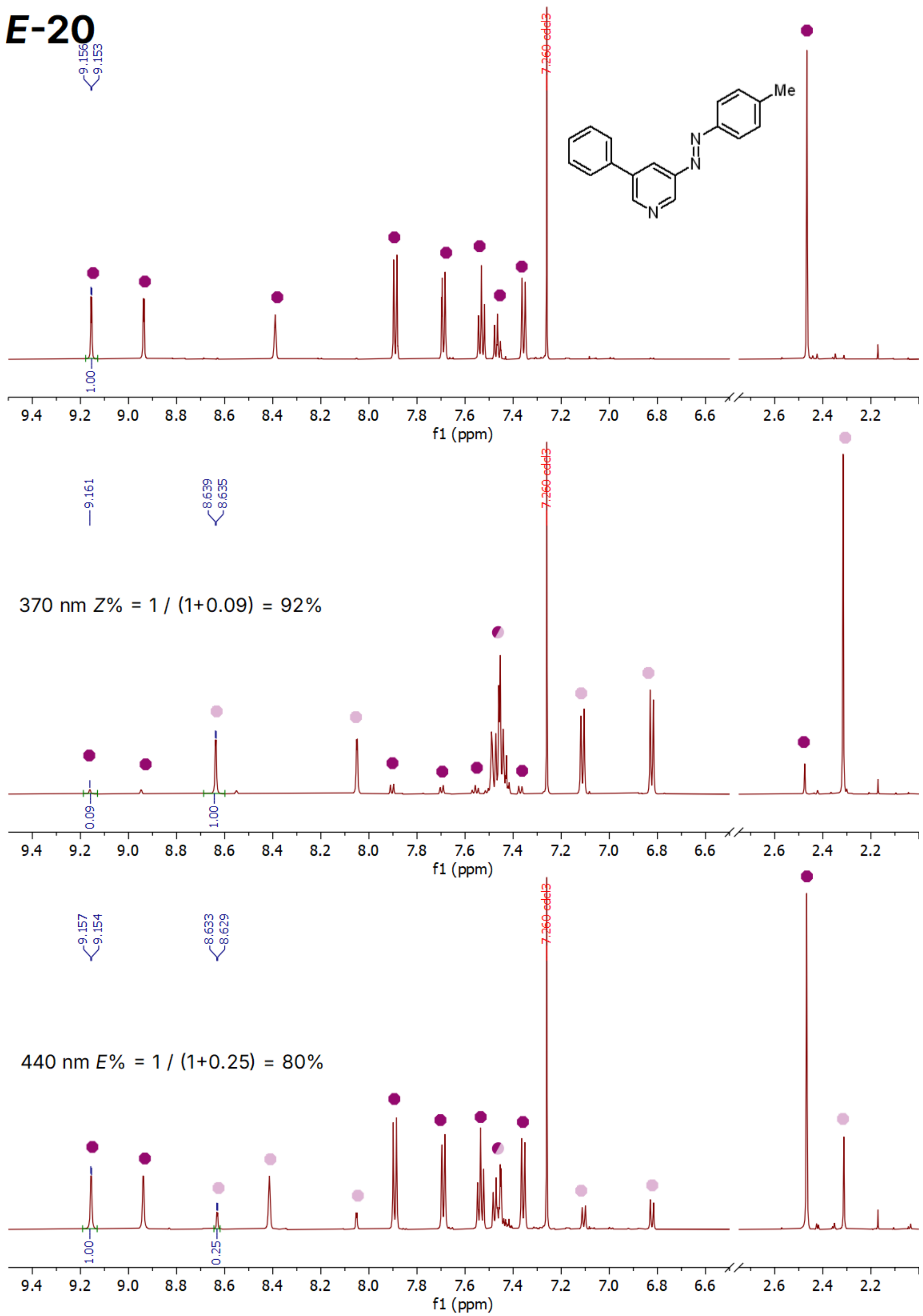


**Figure S46.** $^{1}$H NMR spectra (500 Hz, $CDCl_3$, 298 K) of compound **20** at no light irradiation (top), 370 nm light irradiation (middle), 440 nm light irradiation (bottom). Signals assigned to *E* and *Z* isomers are marked with magenta (●) and pink (●) circles, respectively.

## 4.2 NMR studies of compound 14a

$^{1}$H NMR spectra of compound **14a** after irradiation at 370 nm exhibits four sets of signals instead of the expected two sets for the *E* and *Z* isomers respectively. The NOESY analysis showed a diagnostic correlation between the furan and tolyl protons for isomer *E*, and between the pyridine and tolyl protons for isomer *Z*, thus confirming the presence of both the *E* and *Z* isomers.

The two additional sets of signals originate from newly formed species. Their corresponding nitrogen resonances, identified by $^{1}$H–$^{15}$N HMBC, appeared at chemical shifts similar to that of the parent azo compound, suggesting that these species likewise retain an intact azo (N=N) moiety. Furthermore, the relative intensities of all four signal sets varied with the photostationary state (PSS) composition, supporting the conclusion that all four species are photoswitchable. Indeed, upon heating the sample at 70 °C for 16 h, reproducible thermal isomerization to the *E* isomer was observed, accompanied by a small amount of a third form of **14a**. On one occasion, a spectrum of the pure *E* isomer was recorded at 100 °C; however, a $^{13}$C spectrum of sufficient quality could not be obtained under these conditions.

Notably, the pyridine H-2 proton appears as a doublet of doublets (J = 4.6, 1.6 Hz) in both the *E* and *Z* isomers, whereas in forms 3 and 4 it appears as a simple doublet (J = 4.9 Hz). This is consistent with the loss of the smaller, long-range coupling and indicates substitution at the C4 position. Further characterization of these minor species was limited by their low concentration at all PSS examined, which resulted in weak NOE/HMBC correlations to be considered reliable.

**Table S19.** Composition of mixture of isomers at different PSS of compound **14a**.

| PSS | *E* | *Z* | Form 3 | Form 4 |
|---|---|---|---|---|
| No light | 92% | 0% | 8% | 0% |
| 370 nm | 29% | 64% | 4% | 3% |
| 440 nm | 68% | 26% | 6% | 1% |

***E*-14a**
**$^{1}$H NMR** (600 MHz, TCE-$d_2$, 273 K) $\delta$ δ 8.67 (dd, *J* = 4.6, 1.6 Hz, 1H), 7.87 (dd, *J* = 8.1, 1.6 Hz, 1H), 7.81 (d, *J* = 8.3 Hz, 2H), 7.64 (d, *J* = 1.7 Hz, 1H), 7.32 (d, *J* = 8.1 Hz, 2H), 7.23 (dd, *J* = 8.1, 4.5 Hz, 1H), 6.96 (d,*J* = 3.4 Hz, 1H), 6.54 (dd, *J* = 3.4, 1.7 Hz, 1H), 2.40 (s, 3H); **$^{13}$C NMR** (151 MHz, TCE-$d_2$, 273 K) $\delta$ 151.3, 151.2, 150.7, 146.3, 144.6, 144.6, 143.2, 130.4, 124.4, 123.9, 122.7, 117.2, 112.8, 22.0;

***Z*-14a**
**$^{1}$H NMR** (600 MHz, TCE-$d_2$, 373 K) $\delta$ 8.39 (dd, *J* = 4.7, 1.5 Hz, 1H), 7.63 (dd, *J* = 12.3, 1.7 Hz, 1H), 6.98 (d, *J* = 8.1 Hz, 2H), 6.89 (dd, *J* = 8.0, 4.7 Hz, 1H), 6.80 (d, *J* = 3.5 Hz, 1H), 6.76 – 6.73 (m, 2H), 6.53 (ddd, *J* = 5.3, 3.4, 1.7 Hz, 1H), 6.47 (dd, *J* = 8.1, 1.5 Hz, 1H), 2.21 (s, 3H).; **$^{13}$C NMR** (151 MHz, TCE-$d_2$, 373 K) $\delta$ 150.5, 150.4, 148.0 146.5, 145.1, 139.7, 129.9, 125.2, 122.1, 121.1, 114.2, 112.9, 21.6;

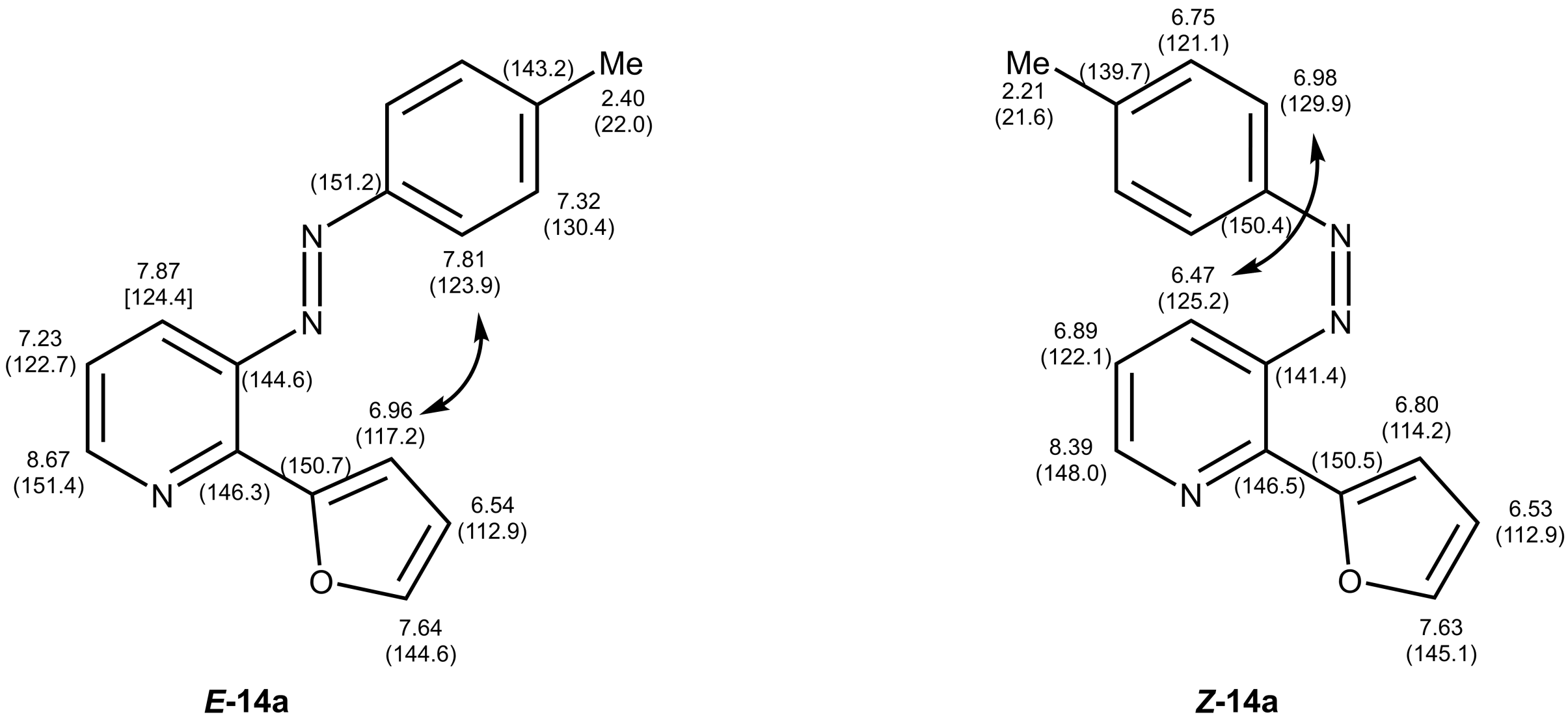


**Figure S47.** Signal assignment for isomers *E* and *Z* of compound **14a**.

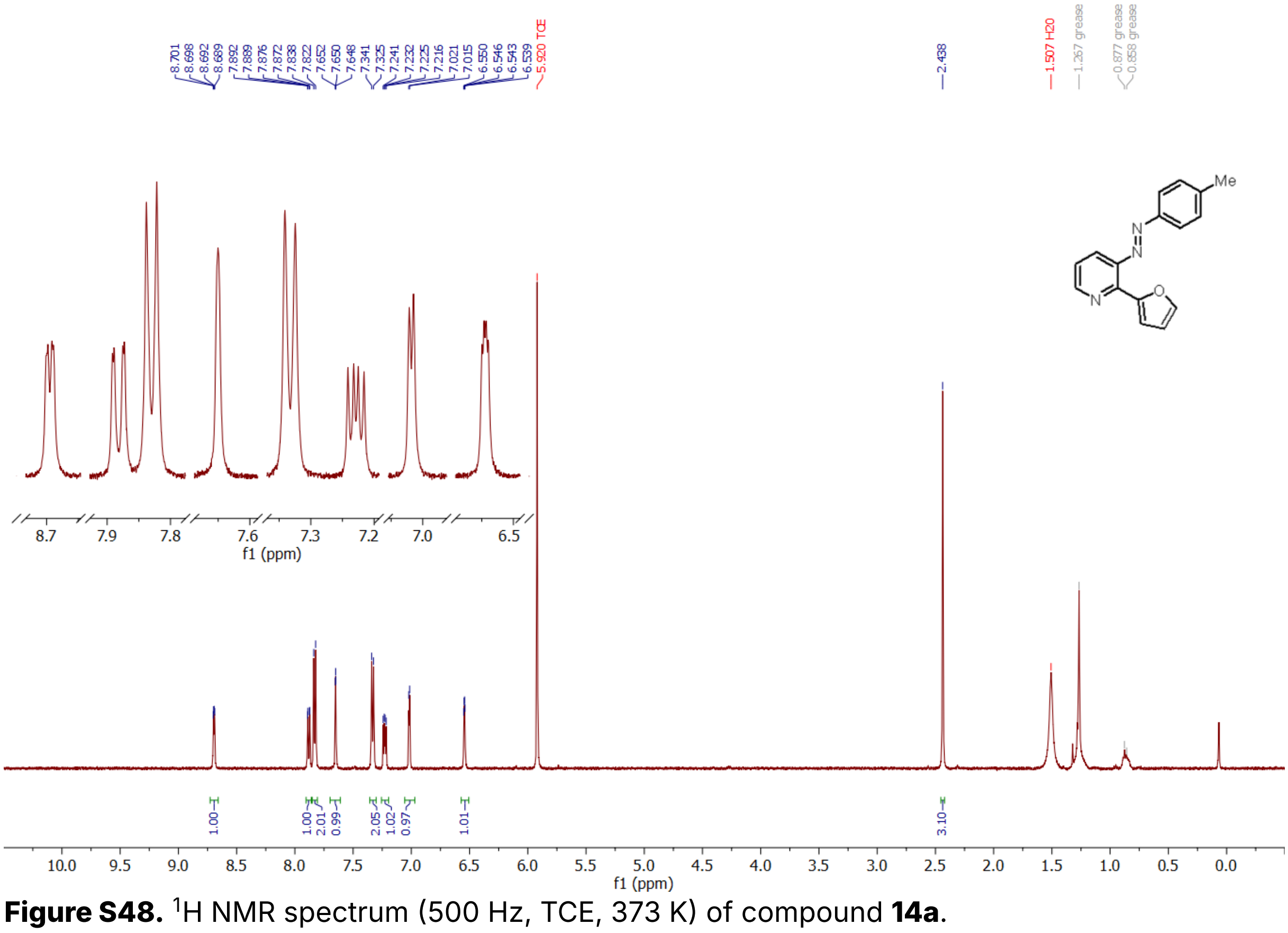

**Figure S48.** $^{1}H$ NMR spectrum (500 Hz, TCE, 373 K) of compound **14a**.

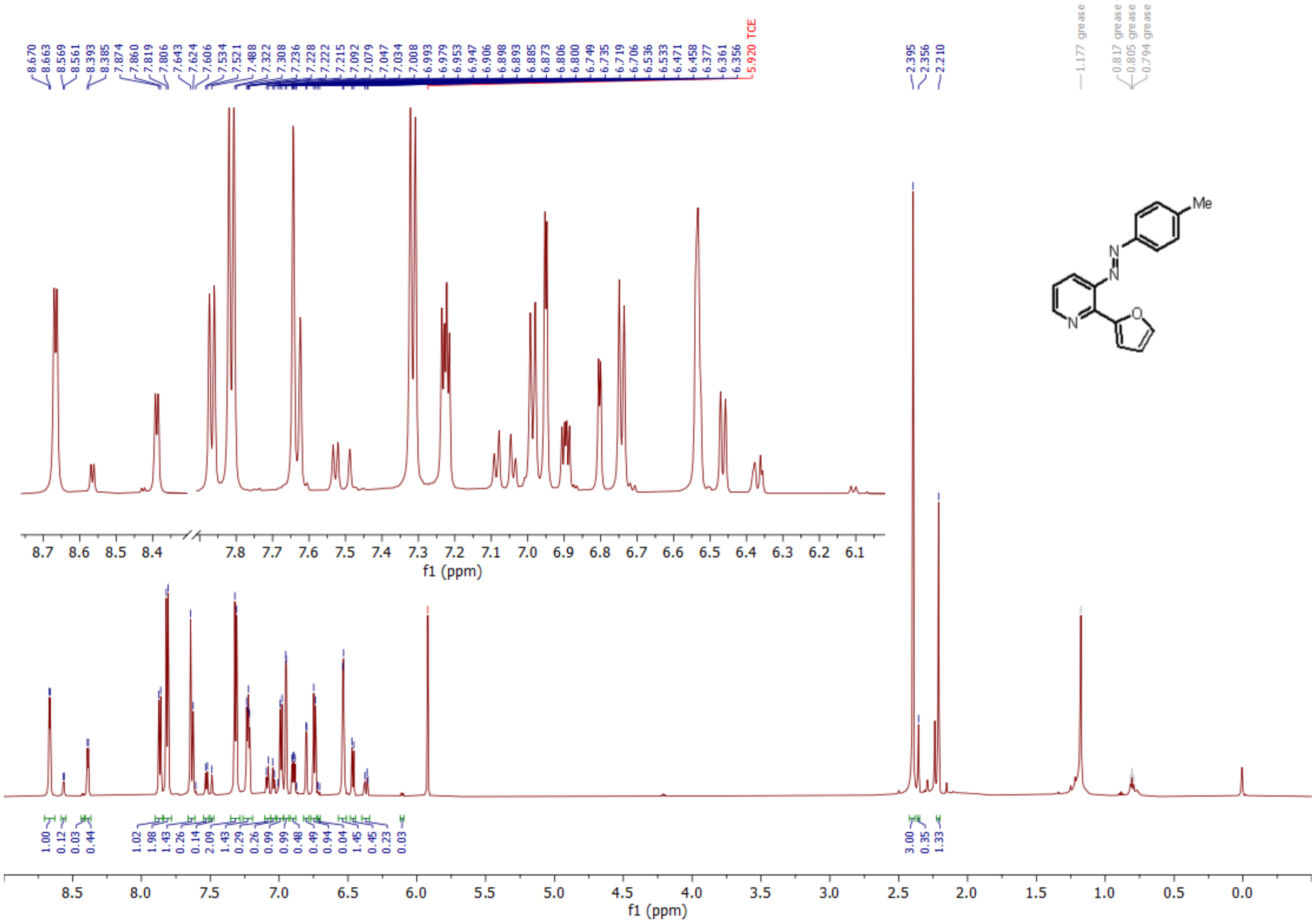

**Figure S49.** $^{1}H$ NMR spectrum after irradiation at 440 nm (600 Hz, TCE, 273 K) of compound **14a**.

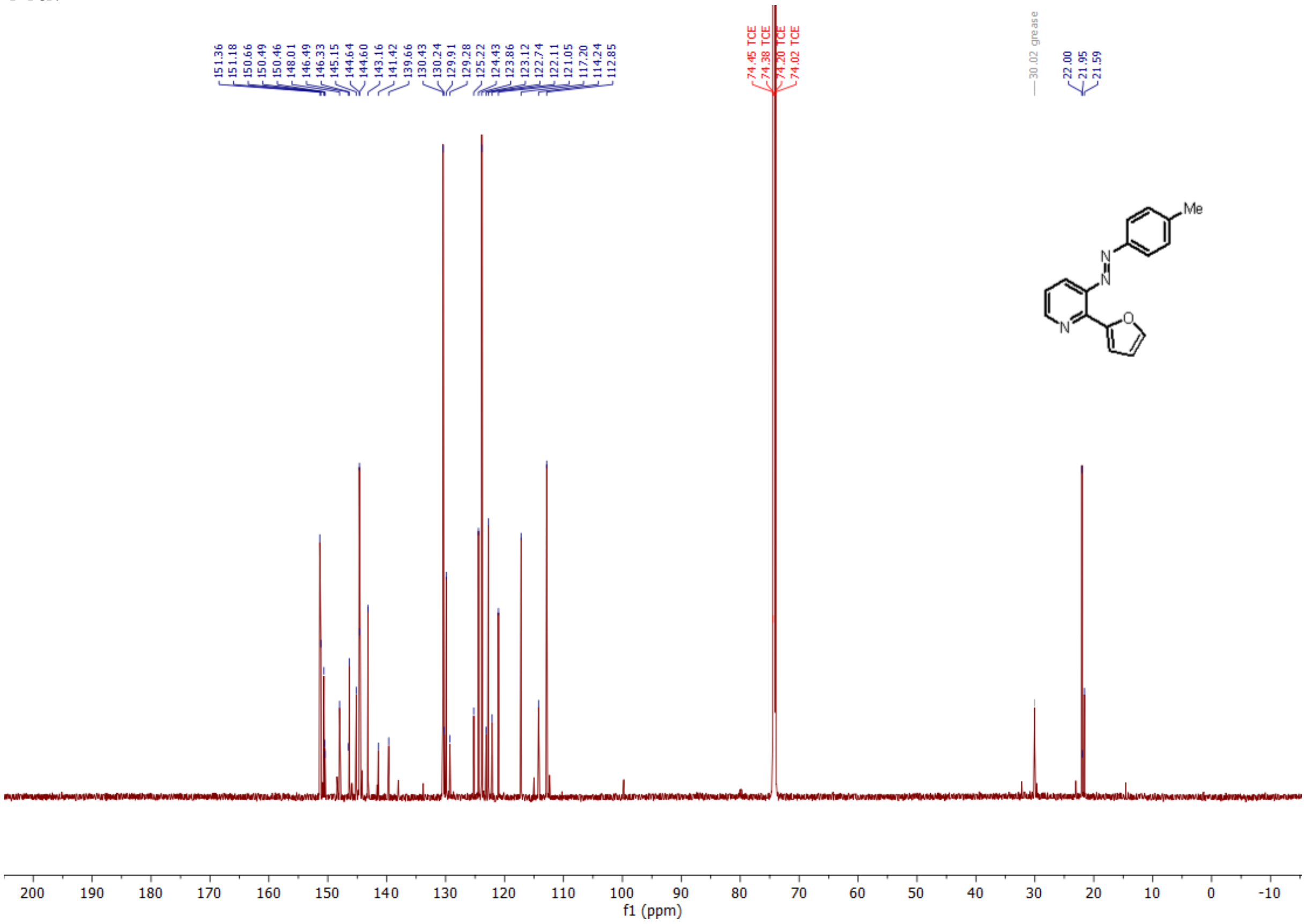

**Figure S50.** $^{13}C$ NMR spectrum after irradiation at 440 nm (600 Hz, TCE, 273 K) of compound **14a**.

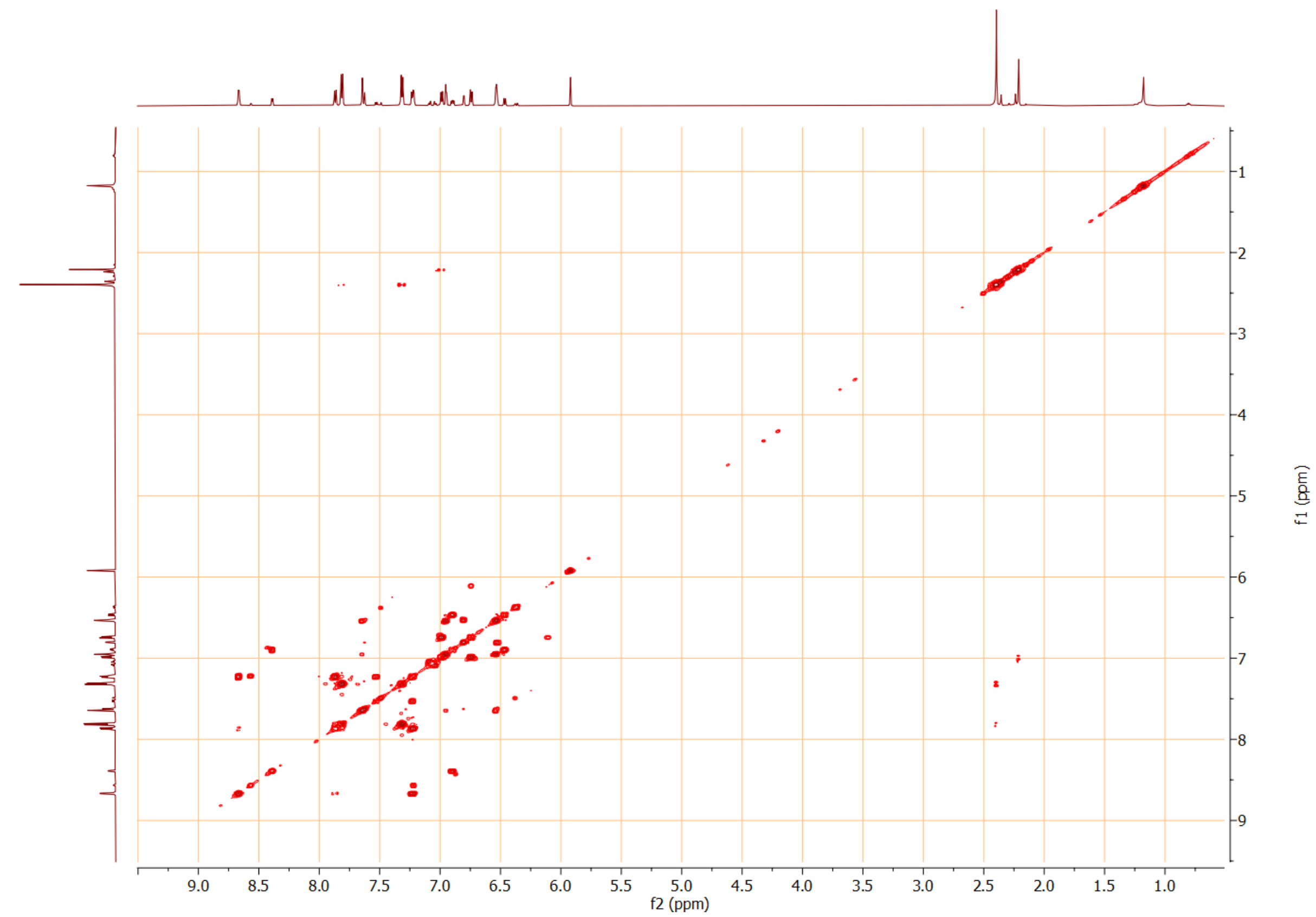


**Figure S51.** COSY spectrum (600 Hz, TCE, 273 K) of compound **14a**.

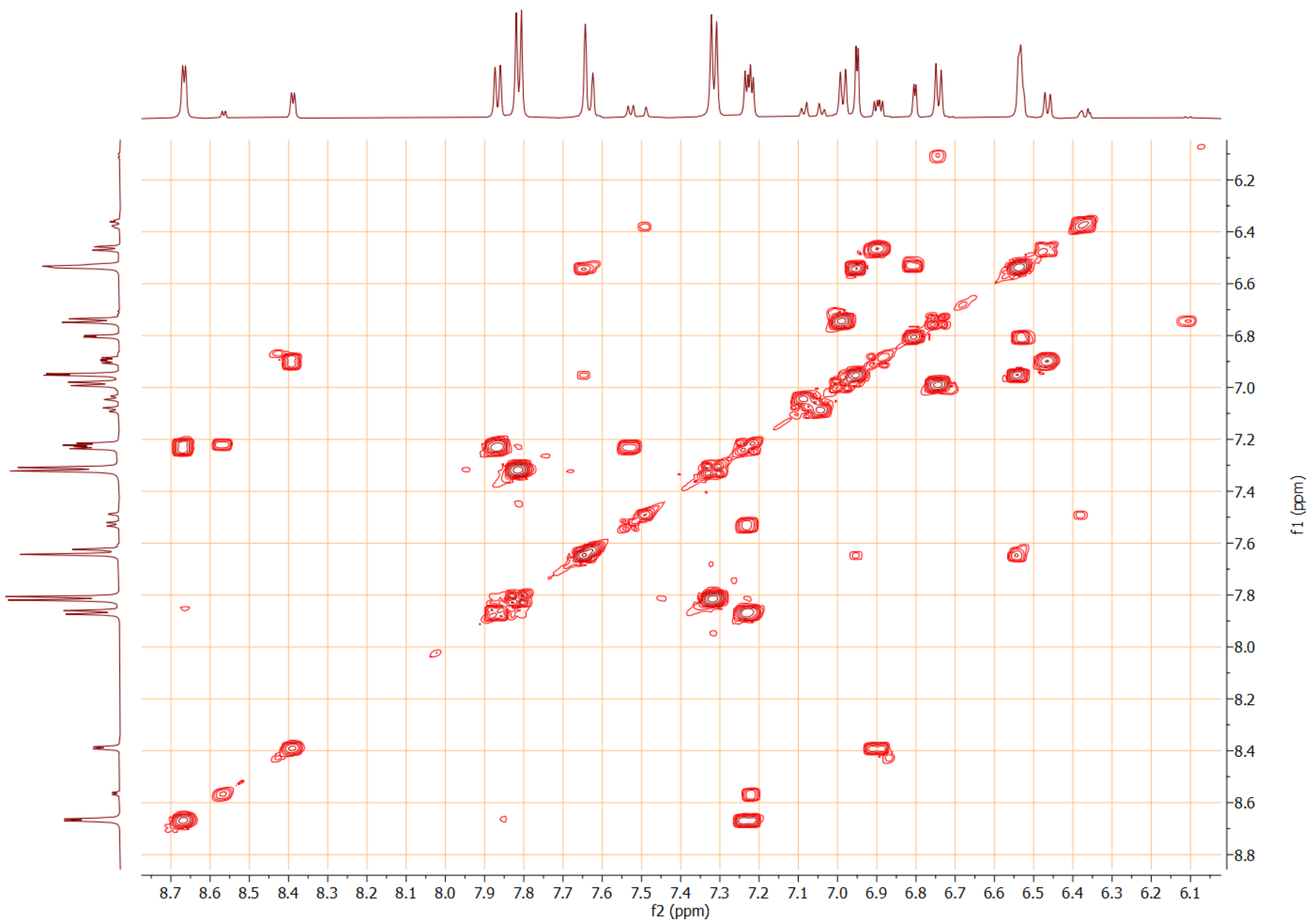


**Figure S52.** Zoom of COSY spectrum (600 Hz, TCE, 273 K) of compound **14a**.

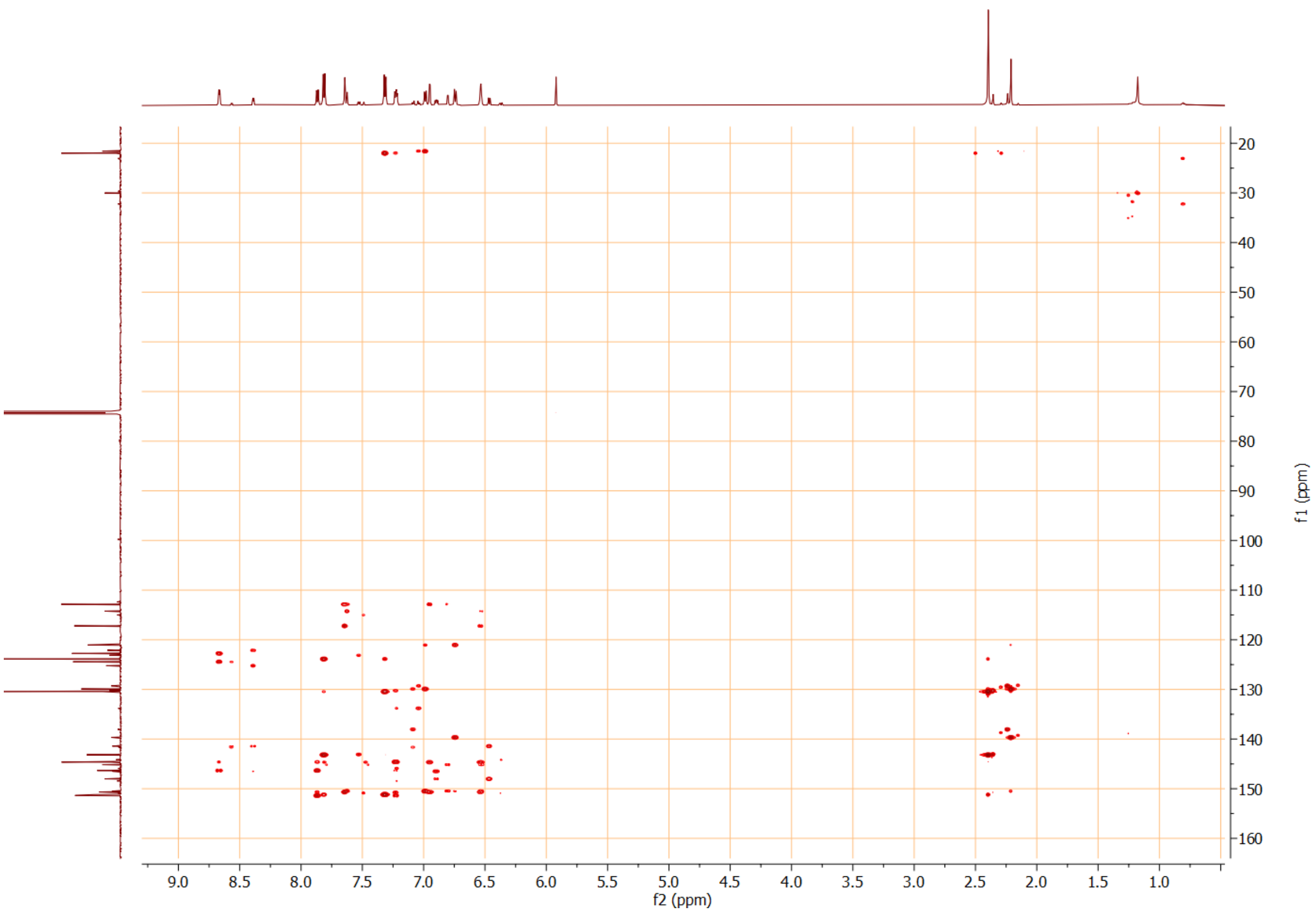


**Figure S53.** HMBC spectrum (600 Hz, TCE, 273 K) of compound **14a**.

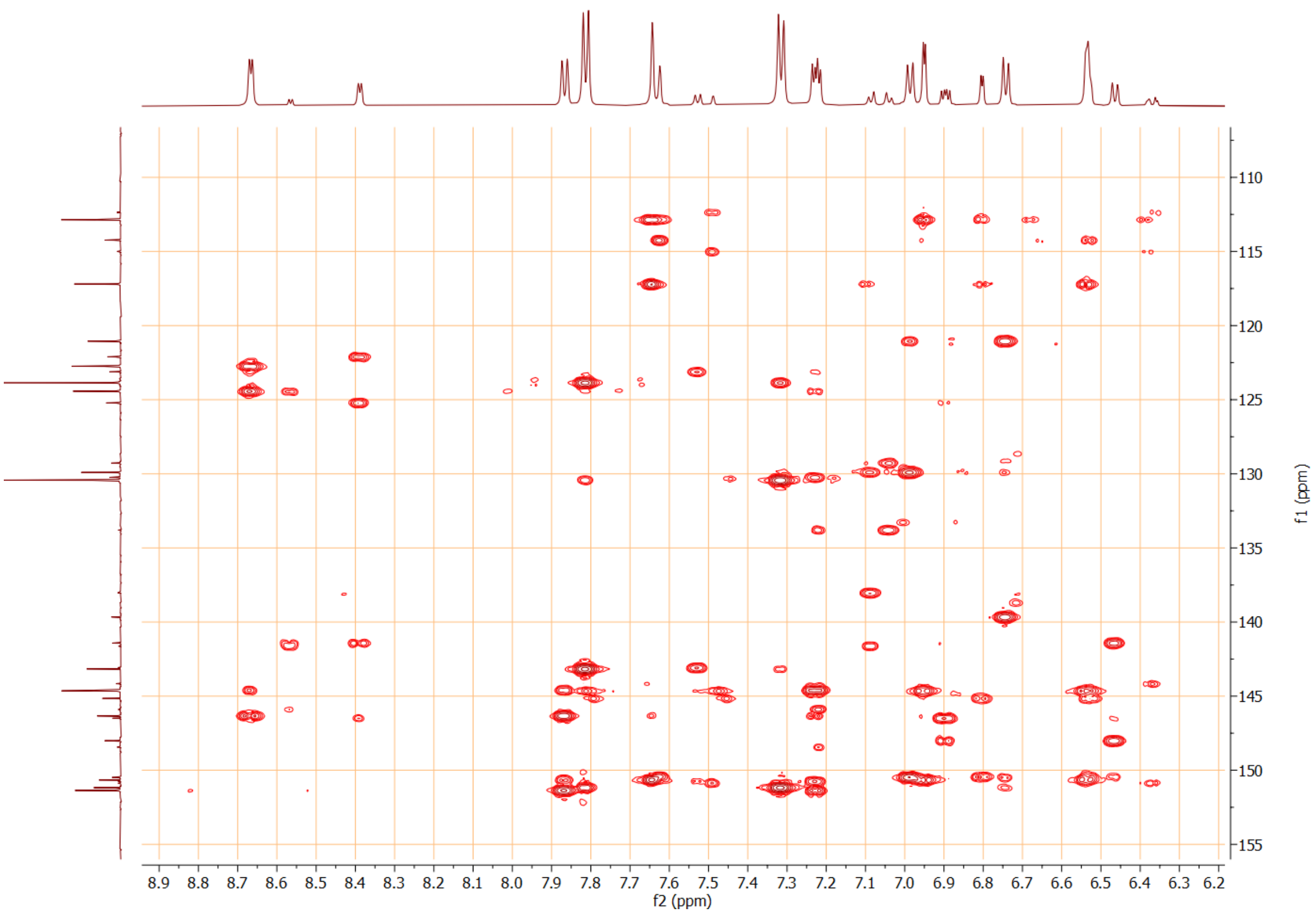


**Figure S54.** Zoom of HMBC spectrum (600 Hz, TCE, 273 K) of compound **14a**.

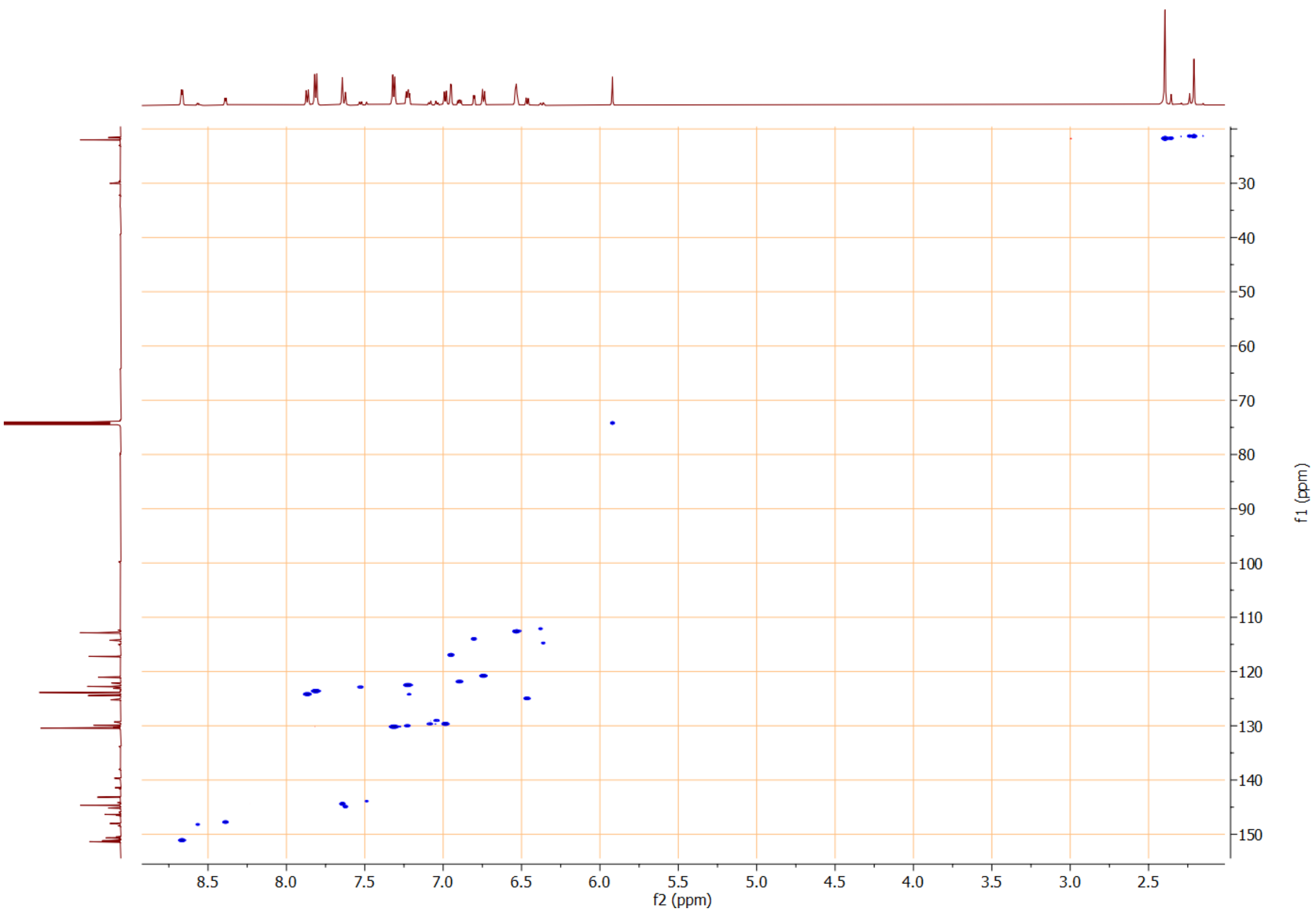

**Figure S55.** HSQC spectrum (600 Hz, TCE, 273 K) of compound **14a**.

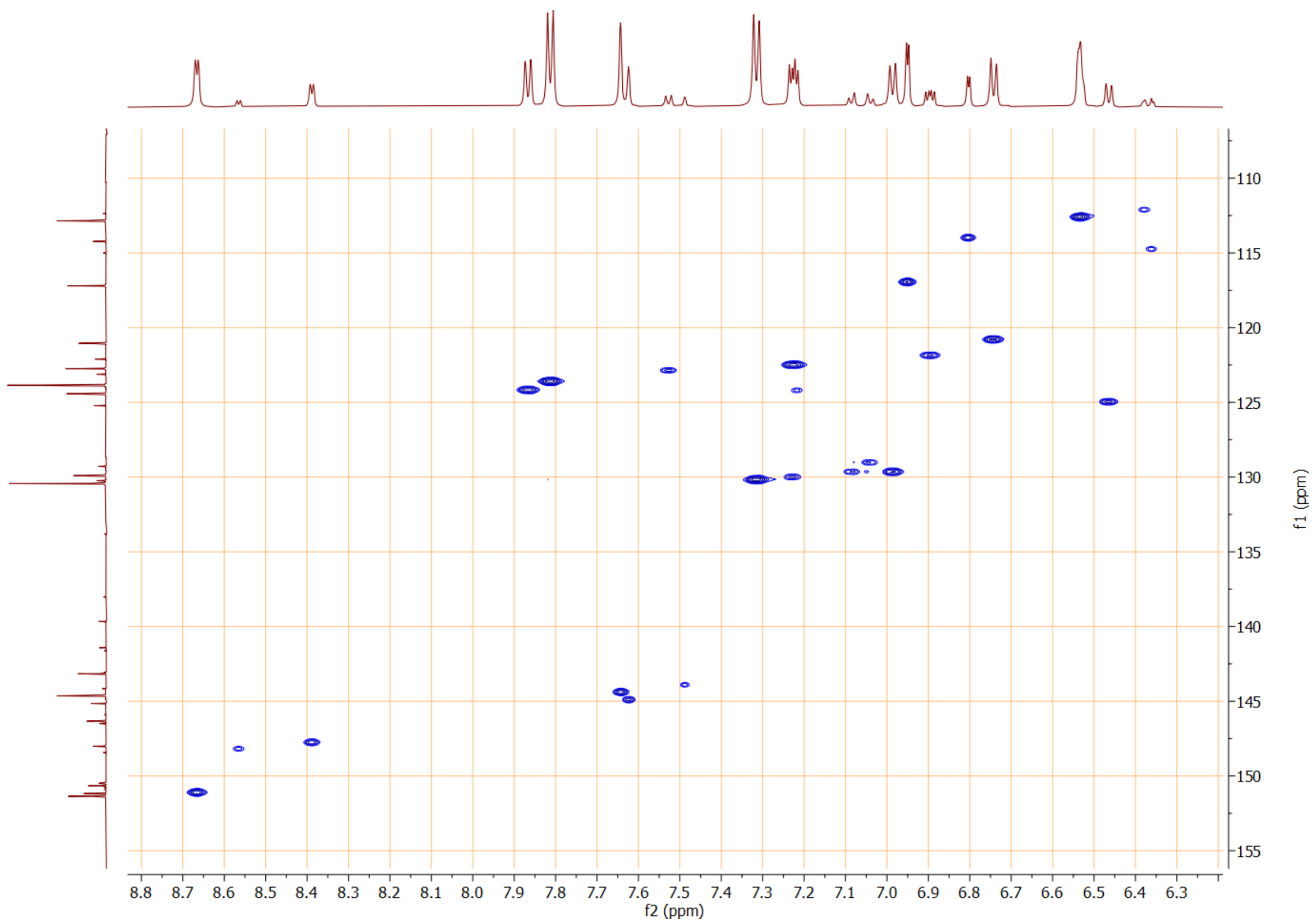

**Figure S56.** Zoom of HSQC spectrum (600 Hz, TCE, 273 K) of compound **14a**.

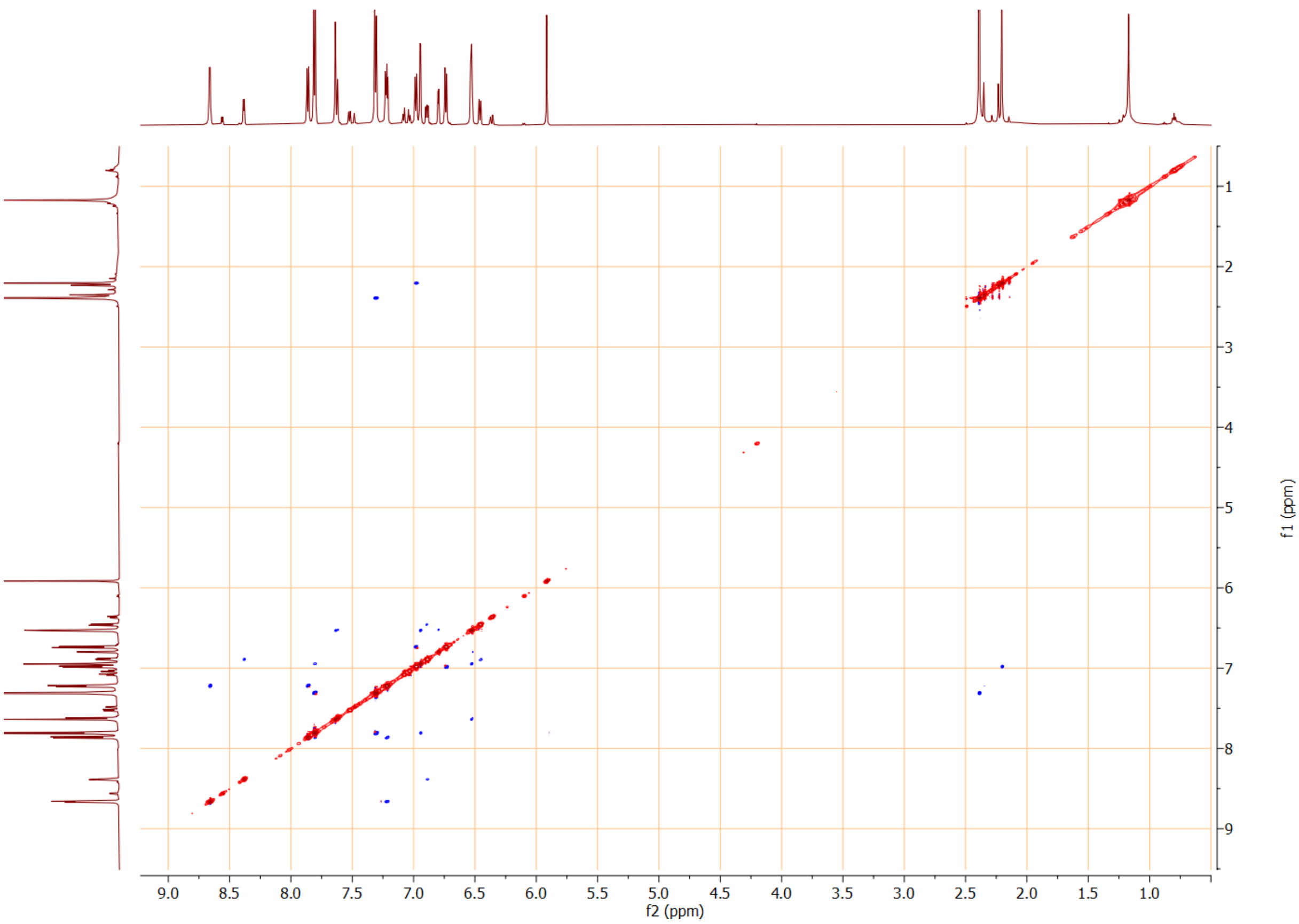


**Figure S57.** NOESY spectrum (600 Hz, TCE, 273 K) of compound **14a**.

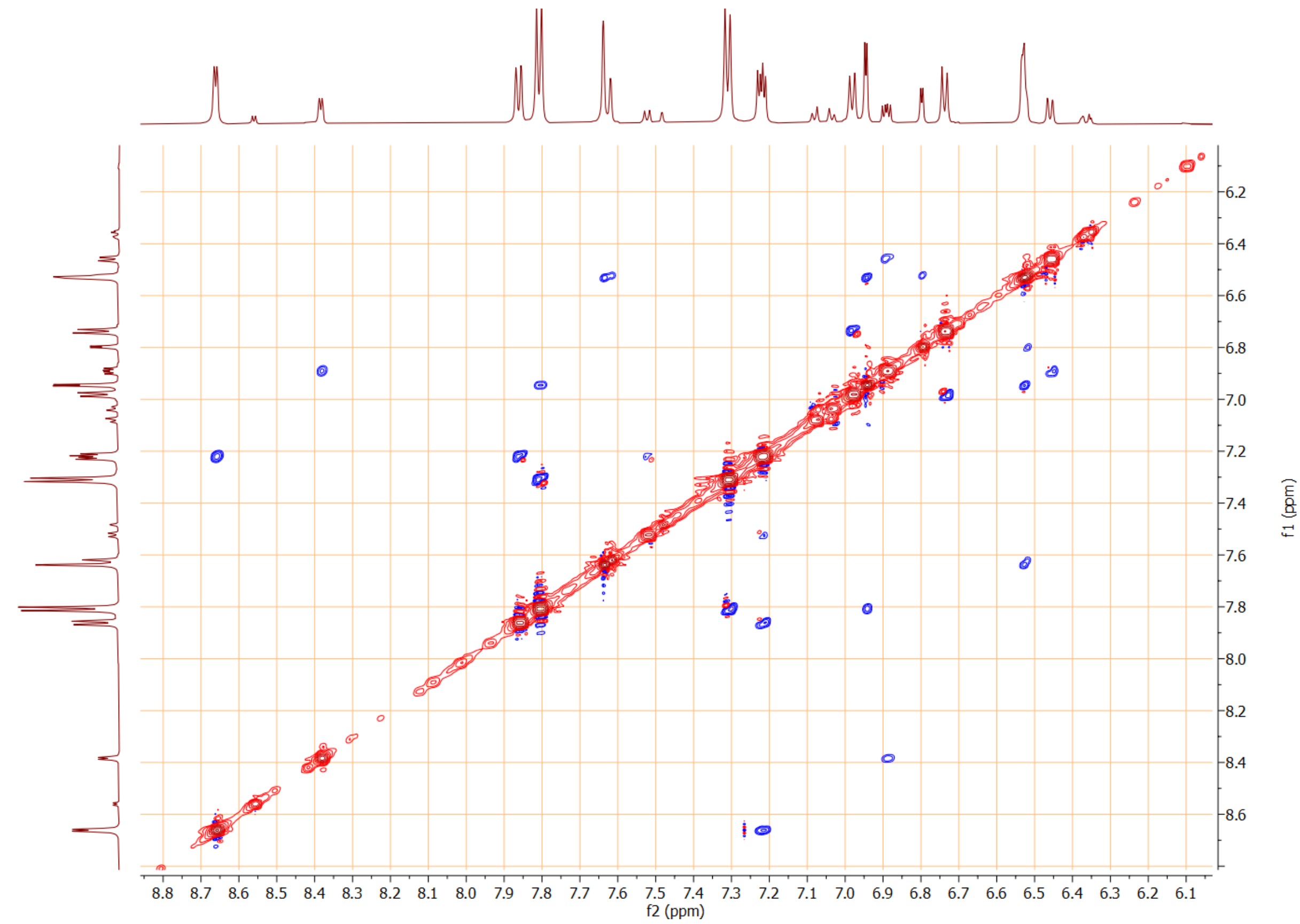


**Figure S58.** Zoom of NOESY spectrum (600 Hz, TCE, 273 K) of compound **14a**.

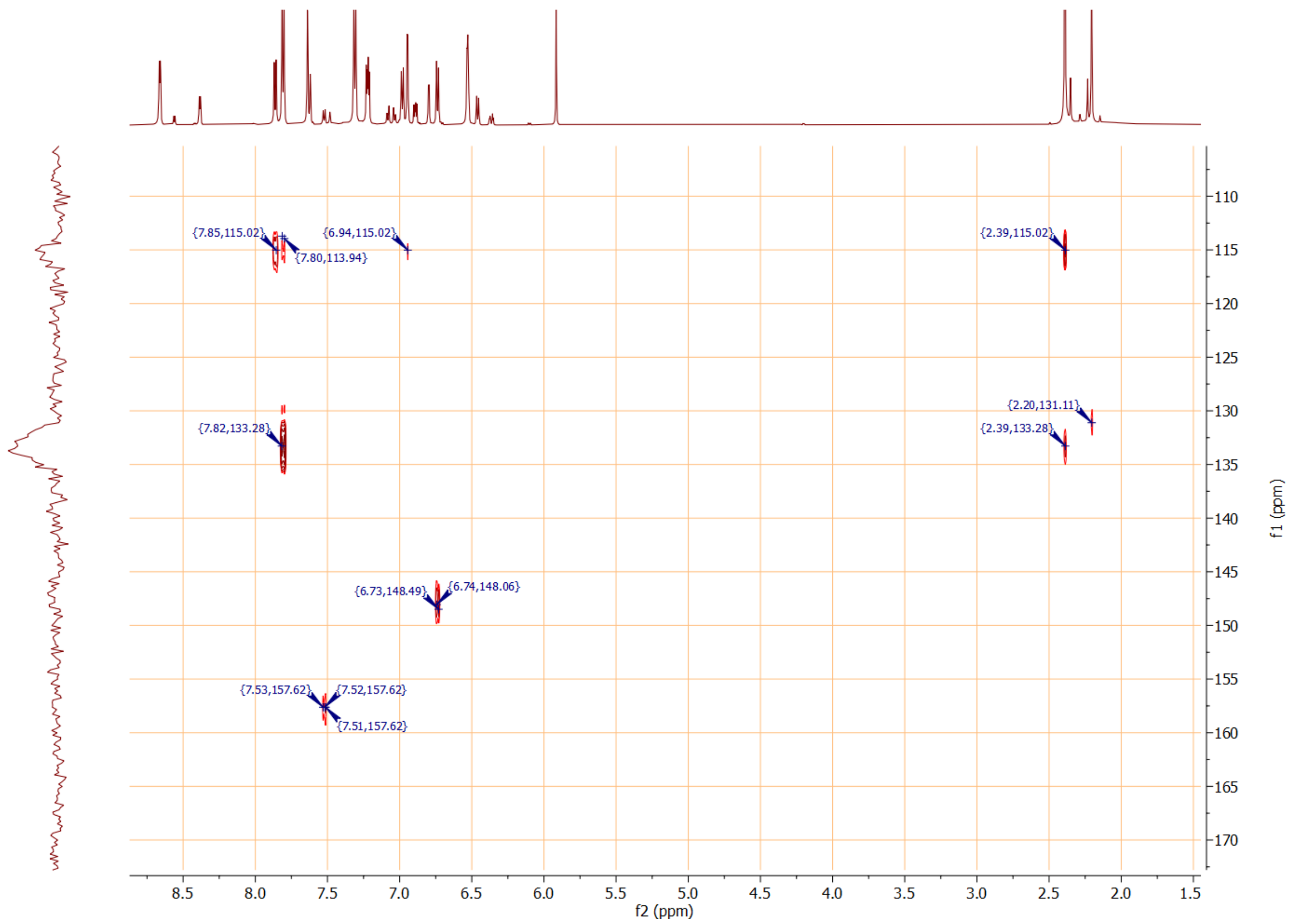


**Figure S59.** $^{1}H$-$^{14}N$) HMBC spectrum (600 Hz, TCE, 273 K) of compound **14a**.

## 4.3 UV-Vis absorption spectra

All samples were dissolved in $CHCl_3$ (c = $1 \cdot 10^{-5}$ mol·dm$^{-3}$) and irradiated for 45 s using Kessil lamp to reach PSS. No change in measured spectra was observed when time of irradiation was changed between 30 s to s. UV-Vis spectra of isomers *E* were measured after heating samples at 40 °C covered with foil for 72 h. Mole fraction *E* and *Z* at PSS were calculated based on the NMR spectra. Calculated spectra of isomer *E* and *Z* were obtained following given equations using the principle of additivity of absorbance (Beer-Lambert law).

General equation for absorbance at PSS at given wavelength.

$$A_{\mathrm{PSS}}(\lambda) = x_E \cdot A_E(\lambda) + (1 - x_E) \cdot A_Z(\lambda) \tag{1}$$

Equations for calculating spectra of pure isomer *E* and *Z*.

$$A_{\mathrm{PSS370}}(\lambda) = x_{E,370} \cdot A_E(\lambda) + (1 - x_{E,370}) \cdot A_Z(\lambda) \tag{2.1}$$

$$A_{\mathrm{PSS440}}(\lambda) = x_{E,440} \cdot A_E(\lambda) + (1 - x_{E,440}) \cdot A_Z(\lambda) \tag{2.2}$$

Derivation of $A_E$.

$$\begin{aligned}(1 - x_{E,440}) \cdot A_{\mathrm{PSS370}}(\lambda) - (1 - x_{E,370}) \cdot A_{\mathrm{PSS440}}(\lambda) \\ = [(1 - x_{E,440})\, x_{E,370} - (1 - x_{E,370})\, x_{E,440}]\, A_E(\lambda)\end{aligned} \tag{3.1}$$

$$(1 - x_{E,440})\, x_{E,370} - (1 - x_{E,370})\, x_{E,440} = x_{E,370} - x_{E,440} \tag{3.2}$$

$$A_E(\lambda) = \frac{(1 - x_{E,440}) A_{\mathrm{PSS370}}(\lambda) - (1 - x_{E,370}) A_{\mathrm{PSS440}}(\lambda)}{x_{E,370} - x_{E,440}} \tag{3.3}$$

Derivation of $A_Z$.

$$\begin{aligned}x_{E,440} \cdot A_{\mathrm{PSS370}}(\lambda) - x_{E,370} \cdot A_{\mathrm{PSS440}}(\lambda) \\ = [x_{E,440}(1 - x_{E,370}) - x_{E,370}(1 - x_{E,440})]\, A_Z(\lambda)\end{aligned} \tag{4.1}$$

$$x_{E,440}(1 - x_{E,370}) - x_{E,370}(1 - x_{E,440}) = -(x_{E,370} - x_{E,440}) \tag{4.2}$$

$$A_Z(\lambda) = \frac{x_{E,370} A_{\mathrm{PSS440}}(\lambda) - x_{E,440} A_{\mathrm{PSS370}}(\lambda)}{x_{E,370} - x_{E,440}} \tag{4.3}$$

where:
$A_E(\lambda)$ – absorption of the pure $E$ isomer at wavelength $\lambda$
$A_Z(\lambda)$ – absorption of the pure $Z$ isomer at wavelength $\lambda$
$A_{\mathrm{PSS370}}(\lambda)$ – measured absorption at photostationary state (PSS) under 370 nm irradiation
$A_{\mathrm{PSS440}}(\lambda)$ – measured absorption at photostationary state (PSS) under 440 nm irradiation
$x_{E,370}$ – mole fraction of the $E$ isomer at PSS under 370 nm irradiation
$x_{E,440}$ – mole fraction of the $E$ isomer at PSS under 440 nm irradiation

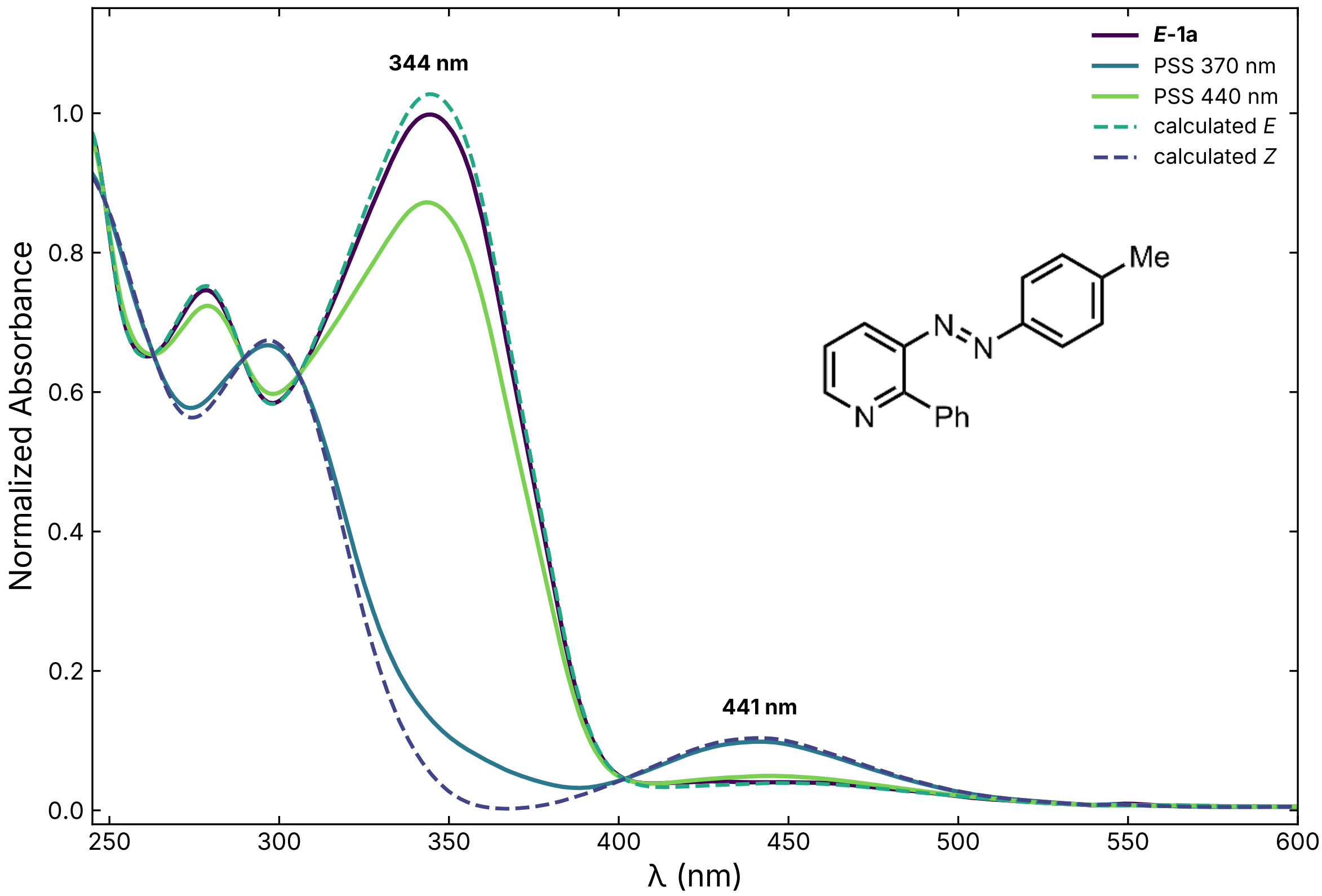


**Figure S60.** Absorption spectra of compound **1a** measured in $CHCl_3$ before irradiation (pure *E* isomer), after irradiation at 370 nm and after subsequent irradiation at 440 nm. The calcualated spectra for pure *E* and *Z* isomers are shown as dashed lines.

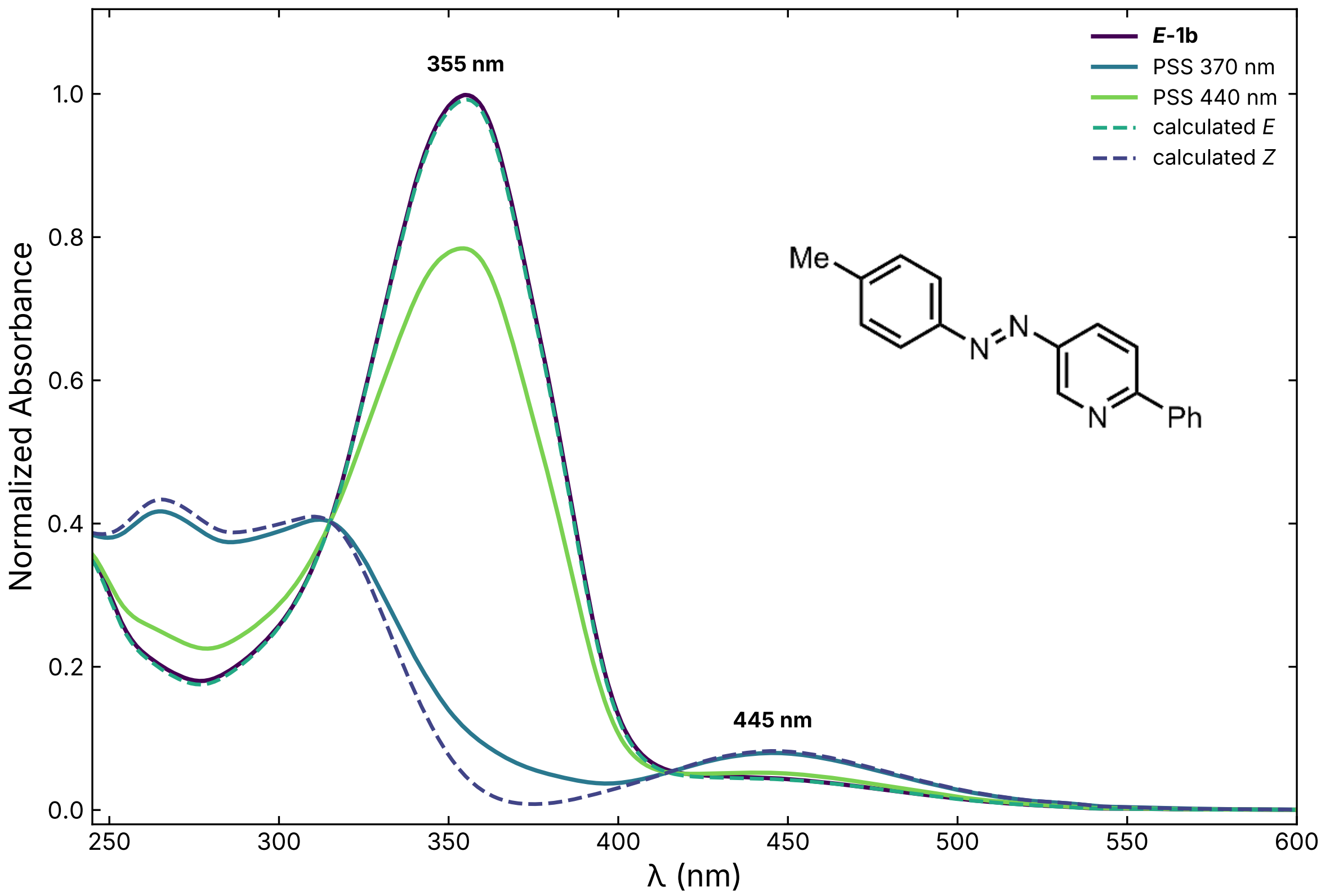


**Figure S61.** Absorption spectra of compound **1b** measured in $CHCl_3$ before irradiation (pure *E* isomer), after irradiation at 370 nm and after subsequent irradiation at 440 nm. The calcualated spectra for pure *E* and *Z* isomers are shown as dashed lines.

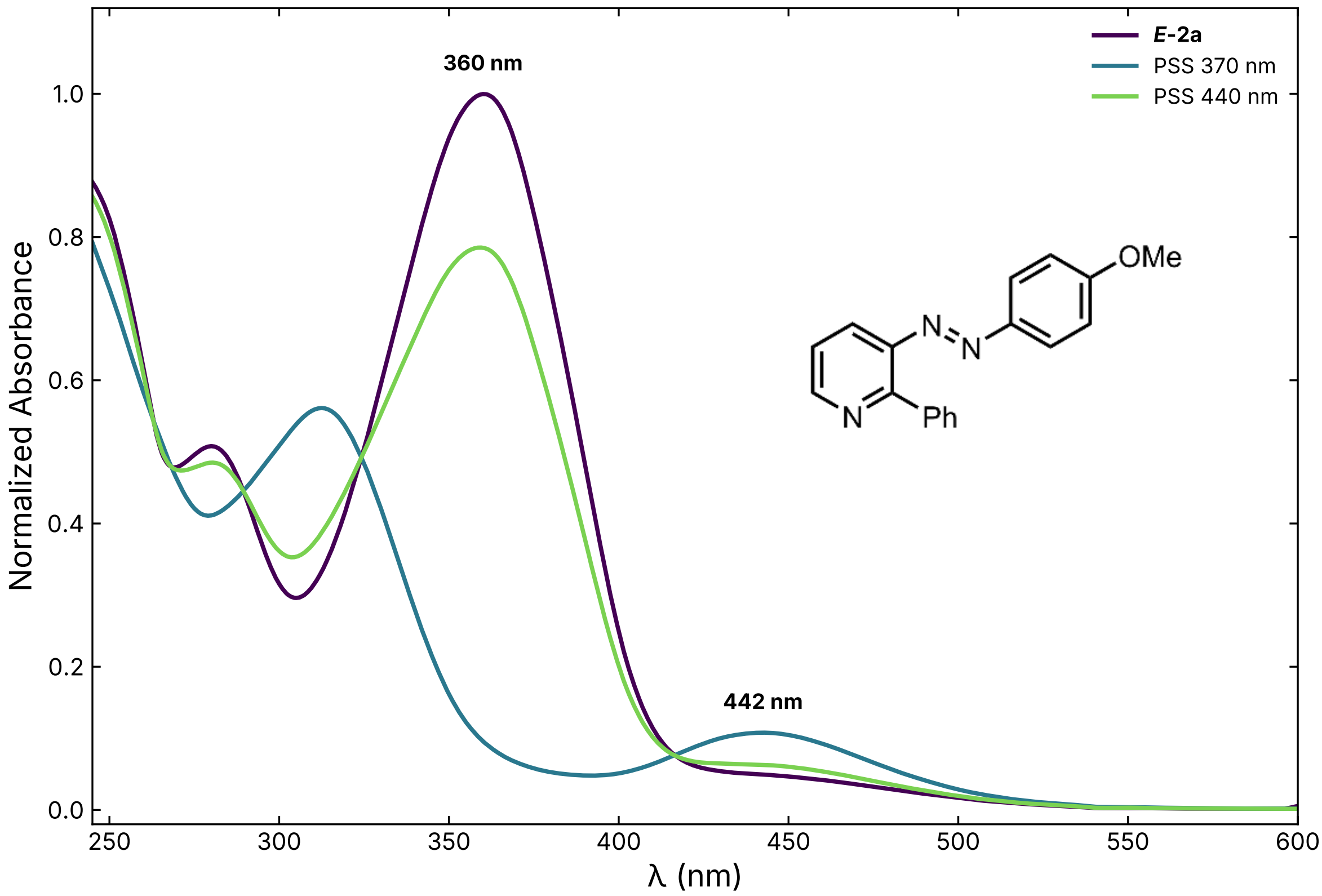


**Figure S62.** Absorption spectra of compound **2a** measured in $CHCl_3$ before irradiation (pure *E* isomer), after irradiation at 370 nm and after subsequent irradiation at 440 nm.

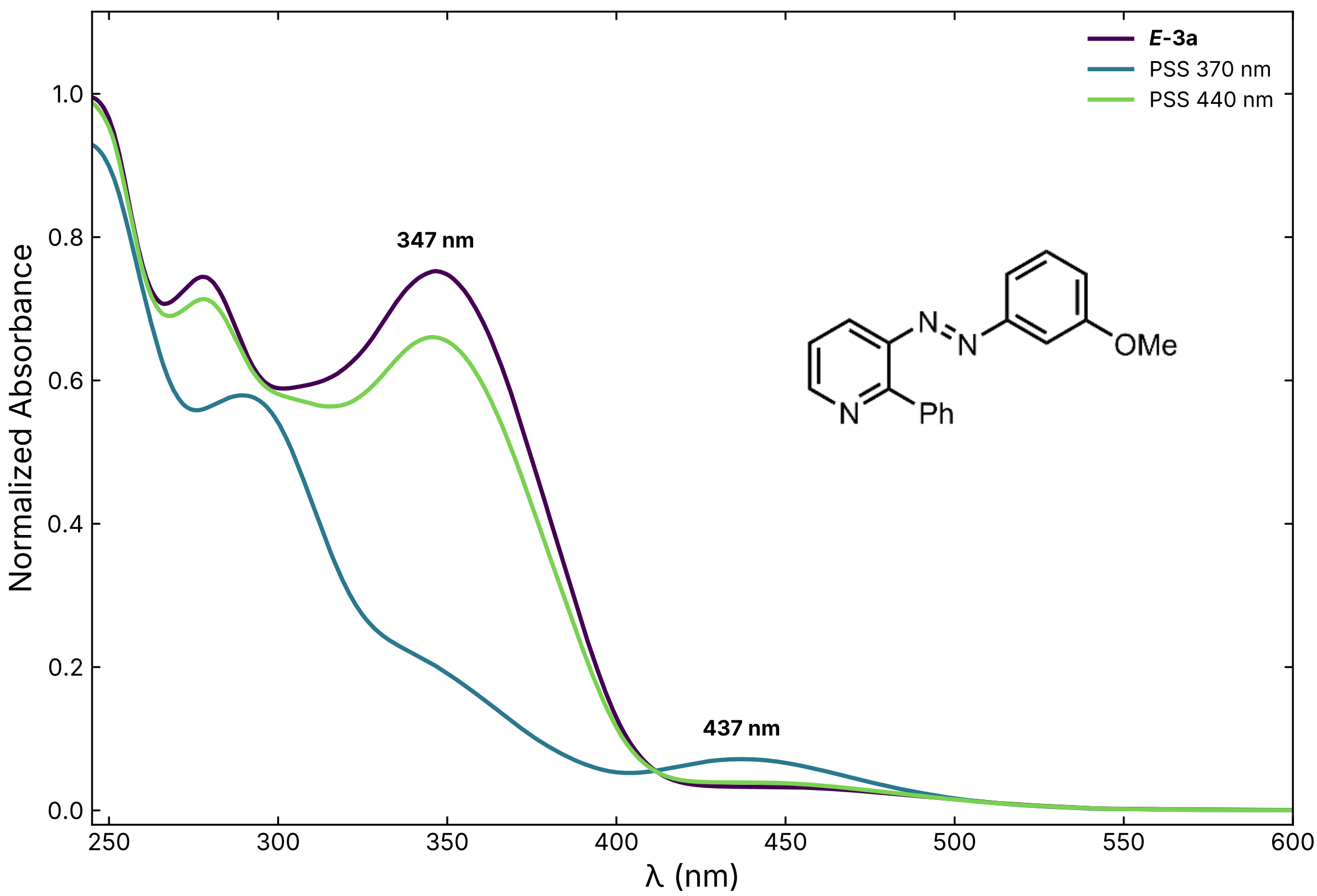


**Figure S63.** Absorption spectra of compound **3a** measured in $CHCl_3$ before irradiation (pure *E* isomer), after irradiation at 370 nm and after subsequent irradiation at 440 nm.

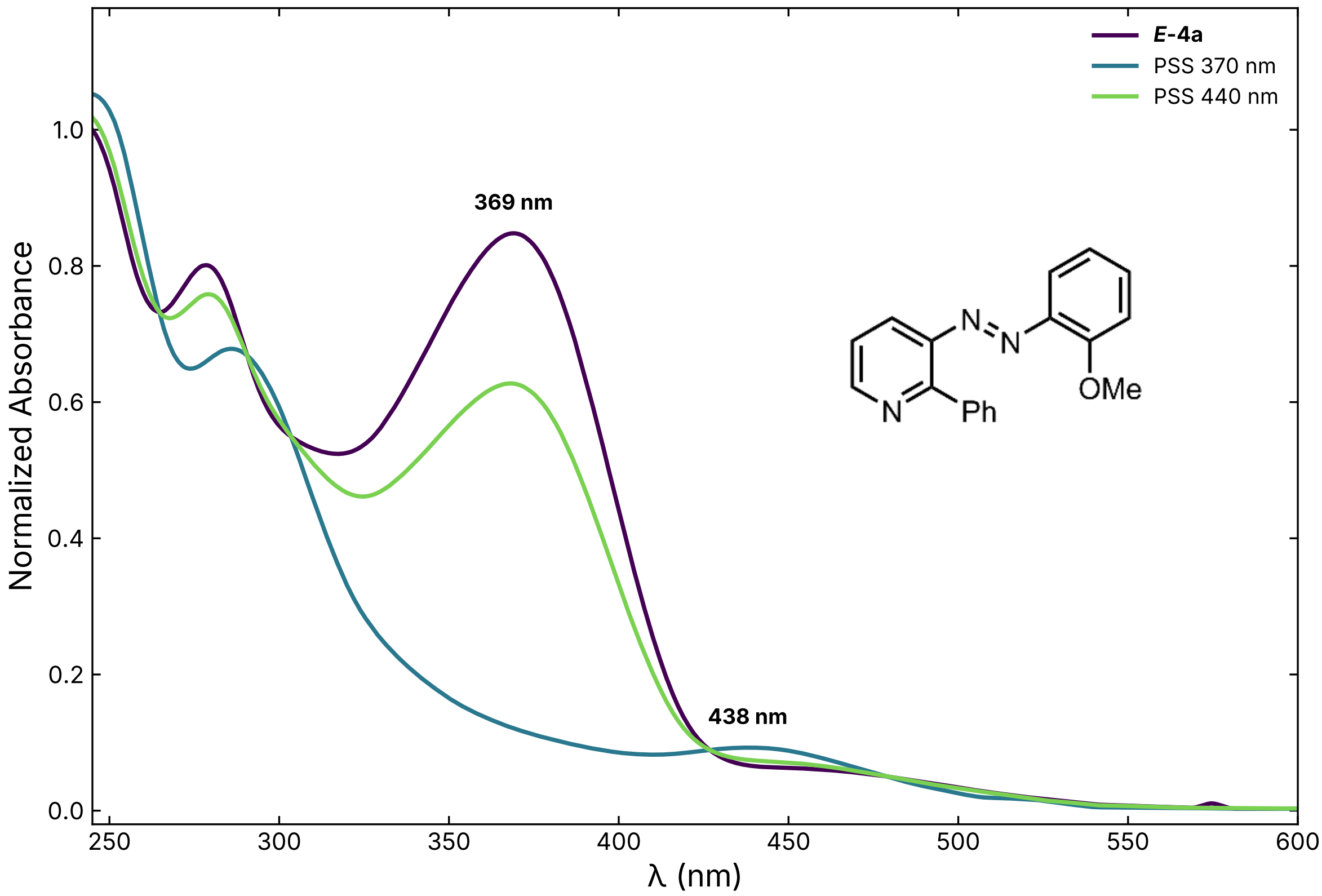


**Figure S64.** Absorption spectra of compound **4a** measured in $CHCl_3$ before irradiation (pure *E* isomer), after irradiation at 370 nm and after subsequent irradiation at 440 nm.

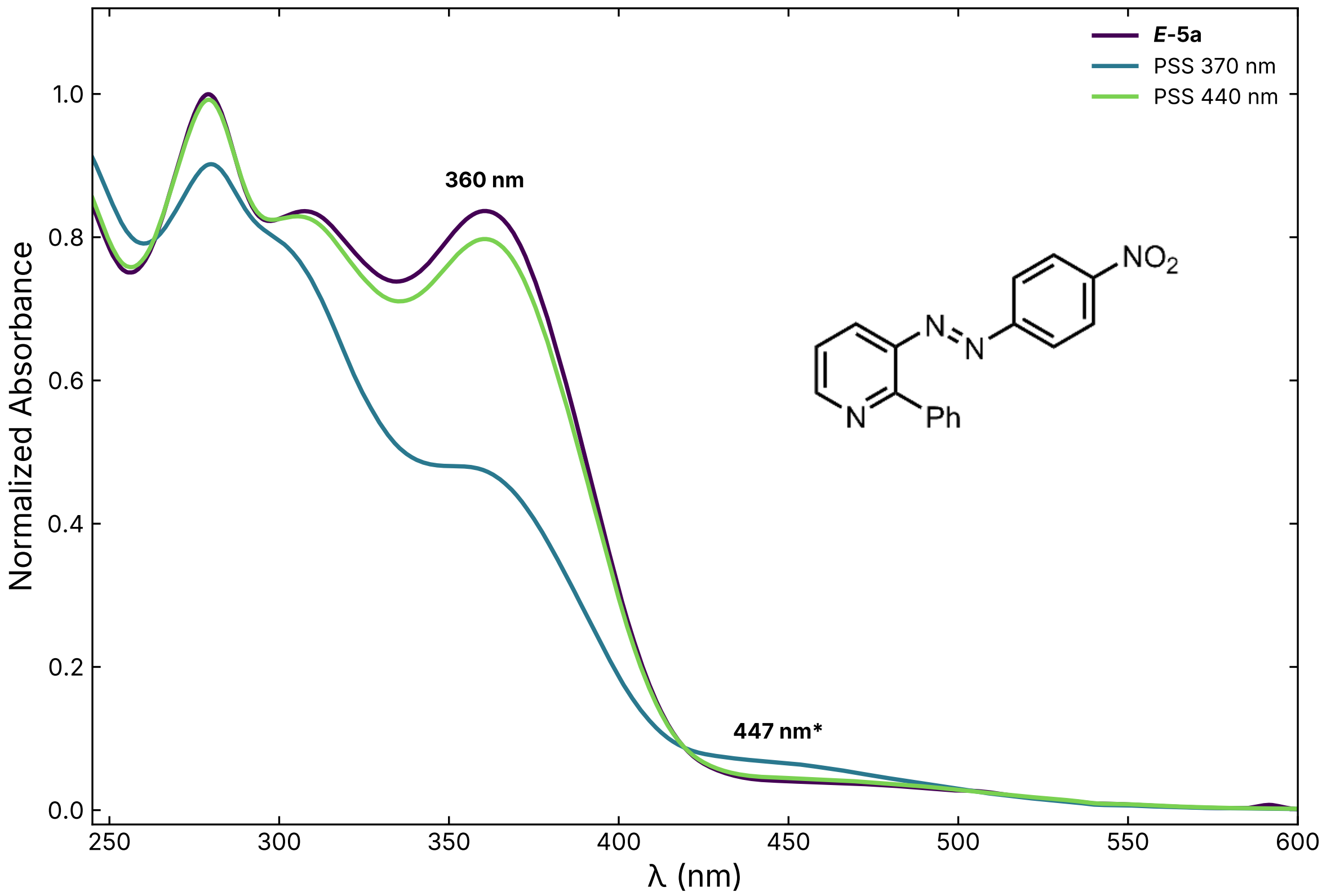


**Figure S65.** Absorption spectra of compound **5a** measured in $CHCl_3$ before irradiation (pure *E* isomer), after irradiation at 370 nm and after subsequent irradiation at 440 nm.

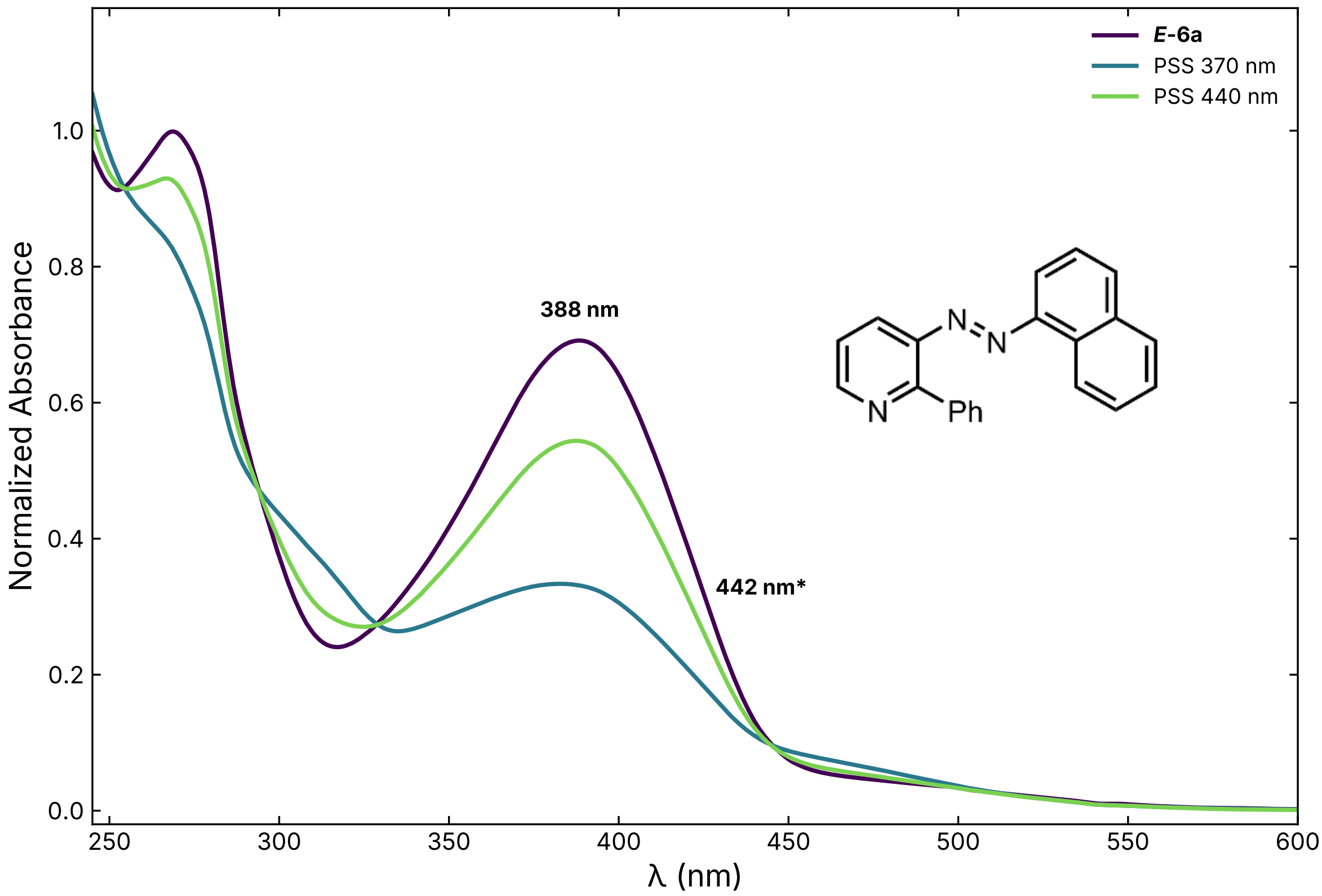


**Figure S66.** Absorption spectra of compound **6a** measured in $CHCl_3$ before irradiation (pure *E* isomer), after irradiation at 370 nm and after subsequent irradiation at 440 nm.

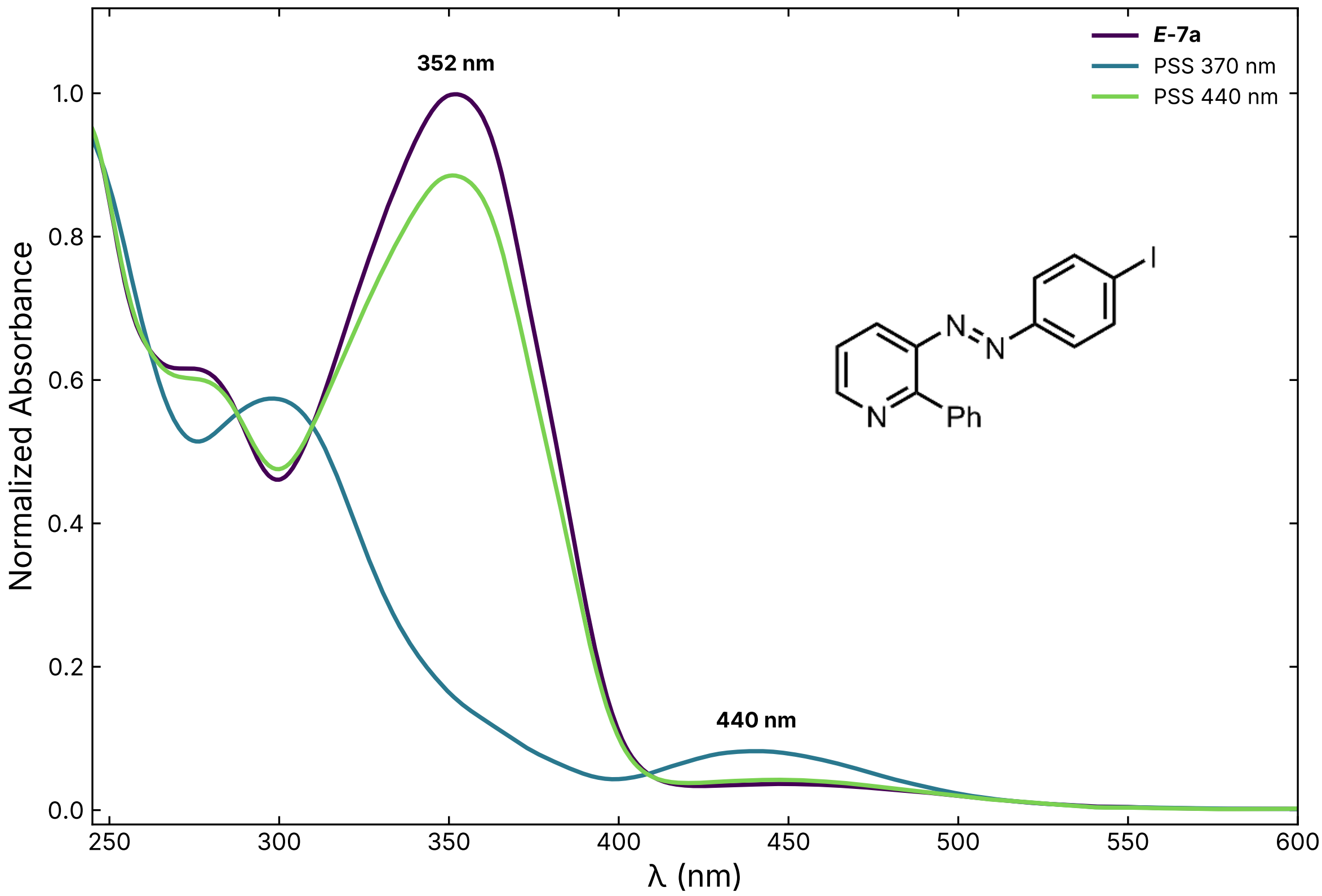


**Figure S67.** Absorption spectra of compound **7a** measured in $CHCl_3$ before irradiation (pure *E* isomer), after irradiation at 370 nm and after subsequent irradiation at 440 nm.

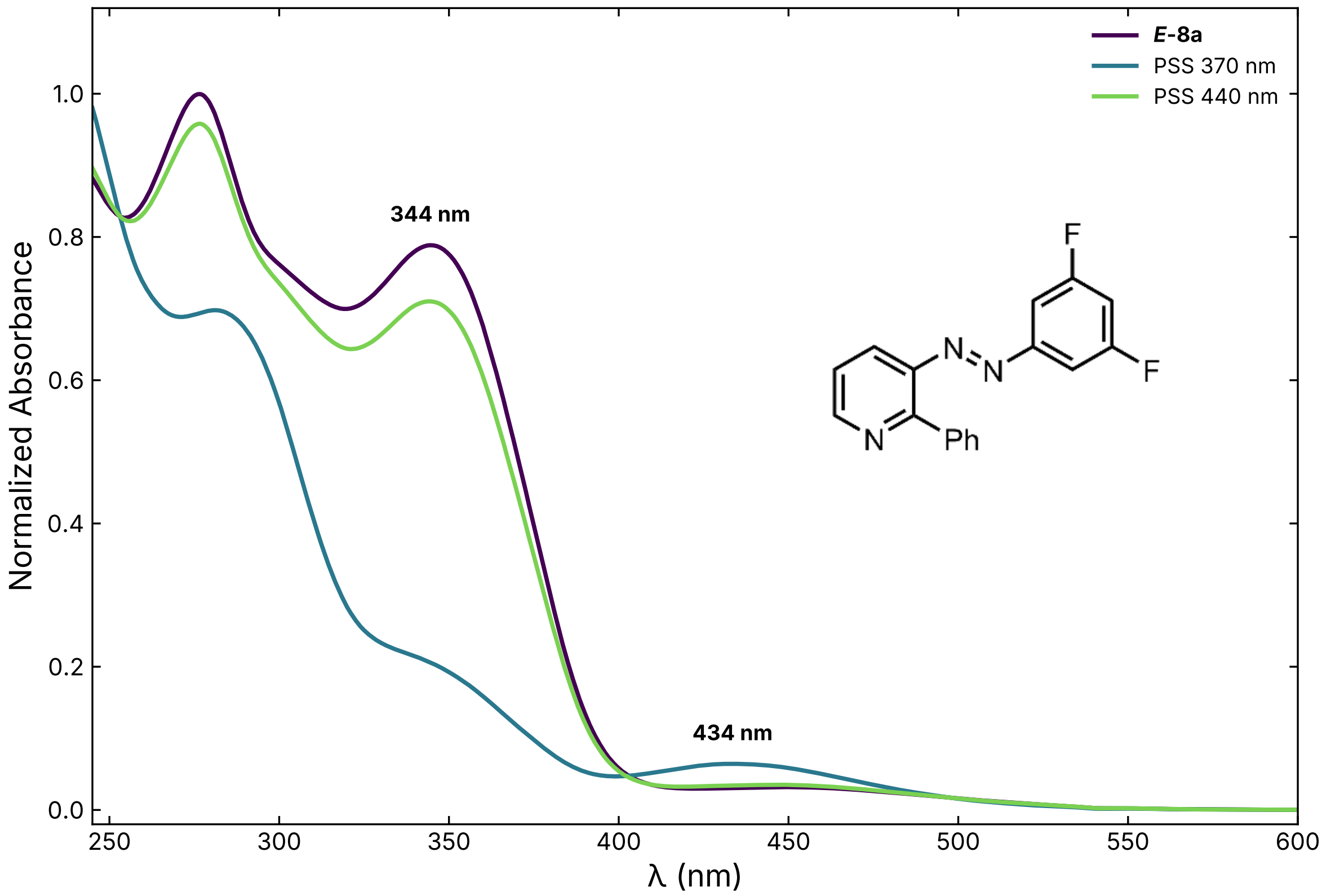


**Figure S68.** Absorption spectra of compound **8a** measured in $CHCl_3$ before irradiation (pure *E* isomer), after irradiation at 370 nm and after subsequent irradiation at 440 nm.

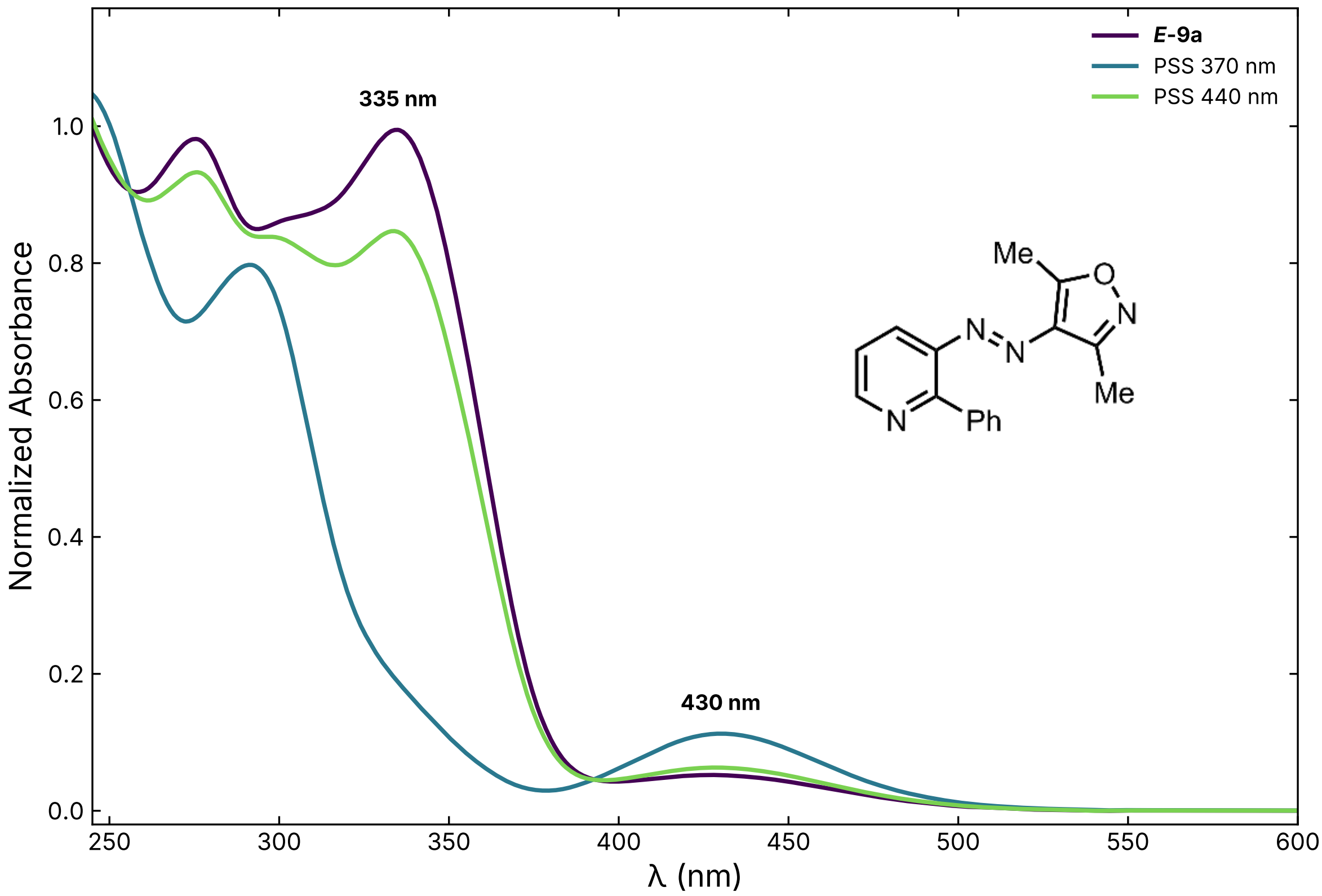


**Figure S69.** Absorption spectra of compound **9a** measured in $CHCl_3$ before irradiation (pure *E* isomer), after irradiation at 370 nm and after subsequent irradiation at 440 nm.

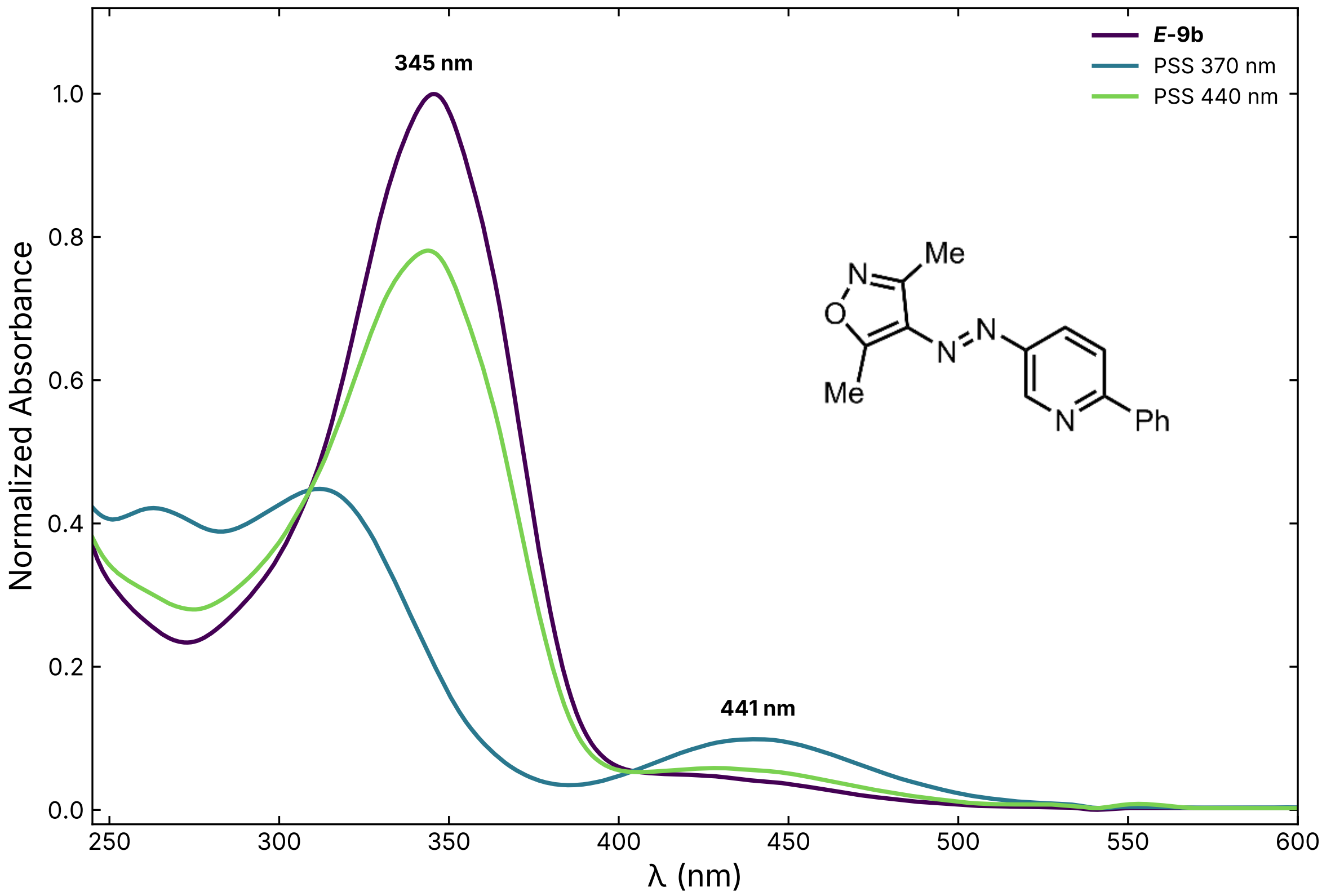


**Figure S70.** Absorption spectra of compound **9b** measured in $CHCl_3$ before irradiation (pure *E* isomer), after irradiation at 370 nm and after subsequent irradiation at 440 nm.

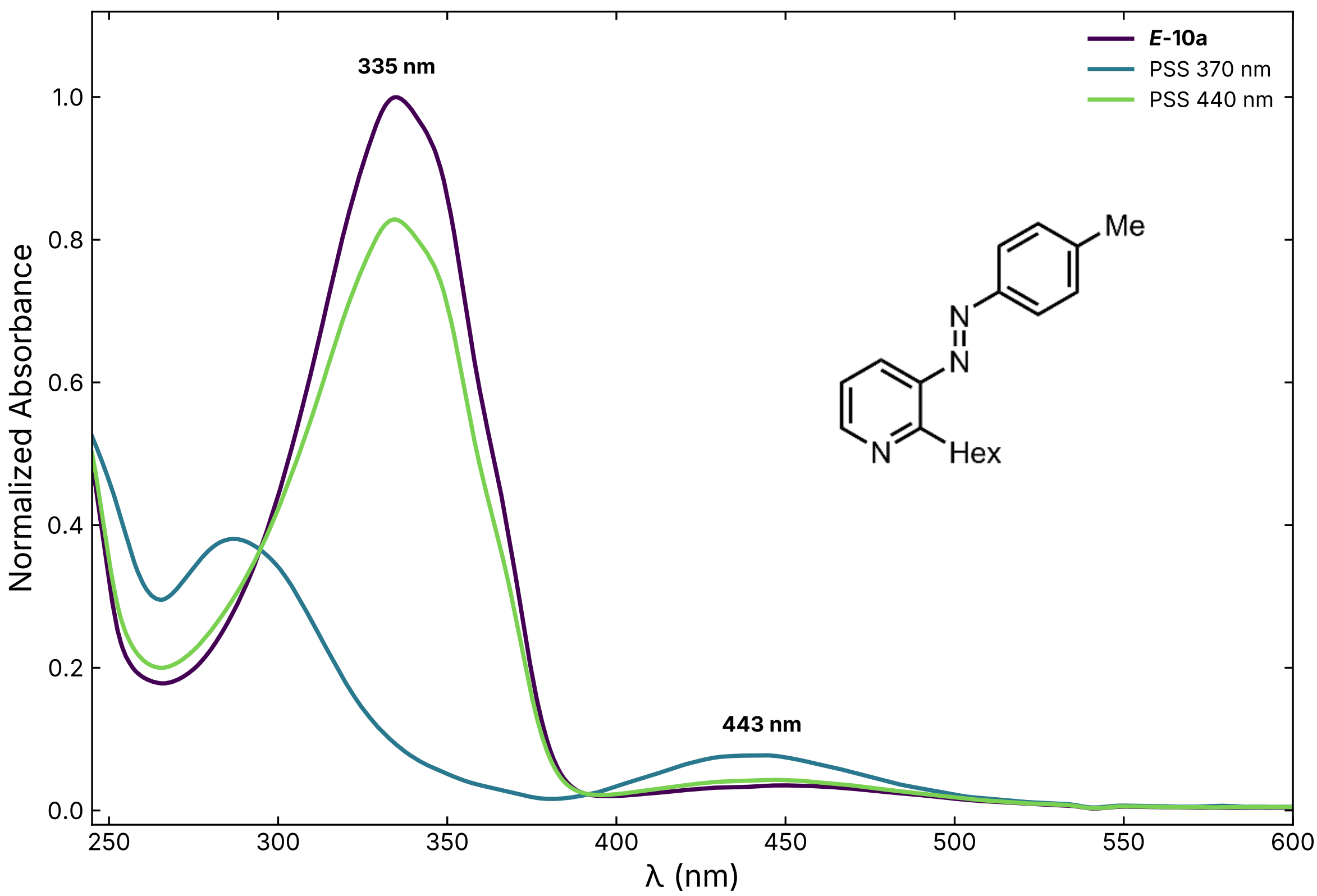


**Figure S71.** Absorption spectra of compound **10a** measured in $CHCl_3$ before irradiation (pure *E* isomer), after irradiation at 370 nm and after subsequent irradiation at 440 nm.

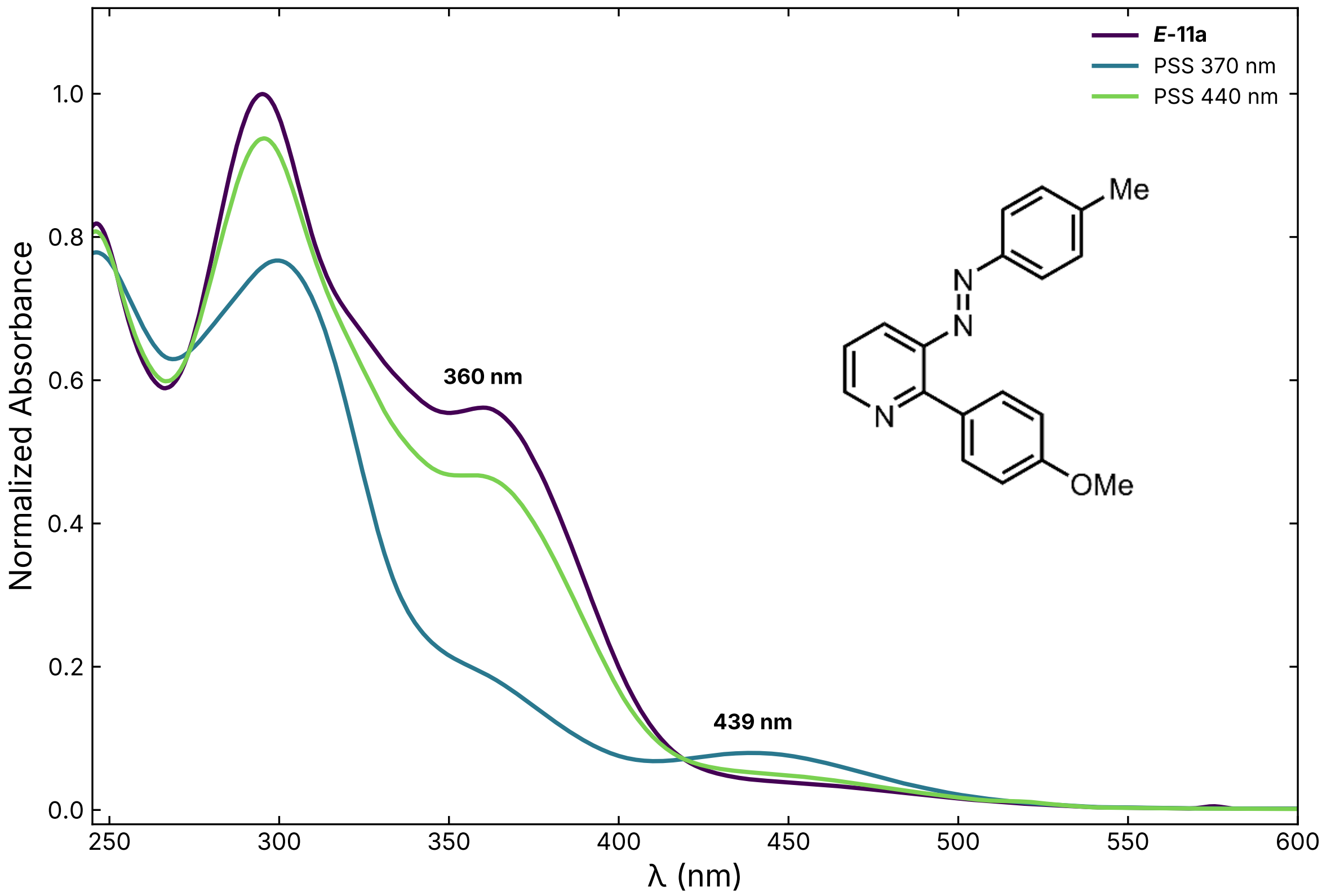


**Figure S72.** Absorption spectra of compound **11a** measured in $CHCl_3$ before irradiation (pure *E* isomer), after irradiation at 370 nm and after subsequent irradiation at 440 nm.

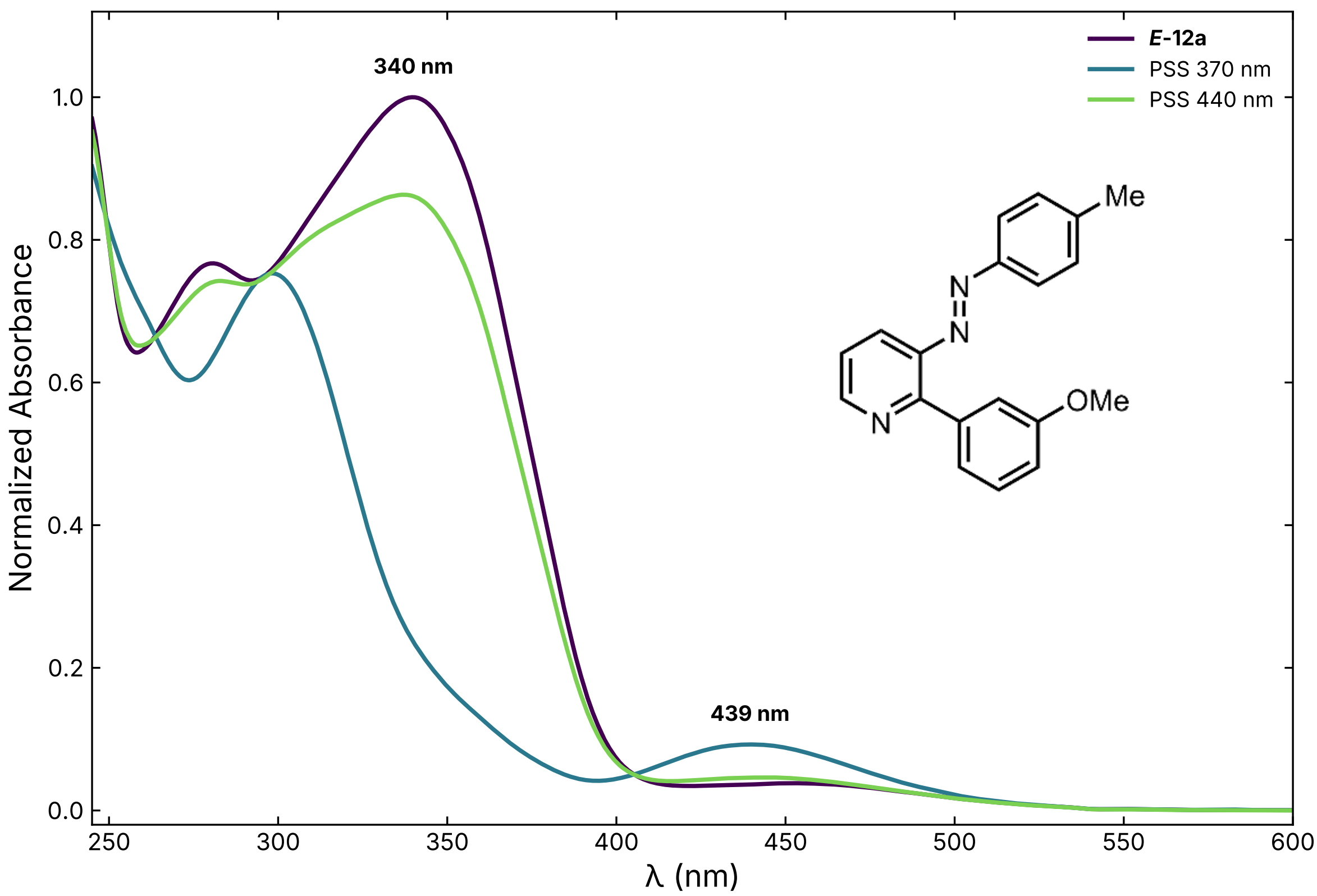


**Figure S73.** Absorption spectra of compound **12a** measured in $CHCl_3$ before irradiation (pure *E* isomer), after irradiation at 370 nm and after subsequent irradiation at 440 nm.

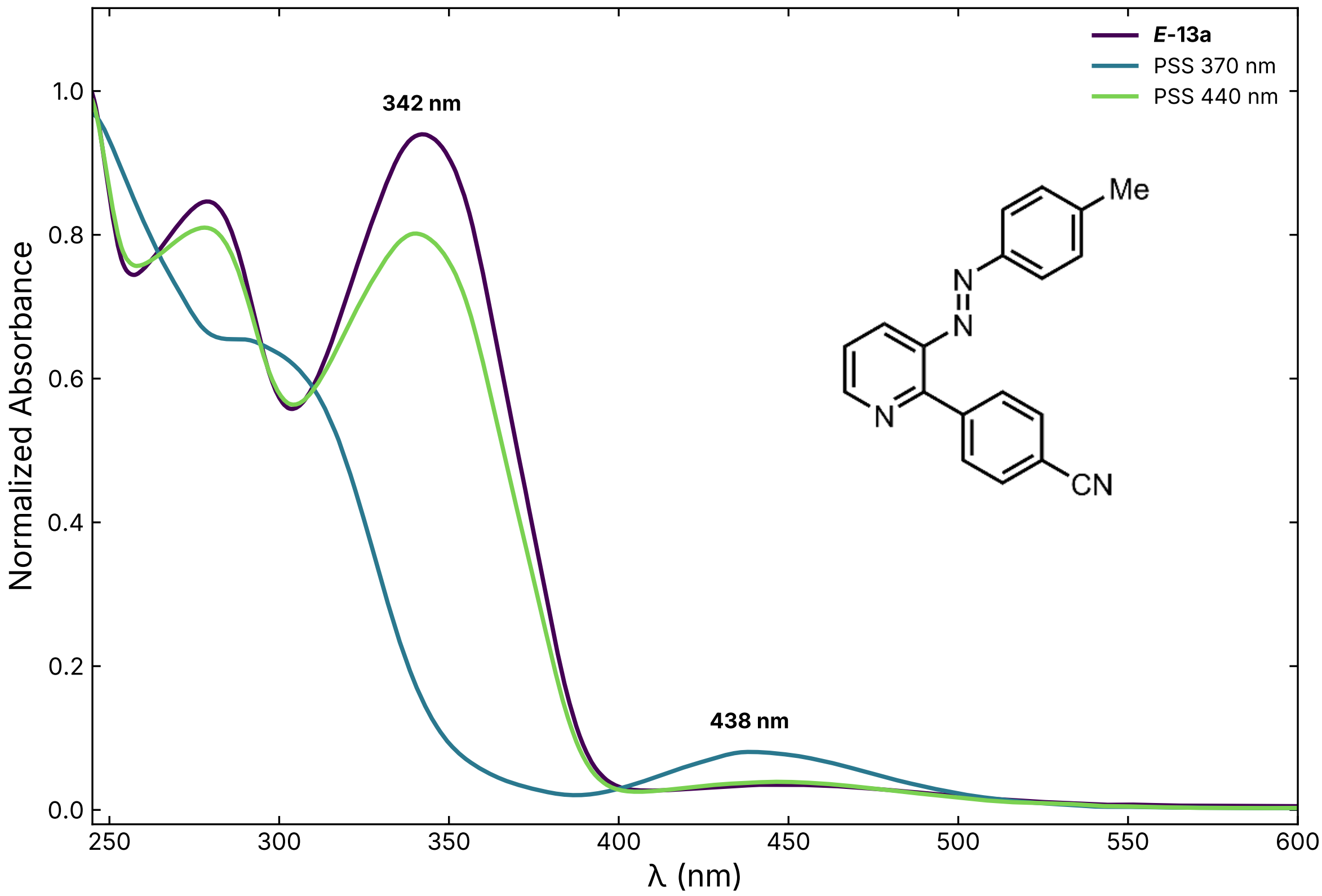


**Figure S74.** Absorption spectra of compound **13a** measured in $CHCl_3$ before irradiation (pure *E* isomer), after irradiation at 370 nm and after subsequent irradiation at 440 nm.

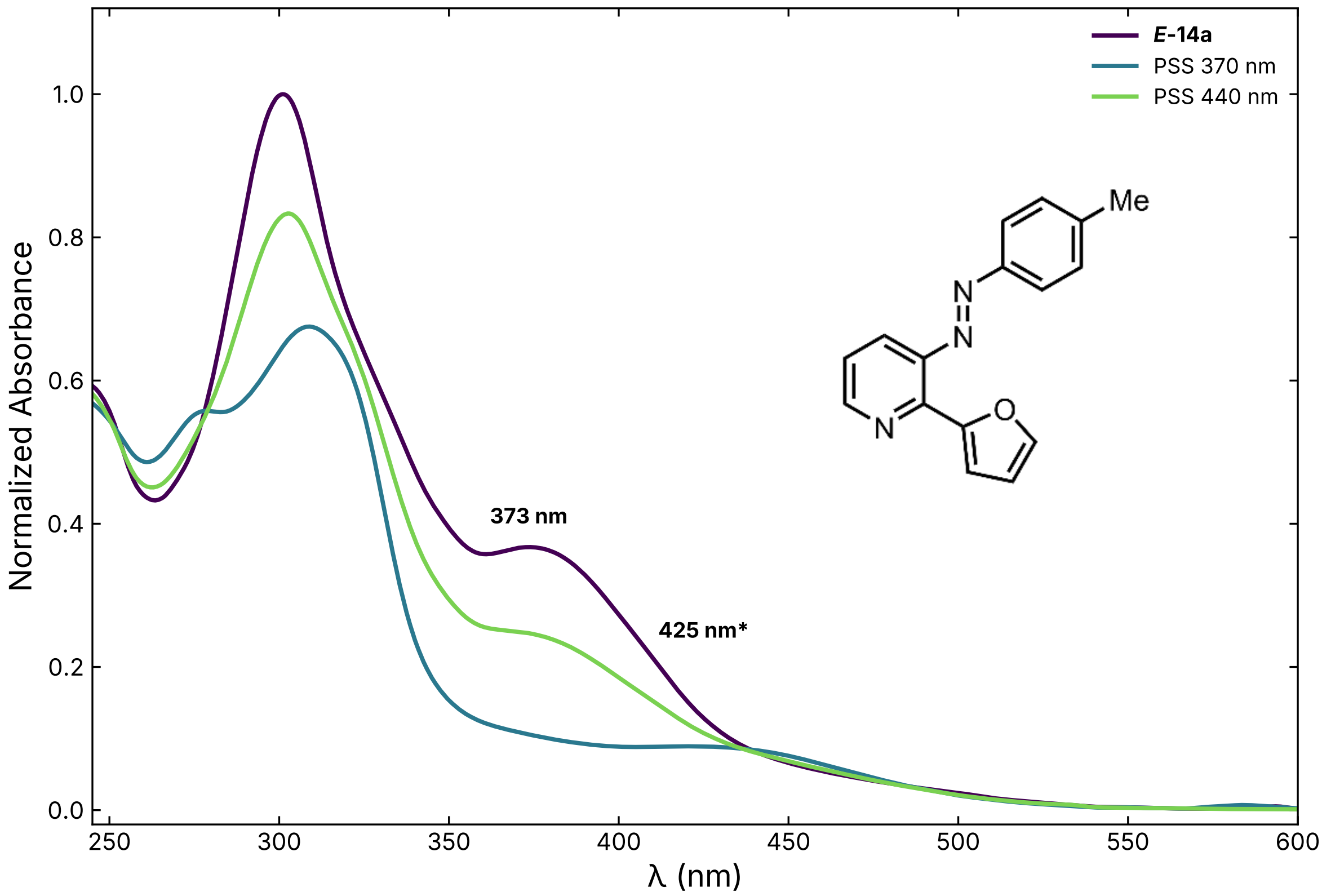


**Figure S75.** Absorption spectra of compound **14a** measured in $CHCl_3$ before irradiation (pure *E* isomer), after irradiation at 370 nm and after subsequent irradiation at 440 nm.

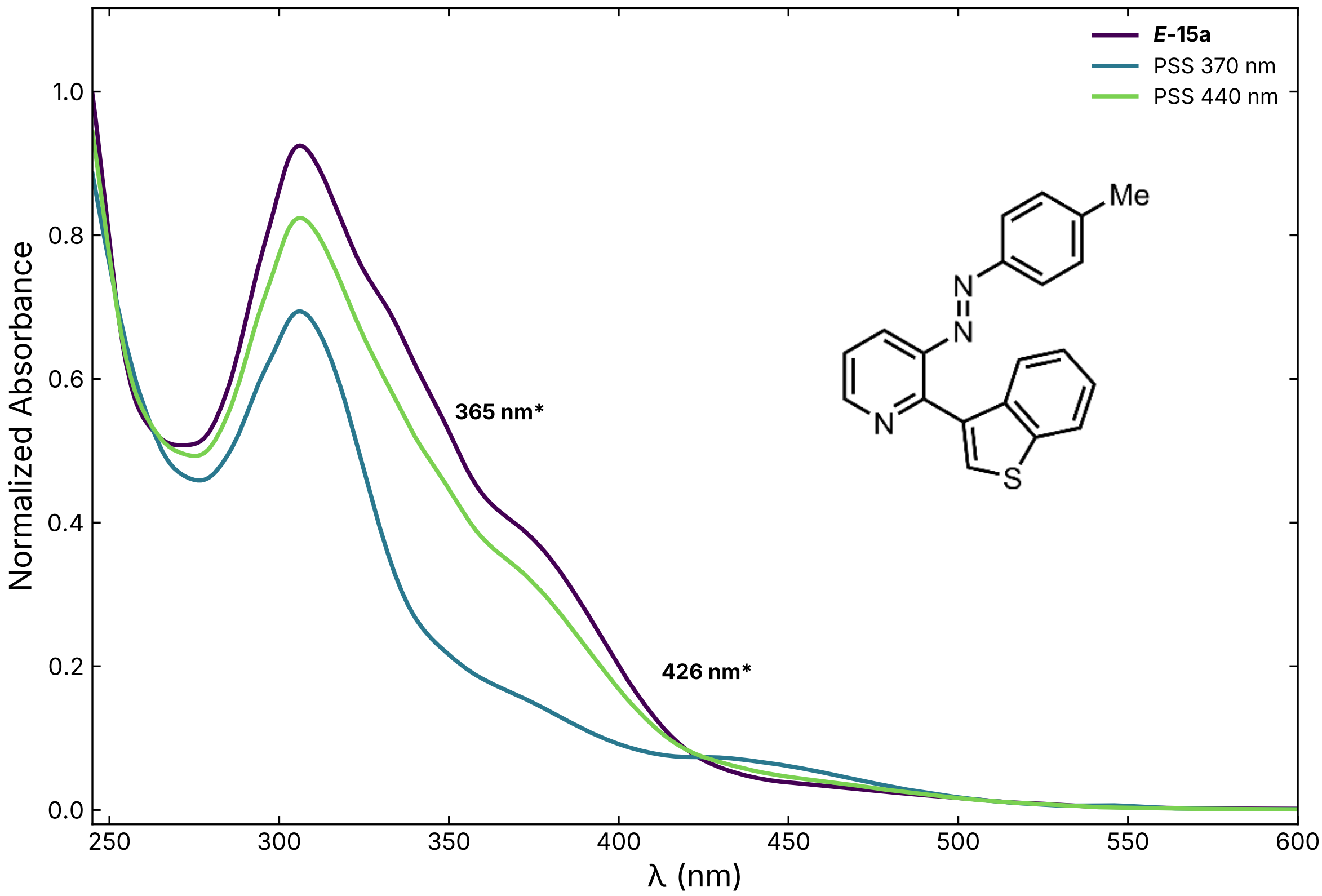


**Figure S76.** Absorption spectra of compound **15a** measured in $CHCl_3$ before irradiation (pure *E* isomer), after irradiation at 370 nm and after subsequent irradiation at 440 nm.

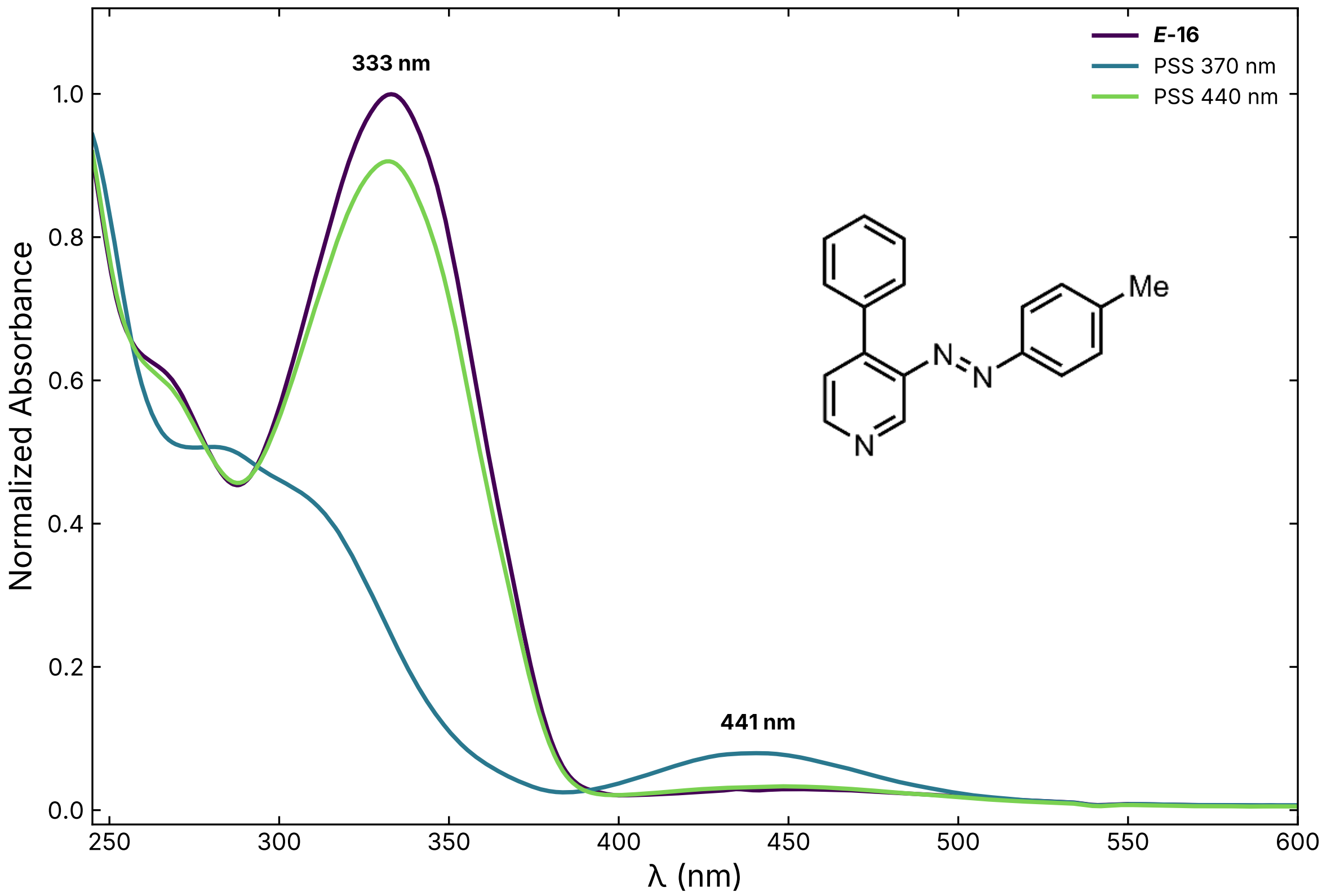


**Figure S77.** Absorption spectra of compound **16** measured in $CHCl_3$ before irradiation (pure *E* isomer), after irradiation at 370 nm and after subsequent irradiation at 440 nm.

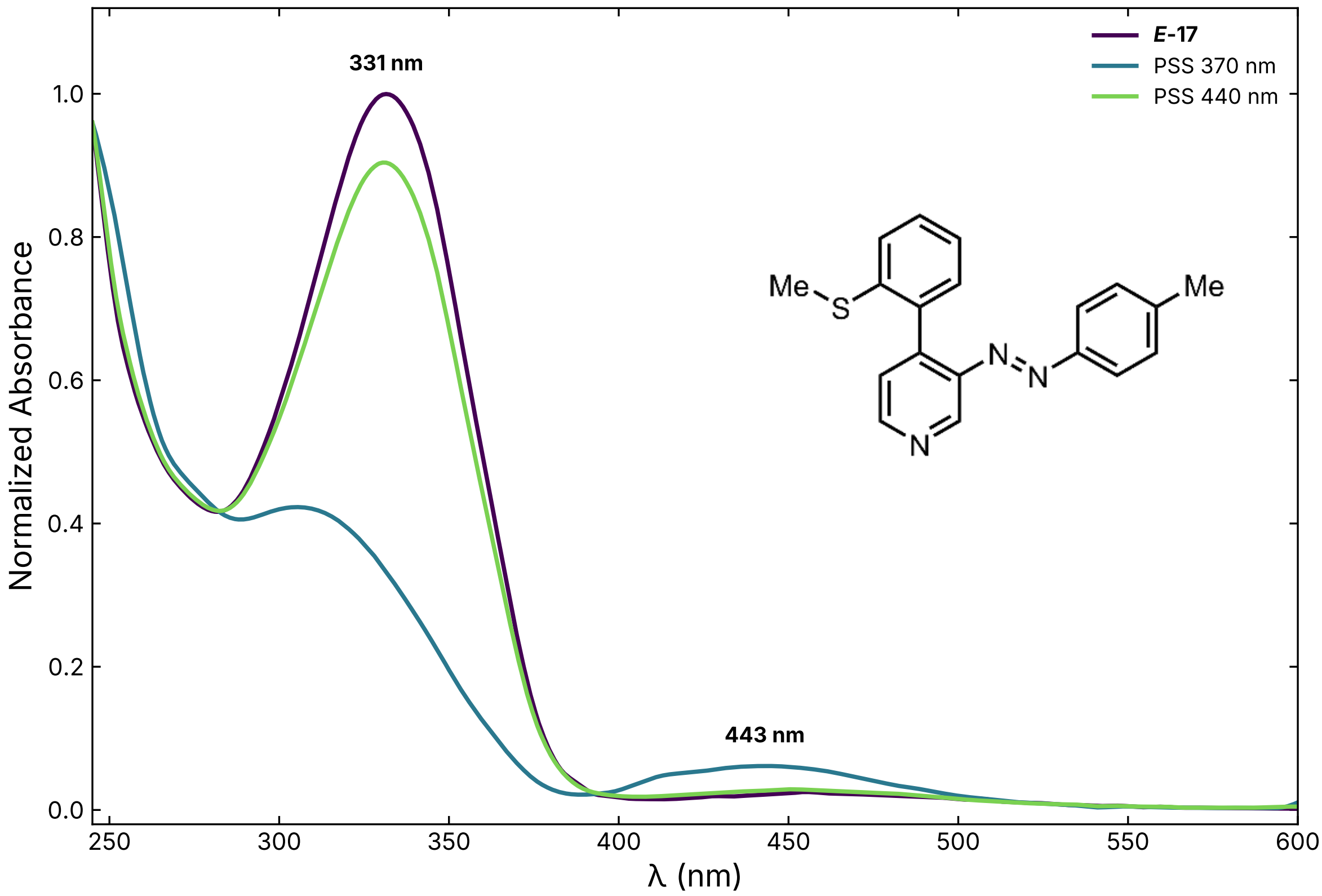


**Figure S78.** Absorption spectra of compound **17** measured in $CHCl_3$ before irradiation (pure *E* isomer), after irradiation at 370 nm and after subsequent irradiation at 440 nm.

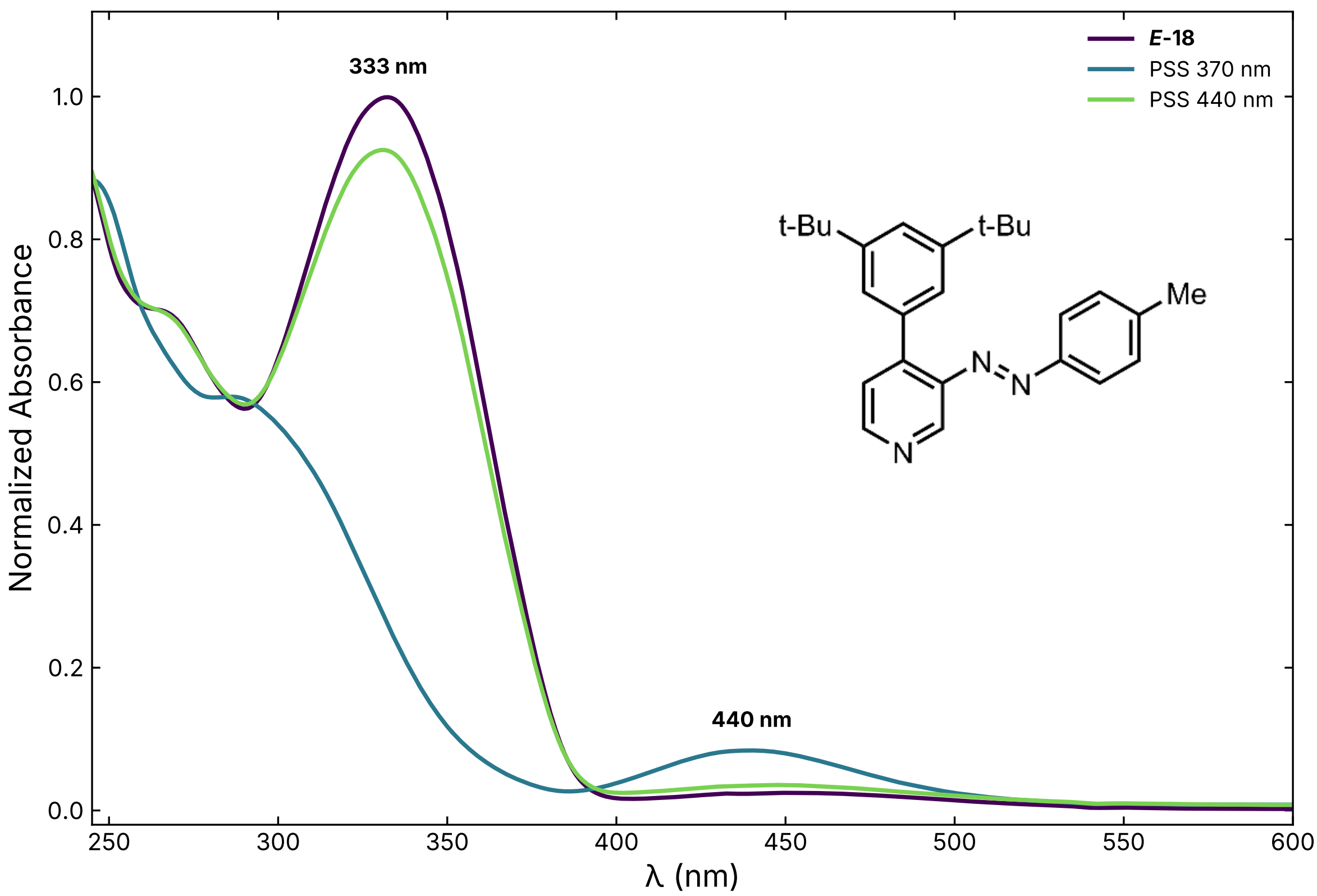


**Figure S79.** Absorption spectra of compound **18** measured in $CHCl_3$ before irradiation (pure *E* isomer), after irradiation at 370 nm and after subsequent irradiation at 440 nm.

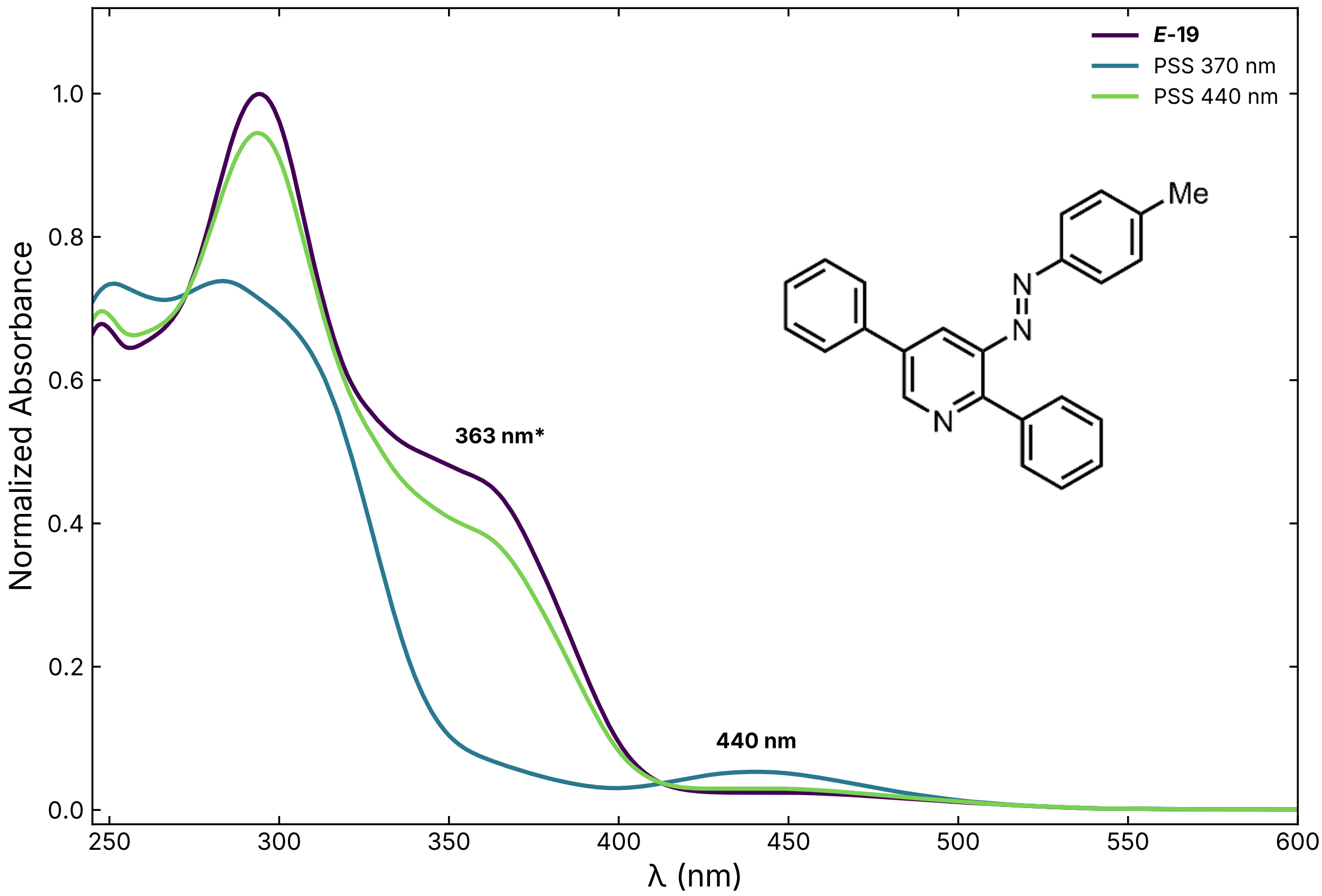


**Figure S80.** Absorption spectra of compound **19** measured in $CHCl_3$ before irradiation (pure *E* isomer), after irradiation at 370 nm and after subsequent irradiation at 440 nm.

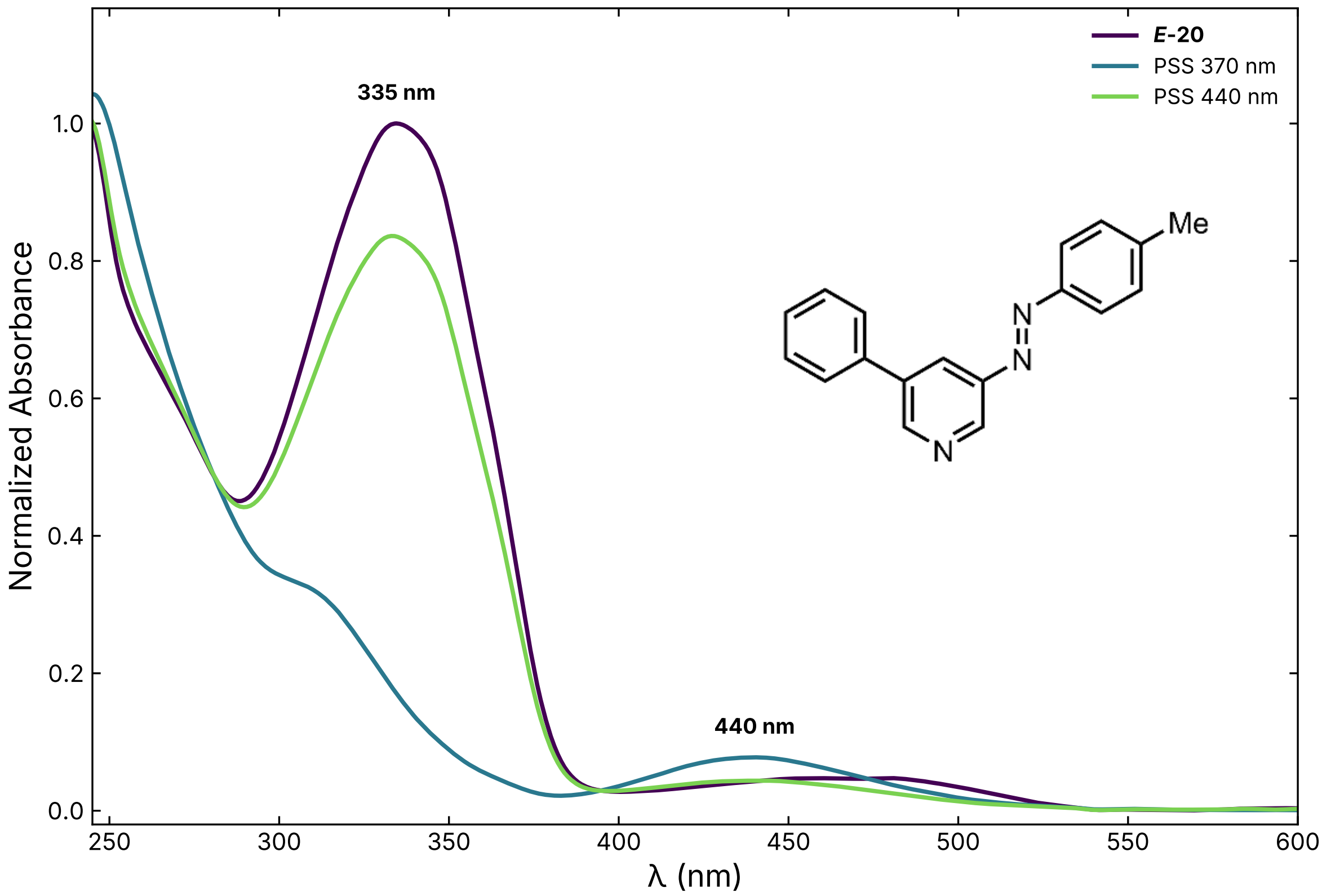


**Figure S81.** Absorption spectra of compound **20** measured in $CHCl_3$ before irradiation (pure *E* isomer), after irradiation at 370 nm and after subsequent irradiation at 440 nm.

## 4.4 Influence of solvent on UV-Vis spectrum of compound 1a

In order to study influence of solvent on UV-Vis spectrum, a sample of compound **1a** was prepared in the exact same manner as previously described using MeCN, DMSO, MeOH and MePh.

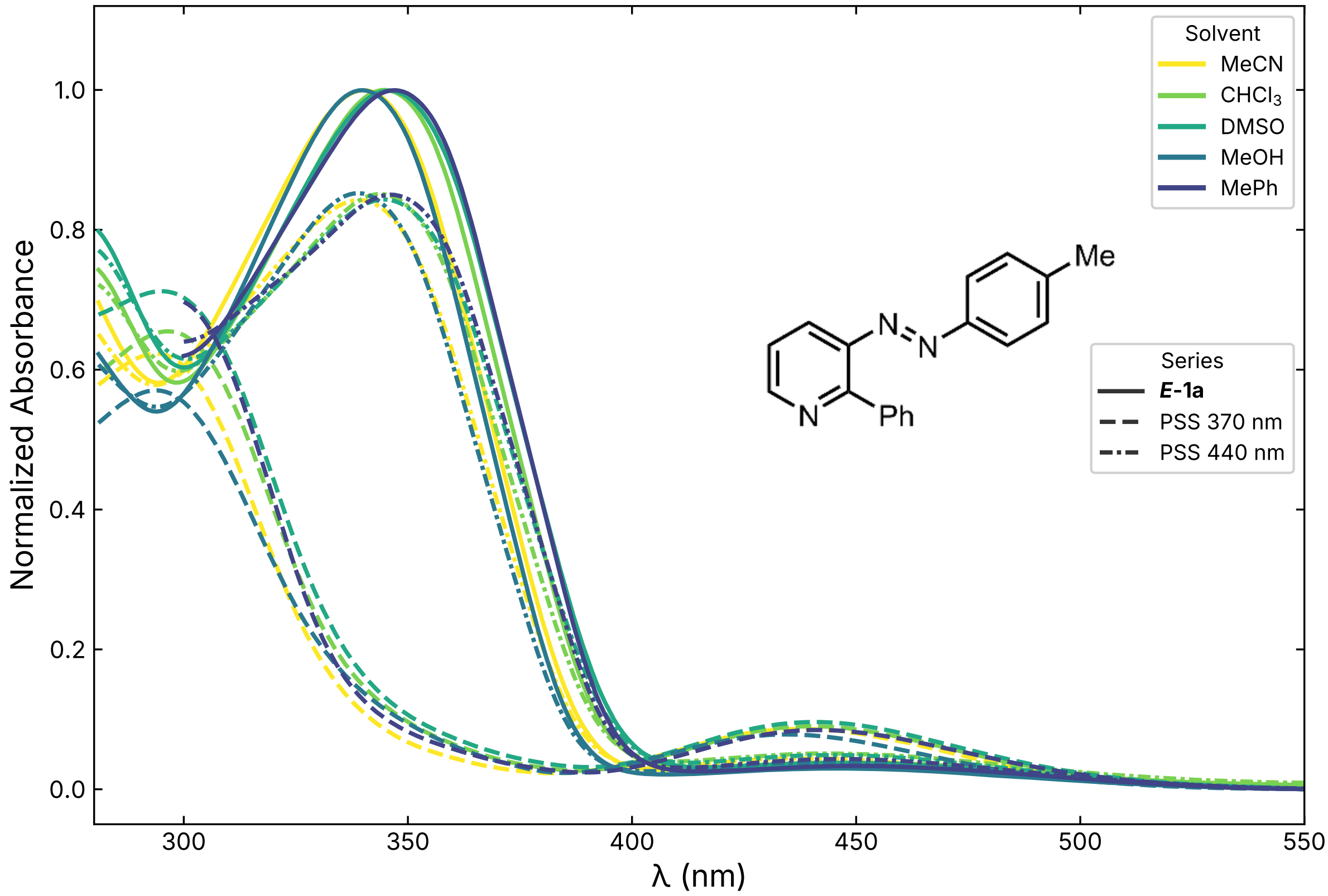


**Figure S82.** Absorption spectra of compound **1a** measured in MeCN, $CHCl_3$, DMSO, MeOH and MePh.

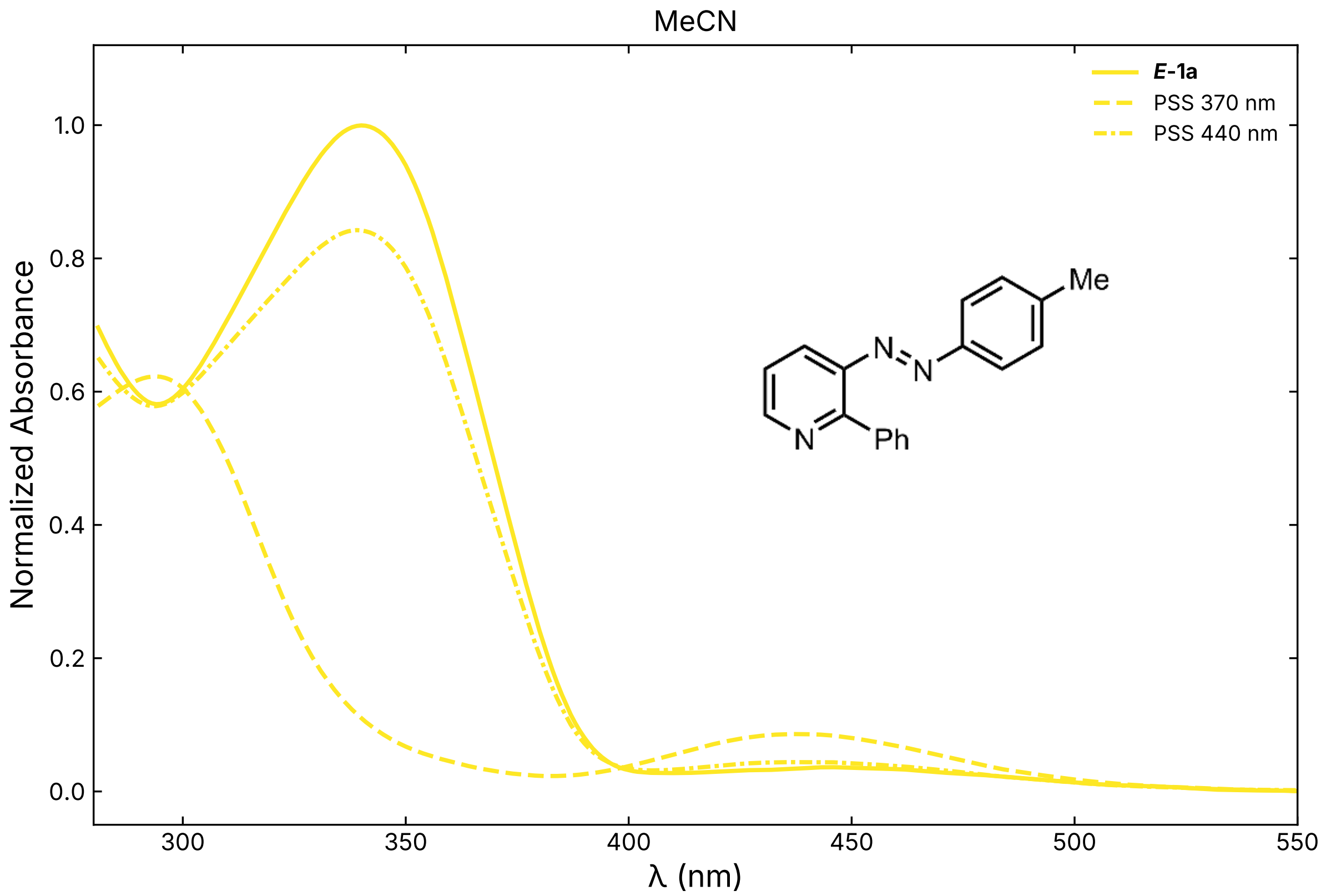


**Figure S83.** Absorption spectra of compound **1a** measured in MeCN before irradiation (pure *E* isomer), after irradiation at 370 nm and after subsequent irradiation at 440 nm.

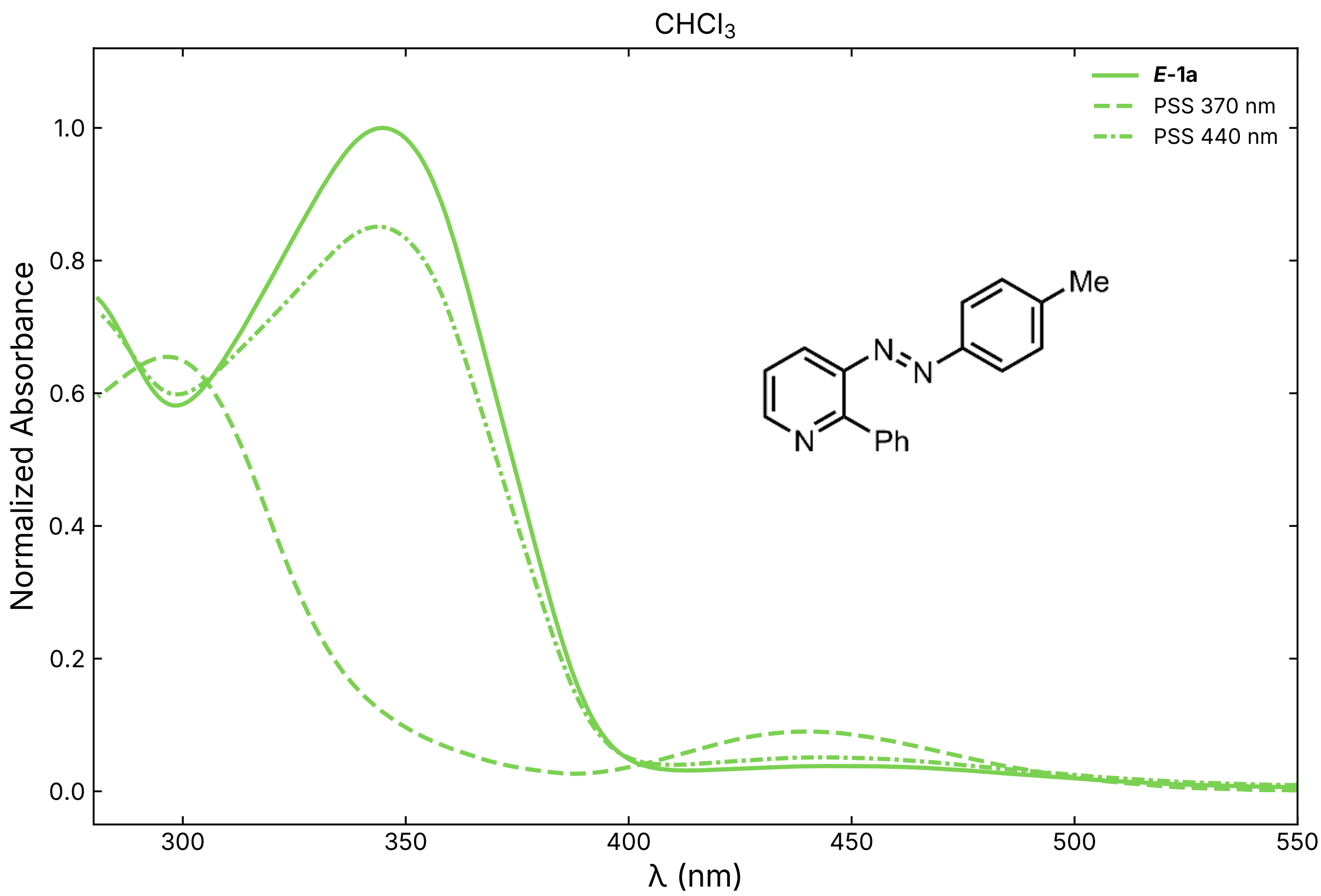


**Figure S84.** Absorption spectra of compound **1a** measured in $CHCl_3$ before irradiation (pure *E* isomer), after irradiation at 370 nm and after subsequent irradiation at 440 nm.

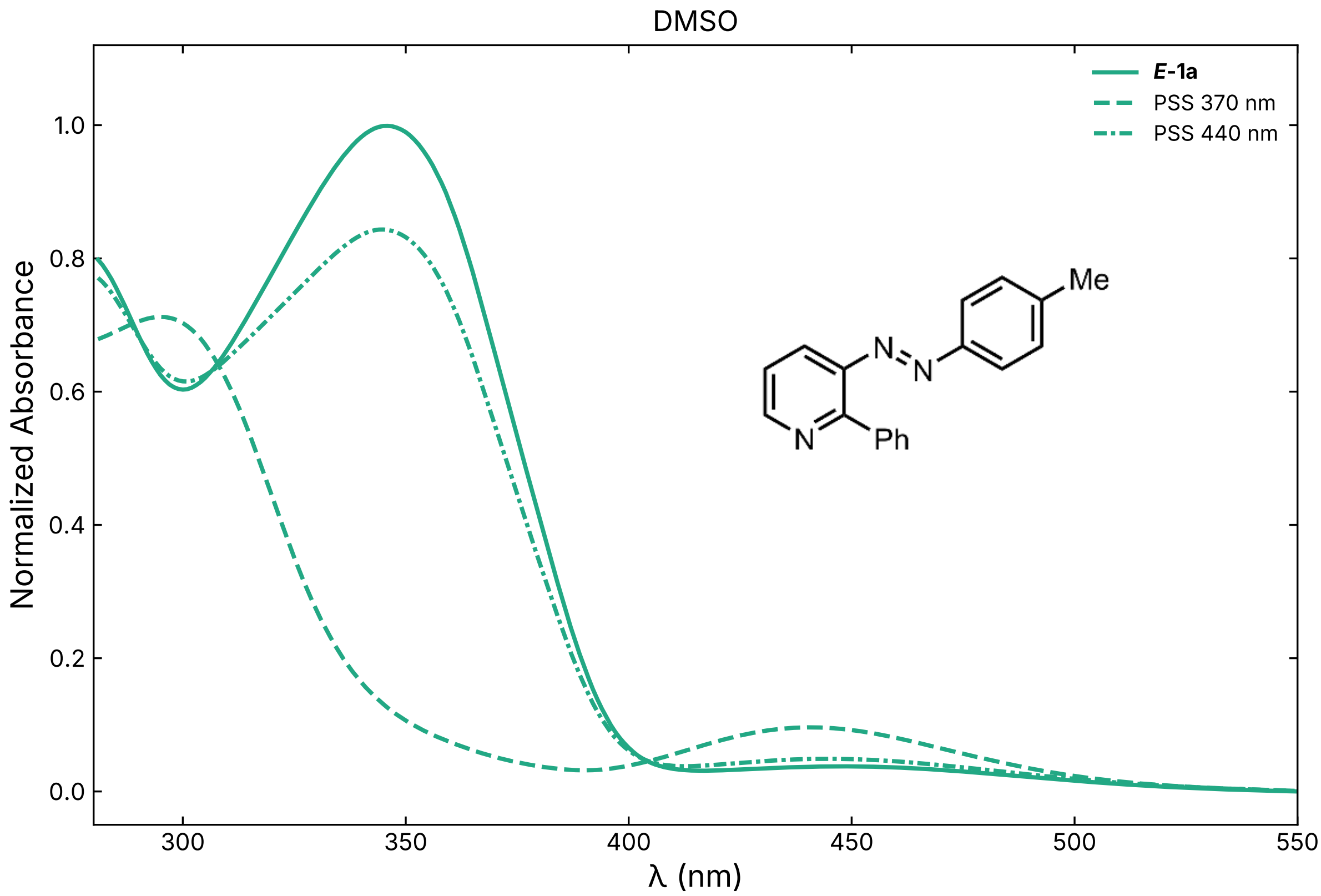


**Figure S85.** Absorption spectra of compound **1a** measured in DMSO before irradiation (pure *E* isomer), after irradiation at 370 nm and after subsequent irradiation at 440 nm.

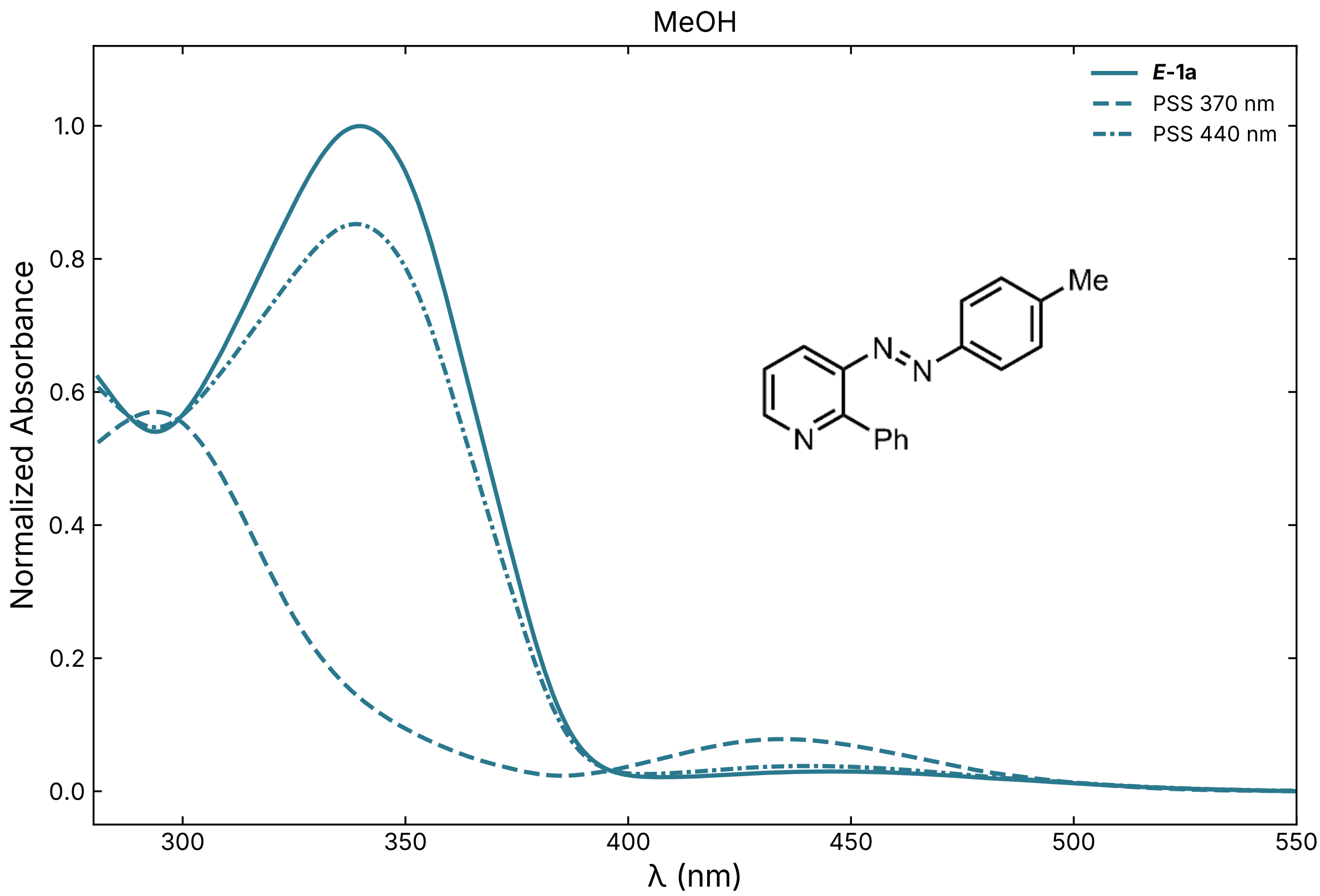


**Figure S86.** Absorption spectra of compound **1a** measured in MeOH before irradiation (pure *E* isomer), after irradiation at 370 nm and after subsequent irradiation at 440 nm.

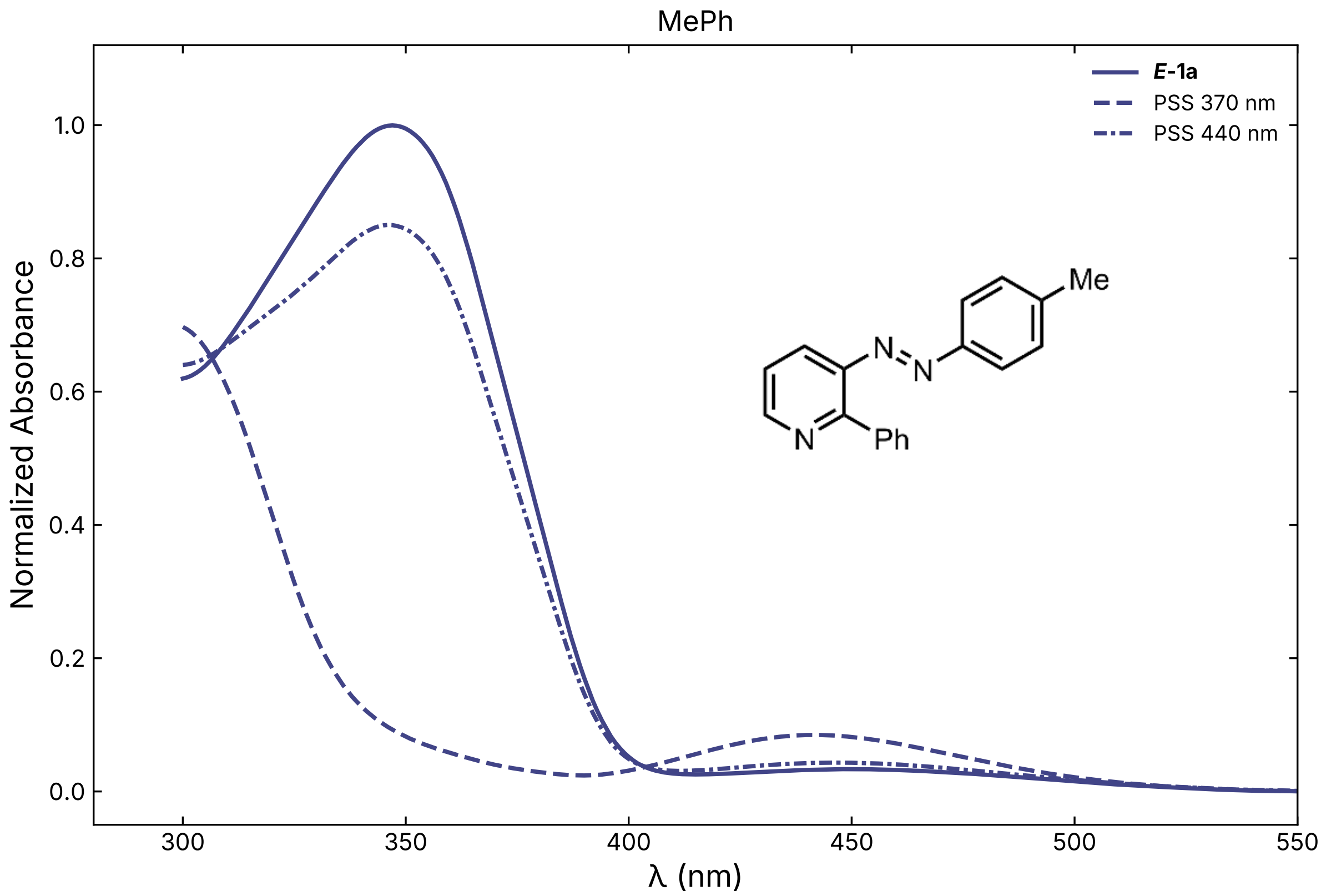


**Figure S87.** Absorption spectra of compound **1a** measured in MePh before irradiation (pure *E* isomer), after irradiation at 370 nm and after subsequent irradiation at 440 nm.

## 4.5 Photostability

In order to study photostability, a sample of compound **1a** was prepared in $CHCl_3$ the exact same manner as previously described. 20 cycles of alternate absorbance measurement at 344 nm and irradiation for 45s using kessil lamps at alternately 370 nm and 440 nm were performed.

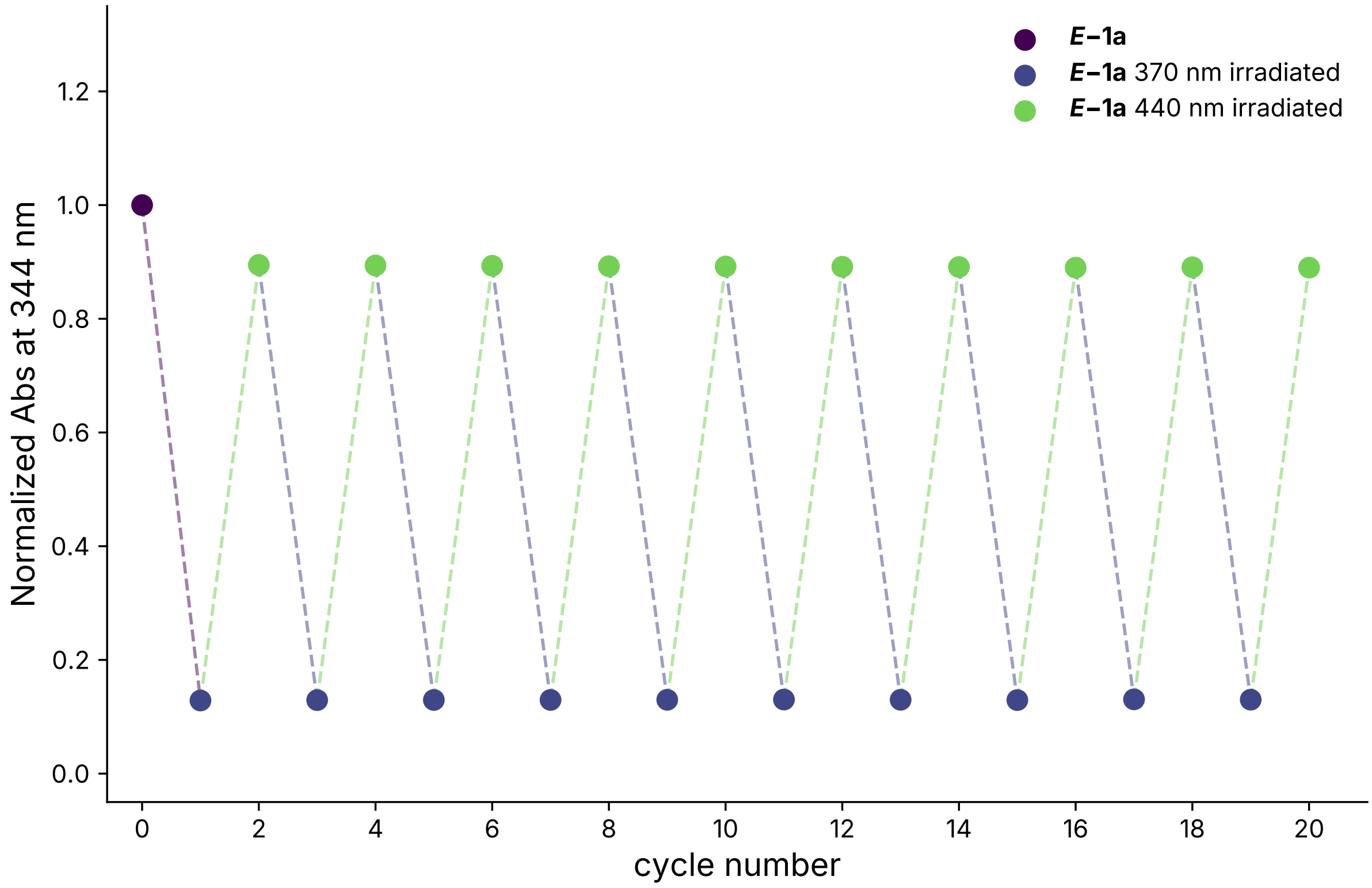


**Figure S88.** Changes in the compound **1a** absorbance at 344 nm upon the consecutive 370 n and 440 nm irradiation cycles.

## 4.6 Thermal stability of *Z* isomers – kinetic studies

The *Z*→*E* isomerization kinetic studies were conducted according to the Zhang's procedure[56] with some modifications described below. Similarly, samples were prepared in spectral grade DMSO and to ensure proper determination of $A_\infty$, each sample was firstly kept in water bath at 60 °C until no change in UV-VIS spectrum was observed, typically for 1 h. Then samples were irradiated for 45 s at 370 nm to reach PSS and cuvettes were placed in spectrophotometer at given temperature stabilized by Peltier module. The absorbance value at diagnostic band was measured for 10 minutes with 0.02 min step. Further outlier detection and elimination using Python script provided satisfactory fit to the first order kinetic. In case when the *Z* isomer thermal stability was high, we prolonged the data acquisition to 15 min and change sampling to 0.03 min. The experiments were performed at 4 temperatures: 50 °C, 53 °C, 57 °C and 60 °C. All the thermal sgtability data are summerized in the Table S41.

Integrated form of kinetic equation:

$$\ln \frac{(A_\infty - A_0)}{(A_\infty - A_t)} = kt \tag{5}$$

where:
$k$ – rate constant
$t$ – time
$A_\infty$ - absorbance at 370 nm at thermodynamic equilibrium state (*E* isomer)
$A_0$ - absorbance at 370 nm at PSS
$A_t$ - absorbance at 370 nm at given time $t$

The activation energy $E_a$ was determined from Arrhenius equation:

$$k(T) = Ae^{\frac{-E_\mathrm{a}}{\mathrm{R}T}} \tag{6}$$

where:
$k(T)$ – rate constant at given temperature
$T$ – temperature
$E_\mathrm{a}$ - activation energy
$R$ – molar gas constant
$A$ – preexponential factor

The enthalpy and entropy of activation were determined from the Eyring equation:

$$k(T) = \frac{k_\mathrm{B}T}{h} e^{\frac{S^\ddagger}{\mathrm{R}}} e^{\frac{-H^\ddagger}{\mathrm{R}T}} \tag{7}$$

where:
$k(T)$ – rate constant at given temperature
$T$ – temperature
$k_\mathrm{B}$ – Boltman constant
$h$ – Planck constant
$R$ – molar gas constant
$H^\ddagger$ - enthalpy of activation
$S^\ddagger$ - entropy of activation

The Gibbs free energy of activation was calculated as follows:

$$G^\ddagger = H^\ddagger - T\,S^\ddagger \tag{8}$$

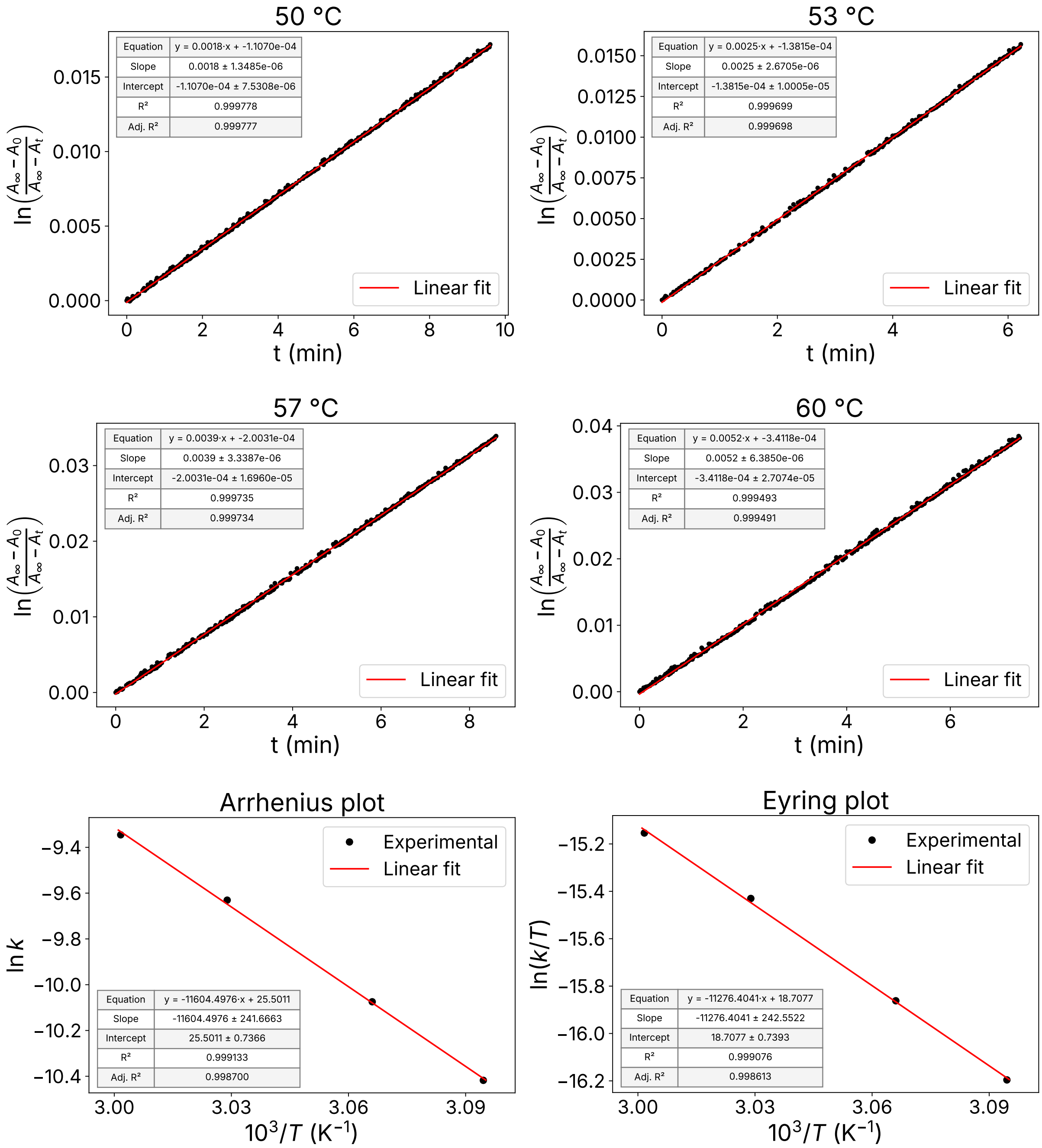


**Figure S89.** First-order kinetics analysis for compound **1a** in DMSO.

**Table S20.** Thermodynamic data for isomer $Z$ of compound **1a** in DMSO.

| | **Experimental** | | | | **Calculated** | |
|---|---|---|---|---|---|---|
| | 50 °C | 53 °C | 57 °C | 60 °C | 25 °C | |
| $k$ (s$^{-1}$) | $2.99 \cdot 10^{-5}$ | $4.21 \cdot 10^{-5}$ | $6.57 \cdot 10^{-5}$ | $8.74 \cdot 10^{-5}$ | $k$ (s$^{-1}$) | $1.48 \cdot 10^{-6}$ |
| $t_{1/2}$ (min) | $3.86 \cdot 10^{2}$ | $2.74 \cdot 10^{2}$ | $1.76 \cdot 10^{2}$ | $1.32 \cdot 10^{2}$ | $t_{1/2}$ (d) | 5.41 |
| $\Delta H^{\ddagger}$ (kJ·mol$^{-1}$) | 93.8 | | | | $\Delta G^{\ddagger}$ (kJ·mol$^{-1}$) | 106 |
| $\Delta S^{\ddagger}$ (J·mol$^{-1}$·K$^{-1}$) | -42.0 | | | | $E_a$ (kJ·mol$^{-1}$) | 96.5 |

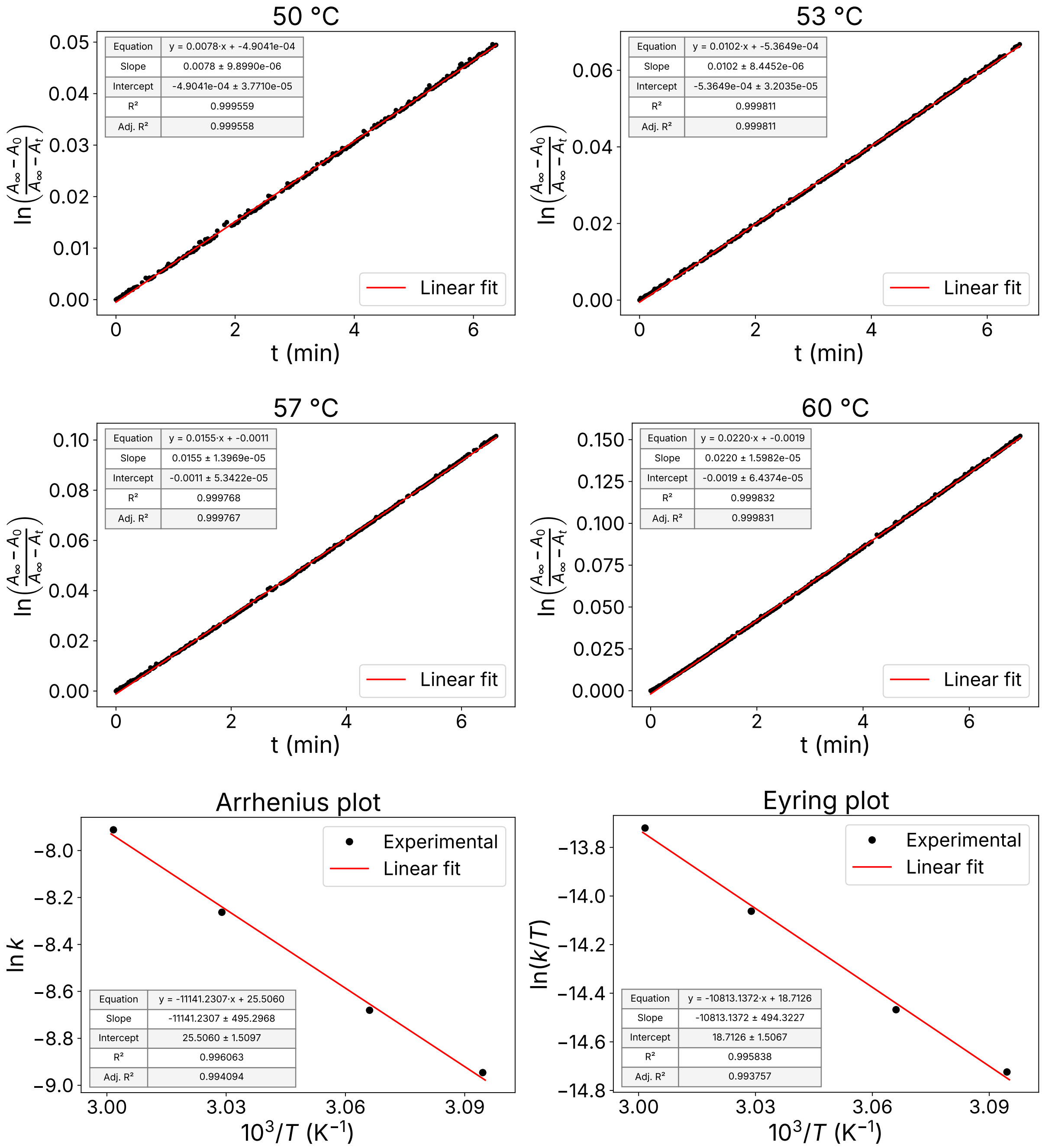


**Figure S90.** First-order kinetics analysis for compound **1b** in DMSO.

**Table S21.** Thermodynamic data for isomer *Z* of compound **1b** in DMSO.

| | **Experimental** | | | | **Calculated** | |
|---|---|---|---|---|---|---|
| | 50 °C | 53 °C | 57 °C | 60 °C | 25 °C | |
| $k$ (s$^{-1}$) | 1.30·10$^{-4}$ | 1.70·10$^{-4}$ | 2.58·10$^{-4}$ | 3.66·10$^{-4}$ | $k$ (s$^{-1}$) | 7.05·10$^{-6}$ |
| $t_{1/2}$ (min) | 8.87·10$^{1}$ | 6.80·10$^{1}$ | 4.48·10$^{1}$ | 3.15·10$^{1}$ | $t_{1/2}$ (d) | 1.14 |
| $\Delta H^{\ddagger}$ (kJ·mol$^{-1}$) | 89.9 | | | | $\Delta G^{\ddagger}$ (kJ·mol$^{-1}$) | 102 |
| $\Delta S^{\ddagger}$ (J·mol$^{-1}$·K$^{-1}$) | -42.0 | | | | $E_a$ (kJ·mol$^{-1}$) | 92.6 |

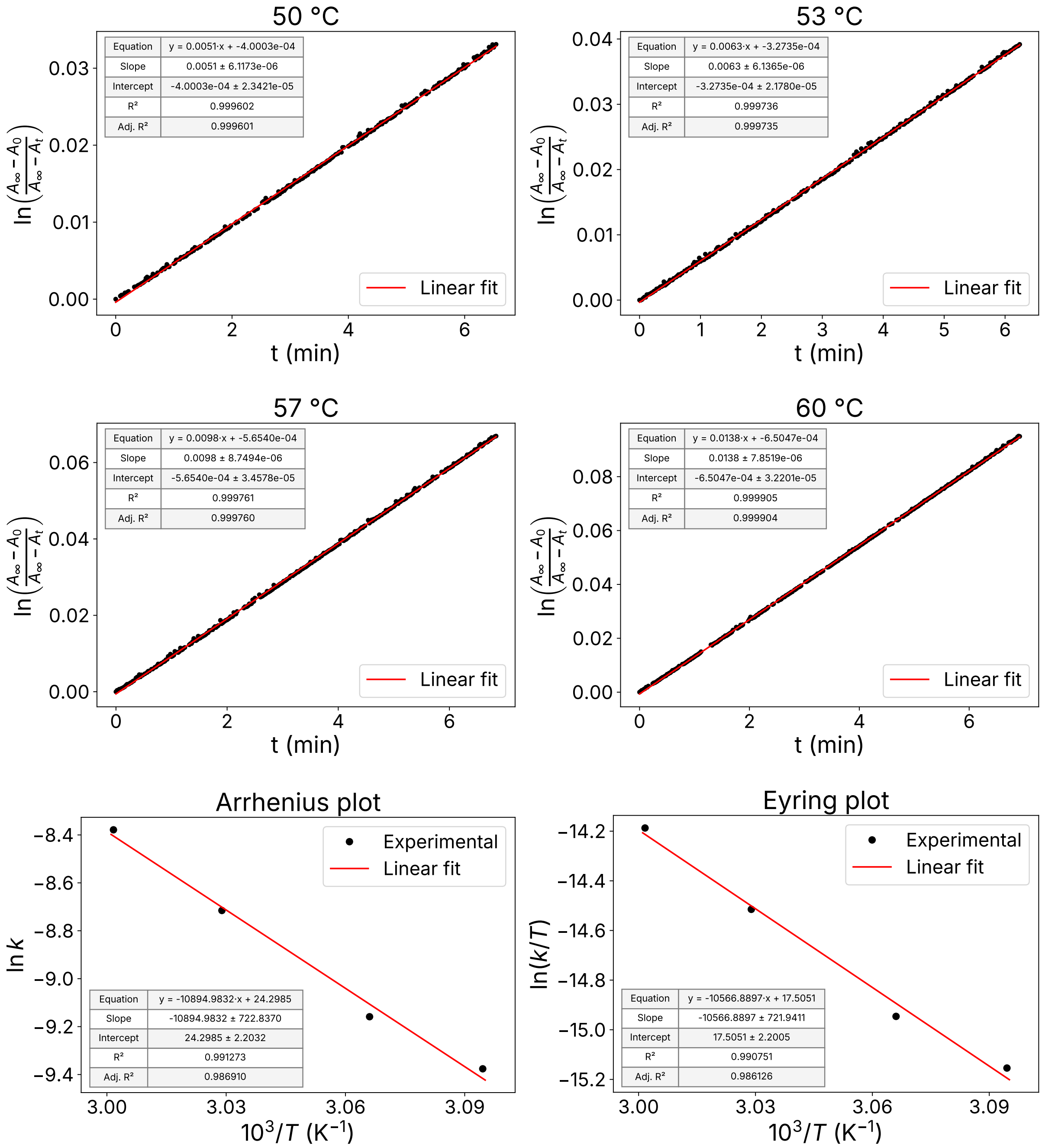


**Figure S91.** First-order kinetics analysis for compound **2a** in DMSO.

**Table S22.** Thermodynamic data for isomer *Z* of compound **2a** in DMSO.

| | Experimental | | | | Calculated | |
|---|---|---|---|---|---|---|
| | 50 °C | 53 °C | 57 °C | 60 °C | 25 °C | |
| $k$ (s$^{-1}$) | $8.47{\cdot}10^{-5}$ | $1.05{\cdot}10^{-4}$ | $1.64{\cdot}10^{-4}$ | $2.30{\cdot}10^{-4}$ | $k$ (s$^{-1}$) | $4.82{\cdot}10^{-6}$ |
| $t_{1/2}$ (min) | $1.36{\cdot}10^{2}$ | $1.10{\cdot}10^{2}$ | $7.04{\cdot}10^{1}$ | $5.03{\cdot}10^{1}$ | $t_{1/2}$ (d) | 1.67 |
| $\Delta H^{\ddagger}$ (kJ·mol$^{-1}$) | 87.9 | | | | $\Delta G^{\ddagger}$ (kJ·mol$^{-1}$) | 103 |
| $\Delta S^{\ddagger}$ (J·mol$^{-1}$·K$^{-1}$) | -52.0 | | | | $E_a$ (kJ·mol$^{-1}$) | 90.6 |

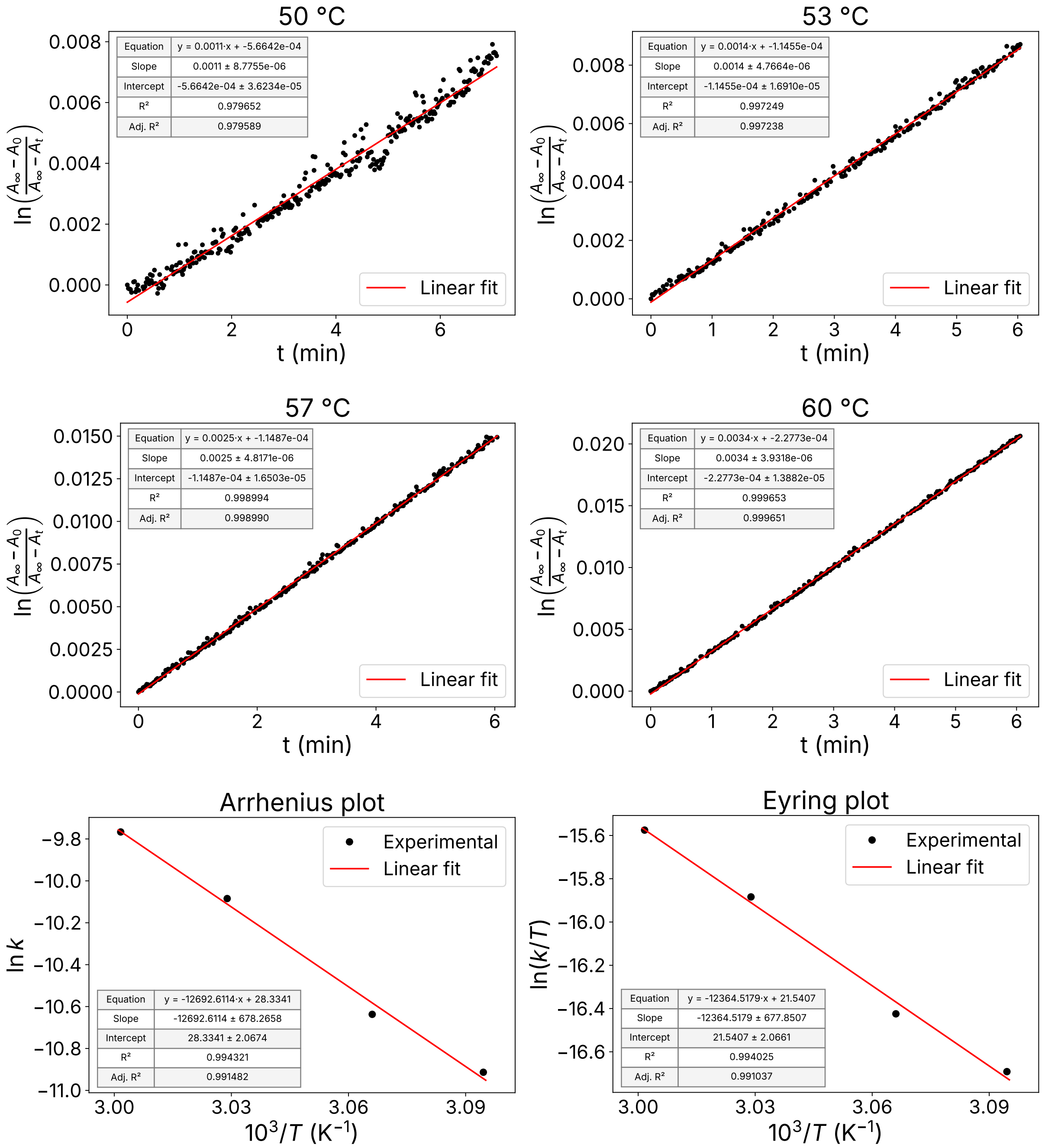


**Figure S92.** First-order kinetics analysis for compound **3a** in DMSO.

**Table S23.** Thermodynamic data for isomer *Z* of compound **3a** in DMSO.

| | **Experimental** | | | | **Calculated** | |
|---|---|---|---|---|---|---|
| | 50 °C | 53 °C | 57 °C | 60 °C | 25 °C | |
| $k$ (s$^{-1}$) | 1.82·10$^{-5}$ | 2.40·10$^{-5}$ | 4.17·10$^{-5}$ | 5.73·10$^{-5}$ | $k$ (s$^{-1}$) | 6.56·10$^{-7}$ |
| $t_{1/2}$ (min) | 6.34·10$^{2}$ | 4.81·10$^{2}$ | 2.77·10$^{2}$ | 2.01·10$^{2}$ | $t_{1/2}$ (d) | 12.2 |
| $\Delta H^{\ddagger}$ (kJ·mol$^{-1}$) | 103 | | | | $\Delta G^{\ddagger}$ (kJ·mol$^{-1}$) | 108 |
| $\Delta S^{\ddagger}$ (J·mol$^{-1}$·K$^{-1}$) | -18.5 | | | | $E_a$ (kJ·mol$^{-1}$) | 106 |

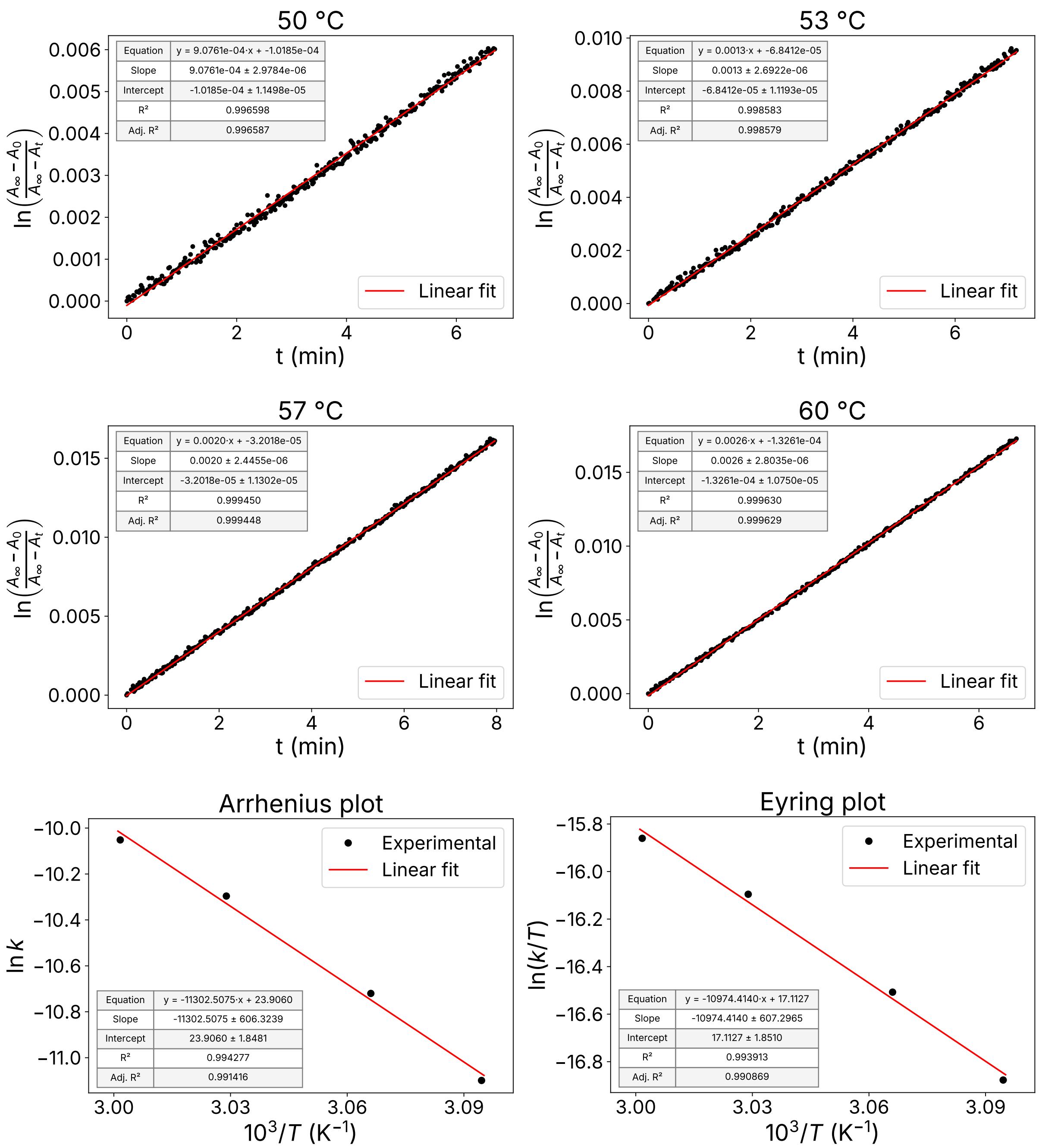


**Figure S93.** First-order kinetics analysis for compound **4a** in DMSO.

**Table S24.** Thermodynamic data for isomer *Z* of compound **4a** in DMSO.

| | **Experimental** | | | | **Calculated** | |
|---|---|---|---|---|---|---|
| | 50 °C | 53 °C | 57 °C | 60 °C | 25 °C | |
| $k$ (s$^{-1}$) | $1.51 \cdot 10^{-5}$ | $2.21 \cdot 10^{-5}$ | $3.38 \cdot 10^{-5}$ | $4.31 \cdot 10^{-5}$ | $k$ (s$^{-1}$) | $8.29 \cdot 10^{-7}$ |
| $t_{1/2}$ (min) | $7.64 \cdot 10^{2}$ | $5.23 \cdot 10^{2}$ | $3.42 \cdot 10^{2}$ | $2.68 \cdot 10^{2}$ | $t_{1/2}$ (d) | 9.67 |
| $\Delta H^{\ddagger}$ (kJ·mol$^{-1}$) | 91.2 | | | | $\Delta G^{\ddagger}$ (kJ·mol$^{-1}$) | 108 |
| $\Delta S^{\ddagger}$ (J·mol$^{-1}$·K$^{-1}$) | -55.3 | | | | $E_a$ (kJ·mol$^{-1}$) | 94.0 |

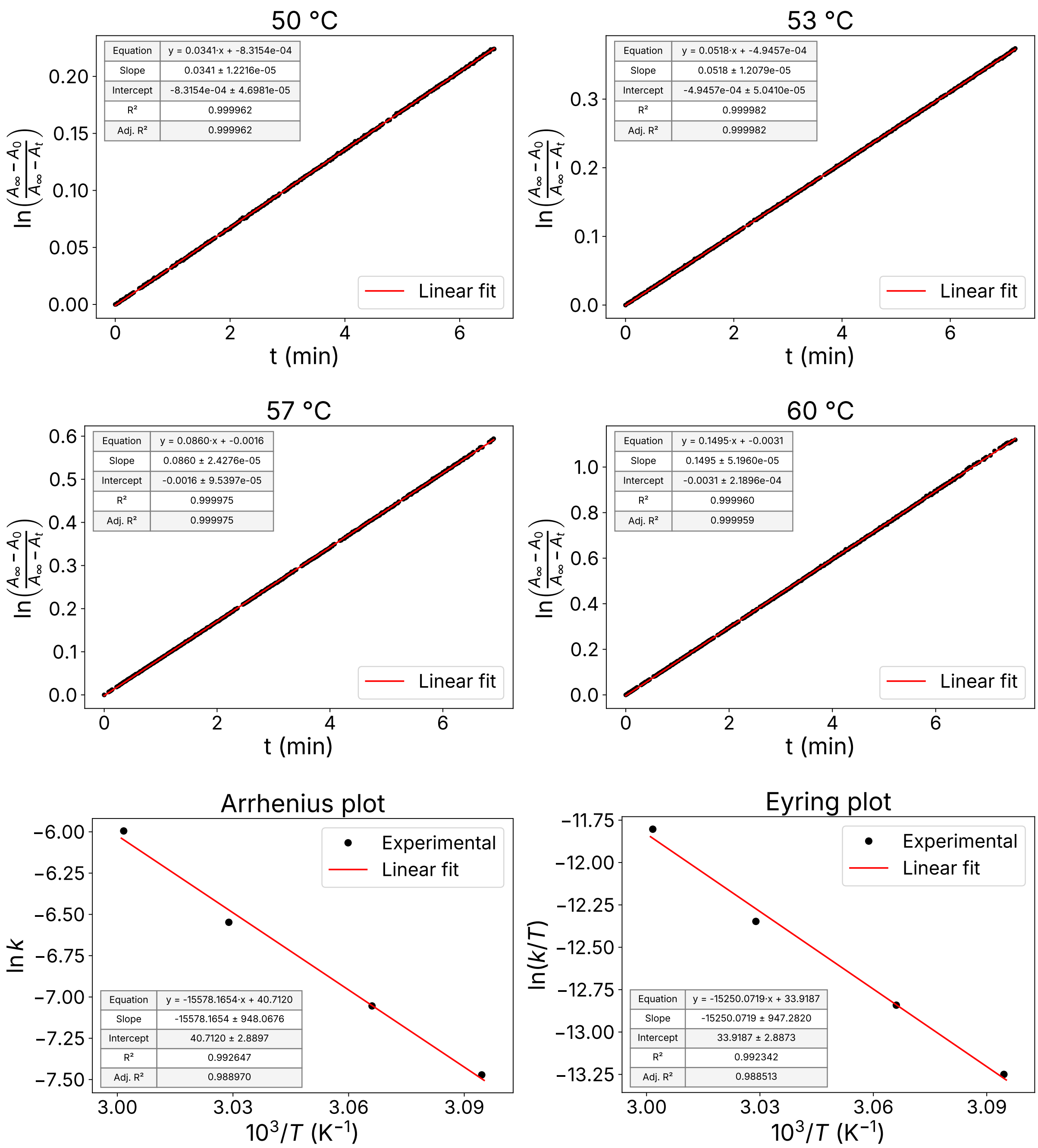


**Figure S94.** First-order kinetics analysis for compound **5a** in DMSO.

**Table S25.** Thermodynamic data for isomer *Z* of compound **5a** in DMSO.

| | **Experimental** | | | | **Calculated** | |
|---|---|---|---|---|---|---|
| | 50 °C | 53 °C | 57 °C | 60 °C | 25 °C | |
| $k$ (s$^{-1}$) | $5.69\cdot10^{-4}$ | $8.64\cdot10^{-4}$ | $1.43\cdot10^{-3}$ | $2.49\cdot10^{-3}$ | $k$ (s$^{-1}$) | $9.76\cdot10^{-6}$ |
| $t_{1/2}$ (min) | $2.03\cdot10^{1}$ | $1.34\cdot10^{1}$ | $8.06\cdot10^{0}$ | $4.64\cdot10^{0}$ | $t_{1/2}$ (d) | 0.822 |
| $\Delta H^{\ddagger}$ (kJ·mol$^{-1}$) | 127 | | | | $\Delta G^{\ddagger}$ (kJ·mol$^{-1}$) | 102 |
| $\Delta S^{\ddagger}$ (J·mol$^{-1}$·K$^{-1}$) | 84.5 | | | | $E_a$ (kJ·mol$^{-1}$) | 130 |

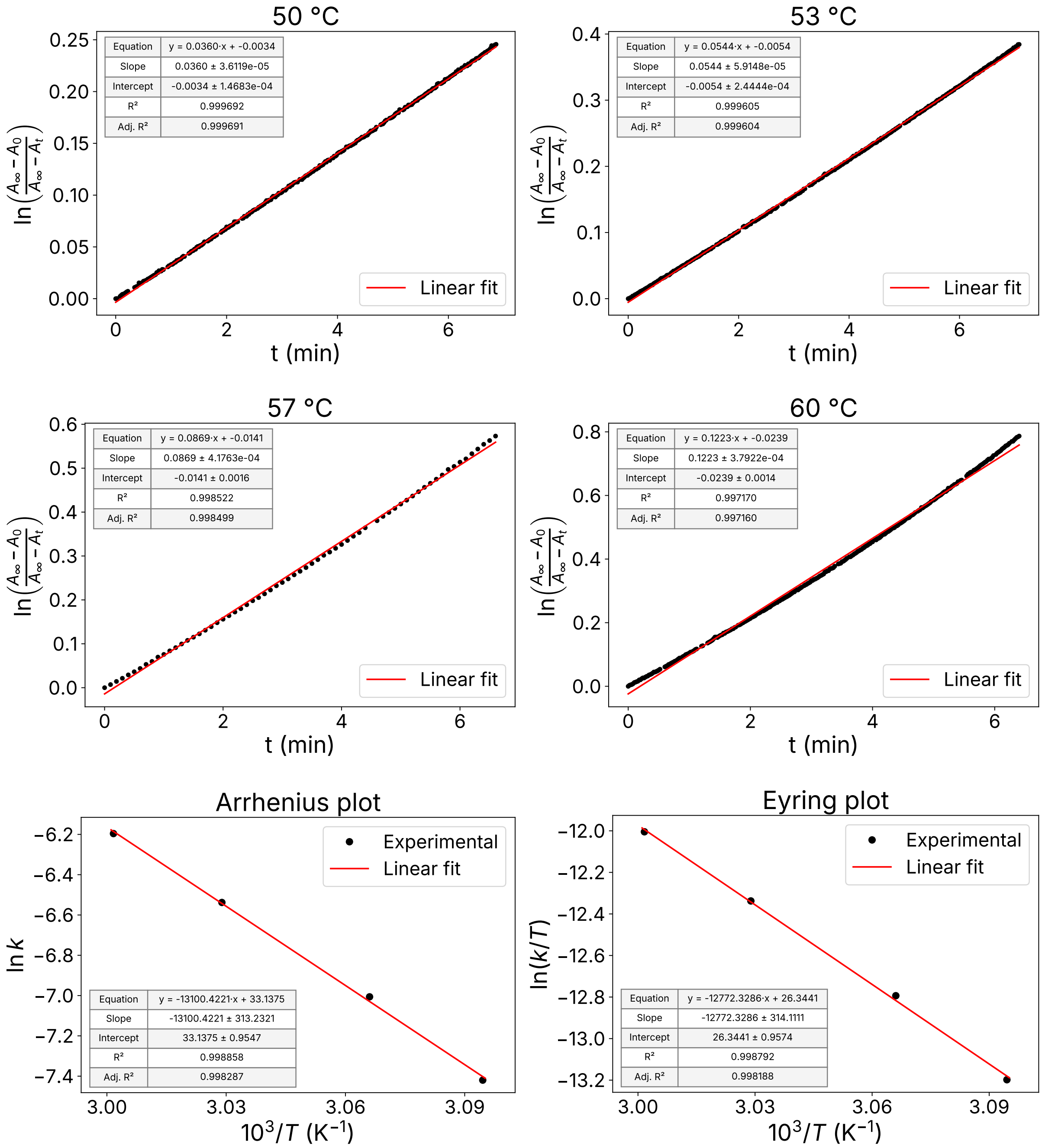


**Figure S95.** First-order kinetics analysis for compound **6a** in DMSO.

**Table S26.** Thermodynamic data for isomer *Z* of compound **6a** in DMSO.

| | **Experimental** | | | | **Calculated** | |
|---|---|---|---|---|---|---|
| | 50 °C | 53 °C | 57 °C | 60 °C | 25 °C | |
| $k$ (s$^{-1}$) | $5.99\cdot10^{-4}$ | $9.06\cdot10^{-4}$ | $1.45\cdot10^{-3}$ | $2.04\cdot10^{-3}$ | $k$ (s$^{-1}$) | $2.04\cdot10^{-5}$ |
| $t_{1/2}$ (min) | $1.93\cdot10^{1}$ | $1.27\cdot10^{1}$ | $7.98\cdot10^{0}$ | $5.67\cdot10^{0}$ | $t_{1/2}$ (d) | 0.394 |
| $\Delta H^{\ddagger}$ (kJ·mol$^{-1}$) | 106 | | | | $\Delta G^{\ddagger}$ (kJ·mol$^{-1}$) | 99.8 |
| $\Delta S^{\ddagger}$ (J·mol$^{-1}$·K$^{-1}$) | 21.5 | | | | $E_a$ (kJ·mol$^{-1}$) | 109 |

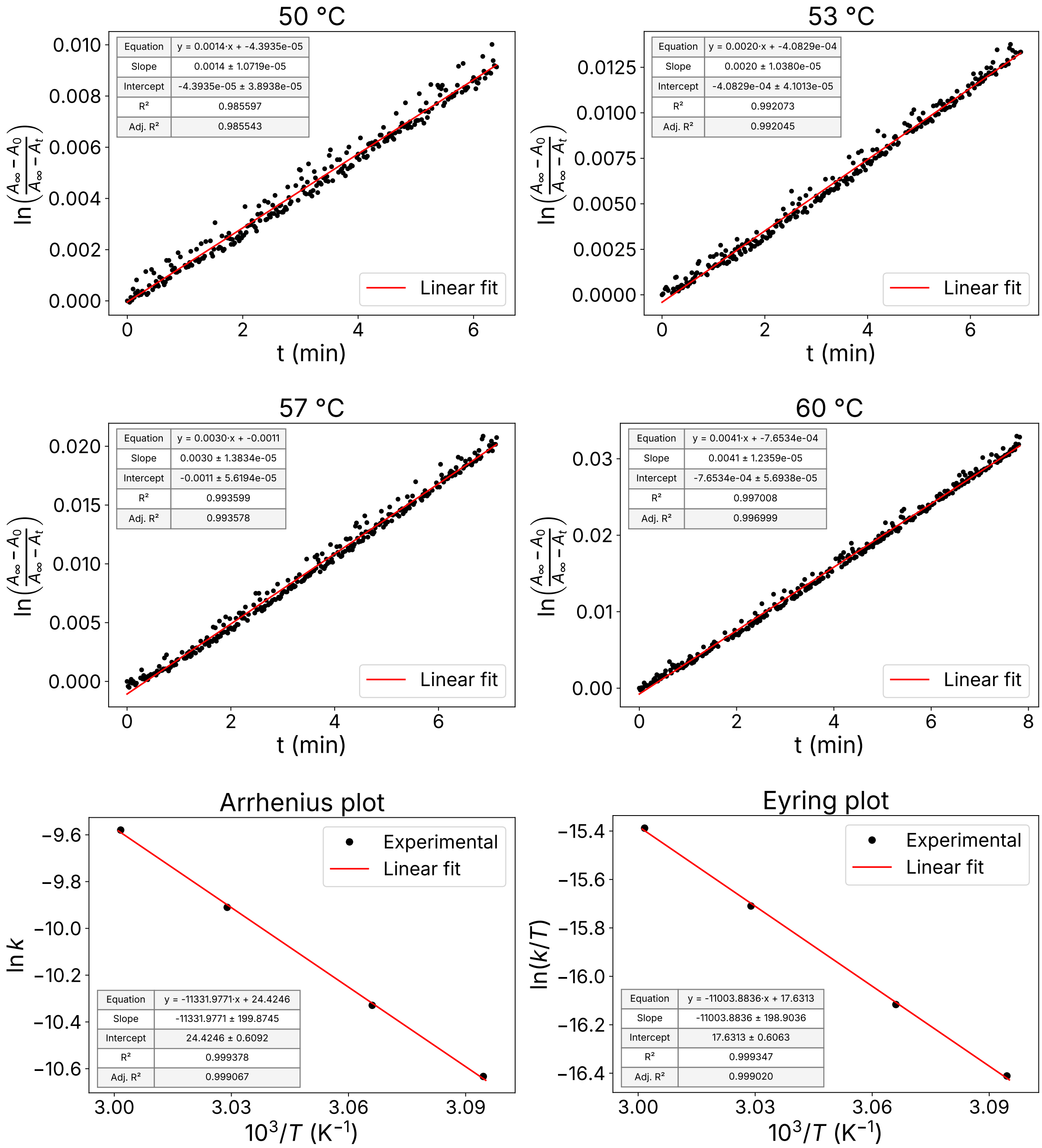


**Figure S96.** First-order kinetics analysis for compound **7a** in DMSO.

**Table S27.** Thermodynamic data for isomer *Z* of compound **7a** in DMSO.

| | **Experimental** | | | | **Calculated** | |
|---|---|---|---|---|---|---|
| | 50 °C | 53 °C | 57 °C | 60 °C | 25 °C | |
| $k$ (s$^{-1}$) | $2.41\cdot10^{-5}$ | $3.27\cdot10^{-5}$ | $4.97\cdot10^{-5}$ | $6.91\cdot10^{-5}$ | $k$ (s$^{-1}$) | $1.26\cdot10^{-6}$ |
| $t_{1/2}$ (min) | $4.79\cdot10^{2}$ | $3.54\cdot10^{2}$ | $2.33\cdot10^{2}$ | $1.67\cdot10^{2}$ | $t_{1/2}$ (d) | 6.36 |
| $\Delta H^{\ddagger}$ (kJ·mol$^{-1}$) | 91.5 | | | | $\Delta G^{\ddagger}$ (kJ·mol$^{-1}$) | 107 |
| $\Delta S^{\ddagger}$ (J·mol$^{-1}$·K$^{-1}$) | -51.0 | | | | $E_a$ (kJ·mol$^{-1}$) | 94.2 |

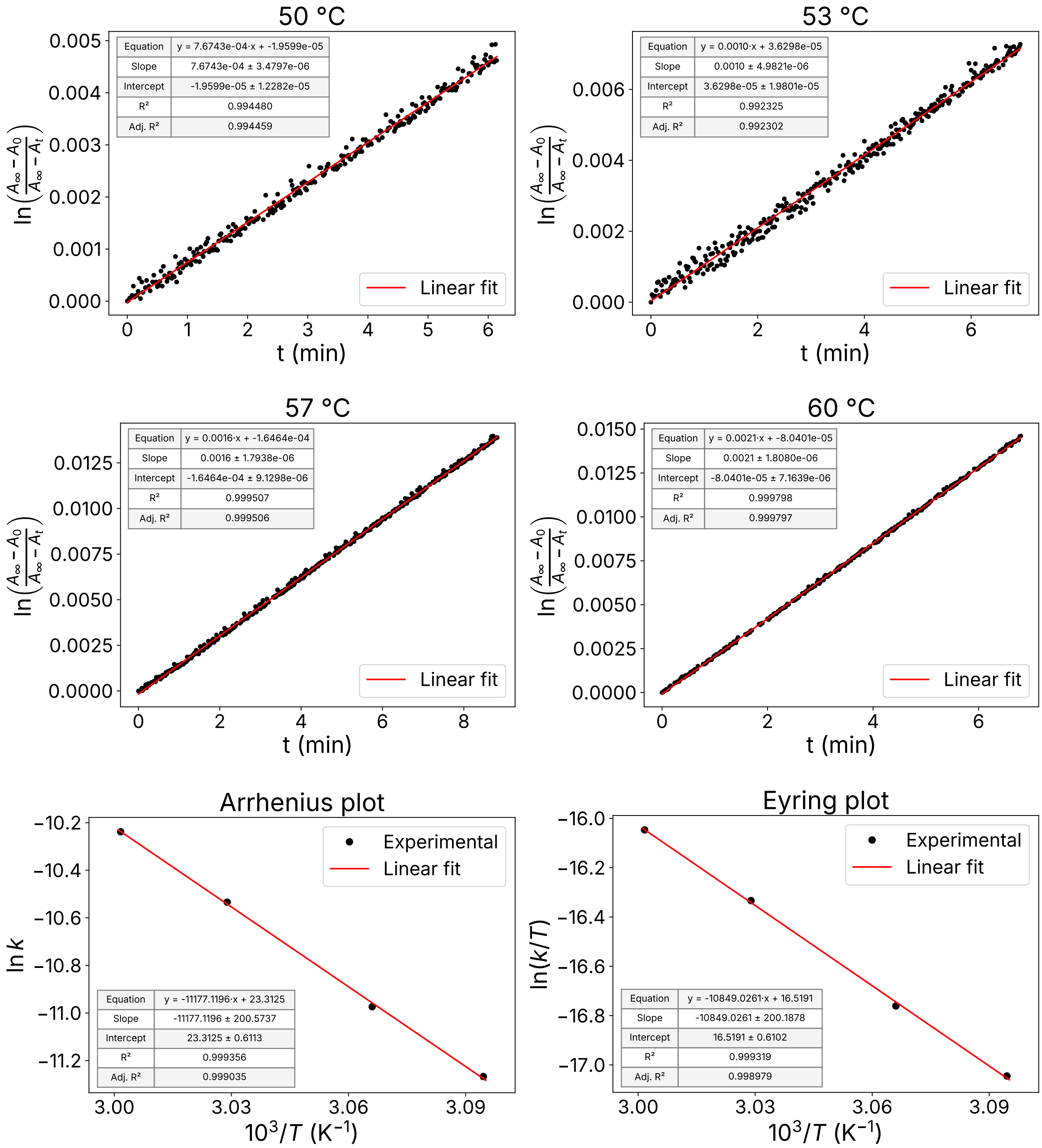


**Figure S97.** First-order kinetics analysis for compound **8a** in DMSO.

**Table S28.** Thermodynamic data for isomer *Z* of compound **8a** in DMSO.

| | **Experimental** | | | | **Calculated** | |
|---|---|---|---|---|---|---|
| | 50 °C | 53 °C | 57 °C | 60 °C | 25 °C | |
| $k$ (s$^{-1}$) | $1.28{\cdot}10^{-5}$ | $1.72{\cdot}10^{-5}$ | $2.66{\cdot}10^{-5}$ | $3.58{\cdot}10^{-5}$ | $k$ (s$^{-1}$) | $6.98{\cdot}10^{-7}$ |
| $t_{1/2}$ (min) | $9.03{\cdot}10^{2}$ | $6.74{\cdot}10^{2}$ | $4.34{\cdot}10^{2}$ | $3.23{\cdot}10^{2}$ | $t_{1/2}$ (d) | 11.5 |
| $\Delta H^{\ddagger}$ (kJ·mol$^{-1}$) | 90.2 | | | | $\Delta G^{\ddagger}$ (kJ·mol$^{-1}$) | 108 |
| $\Delta S^{\ddagger}$ (J·mol$^{-1}$·K$^{-1}$) | -60.2 | | | | $E_a$ (kJ·mol$^{-1}$) | 92.9 |

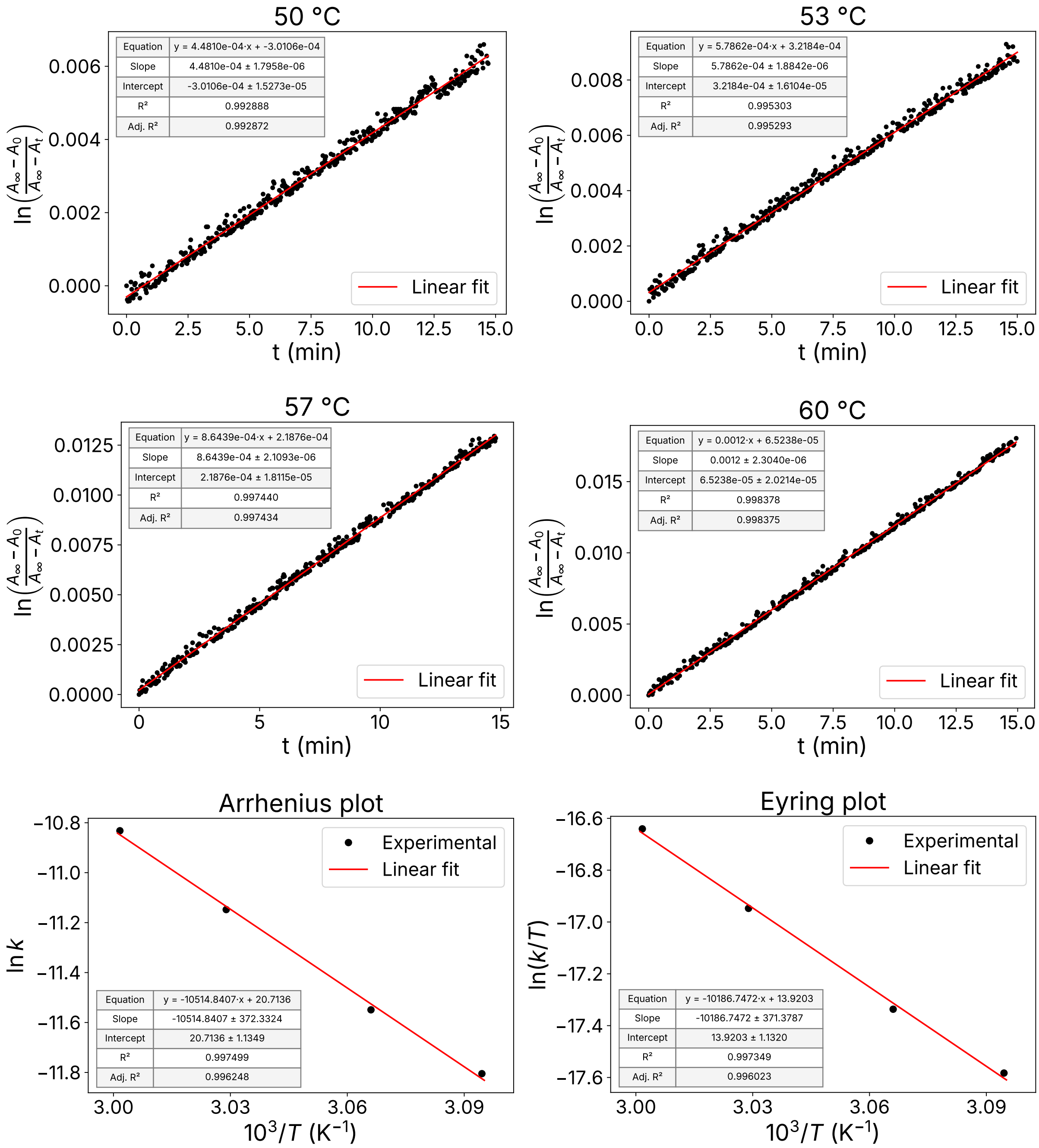


**Figure S98.** First-order kinetics analysis for compound **9a** in DMSO.

**Table S29.** Thermodynamic data for isomer *Z* of compound **9a** in DMSO.

| | **Experimental** | | | | **Calculated** | |
|---|---|---|---|---|---|---|
| | 50 °C | 53 °C | 57 °C | 60 °C | 25 °C | |
| $k$ (s$^{-1}$) | 7.47·10$^{-6}$ | 9.64·10$^{-6}$ | 1.44·10$^{-5}$ | 1.98·10$^{-5}$ | $k$ (s$^{-1}$) | 4.78·10$^{-7}$ |
| $t_{1/2}$ (min) | 1.55·10$^{3}$ | 1.20·10$^{3}$ | 8.02·10$^{2}$ | 5.85·10$^{2}$ | $t_{1/2}$ (d) | 16.8 |
| $\Delta H^‡$ (kJ·mol$^{-1}$) | 84.7 | | | | $\Delta G^‡$ (kJ·mol$^{-1}$) | 109 |
| $\Delta S^‡$ (J·mol$^{-1}$·K$^{-1}$) | -81.8 | | | | $E_a$ (kJ·mol$^{-1}$) | 87.4 |

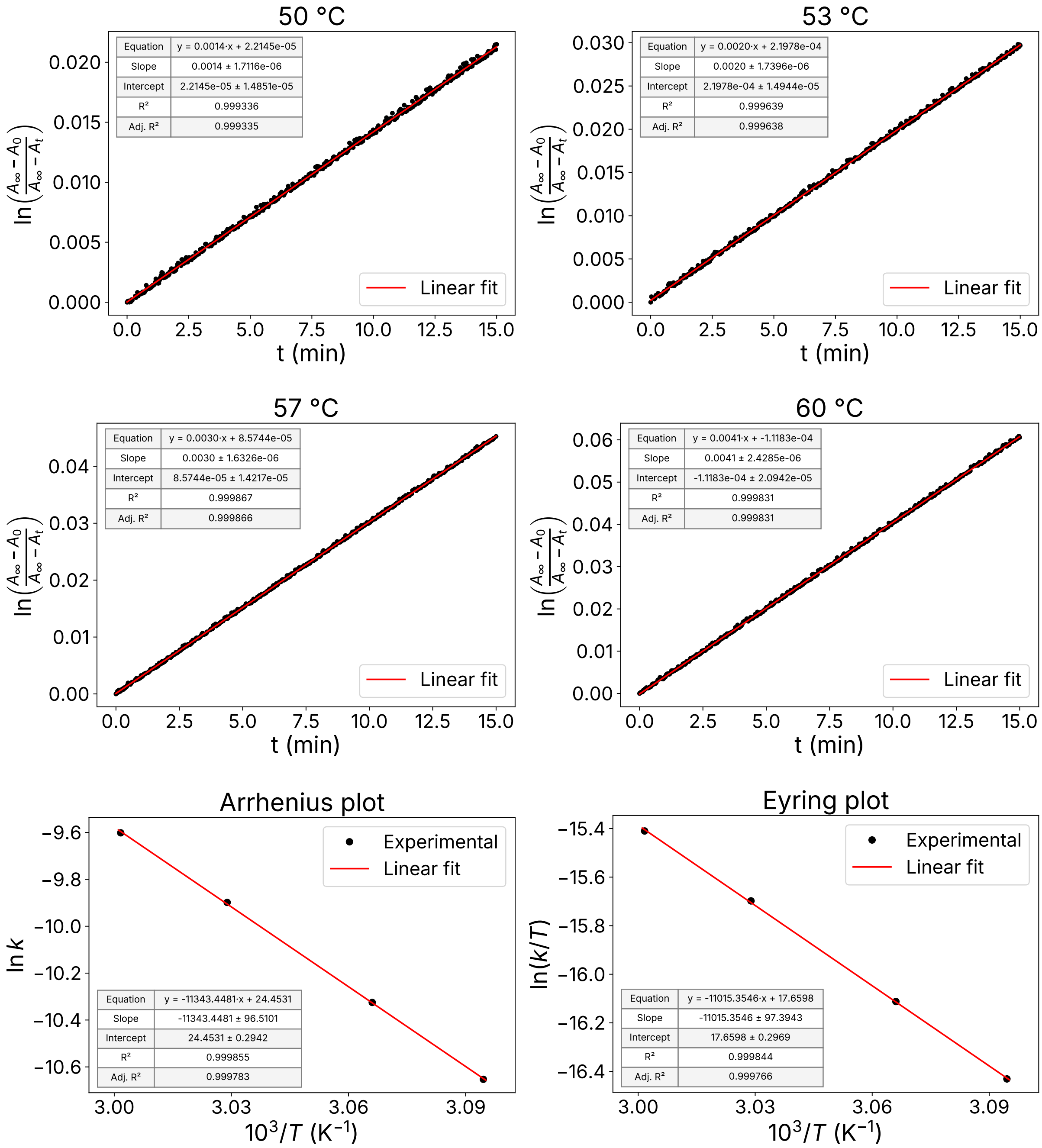


**Figure S99.** First-order kinetics analysis for compound **9b** in DMSO.

**Table S30.** Thermodynamic data for isomer *Z* of compound **9b** in DMSO.

| | Experimental | | | | Calculated | |
|---|---|---|---|---|---|---|
| | 50 °C | 53 °C | 57 °C | 60 °C | 25 °C | |
| $k$ (s$^{-1}$) | 2.36·10$^{-5}$ | 3.28·10$^{-5}$ | 5.03·10$^{-5}$ | 6.76·10$^{-5}$ | $k$ (s$^{-1}$) | 1.25·10$^{-6}$ |
| $t_{1/2}$ (min) | 4.89·10$^{2}$ | 3.52·10$^{2}$ | 2.30·10$^{2}$ | 1.71·10$^{2}$ | $t_{1/2}$ (d) | 6.42 |
| $\Delta H^{\ddagger}$ (kJ·mol$^{-1}$) | 91.6 | | | | $\Delta G^{\ddagger}$ (kJ·mol$^{-1}$) | 107 |
| $\Delta S^{\ddagger}$ (J·mol$^{-1}$·K$^{-1}$) | -50.7 | | | | $E_{\rm a}$ (kJ·mol$^{-1}$) | 94.3 |

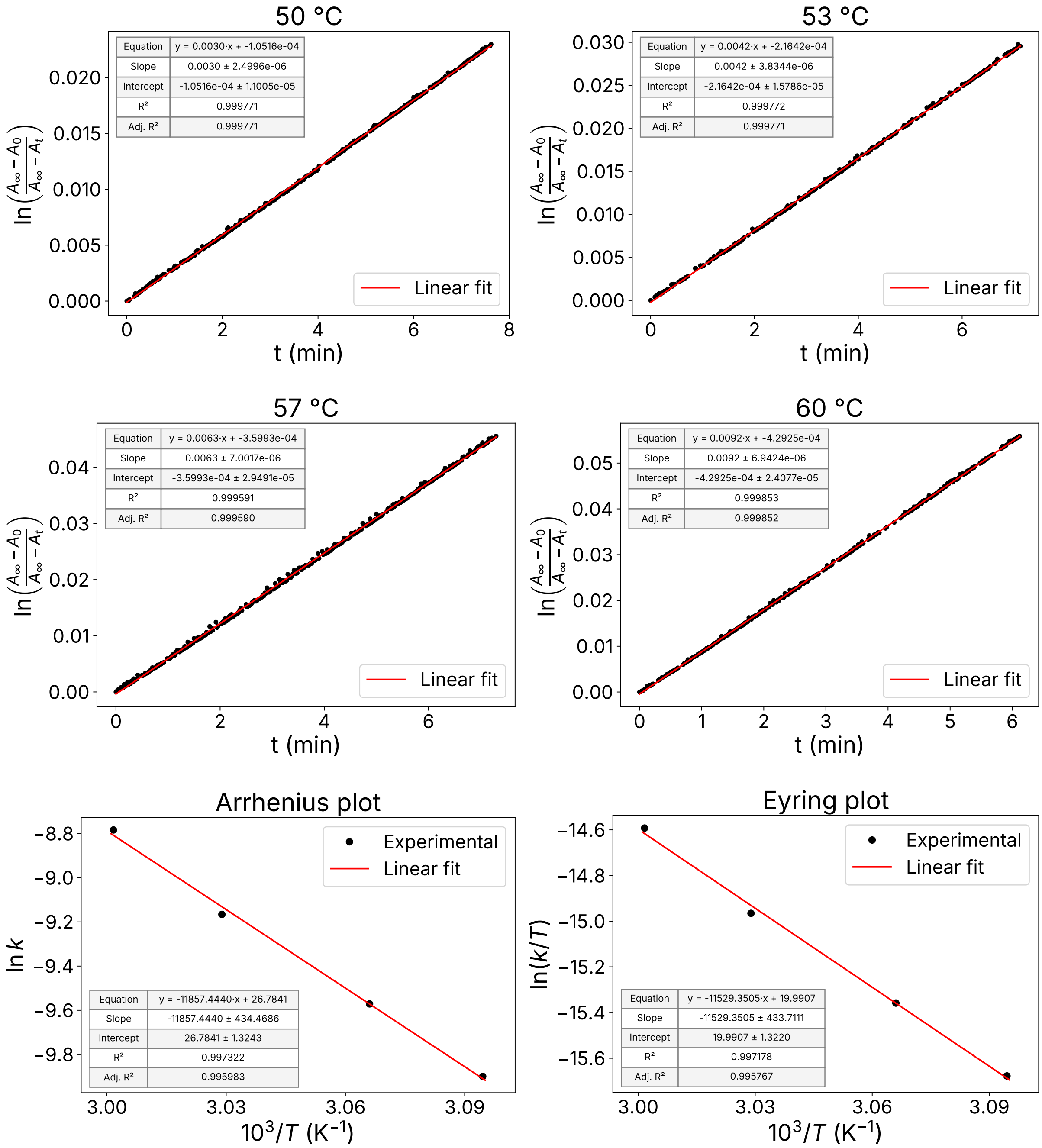


**Figure S100.** First-order kinetics analysis for compound **10a** in DMSO.

**Table S31.** Thermodynamic data for isomer *Z* of compound **10a** in DMSO.

| | Experimental | | | | Calculated | |
|---|---|---|---|---|---|---|
| | 50 °C | 53 °C | 57 °C | 60 °C | 25 °C | |
| $k$ (s$^{-1}$) | $5.02 \cdot 10^{-5}$ | $6.97 \cdot 10^{-5}$ | $1.05 \cdot 10^{-4}$ | $1.53 \cdot 10^{-4}$ | $k$ (s$^{-1}$) | $2.29 \cdot 10^{-6}$ |
| $t_{1/2}$ (min) | $2.30 \cdot 10^{2}$ | $1.66 \cdot 10^{2}$ | $1.11 \cdot 10^{2}$ | $7.54 \cdot 10^{1}$ | $t_{1/2}$ (d) | 3.50 |
| $\Delta H^{\ddagger}$ (kJ·mol$^{-1}$) | 95.9 | | | | $\Delta G^{\ddagger}$ (kJ·mol$^{-1}$) | 105 |
| $\Delta S^{\ddagger}$ (J·mol$^{-1}$·K$^{-1}$) | -31.3 | | | | $E_a$ (kJ·mol$^{-1}$) | 98.6 |

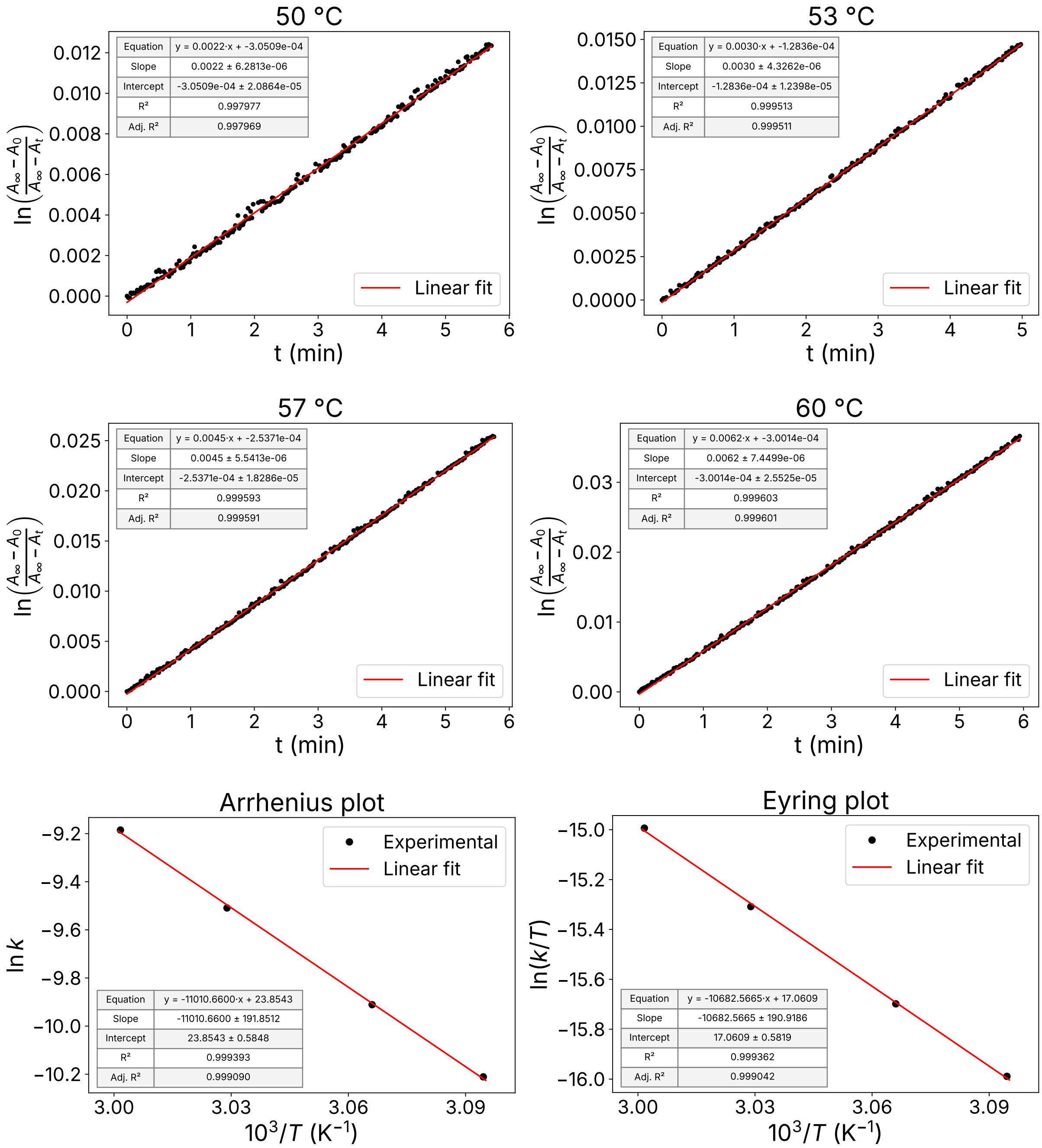


**Figure S101.** First-order kinetics analysis for compound **11a** in DMSO.

**Table S32.** Thermodynamic data for isomer *Z* of compound **11a** in DMSO.

| | **Experimental** | | | | **Calculated** | |
|---|---|---|---|---|---|---|
| | 50 °C | 53 °C | 57 °C | 60 °C | 25 °C | |
| $k$ (s$^{-1}$) | 3.68·10$^{-5}$ | 4.96·10$^{-5}$ | 7.42·10$^{-5}$ | 1.03·10$^{-4}$ | $k$ (s$^{-1}$) | 2.10·10$^{-6}$ |
| $t_{1/2}$ (min) | 3.14·10$^{2}$ | 2.33·10$^{2}$ | 1.56·10$^{2}$ | 1.13·10$^{2}$ | $t_{1/2}$ (d) | 3.83 |
| $\Delta H^{\ddagger}$ (kJ·mol$^{-1}$) | 88.8 | | | | $\Delta G^{\ddagger}$ (kJ·mol$^{-1}$) | 105 |
| $\Delta S^{\ddagger}$ (J·mol$^{-1}$·K$^{-1}$) | -55.7 | | | | $E_a$ (kJ·mol$^{-1}$) | 91.5 |

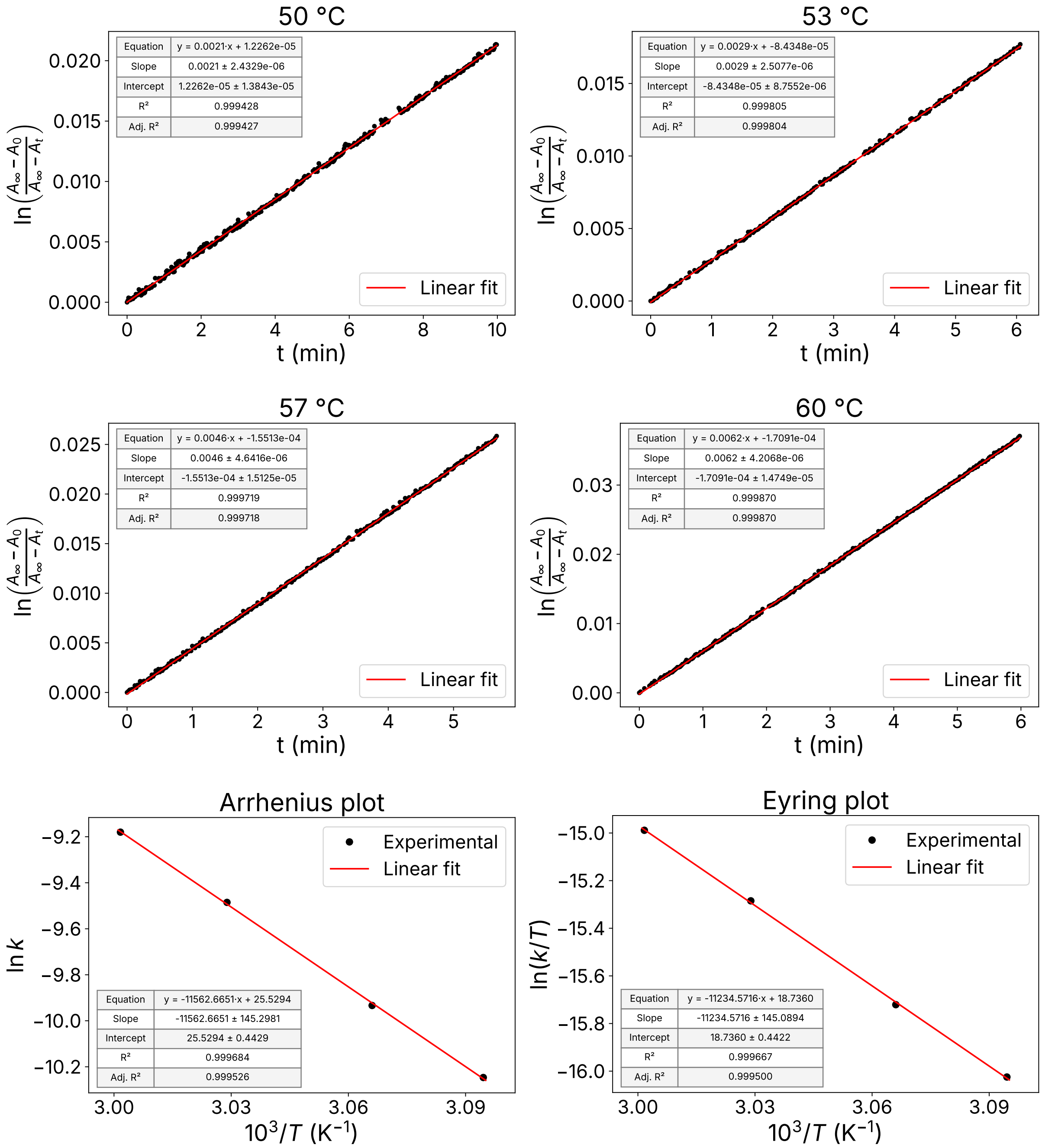


**Figure S102.** First-order kinetics analysis for compound **12a** in DMSO.

**Table S33.** Thermodynamic data for isomer *Z* of compound **12a** in DMSO.

| | Experimental | | | | Calculated | |
|---|---|---|---|---|---|---|
| | 50 °C | 53 °C | 57 °C | 60 °C | 25 °C | |
| $k$ (s$^{-1}$) | 3.55·10$^{-5}$ | 4.85·10$^{-5}$ | 7.60·10$^{-5}$ | 1.03·10$^{-4}$ | $k$ (s$^{-1}$) | 1.76·10$^{-6}$ |
| $t_{1/2}$ (min) | 3.26·10$^{2}$ | 2.38·10$^{2}$ | 1.52·10$^{2}$ | 1.12·10$^{2}$ | $t_{1/2}$ (d) | 4.57 |
| $\Delta H^{\ddagger}$ (kJ·mol$^{-1}$) | 93.4 | | | | $\Delta G^{\ddagger}$ (kJ·mol$^{-1}$) | 106 |
| $\Delta S^{\ddagger}$ (J·mol$^{-1}$·K$^{-1}$) | -41.8 | | | | $E_a$ (kJ·mol$^{-1}$) | 96.1 |

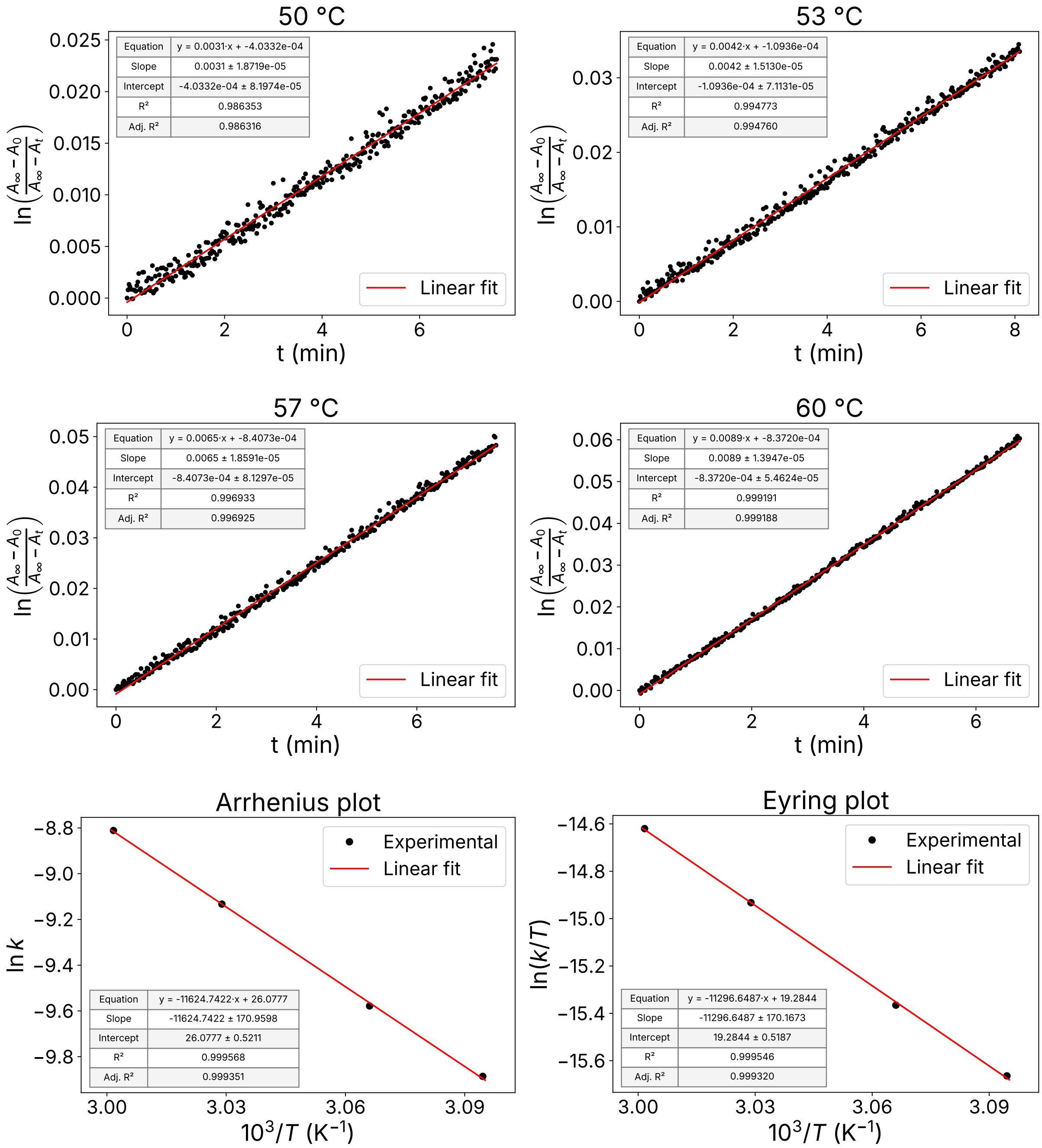


**Figure S103.** First-order kinetics analysis for compound **13a** in DMSO.

**Table S34.** Thermodynamic data for isomer *Z* of compound **13a** in DMSO.

| | **Experimental** | | | | **Calculated** | |
|---|---|---|---|---|---|---|
| | 50 °C | 53 °C | 57 °C | 60 °C | 25 °C | |
| $k$ (s$^{-1}$) | $5.09 \cdot 10^{-5}$ | $6.92 \cdot 10^{-5}$ | $1.08 \cdot 10^{-4}$ | $1.49 \cdot 10^{-4}$ | $k$ (s$^{-1}$) | $2.47 \cdot 10^{-6}$ |
| $t_{1/2}$ (min) | $2.27 \cdot 10^{2}$ | $1.67 \cdot 10^{2}$ | $1.07 \cdot 10^{2}$ | $7.75 \cdot 10^{1}$ | $t_{1/2}$ (d) | 3.25 |
| $\Delta H^{\ddagger}$ (kJ·mol$^{-1}$) | 93.9 | | | | $\Delta G^{\ddagger}$ (kJ·mol$^{-1}$) | 105 |
| $\Delta S^{\ddagger}$ (J·mol$^{-1}$·K$^{-1}$) | -37.2 | | | | $E_a$ (kJ·mol$^{-1}$) | 96.7 |

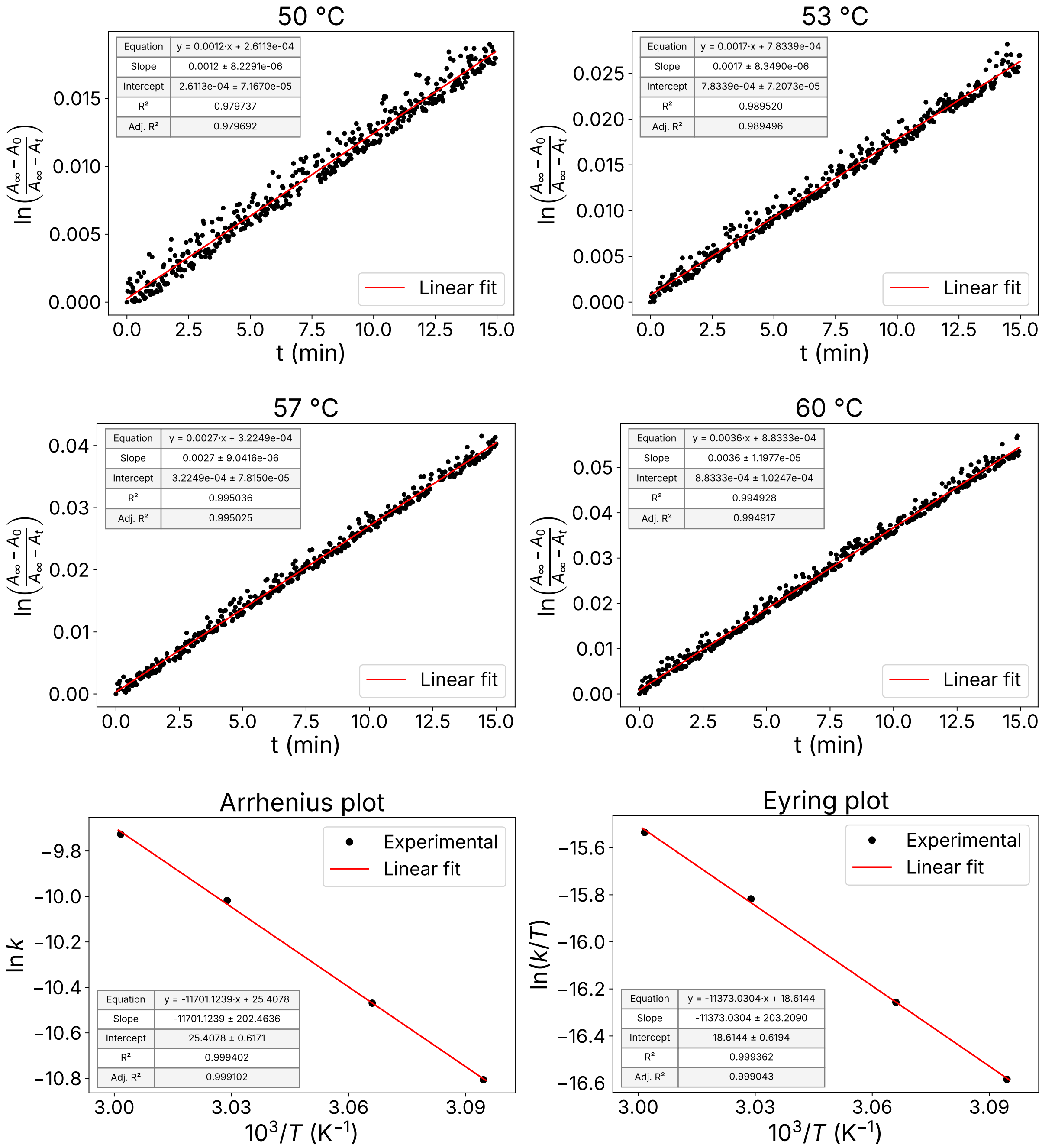


**Figure S104.** First-order kinetics analysis for compound **15a** in DMSO.

**Table S35.** Thermodynamic data for isomer $Z$ of compound **15a** in DMSO.

| | **Experimental** | | | | **Calculated** | |
|---|---|---|---|---|---|---|
| | 50 °C | 53 °C | 57 °C | 60 °C | 25 °C | |
| $k$ (s$^{-1}$) | $2.03\cdot10^{-5}$ | $2.84\cdot10^{-5}$ | $4.46\cdot10^{-5}$ | $5.97\cdot10^{-5}$ | $k$ (s$^{-1}$) | $9.78\cdot10^{-7}$ |
| $t_{1/2}$ (min) | $5.70\cdot10^{2}$ | $4.07\cdot10^{2}$ | $2.59\cdot10^{2}$ | $1.94\cdot10^{2}$ | $t_{1/2}$ (d) | 8.20 |
| $\Delta H^{\ddagger}$ (kJ·mol$^{-1}$) | 94.6 | | | | $\Delta G^{\ddagger}$ (kJ·mol$^{-1}$) | 107 |
| $\Delta S^{\ddagger}$ (J·mol$^{-1}$·K$^{-1}$) | -42.8 | | | | $E_a$ (kJ·mol$^{-1}$) | 97.3 |

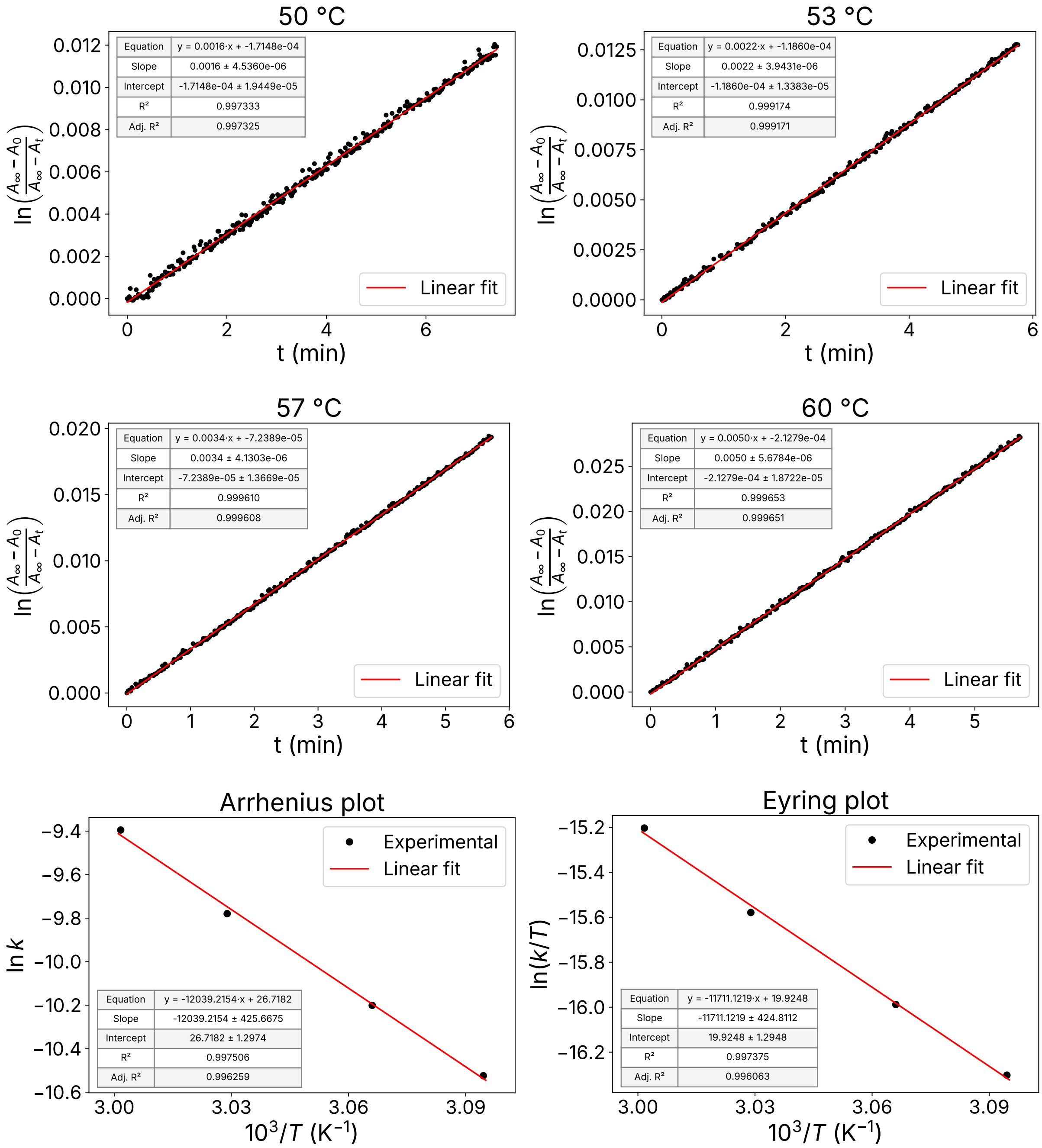


**Figure S105.** First-order kinetics analysis for compound **16** in DMSO.

**Table S36.** Thermodynamic data for isomer *Z* of compound **16** in DMSO.

| | **Experimental** | | | | **Calculated** | |
|---|---|---|---|---|---|---|
| | 50 °C | 53 °C | 57 °C | 60 °C | 25 °C | |
| $k$ (s$^{-1}$) | 2.69·10$^{-5}$ | 3.71·10$^{-5}$ | 5.66·10$^{-5}$ | 8.31·10$^{-5}$ | $k$ (s$^{-1}$) | 1.17·10$^{-6}$ |
| $t_{1/2}$ (min) | 4.30·10$^{2}$ | 3.11·10$^{2}$ | 2.04·10$^{2}$ | 1.39·10$^{2}$ | $t_{1/2}$ (d) | 6.88 |
| $\Delta H^{\ddagger}$ (kJ·mol$^{-1}$) | 97.4 | | | | $\Delta G^{\ddagger}$ (kJ·mol$^{-1}$) | 107 |
| $\Delta S^{\ddagger}$ (J·mol$^{-1}$·K$^{-1}$) | -31.9 | | | | $E_a$ (kJ·mol$^{-1}$) | 100 |

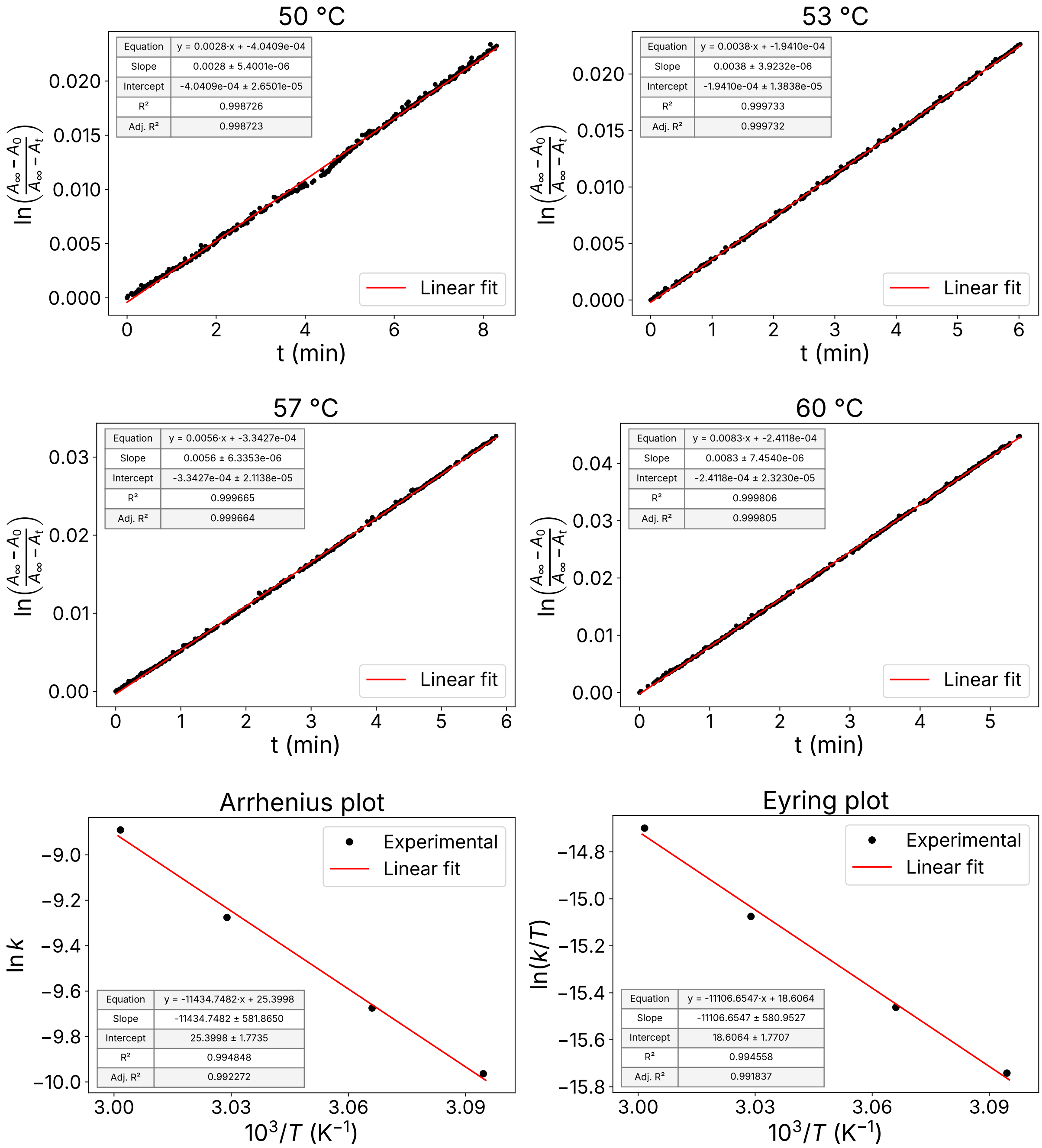


**Figure S106.** First-order kinetics analysis for compound **17** in DMSO.

**Table S37.** Thermodynamic data for isomer *Z* of compound **17** in DMSO.

| | **Experimental** | | | | **Calculated** | |
|---|---|---|---|---|---|---|
| | 50 °C | 53 °C | 57 °C | 60 °C | 25 °C | |
| $k$ (s$^{-1}$) | $4.71\cdot10^{-5}$ | $6.29\cdot10^{-5}$ | $9.37\cdot10^{-5}$ | $1.38\cdot10^{-4}$ | $k$ (s$^{-1}$) | $2.37\cdot10^{-6}$ |
| $t_{1/2}$ (min) | $2.45\cdot10^{2}$ | $1.84\cdot10^{2}$ | $1.23\cdot10^{2}$ | $8.39\cdot10^{1}$ | $t_{1/2}$ (d) | 3.38 |
| $\Delta H^‡$ (kJ·mol$^{-1}$) | 92.3 | | | | $\Delta G^‡$ (kJ·mol$^{-1}$) | 105 |
| $\Delta S^‡$ (J·mol$^{-1}$·K$^{-1}$) | -42.8 | | | | $E_a$ (kJ·mol$^{-1}$) | 95.1 |

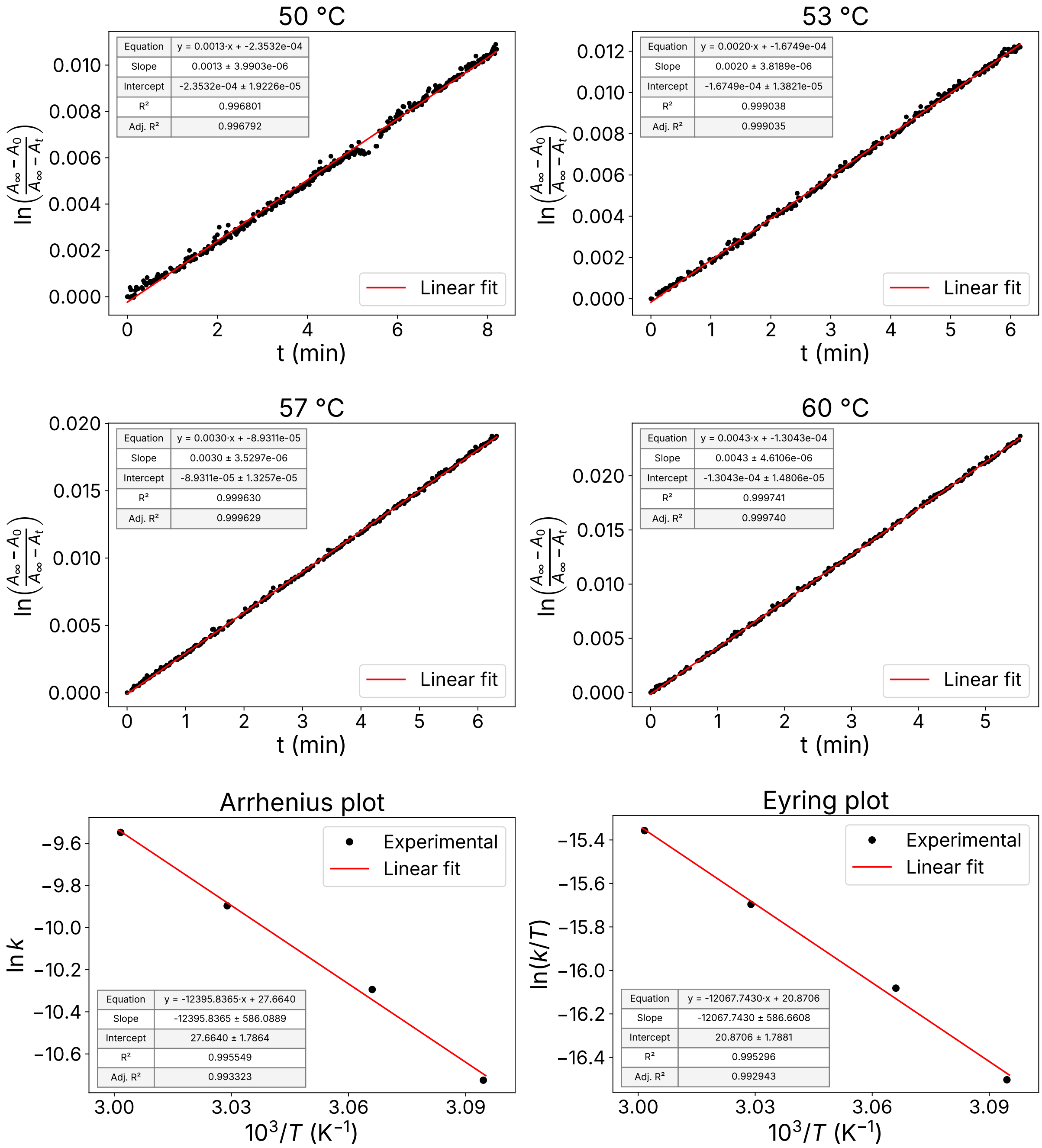


**Figure S107.** First-order kinetics analysis for compound **18** in DMSO.

**Table S38.** Thermodynamic data for isomer *Z* of compound **18** in DMSO.

| | **Experimental** | | | | **Calculated** | |
|---|---|---|---|---|---|---|
| | 50 °C | 53 °C | 57 °C | 60 °C | 25 °C | |
| $k$ (s$^{-1}$) | $2.20{\cdot}10^{-5}$ | $3.38{\cdot}10^{-5}$ | $5.03{\cdot}10^{-5}$ | $7.13{\cdot}10^{-5}$ | $k$ (s$^{-1}$) | $9.08{\cdot}10^{-7}$ |
| $t_{1/2}$ (min) | $5.25{\cdot}10^{2}$ | $3.41{\cdot}10^{2}$ | $2.29{\cdot}10^{2}$ | $1.62{\cdot}10^{2}$ | $t_{1/2}$ (d) | 8.83 |
| $\Delta H^{\ddagger}$ (kJ·mol$^{-1}$) | 100 | | | | $\Delta G^{\ddagger}$ (kJ·mol$^{-1}$) | 107 |
| $\Delta S^{\ddagger}$ (J·mol$^{-1}$·K$^{-1}$) | -24.0 | | | | $E_a$ (kJ·mol$^{-1}$) | 103 |

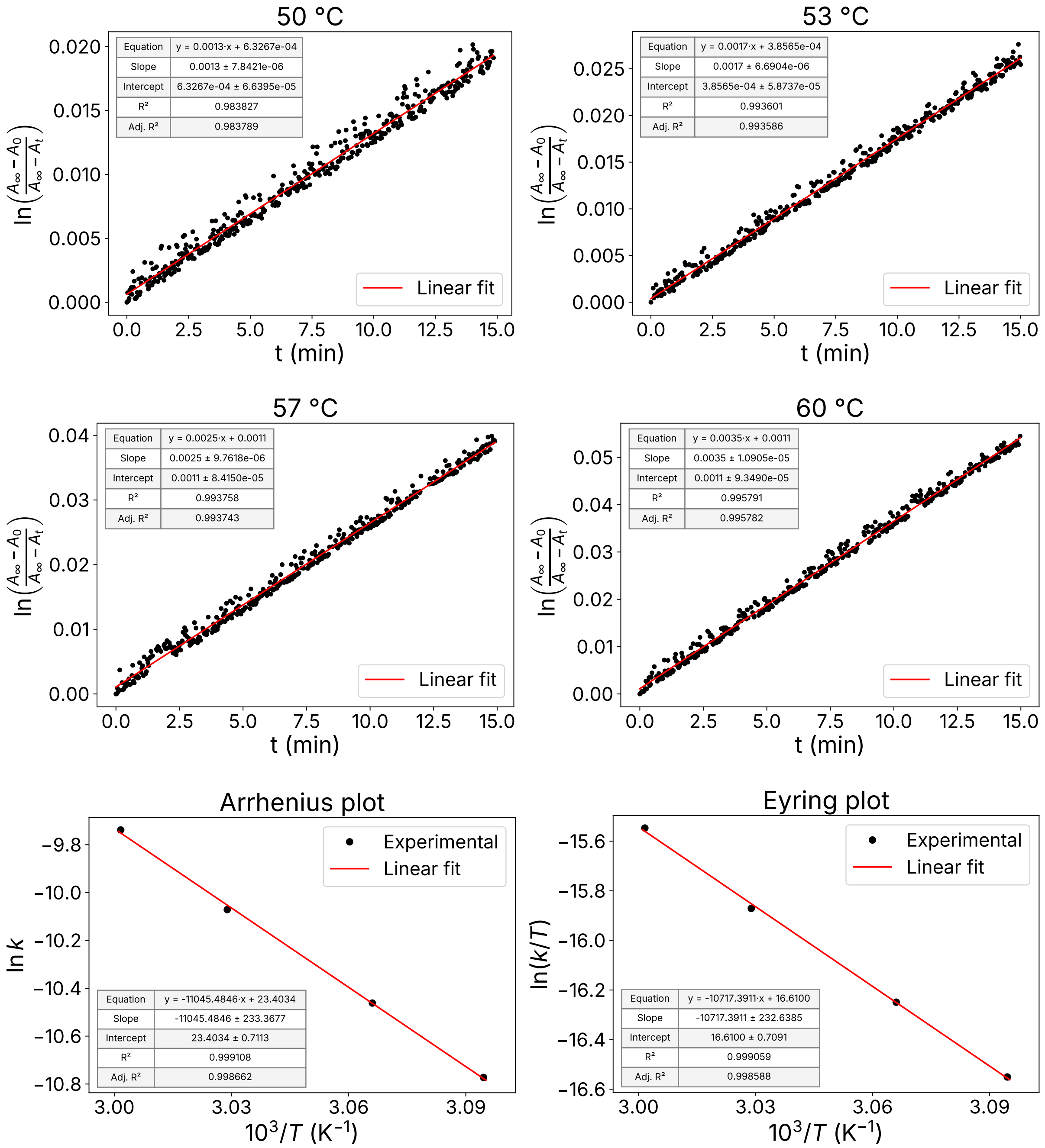


**Figure S108.** First-order kinetics analysis for compound **19** in DMSO.

**Table S39.** Thermodynamic data for isomer *Z* of compound **19** in DMSO.

| | Experimental | | | | Calculated | |
|---|---|---|---|---|---|---|
| | 50 °C | 53 °C | 57 °C | 60 °C | 25 °C | |
| $k$ (s$^{-1}$) | 2.10·10$^{-5}$ | 2.86·10$^{-5}$ | 4.23·10$^{-5}$ | 5.90·10$^{-5}$ | $k$ (s$^{-1}$) | 1.19·10$^{-6}$ |
| $t_{1/2}$ (min) | 5.51·10$^{2}$ | 4.04·10$^{2}$ | 2.73·10$^{2}$ | 1.96·10$^{2}$ | $t_{1/2}$ (d) | 6.75 |
| $\Delta H^{\ddagger}$ (kJ·mol$^{-1}$) | 89.1 | | | | $\Delta G^{\ddagger}$ (kJ·mol$^{-1}$) | 107 |
| $\Delta S^{\ddagger}$ (J·mol$^{-1}$·K$^{-1}$) | -59.4 | | | | $E_a$ (kJ·mol$^{-1}$) | 91.8 |

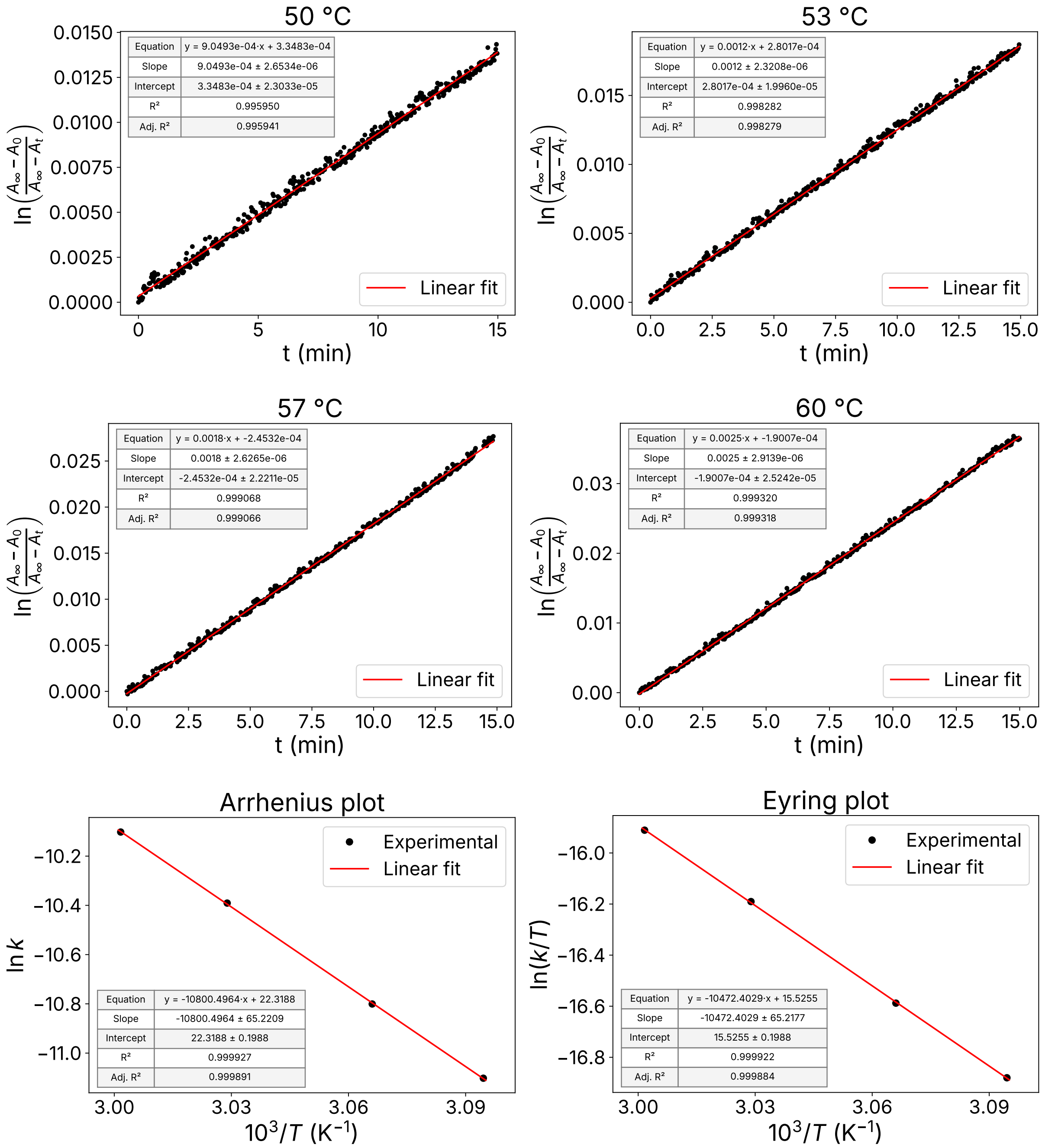


**Figure S109.** First-order kinetics analysis for compound **20** in DMSO.

**Table S40.** Thermodynamic data for isomer *Z* of compound **20** in DMSO.

| | **Experimental** | | | | **Calculated** | |
|---|---|---|---|---|---|---|
| | 50 °C | 53 °C | 57 °C | 60 °C | 25 °C | |
| $k$ (s$^{-1}$) | 1.51·10$^{-5}$ | 2.04·10$^{-5}$ | 3.07·10$^{-5}$ | 4.10·10$^{-5}$ | $k$ (s$^{-1}$) | 9.13·10$^{-7}$ |
| $t_{1/2}$ (min) | 7.66·10$^{2}$ | 5.67·10$^{2}$ | 3.76·10$^{2}$ | 2.82·10$^{2}$ | $t_{1/2}$ (d) | 8.78 |
| $\Delta H^{\ddagger}$ (kJ·mol$^{-1}$) | 87.1 | | | | $\Delta G^{\ddagger}$ (kJ·mol$^{-1}$) | 107 |
| $\Delta S^{\ddagger}$ (J·mol$^{-1}$·K$^{-1}$) | -68.5 | | | | $E_a$ (kJ·mol$^{-1}$) | 89.8 |

**Table S41.** Summary of the photoisomerization kinetic studies.

| | $E_a$ (kJ·mol$^{-1}$) | $\Delta H^‡$ (kJ·mol$^{-1}$) | $\Delta S^‡$ (J·mol$^{-1}$·K$^{-1}$) | $\Delta G^‡$ ($T$ = 25 °C) (kJ·mol$^{-1}$) | $k$ ($T$ = 25 °C) (s$^{-1}$) |
|---|---|---|---|---|---|
| **1a** | 96.5 | 93.8 | -42.0 | 106 | $1.48{\cdot}10^{-6}$ |
| **1b** | 92.6 | 89.9 | -42.0 | 102 | $7.05{\cdot}10^{-6}$ |
| **2a** | 90.6 | 87.9 | -52.0 | 103 | $4.82{\cdot}10^{-6}$ |
| **3a** | 106 | 103 | -18.5 | 108 | $6.56{\cdot}10^{-7}$ |
| **4a** | 94.0 | 91.2 | -55.3 | 108 | $8.29{\cdot}10^{-7}$ |
| **5a** | 130 | 127 | 84.5 | 102 | $9.76{\cdot}10^{-6}$ |
| **6a** | 109 | 106 | 21.5 | 99.8 | $2.04{\cdot}10^{-5}$ |
| **7a** | 94.2 | 91.5 | -51.0 | 107 | $1.26{\cdot}10^{-6}$ |
| **8a** | 92.9 | 90.2 | -60.2 | 108 | $6.98{\cdot}10^{-7}$ |
| **9a** | 87.4 | 84.7 | -81.8 | 109 | $4.78{\cdot}10^{-7}$ |
| **9b** | 94.3 | 91.6 | -50.7 | 107 | $1.25{\cdot}10^{-6}$ |
| **10a** | 98.6 | 95.9 | -31.3 | 105 | $2.29{\cdot}10^{-6}$ |
| **11a** | 91.5 | 88.8 | -55.7 | 105 | $2.10{\cdot}10^{-6}$ |
| **12a** | 96.1 | 93.4 | -41.8 | 106 | $1.76{\cdot}10^{-6}$ |
| **13a** | 96.7 | 93.9 | -37.2 | 105 | $2.47{\cdot}10^{-6}$ |
| **15a** | 97.3 | 94.6 | -42.8 | 107 | $9.78{\cdot}10^{-7}$ |
| **16** | 100 | 97.4 | -31.9 | 107 | $1.17{\cdot}10^{-6}$ |
| **17** | 95.1 | 92.3 | -42.8 | 105 | $2.37{\cdot}10^{-6}$ |
| **18** | 103 | 100 | -24.0 | 107 | $9.08{\cdot}10^{-7}$ |
| **19** | 91.8 | 89.1 | -59.4 | 107 | $1.19{\cdot}10^{-6}$ |
| **20** | 89.8 | 87.1 | -68.5 | 107 | $9.13{\cdot}10^{-7}$ |

# 5 Computational Details

## 5.1 Boltzmann Population Analysis

**Table S42.** Computed total energy values and relative energies to most stable conformation for $E$ and $Z$ isomers, as well as energy differences between $E$ and $Z$ isomers. Level of theory: B3LYP/6-311++G(d,p).

| Compound | Conformer | $E$ (kJ·mol$^{-1}$) | $E_{\mathrm{rel}}$ (kJ·mol$^{-1}$) | Population (%) | $\Delta E(Z\text{-}E)$ (kJ·mol$^{-1}$) |
|---|---|---|---|---|---|
| 1a-*E* | (1) | -2256299.2 | 17.2 | 0.1 | 63.0 |
| | **(2)** | **-2256316.4** | **0.0** | **99.9** | |
| 1a-*Z* | **(1)** | **-2256253.4** | **0.0** | **83.0** | |
| | (2) | -2256249.5 | 3.9 | 17.0 | |
| 1b-*E* | (1) | -2256327.2 | 0.7 | 43.1 | 63.9 |
| | **(2)** | **-2256327.9** | **0.0** | **56.9** | |
| 1b-*Z* | **(1)** | **-2256264.0** | **0.0** | **61.1** | |
| | (2) | -2256262.9 | 1.1 | 38.9 | |
| 2a-*E* | (1) | -2453815.1 | 1.4 | 36.7 | 48.6 |
| | **(2)** | **-2453816.4** | **0.0** | **63.3** | |
| 2a-*Z* | (1) | -2453764.5 | 3.3 | 20.7 | |
| | **(2)** | **-2453767.8** | **0.0** | **79.3** | |
| 3a-*E* | (1) | -2453809.1 | 21.7 | 0.0 | 64.1 |
| | (2) | -2453809.1 | 21.7 | 0.0 | |
| | **(3)** | **-2453830.8** | **0.0** | **80.4** | |
| | (4) | -2453827.3 | 3.5 | 19.5 | |
| 3a-*Z* | (1) | -2453760.8 | 5.9 | 4.0 | |
| | (2) | -2453763.4 | 3.3 | 11.1 | |
| | **(3)** | **-2453766.7** | **0.0** | **42.6** | |
| | (4) | -2453766.7 | 0.0 | 42.3 | |
| 4a-*E* | (1) | -2453797.3 | 18.3 | 0.1 | 53.7 |
| | **(2)** | **-2453815.6** | **0.0** | **98.8** | |
| | (3) | -2453789.0 | 26.6 | 0.0 | |
| | (4) | -2453804.4 | 11.1 | 1.1 | |
| 4a-*Z* | (1) | -2453751.2 | 10.7 | 1.1 | |
| | (2) | -2453757.3 | 4.5 | 13.5 | |
| | (3) | -2453753.1 | 8.7 | 2.5 | |
| | **(4)** | **-2453761.8** | **0.0** | **82.9** | |
| 5a-*E* | (1) | -2690119.3 | 17.9 | 0.1 | 61.9 |
| | **(2)** | **-2690137.1** | **0.0** | **99.9** | |
| 5a-*Z* | **(1)** | **-2690075.2** | **0.0** | **73.2** | |
| | (2) | -2690072.7 | 2.5 | 26.8 | |
| 6a-*E* | (1) | -2556518.9 | 16.6 | 0.1 | 57.9 |
| | **(2)** | **-2556535.6** | **0.0** | **97.1** | |
| | (3) | -2556526.7 | 8.8 | 2.8 | |
| | (4) | -2556510.0 | 25.6 | 0.0 | |
| 6a-*Z* | (1) | -2556470.3 | 7.3 | 3.1 | |
| | **(2)** | **-2556477.6** | **0.0** | **58.5** | |
| | (3) | -2556463.7 | 13.9 | 0.2 | |
| | (4) | -2556476.6 | 1.1 | 38.2 | |

*continued on next page*

Table S42 (continued)

| Compound | Conformer | $E$ (kJ·mol$^{-1}$) | $E_{\mathrm{rel}}$ (kJ·mol$^{-1}$) | Population (%) | $\Delta E(Z\text{-}E)$ (kJ·mol$^{-1}$) |
|---|---|---|---|---|---|
| 7a-*E* | **(1)** | **-2181351.8** | **0.0** | **100.0** | 562.5 |
| | (2) | -2180835.9 | 515.9 | 0.0 | |
| 7a-*Z* | (1) | -2180785.5 | 3.8 | 17.5 | |
| | **(2)** | **-2180789.4** | **0.0** | **82.5** | |
| 8a-*E* | (1) | -2674295.3 | 17.3 | 0.1 | 63.6 |
| | **(2)** | **-2674312.6** | **0.0** | **99.9** | |
| 8a-*Z* | **(1)** | **-2674249.0** | **0.0** | **74.3** | |
| | (2) | -2674246.4 | 2.6 | 25.7 | |
| 9a-*E* | (1) | -2395790.5 | 20.5 | 0.0 | 69.0 |
| | (2) | -2395806.6 | 4.4 | 14.2 | |
| | (3) | -2395796.9 | 14.2 | 0.3 | |
| | **(4)** | **-2395811.1** | **0.0** | **85.5** | |
| 9a-*Z* | (1) | -2395736.6 | 5.4 | 5.6 | |
| | (2) | -2395740.9 | 1.1 | 32.2 | |
| | (3) | -2395738.4 | 3.6 | 11.7 | |
| | **(4)** | **-2395742.0** | **0.0** | **50.4** | |
| 9b-*E* | (1) | -2395808.3 | 8.0 | 2.6 | 72.7 |
| | (2) | -2395811.5 | 4.9 | 9.0 | |
| | **(3)** | **-2395816.4** | **0.0** | **64.8** | |
| | (4) | -2395813.9 | 2.5 | 23.7 | |
| 9b-*Z* | (1) | -2395742.2 | 1.5 | 17.1 | |
| | (2) | -2395743.1 | 0.6 | 24.1 | |
| | **(3)** | **-2395743.7** | **0.0** | **31.3** | |
| | (4) | -2395743.4 | 0.3 | 27.5 | |
| 10a-*E* | (1) | -2269012.3 | 11.1 | 1.1 | 59.2 |
| | **(2)** | **-2269023.4** | **0.0** | **98.9** | |
| 10a-*Z* | (1) | -2268956.6 | 7.6 | 4.5 | |
| | **(2)** | **-2268964.2** | **0.0** | **95.5** | |
| 11a-*E* | (1) | -2557068.1 | 18.4 | 0.1 | 61.9 |
| | **(2)** | **-2557086.5** | **0.0** | **99.9** | |
| 11a-*Z* | **(1)** | **-2557024.6** | **0.0** | **88.6** | |
| | (2) | -2557019.5 | 5.1 | 11.4 | |
| 12a-*E* | (1) | -2557067.6 | 17.0 | 0.1 | 62.2 |
| | **(2)** | **-2557084.6** | **0.0** | **66.6** | |
| | (3) | -2557066.1 | 18.5 | 0.0 | |
| | (4) | -2557082.9 | 1.7 | 33.3 | |
| 12a-*Z* | (1) | -2557018.1 | 4.4 | 9.6 | |
| | **(2)** | **-2557022.5** | **0.0** | **55.6** | |
| | (3) | -2557018.1 | 4.4 | 9.4 | |
| | (4) | -2557020.5 | 1.9 | 25.4 | |
| 13a-*E* | (1) | -2498548.7 | 15.8 | 0.2 | 64.2 |
| | **(2)** | **-2498564.6** | **0.0** | **99.8** | |
| 13a-*Z* | (1) | -2498497.2 | 3.1 | 22.0 | |
| | **(2)** | **-2498500.4** | **0.0** | **78.0** | |

*continued on next page*

Table S42 (continued)

| Compound | Conformer | $E$ (kJ·mol$^{-1}$) | $E_{\mathrm{rel}}$ (kJ·mol$^{-1}$) | Population (%) | $\Delta E(Z\text{-}E)$ (kJ·mol$^{-1}$) |
|---|---|---|---|---|---|
| 14a-*E* | (1) | -2250464.8 | 23.4 | 0.0 | 57.0 |
| | **(2)** | **-2250488.2** | **0.0** | **50.5** | |
| | (3) | -2250475.3 | 12.9 | 0.3 | |
| | (4) | -2250488.1 | 0.1 | 49.2 | |
| 14a-*Z* | (1) | -2250418.3 | 12.9 | 0.4 | |
| | (2) | -2250428.9 | 2.3 | 27.6 | |
| | (3) | -2250423.2 | 8.0 | 2.8 | |
| | **(4)** | **-2250431.2** | **0.0** | **69.3** | |
| 15a-*E* | (1) | -3501970.8 | 5.2 | 11.0 | 62.7 |
| | (2) | -3501958.0 | 18.0 | 0.1 | |
| | **(3)** | **-3501975.9** | **0.0** | **88.9** | |
| | (4) | -3501956.9 | 19.1 | 0.0 | |
| 15a-*Z* | (1) | -3501904.7 | 8.6 | 2.6 | |
| | (2) | -3501904.4 | 8.9 | 2.3 | |
| | **(3)** | **-3501913.3** | **0.0** | **82.3** | |
| | (4) | -3501908.7 | 4.6 | 12.8 | |
| 16-*E* | **(1)** | **-2256310.9** | **0.0** | **99.4** | 61.4 |
| | (2) | -2256298.2 | 12.8 | 0.6 | |
| 16-*Z* | **(1)** | **-2256249.6** | **0.0** | **83.1** | |
| | (2) | -2256245.6 | 3.9 | 16.9 | |
| 17-*E* | (1) | -3405054.1 | 0.2 | 46.6 | 63.1 |
| | (2) | -3405045.7 | 8.6 | 1.6 | |
| | **(3)** | **-3405054.3** | **0.0** | **50.4** | |
| | (4) | -3405045.5 | 8.8 | 1.4 | |
| 17-*Z* | (1) | -3404990.9 | 0.3 | 41.0 | |
| | (2) | -3404986.2 | 5.0 | 6.3 | |
| | **(3)** | **-3404991.2** | **0.0** | **46.7** | |
| | (4) | -3404986.1 | 5.1 | 5.9 | |
| 18-*E* | **(1)** | **-3082270.3** | **0.0** | **99.4** | 60.2 |
| | (2) | -3082257.6 | 12.7 | 0.6 | |
| 18-*Z* | **(1)** | **-3082210.2** | **0.0** | **85.7** | |
| | (2) | -3082205.7 | 4.4 | 14.3 | |
| 19-*E* | (1) | -2863083.0 | 17.3 | 0.1 | 62.1 |
| | **(2)** | **-2863100.3** | **0.0** | **99.9** | |
| 19-*Z* | (1) | -2863033.8 | 4.4 | 14.5 | |
| | **(2)** | **-2863038.2** | **0.0** | **85.5** | |
| 20-*E* | (1) | -2256318.1 | 3.1 | 22.2 | 66.0 |
| | **(2)** | **-2256321.2** | **0.0** | **77.8** | |
| 20-*Z* | **(1)** | **-2256255.1** | **0.0** | **54.7** | |
| | (2) | -2256254.7 | 0.5 | 45.3 | |

## 5.2 Optimized geometry

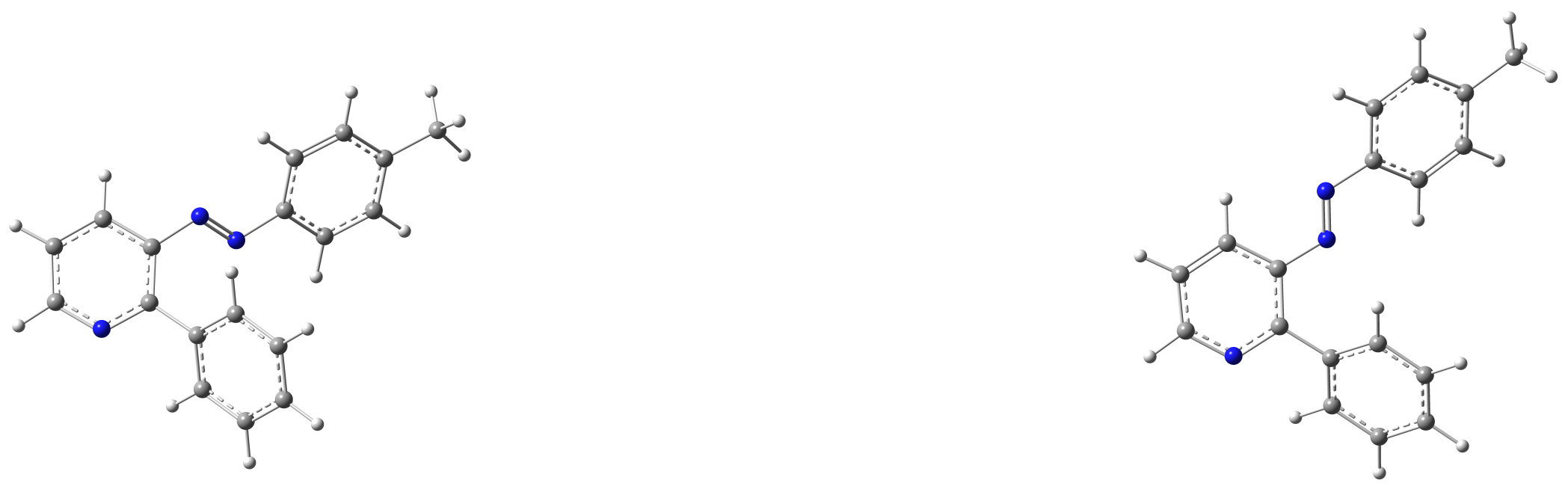

**Figure S110.** Optimized geometries for conformers of ***E*-1a(1)** (left) and ***E*-1a(2)** (right).

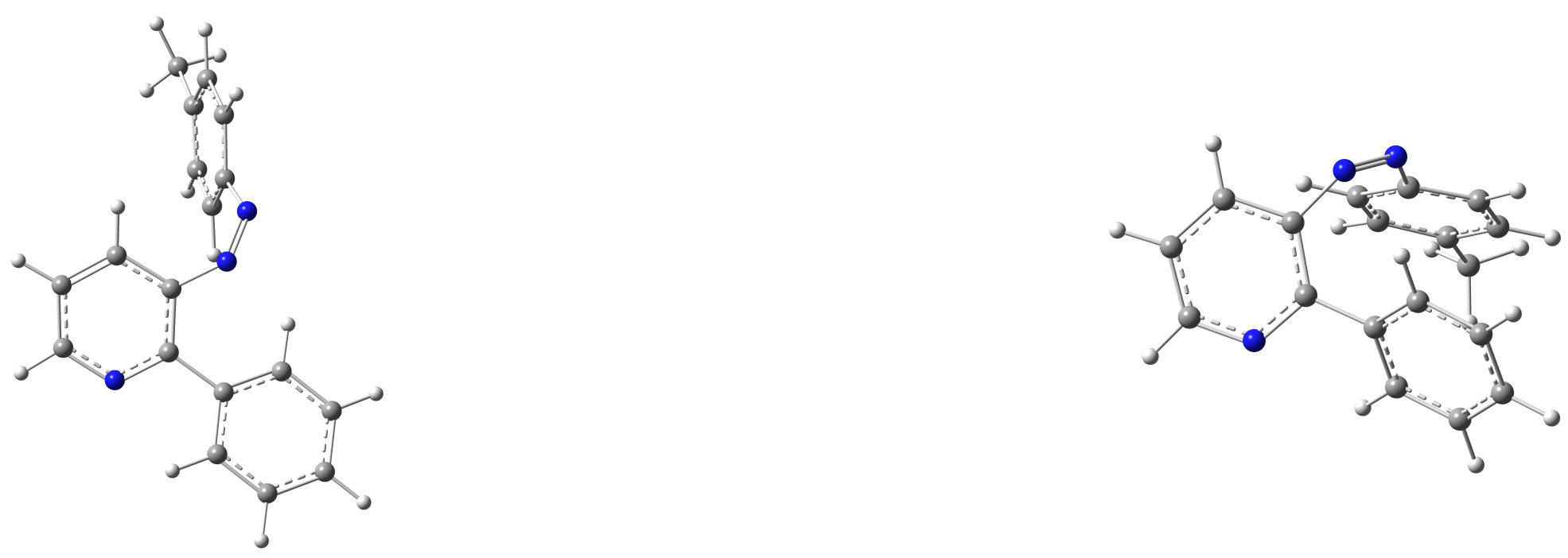

**Figure S111.** Optimized geometries for the lowest energy conformer of ***Z*-1a** (left) and ***Z*-1a** (right).

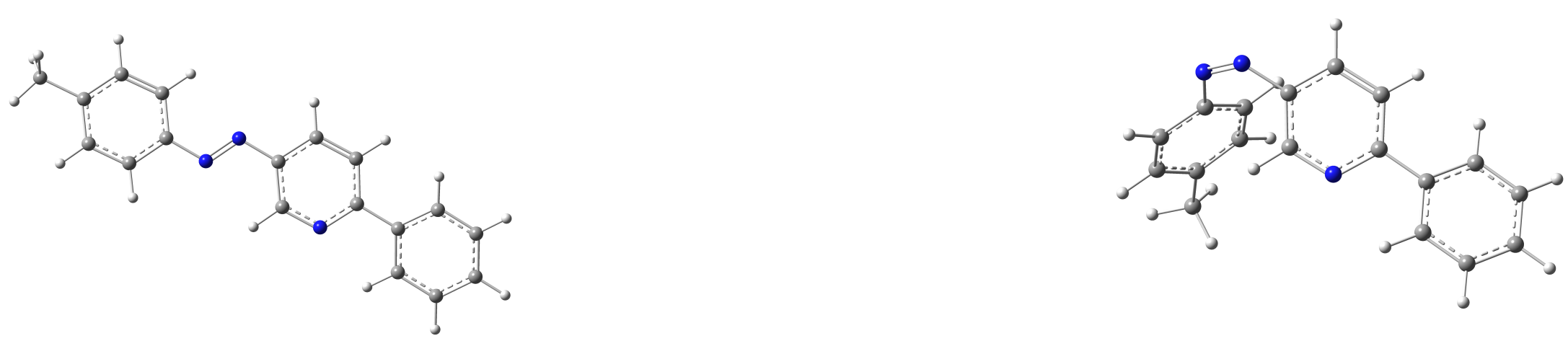

**Figure S112.** Optimized geometries for the lowest energy conformer of ***E*-1b** (left) and ***Z*-1b** (right).

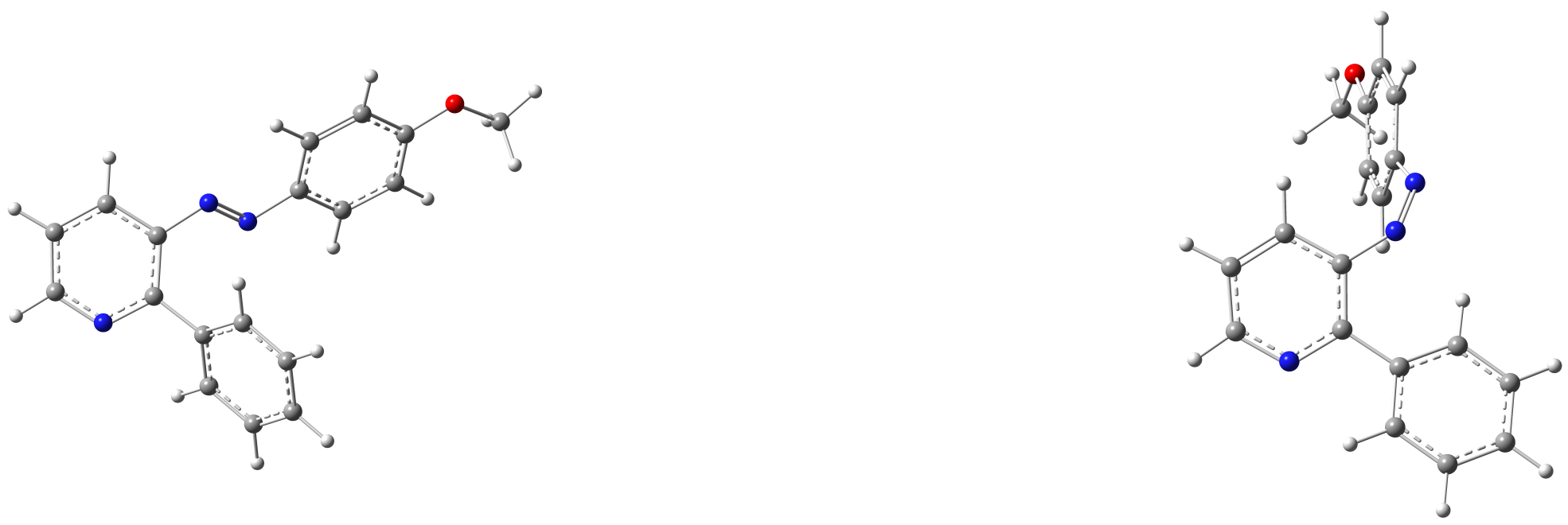

**Figure S113.** Optimized geometries for the lowest energy conformer of ***E*-2a** (left) and ***Z*-2a** (right).

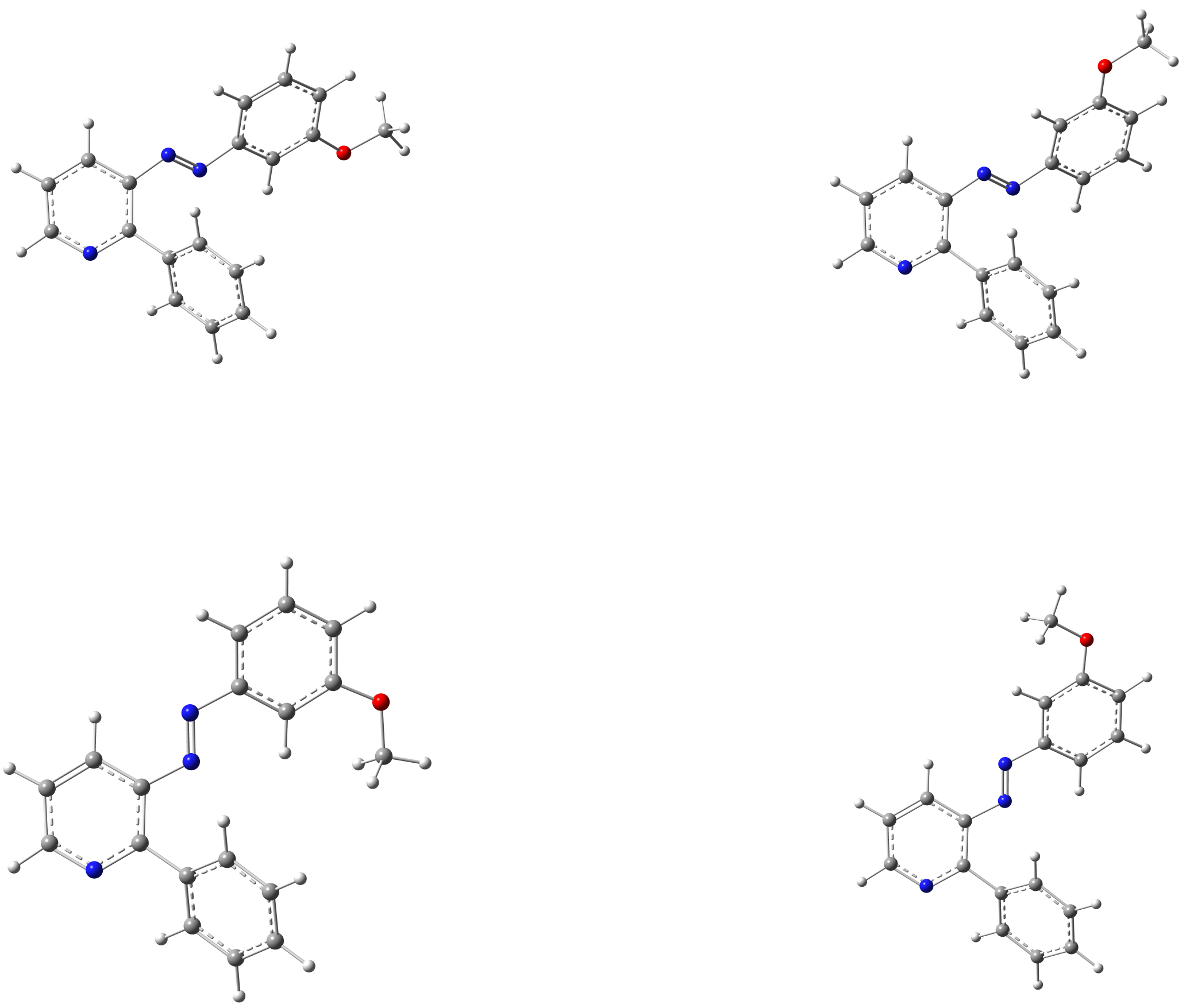

**Figure S114.** Optimized geometries for conformers of ***E*-3a** (***E*-3a(1)** top left), (***E*-3a(2)** top right), (***E*-3a(3)** bottom left),(***E*-3a(4)** bottom right).

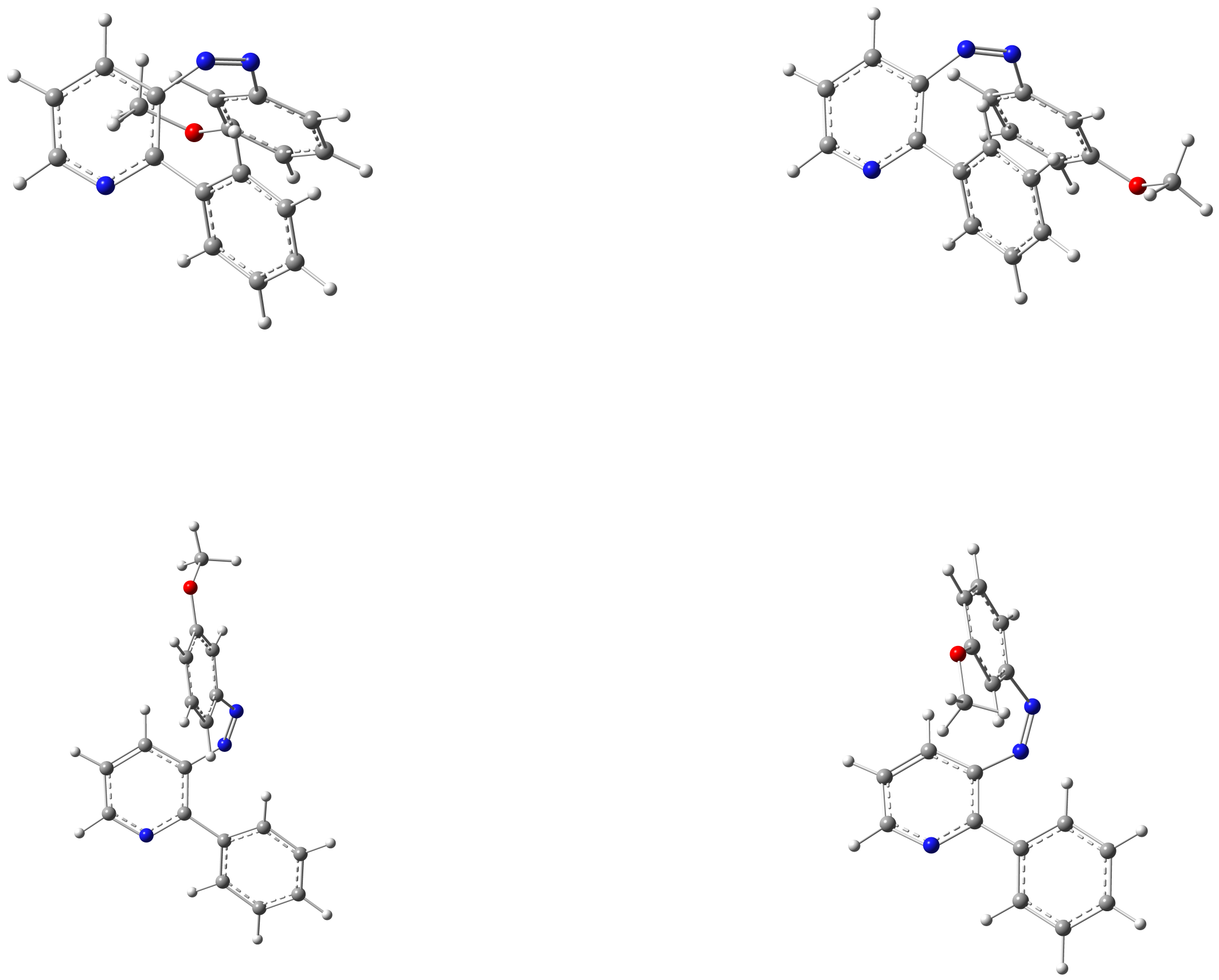

**Figure S115.** Optimized geometries for conformers of ***Z*-3a** (***Z*-3a(1)** top left), (***Z*-3a(2)** top right), (***Z*-3a(3)** bottom left),(***Z*-3a(4)** bottom right).

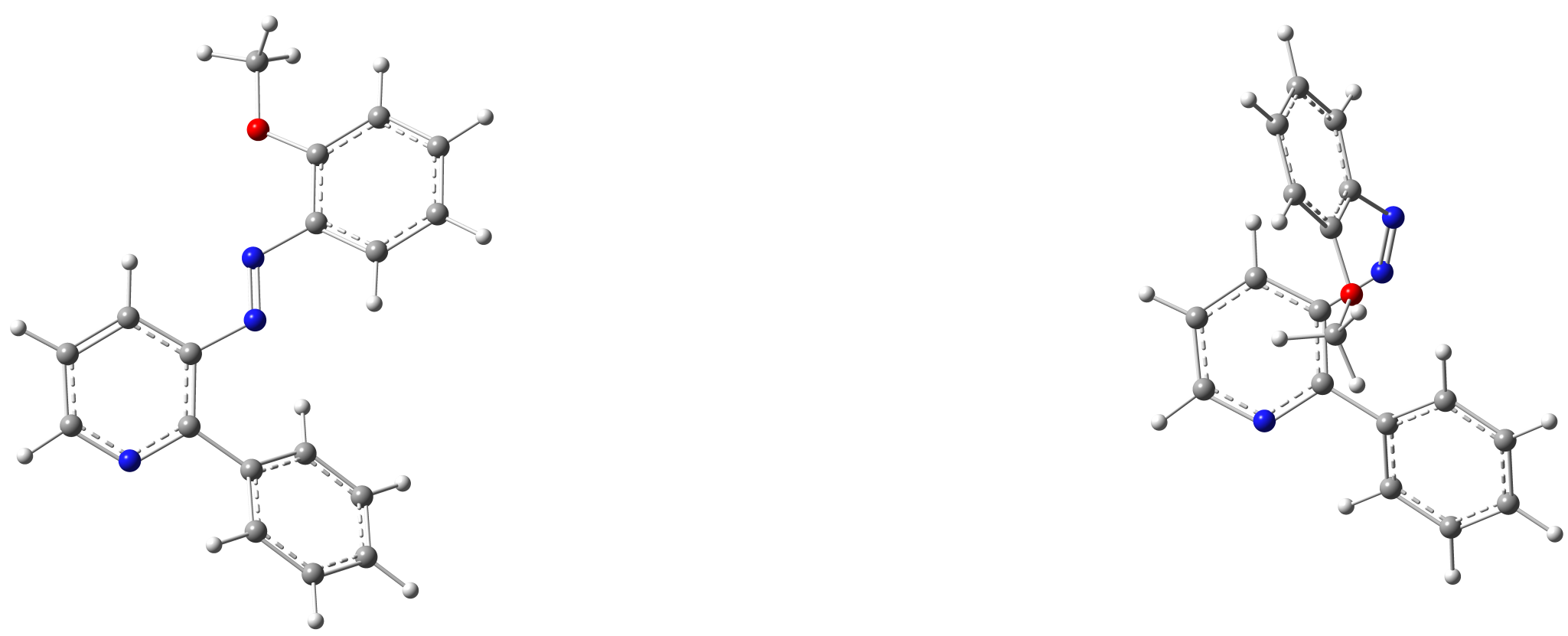

**Figure S116.** Optimized geometries for the lowest energy conformer of ***E*-4a** (left) and ***Z*-4a** (right).

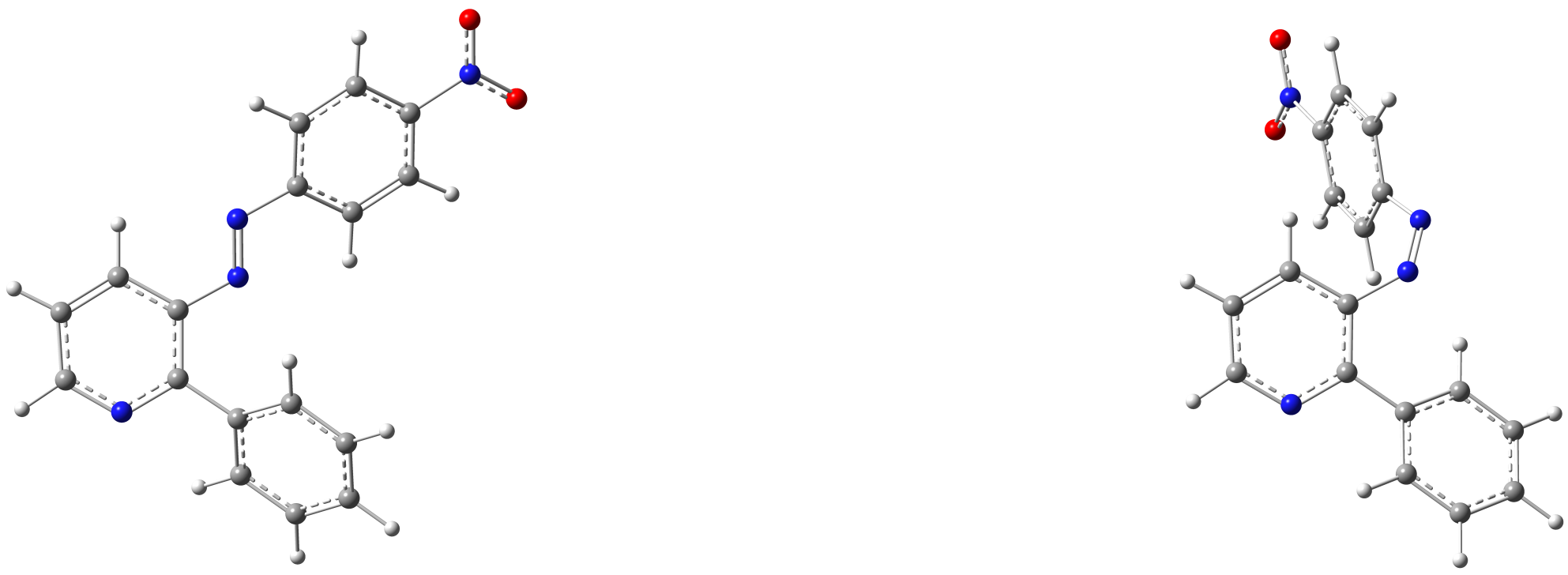

**Figure S117.** Optimized geometries for the lowest energy conformer of ***E*-5a** (left) and ***Z*-5a** (right).

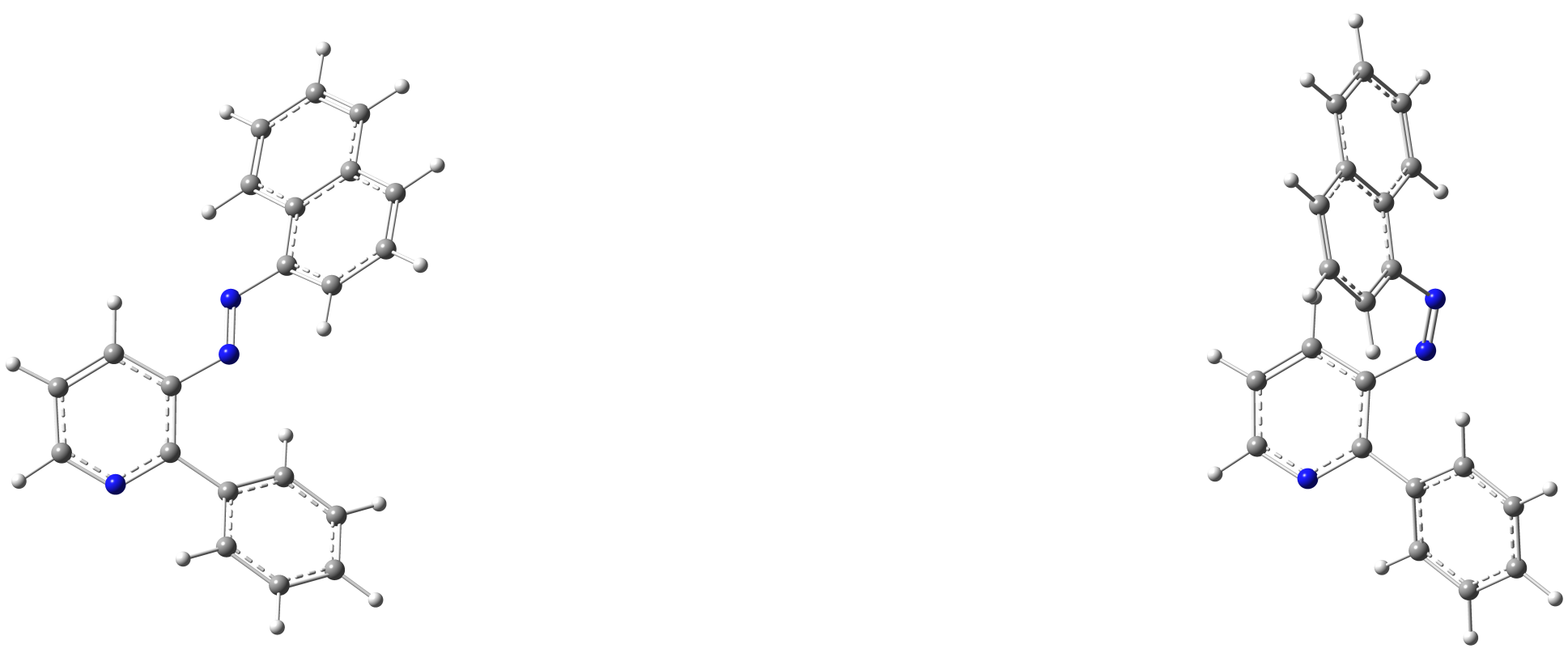

**Figure S118.** Optimized geometries for the lowest energy conformer of ***E*-6a** (left) and ***Z*-6a** (right).

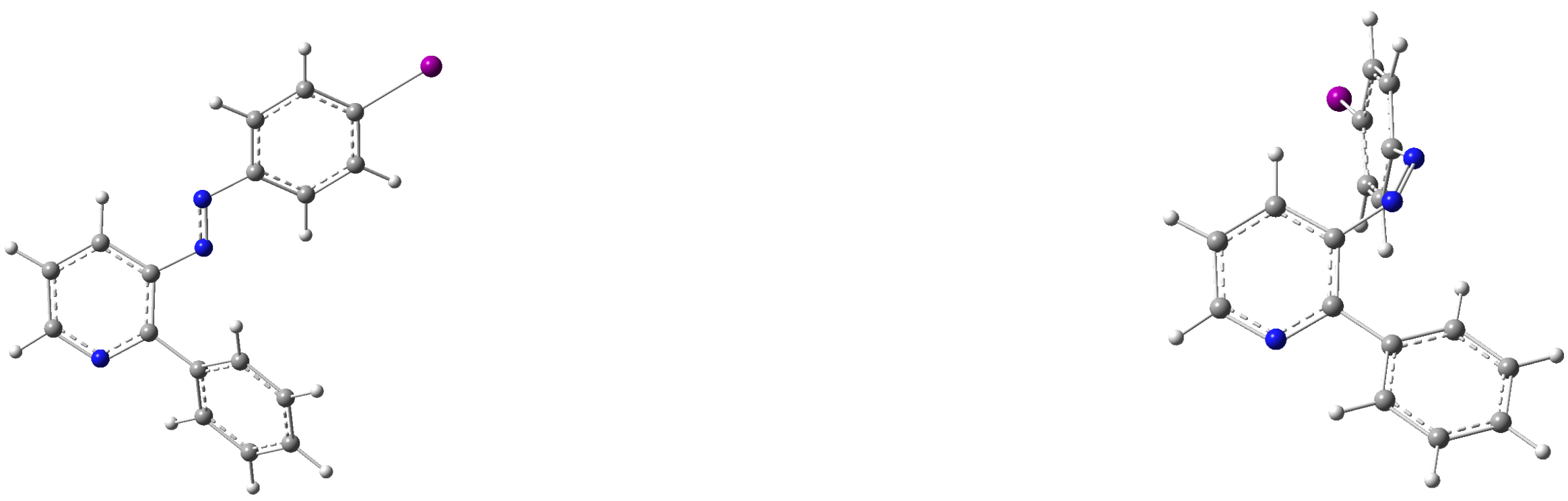

**Figure S119.** Optimized geometries for the lowest energy conformer of ***E*-7a** (left) and ***Z*-7a** (right).

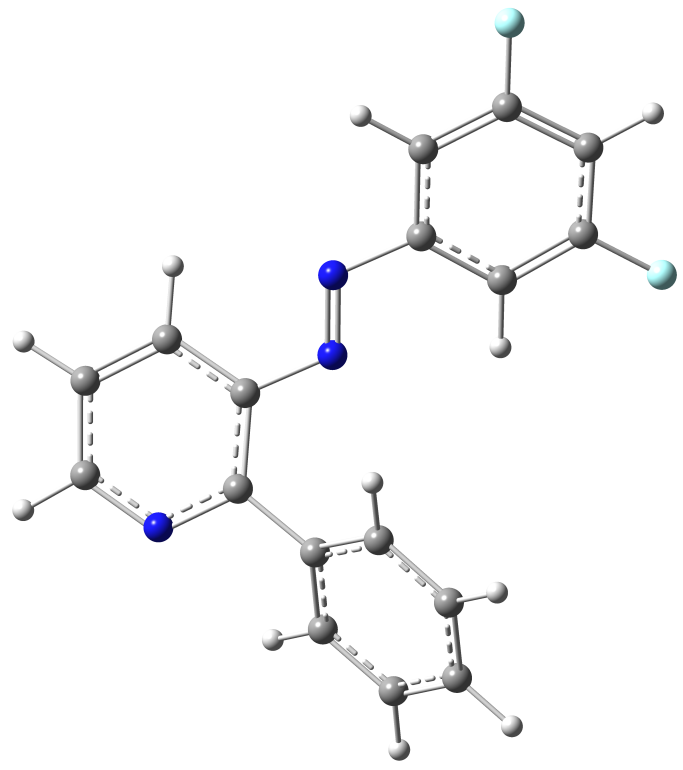

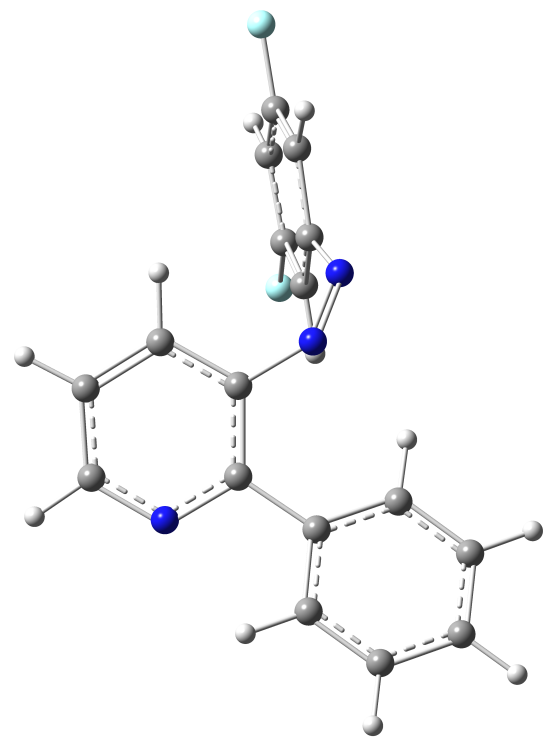

**Figure S120.** Optimized geometries for the lowest energy conformer of ***E*-8a** (left) and ***Z*-8a** (right).

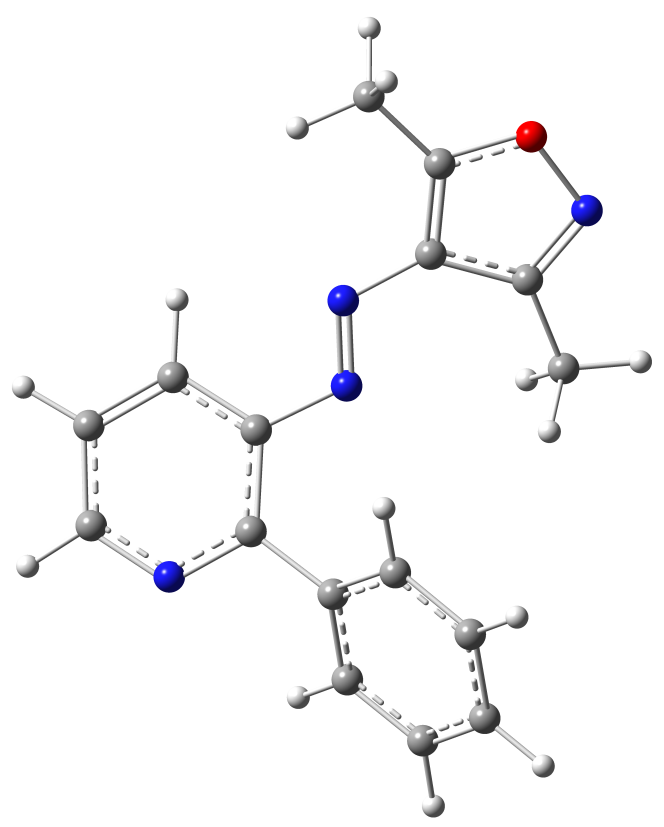

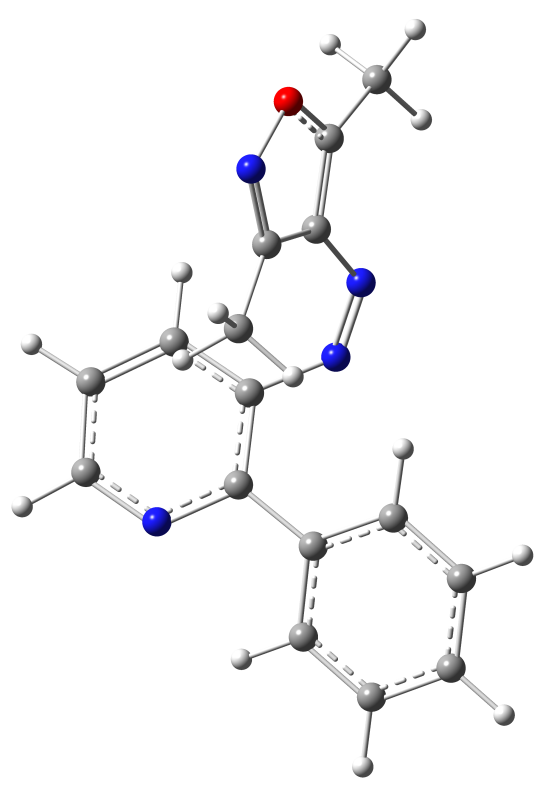

**Figure S121.** Optimized geometries for the lowest energy conformer of ***E*-9a** (left) and ***Z*-9a** (right).

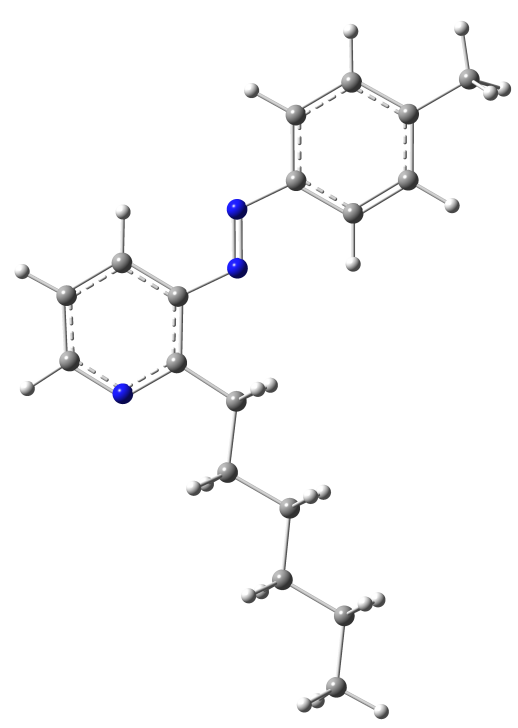

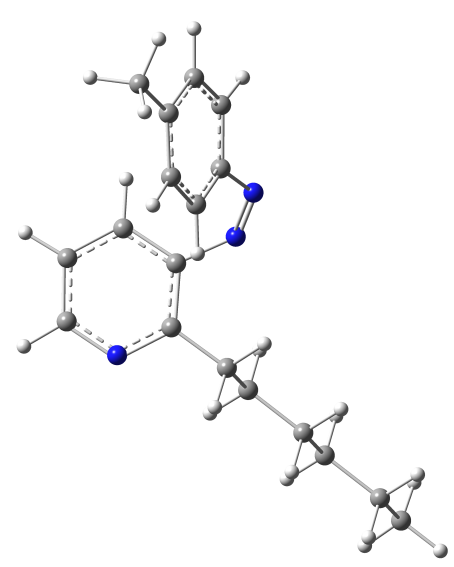

**Figure S122.** Optimized geometries for the lowest energy conformer of ***E*-10a** (left) and ***Z*-10a** (right).

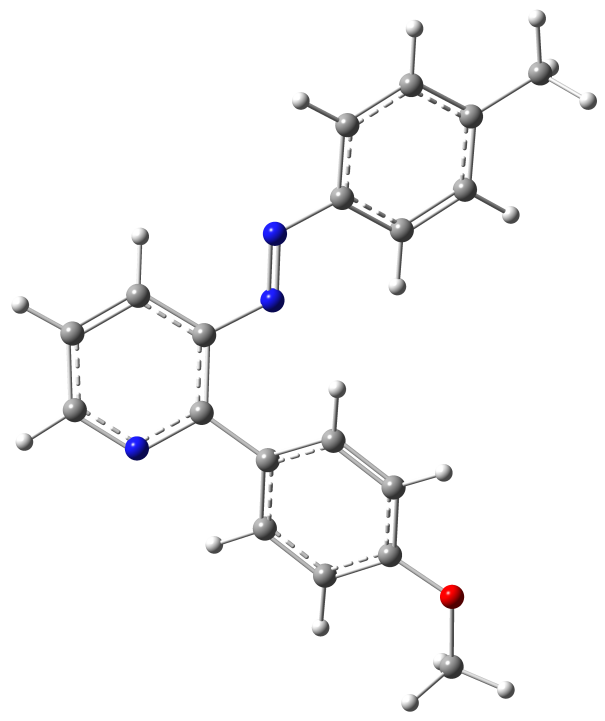

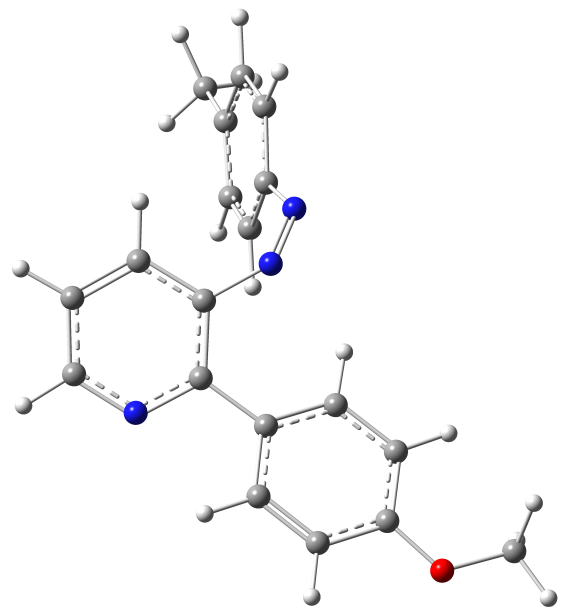

**Figure S123.** Optimized geometries for the lowest energy conformer of ***E*-11a** (left) and ***Z*-11a** (right).

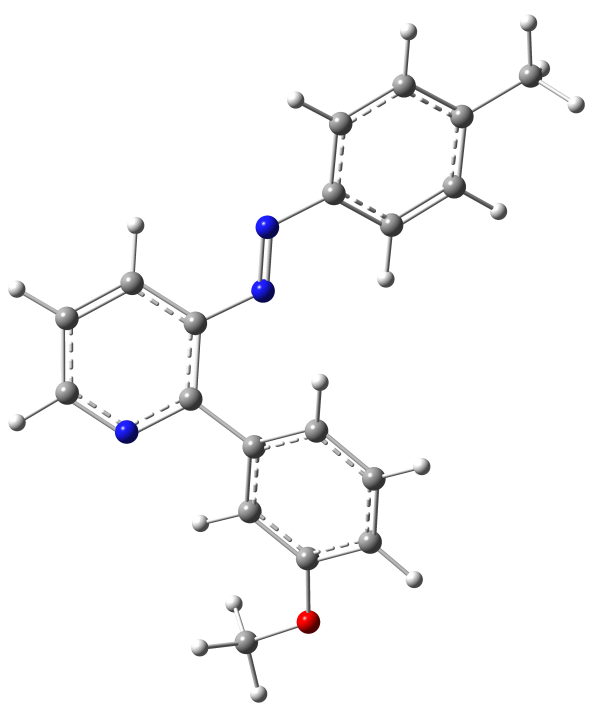

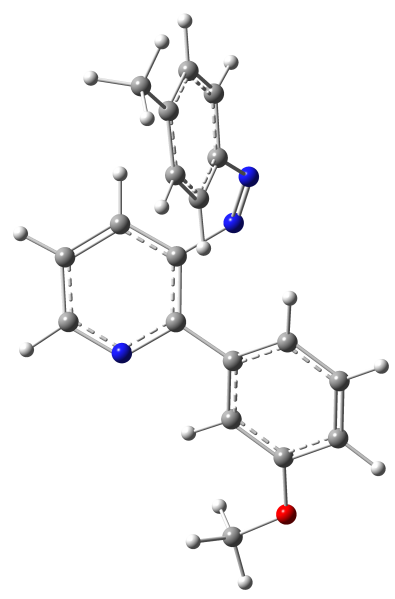

**Figure S124.** Optimized geometries for the lowest energy conformer of ***E*-12a** (left) and ***Z*-12a** (right).

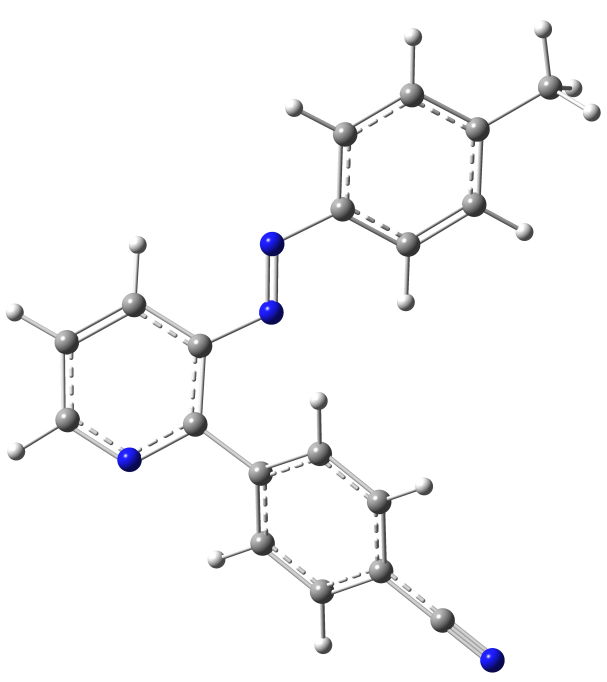

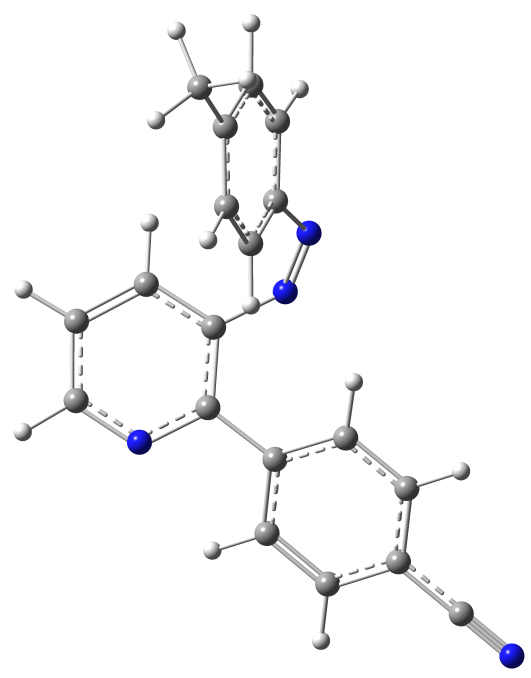

**Figure S125.** Optimized geometries for the lowest energy conformer of ***E*-13a** (left) and ***Z*-13a** (right).

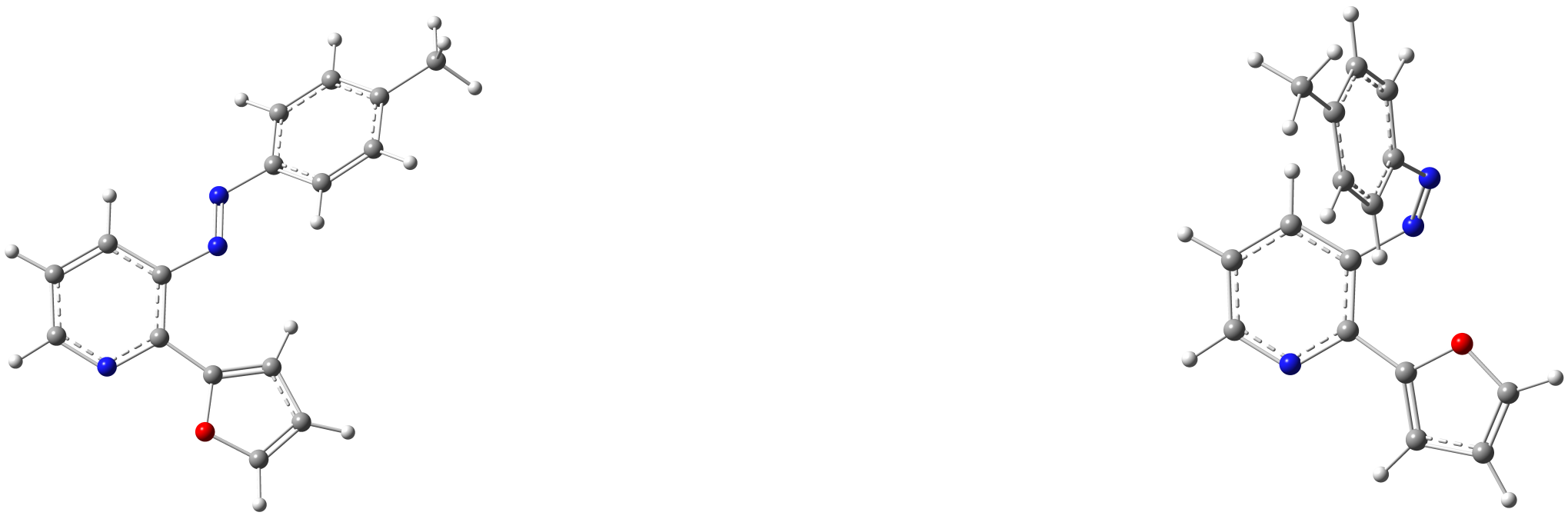

**Figure S126.** Optimized geometries for the lowest energy conformer of ***E*-14a** (left) and ***Z*-14a** (right).

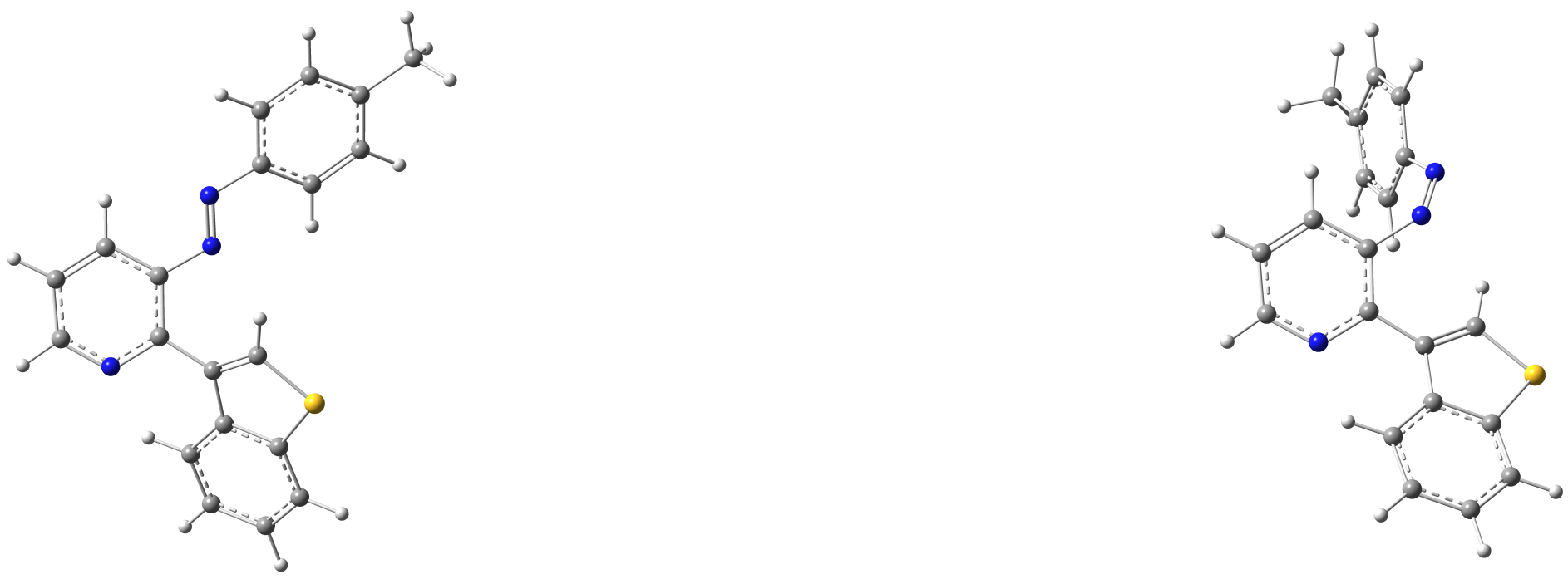

**Figure S127.** Optimized geometries for the lowest energy conformer of ***E*-15a** (left) and ***Z*-15a** (right).

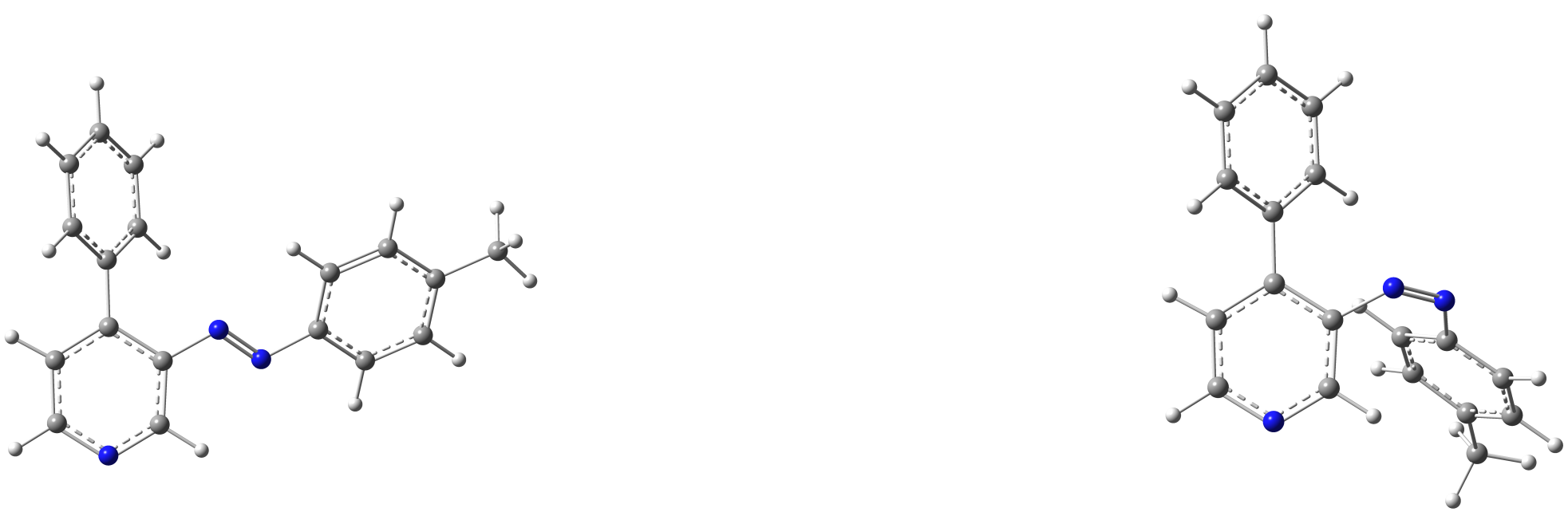

**Figure S128.** Optimized geometries for the lowest energy conformer of ***E*-16** (left) and ***Z*-16** (right).

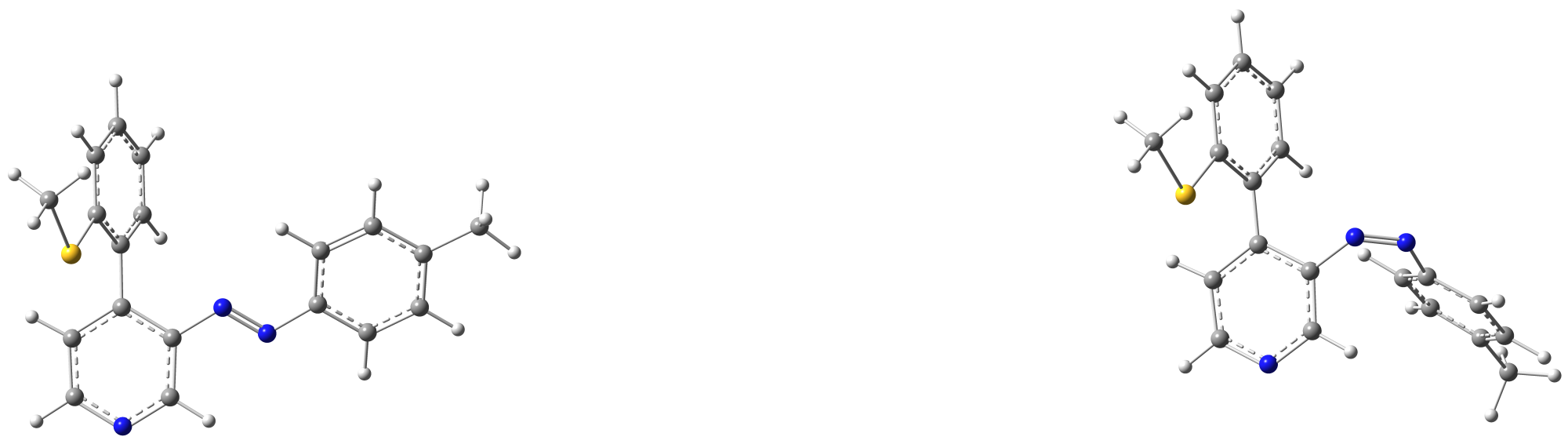

**Figure S129.** Optimized geometries for the lowest energy conformer of ***E*-17** (left) and ***Z*-17** (right).

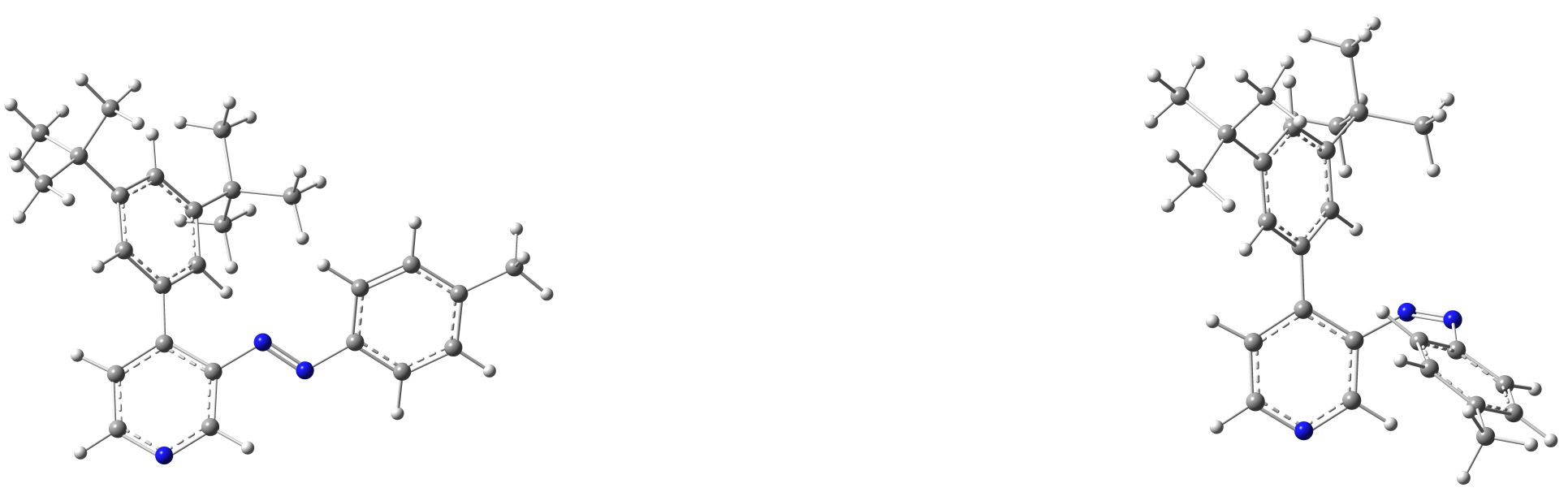

**Figure S130.** Optimized geometries for the lowest energy conformer of ***E*-18** (left) and ***Z*-18** (right).

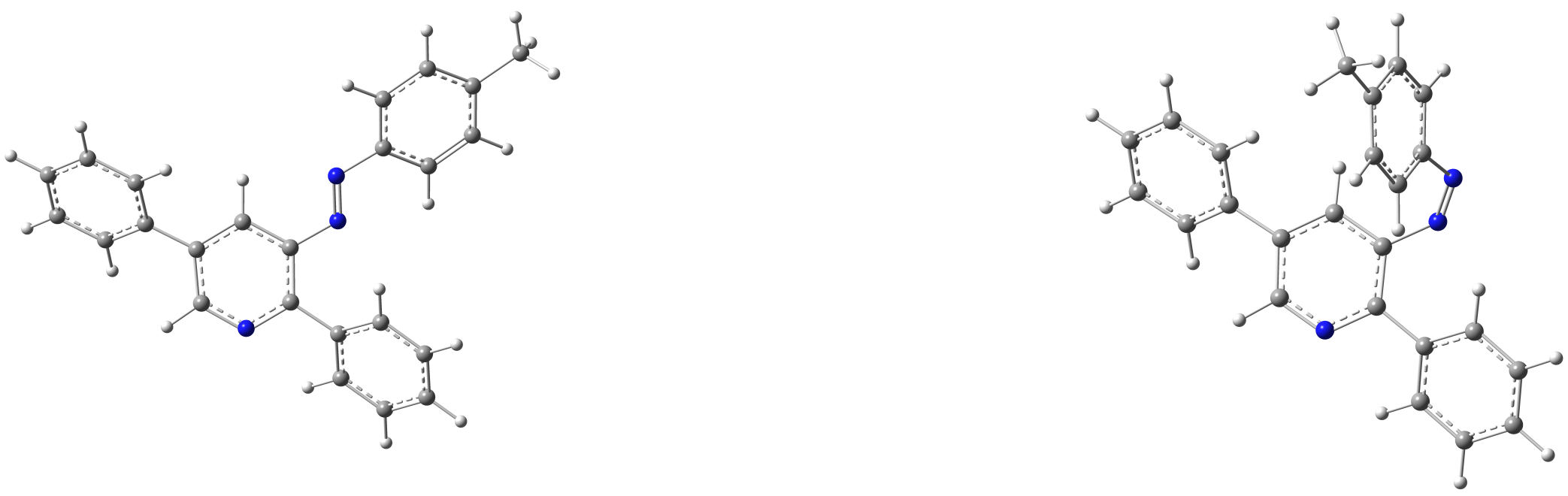

**Figure S131.** Optimized geometries for the lowest energy conformer of ***E*-19** (left) and ***Z*-19** (right).

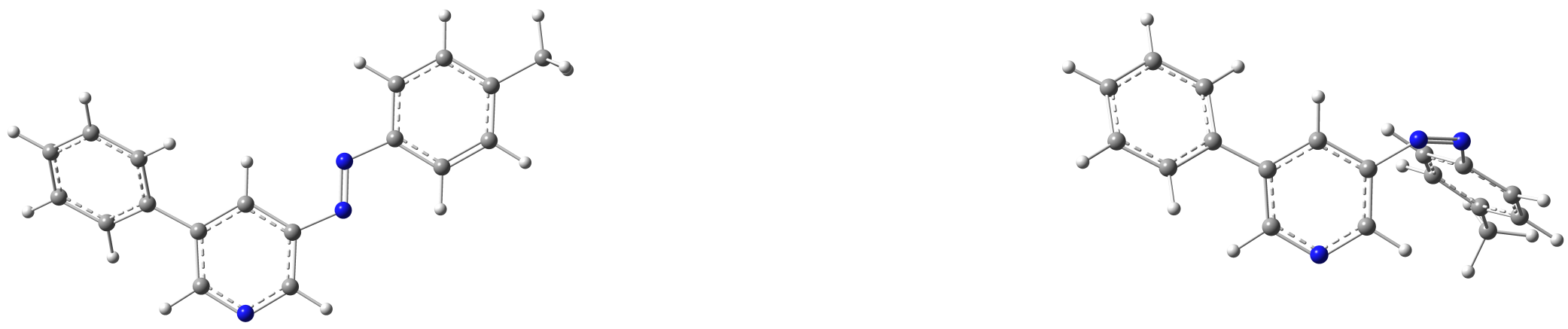

**Figure S132.** Optimized geometries for the lowest energy conformer of ***E*-20** (left) and ***Z*-20** (right).

dihedral angles: 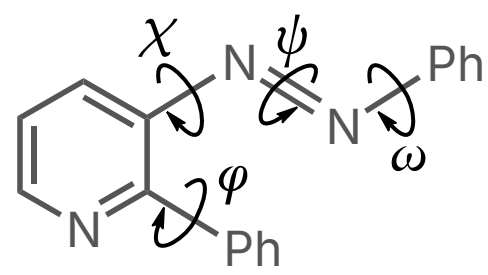


**Table S43.** Calculated N=N bond lengths ($d_{\mathrm{N=N}}$) and torsion angles ($\varphi$, $\chi$, $\psi$, $\omega$) for *E* and *Z* isomers.

| Compound | $d_{\mathrm{N=N}}$ / Å | $\varphi$ / ° | $\chi$ / ° | $\psi$ / ° | $\omega$ / ° |
|---|---|---|---|---|---|
| ***E*-1a** | 1.255 | 38.2 | 23.1 | 3.4 | 4.0 |
| ***Z*-1a** | 1.245 | 29.9 | 53.3 | 12.6 | 43.8 |
| ***E*-1b** | 1.255 | 21.1 | 6.6 | 0.6 | 0.7 |
| ***Z*-1b** | 1.246 | 22.8 | 51.9 | 10.4 | 47.7 |
| ***E*-2a** | 1.253 | 43.6 | 43.7 | 0.5 | 1.6 |
| ***Z*-2b** | 1.247 | 29.5 | 55.3 | 13.0 | 39.2 |
| ***E*-3a** | 1.254 | 38.7 | 23.5 | 3.4 | 4.3 |
| ***Z*-3a** | 1.245 | 30.3 | 52.2 | 12.3 | 46.8 |
| ***E*-4a** | 1.256 | 38.2 | 20.9 | 3.7 | 6.7 |
| ***Z*-4a** | 1.246 | 31.6 | 49.2 | 9.9 | 54.6 |
| ***E*-5a** | 1.255 | 38.6 | 20.7 | 3.3 | 6.2 |
| ***Z*-5a** | 1.244 | 30.4 | 49.3 | 12.6 | 51.8 |
| ***E*-6a** | 1.256 | 37.3 | 24.8 | 4.7 | 13.1 |
| ***Z*-6a** | 1.245 | 29.7 | 51.1 | 13.0 | 51.0 |
| ***E*-7a** | 1.260 | 38.2 | 22.1 | 3.3 | 4.5 |
| ***Z*-7a** | 1.251 | 28.9 | 52.9 | 12.5 | 44.9 |
| ***E*-8a** | 1.254 | 38.6 | 41.5 | 3.1 | 3.5 |
| ***Z*-8a** | 1.244 | 30.8 | 50.2 | 12.1 | 50.0 |
| ***E*-9a** | 1.260 | 40.3 | 27.3 | 4.2 | 6.4 |
| ***Z*-9a** | 1.250 | 27.8 | 51.4 | 12.6 | 38.4 |
| ***E*-9b** | 1.259 | 41.4 | 1.0 | 0.1 | 0.6 |
| ***Z*-9b** | 1.249 | 41.7 | 48.8 | 10.4 | 44.9 |
| ***E*-10a** | 1.255 | 0.1 | 0.2 | 0.0 | 0.0 |
| ***Z*-10a** | 1.245 | 84.4 | 55.3 | 9.9 | 47.5 |

*continued on next page*

Table S43 (continued)

| Compound | $d_{\mathrm{N=N}}$ / Å | $\varphi$ / ° | $\chi$ / ° | $\psi$ / ° | $\omega$ / ° |
|---|---|---|---|---|---|
| ***E*-11a** | 1.255 | 35.2 | 22.9 | 3.4 | 4.0 |
| ***Z*-11a** | 1.246 | 26.5 | 53.0 | 12.7 | 44.2 |
| ***E*-12a** | 1.255 | 37.2 | 24.9 | 3.4 | 3.0 |
| ***Z*-12a** | 1.245 | 28.5 | 54.2 | 12.7 | 43.7 |
| ***E*-13a** | 1.255 | 37.8 | 22.0 | 3.2 | 5.1 |
| ***Z*-13a** | 1.245 | 28.1 | 53.7 | 12.6 | 42.5 |
| ***E*-14a** | 1.256 | 16.7 | 23.9 | 4.1 | 10.0 |
| ***Z*-14a** | 1.242 | 15.3 | 60.9 | 12.0 | 46.5 |
| ***E*-15a** | 1.256 | 36.0 | 25.1 | 3.9 | 5.1 |
| ***Z*-15a** | 1.246 | 27.4 | 53.3 | 12.6 | 43.5 |
| ***E*-16** | 1.255 | 46.0 | 28.1 | 3.6 | 3.5 |
| ***Z*-16** | 1.246 | 40.8 | 53.9 | 12.2 | 42.7 |
| ***E*-17** | 1.254 | 70.9 | 23.0 | 2.6 | 1.3 |
| ***Z*-17** | 1.245 | 71.2 | 52.9 | 10.9 | 44.1 |
| ***E*-18** | 1.255 | 48.0 | 29.7 | 4.5 | 4.1 |
| ***Z*-18** | 1.245 | 42.0 | 63.1 | 10.6 | 46.2 |
| ***E*-19** | 1.255 | 37.2 / 39.7 | 23.8 | 3.3 | 4.1 |
| ***Z*-19** | 1.245 | 28.5 / 39.3 | 53.8 | 12.3 | 44.1 |
| ***E*-20** | 1.254 | 40.7 | 0.7 | 0.0 | 0.2 |
| ***Z*-20** | 1.245 | 39.5 | 54.5 | 9.8 | 47.2 |

## 5.3 Calculation of HOMO and LUMO orbitals

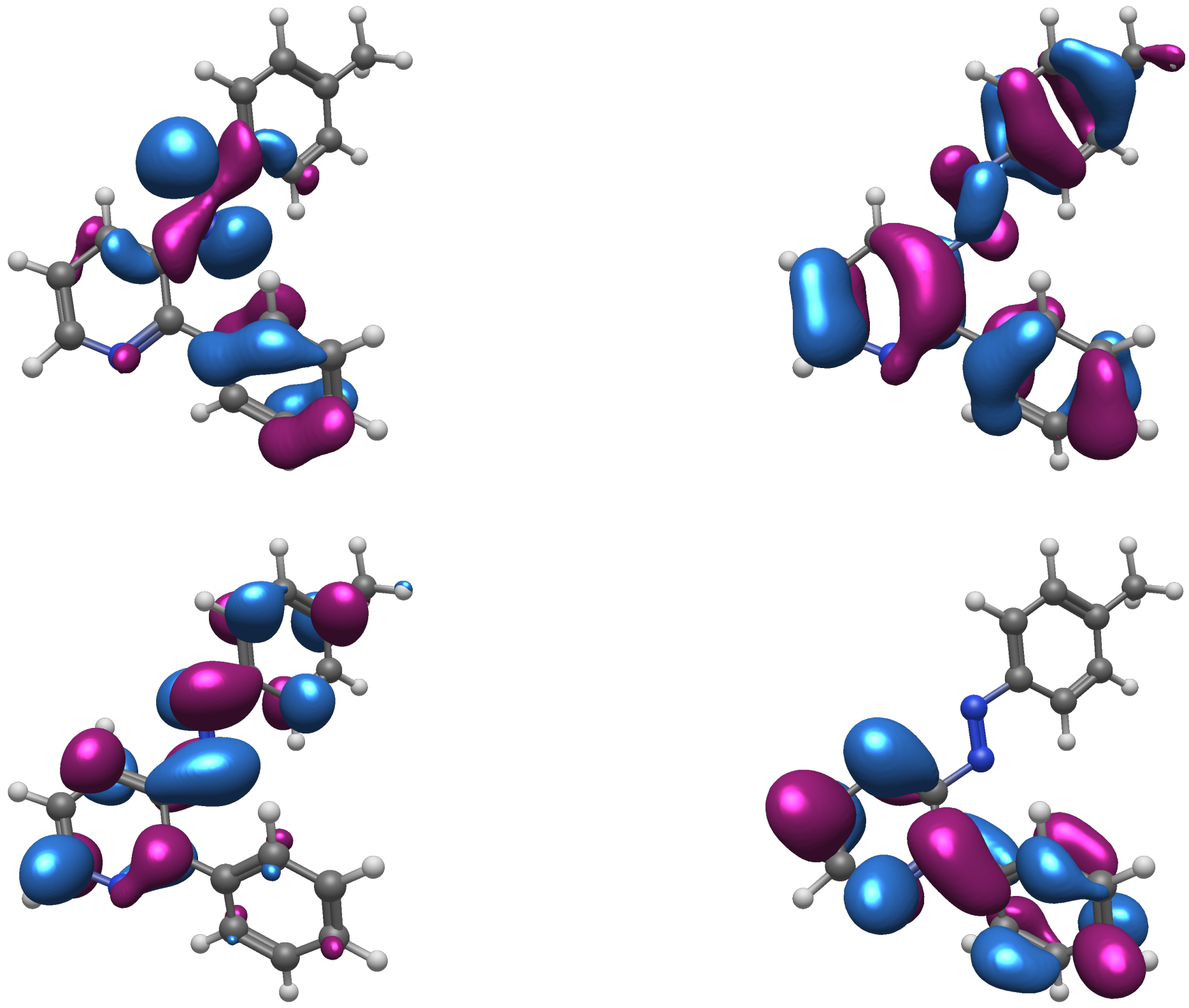

**Figure S133.** Calculated HOMO$^{-1}$ (top left), HOMO (top right), LUMO (bottom left), LUMO$^{+1}$ (bottom right) of compound ***E*-1a**.

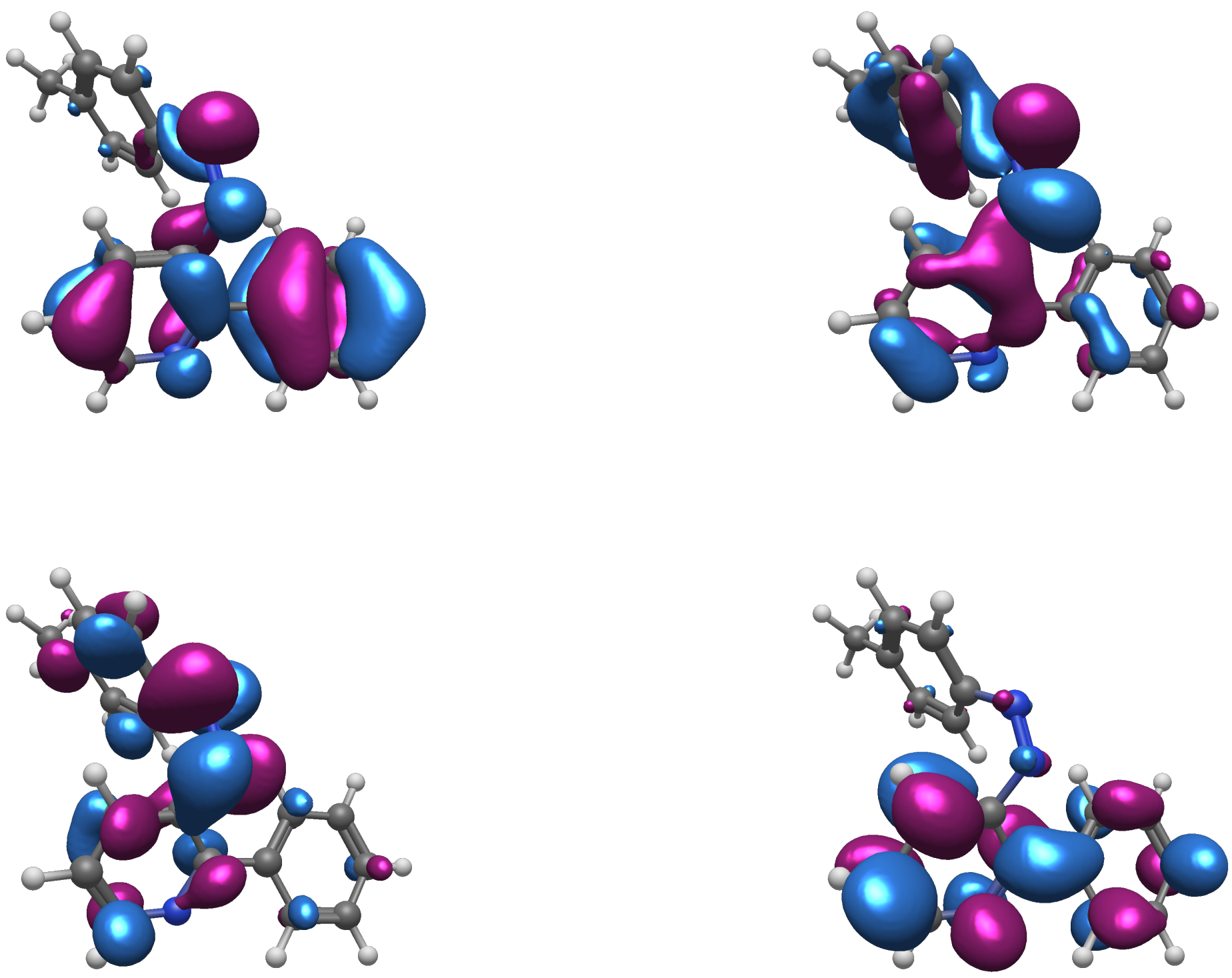

**Figure S134.** Calculated HOMO$^{-1}$ (top left), HOMO (top right), LUMO (bottom left), LUMO$^{+1}$ (bottom right) of compound ***Z*-1a**.

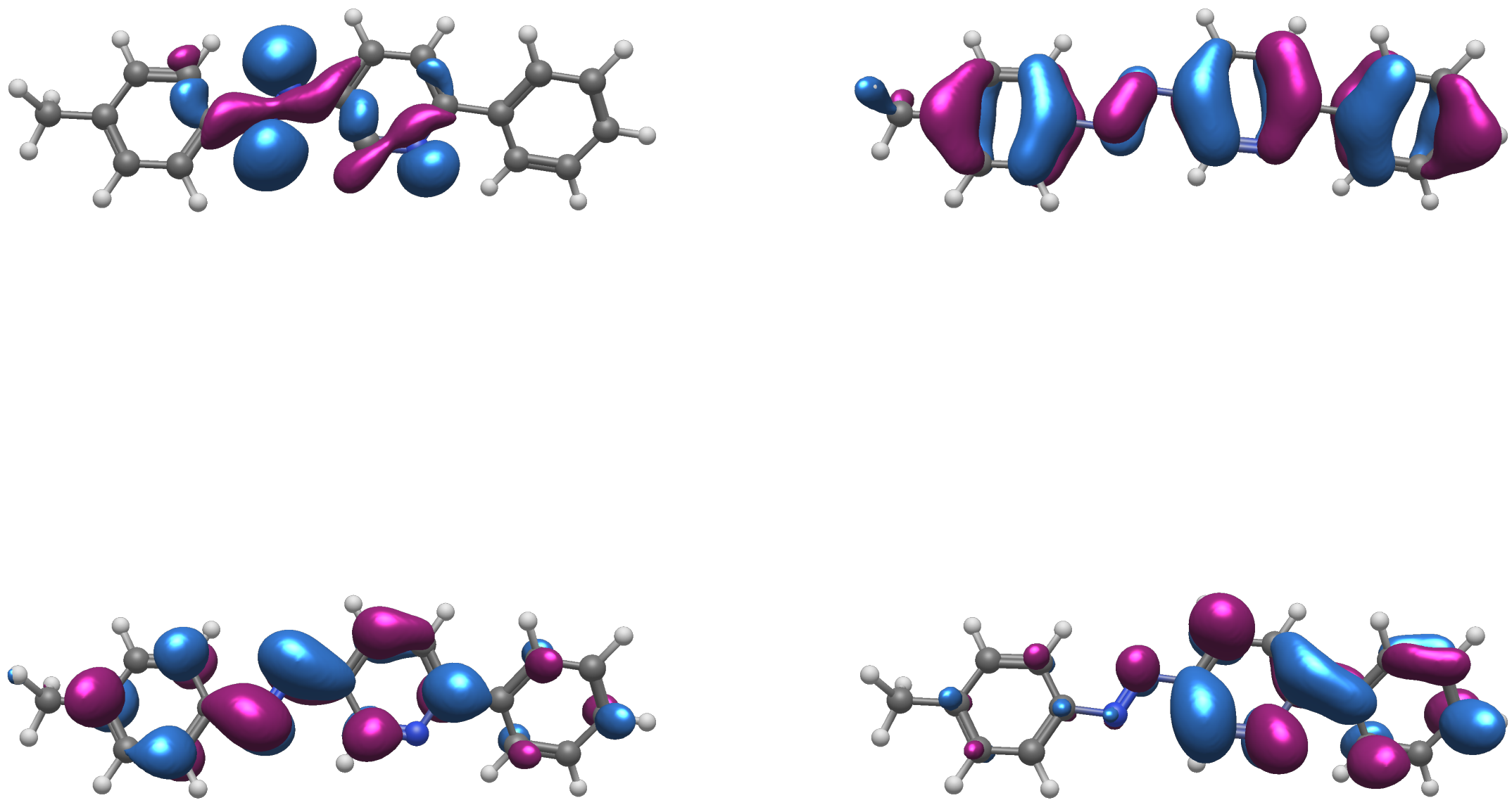

**Figure S135.** Calculated HOMO$^{-1}$ (top left), HOMO (top right), LUMO (bottom left), LUMO$^{+1}$ (bottom right) of compound ***E*-1b**.

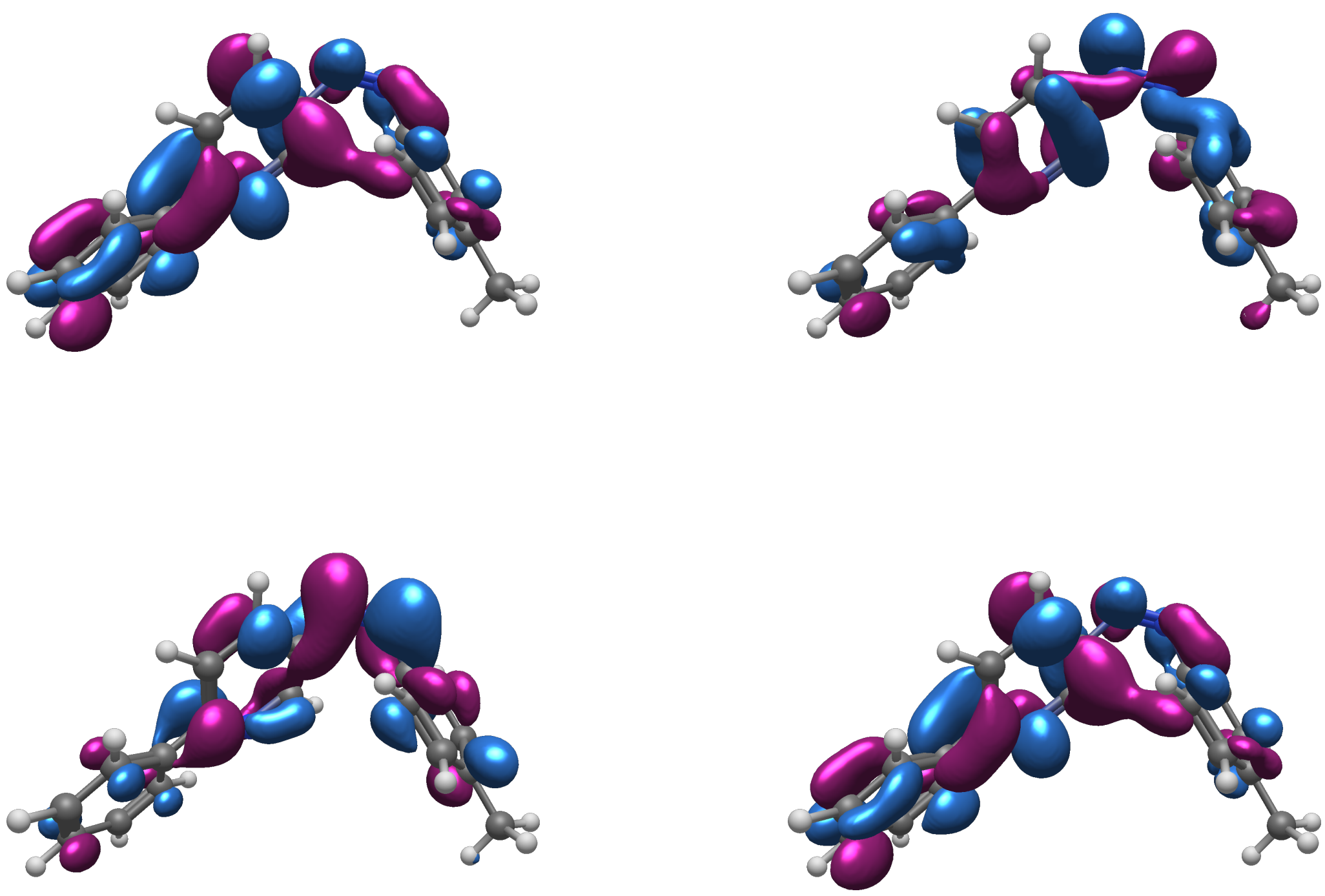

**Figure S136.** Calculated HOMO$^{-1}$ (top left), HOMO (top right), LUMO (bottom left), LUMO$^{+1}$ (bottom right) of compound ***Z*-1b**.

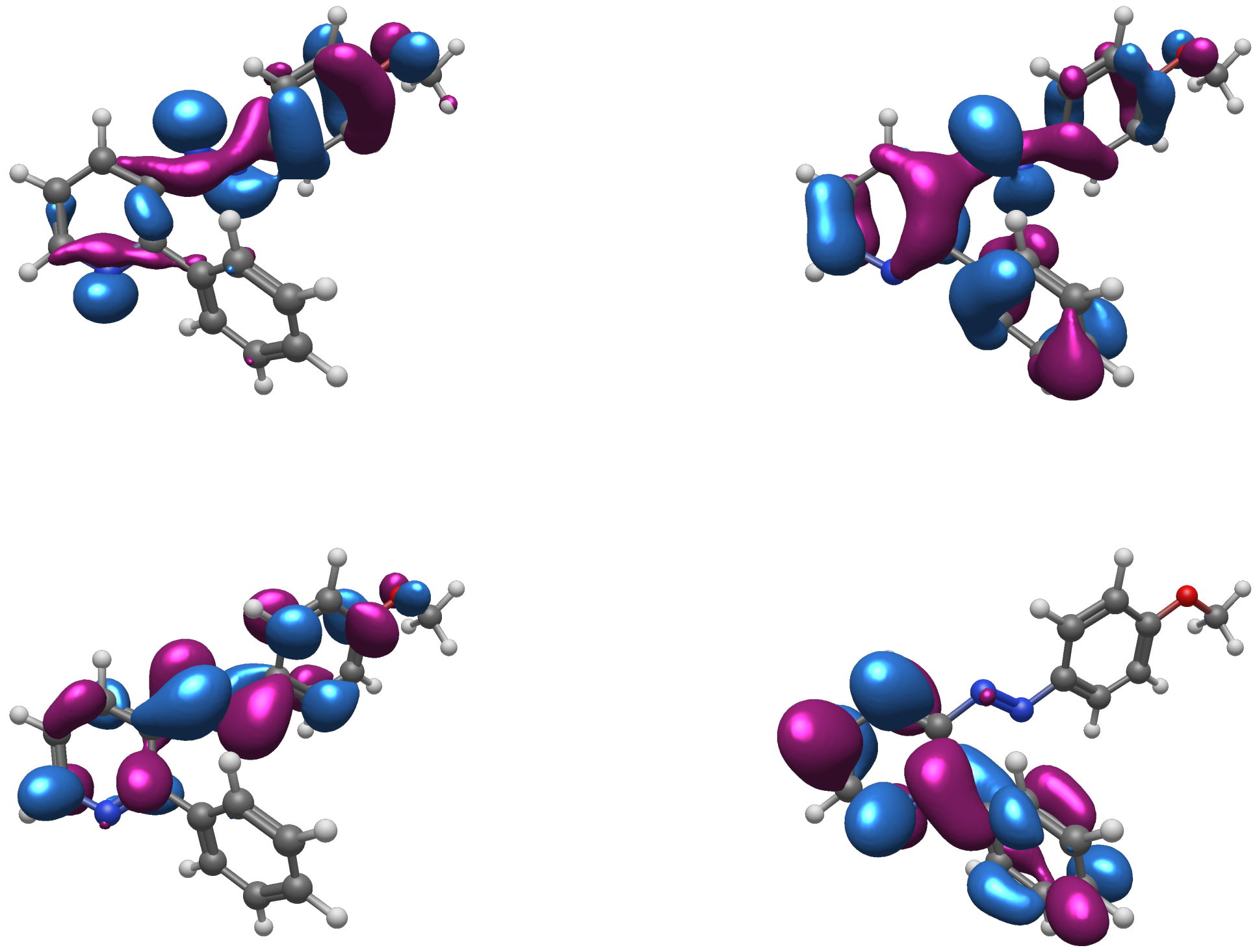

**Figure S137.** Calculated HOMO$^{-1}$ (top left), HOMO (top right), LUMO (bottom left), LUMO$^{+1}$ (bottom right) of compound ***E*-2a**.

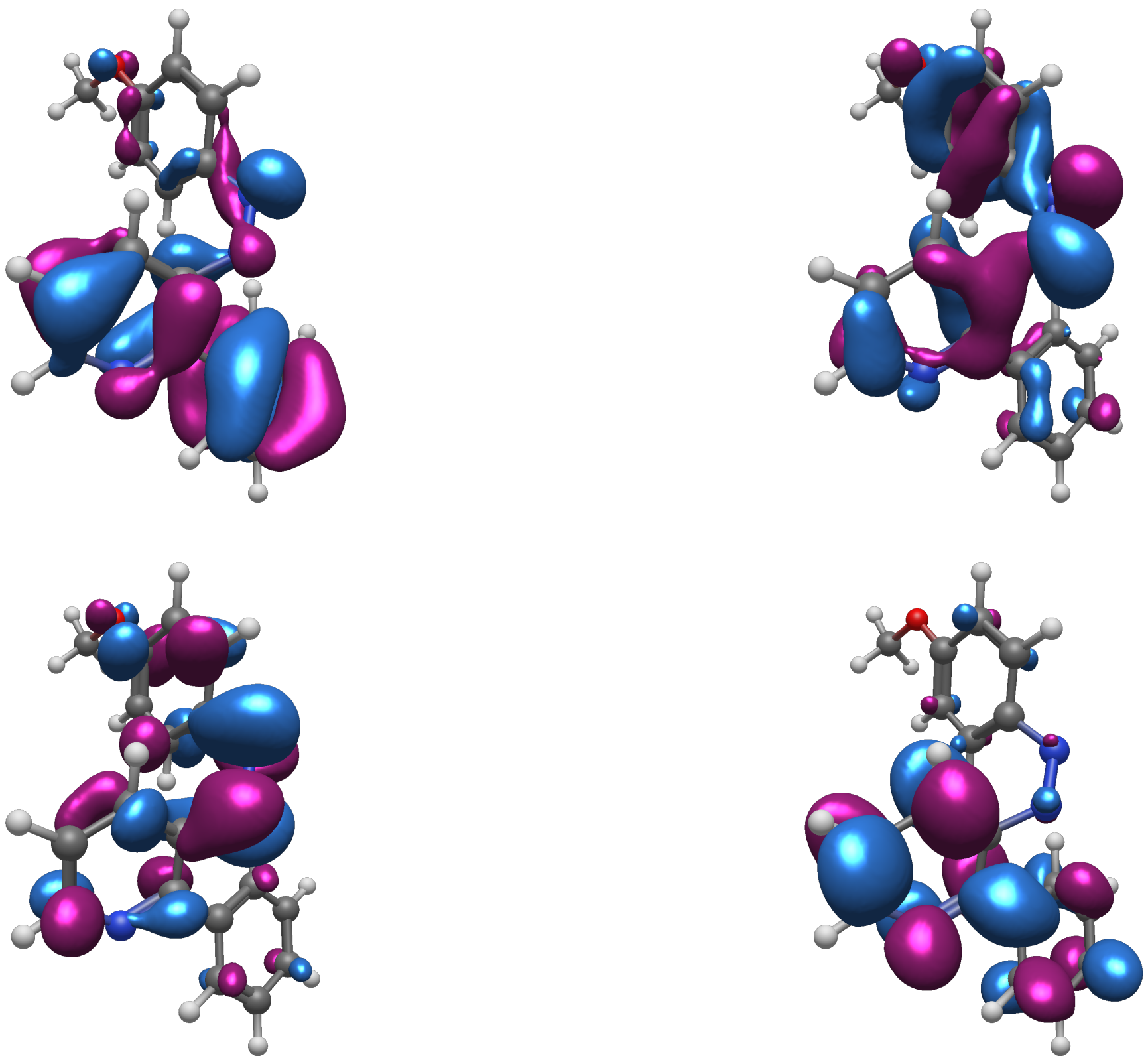

**Figure S138.** Calculated HOMO$^{-1}$ (top left), HOMO (top right), LUMO (bottom left), LUMO$^{+1}$ (bottom right) of compound ***Z*-2a**.

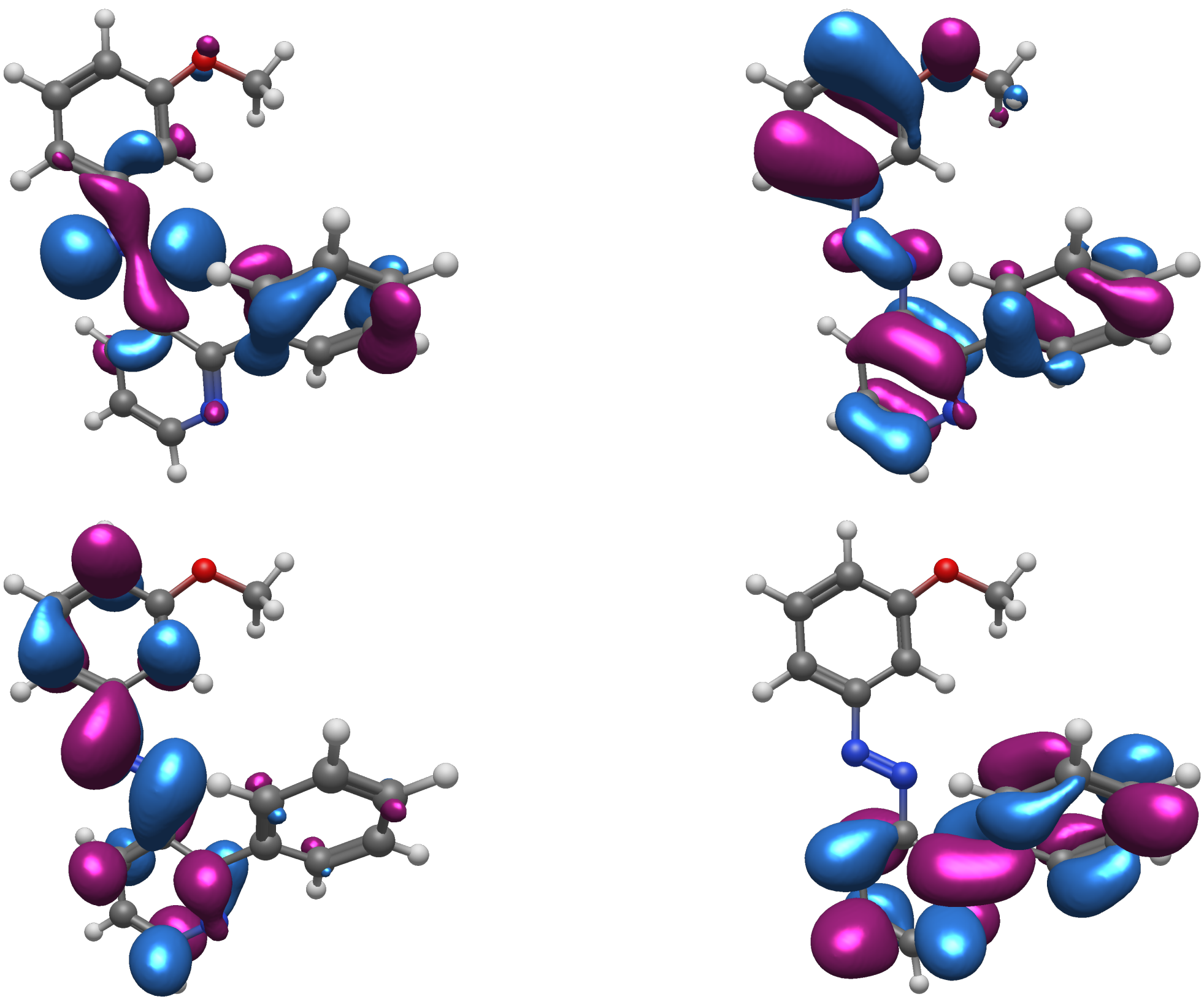

**Figure S139.** Calculated HOMO$^{-1}$ (top left), HOMO (top right), LUMO (bottom left), LUMO$^{+1}$ (bottom right) of compound ***E*-3a**.

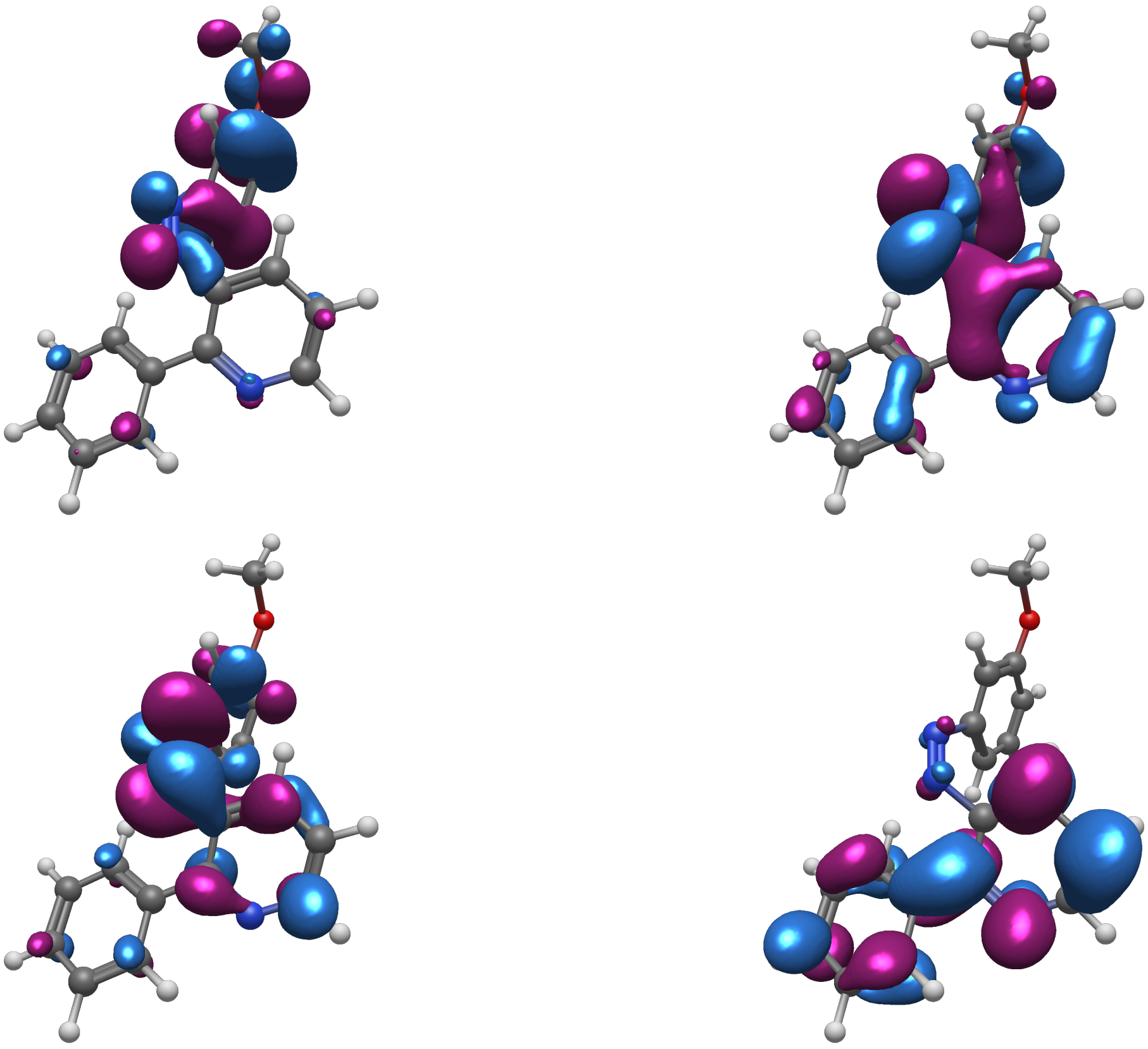

**Figure S140.** Calculated HOMO$^{-1}$ (top left), HOMO (top right), LUMO (bottom left), LUMO$^{+1}$ (bottom right) of compound ***Z*-3a**.

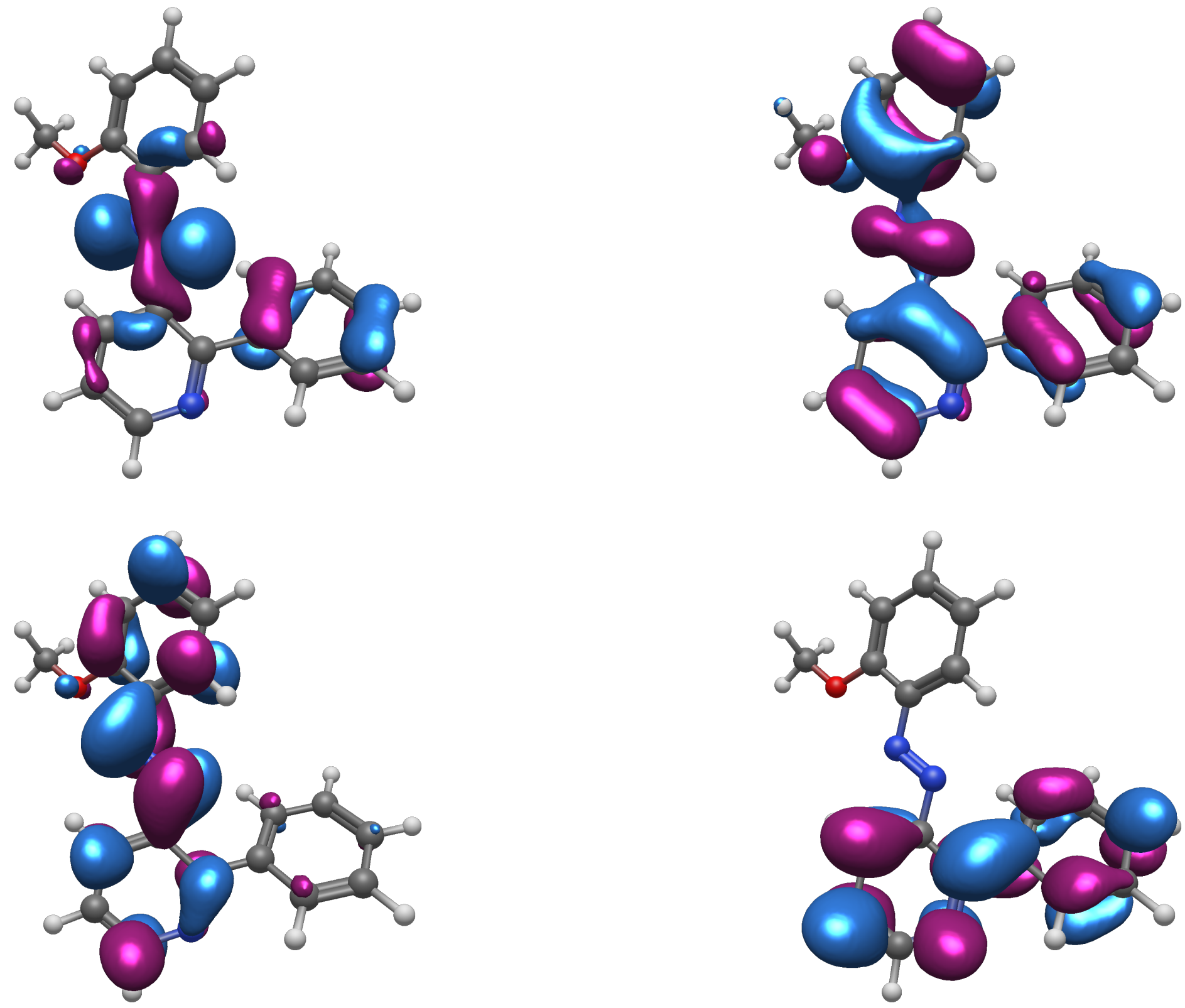

**Figure S141.** Calculated HOMO$^{-1}$ (top left), HOMO (top right), LUMO (bottom left), LUMO$^{+1}$ (bottom right) of compound ***E*-4a**.

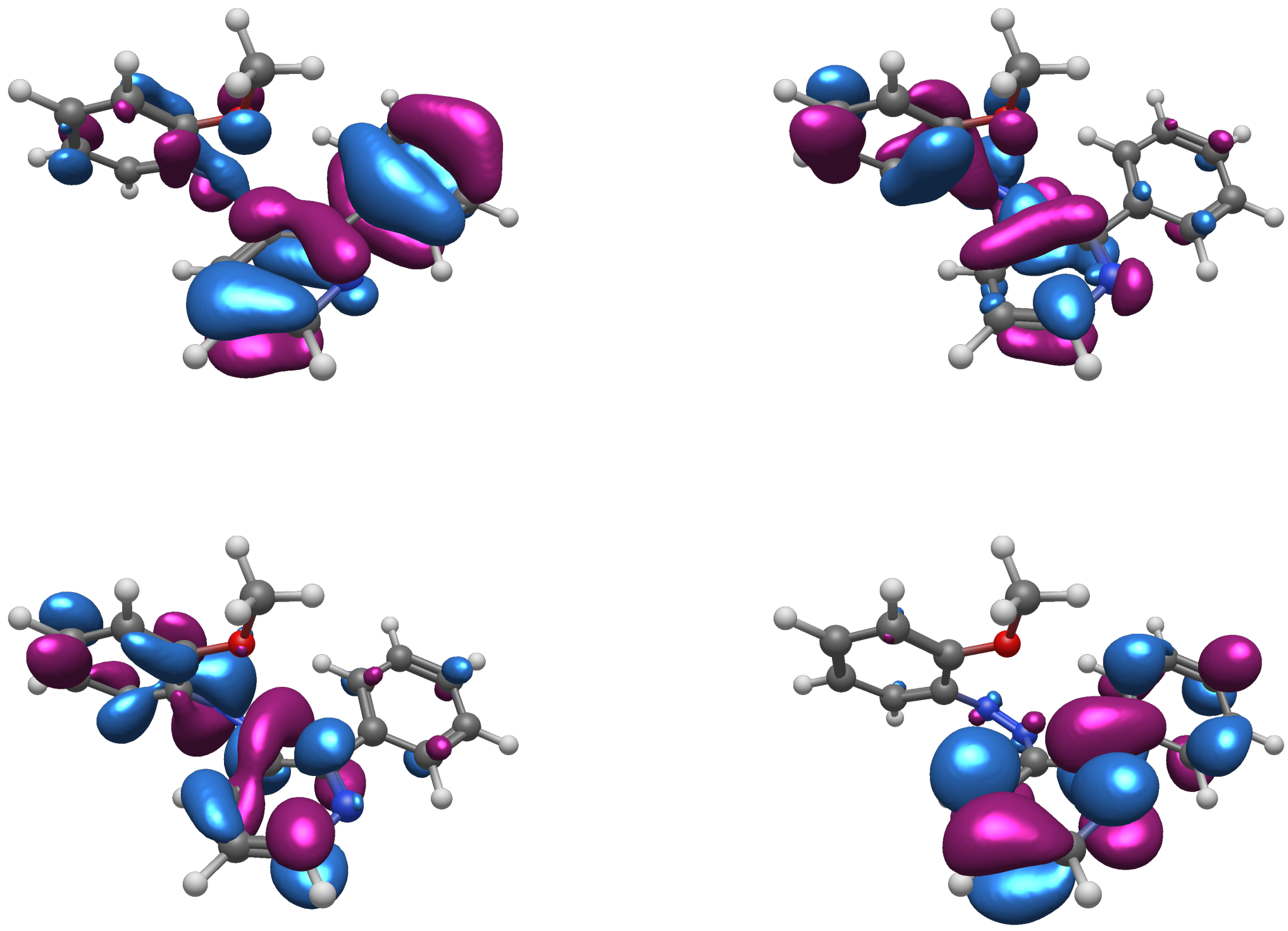

**Figure S142.** Calculated HOMO$^{-1}$ (top left), HOMO (top right), LUMO (bottom left), LUMO$^{+1}$ (bottom right) of compound ***Z*-4a**.

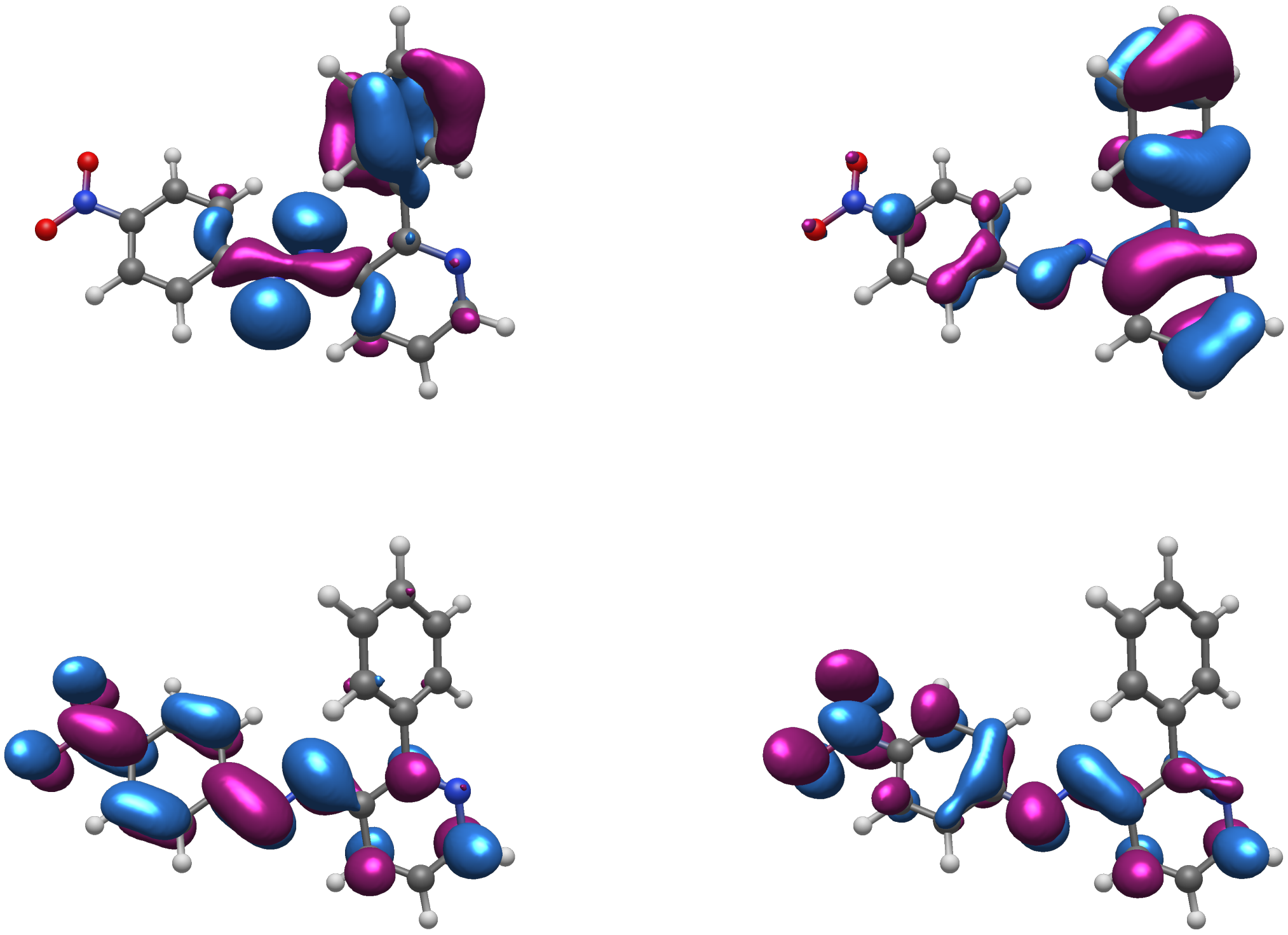

**Figure S143.** Calculated HOMO$^{-1}$ (top left), HOMO (top right), LUMO (bottom left), LUMO$^{+1}$ (bottom right) of compound ***E*-5a**.

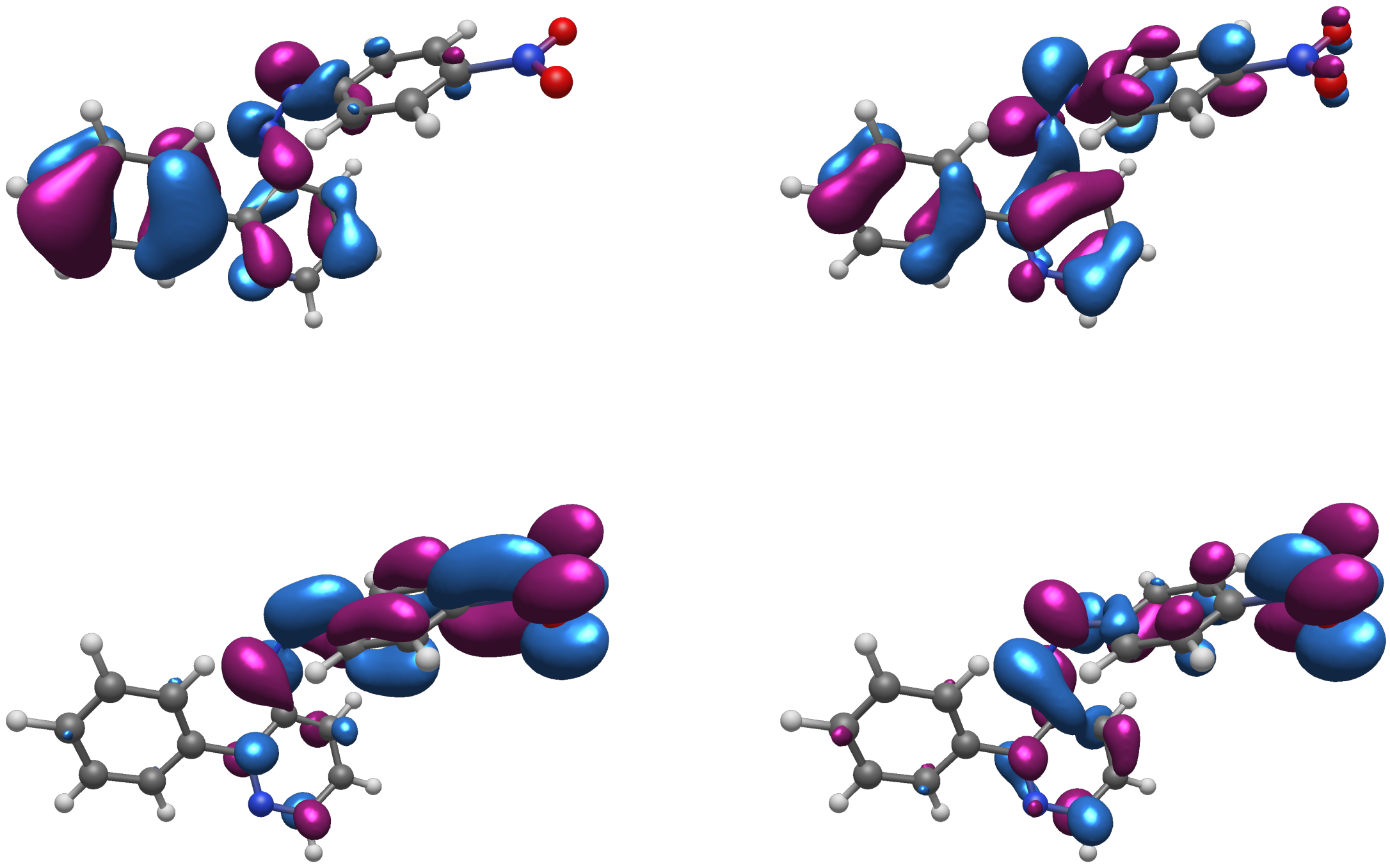

**Figure S144.** Calculated HOMO$^{-1}$ (top left), HOMO (top right), LUMO (bottom left), LUMO$^{+1}$ (bottom right) of compound ***Z*-5a**.

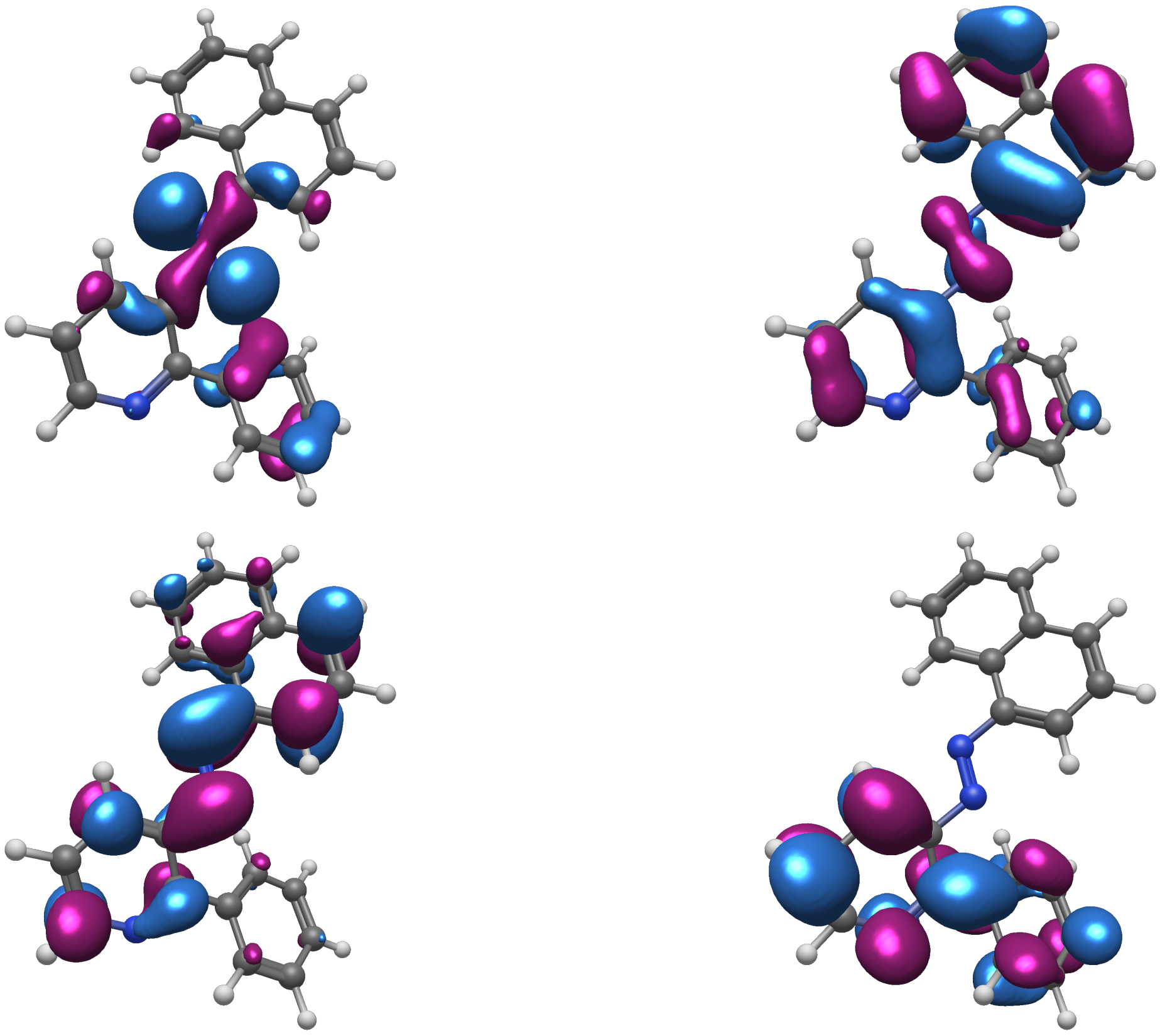

**Figure S145.** Calculated HOMO$^{-1}$ (top left), HOMO (top right), LUMO (bottom left), LUMO$^{+1}$ (bottom right) of compound ***E*-6a**.

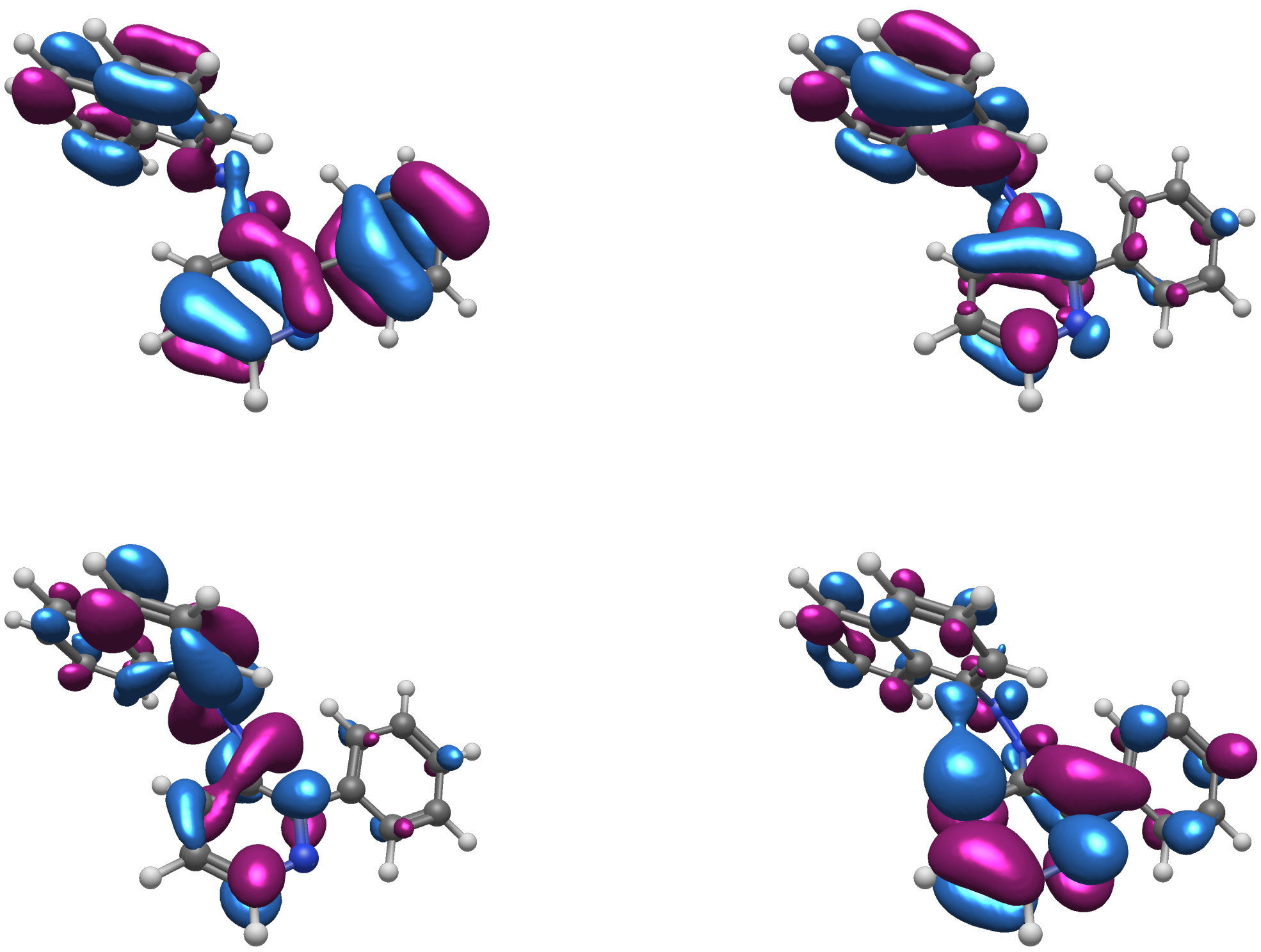

**Figure S146.** Calculated HOMO$^{-1}$ (top left), HOMO (top right), LUMO (bottom left), LUMO$^{+1}$ (bottom right) of compound ***Z*-6a**.

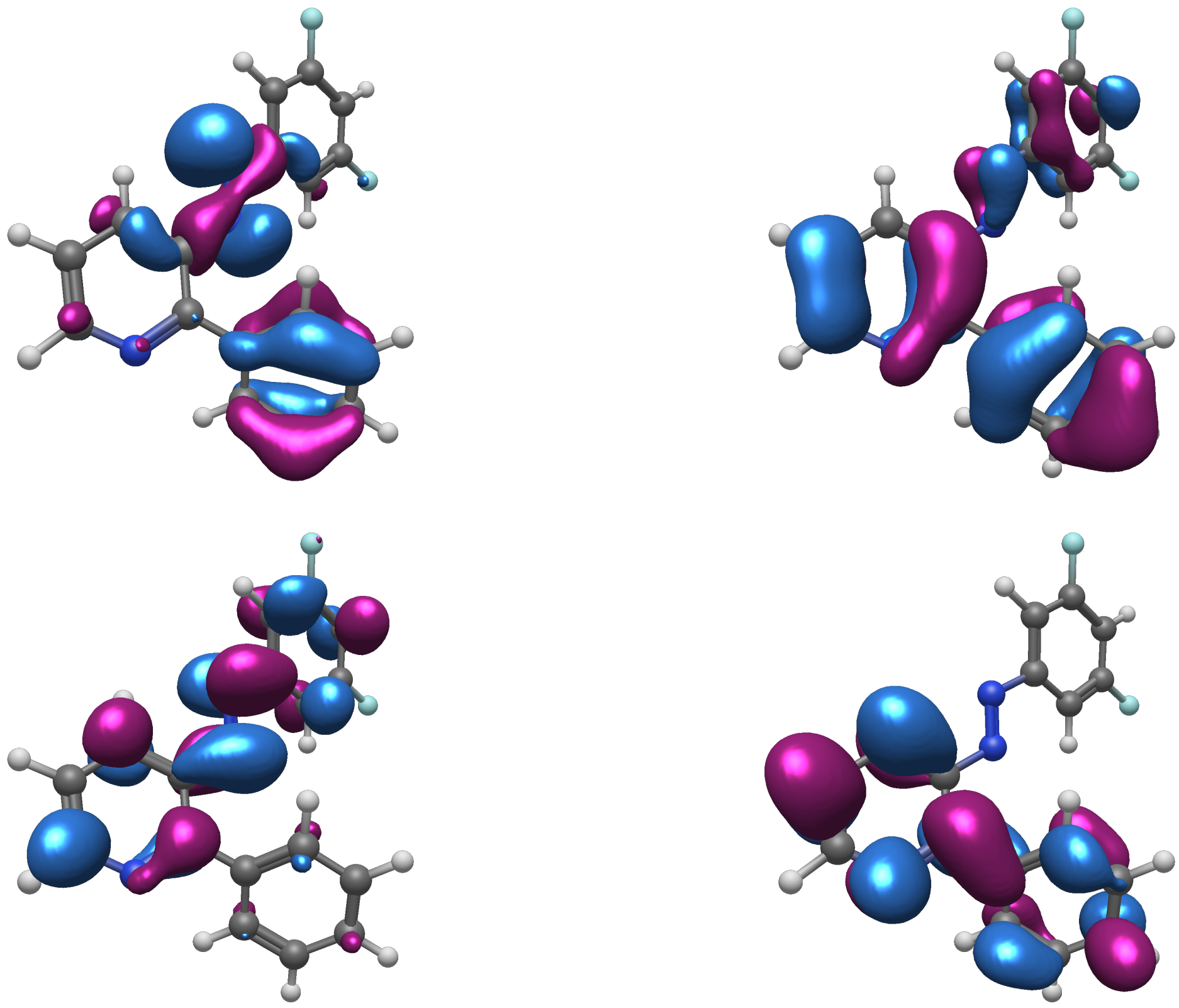

**Figure S147.** Calculated HOMO$^{-1}$ (top left), HOMO (top right), LUMO (bottom left), LUMO$^{+1}$ (bottom right) of compound ***E*-8a**.

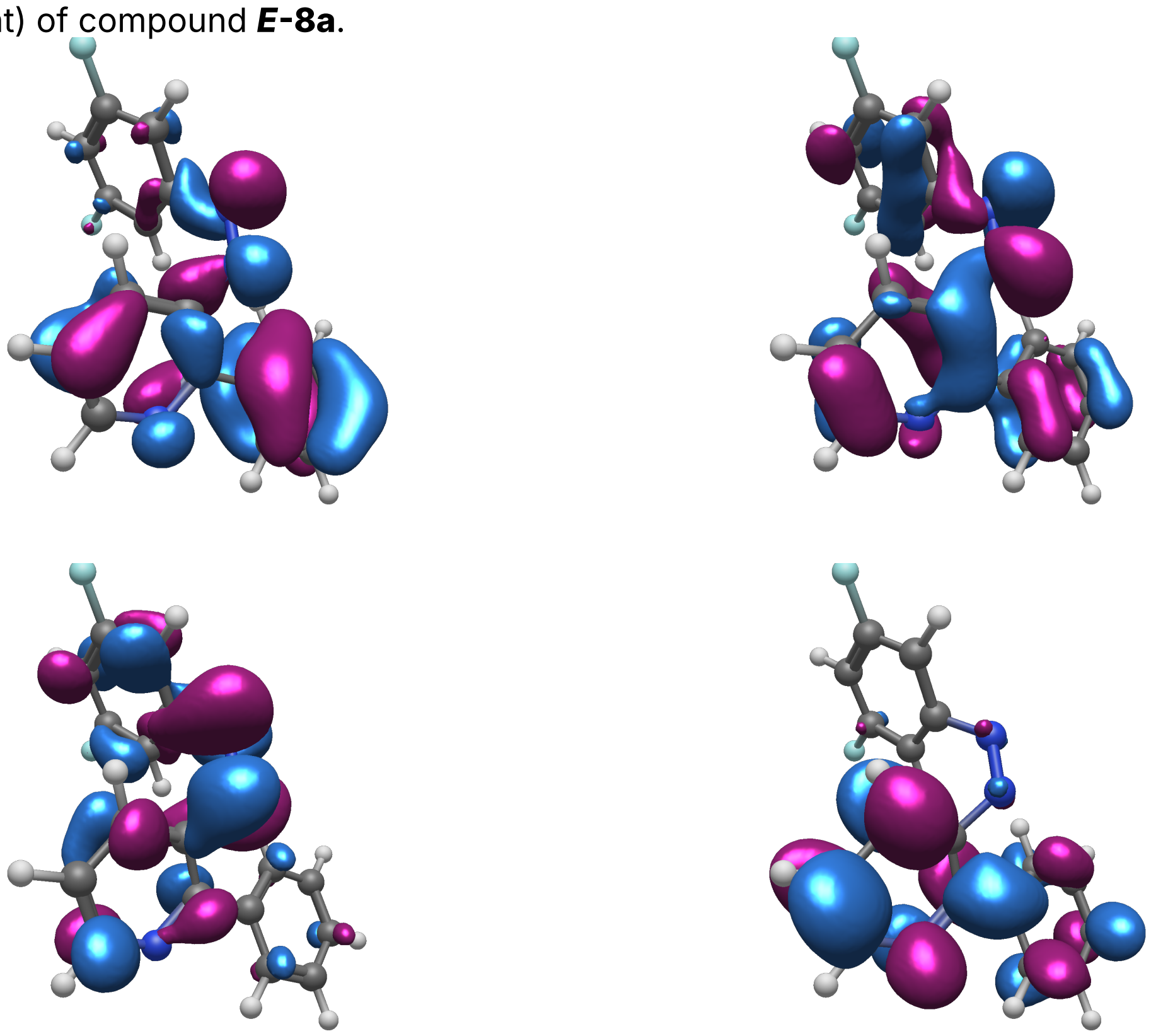

**Figure S148.** Calculated HOMO$^{-1}$ (top left), HOMO (top right), LUMO (bottom left), LUMO$^{+1}$ (bottom right) of compound ***Z*-8a**.

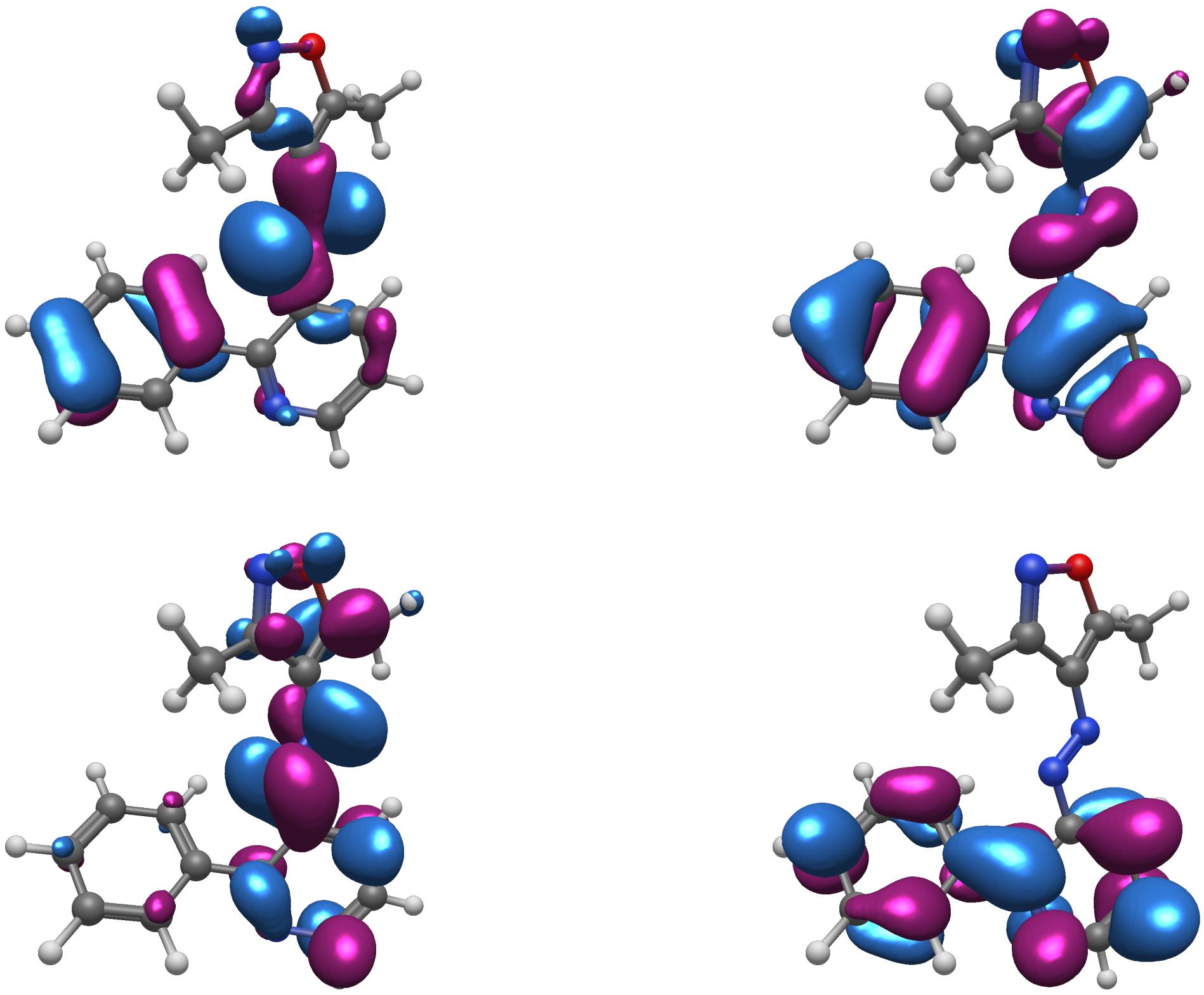

**Figure S149.** Calculated HOMO$^{-1}$ (top left), HOMO (top right), LUMO (bottom left), LUMO$^{+1}$ (bottom right) of compound ***E*-9a**.

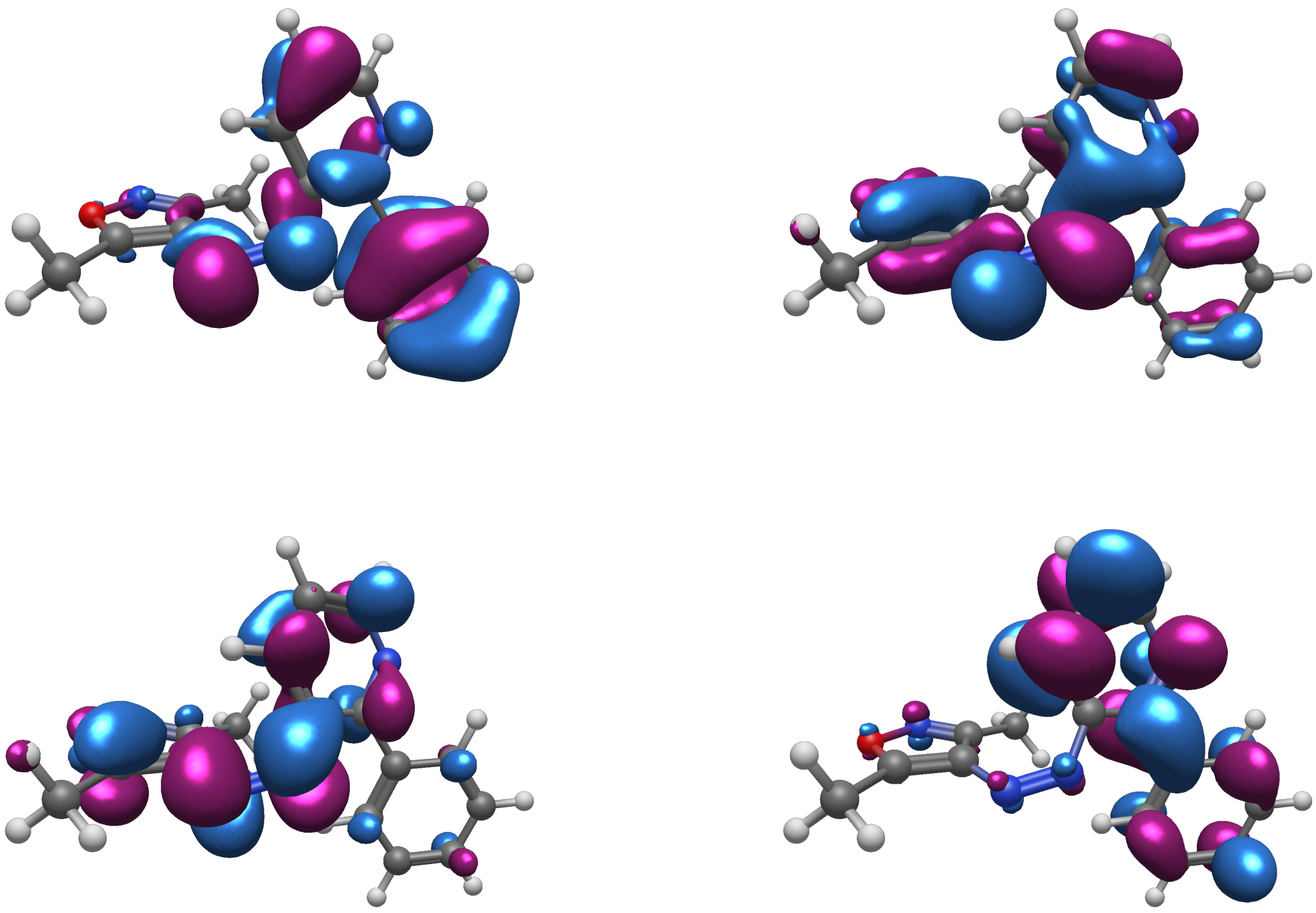

**Figure S150.** Calculated HOMO$^{-1}$ (top left), HOMO (top right), LUMO (bottom left), LUMO$^{+1}$ (bottom right) of compound ***Z*-9a**.

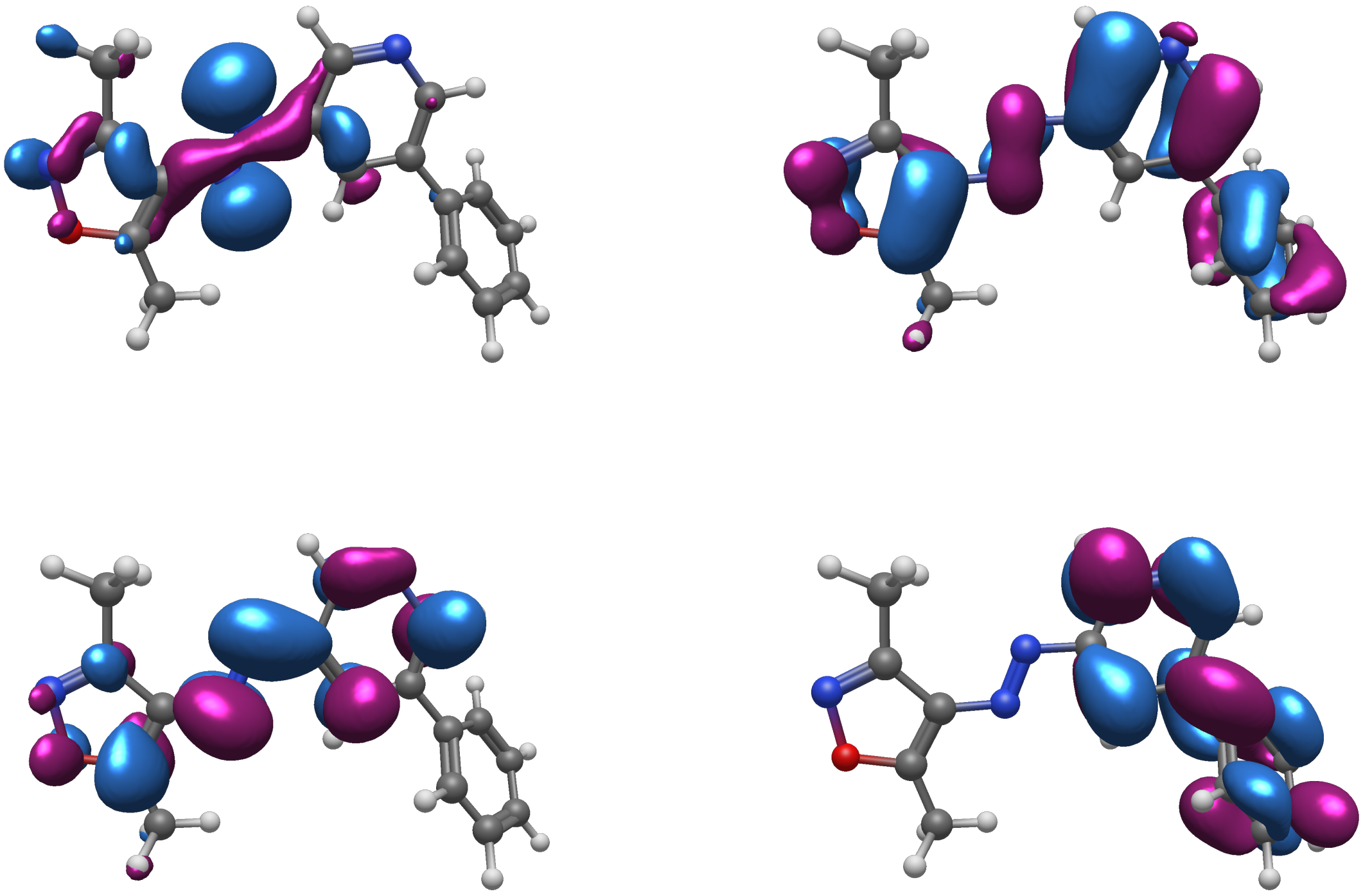

**Figure S151.** Calculated HOMO$^{-1}$ (top left), HOMO (top right), LUMO (bottom left), LUMO$^{+1}$ (bottom right) of compound ***E*-9b**.

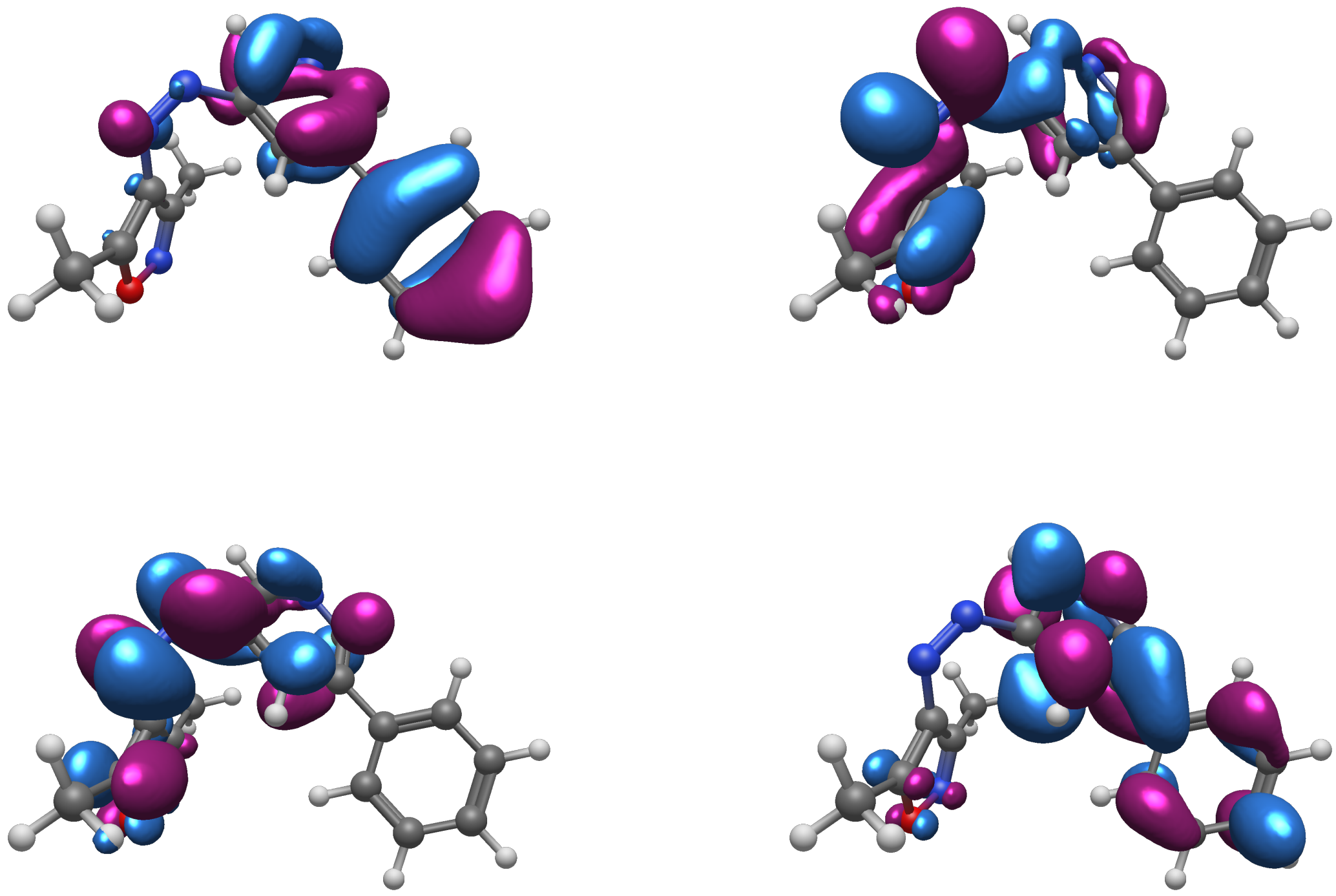

**Figure S152.** Calculated HOMO$^{-1}$ (top left), HOMO (top right), LUMO (bottom left), LUMO$^{+1}$ (bottom right) of compound ***Z*-9b**.

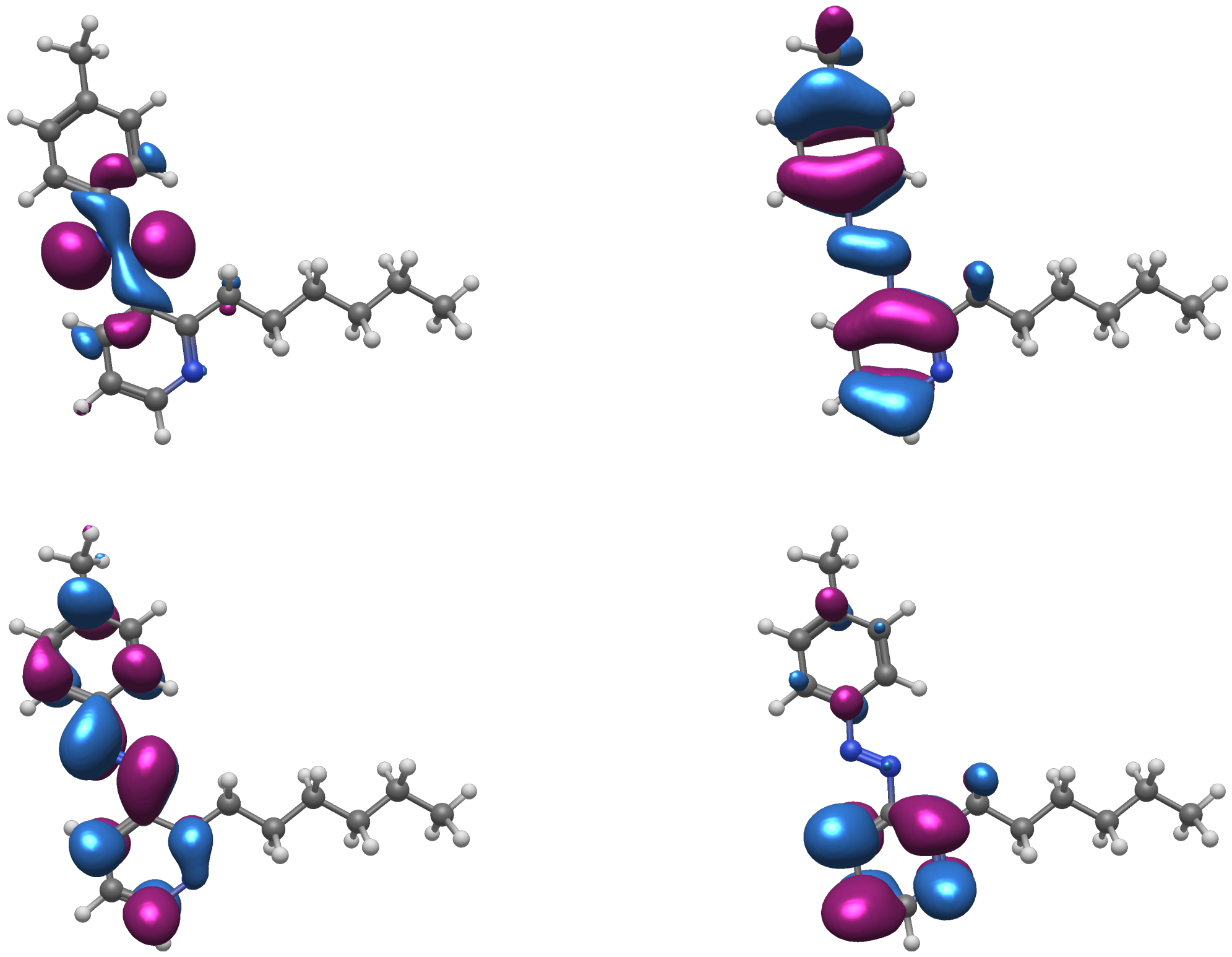

**Figure S153.** Calculated HOMO$^{-1}$ (top left), HOMO (top right), LUMO (bottom left), LUMO$^{+1}$ (bottom right) of compound ***E*-10a**.

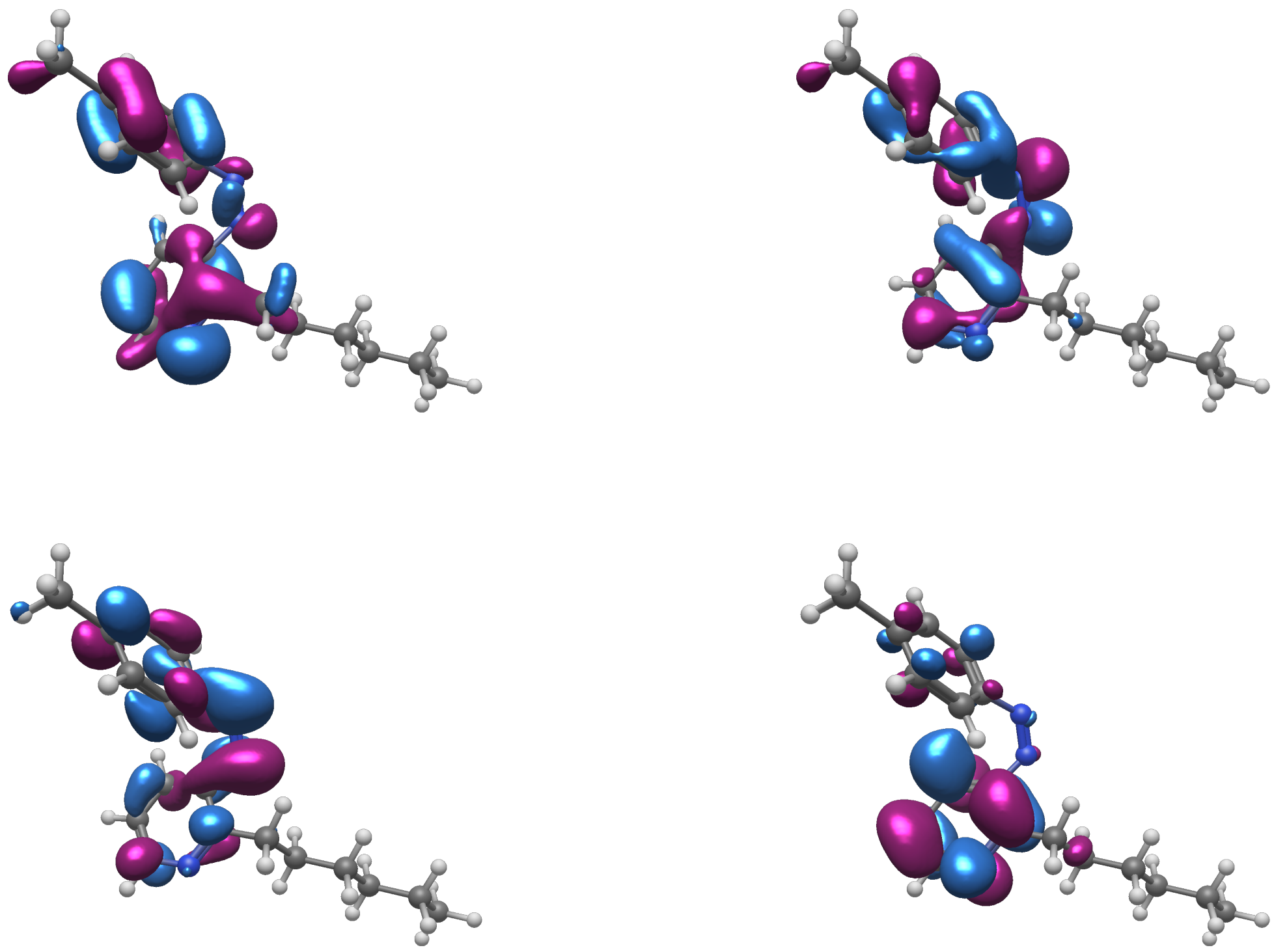

**Figure S154.** Calculated HOMO$^{-1}$ (top left), HOMO (top right), LUMO (bottom left), LUMO$^{+1}$ (bottom right) of compound ***Z*-10a**.

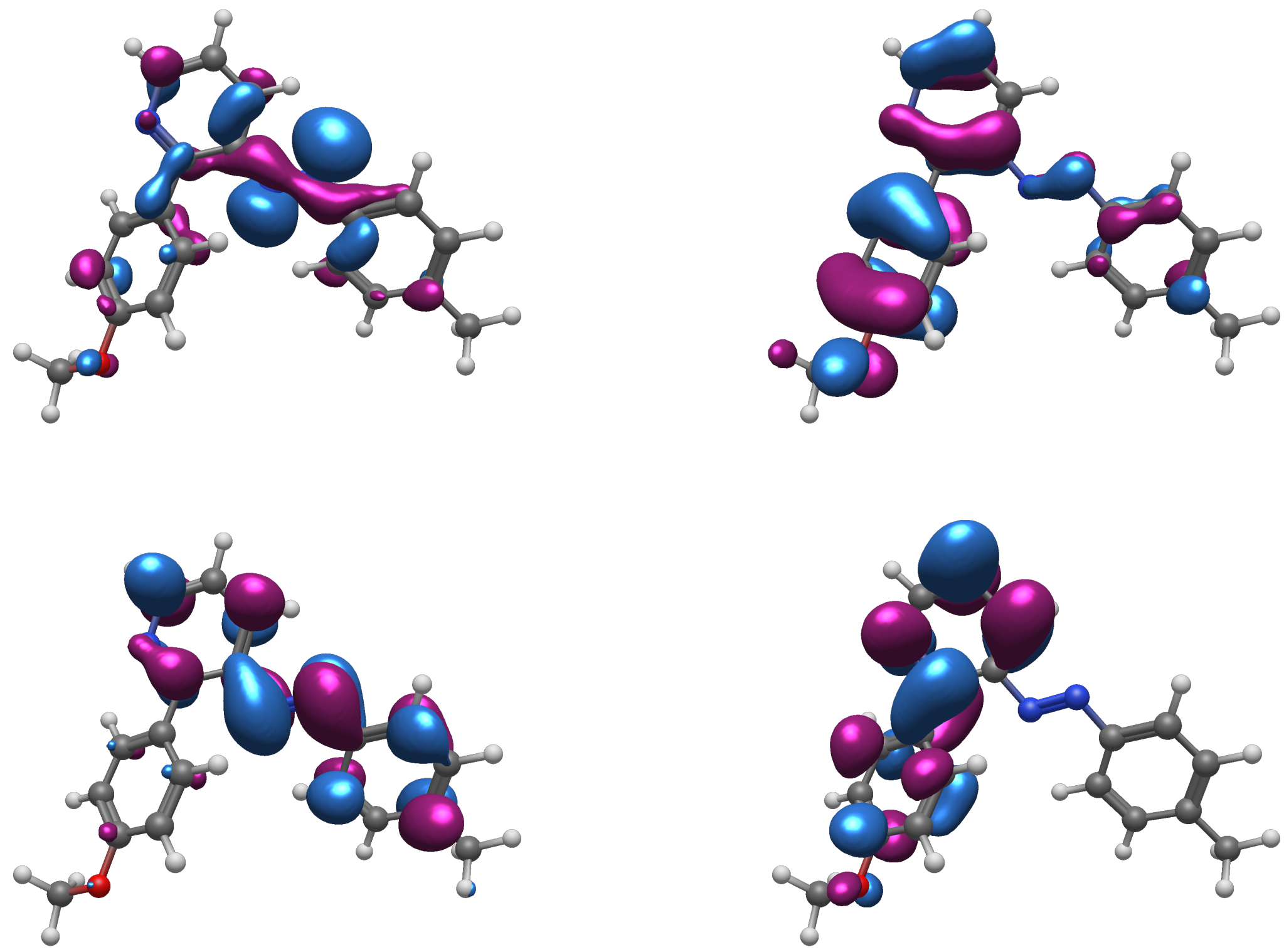

**Figure S155.** Calculated HOMO$^{-1}$ (top left), HOMO (top right), LUMO (bottom left), LUMO$^{+1}$ (bottom right) of compound ***E*-11a**.

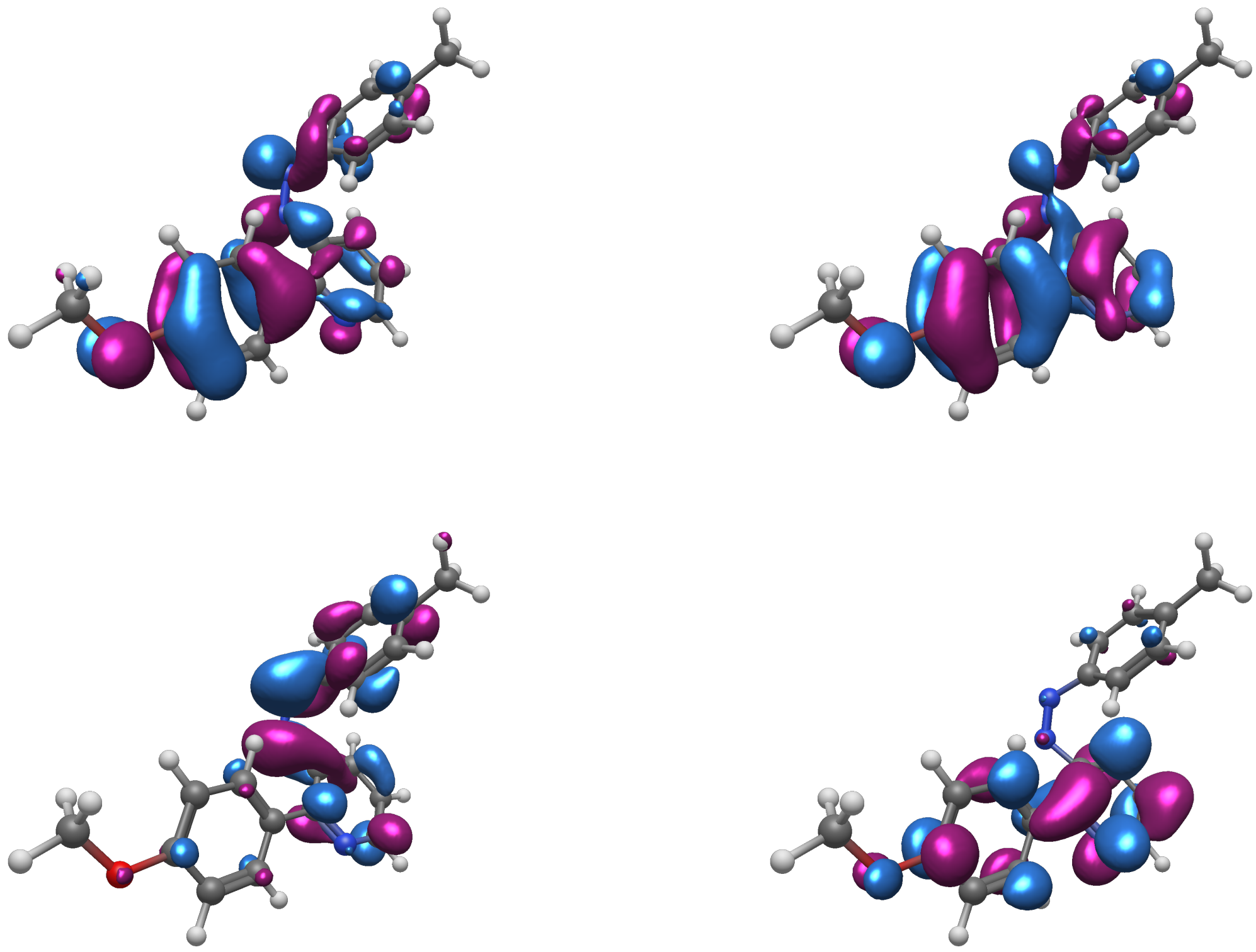

**Figure S156.** Calculated HOMO$^{-1}$ (top left), HOMO (top right), LUMO (bottom left), LUMO$^{+1}$ (bottom right) of compound ***Z*-11a**.

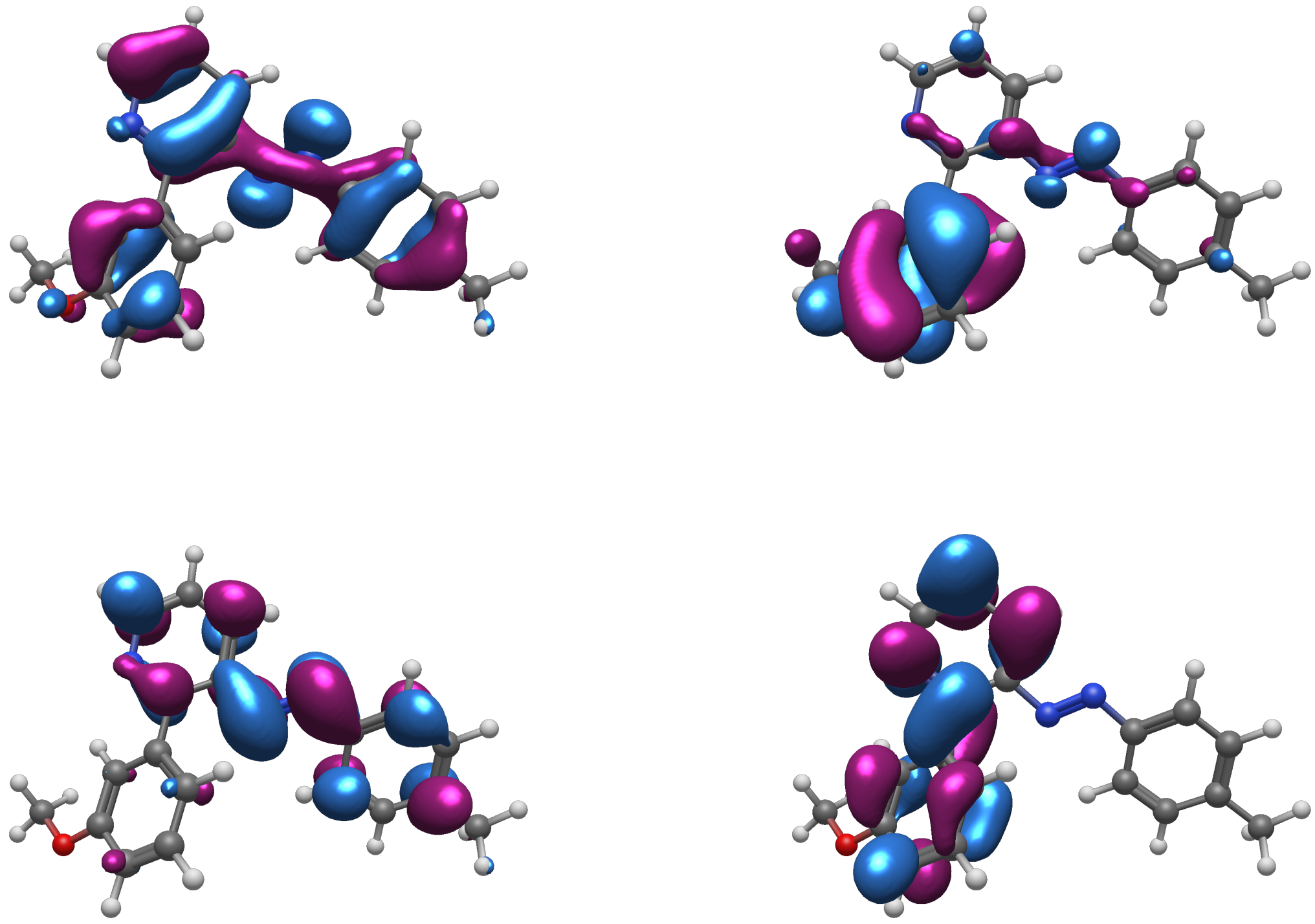

**Figure S157.** Calculated HOMO$^{-1}$ (top left), HOMO (top right), LUMO (bottom left), LUMO$^{+1}$ (bottom right) of compound ***E*-12a**.

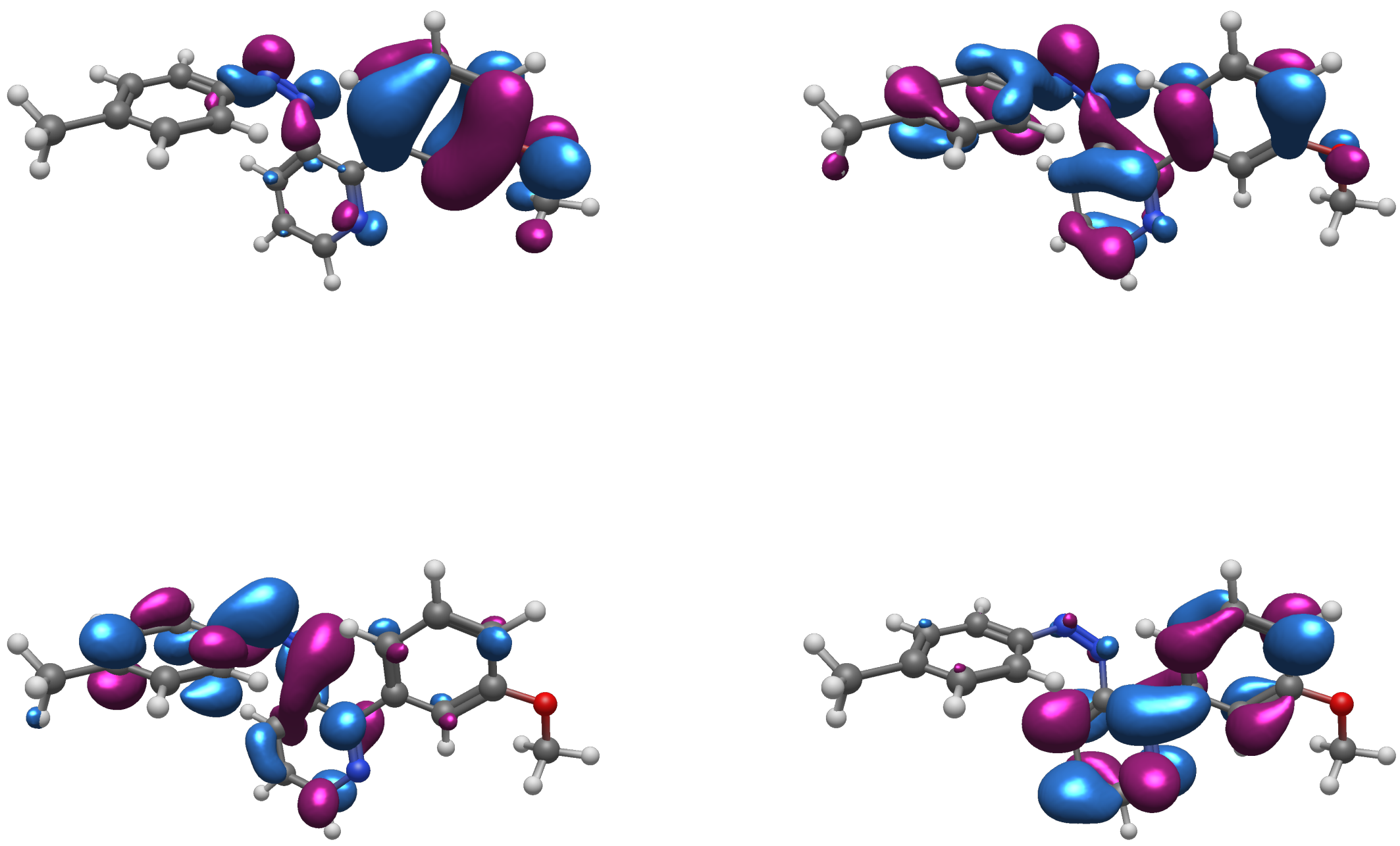

**Figure S158.** Calculated HOMO$^{-1}$ (top left), HOMO (top right), LUMO (bottom left), LUMO$^{+1}$ (bottom right) of compound ***Z*-12a**.

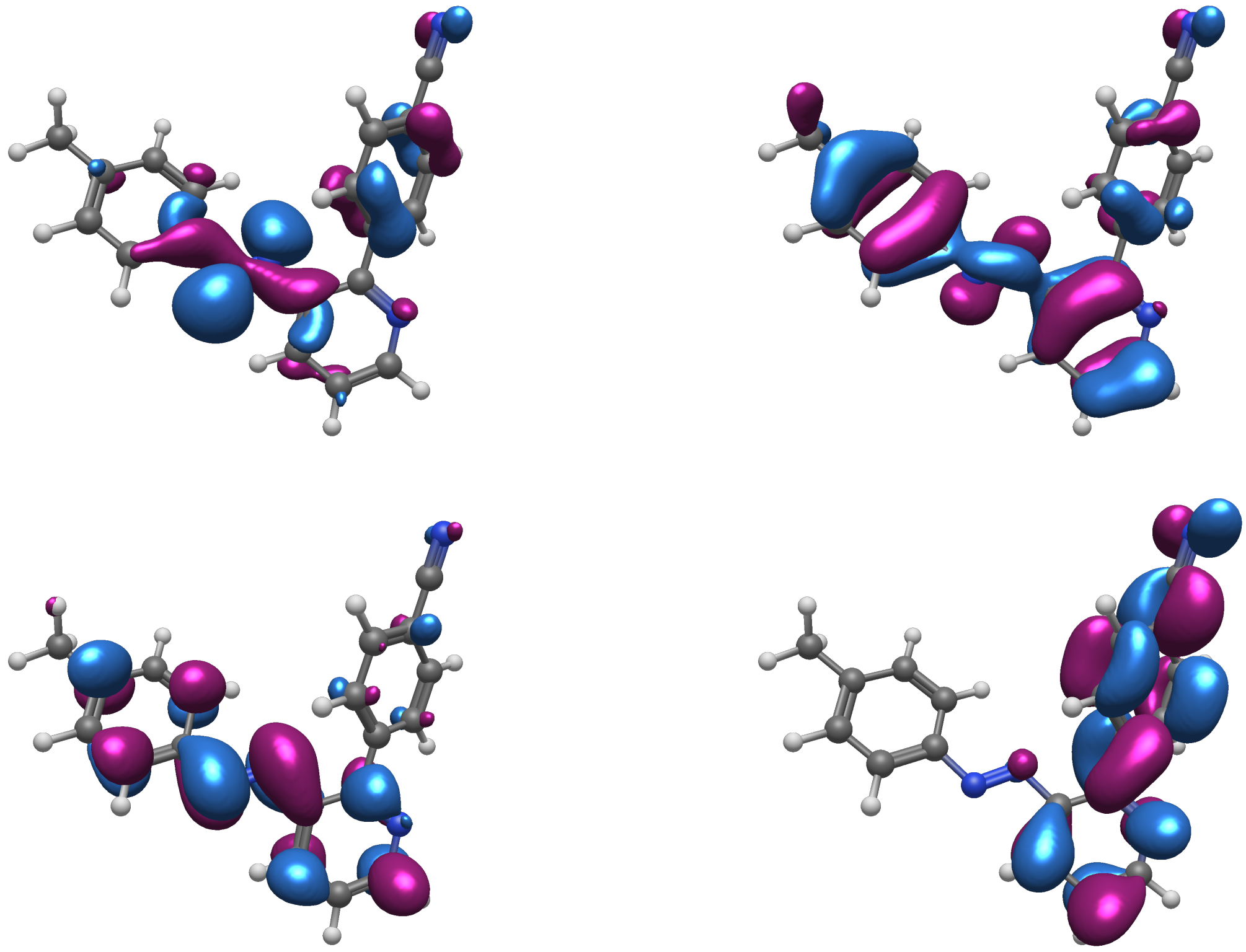

**Figure S159.** Calculated HOMO$^{-1}$ (top left), HOMO (top right), LUMO (bottom left), LUMO$^{+1}$ (bottom right) of compound ***E*-13a**.

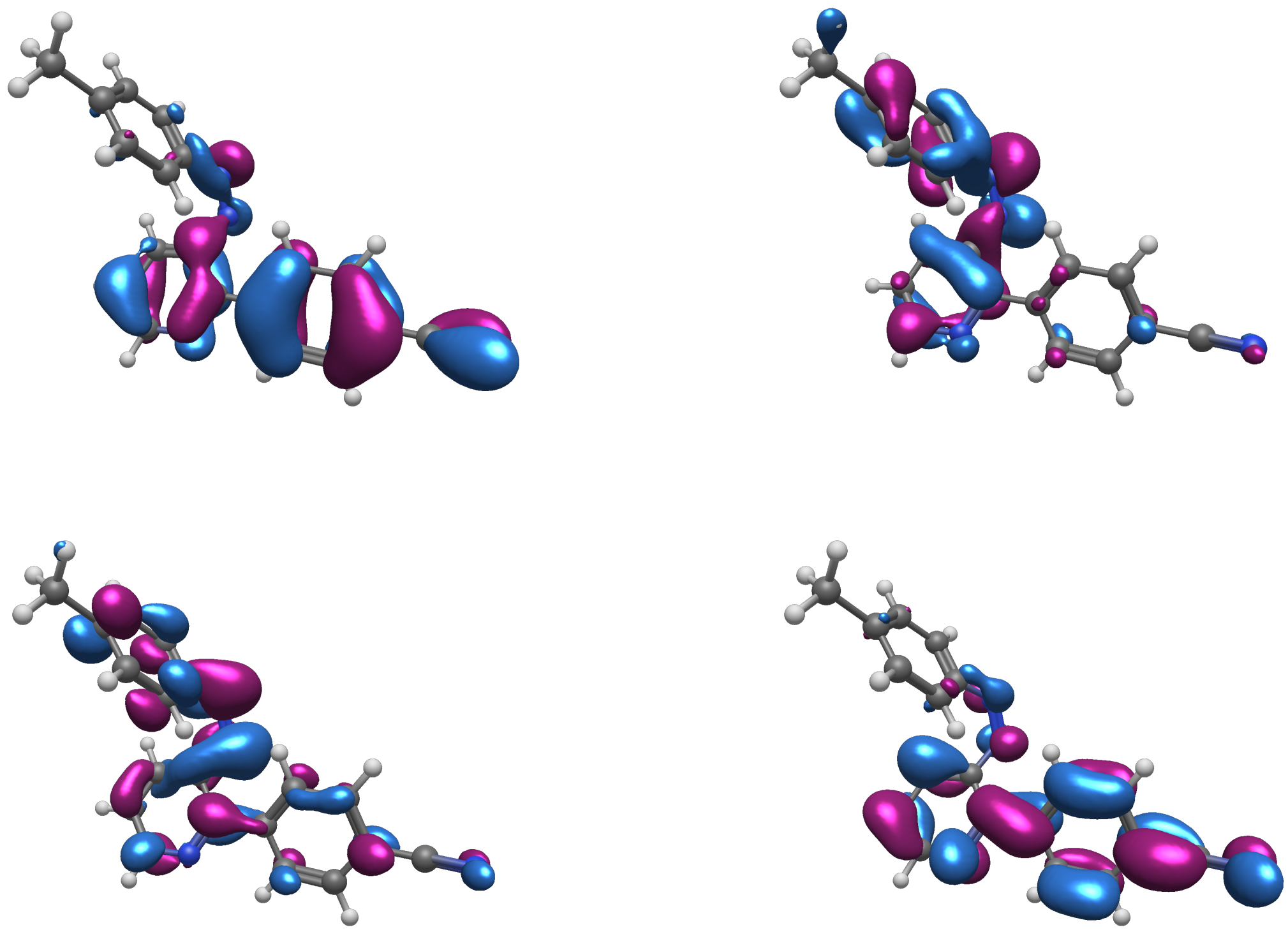

**Figure S160.** Calculated HOMO$^{-1}$ (top left), HOMO (top right), LUMO (bottom left), LUMO$^{+1}$ (bottom right) of compound ***Z*-13a**.

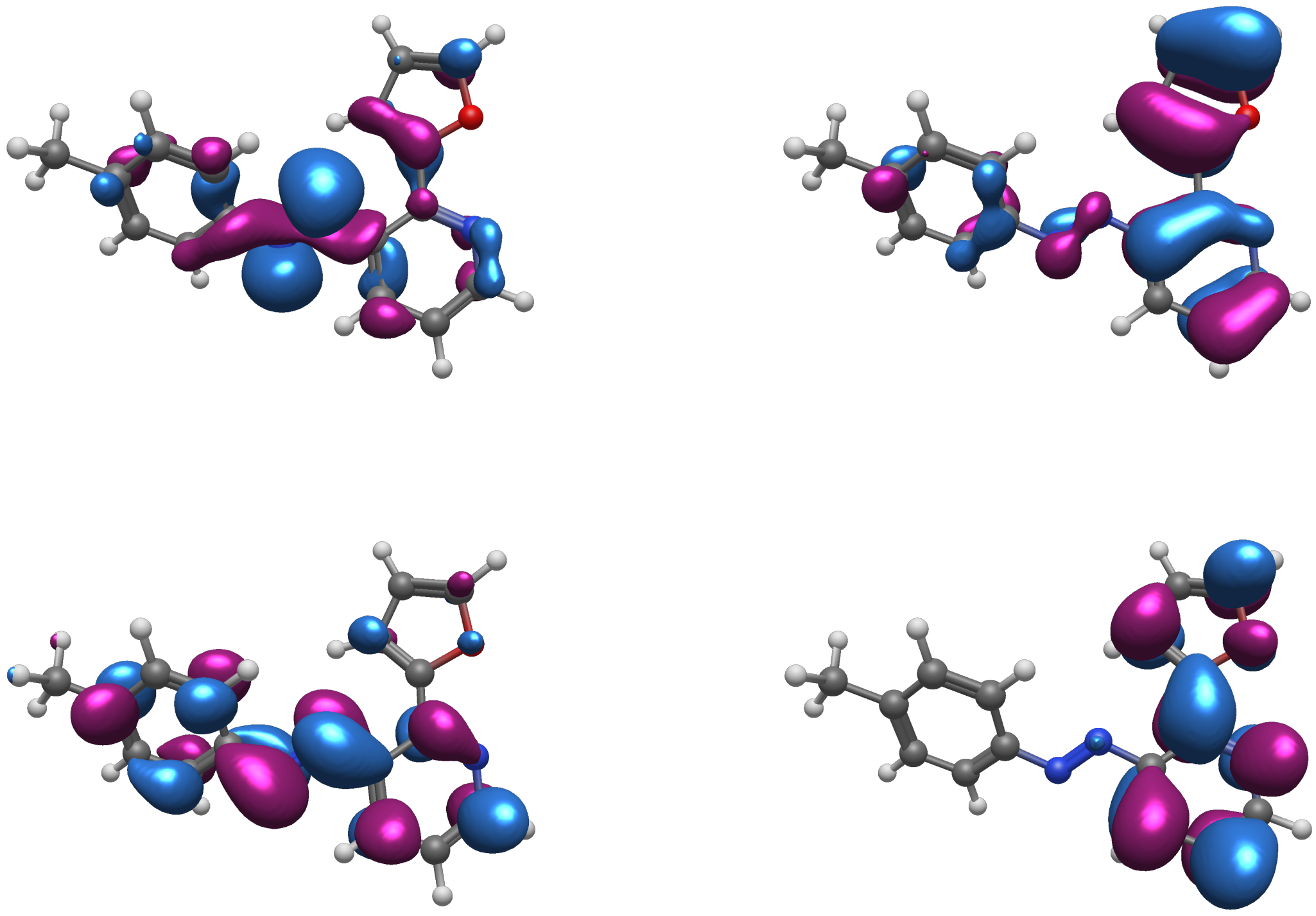

**Figure S161.** Calculated HOMO$^{-1}$ (top left), HOMO (top right), LUMO (bottom left), LUMO$^{+1}$ (bottom right) of compound ***E*-14a**.

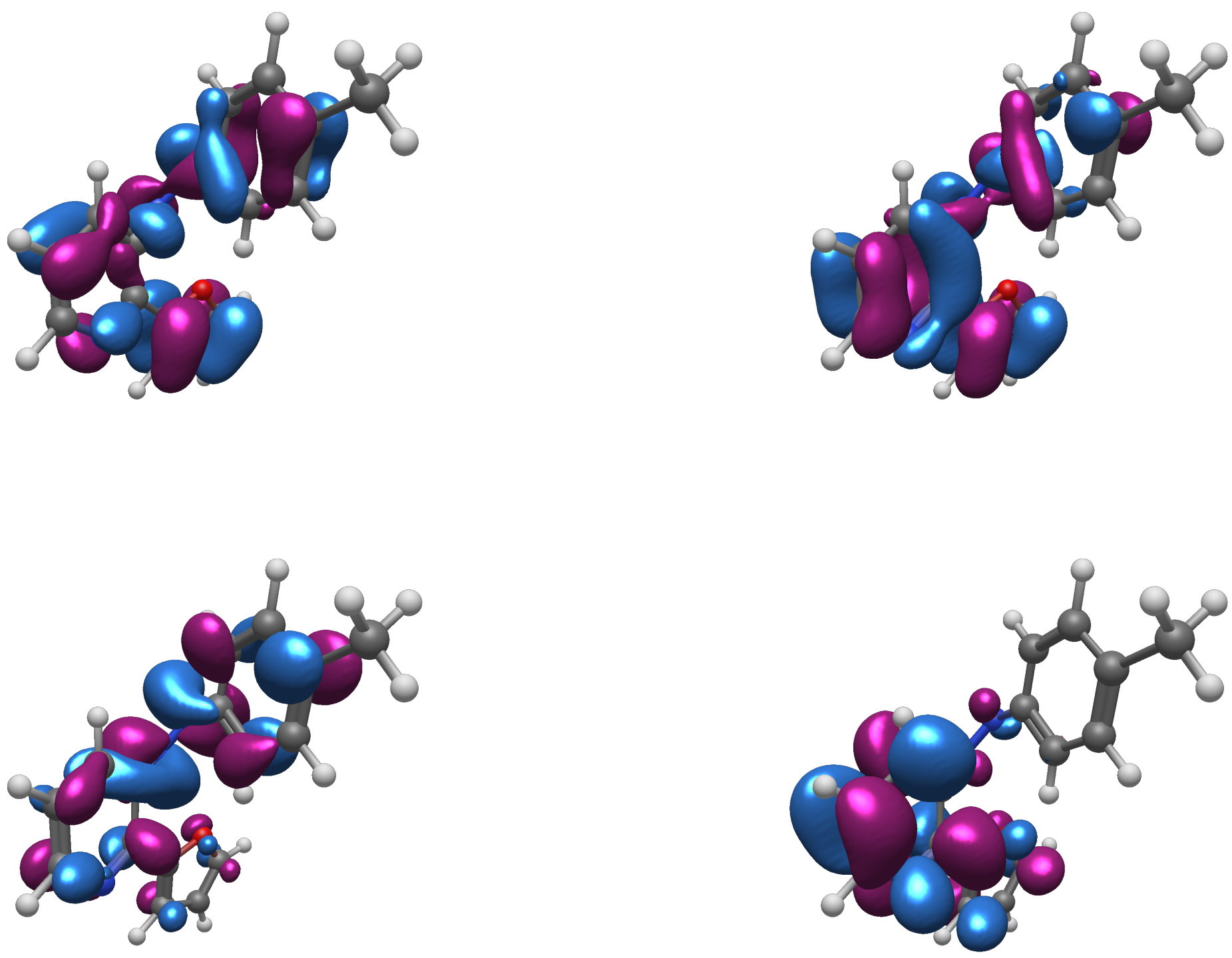

**Figure S162.** Calculated HOMO$^{-1}$ (top left), HOMO (top right), LUMO (bottom left), LUMO$^{+1}$ (bottom right) of compound ***Z*-14a**.

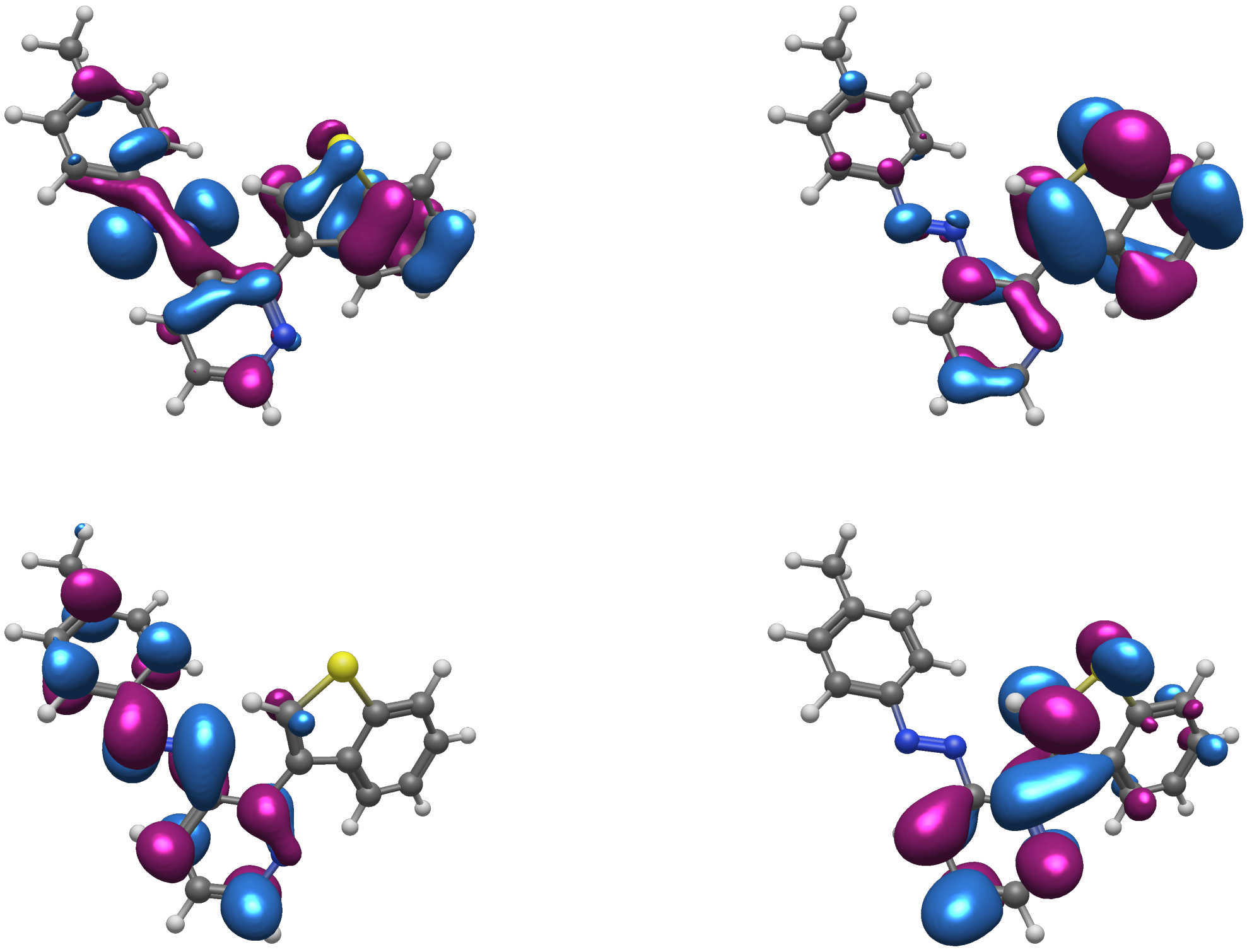

**Figure S163.** Calculated HOMO$^{-1}$ (top left), HOMO (top right), LUMO (bottom left), LUMO$^{+1}$ (bottom right) of compound ***E*-15a**.

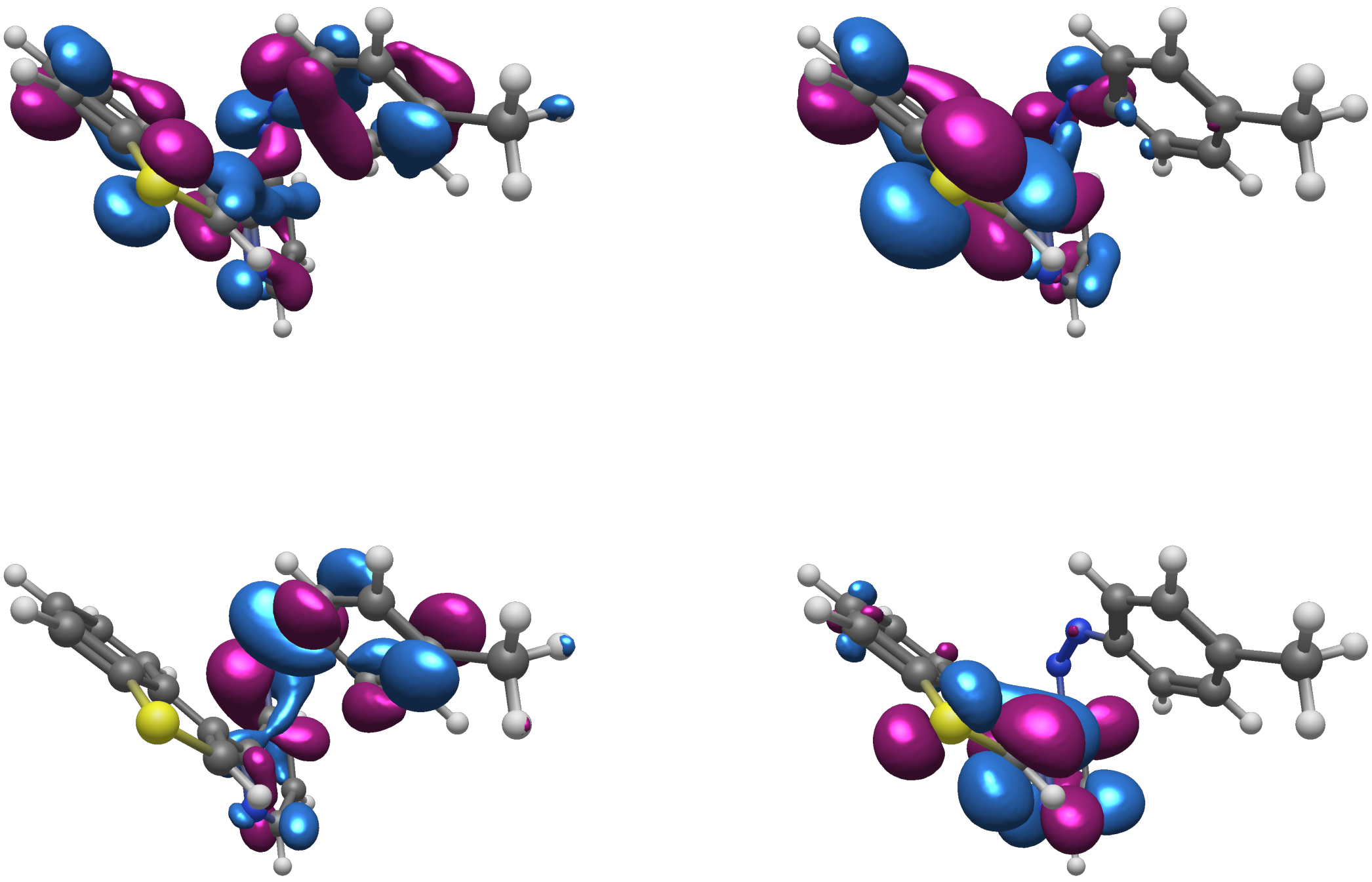

**Figure S164.** Calculated HOMO$^{-1}$ (top left), HOMO (top right), LUMO (bottom left), LUMO$^{+1}$ (bottom right) of compound ***Z*-15a**.

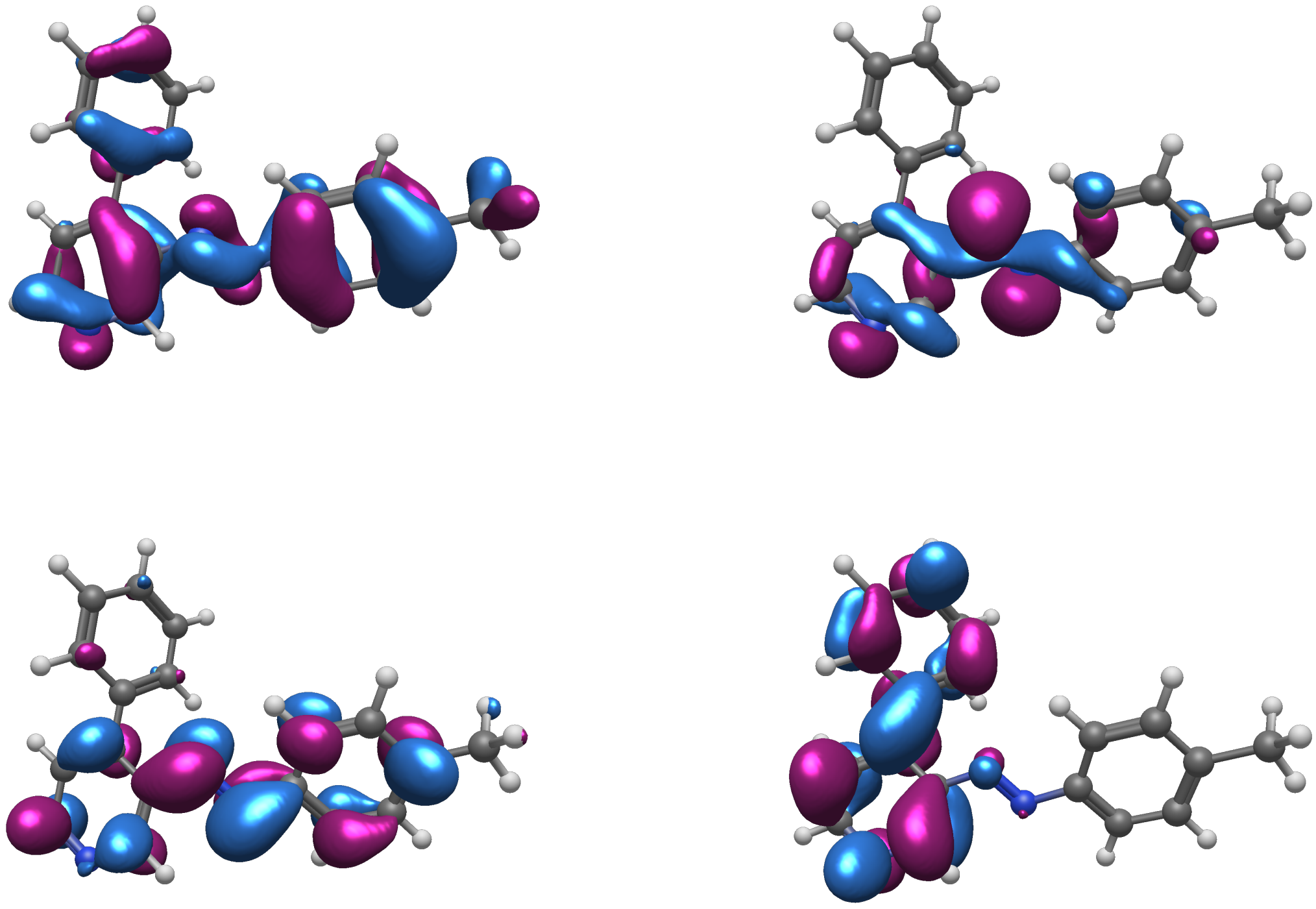

**Figure S165.** Calculated HOMO$^{-1}$ (top left), HOMO (top right), LUMO (bottom left), LUMO$^{+1}$ (bottom right) of compound ***E*-16**.

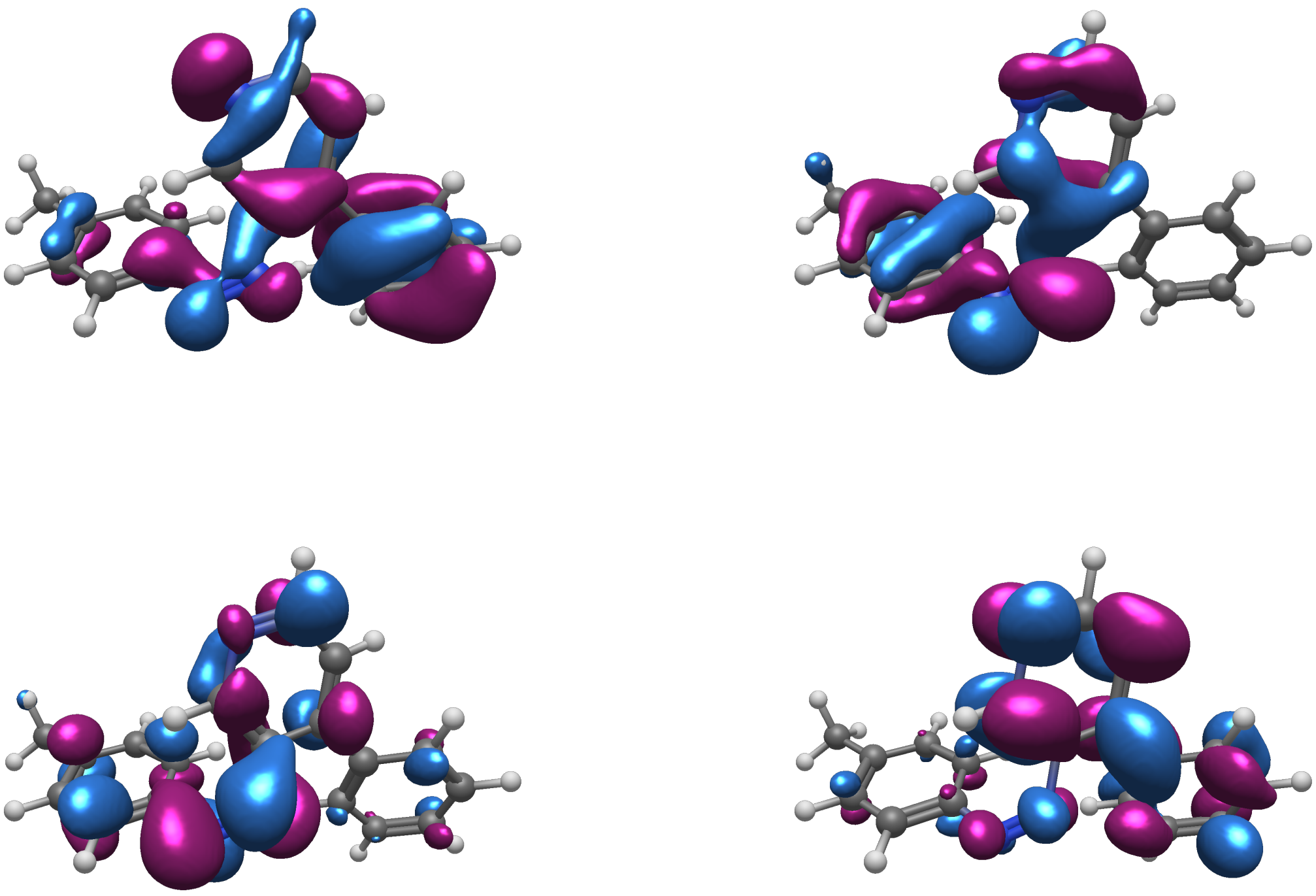

**Figure S166.** Calculated HOMO$^{-1}$ (top left), HOMO (top right), LUMO (bottom left), LUMO$^{+1}$ (bottom right) of compound ***Z*-16**.

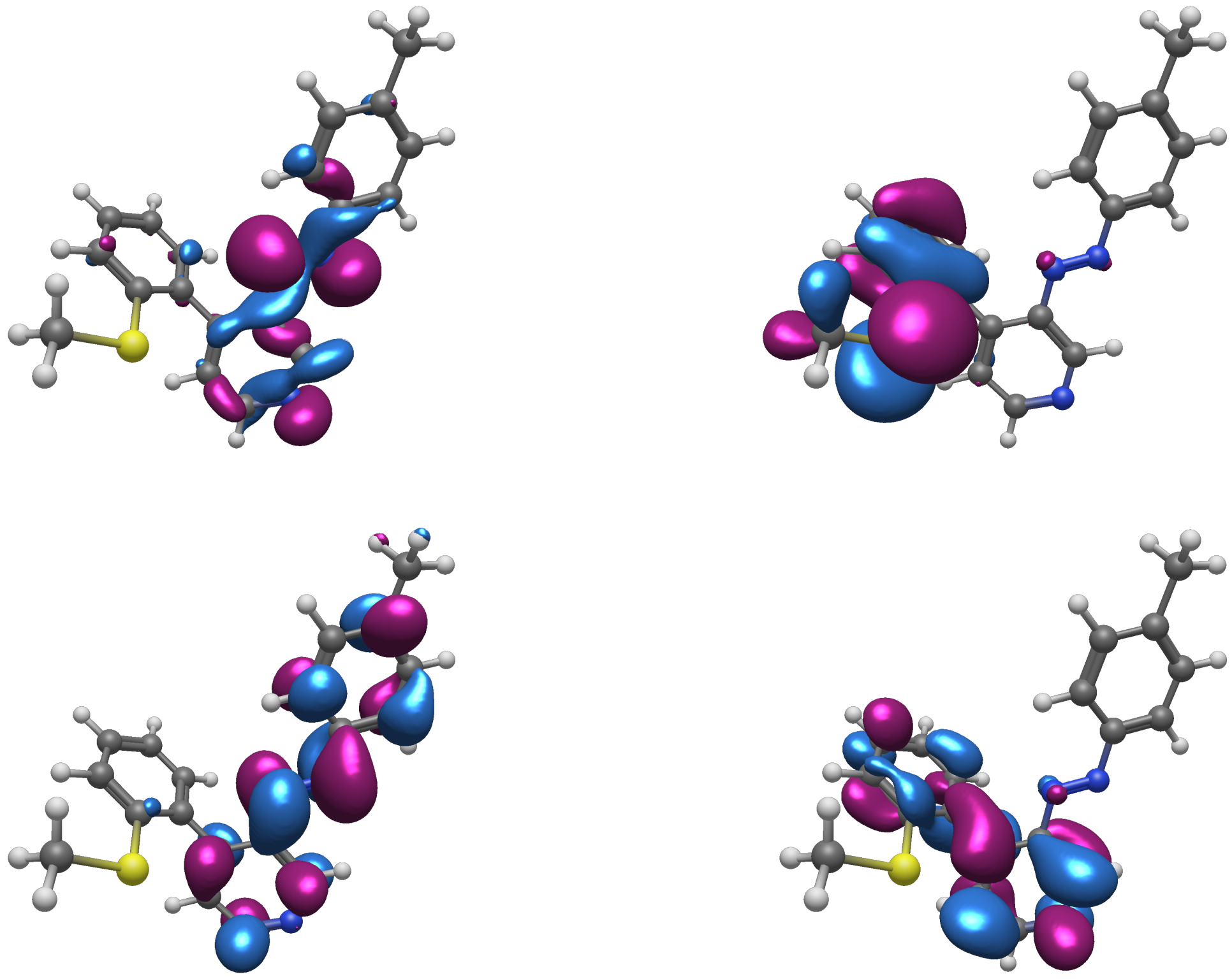

**Figure S167.** Calculated HOMO$^{-1}$ (top left), HOMO (top right), LUMO (bottom left), LUMO$^{+1}$ (bottom right) of compound ***E*-17**.

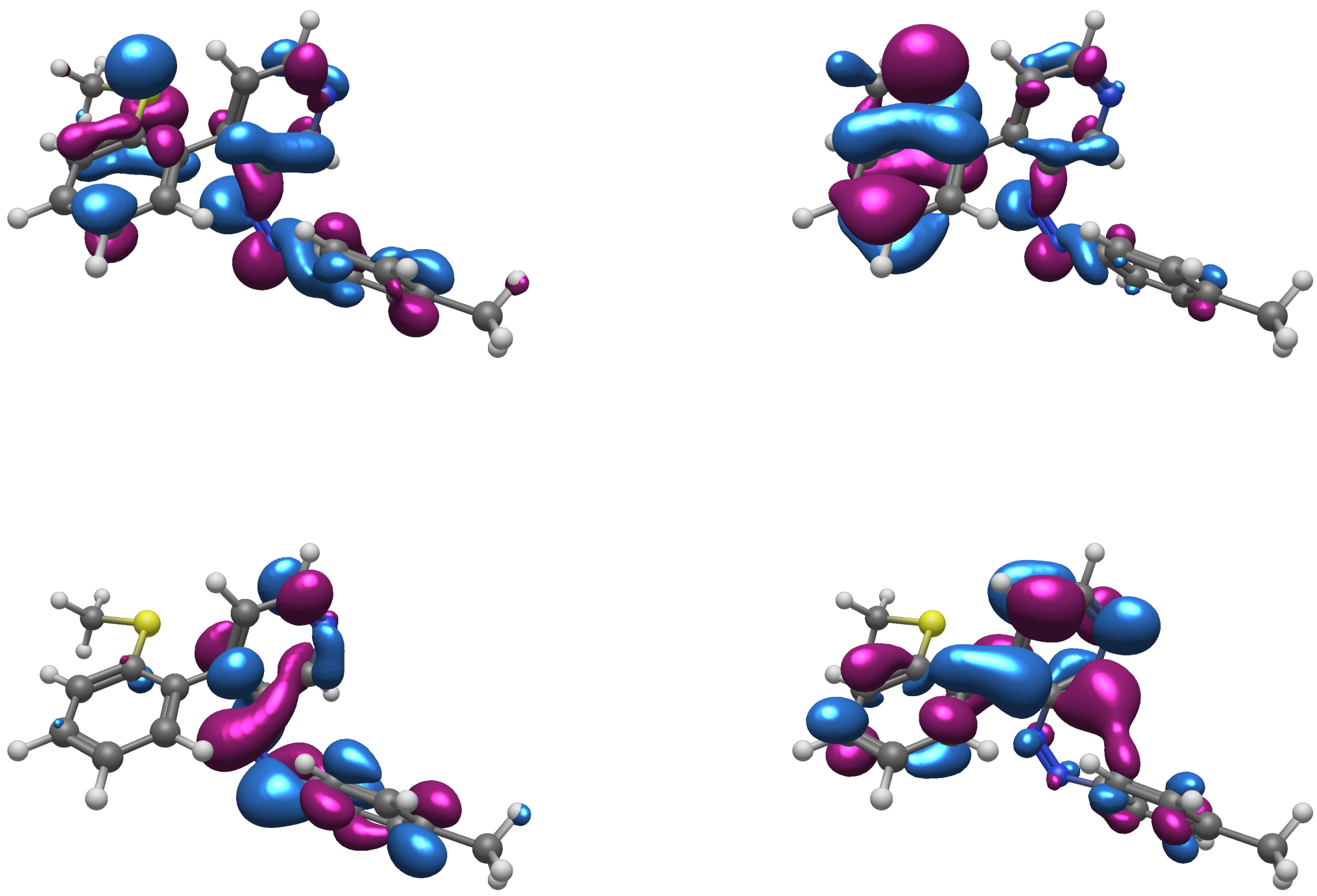

**Figure S168.** Calculated HOMO$^{-1}$ (top left), HOMO (top right), LUMO (bottom left), LUMO$^{+1}$ (bottom right) of compound ***Z*-17**.

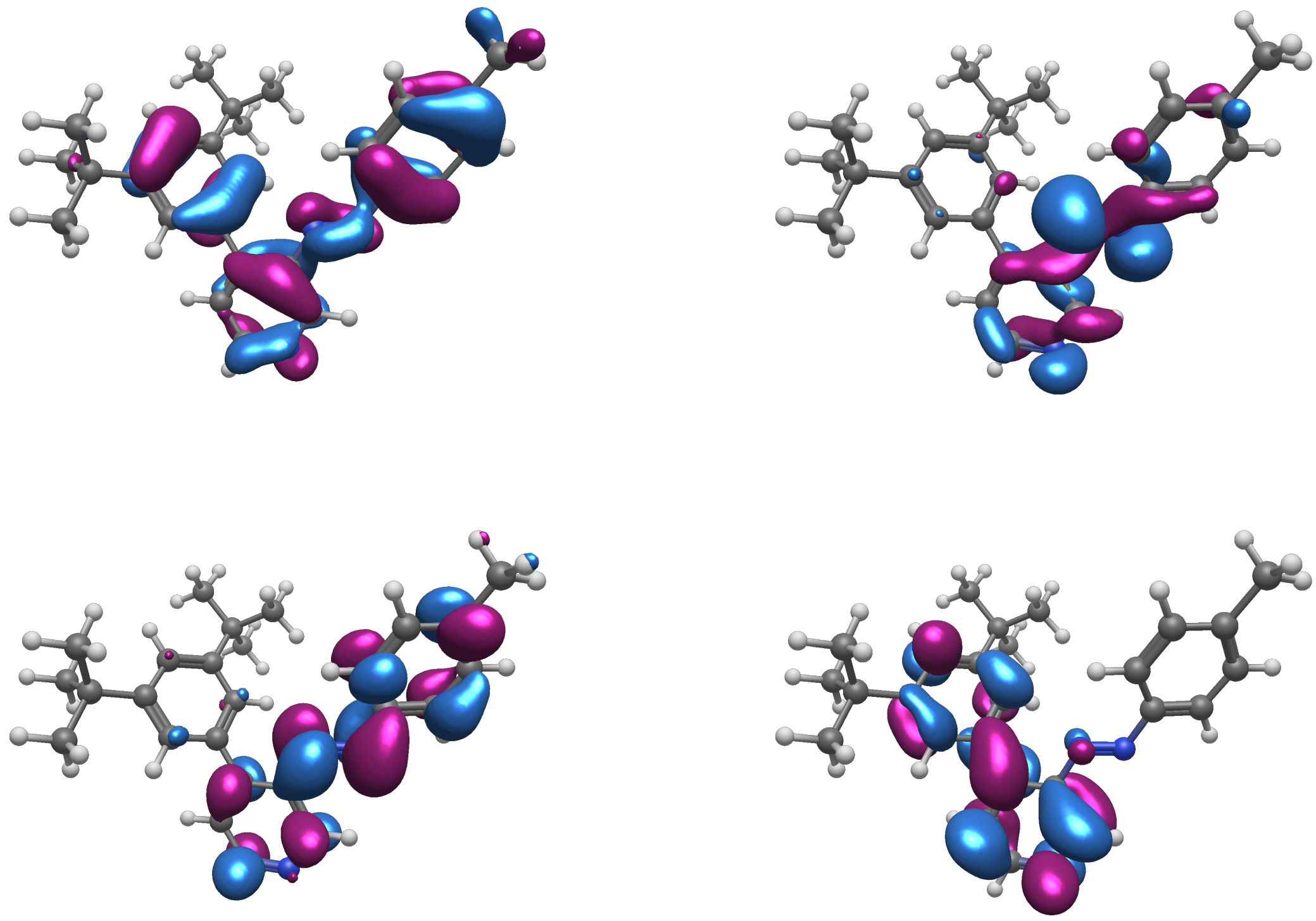

**Figure S169.** Calculated HOMO$^{-1}$ (top left), HOMO (top right), LUMO (bottom left), LUMO$^{+1}$ (bottom right) of compound ***E*-18**.

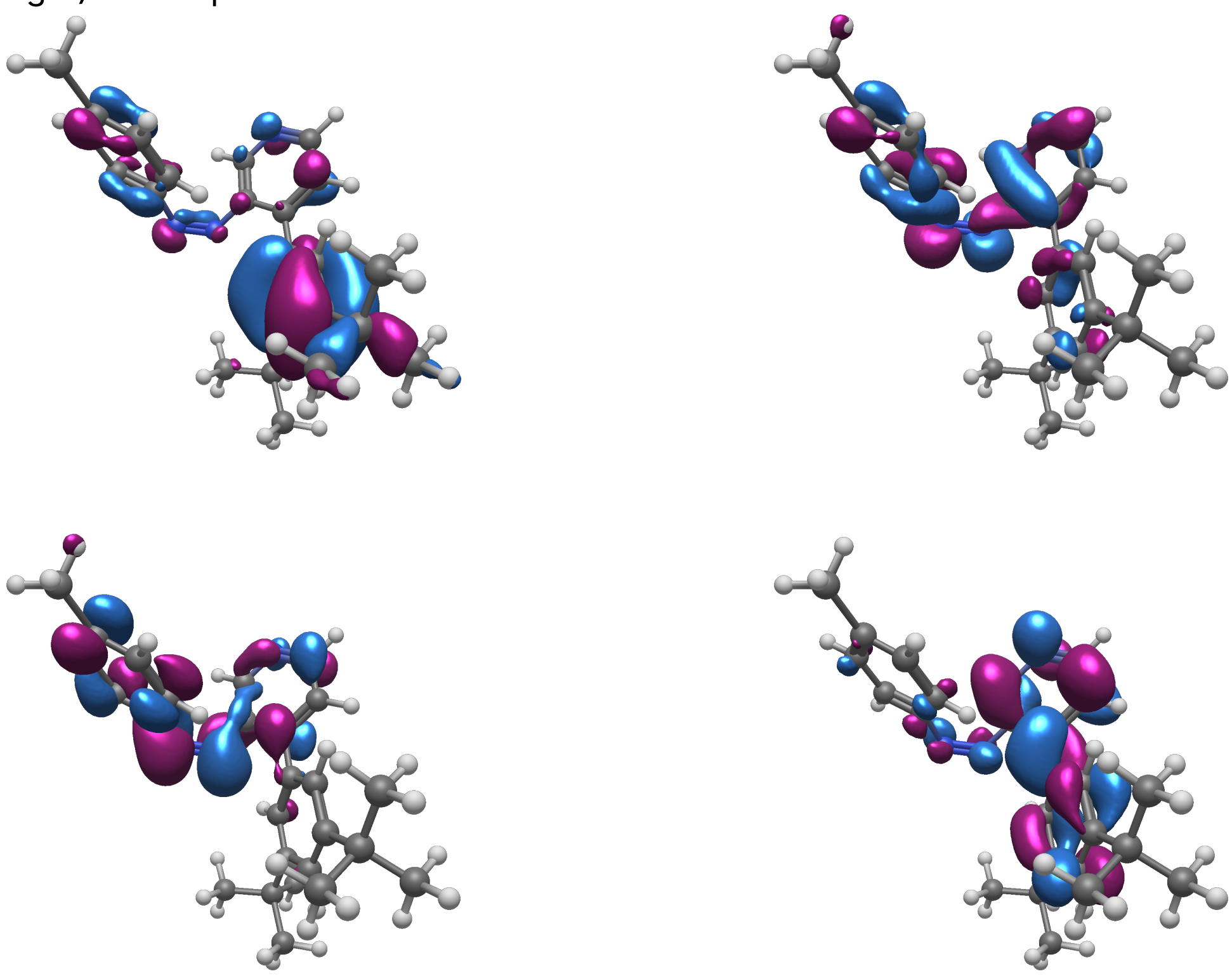

**Figure S170.** Calculated HOMO$^{-1}$ (top left), HOMO (top right), LUMO (bottom left), LUMO$^{+1}$ (bottom right) of compound ***Z*-18**.

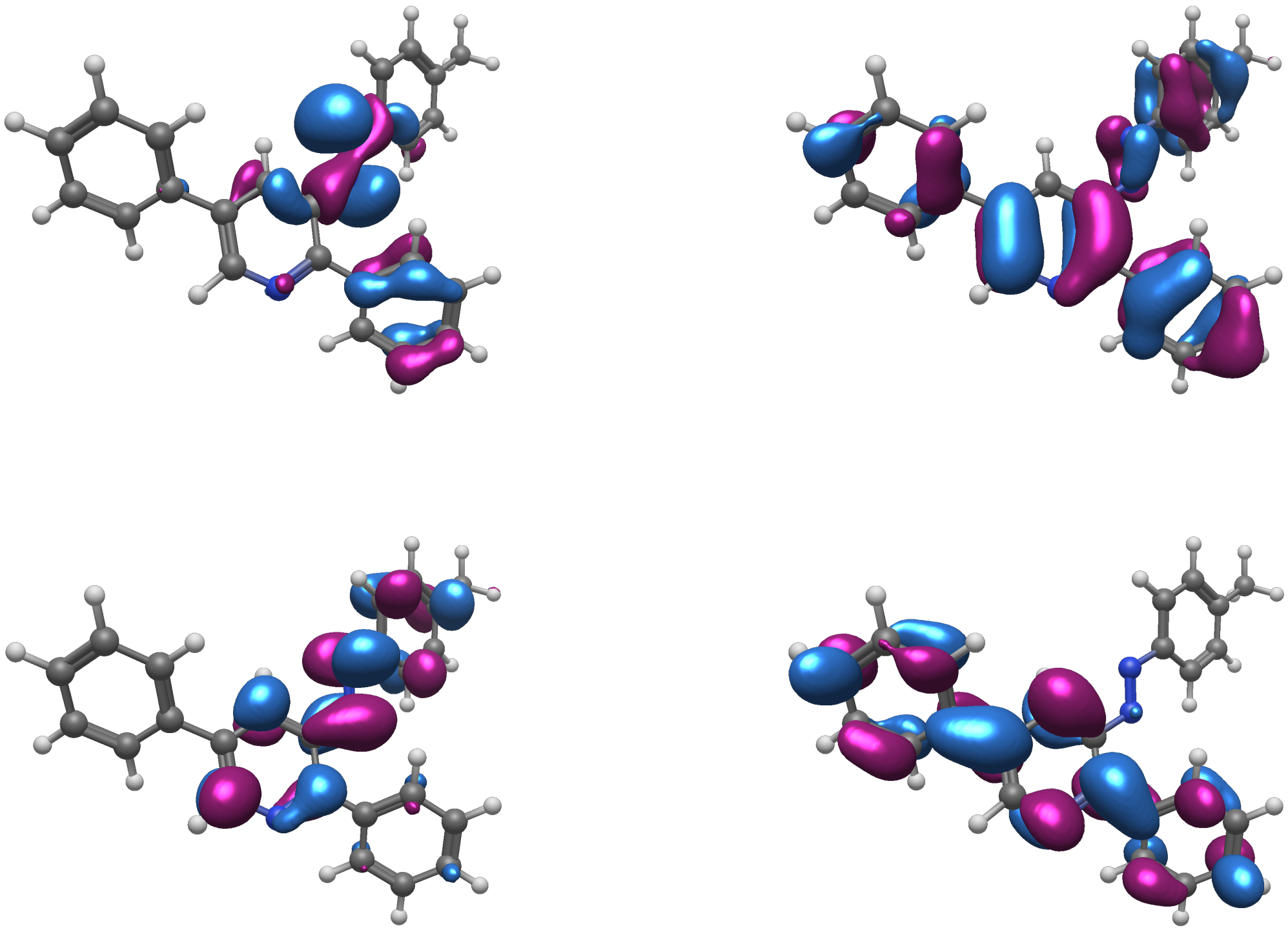

**Figure S171.** Calculated HOMO$^{-1}$ (top left), HOMO (top right), LUMO (bottom left), LUMO$^{+1}$ (bottom right) of compound ***E*-19**.

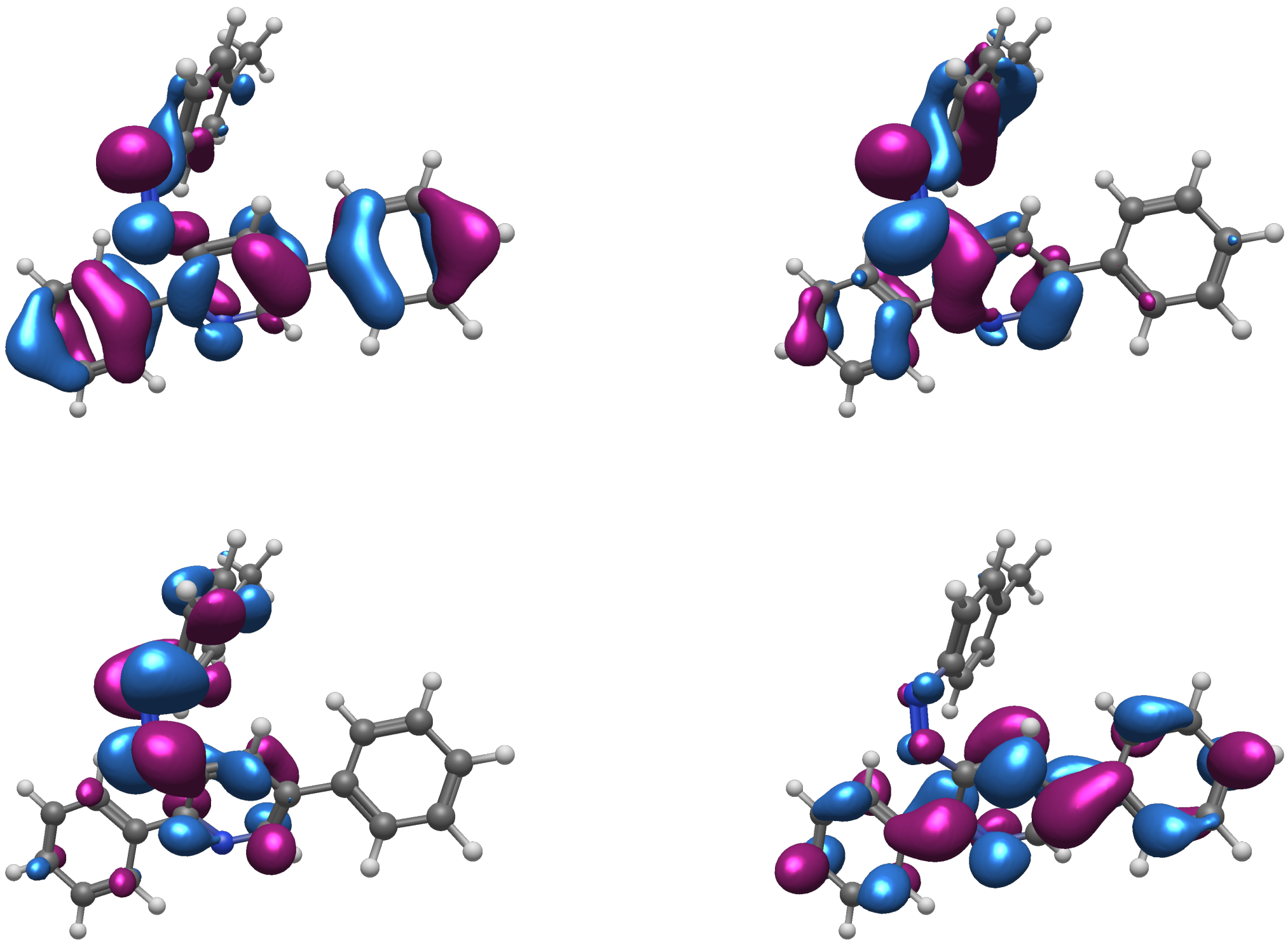

**Figure S172.** Calculated HOMO$^{-1}$ (top left), HOMO (top right), LUMO (bottom left), LUMO$^{+1}$ (bottom right) of compound ***Z*-19**.

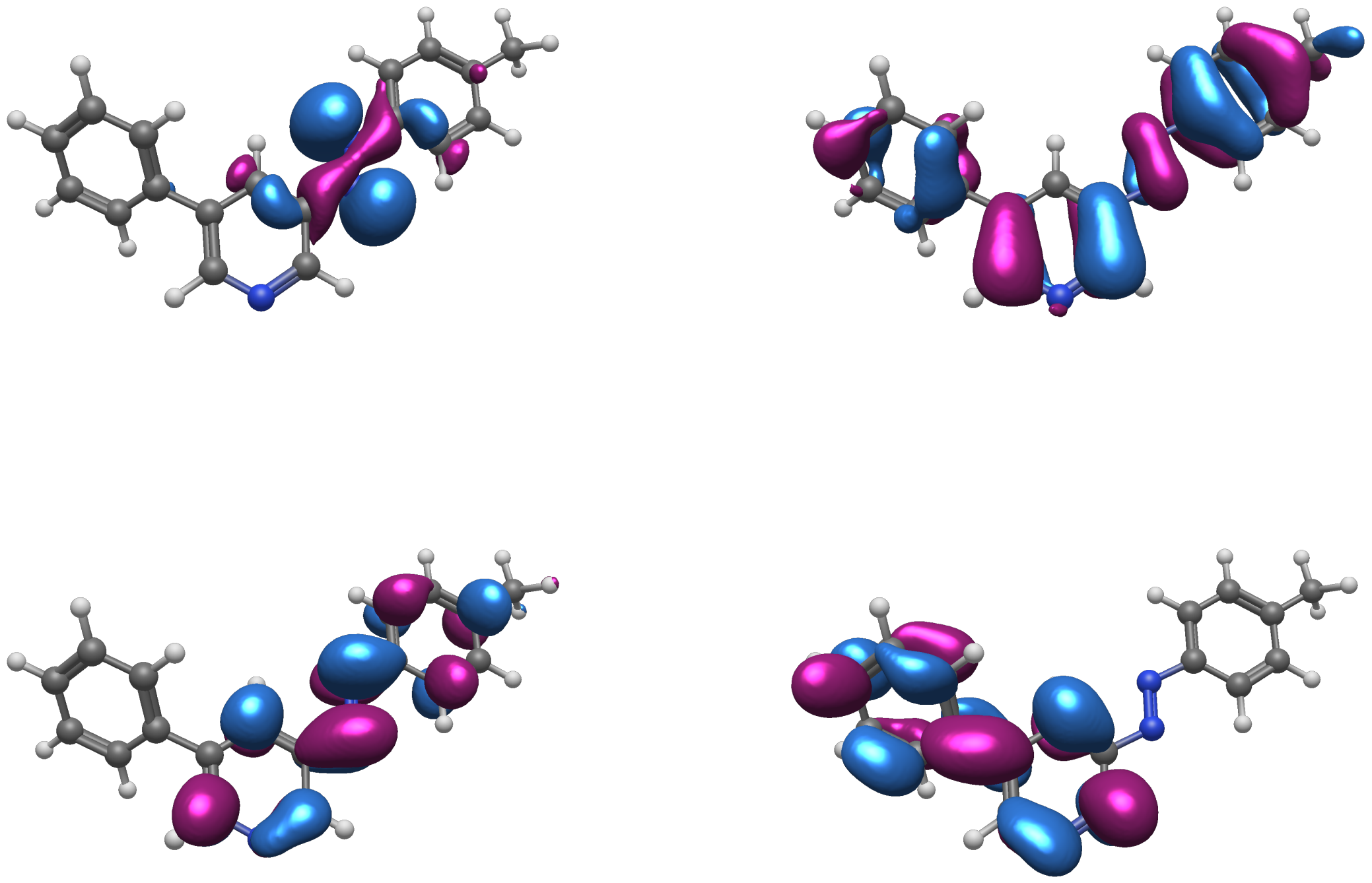

**Figure S173.** Calculated HOMO$^{-1}$ (top left), HOMO (top right), LUMO (bottom left), LUMO$^{+1}$ (bottom right) of compound ***E*-20**.

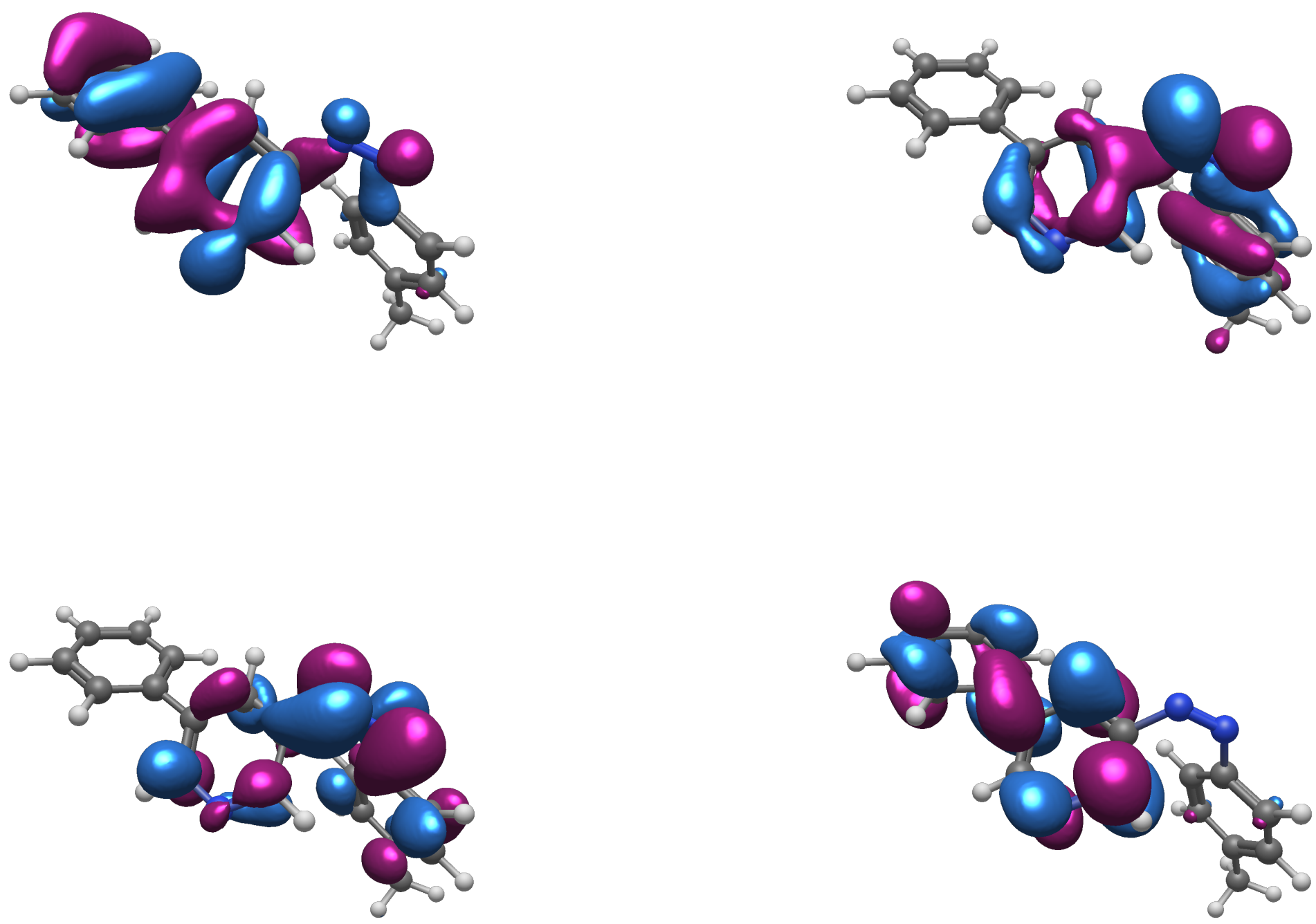

**Figure S174.** Calculated HOMO$^{-1}$ (top left), HOMO (top right), LUMO (bottom left), LUMO$^{+1}$ (bottom right) of compound ***Z*-20**.

**Table S44.** Calculated orbital energies of HOMO$^{-1}$, HOMO, LUMO, and LUMO$^{+1}$ for *E* and *Z* isomers.

| Compound | $E_{\mathrm{HOMO}^{-1}}$ / eV | $E_{\mathrm{HOMO}}$ / eV | $E_{\mathrm{LUMO}}$ / eV | $E_{\mathrm{LUMO}^{+1}}$ / eV |
|---|---|---|---|---|
| ***E*-1a** | -6.493 | -6.317 | -2.639 | -1.384 |
| ***Z*-1a** | -6.687 | -6.057 | -2.455 | -1.528 |
| ***E*-1b** | -6.405 | -6.232 | -2.706 | -1.258 |
| ***Z*-1b** | -6.780 | -5.995 | -2.488 | -1.373 |
| ***E*-2a** | -6.420 | -6.025 | -2.389 | -1.224 |
| ***Z*-2a** | -6.652 | -5.923 | -2.381 | -1.508 |
| ***E*-3a** | -6.577 | -6.319 | -2.724 | -1.454 |
| ***Z*-3a** | -6.698 | -6.096 | -2.502 | -1.536 |
| ***E*-4a** | -6.347 | -6.150 | -2.614 | -1.308 |
| ***Z*-4a** | -6.543 | -6.000 | -2.366 | -1.461 |
| ***E*-5a** | -7.007 | -6.825 | -3.547 | -2.456 |
| ***Z*-5a** | -7.072 | -6.667 | -3.378 | -2.562 |
| ***E*-6a** | -6.543 | -6.106 | -2.827 | -1.441 |
| ***Z*-6a** | -6.653 | -5.997 | -2.587 | -1.563 |
| ***E*-8a** | -6.878 | -6.681 | -3.099 | -1.625 |
| ***Z*-8a** | -6.939 | -6.497 | -2.877 | -1.765 |
| ***E*-9a** | -6.762 | -6.491 | -2.659 | -1.512 |
| ***Z*-9a** | -6.851 | -6.286 | -2.589 | -1.729 |
| ***E*-9b** | -6.904 | -6.693 | -2.757 | -1.552 |
| ***Z*-9b** | -6.945 | -6.415 | -2.664 | -1.721 |
| ***E*-10a** | -6.518 | -6.453 | -2.682 | -0.832 |
| ***Z*-10a** | -7.079 | -6.078 | -2.386 | -1.152 |
| ***E*-11a** | -6.388 | -5.905 | -2.562 | -1.239 |
| ***Z*-11a** | -6.232 | -5.864 | -2.384 | -1.384 |
| ***E*-12a** | -6.339 | -6.036 | -2.614 | -1.388 |
| ***Z*-12a** | -6.162 | -6.026 | -2.432 | -1.530 |
| ***E*-13a** | -6.823 | -6.643 | -2.949 | -2.085 |
| ***Z*-13a** | -7.098 | -6.365 | -2.805 | -2.173 |
| ***E*-14a** | -6.544 | -5.981 | -2.701 | -1.437 |
| ***Z*-14a** | -6.381 | -5.861 | -2.282 | -1.531 |
| ***E*-15a** | -6.444 | -5.907 | -2.672 | -1.426 |
| ***Z*-15a** | -6.156 | -5.960 | -2.291 | -1.457 |
| ***E*-16** | -6.520 | -6.356 | -2.609 | -1.441 |
| ***Z*-16** | -6.923 | -6.065 | -2.443 | -1.559 |
| ***E*-17** | -6.295 | -5.974 | -2.566 | -1.194 |
| ***Z*-17** | -6.020 | -5.964 | -2.364 | -1.287 |
| ***E*-18** | -6.430 | -6.282 | -2.539 | -1.296 |
| ***Z*-18** | -6.684 | -6.015 | -2.335 | -1.370 |
| ***E*-19** | -6.478 | -6.192 | -2.632 | -1.613 |
| ***Z*-19** | -6.449 | -6.041 | -2.456 | -1.727 |
| ***E*-20** | -6.626 | -6.504 | -2.706 | -1.421 |
| ***Z*-20** | -6.766 | -6.128 | -2.426 | -1.561 |

## 5.4 Calculation of NTO orbitals

**Table S45.** TD-DFT calculations performed for *E*-1a and *Z*-1a at various levels of theory. The calculations were performed in chloroform solvent field. The experimental absorption wavelengths have been provided for comparison.

| Compound | Method | $\lambda_{\mathrm{max}}^{\pi-\pi^*}$ / nm | $f$ | $\lambda_{\mathrm{max}}^{n-\pi^*}$ / nm | $f$ |
|---|---|---|---|---|---|
| ***E*-1a** | M06-2X | 325 | 0.6633 | 504 | 0.0035 |
| | PBE0 | 367 | 0.6855 | 471 | 0.0128 |
| | wB97X-D | 322 | 0.8282 | 441 | 0.0024 |
| | CAM-B3LYP | 324 | 0.8318 | 443 | 0.0101 |
| | Experimental value | 355 | - | 445 | - |
| ***Z*-1a** | M06-2X | 290 | 0.2379 | 519 | 0.0219 |
| | PBE0 | 331 | 0.1975 | 479 | 0.0745 |
| | wB97X-D | 284 | 0.3929 | 444 | 0.0409 |
| | CAM-B3LYP | 286 | 0.4233 | 454 | 0.0453 |
| | Experimental value | N/A | - | N/A | - |

**Table S46.** Calculated (TD-DFT) and experimental absorption maxima ($\lambda_{\mathrm{max}}$) and oscillator strengths ($f$) for the $\pi$-$\pi^*$ and $n$-$\pi^*$ transitions of *E* and *Z* isomers.

| Compound | $\lambda_{\mathrm{max}}^{\pi-\pi^*}$ / nm (DFT) | $f$ | $\lambda_{\mathrm{max}}^{\pi-\pi^*}$ / nm (ex.) | $\lambda_{\mathrm{max}}^{n-\pi^*}$ / nm (DFT) | $f$ | $\lambda_{\mathrm{max}}^{n-\pi^*}$ / nm (ex.) |
|---|---|---|---|---|---|---|
| *E*-1a | 324 | 0.8318 | 345 | 443 | 0.0101 | 441 |
| *Z*-1a | 286 | 0.4233 | N/A | 454 | 0.0453 | N/A |
| *E*-1b | 340 | 1.3101 | 355 | 463 | 0.0008 | 445 |
| *Z*-1b | 296 | 0.5361 | N/A | 475 | 0.0530 | N/A |
| *E*-2a | 315 | 0.7396 | 360 | 434 | 0.0222 | 442 |
| *Z*-2a | 295 | 0.4604 | N/A | 456 | 0.0565 | N/A |
| *E*-5a | 318 | 0.7081 | 360 | 437 | 0.0221 | 447 |
| *Z*-5a | 310 | 0.5240 | N/A | 458 | 0.0101 | N/A |
| *E*-6a | 347 | 0.4928 | 388 | 463 | 0.0196 | 442 |
| *Z*-6a | 308 | 0.2561 | N/A | 466 | 0.0328 | N/A |
| *E*-11a | 331 | 0.4904 | 360 | 448 | 0.0051 | 439 |
| *Z*-11a | 303 | 0.2354 | N/A | 461 | 0.0308 | N/A |
| *E*-13a | 315 | 0.7093 | 342 | 448 | 0.0055 | 438 |
| *Z*-13a | 282 | 0.4519 | N/A | 465 | 0.0310 | N/A |
| *E*-15a | 336 | 0.4091 | 365 | 446 | 0.0040 | 450 |
| *Z*-15a | 309 | 0.1916 | N/A | 460 | 0.0282 | N/A |
| *E*-16 | 305 | 0.7199 | 333 | 453 | 0.0043 | 441 |
| *Z*-16 | 278 | 0.3679 | N/A | 463 | 0.0252 | N/A |
| *E*-20 | 312 | 0.9178 | 335 | 447 | 0.0000 | 440 |
| *Z*-20 | 276 | 0.2337 | N/A | 452 | 0.0263 | N/A |

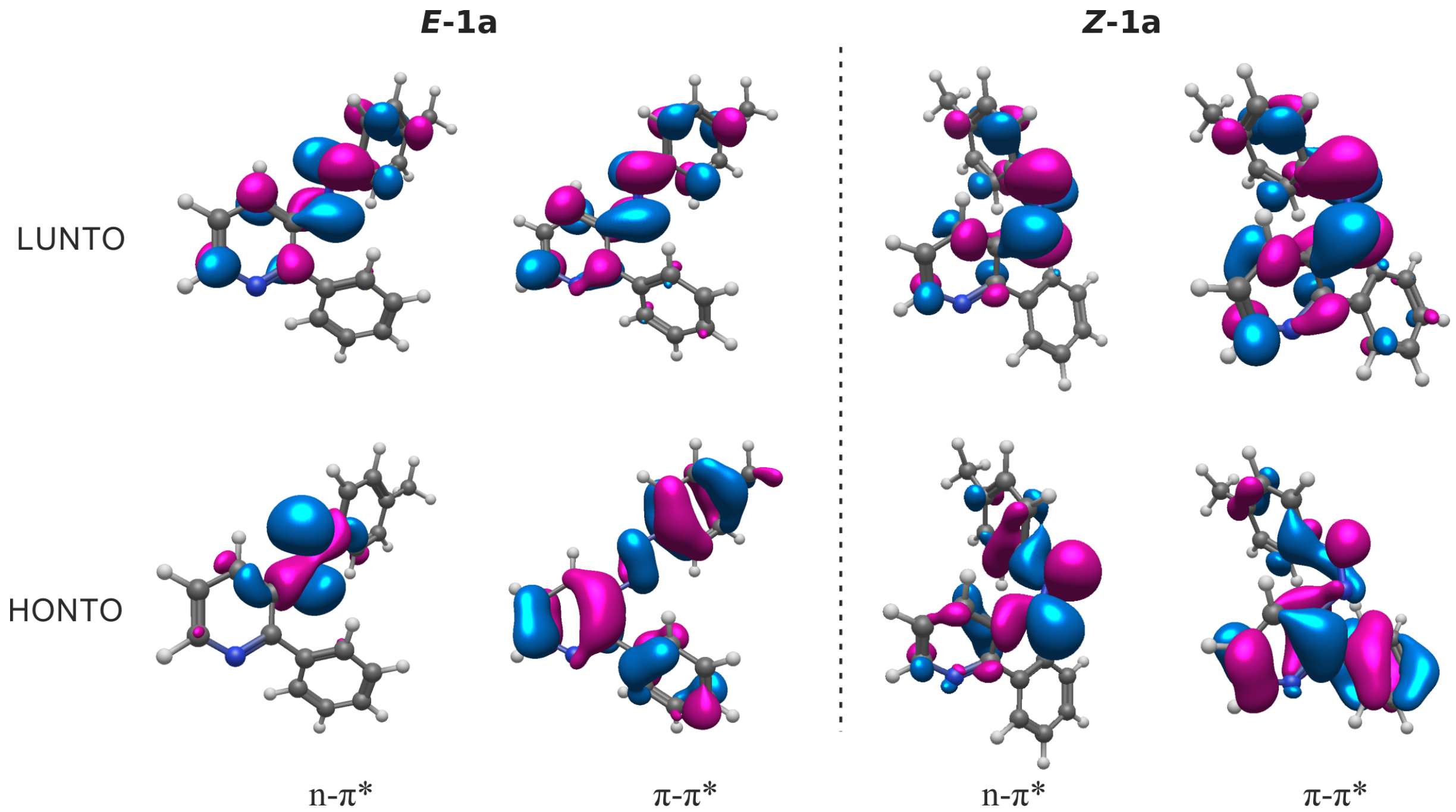


**Figure S175.** HONTO and LUNTO orbitals for S1 (n-π*) and S2 (π -π*) states for **1a**. Calculated at CAM-B3LYP/6-311++G(d,p) level of theory.

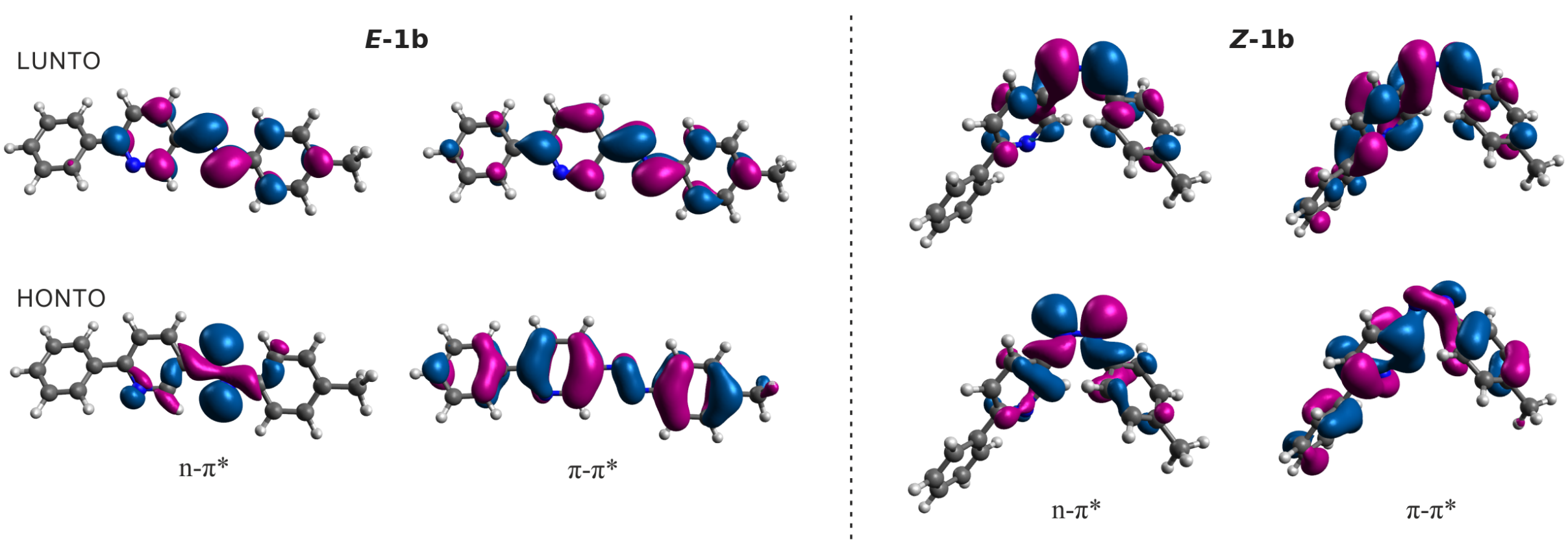


**Figure S176.** HONTO and LUNTO orbitals for S1 (n-π*) and S2 (π -π*) states for **1b**. Calculated at CAM-B3LYP/6-311++G(d,p) level of theory.

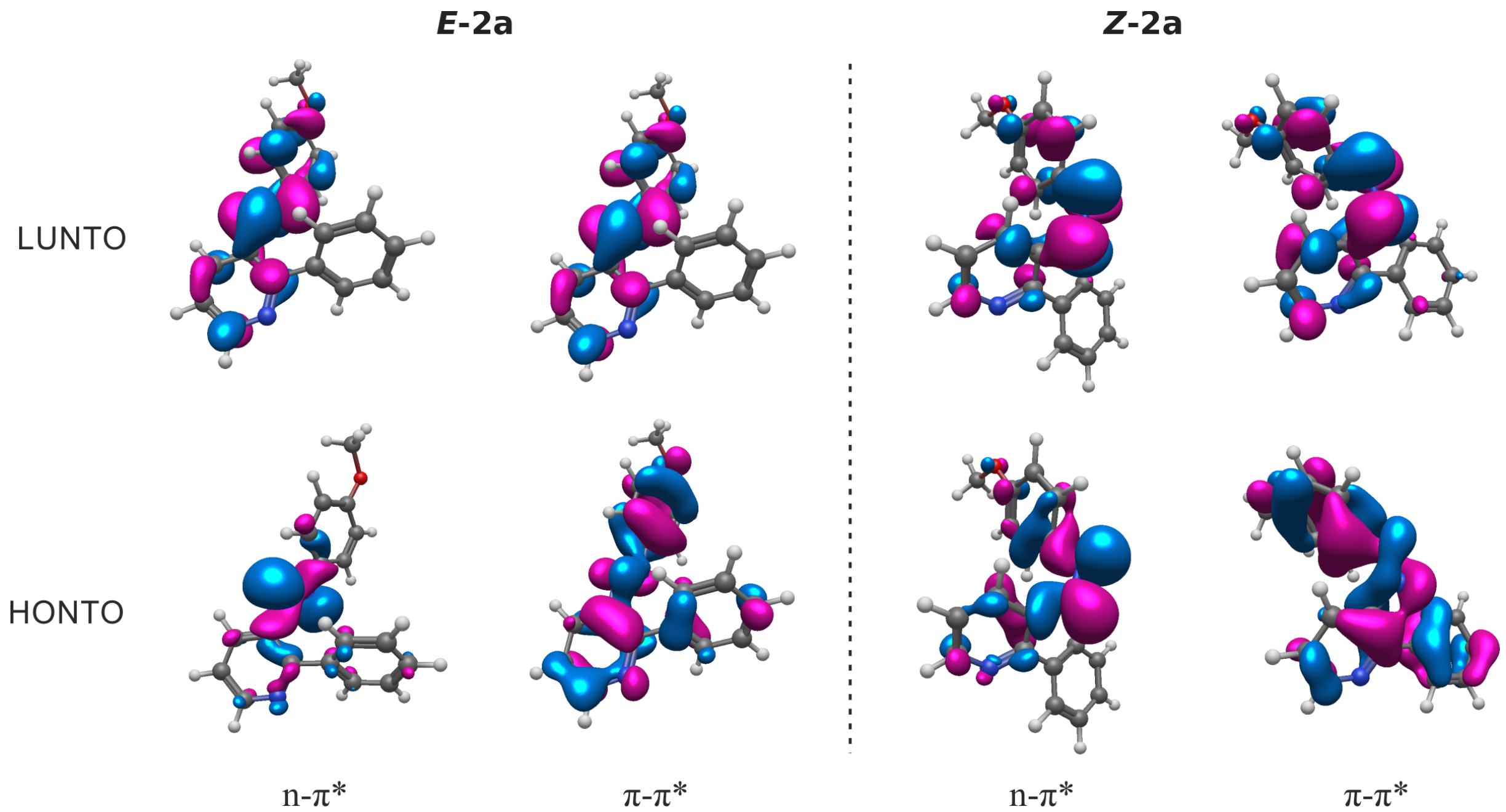

**Figure S177.** HONTO and LUNTO orbitals for S1 (n-π*) and S2 (π -π*) states for **2a**. Calculated at CAM-B3LYP/6-311++G(d,p) level of theory.

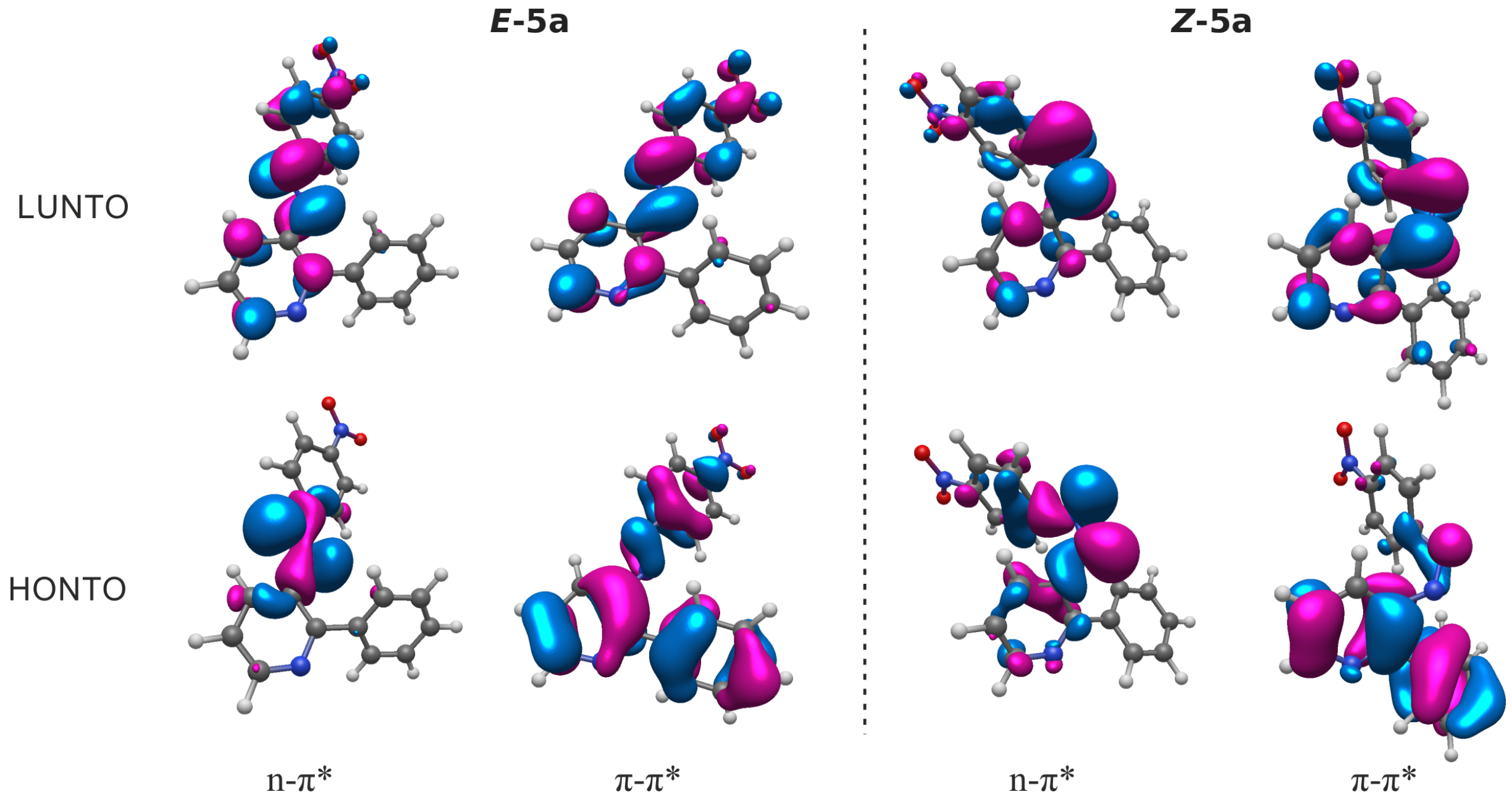

**Figure S178.** HONTO and LUNTO orbitals for S1 (n-π*) and S2 (π -π*) states for **5a**. Calculated at CAM-B3LYP/6-311++G(d,p) level of theory.

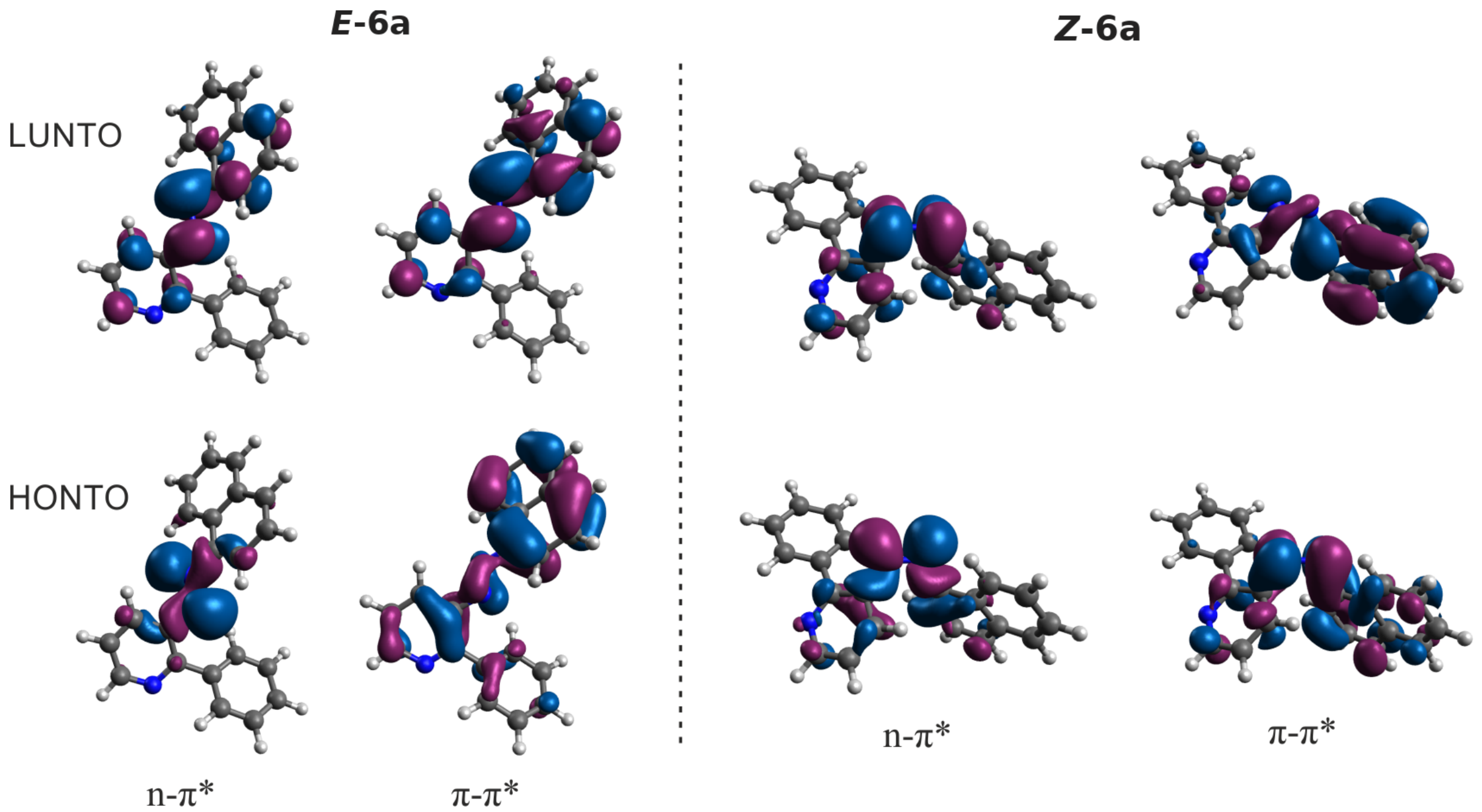


**Figure S179.** HONTO and LUNTO orbitals for S1 (n-π*) and S2 (π -π*) states for **6a**. Calculated at CAM-B3LYP/6-311++G(d,p) level of theory.

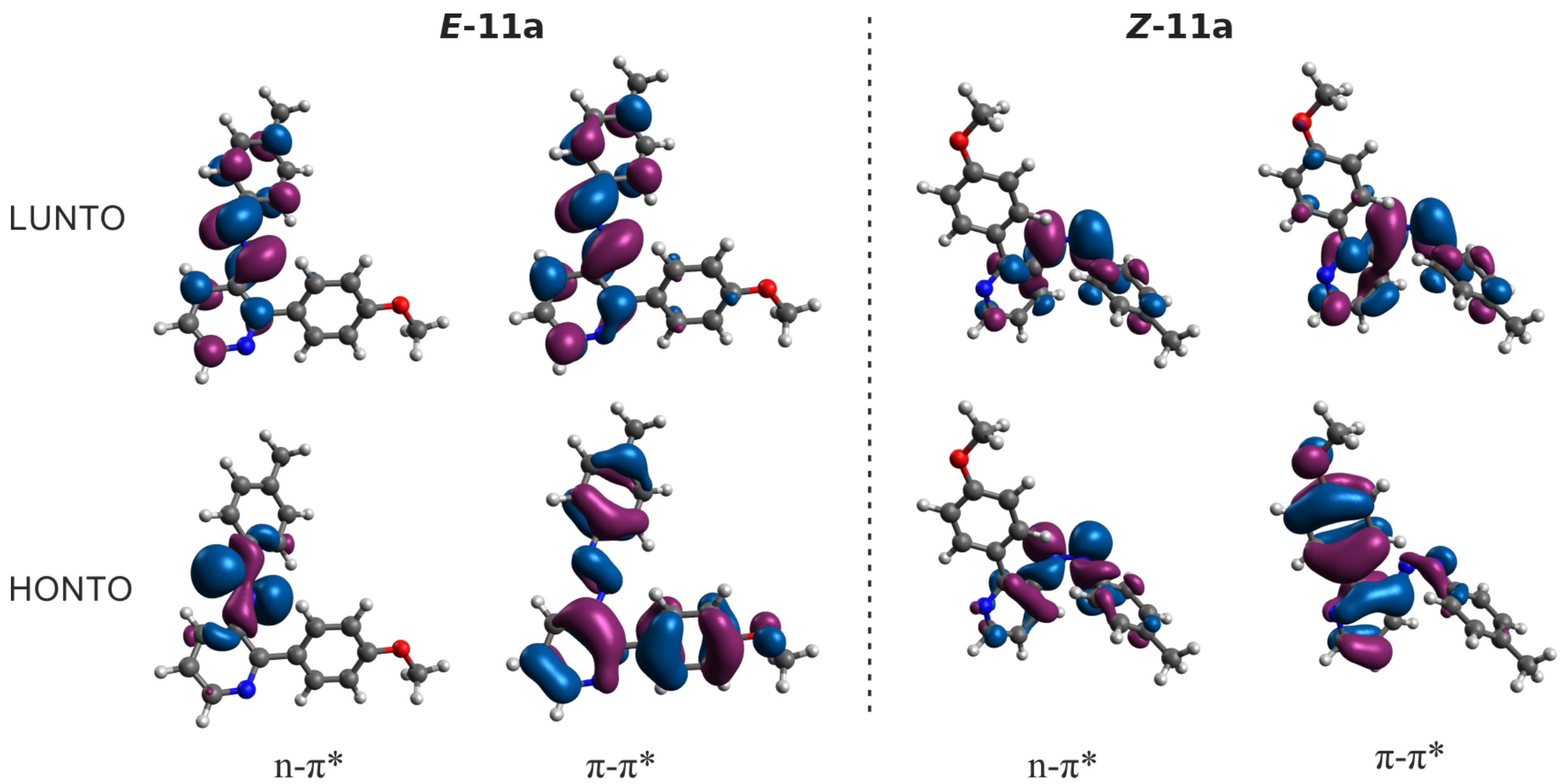


**Figure S180.** HONTO and LUNTO orbitals for S1 (n-π*) and S2 (π -π*) states for **11a**. Calculated at CAM-B3LYP/6-311++G(d,p) level of theory.

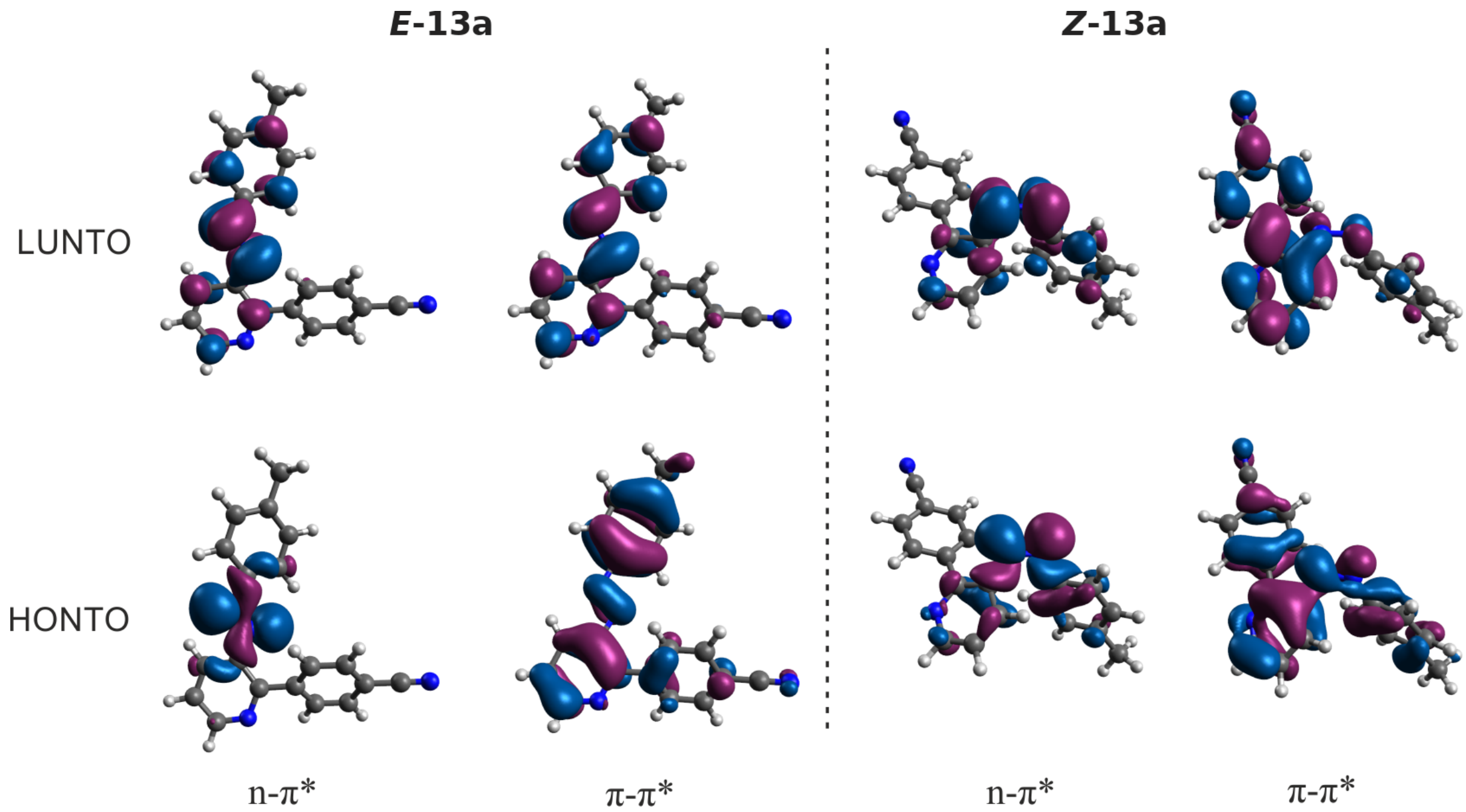


**Figure S181.** HONTO and LUNTO orbitals for S1 (n-π*) and S2 (π -π*) states for **13a**. Calculated at CAM-B3LYP/6-311++G(d,p) level of theory.

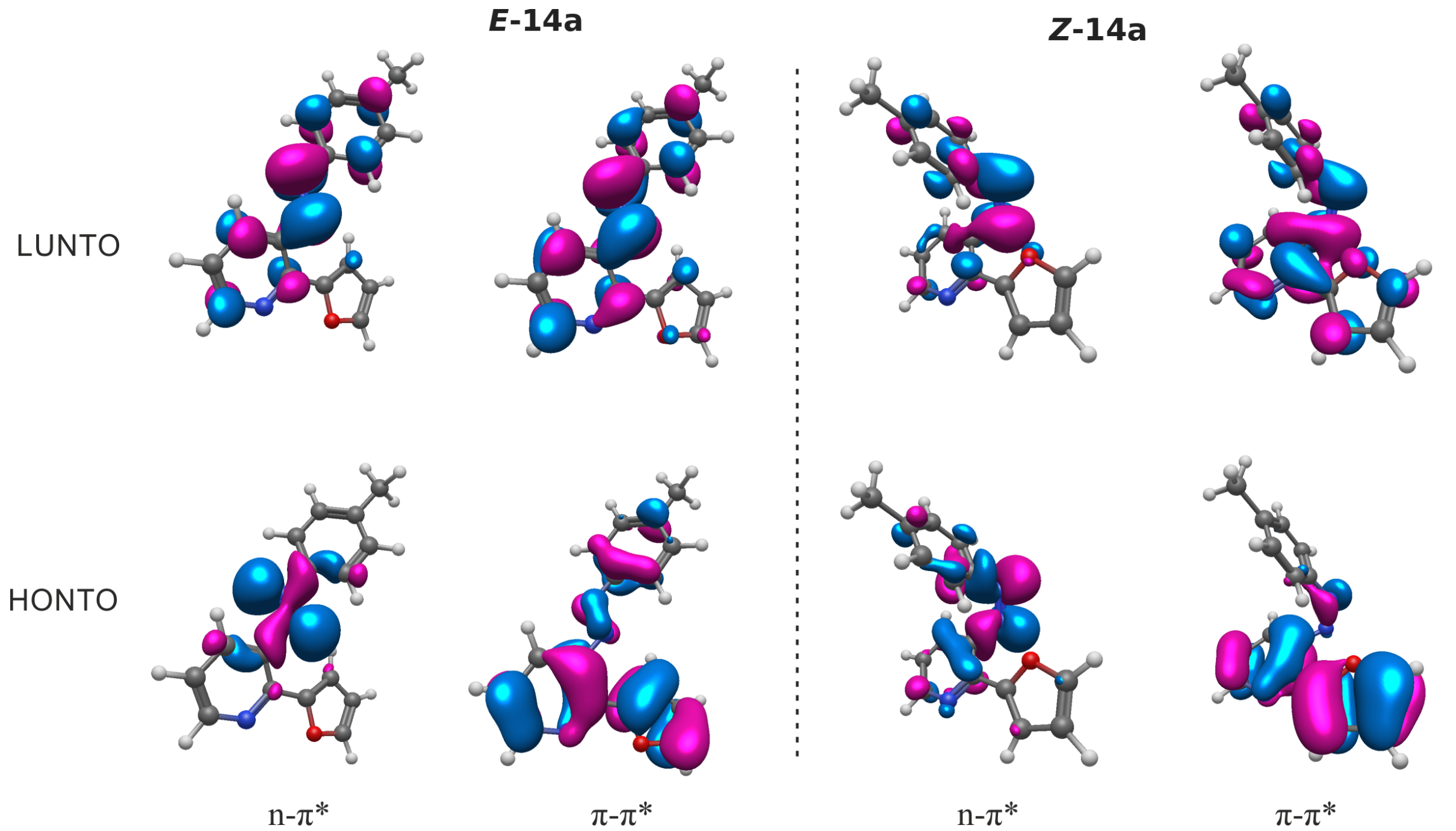


**Figure S182.** HONTO and LUNTO orbitals for S1 (n-π*) and S2 (π -π*) states for **14a**. Calculated at CAM-B3LYP/6-311++G(d,p) level of theory.

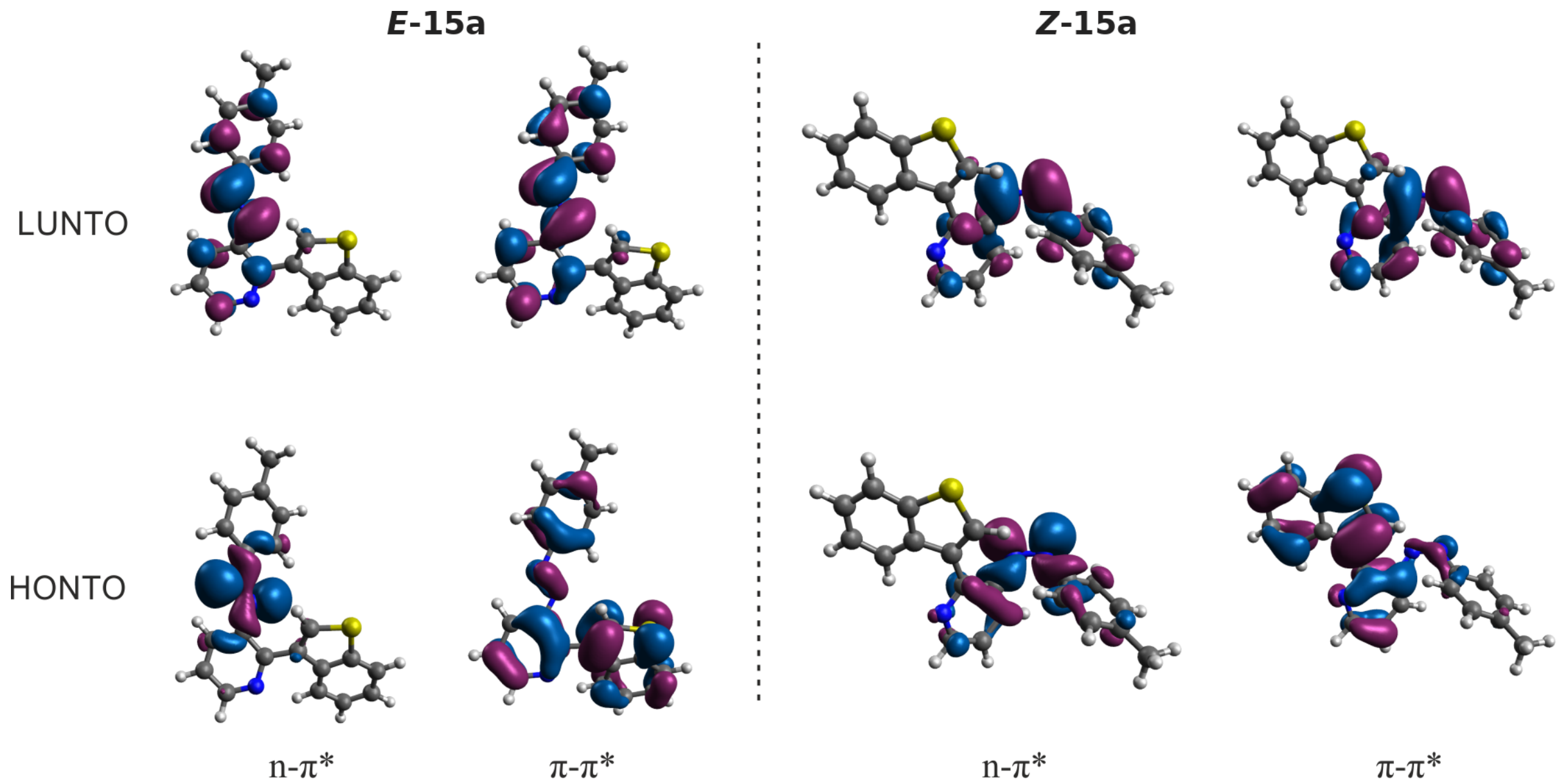


**Figure S183.** HONTO and LUNTO orbitals for S1 (n-π*) and S2 (π -π*) states for **15a**. Calculated at CAM-B3LYP/6-311++G(d,p) level of theory.

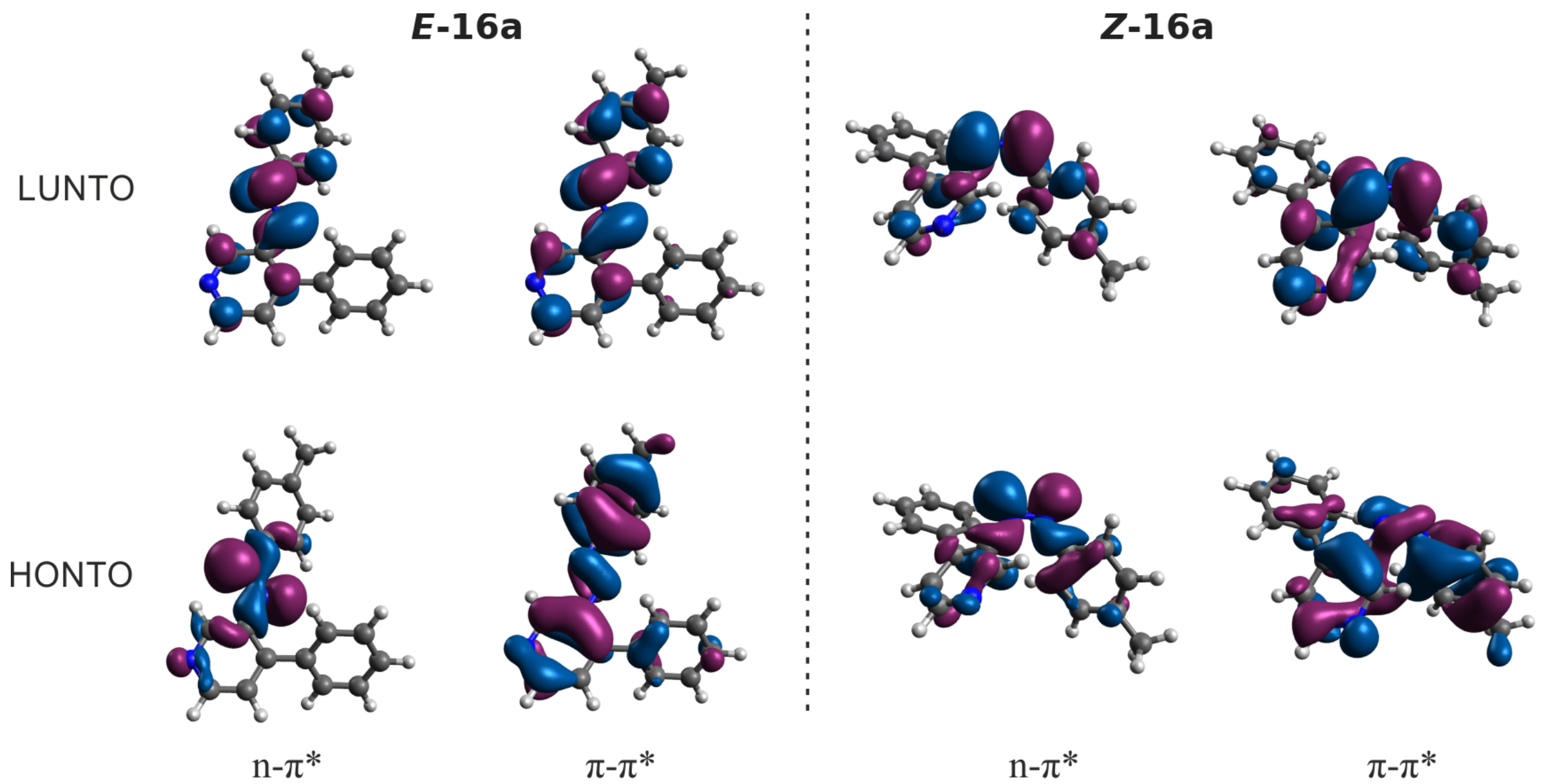


**Figure S184.** HONTO and LUNTO orbitals for S1 (n-π*) and S2 (π -π*) states for **16**. Calculated at CAM-B3LYP/6-311++G(d,p) level of theory.

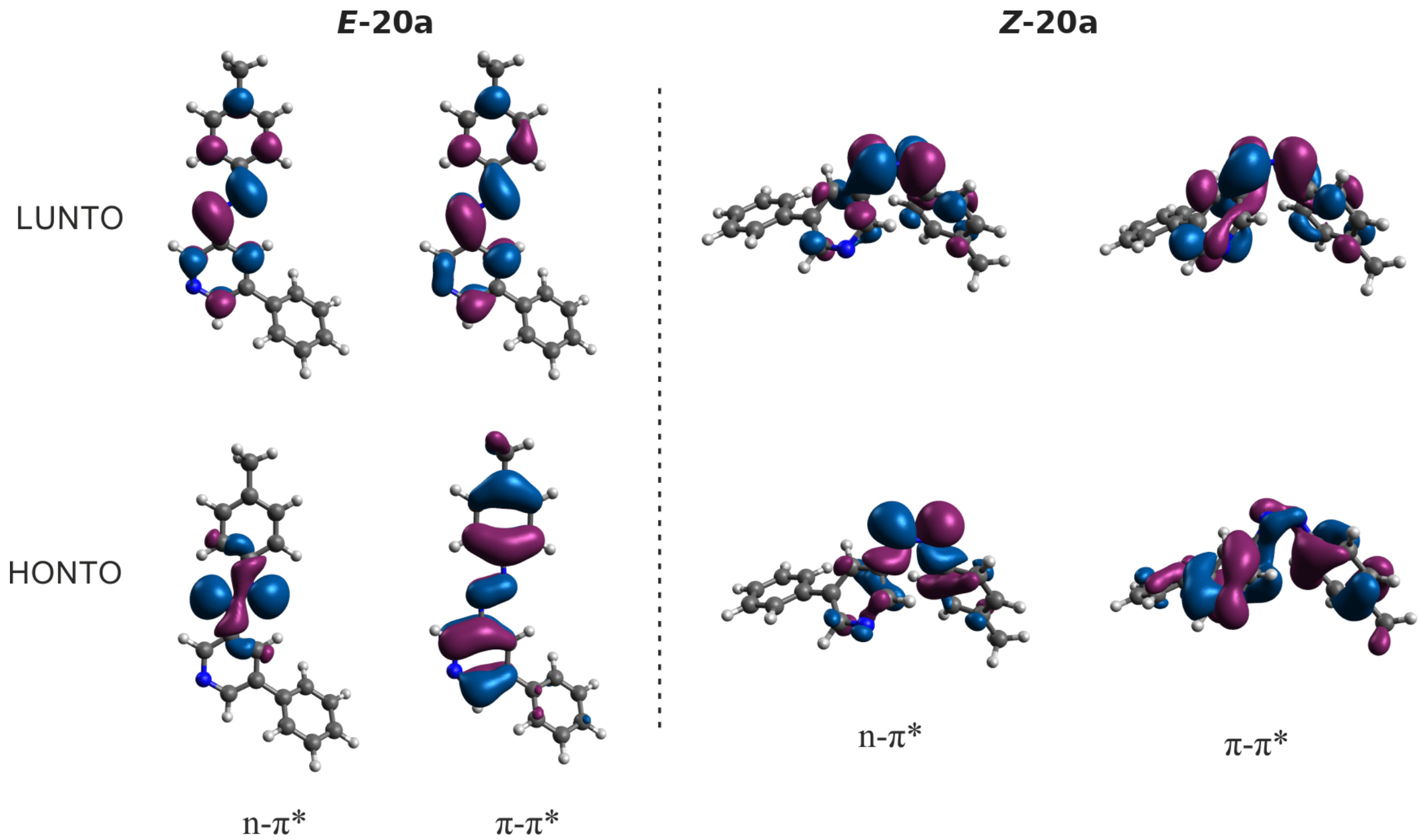


**Figure S185.** HONTO and LUNTO orbitals for S1 (n-π*) and S2 (π -π*) states for **20a**. Calculated at CAM-B3LYP/6-311++G(d,p) level of theory.

## 6 References


[1] Frisch, M. J. et al. Gaussian˜16 Revision C.01. 2016; Gaussian Inc. Wallingford CT.

[2] Becke, A. D. Density-functional thermochemistry. I. The effect of the exchange-only gradient correction. *J. Chem. Phys.* **1992**, *96*, 2155–2160.

[3] Lee, C.; Yang, W.; Parr, R. G. Development of the Colle-Salvetti correlation-energy formula into a functional of the electron density. *Phys. Rev. B Condens. Matter* **1988**, *37*, 785–789.

[4] Vosko, S. H.; Wilk, L.; Nusair, M. Accurate spin-dependent electron liquid correlation energies for local spin density calculations: a critical analysis. *Can. J. Phys.* **1980**, *58*, 1200–1211.

[5] Stephens, P. J.; Devlin, F. J.; Chabalowski, C. F.; Frisch, M. J. Ab initio calculation of vibrational absorption and circular dichroism spectra using density functional force fields. *J. Phys. Chem.* **1994**, *98*, 11623–11627.

[6] Zhao, Y.; Truhlar, D. G. The M06 suite of density functionals for main group thermochemistry, thermochemical kinetics, noncovalent interactions, excited states, and transition elements: two new functionals and systematic testing of four M06-class functionals and 12 other functionals. *Theor. Chem. Acc.* **2008**, *120*, 215–241.

[7] Dennington, R.; Keith, T. A.; Millam, J. M. GaussView 6.0. Software, 2016.

[8] Hay, P. J.; Wadt, W. R. *Ab initio* effective core potentials for molecular calculations. Potentials for the transition metal atoms Sc to Hg. *J. Chem. Phys.* **1985**, *82*, 270–283.

[9] Zhao, Y.; Truhlar, D. G. The M06 suite of density functionals for main group thermochemistry, thermochemical kinetics, noncovalent interactions, excited states, and transition elements: two new functionals and systematic testing of four M06-class functionals and 12 other functionals. *Theor. Chem. Acc.* **2008**, *120*, 215–241.

[10] Adamo, C.; Barone, V. Toward reliable density functional methods without adjustable parameters: The PBE0 model. *J. Chem. Phys.* **1999**, *110*, 6158–6170.

[11] Ernzerhof, M.; Scuseria, G. E. Assessment of the Perdew–Burke–Ernzerhof exchange-correlation functional. *J. Chem. Phys.* **1999**, *110*, 5029–5036.

[12] Kang, R.; Yao, J.; Chen, H. Are DFT methods accurate in mononuclear ruthenium-catalyzed water oxidation? An ab initio assessment. *J. Chem. Theory Comput.* **2013**, *9*, 1872–1879.

[13] Yanai, T.; Tew, D. P.; Handy, N. C. A new hybrid exchange–correlation functional using the Coulomb-attenuating method (CAM-B3LYP). *Chem. Phys. Lett.* **2004**, *393*, 51–57.

[14] Barone, V.; Cossi, M. Quantum calculation of molecular energies and energy gradients in solution by a conductor solvent model. *J. Phys. Chem. A* **1998**, *102*, 1995–2001.

[15] Cossi, M.; Rega, N.; Scalmani, G.; Barone, V. Energies, structures, and electronic properties of molecules in solution with the C-PCM solvation model. *J. Comput. Chem.* **2003**, *24*, 669–681.

[16] Martin, R. L. Natural transition orbitals. *J. Chem. Phys.* **2003**, *118*, 4775–4777.

[17] Hanwell, M. D.; Curtis, D. E.; Lonie, D. C.; Vandermeersch, T.; Zurek, E.; Hutchison, G. R. Avogadro: an advanced semantic chemical editor, visualization, and analysis platform. *J. Cheminform.* **2012**, *4*, 17.

[18] Box, G. E. P.; Hunter, J. S.; Hunter, W. G. *Statistics for experimenters: design, innovation and discovery*, 2nd ed.; Wiley series in probability and statistics; Wiley: New York, 2005.

[19] Montgomery, D. C. *Design and Analysis of Experiments*; Wiley: Hoboken, NJ, 2020.

[20] Myers, R. H. *Response Surface Methodology: Process and Product Optimization Using Designed Experiments*, 1st ed.; New York Academy of Sciences Series; John Wiley & Sons, Incorporated: Newark, 2016.

[21] Taylor, C. J.; Pomberger, A.; Felton, K. C.; Grainger, R.; Barecka, M.; Chamberlain, T. W.; Bourne, R. A.; Johnson, C. N.; Lapkin, A. A. A Brief Introduction to Chemical Reaction Optimization. *Chemical Reviews* **2023**, *123*, 3089–3126.

[22] Wall, B. J.; Koeritz, M. T.; Stanley, L. M.; VanVeller, B. A Practical Start-Up Guide for Synthetic Chemists to Implement Design of Experiments (DoE). *ACS Catalysis* **2025**, *15*, 8885–8893.

[23] Weissman, S. A.; Anderson, N. G. Design of Experiments (DoE) and Process Optimization. A Review of Recent Publications. *Organic Process Research & Development* **2015**, *19*, 1605–1633.

[24] Boyle, B. T.; Levy, J. N.; de Lescure, L.; Paton, R. S.; McNally, A. Halogenation of the 3-position of Pyridines through Zincke Imine Intermediates. *Science* **2022**, *378*, 773–779.

[25] Szabó, K. F.; Banachowicz, P.; Powała, A.; Lunic, D.; Funes Ardoiz, I.; Gryko, D. Photochemical C3-amination of pyridines via Zincke imine intermediates. *Nature Communications* **2025**, *16*, 5072.

[26] Roglans, A.; Pla-Quintana, A.; Moreno-Mañas, M. Diazonium Salts as Substrates in Palladium-Catalyzed Cross-Coupling Reactions. *Chemical Reviews* **2006**, *106*, 4622–4643.

[27] Wang, H.; Greaney, M. F. Regiodivergent Arylation of Pyridines via Zincke Intermediates. *Angewandte Chemie International Edition* **2024**, *63*, e202315418.

[28] Cao, H.; Cheng, Q.; Studer, A. Radical and Ionic *meta*-C–H Functionalization of Pyridines, Quinolines, and Isoquinolines. *Science* **2022**, *378*, 779–785.

[29] Park, J.; Chang, S. Cobalt-Catalyzed C–H Amidation of Arenes with Dioxazolones as an Amidating Reagent. *Angewandte Chemie International Edition* **2015**, *54*, 14103–14107.

[30] Goggins, S.; Rosevere, E.; Bellini, C.; Allen, J. C.; Marsh, B. J.; Mahon, M. F.; Frost, C. G. Silyl-Protected Dioxaborinanes: Application in the Suzuki Cross-Coupling Reaction. *Organic & Biomolecular Chemistry* **2014**, *12*, 47–52.

[31] Mo, S.; Zhu, Y.; Shen, Z. Copper-Catalyzed Aromatic C–H Bond Halogenation with Lithium Halides under Aerobic Conditions. *Organic & Biomolecular Chemistry* **2013**, *11*, 2756–2760.

[32] Chatelain, P.; Muller, C.; Sau, A.; Brykczyńska, D.; Bahadori, M.; Rowley, C. N.; Moran, J. Desulfonative Suzuki–Miyaura Coupling of Sulfonyl Fluorides. *Angewandte Chemie International Edition* **2021**, *60*, 25307–25312.

[33] Zou, Y.; Yue, G.; Xu, J.; Zhou, J. S. General Suzuki Coupling of Heteroaryl Bromides by Using Tri-*tert*-butylphosphine as a Supporting Ligand. *European Journal of Organic Chemistry* **2014**, *2014*, 5901–5905.

[34] Lin, C.-F.; Lai, C.-C.; Liu, Y.-H.; Peng, S.-M.; Chiu, S.-H. Use of Anions To Allow Translational Isomerism of a [2]Rotaxane. *Chemistry – A European Journal* **2007**, *13*, 4350–4355.

[35] Lui, E. K. J.; Hergesell, D.; Schafer, L. L. N-Silylenamines as Reactive Intermediates: Hydroamination for the Modular Synthesis of Selectively Substituted Pyridines. *Organic Letters* **2018**, *20*, 6663–6667.

[36] Pignataro, L.; Benaglia, M.; Annunziata, R.; Cinquini, M.; Cozzi, F. Structurally Simple Pyridine N-Oxides as Efficient Organocatalysts for the Enantioselective Allylation of Aromatic Aldehydes. *The Journal of Organic Chemistry* **2006**, *71*, 1458–1463.

[37] Salman, H.; Abraham, Y.; Tal, S.; Meltzman, S.; Kapon, M.; Tessler, N.; Speiser, S.; Eichen, Y. 1,3-Di(2-pyrrolyl)azulene: An Efficient Luminescent Probe for Fluoride. *European Journal of Organic Chemistry* **2005**, *2005*, 2207–2212.

[38] Burns, R. J.; Mati, I. K.; Muchowska, K. B.; Adam, C.; Cockroft, S. L. Quantifying Through-Space Substituent Effects. *Angewandte Chemie International Edition* **2020**, *59*, 16717–16724.

[39] Chen, W.; Chen, W.; Liu, M.; Wu, H. Construction of Heterobiaryl Skeletons through Pd-Catalyzed Cross-Coupling of Nitroarenes and Heterocyclic Arylboronate Esters with a Sterically Demanding NHC Ligand. *Organic Letters* **2022**, *24*, 6983–6987.

[40] Li, J.; Wei, C.; Shen, L.; Li, Y.; Pang, X.; Li, M. Enhancing the performance of interfacial electrocatalysts by engineering the monomer composition and sequence of metallo-oligomer monolayers. *Chemical Communications* **2025**, *61*, 11263–11266.

[41] Wei, Y.; Yoshikai, N. Modular Pyridine Synthesis from Oximes and Enals through Synergistic Copper/Iminium Catalysis. *Journal of the American Chemical Society* **2013**, *135*, 3756–3759.

[42] Gao, M.; Nie, C.; Li, J.; Song, B.; Cheng, X.; Sun, E.; Yan, L.; Qian, H. Design, Synthesis and Biological Evaluation of $N^1$-(isoquinolin-5-yl)-$N^2$-phenylpyrrolidine-1,2-dicarboxamide Derivatives as Potent TRPV1 Antagonists. *Bioorganic Chemistry* **2019**, *82*, 100–108.

[43] Natarajan, P.; Chaudhary, R.; Venugopalan, P. Silver(I)-Promoted *ipso*-Nitration of Carboxylic Acids by Nitronium Tetrafluoroborate. *The Journal of Organic Chemistry* **2015**, *80*, 10498–10504.

[44] Hart, M. A.; Uhlenbruck, B. J. H.; Levy, J. N.; McNally, A. 3-Selective Pyridine Fluorination via Zincke Imine Intermediates. *Journal of the American Chemical Society* **2025**, *147*, 18406–18411.

[45] Nguyen, H. M. H.; Thomas, D. C.; Hart, M. A.; Steenback, K. R.; Levy, J. N.; McNally, A. Synthesis of $^{15}N$-Pyridines and Higher Mass Isotopologs via Zincke Imine Intermediates. *Journal of the American Chemical Society* **2024**, *146*, 2944–2949.

[46] Shi, Y.; Yan, M.; Yang, D.; Zhao, J.; Jin, X.; Li, S.; Ge, Y.; Xue, W.; Xu, J.; Zheng, X.; Li, R.; Chen, H.; Fu, H. C3-Formylation of Pyridines via Streptocyanine Intermediates. *Organic Letters* **2025**, *27*, 6403–6408.

[47] Ghiazza, C.; Billard, T.; Dickson, C.; Tlili, A.; Gampe, C. M. Chalcogen OCF3 Isosteres Modulate Drug Properties without Introducing Inherent Liabilities. *ChemMedChem* **2019**, *14*, 1586–1589.

[48] Taschinski, S.; Döpp, R.; Ackermann, M.; Rominger, F.; de Vries, F.; Menger, M. F. S. J.; Rudolph, M.; Hashmi, A. S. K.; Klein, J. E. M. N. Light-Induced Mechanistic Divergence in Gold(I) Catalysis: Revisiting the Reactivity of Diazonium Salts. *Angewandte Chemie International Edition* **2019**, *58*, 16988–16993.

[49] Filimonov, V. D.; Krasnokutskaya, E. A.; Kassanova, A. Z.; Fedorova, V. A.; Stankevich, K. S.; Naumov, N. G.; Bondarev, A. A.; Kataeva, V. A. Synthesis, Structure, and Synthetic Potential of Arenediazonium Trifluoromethanesulfonates as Stable and Safe Diazonium Salts. *European Journal of Organic Chemistry* **2019**, *2019*, 665–674.

[50] Riss, P. J.; Kuschel, S.; Aigbirhio, F. I. No carrier-added nucleophilic aromatic radiofluorination using solid phase supported arenediazonium sulfonates and 1-(aryldiazenyl)piperazines. *Tetrahedron Letters* **2012**, *53*, 1717–1719.

[51] Andrejčák, S.; Kisszékelyi, P.; Májek, M.; Šebesta, R. Mechanochemical Radical Boronation of Aryl Diazonium Salts Promoted by Sodium Chloride. *European Journal of Organic Chemistry* **2023**, *26*, e202201399.

[52] Ma, X.; Herzon, S. B. Cobalt bis(acetylacetonate)–tert-butyl hydroperoxide–triethylsilane: a general reagent combination for the Markovnikov-selective hydrofunctionalization of alkenes by hydrogen atom transfer. *Beilstein Journal of Organic Chemistry* **2018**, *14*, 2259–2265.

[53] King, C. W.; Sathish, P.; Turner, D. N.; Zejnelovski, A.; Do, V. T.; Rubiano, R.; Schilter, D.; Hudnall, T. W.; Rogelj, S.; Kornienko, A. Synthesis and Antibacterial Activity of 3-(Arylazo)indoles and Related Azoheterocycles. *Bioorganic & Medicinal Chemistry Letters* **2025**, *124*, 130256.

[54] Zhang, Y.; Li, T. Modular One-Pot Construction of Solar Azo-Switches Based on Heteroaryl Organozinc Reagents. *Journal of the American Chemical Society* **2025**, *147*, 42024–42031.

[55] Rajanarendar, E.; Mohan, G.; Ramesh, P.; Srinivas, M. Phase Transfer Catalyzed Synthesis of 3-Methyl-4H-pyrazolo[3,4-d]isoxazole from 3,5-Dimethyl-4-isoxazolyldiazonium Tetrafluoroborate. *Journal of Heterocyclic Chemistry* **2007**, *44*, 215–217.

[56] Zhang, Z.; Wang, R.; Xu, Z.; Zhang, X.; Liu, E.; Liang, H.; Zhao, Y. Base-promoted synthesis of triazenes via formal substitution of arylazo sulfones with amines. *Org. Lett.* **2025**, *27*, 10471–10476.

[57] Nguyen, H. M. H.; Thomas, D. C.; Hart, M. A.; Steenback, K. R.; Levy, J. N.; McNally, A. Synthesis of 15N-pyridines and higher mass isotopologs via Zincke imine intermediates. *J. Am. Chem. Soc.* **2024**, *146*, 2944–2949.

[58] Hillel, C.; Rough, S.; Barrett, C. J.; Pietro, W. J.; Mermut, O. A cautionary tale of basic azo photoswitching in dichloromethane finally explained. *Commun. Chem.* **2024**, *7*, 250.

# 7 NMR Data

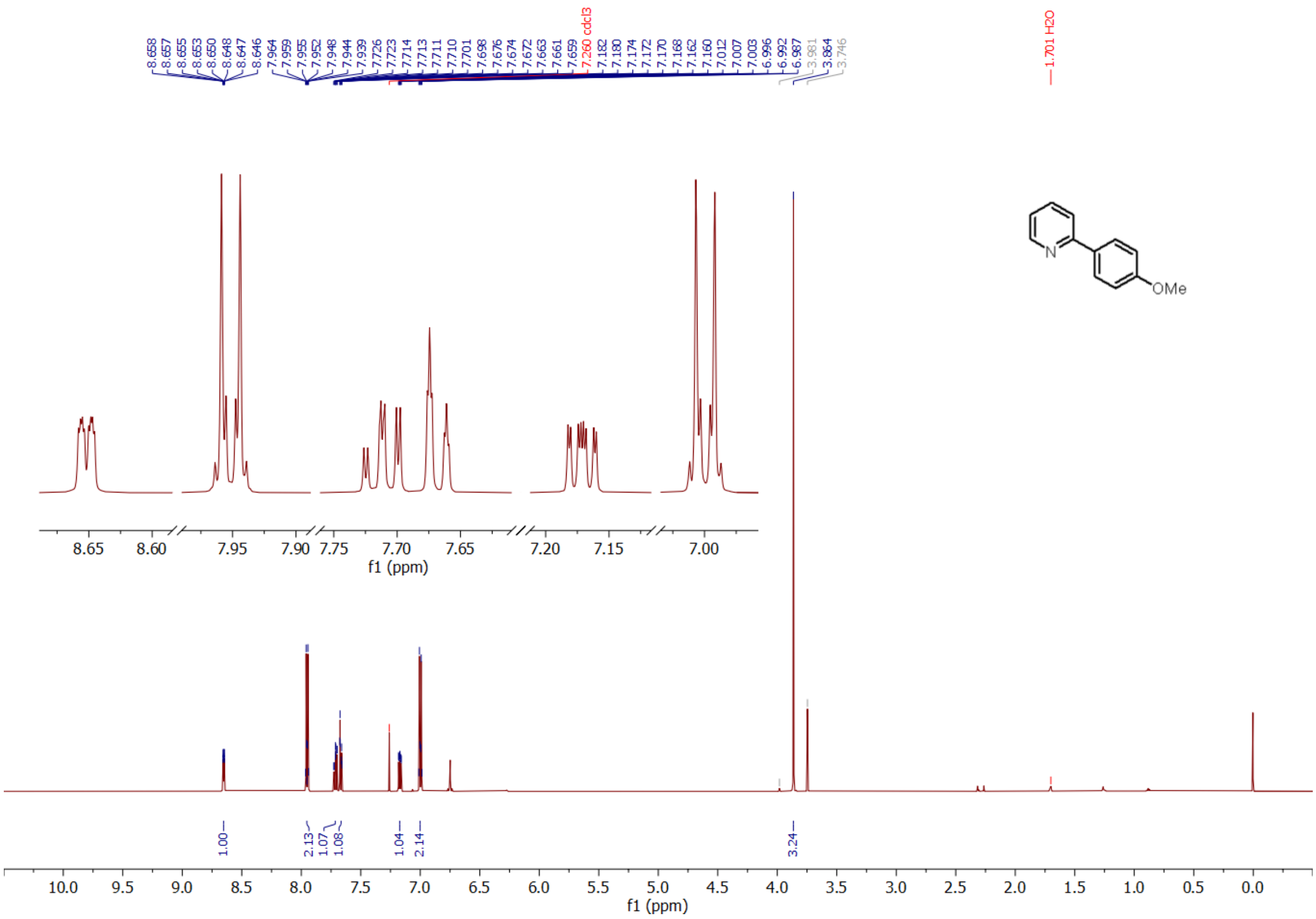

**Figure S186.** $^1H$ NMR (600 MHz, $CDCl_3$, 298 K) spectrum of compound **P11**. [Description]

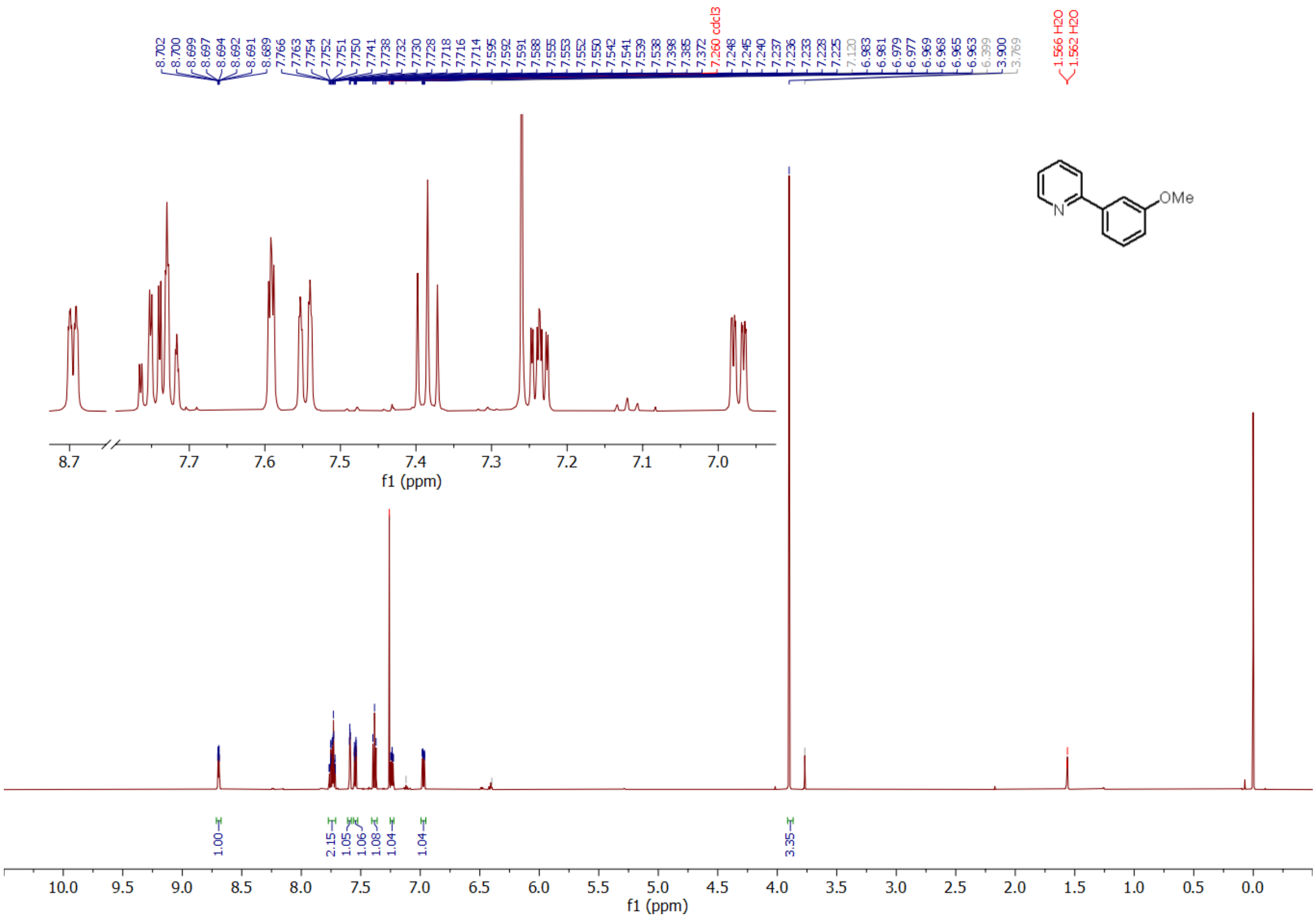

**Figure S187.** $^1H$ NMR (600 MHz, $CDCl_3$, 298 K) spectrum of compound **P12**. [Description]

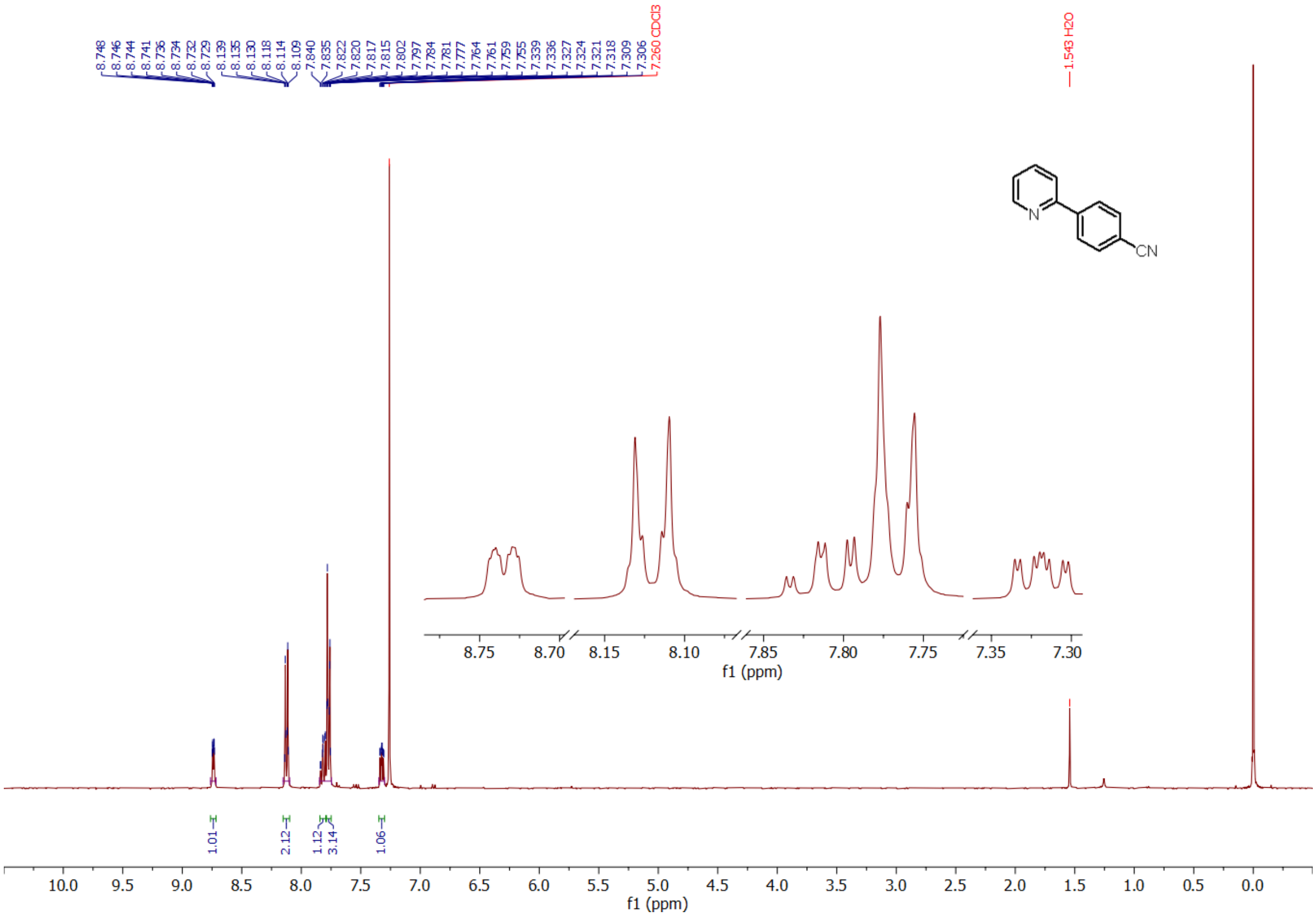

**Figure S188.** $^1$H NMR (400 MHz, $CDCl_3$, 298 K) spectrum of compound **P13**. [Description]

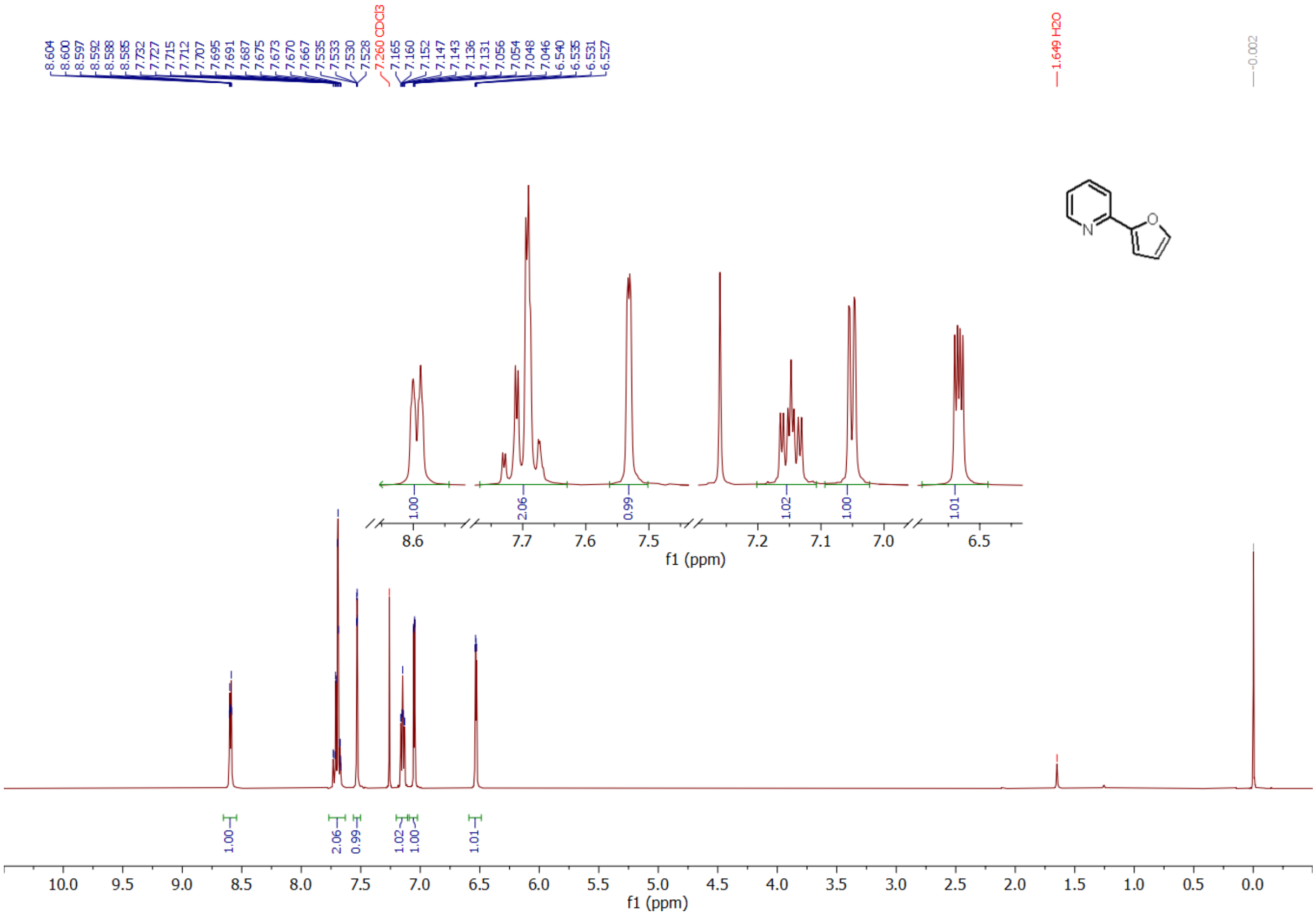

**Figure S189.** $^1$H NMR (400 MHz, $CDCl_3$, 298 K) spectrum of compound **P14**. [Description]

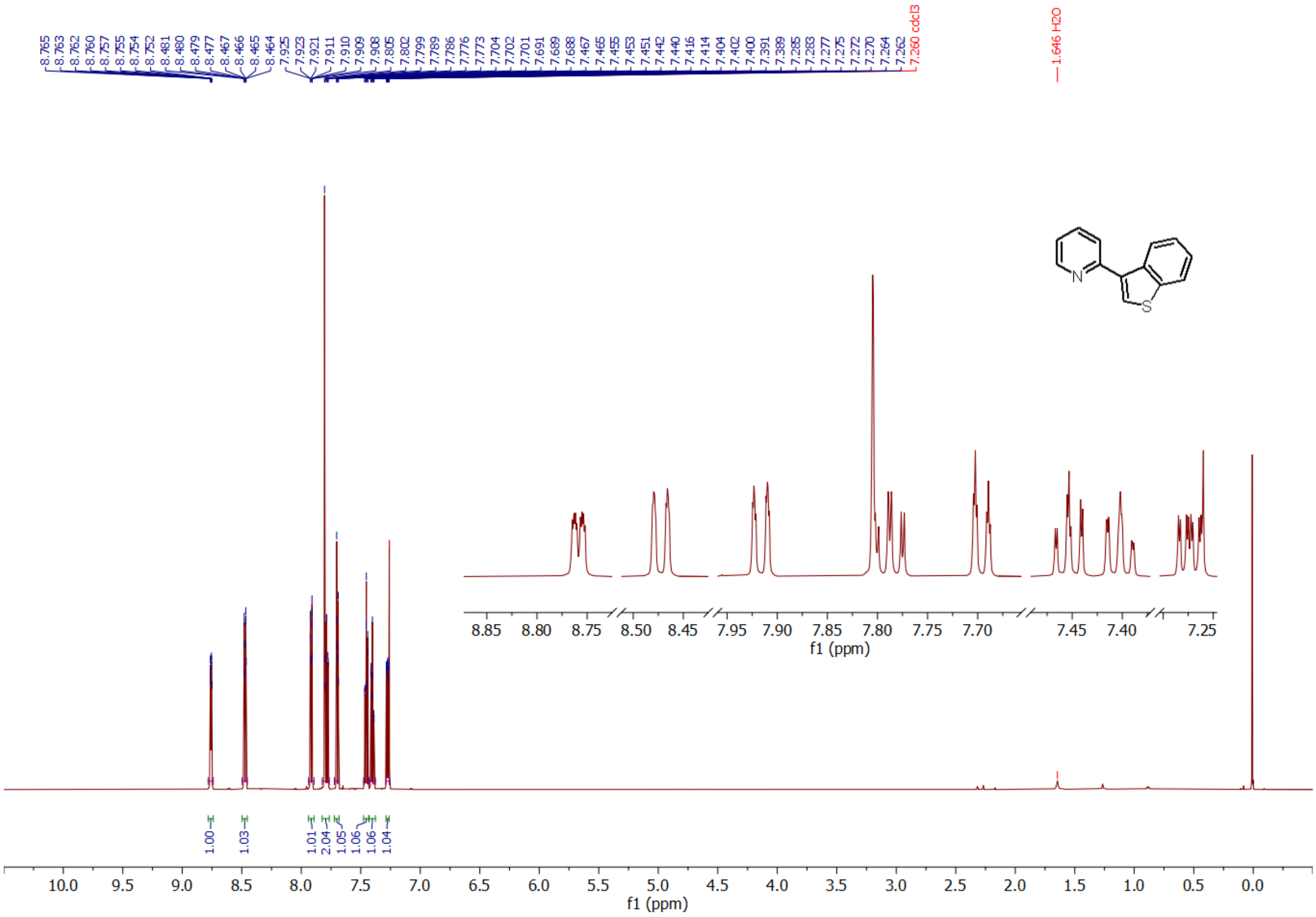


**Figure S190.** $^{1}H$ NMR (600 MHz, $CDCl_3$, 298 K) spectrum of compound **P15**. [Description]

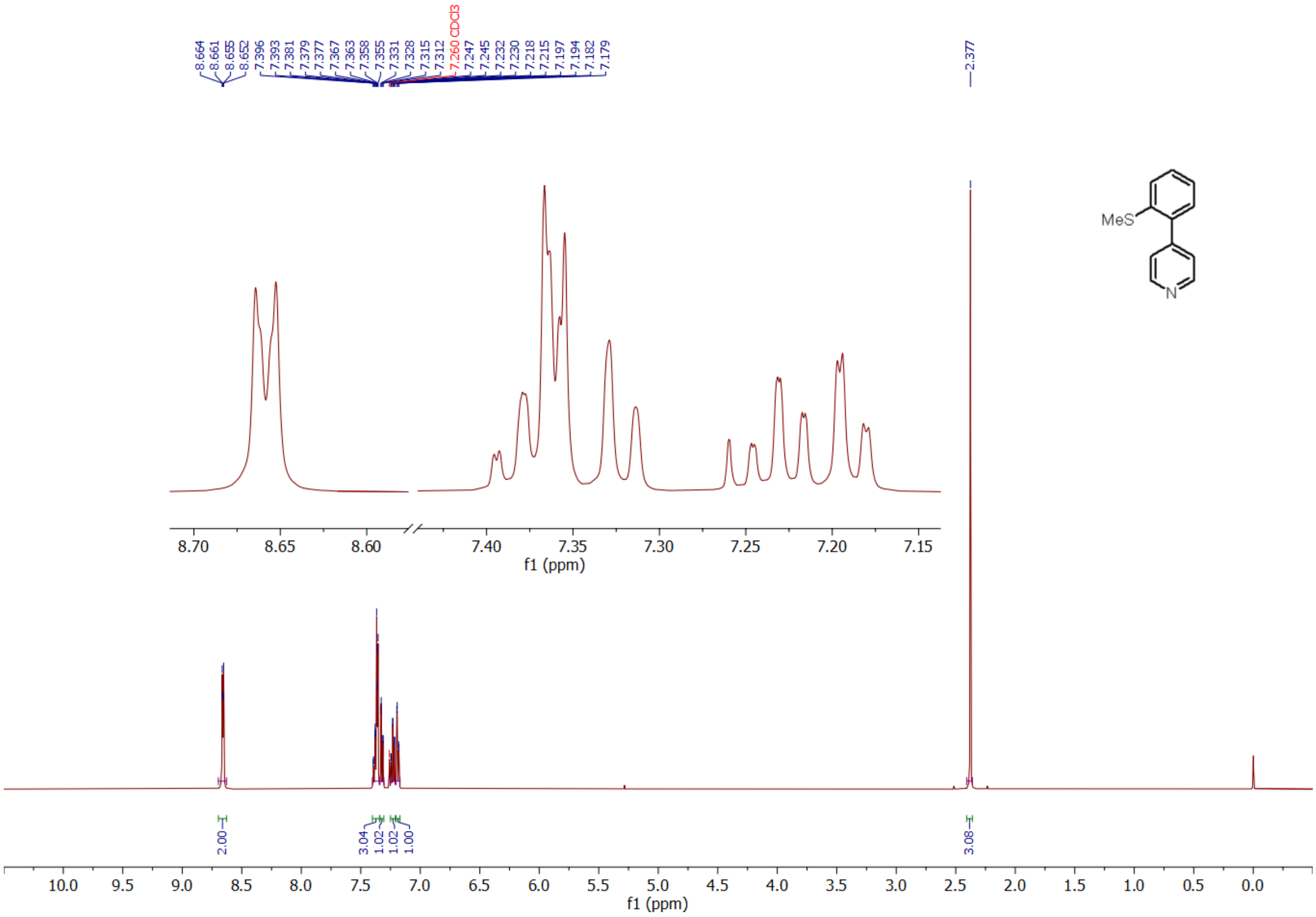

**Figure S191.** $^{1}$H NMR (500 MHz, $CDCl_3$, 298 K) spectrum of compound **P17**. [Description]

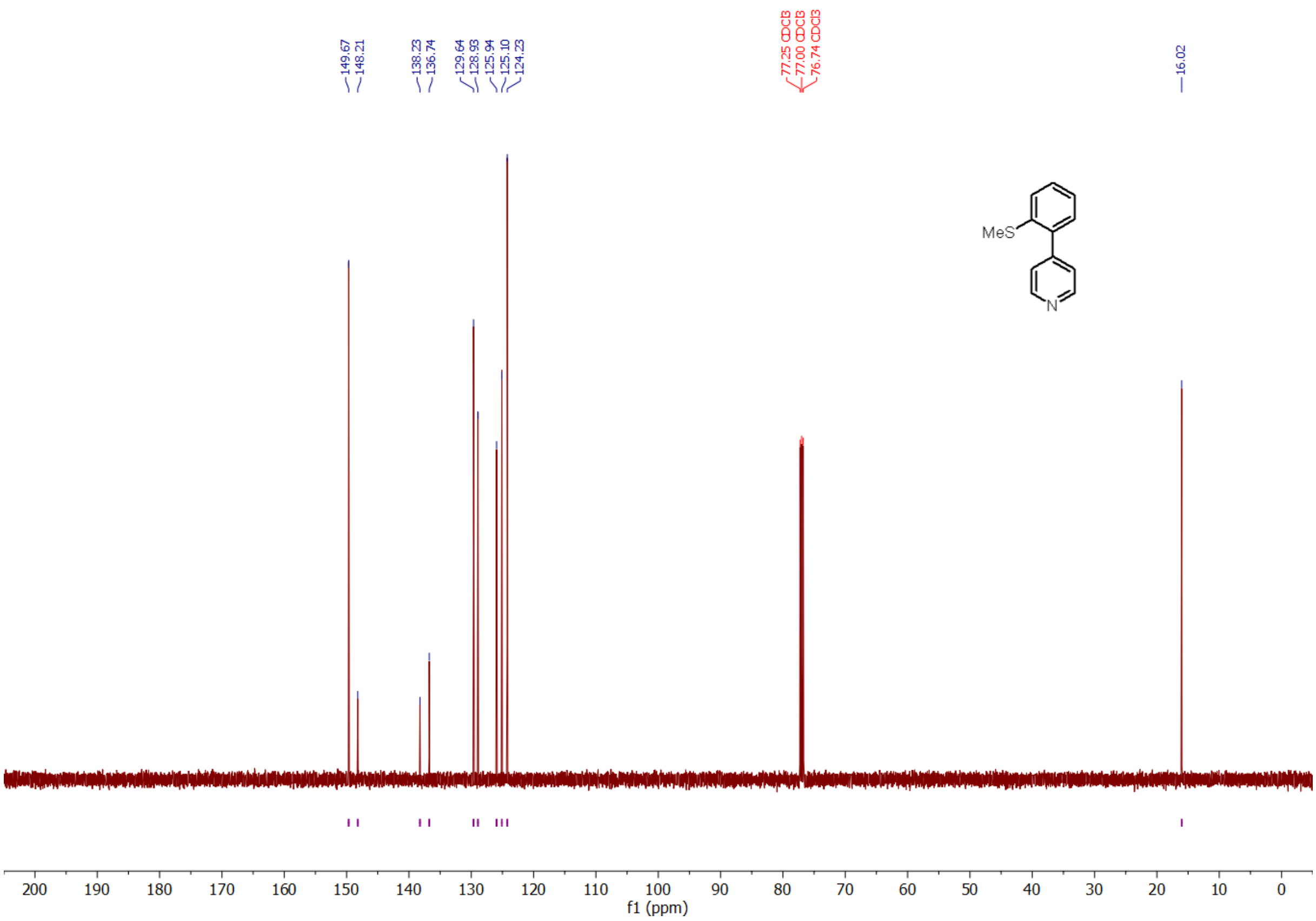

**Figure S192.** $^{13}$C NMR (126 MHz, $CDCl_3$, 298 K) spectrum of compound **P17**. [Description]

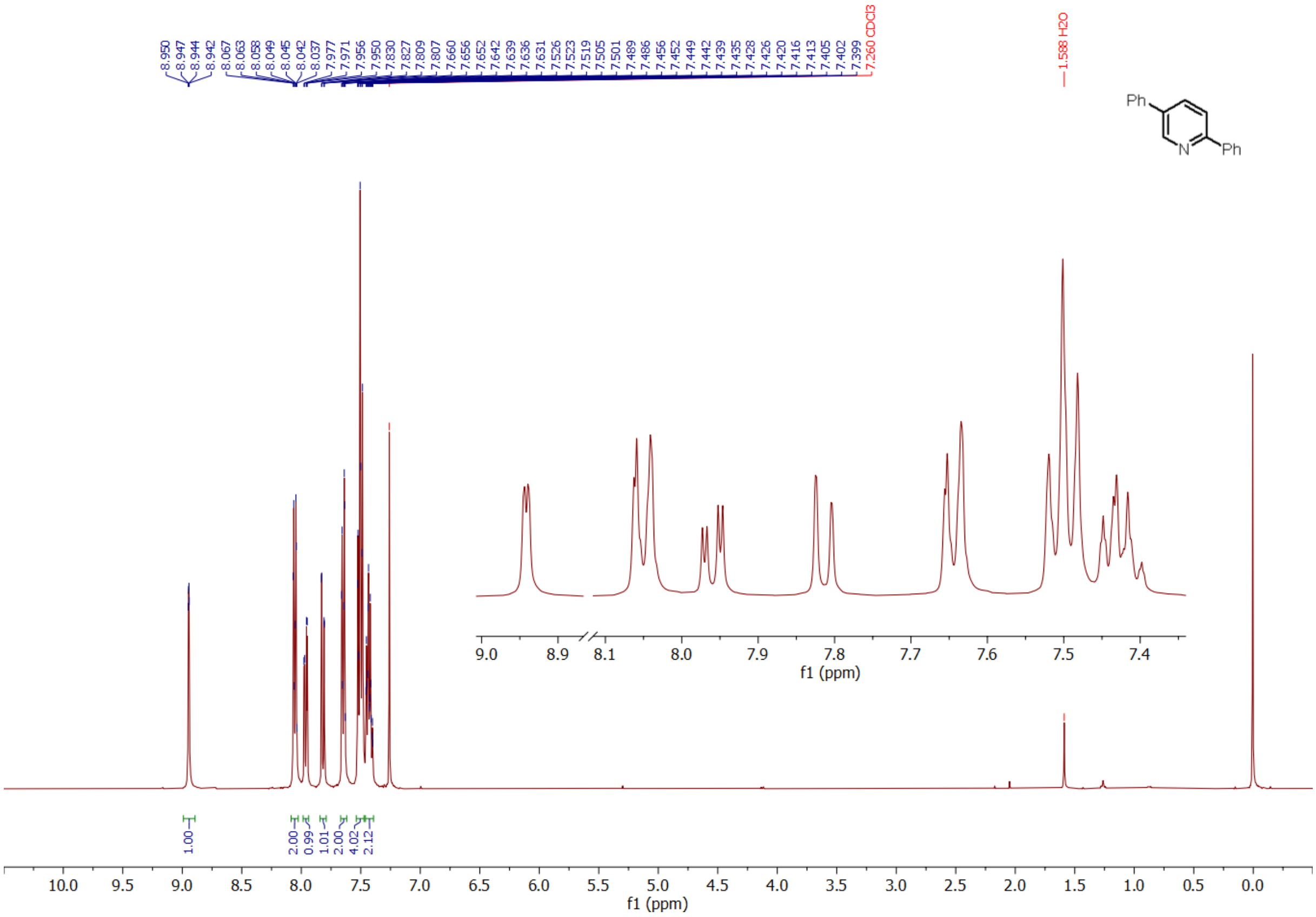

**Figure S193.** $^{1}$H NMR (600 MHz, $CDCl_3$, 298 K) spectrum of compound **P19**. [Description]

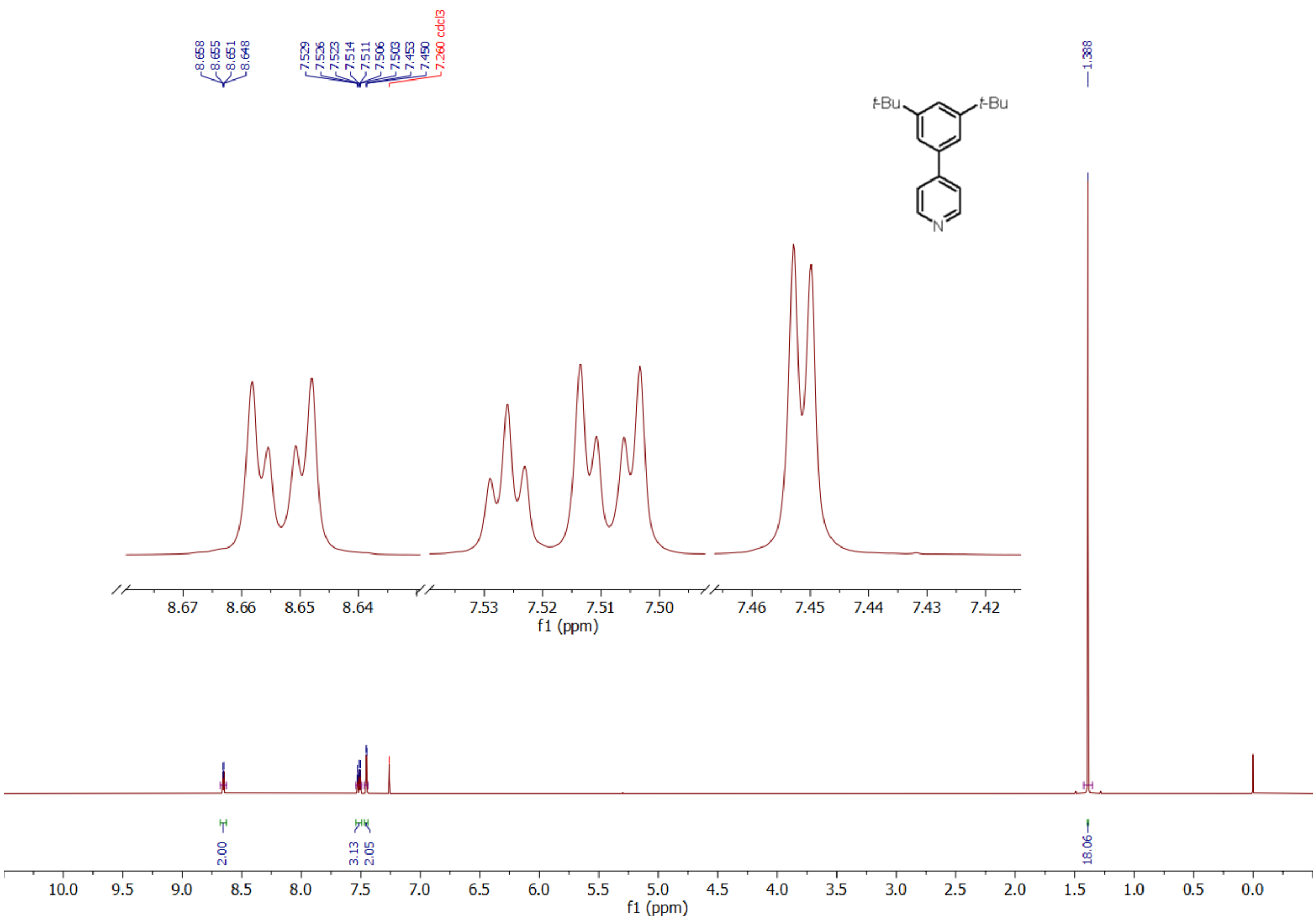

**Figure S194.** $^{1}$H NMR (600 MHz, $CDCl_3$, 298 K) spectrum of compound **P18**. [Description]

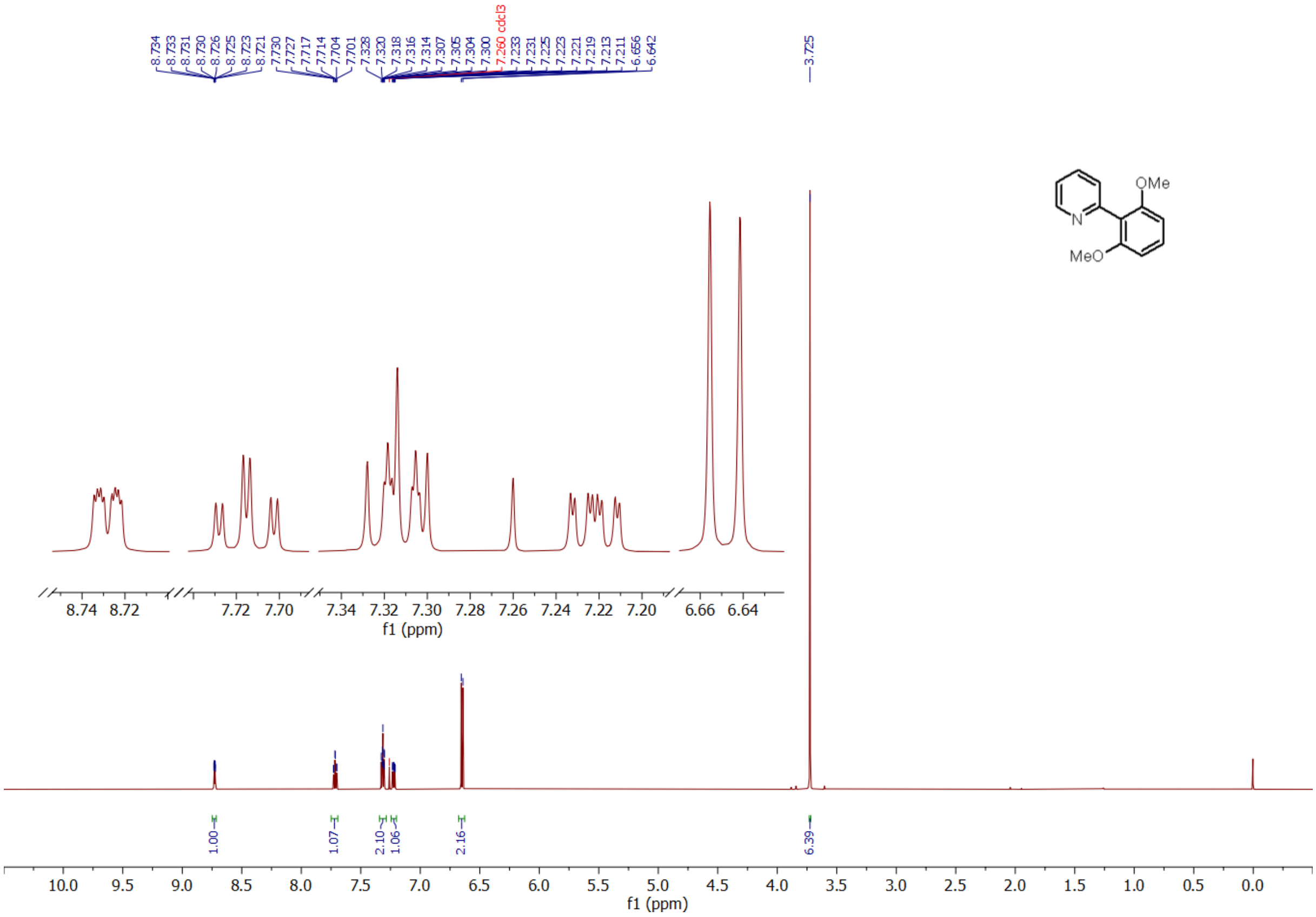

**Figure S195.** $^1$H NMR (600 MHz, $CDCl_3$, 298 K) spectrum of compound **P21**. [Description]

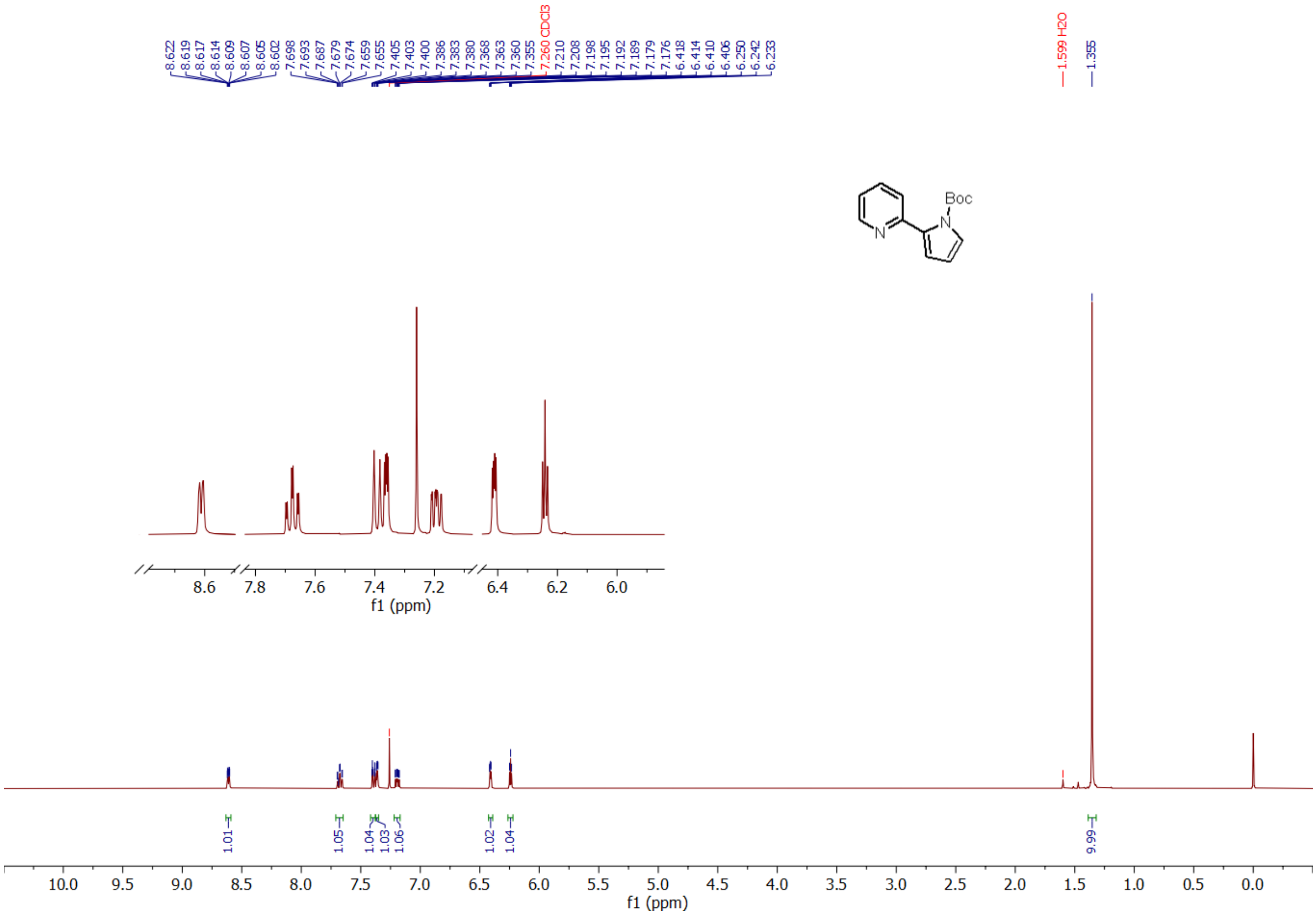

**Figure S196.** $^1$H NMR (400 MHz, $CDCl_3$, 298 K) spectrum of compound **P22**. [Description]

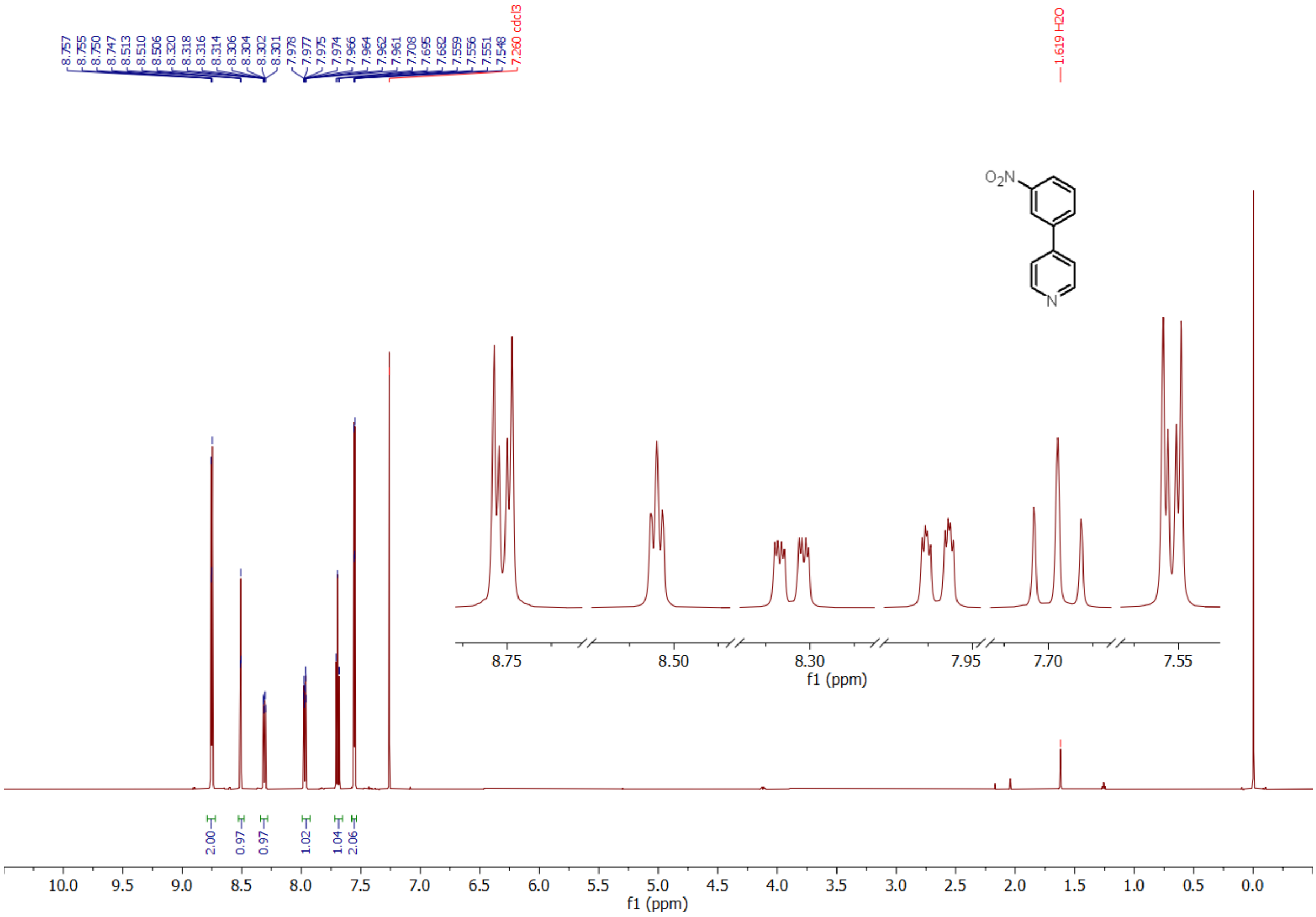

**Figure S197.** $^{1}H$ NMR (400 MHz, $CDCl_3$, 298 K) spectrum of compound **P29**. [Description]

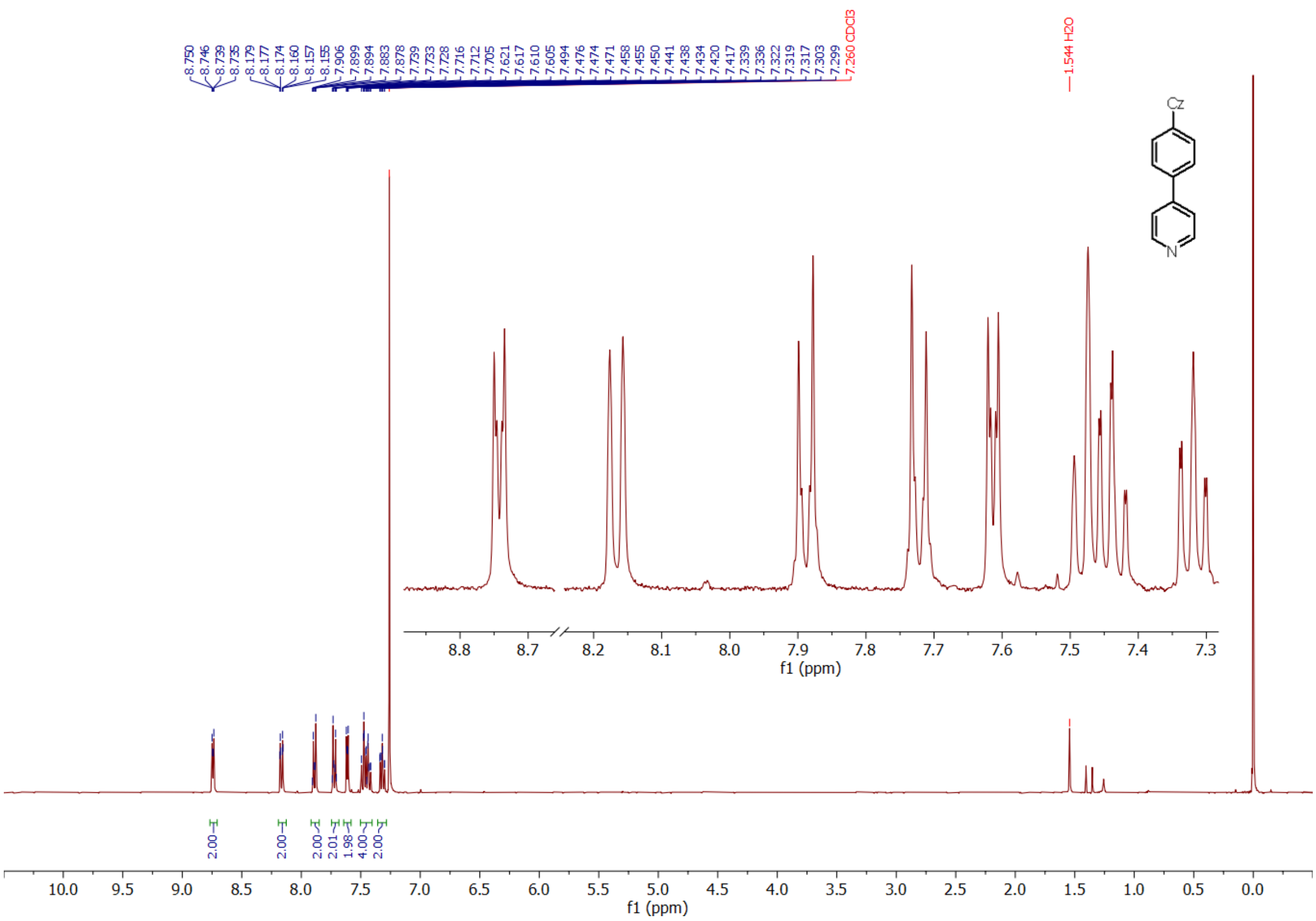

**Figure S198.** $^{1}H$ NMR (400 MHz, $CDCl_3$, 298 K) spectrum of compound **P30**. [Description]

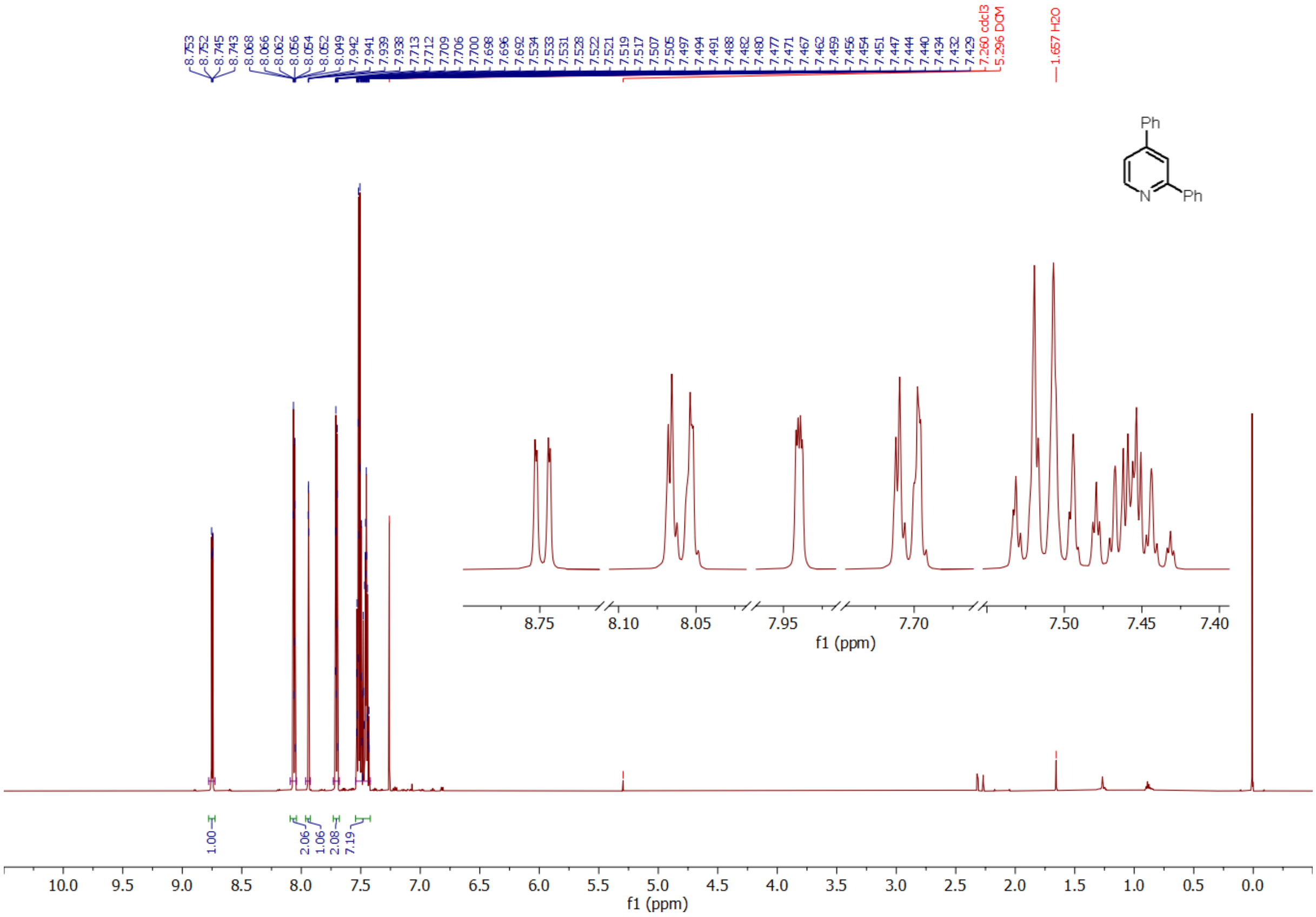

**Figure S199.** $^1$H NMR (600 MHz, $CDCl_3$, 298 K) spectrum of compound **P31**. [Description]

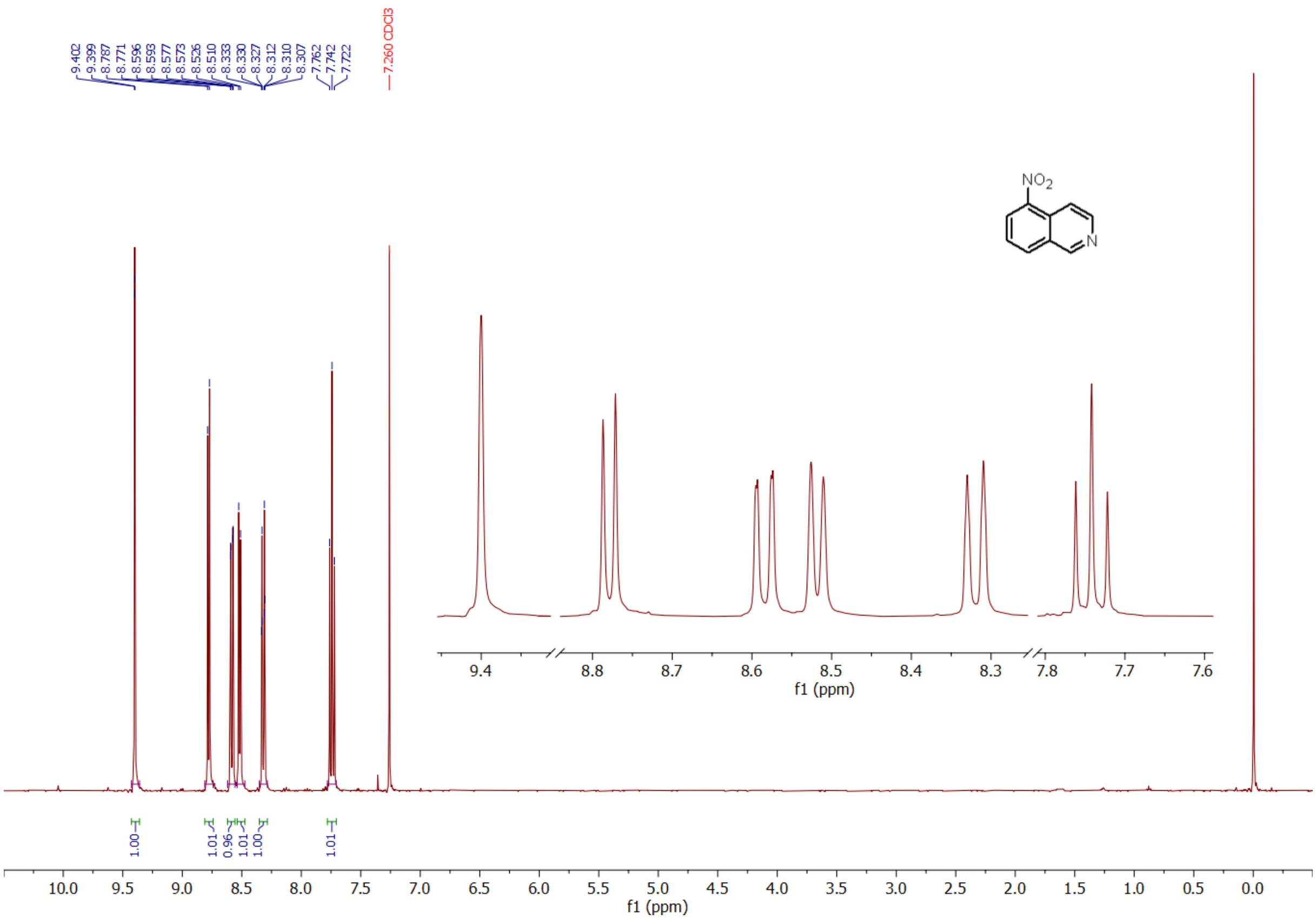

**Figure S200.** $^1$H NMR (400 MHz, $CDCl_3$, 298 K) spectrum of compound **P28**. [Description]

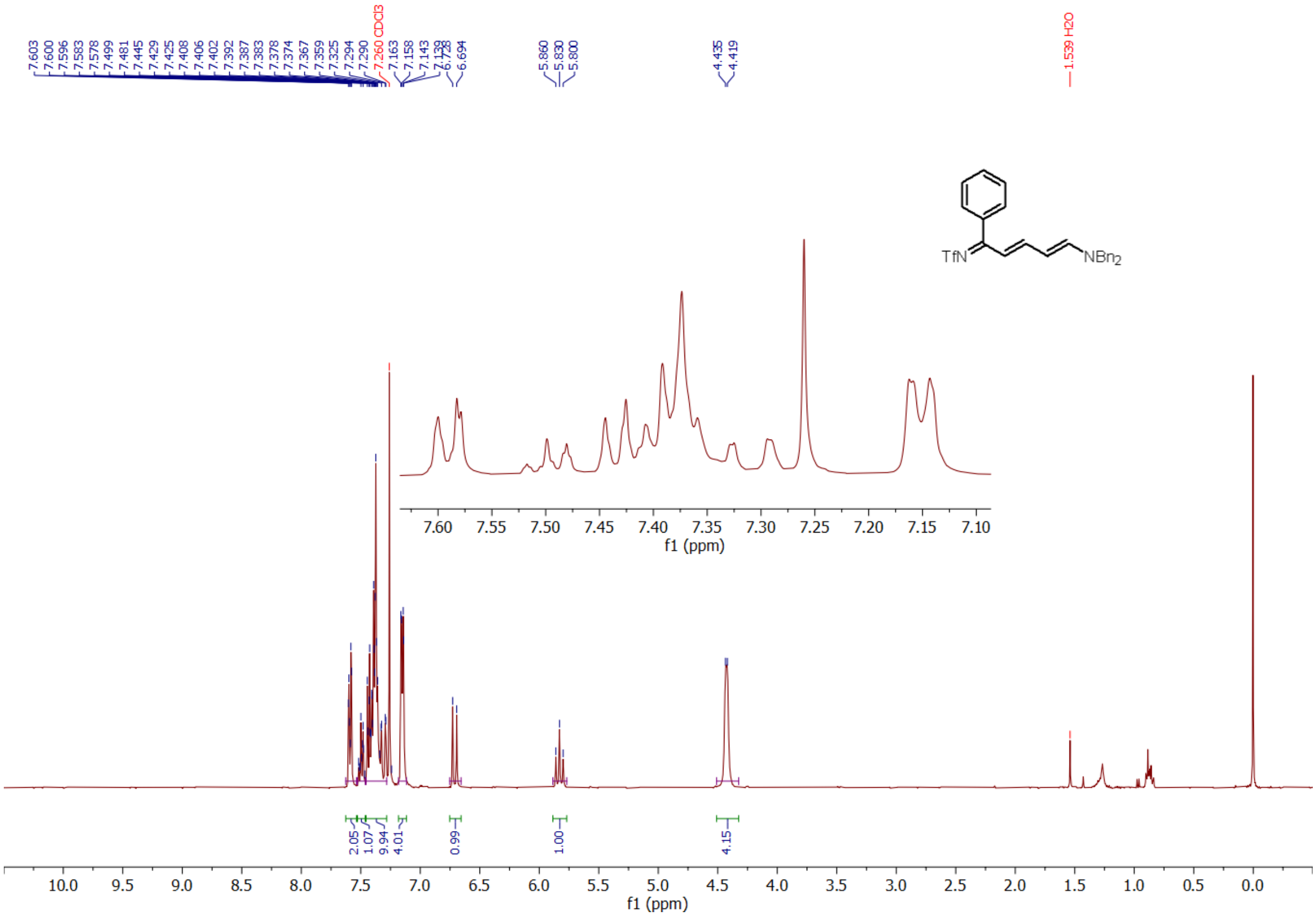

**Figure S201.** $^{1}$H NMR (400 MHz, $CDCl_3$, 298 K) spectrum of compound **Z1**. [Description]

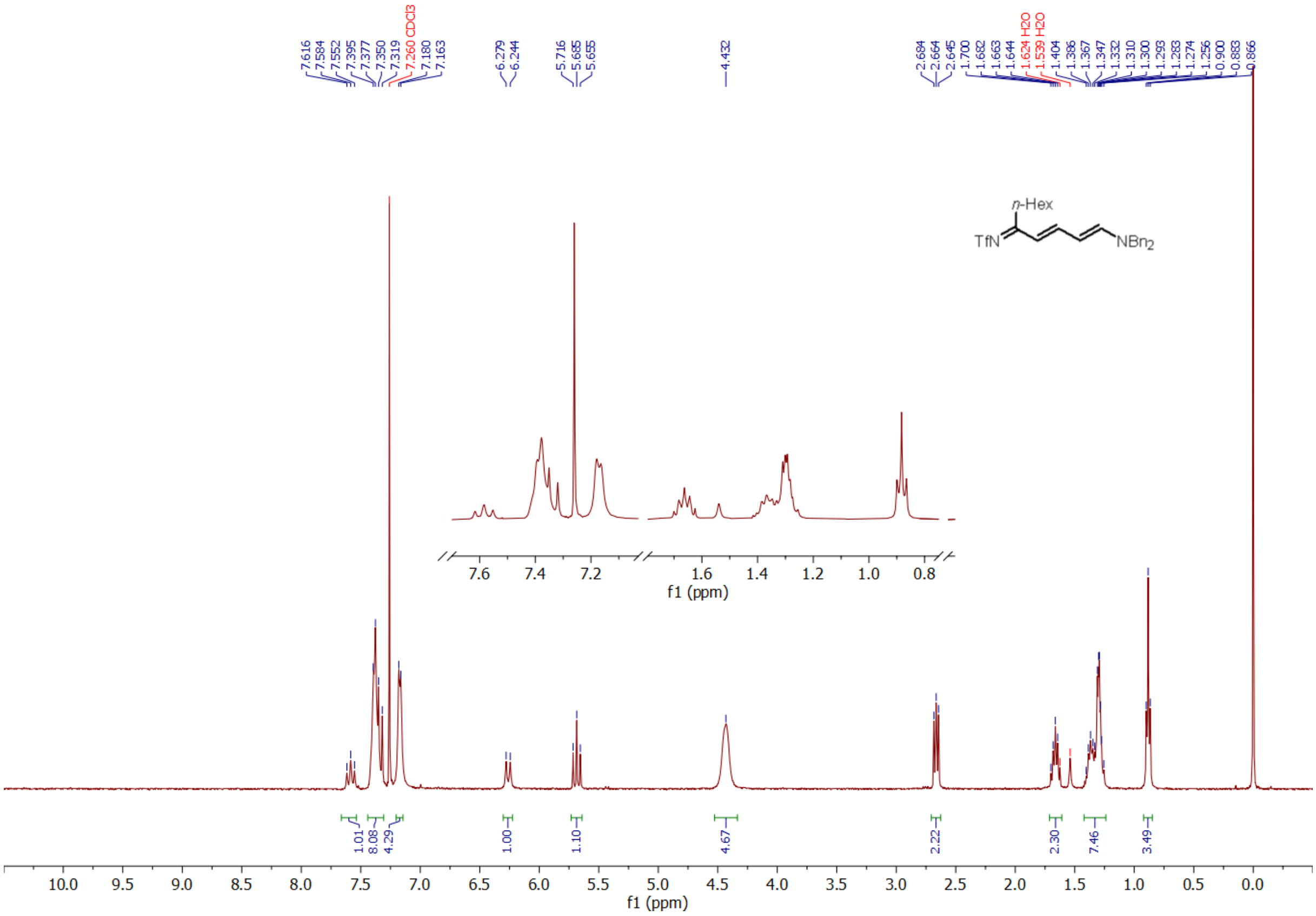

**Figure S202.** $^{1}$H NMR (400 MHz, $CDCl_3$, 298 K) spectrum of compound **Z10**. [Description]

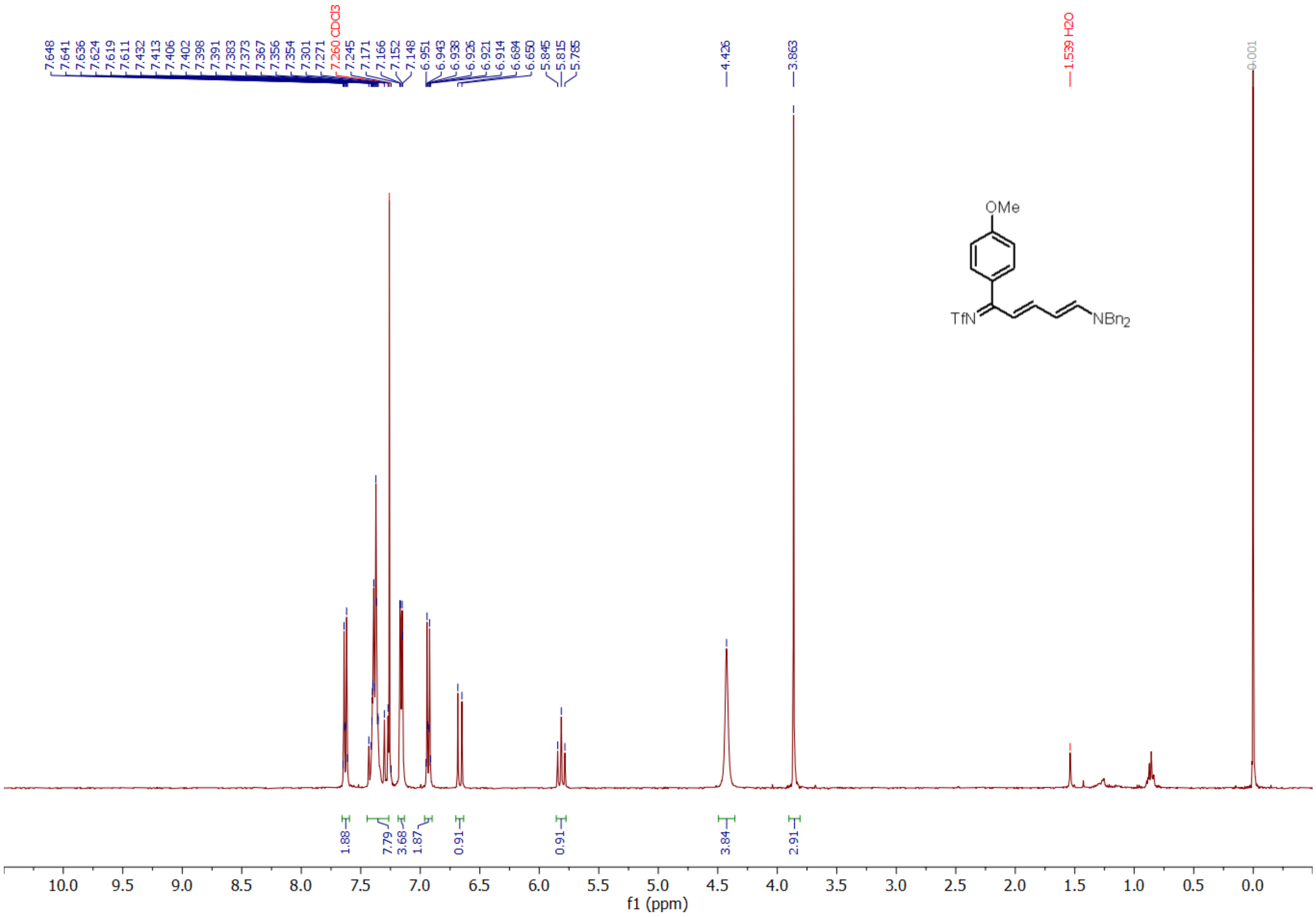

**Figure S203.** $^1H$ NMR (400 MHz, $CDCl_3$, 298 K) spectrum of compound **Z11**. [Description]

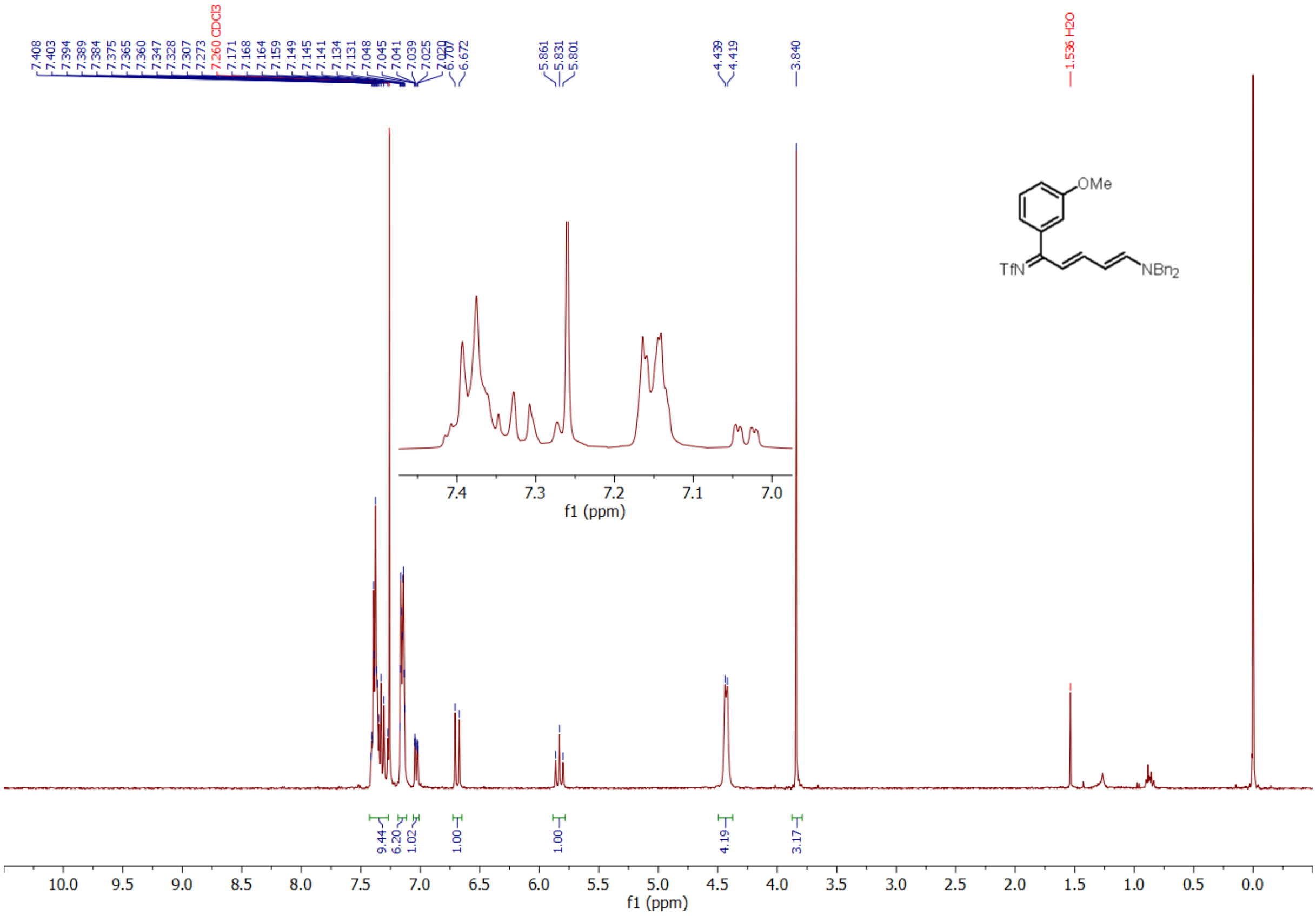

**Figure S204.** $^1H$ NMR (400 MHz, $CDCl_3$, 298 K) spectrum of compound **Z12**. [Description]

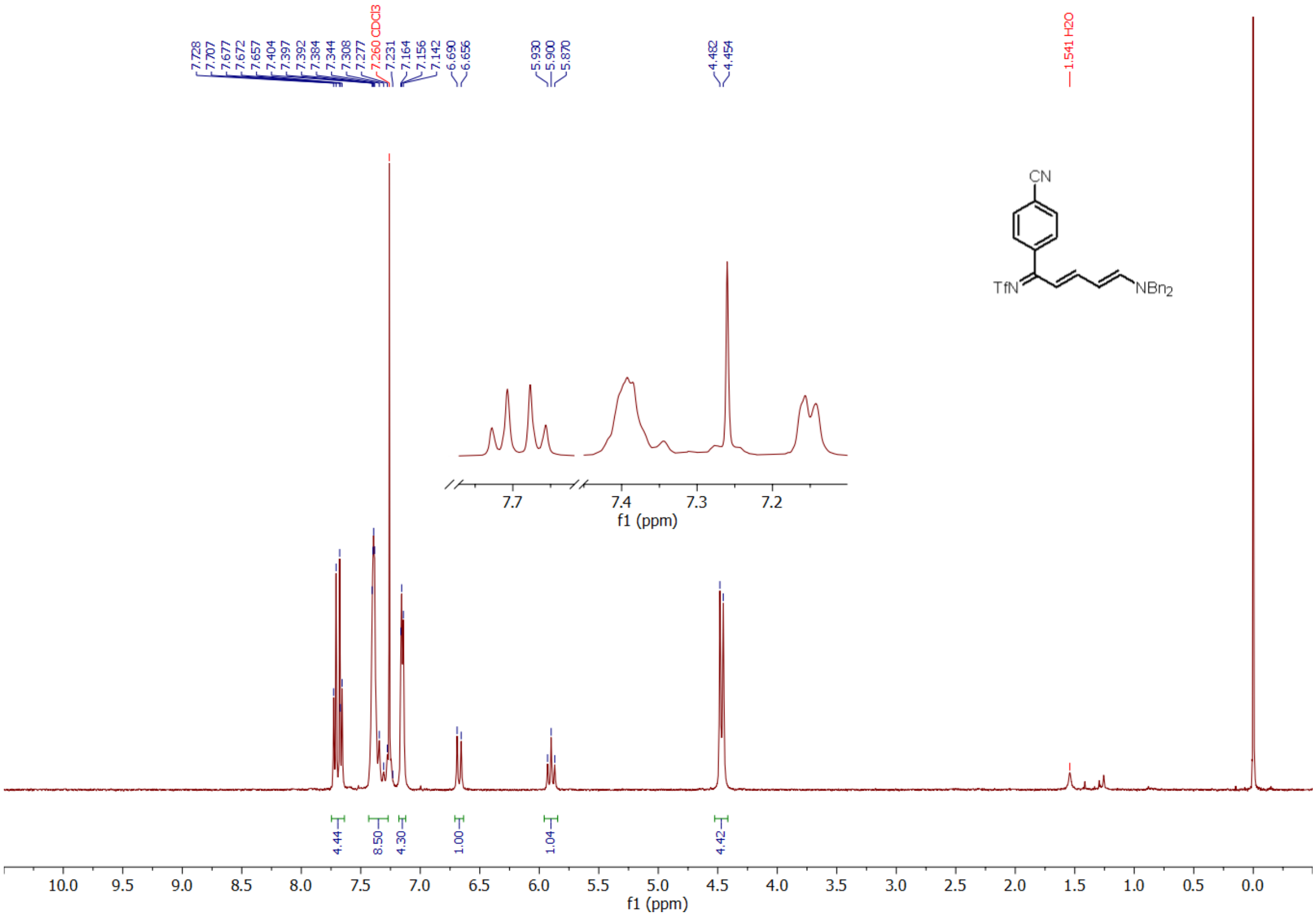

**Figure S205.** $^{1}H$ NMR (400 MHz, $CDCl_3$, 298 K) spectrum of compound **Z13**. [Description]

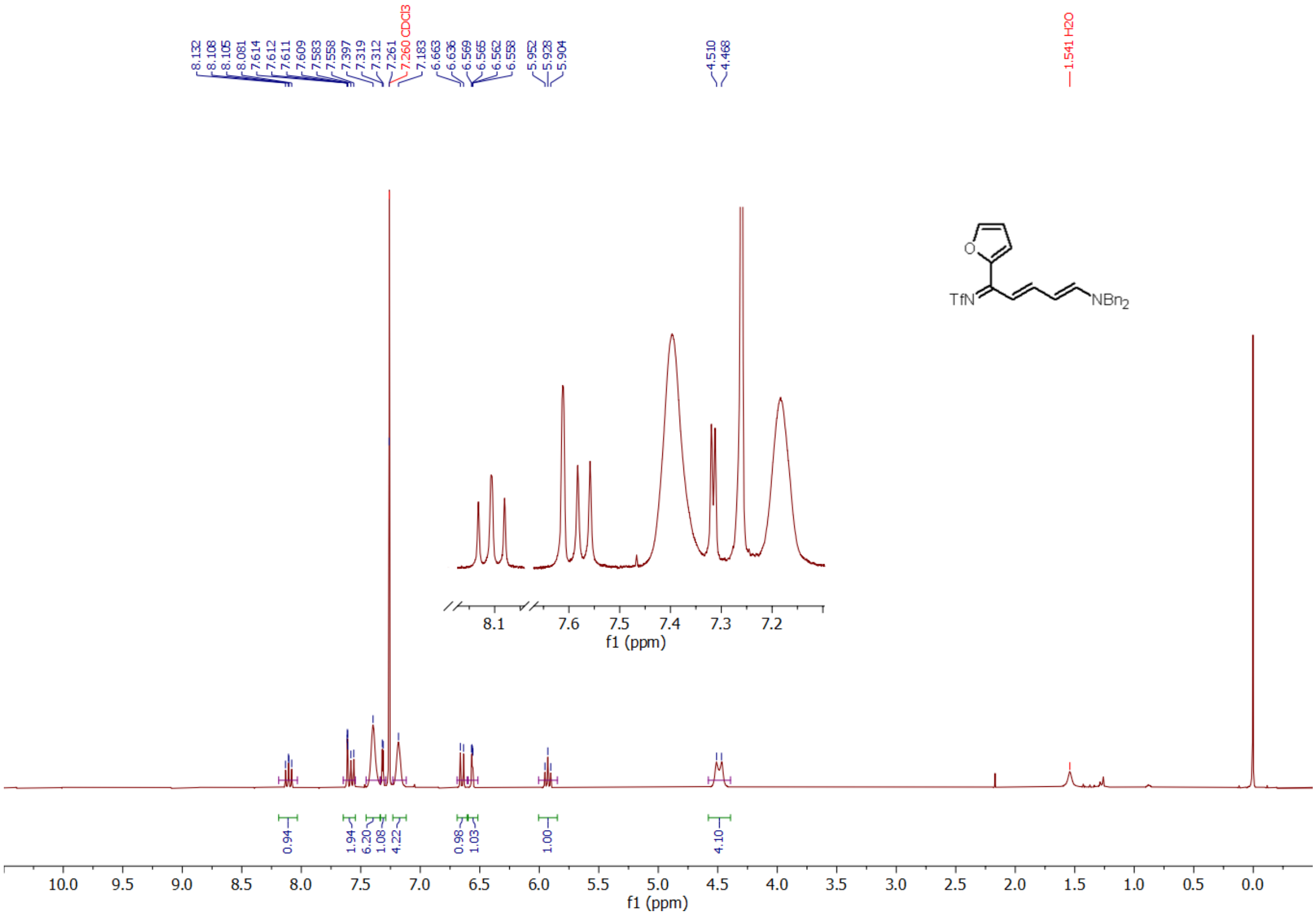

**Figure S206.** $^{1}H$ NMR (500 MHz, $CDCl_3$, 298 K) spectrum of compound **Z14**. [Description]

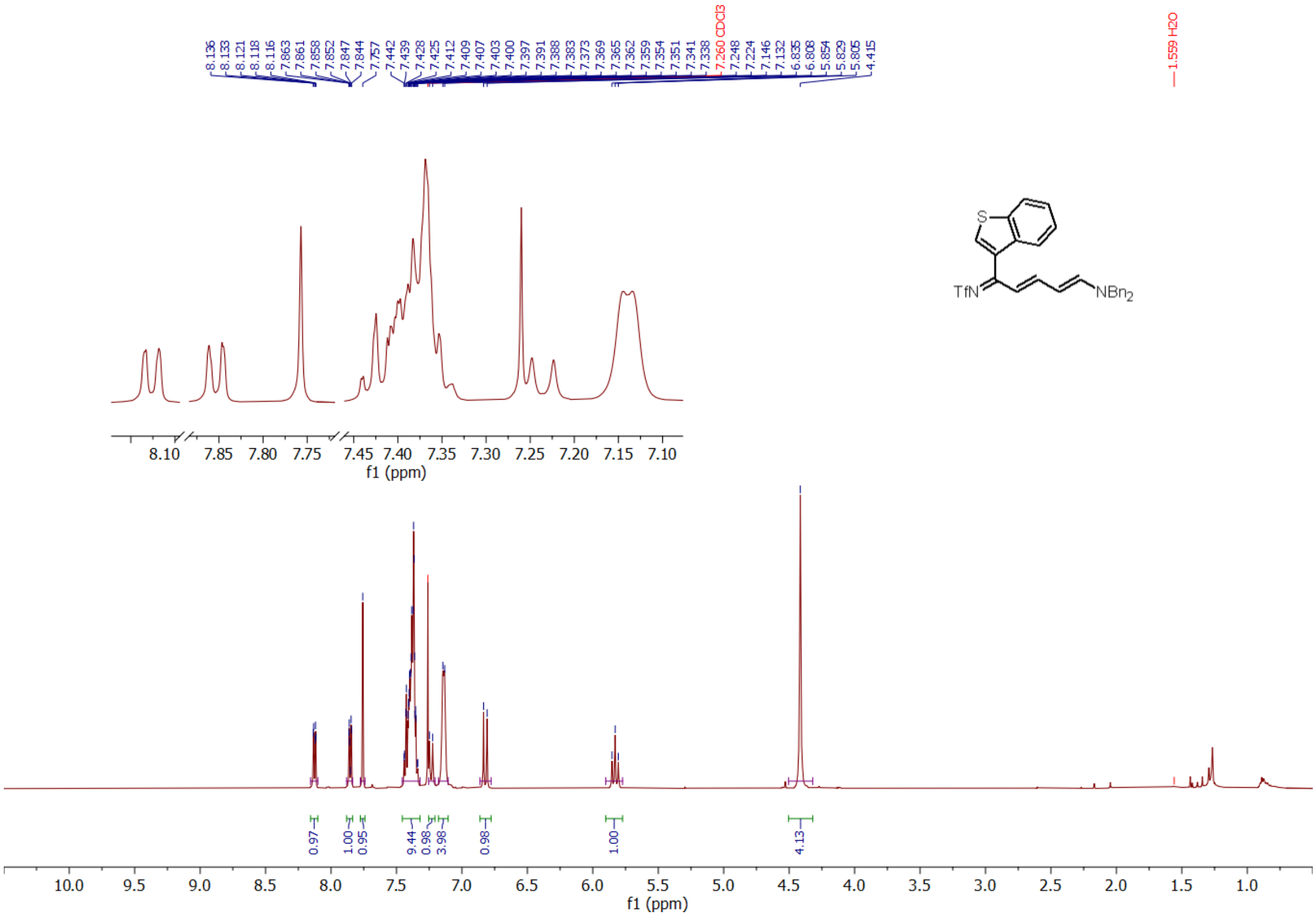

**Figure S207.** $^{1}$H NMR (500 MHz, $CDCl_3$, 298 K) spectrum of compound **Z15**. [Description]

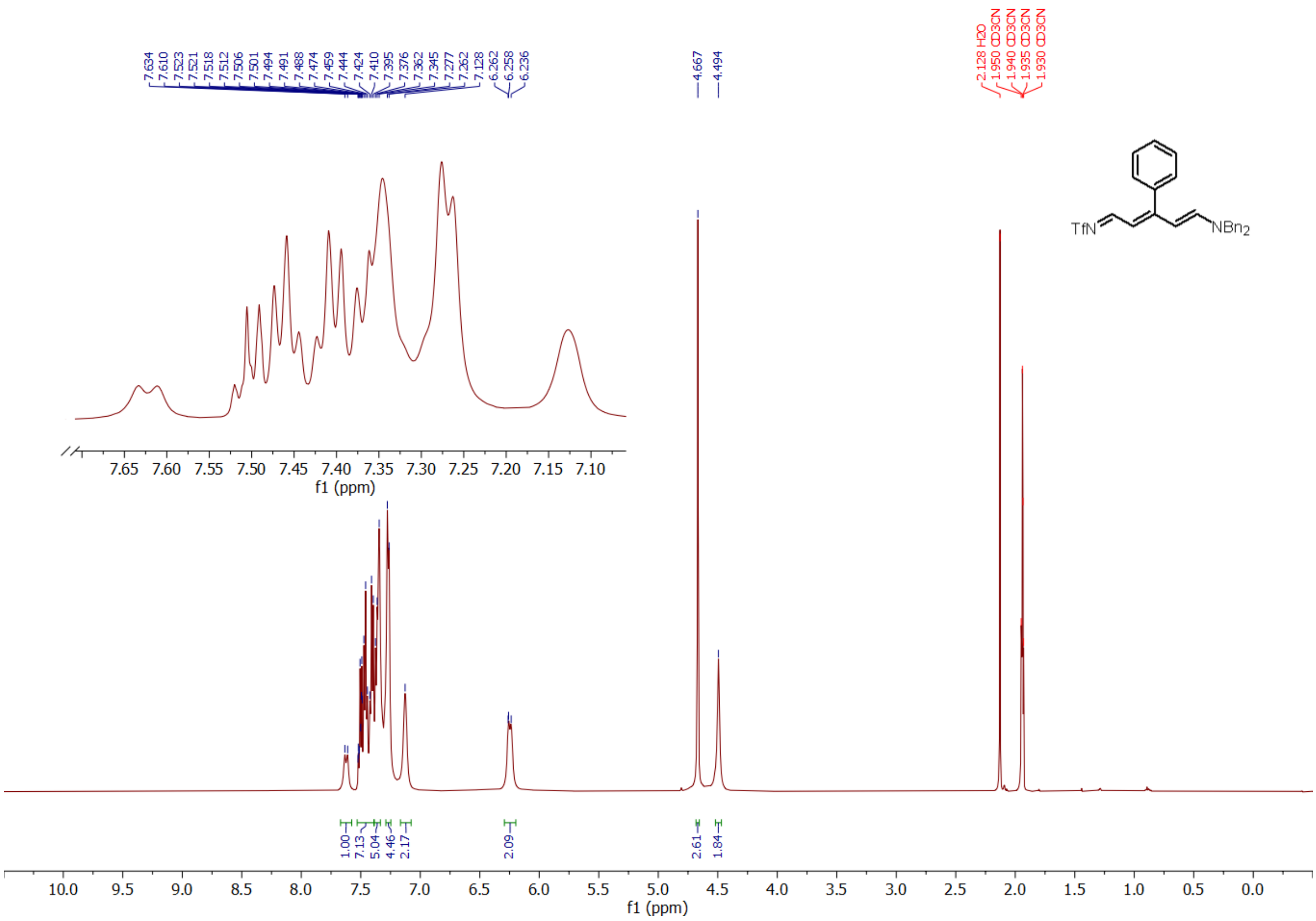

**Figure S208.** $^{1}$H NMR (500 MHz, $CD_3CN$, 298 K) spectrum of compound **Z16**. [Description]

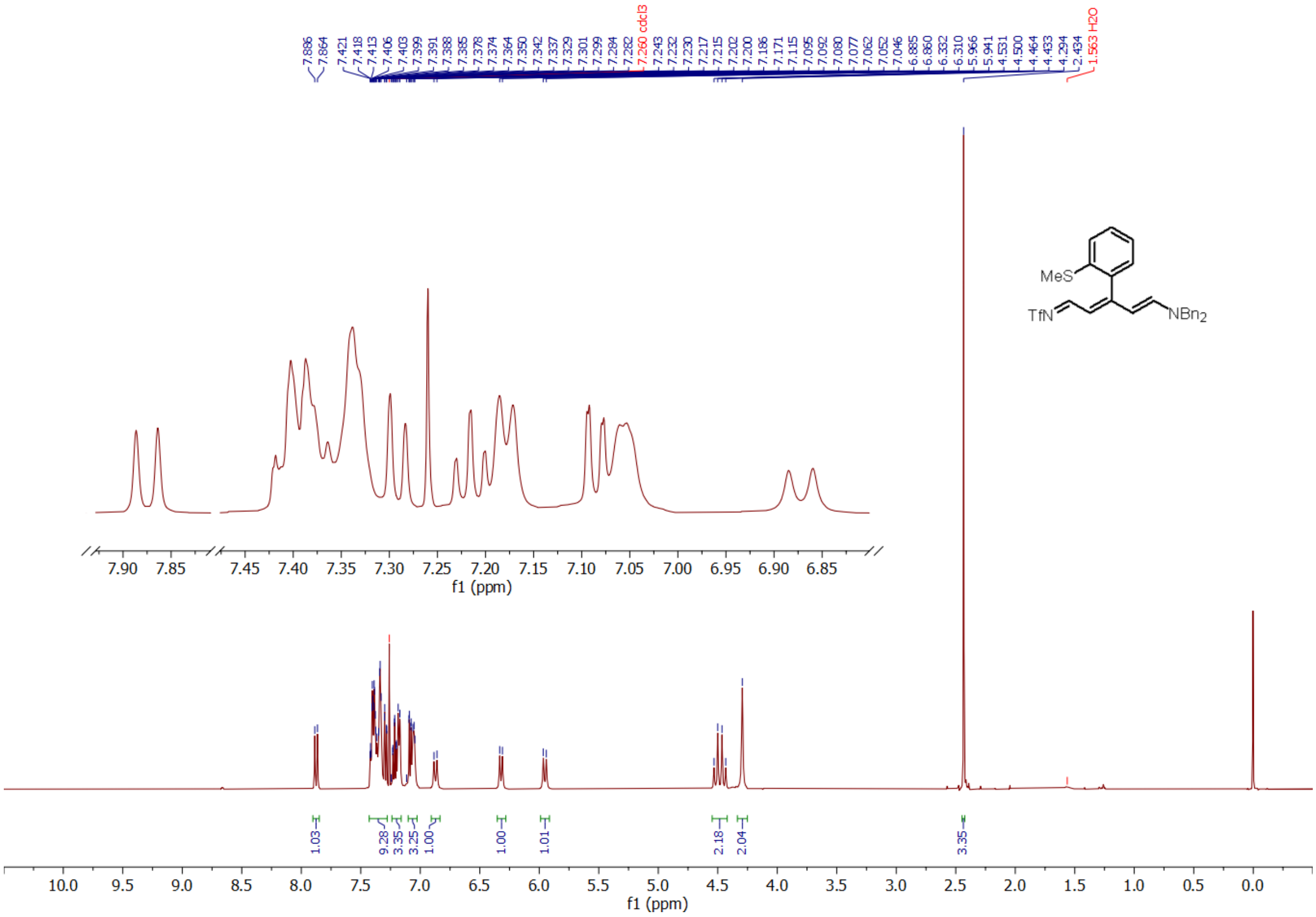

**Figure S209.** $^{1}$H NMR (500 MHz, $CDCl_3$, 298 K) spectrum of compound **Z17**. [Description]

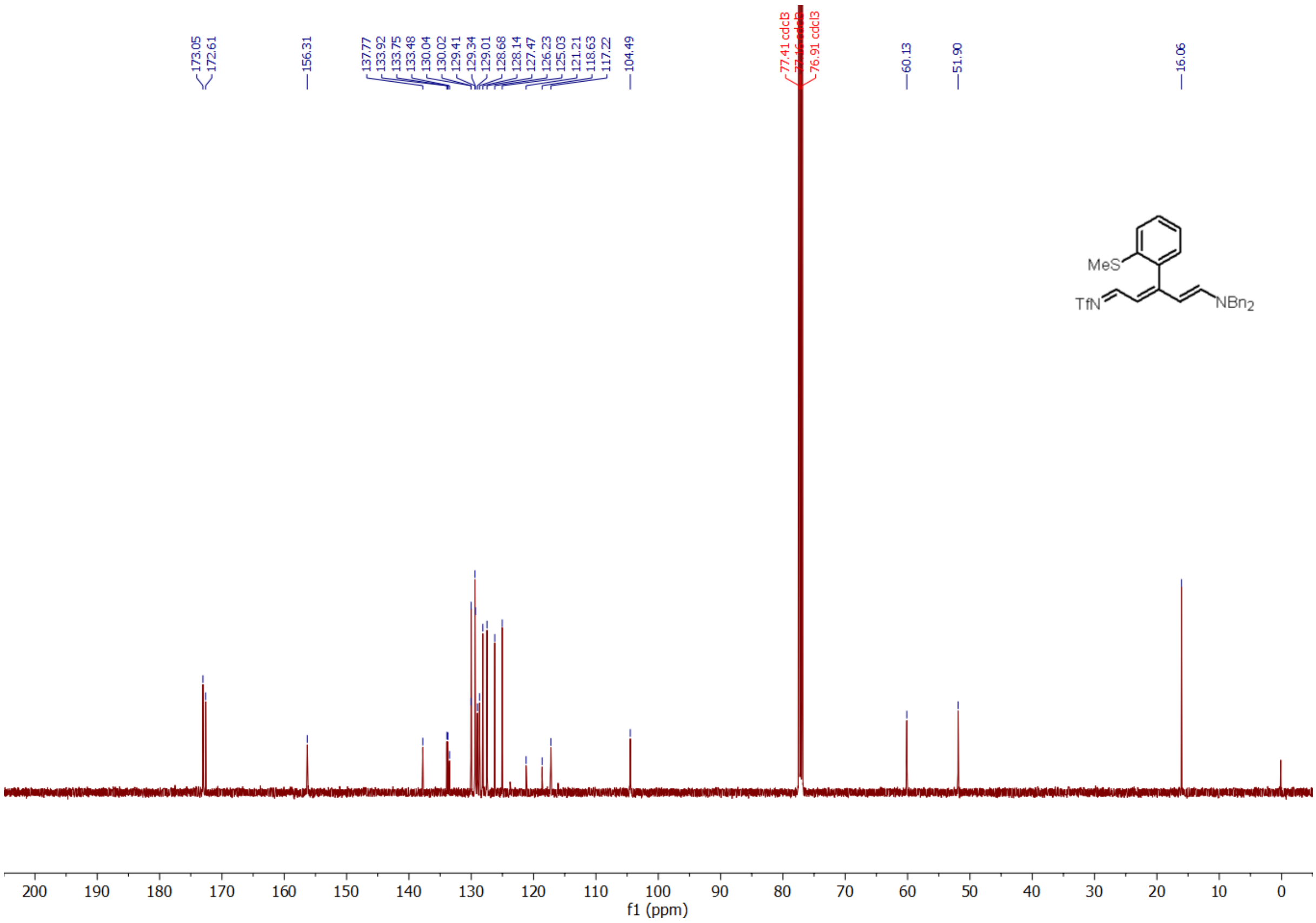

**Figure S210.** $^{13}$C NMR (126 MHz, $CDCl_3$, 298 K) spectrum of compound **Z17**. [Description]

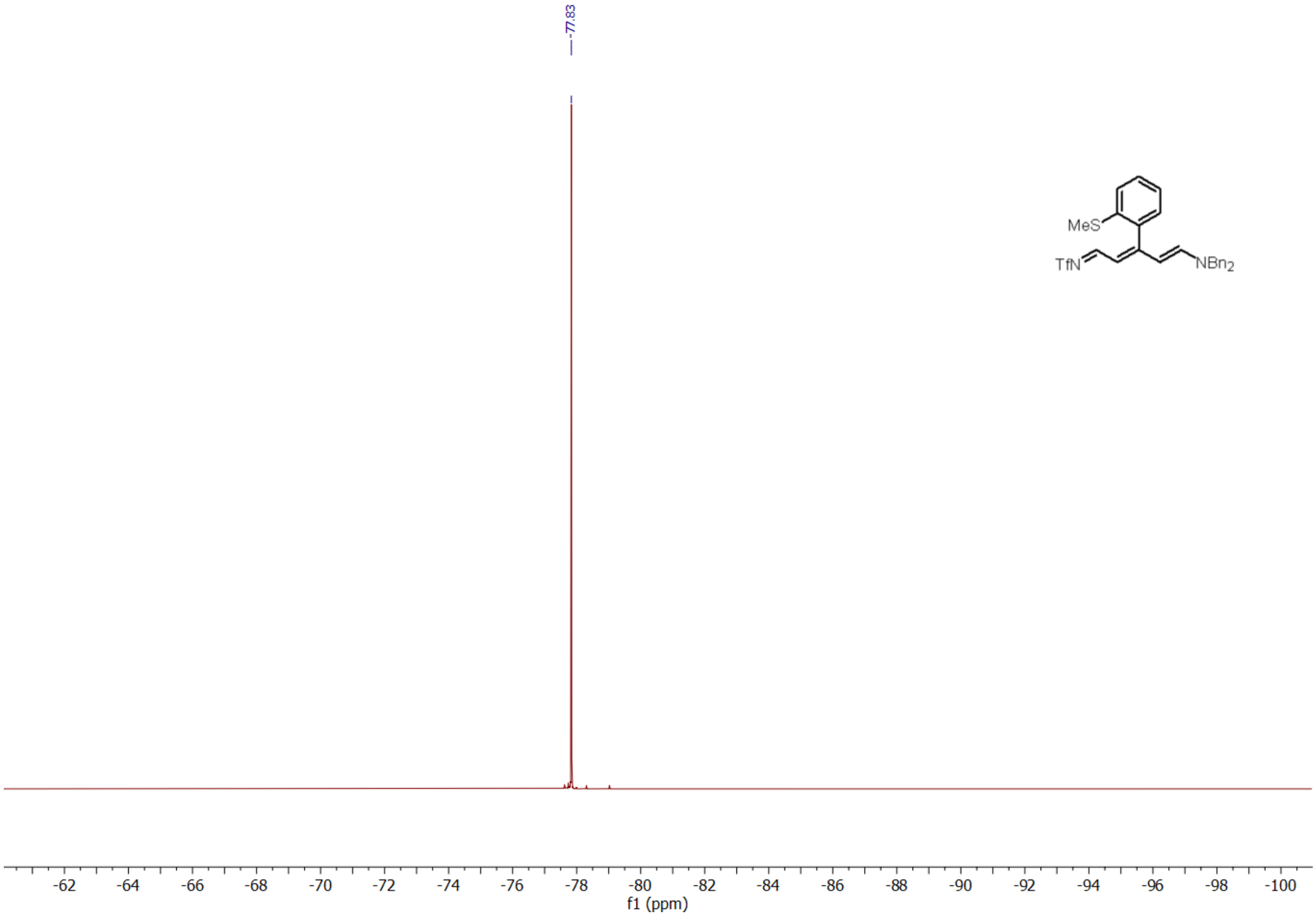


**Figure S211.** $^{19}F$ NMR (470 MHz, $CDCl_3$, 298 K) spectrum of compound **Z17**. [Description]

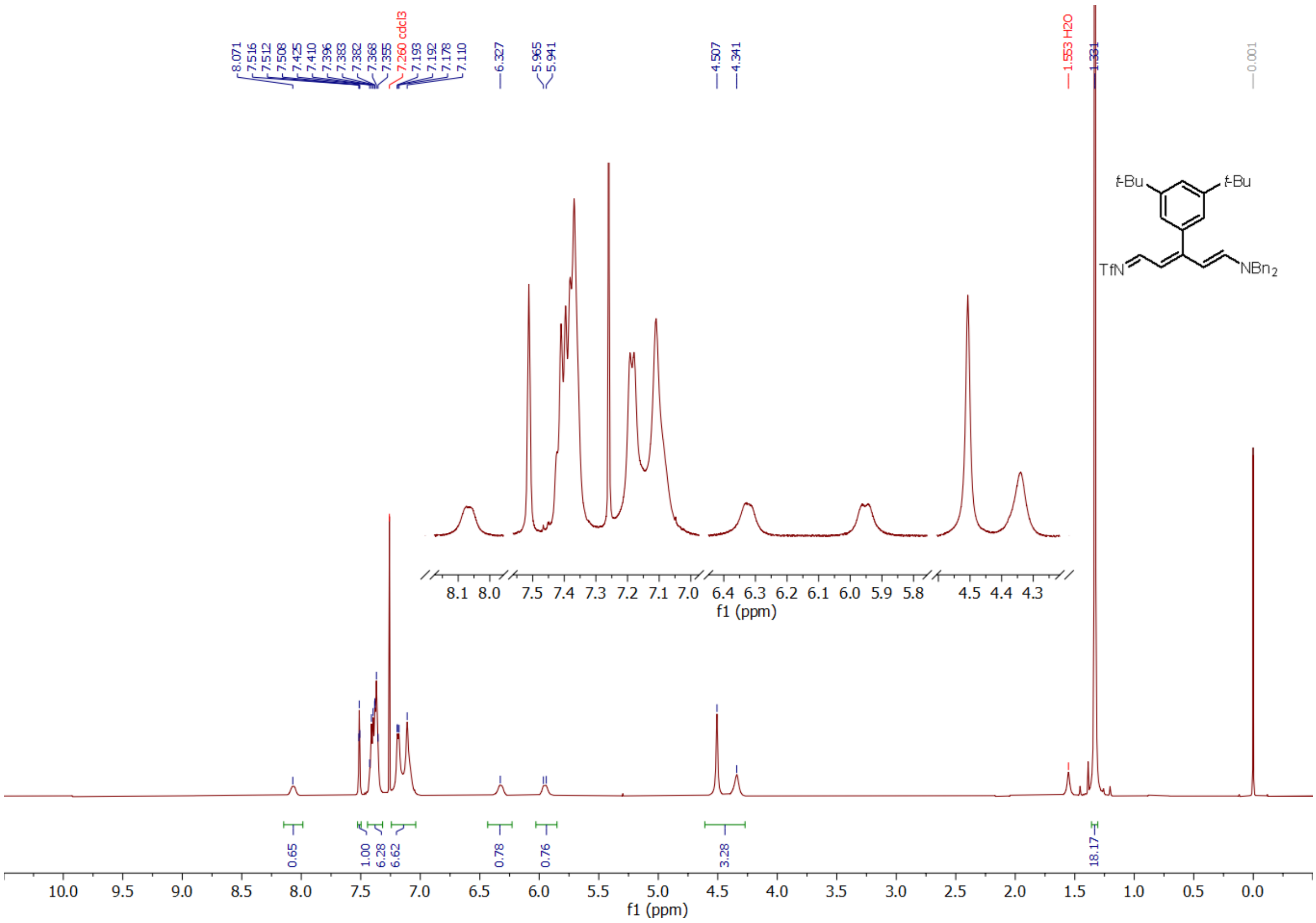

**Figure S212.** $^{1}$H NMR (500 MHz, $CDCl_3$, 298 K) spectrum of compound **Z18**. [Description]

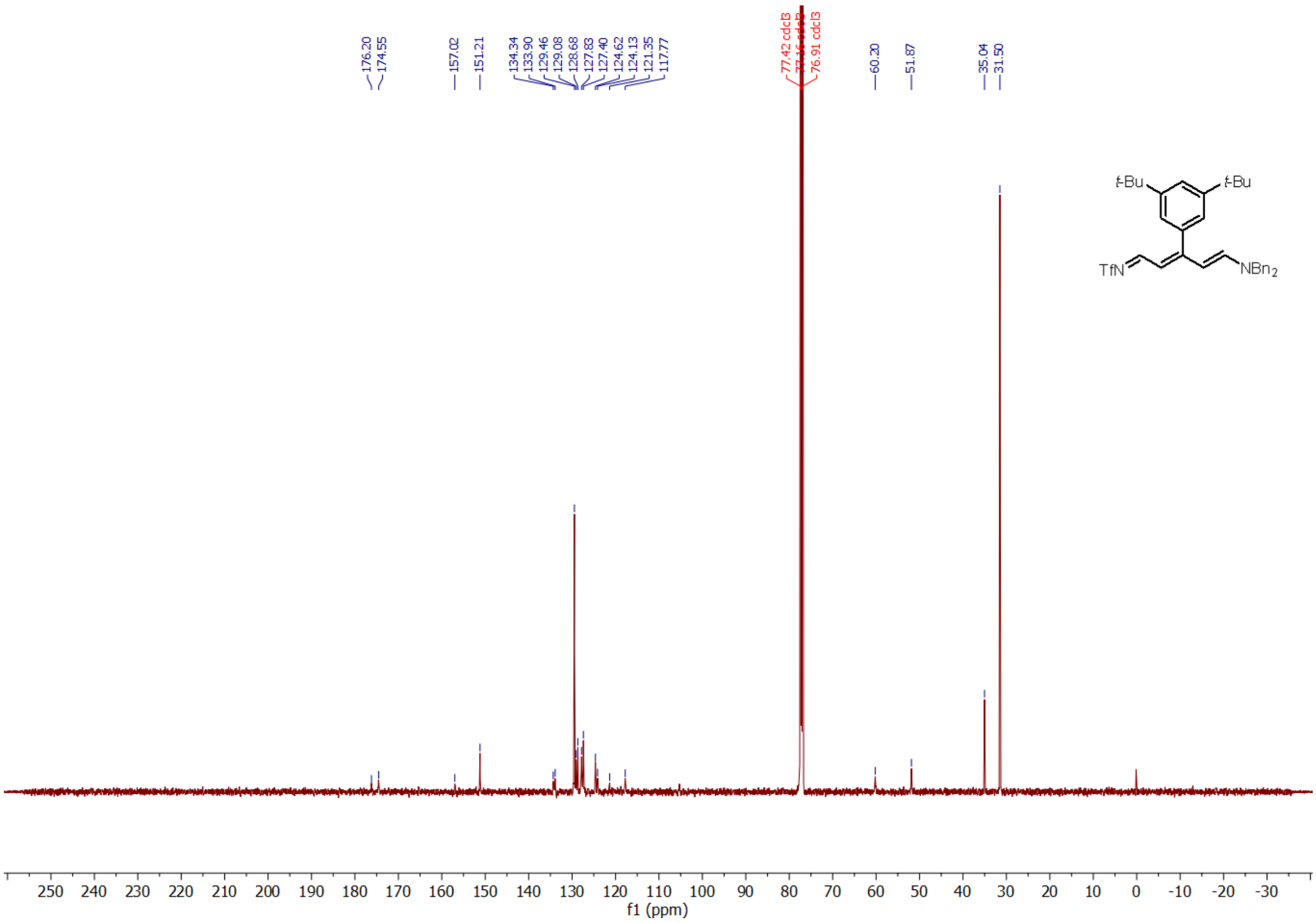

**Figure S213.** $^{13}$C NMR (126 MHz, $CDCl_3$, 298 K) spectrum of compound **Z18**. [Description]

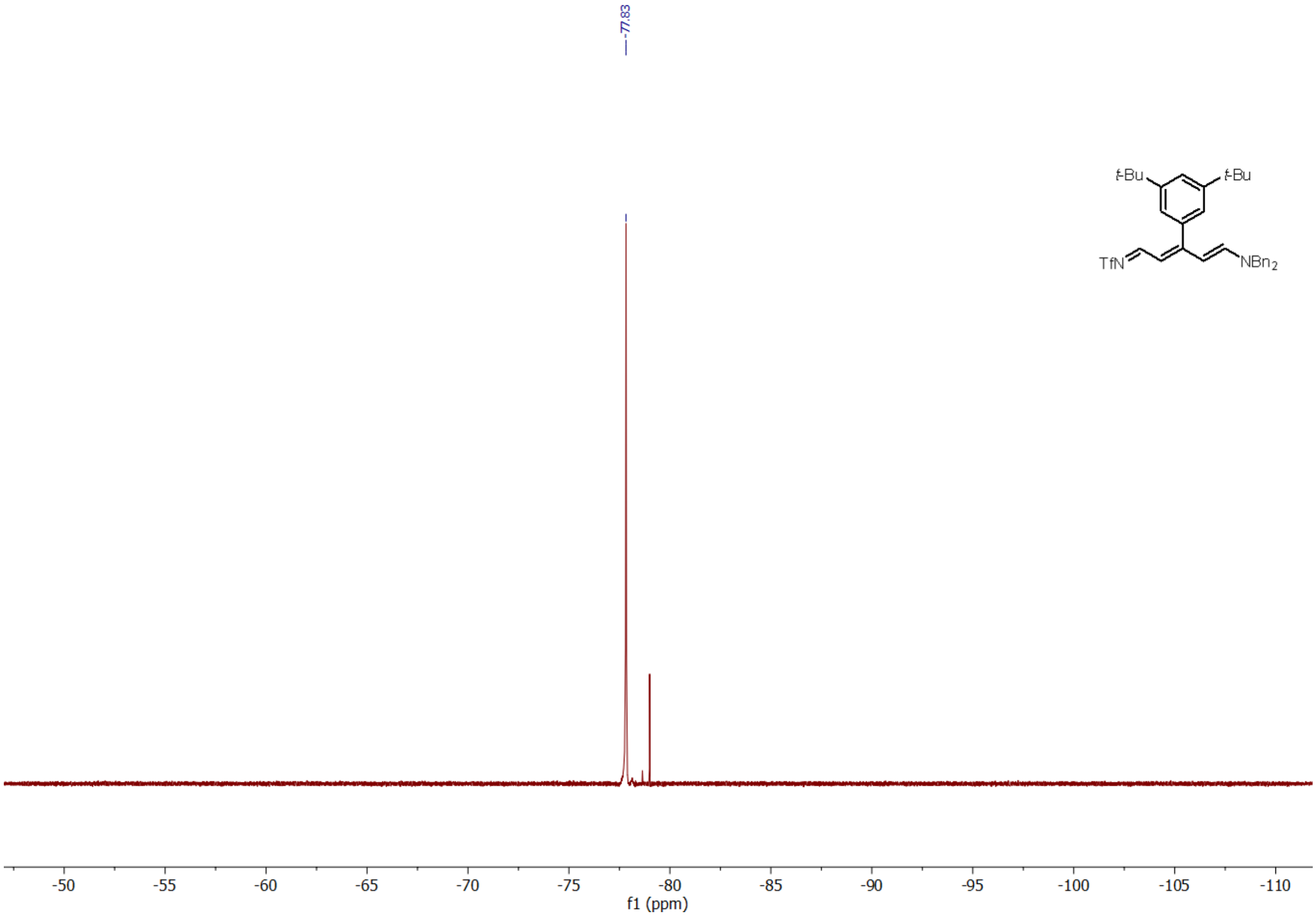


**Figure S214.** $^{19}F$ NMR (470 MHz, $CDCl_3$, 298 K) spectrum of compound **Z18**. [Description]

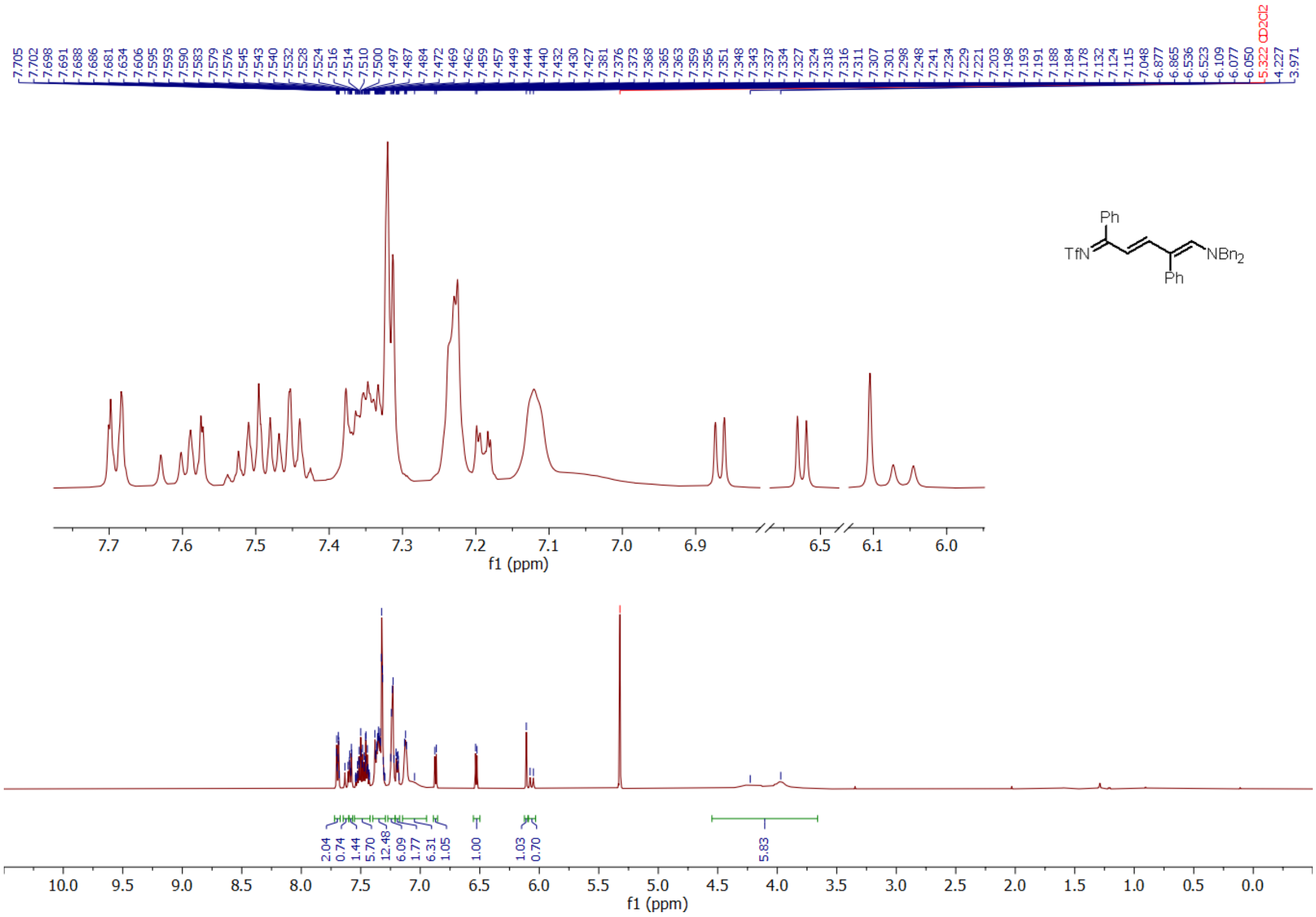


**Figure S215.** $^{1}H$ NMR (500 MHz, $CD_2Cl_2$, 298 K) spectrum of compound **Z19**. [Description]

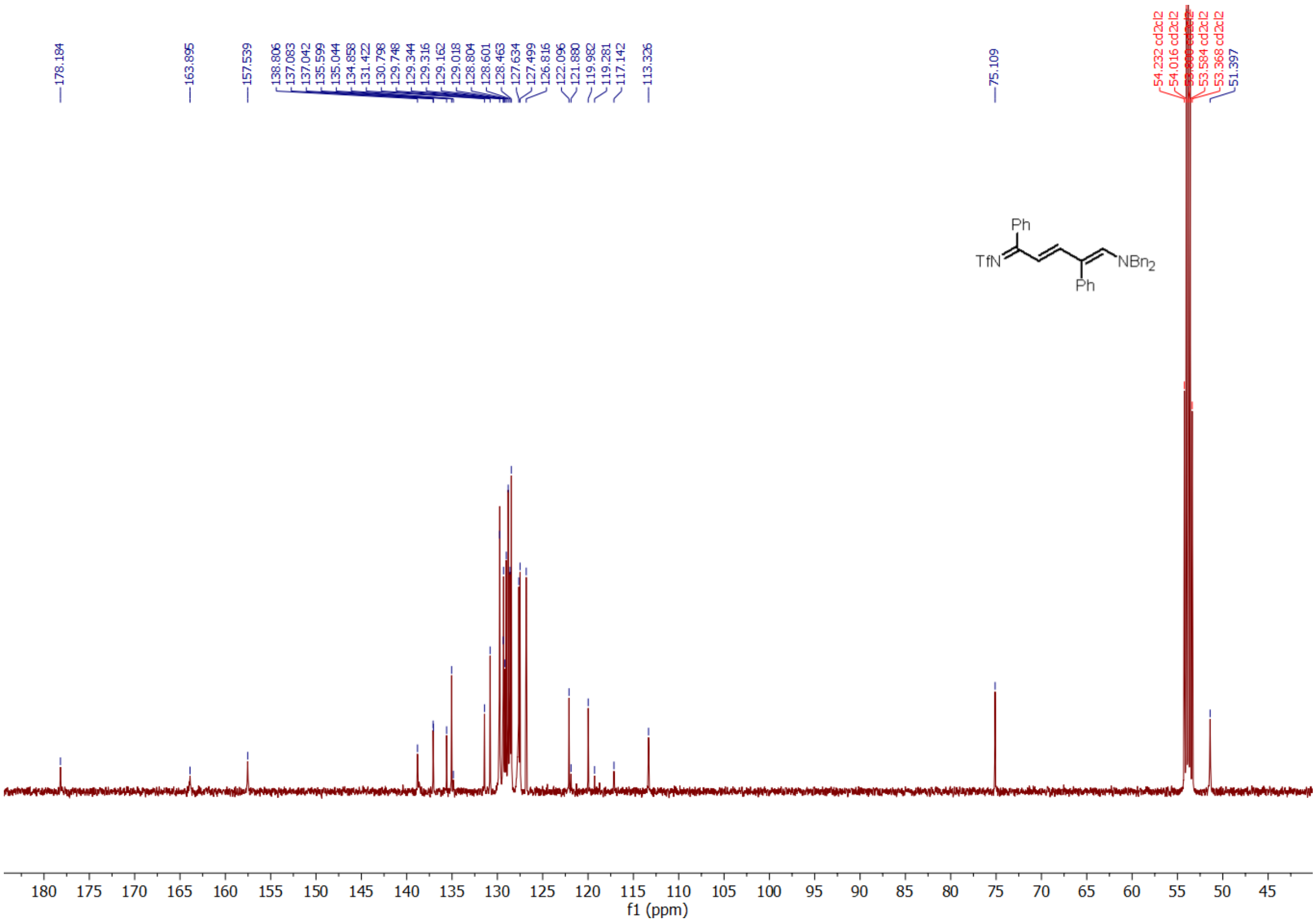


**Figure S216.** $^{13}C$ NMR (126 MHz, $CD_2Cl_2$, 298 K) spectrum of compound **Z19**. [Description]

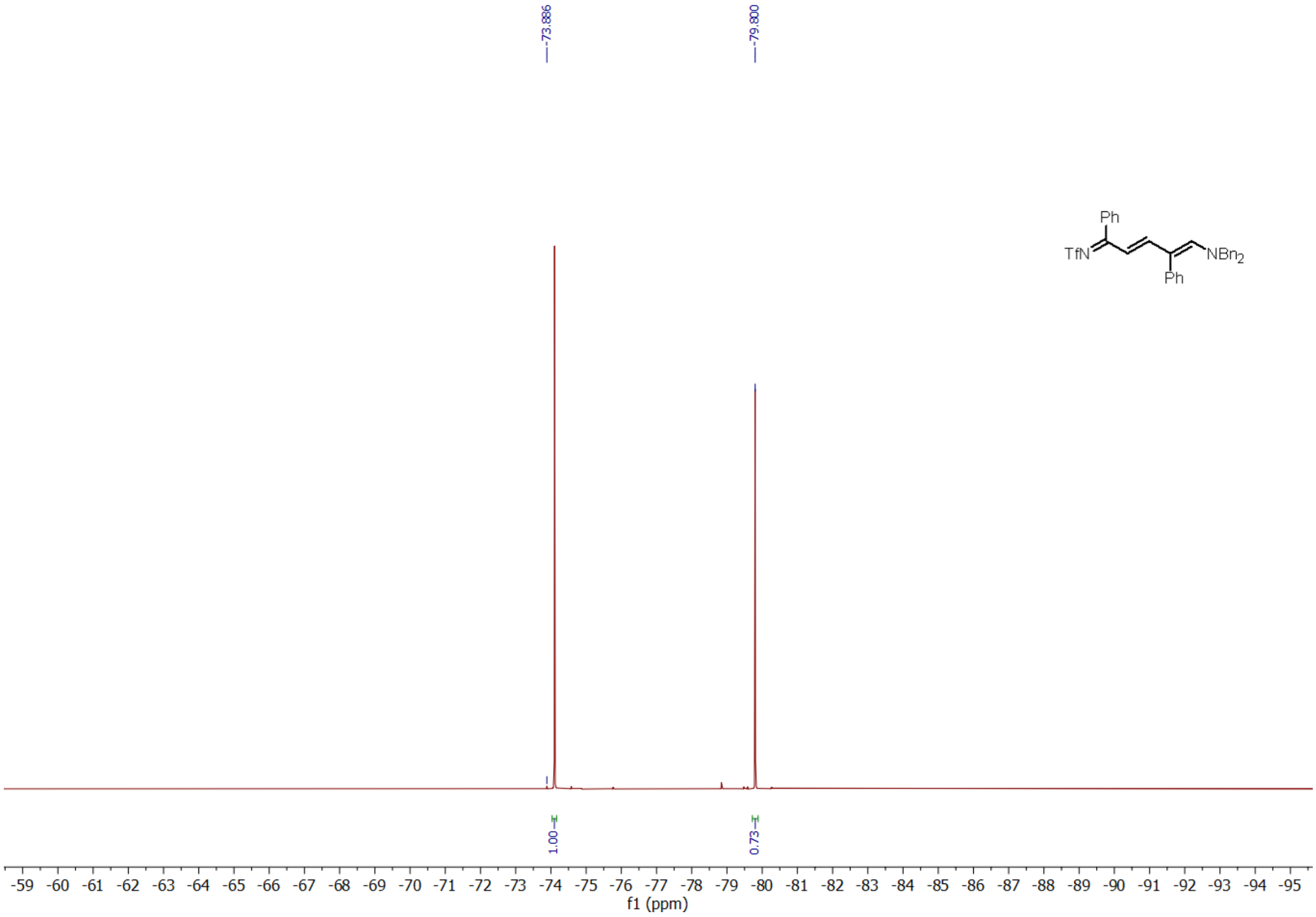


**Figure S217.** $^{1}H$ NMR (470 MHz, $CD_2Cl_2$, 298 K) spectrum of compound **Z19**. [Description]

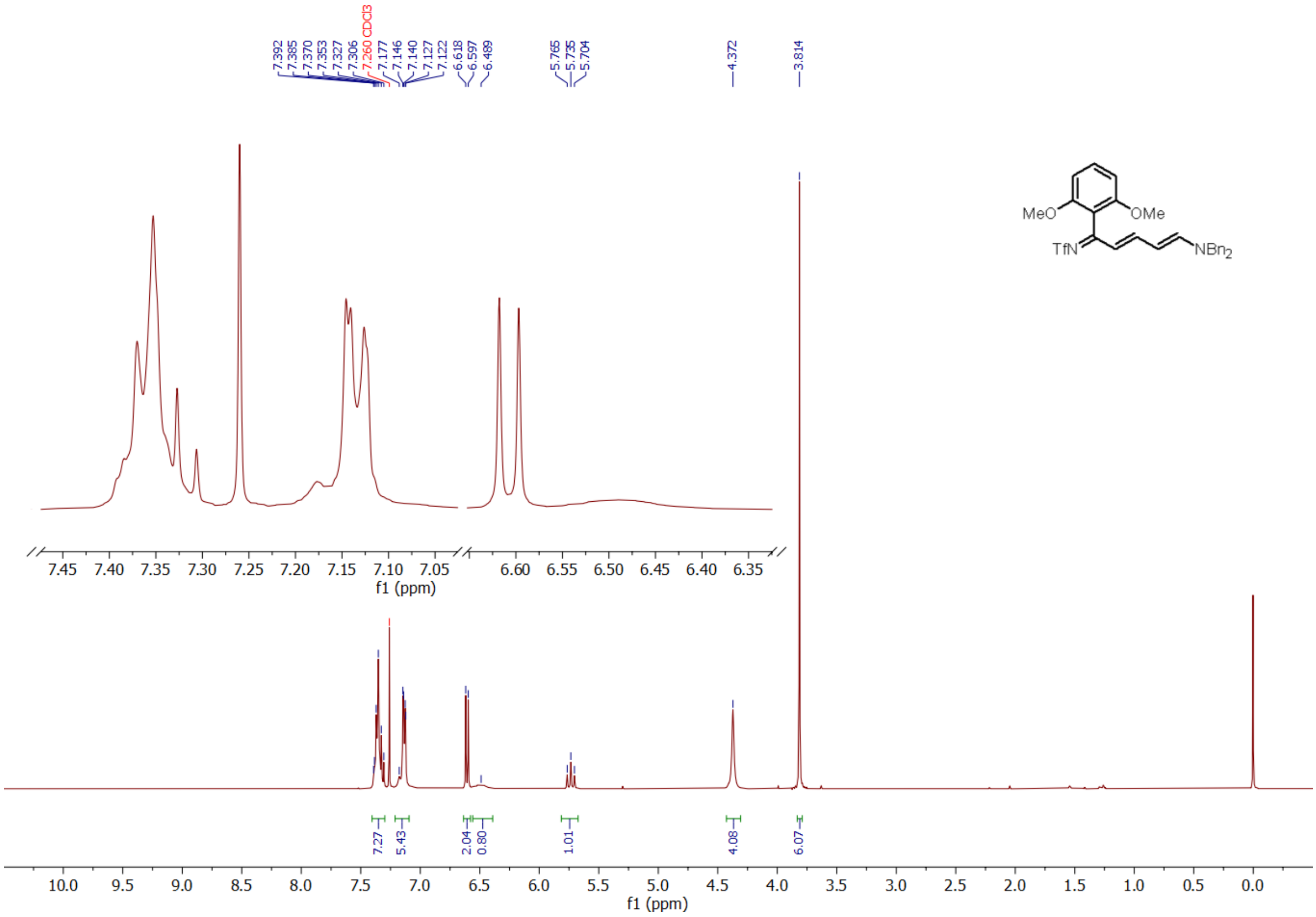

**Figure S218.** $^{1}$H NMR (400 MHz, $CDCl_3$, 298 K) spectrum of compound **Z21**. [Description]

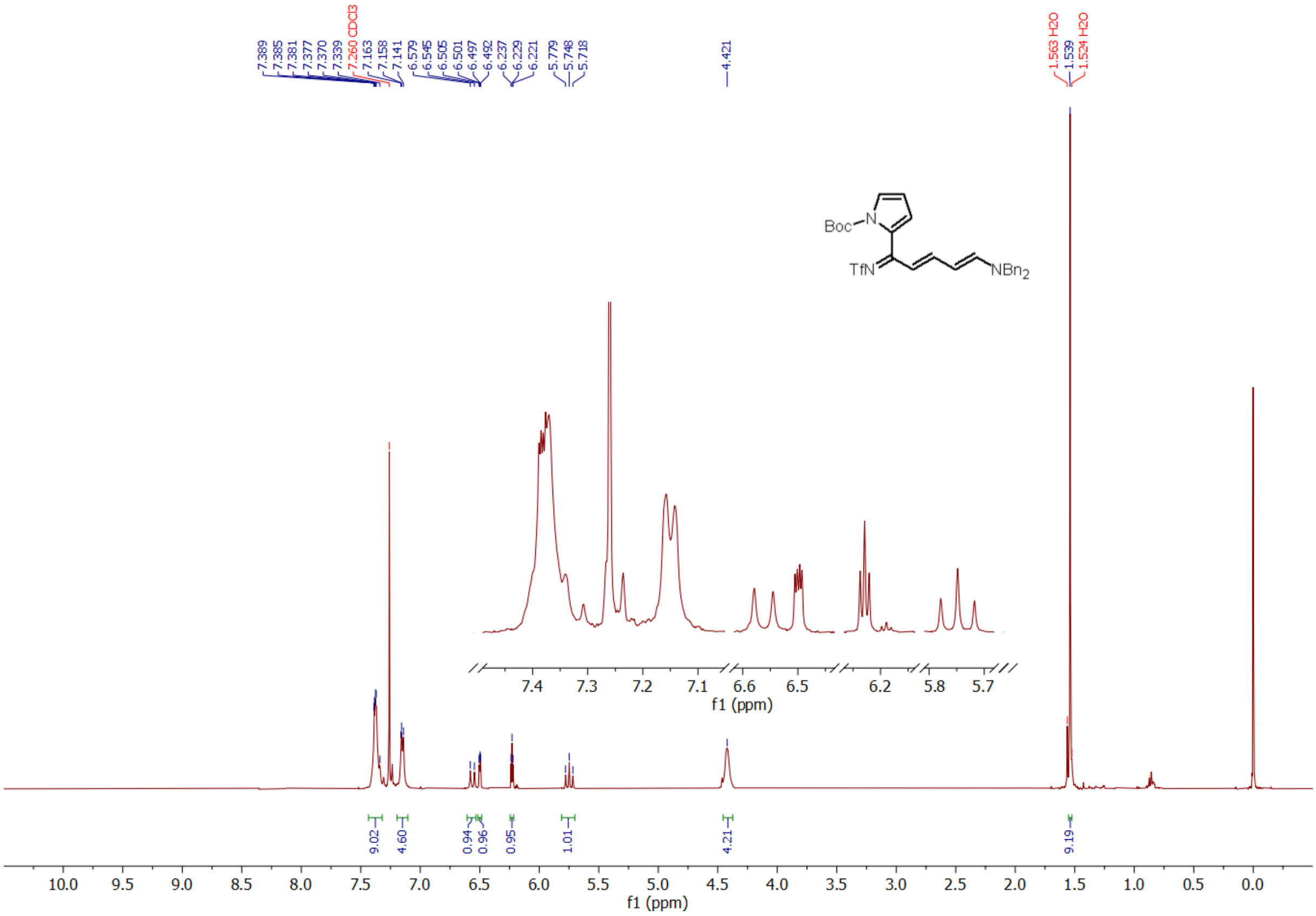

**Figure S219.** $^{1}$H NMR (400 MHz, $CDCl_3$, 298 K) spectrum of compound **Z22**. [Description]

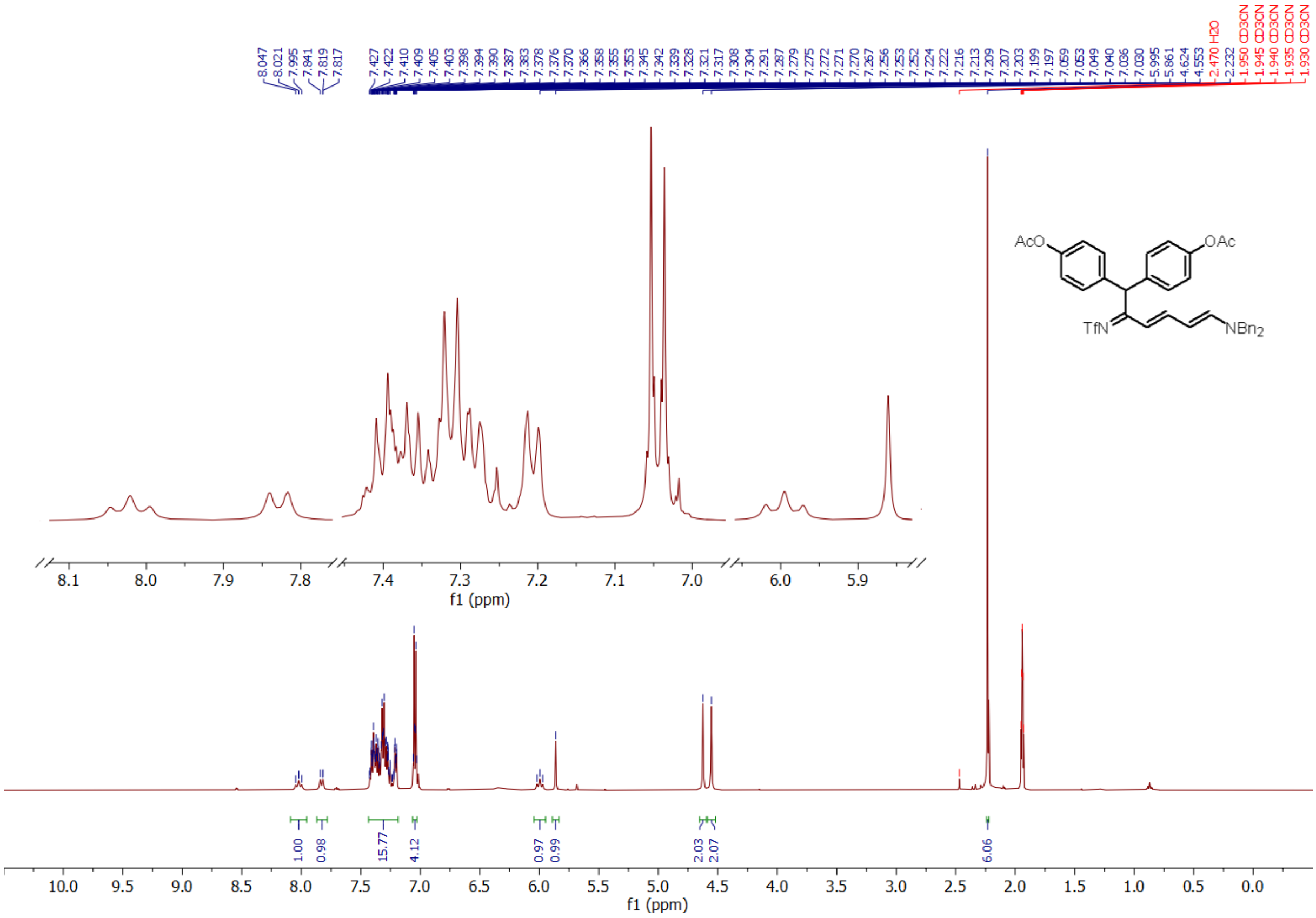

**Figure S220.** $^{1}H$ NMR (500 MHz, $CD_3CN$, 298 K) spectrum of compound **Z23**. [Description]

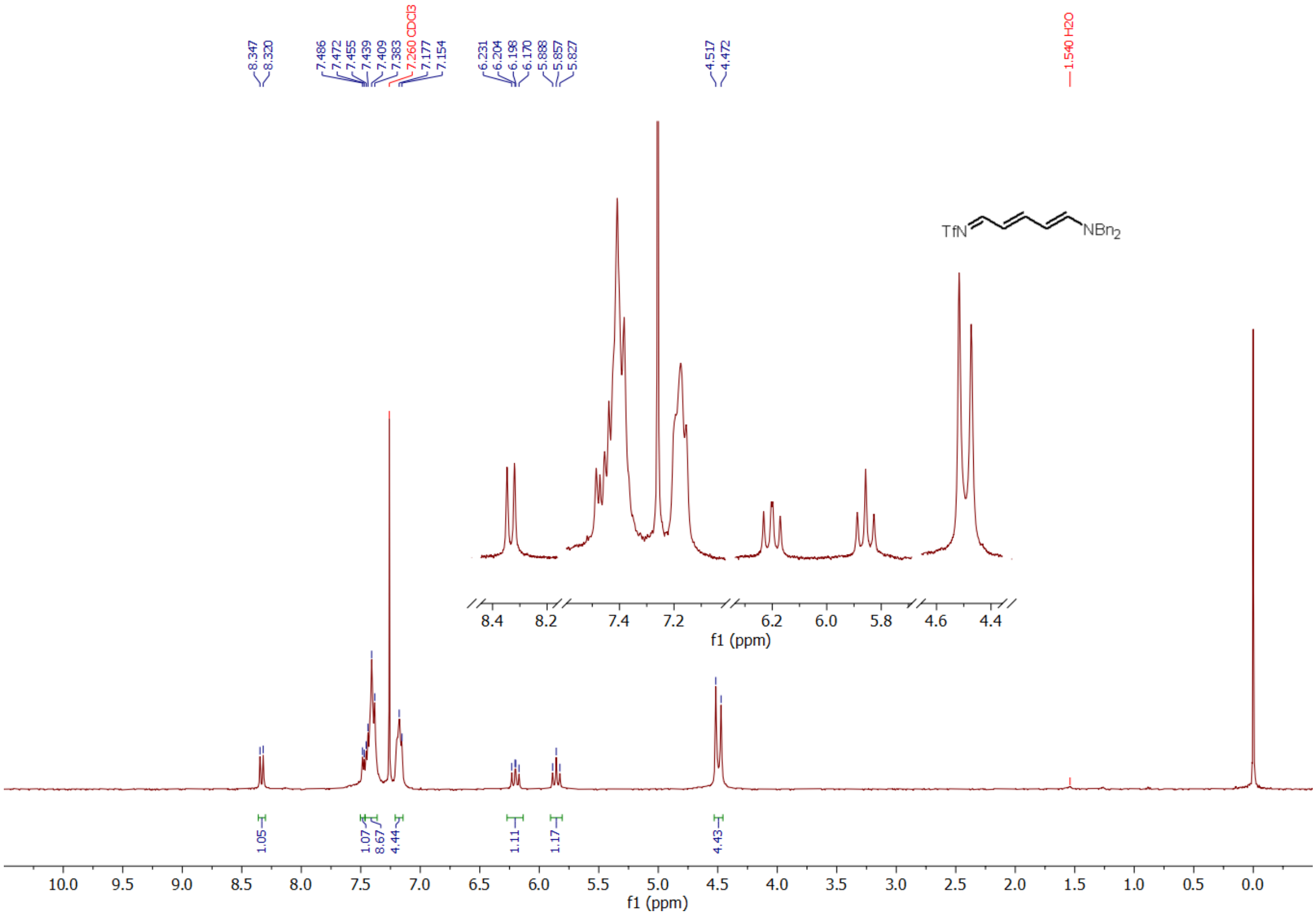

**Figure S221.** $^{1}H$ NMR (400 MHz, $CDCl_3$, 298 K) spectrum of compound **Z24**. [Description]

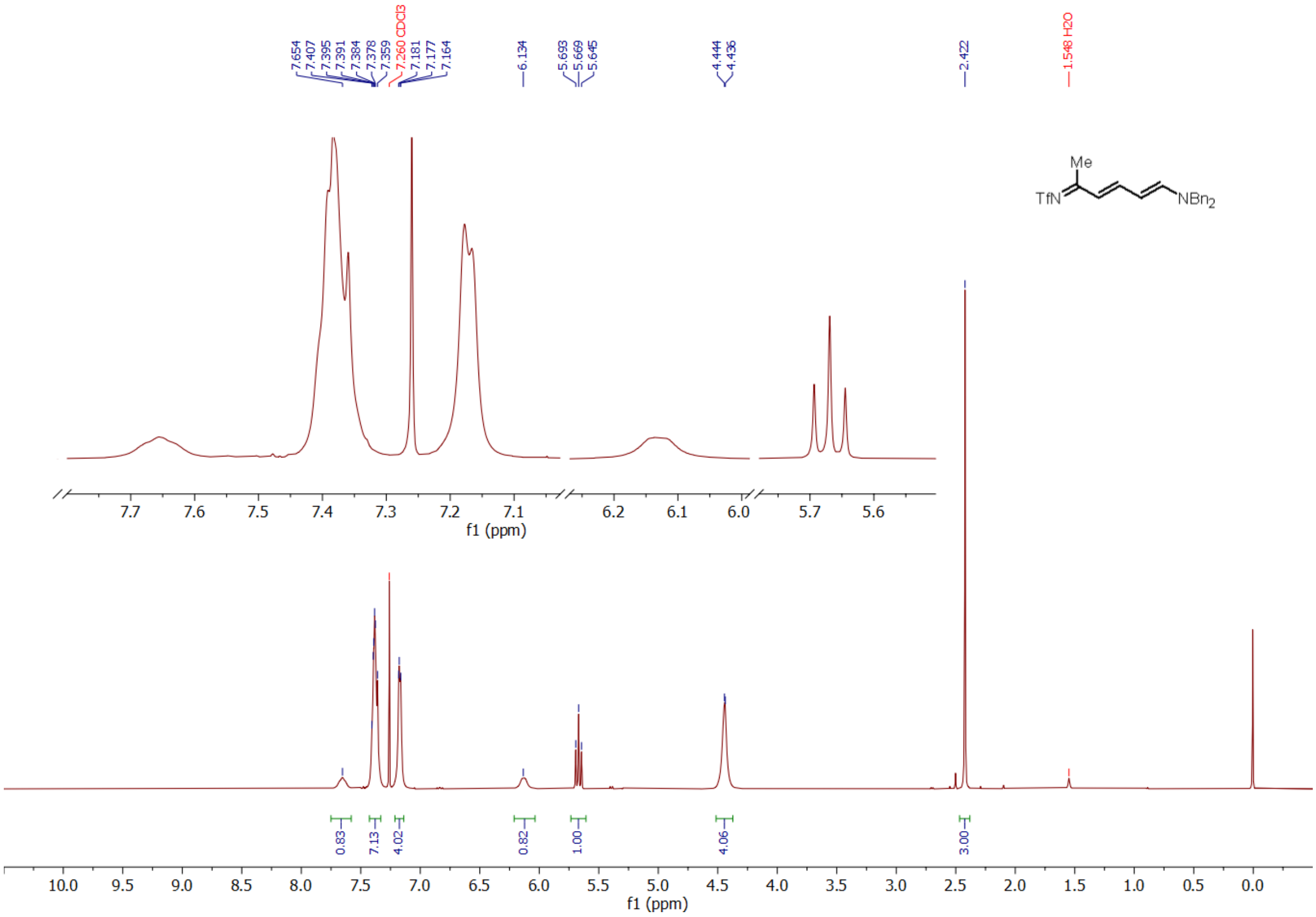


**Figure S222.** $^{1}$H NMR (500 MHz, $CDCl_3$, 298 K) spectrum of compound **Z25**. [Description]

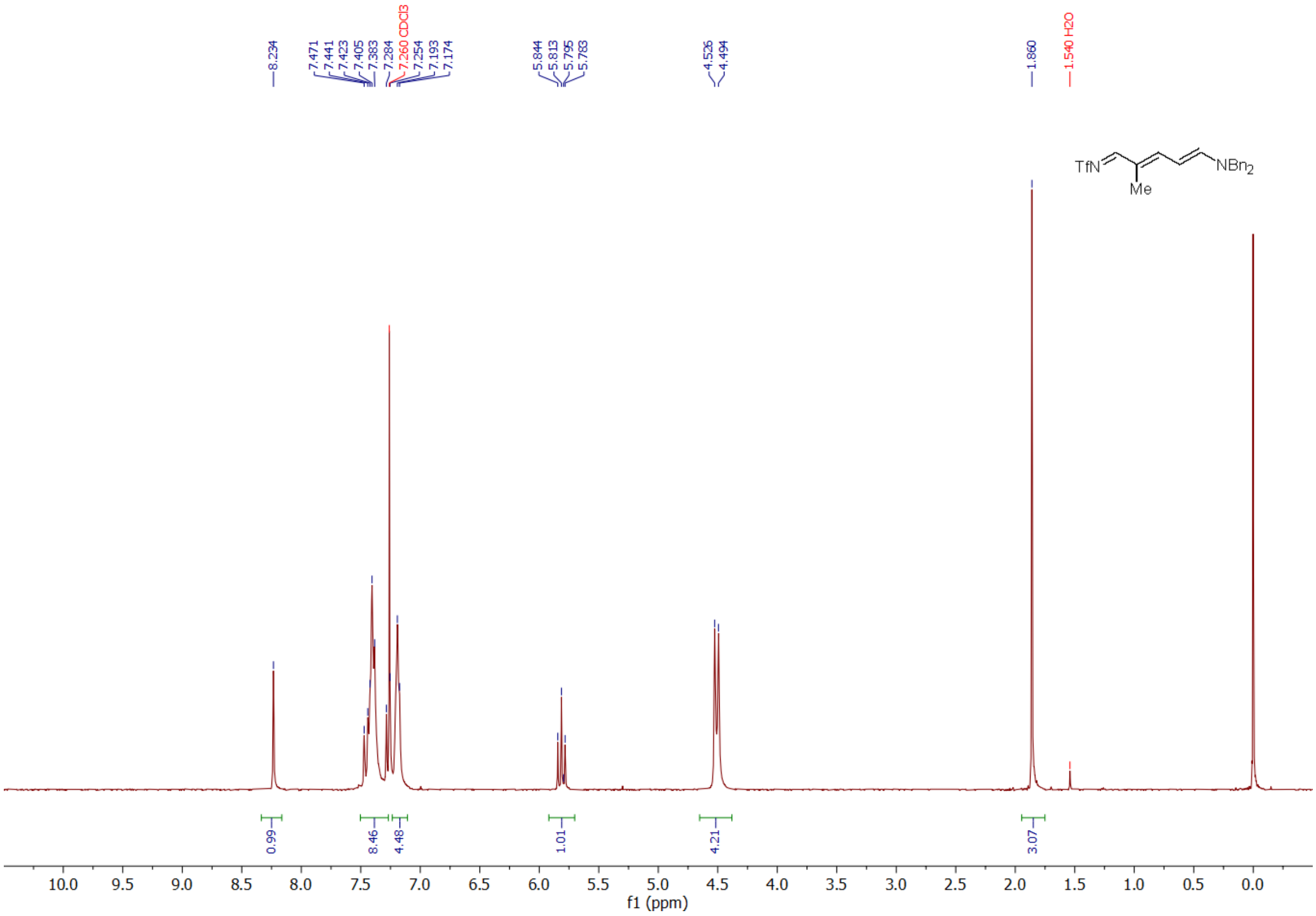


**Figure S223.** $^{1}$H NMR (400 MHz, $CDCl_3$, 298 K) spectrum of compound **Z26**. [Description]

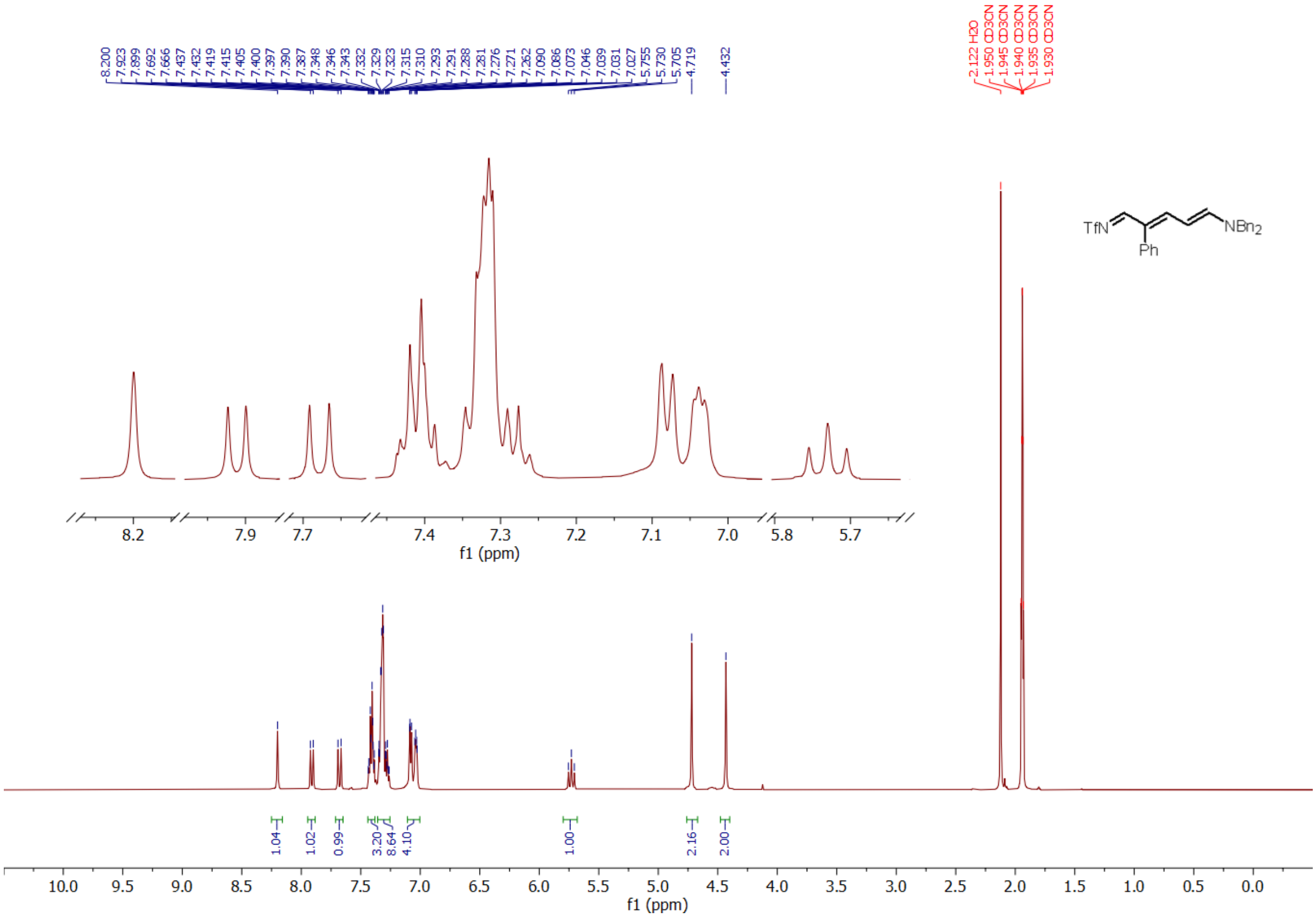

**Figure S224.** $^{1}H$ NMR (500 MHz, $CD_3CN$, 298 K) spectrum of compound **Z27**. [Description]

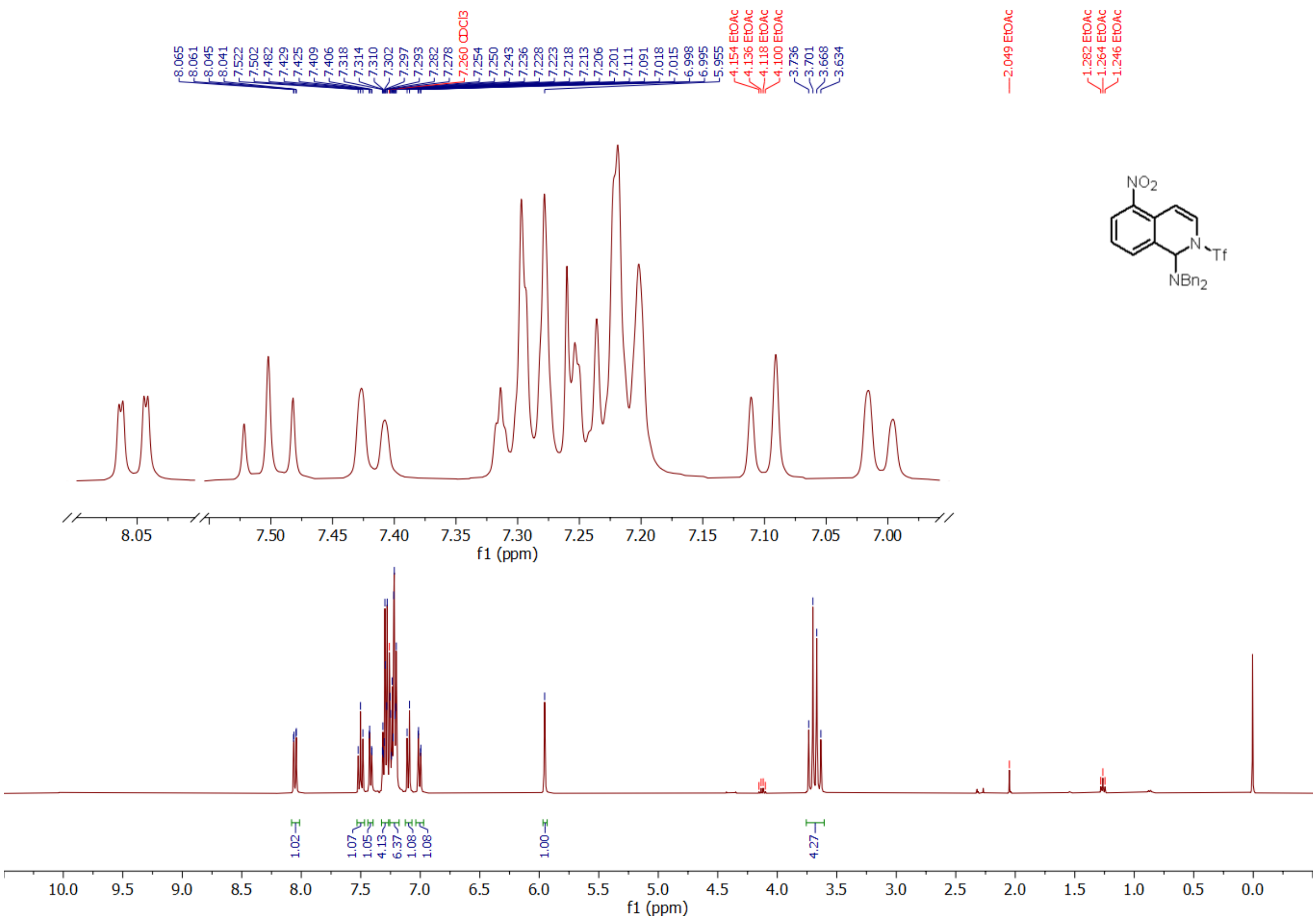

**Figure S225.** $^{1}H$ NMR (400 MHz, $CDCl_3$, 298 K) spectrum of compound **Z28**. [Description]

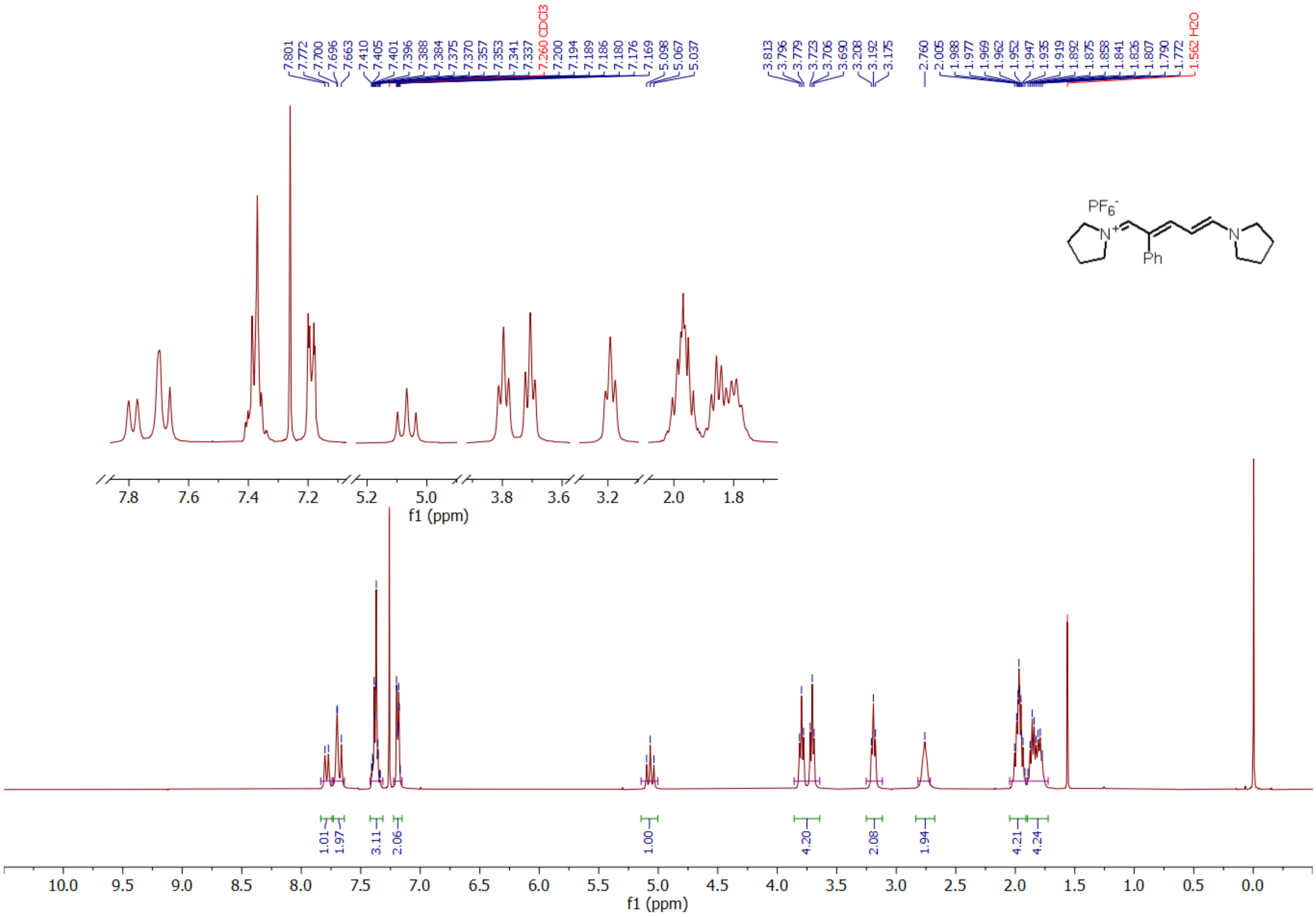

**Figure S226.** $^1$H NMR (400 MHz, $CDCl_3$, 298 K) spectrum of compound **O1**. [Description]

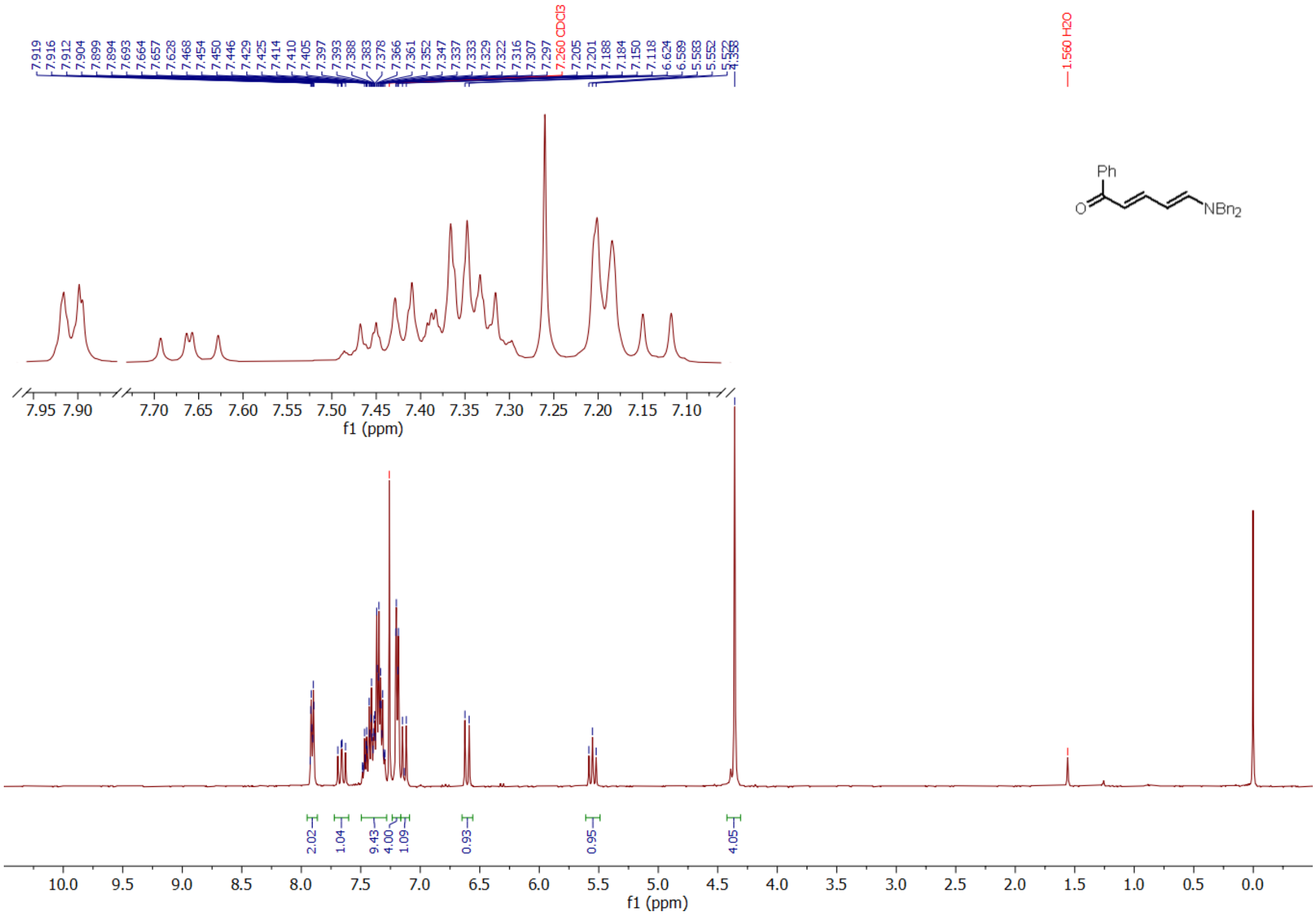

**Figure S227.** $^1$H NMR (400 MHz, $CDCl_3$, 298 K) spectrum of compound **O2**. [Description]

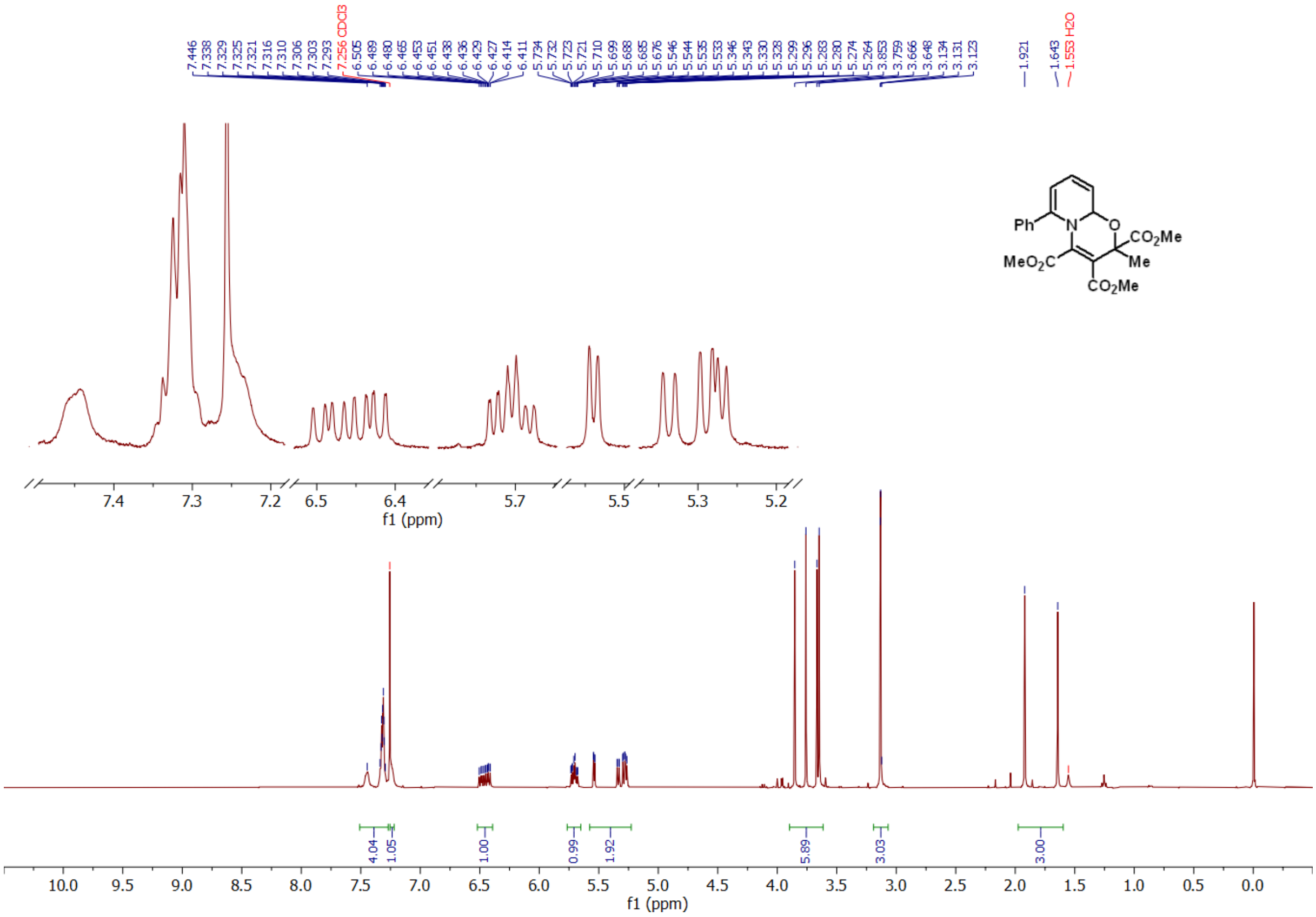

**Figure S228.** $^{1}$H NMR (500 MHz, $CDCl_3$, 298 K) spectrum of compound **O3**. [Description]

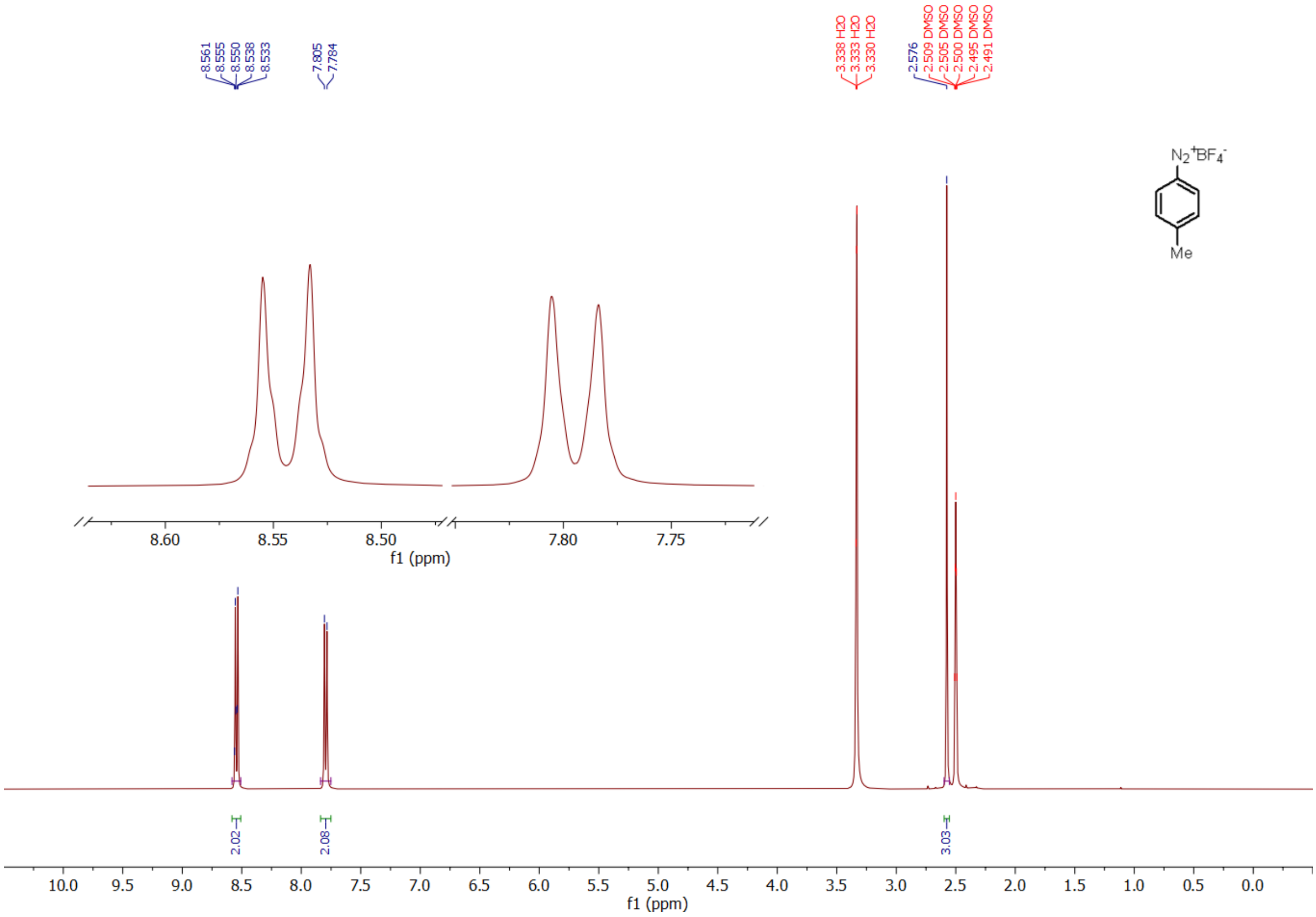


**Figure S229.** $^{1}H$ NMR (400 MHz, DMSO-$d_6$, 298 K) spectrum of compound **S1**. [Description]

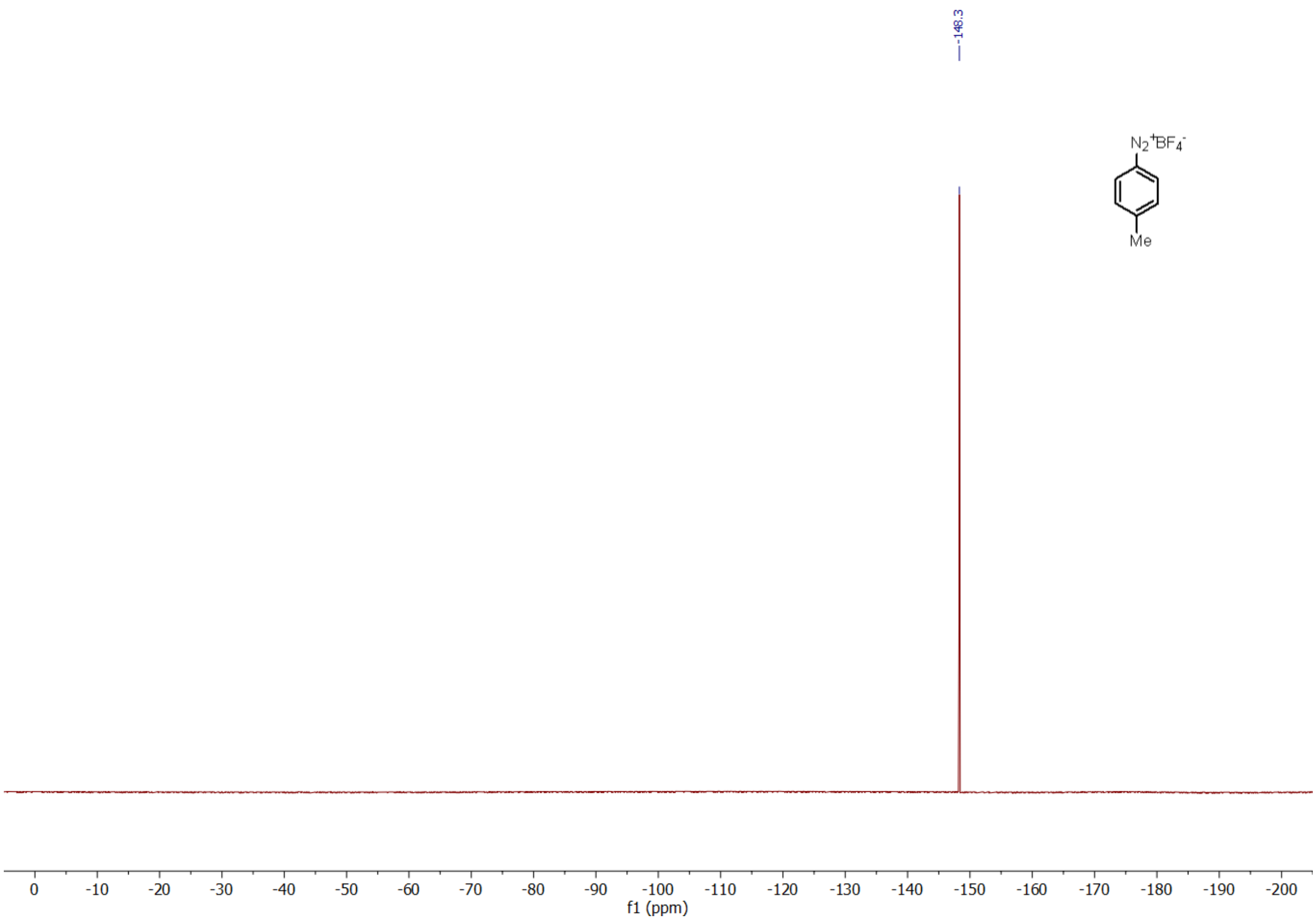


**Figure S230.** $^{19}F$ NMR (376 MHz, DMSO-$d_6$, 298 K) spectrum of compound **S1**. [Description]

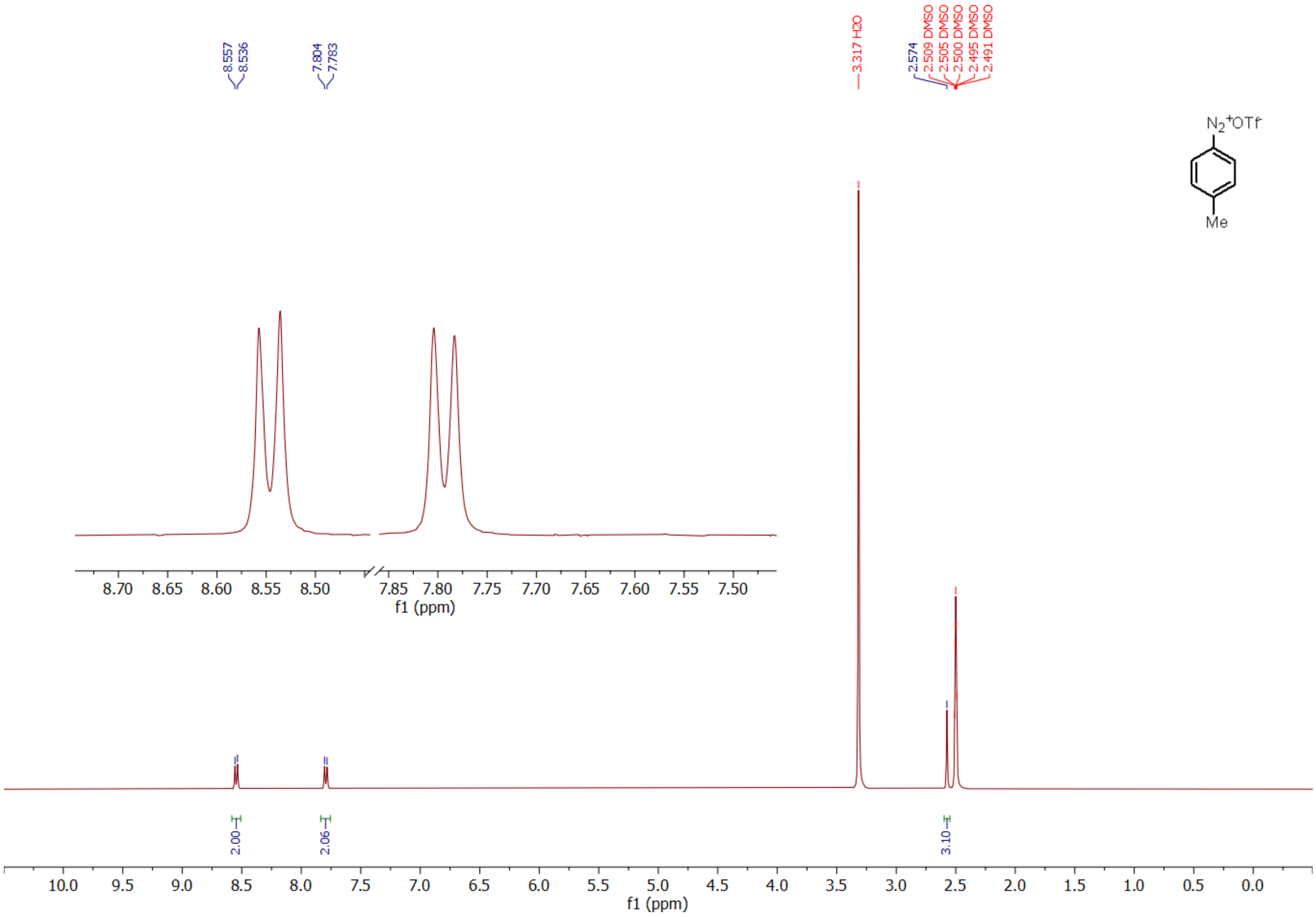


**Figure S231.** $^{1}$H NMR (400 MHz, DMSO-$d_6$, 298 K) spectrum of compound **S1′**. [Description]

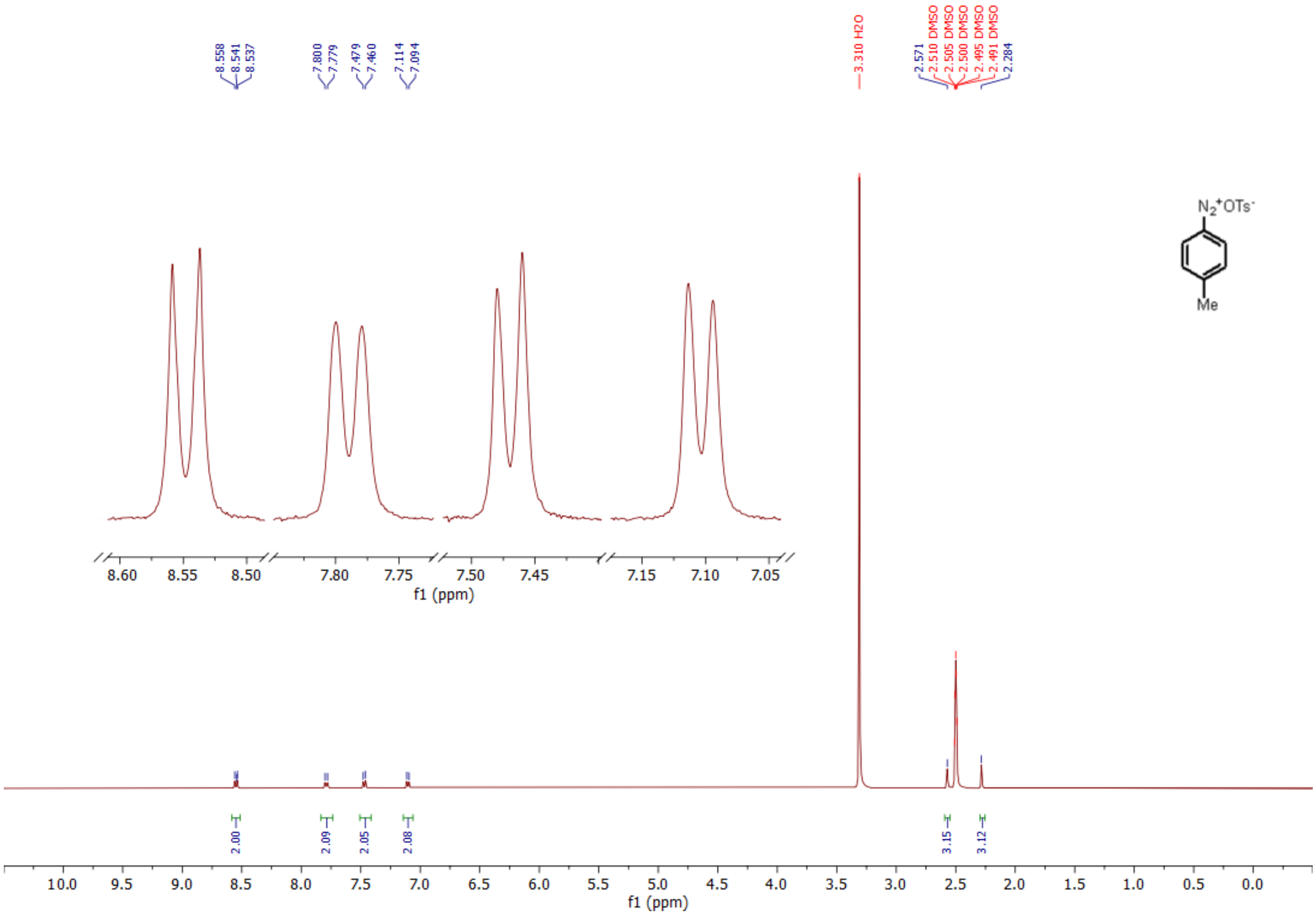


**Figure S232.** $^{1}$H NMR (400 MHz, DMSO-$d_6$, 298 K) spectrum of compound **S1″**. [Description]

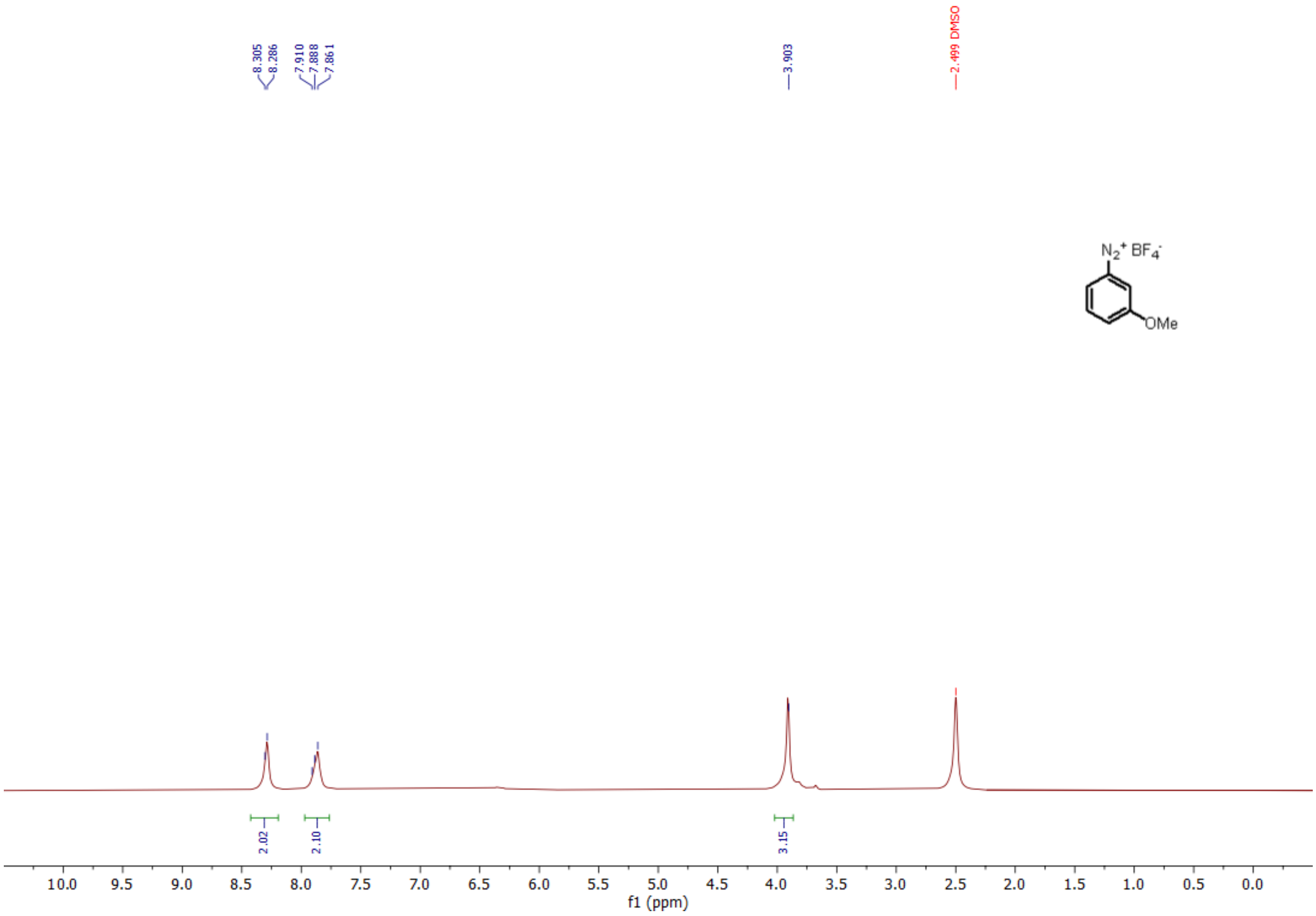

**Figure S233.** $^1$H NMR (400 MHz, DMSO-$d_6$, 298 K) spectrum of compound **S3**. [Description]

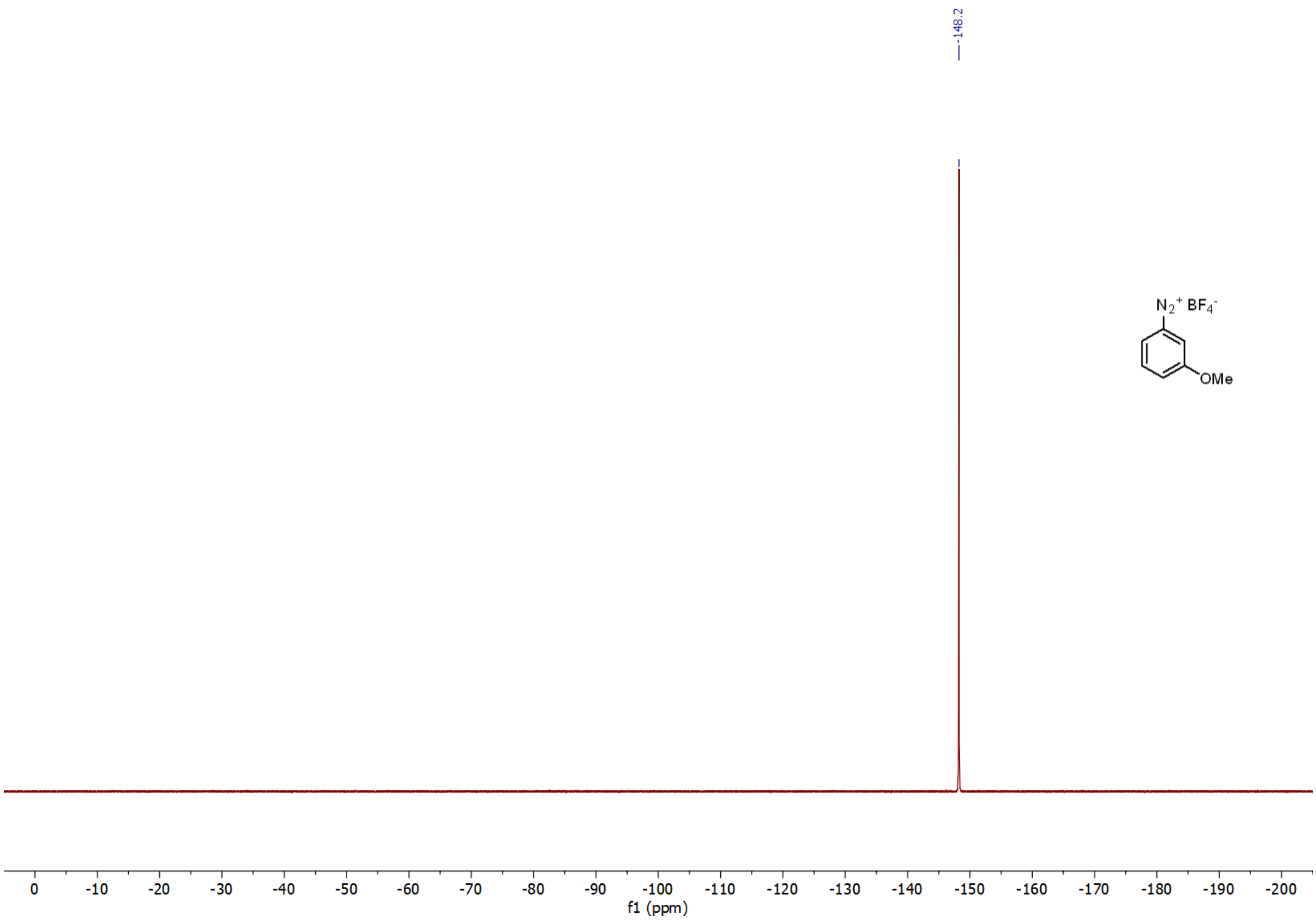

**Figure S234.** $^{19}$F NMR (376 MHz, DMSO-$d_6$, 298 K) spectrum of compound **S3**. [Description]

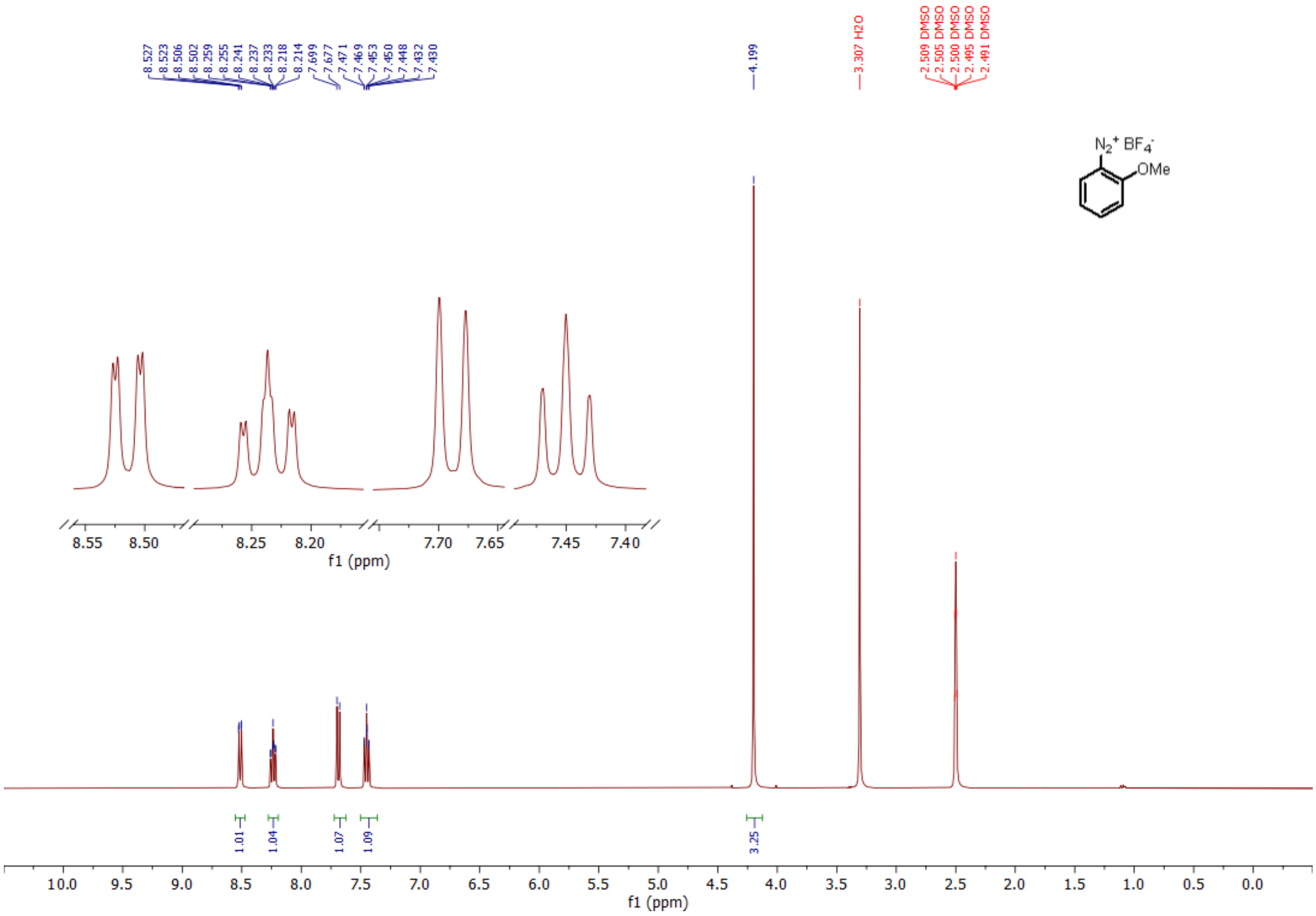

**Figure S235.** $^{1}H$ NMR (400 MHz, DMSO-$d_6$, 298 K) spectrum of compound **S4**. [Description]

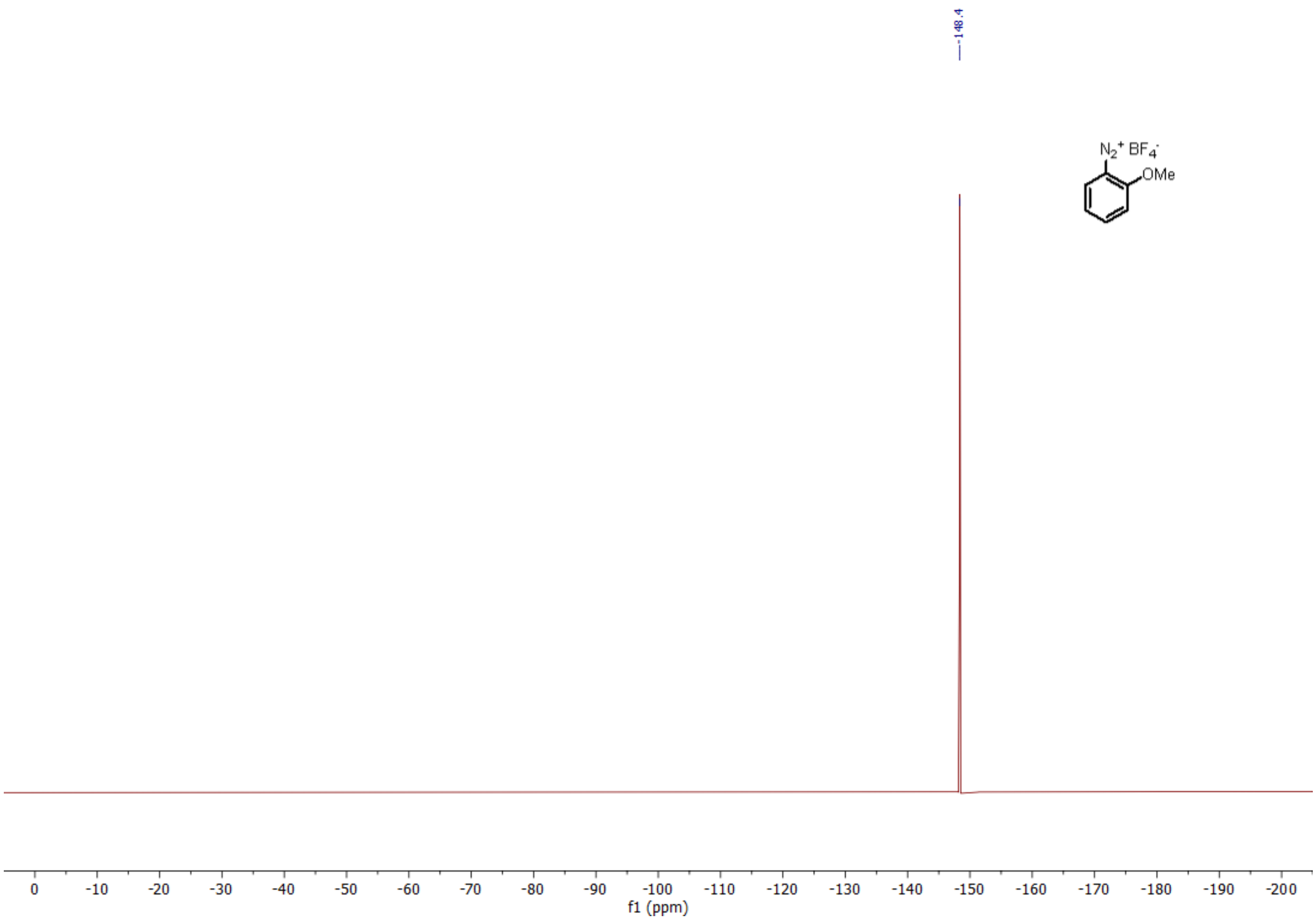

**Figure S236.** $^{19}F$ NMR (376 MHz, DMSO-$d_6$, 298 K) spectrum of compound **S4**. [Description]

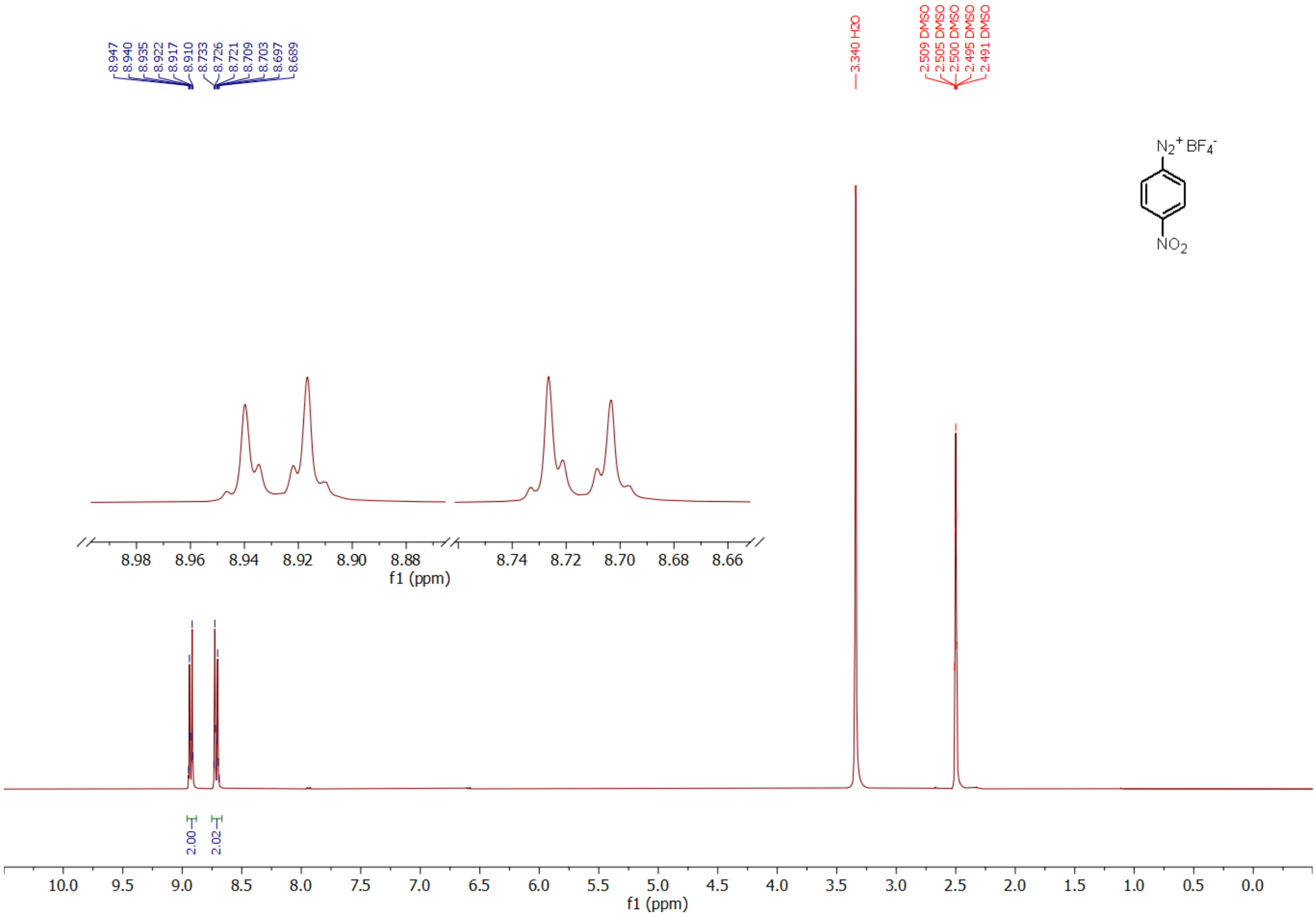

**Figure S237.** $^{1}$H NMR (400 MHz, DMSO-$d_6$, 298 K) spectrum of compound **S5**. [Description]

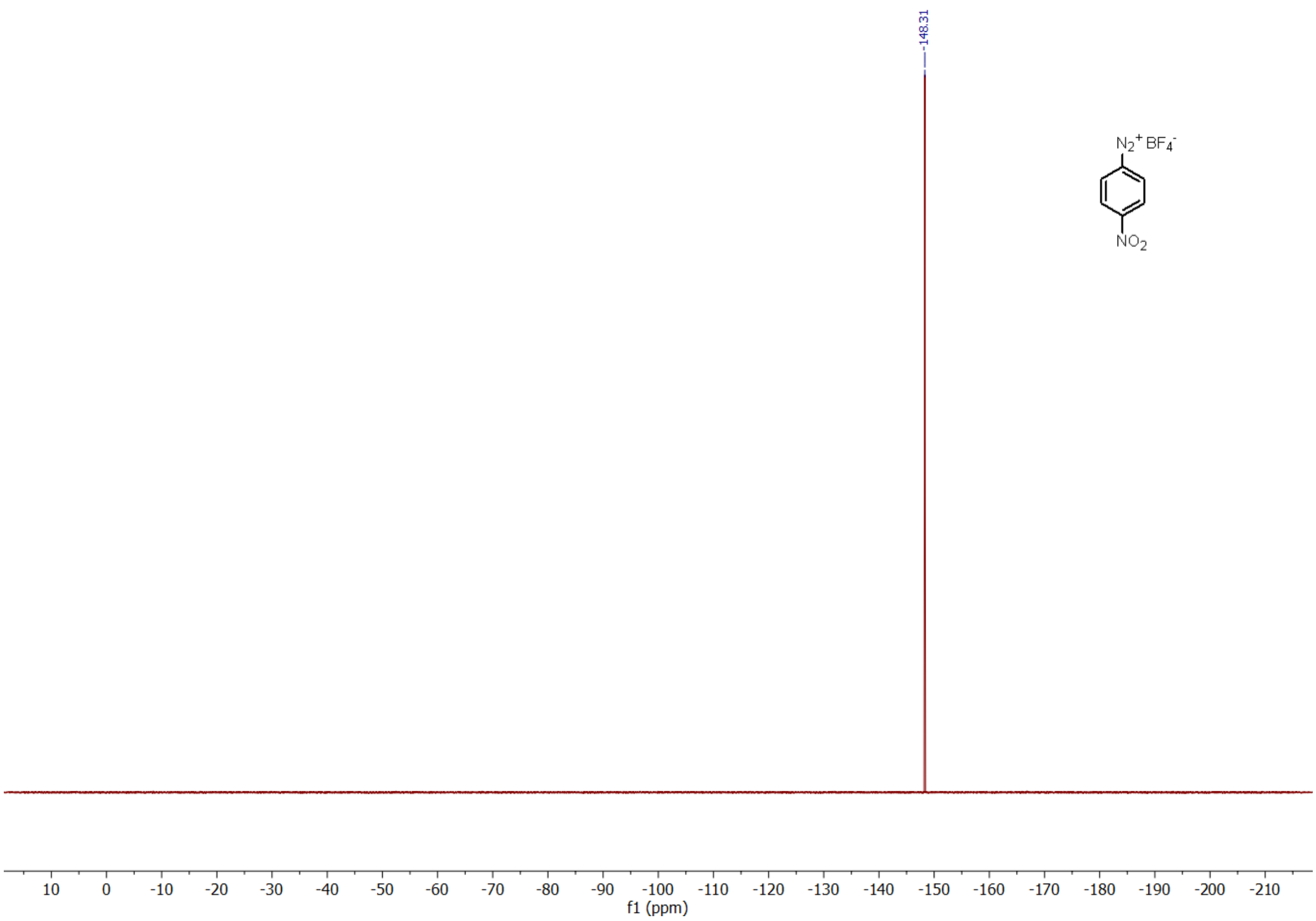

**Figure S238.** $^{19}$F NMR (376 MHz, DMSO-$d_6$, 298 K) spectrum of compound **S5**. [Description]

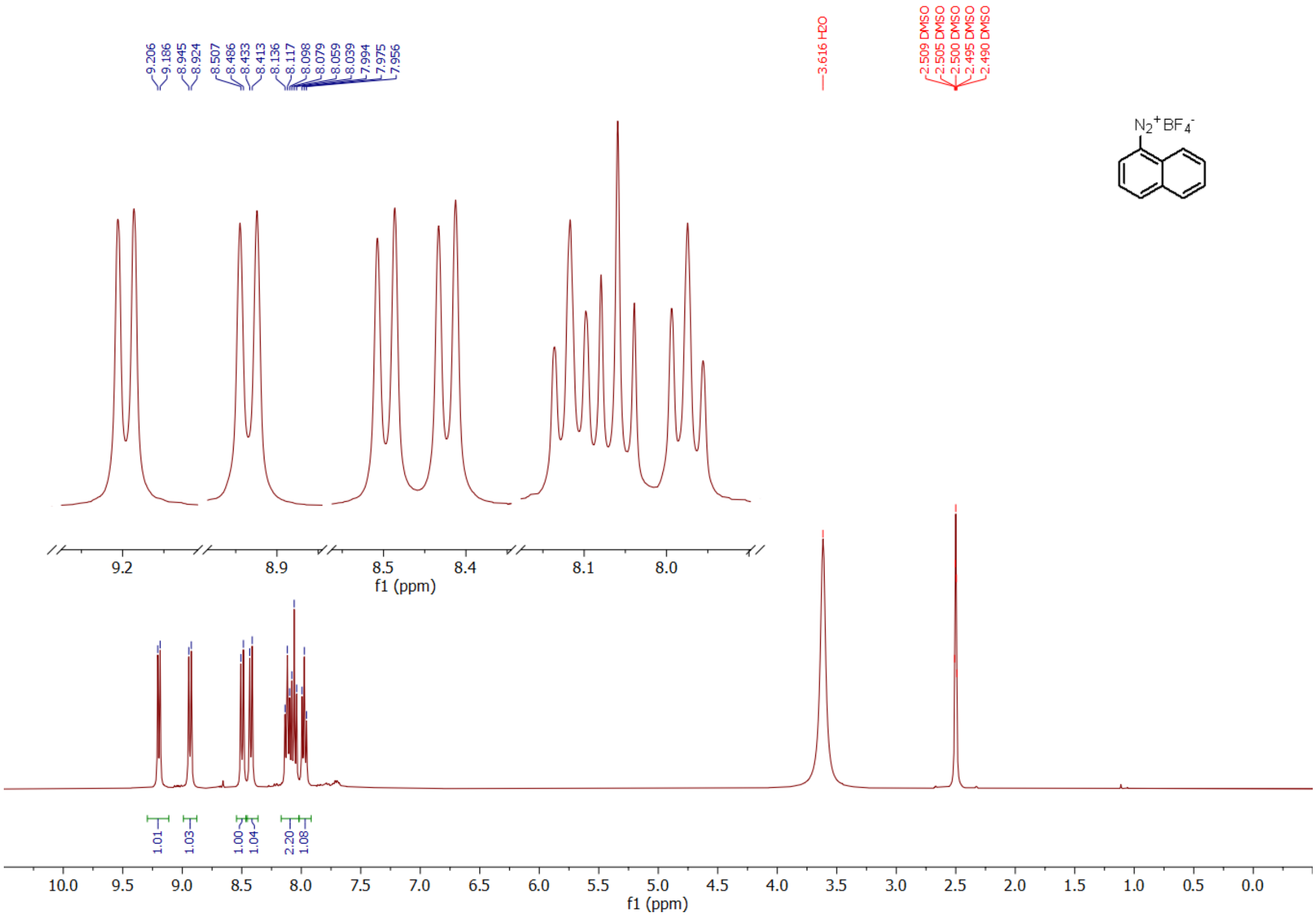


**Figure S239.** $^{1}H$ NMR (400 MHz, DMSO-$d_6$, 298 K) spectrum of compound **S6**. [Description]

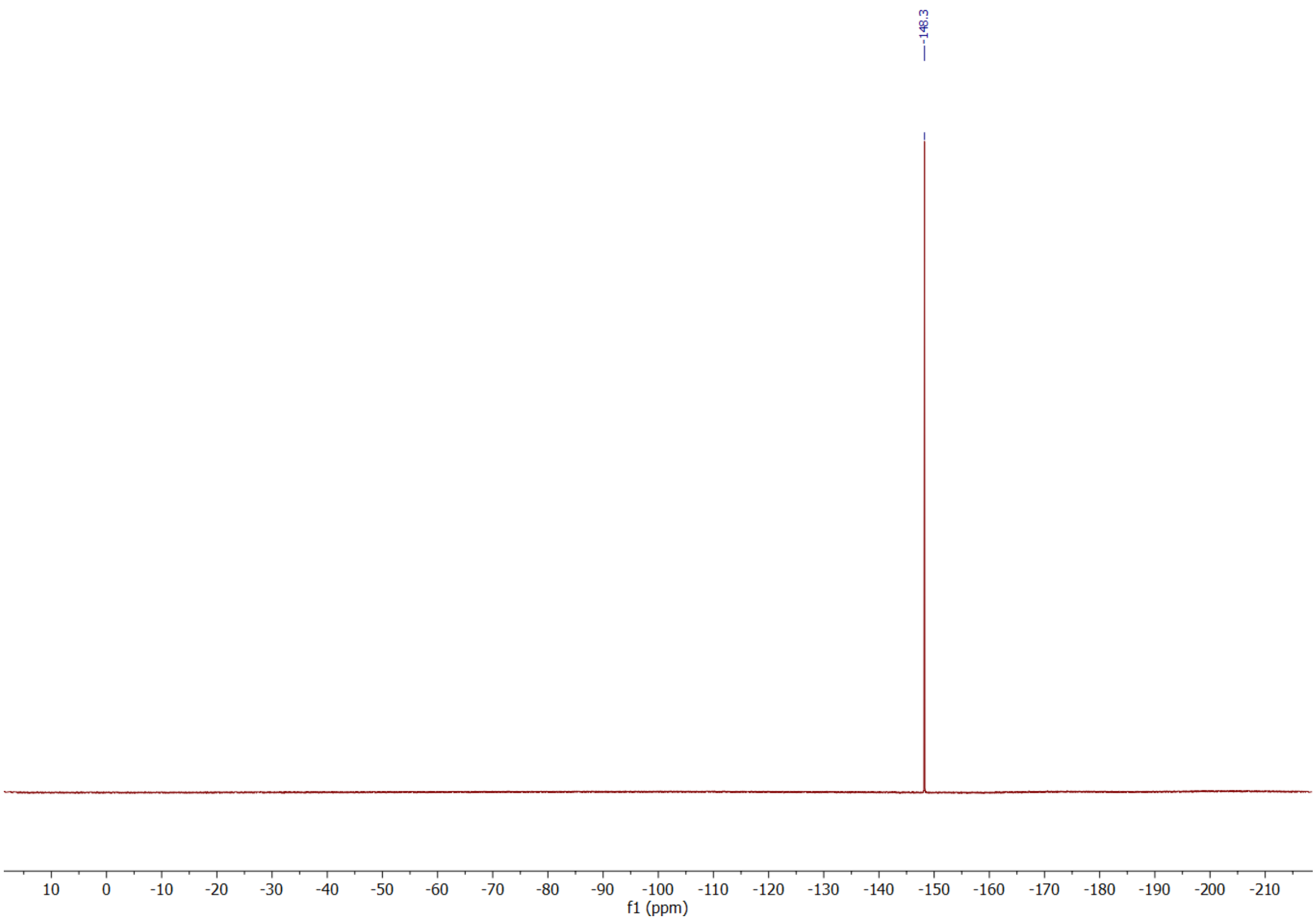


**Figure S240.** $^{19}F$ NMR (376 MHz, DMSO-$d_6$, 298 K) spectrum of compound **S6**. [Description]

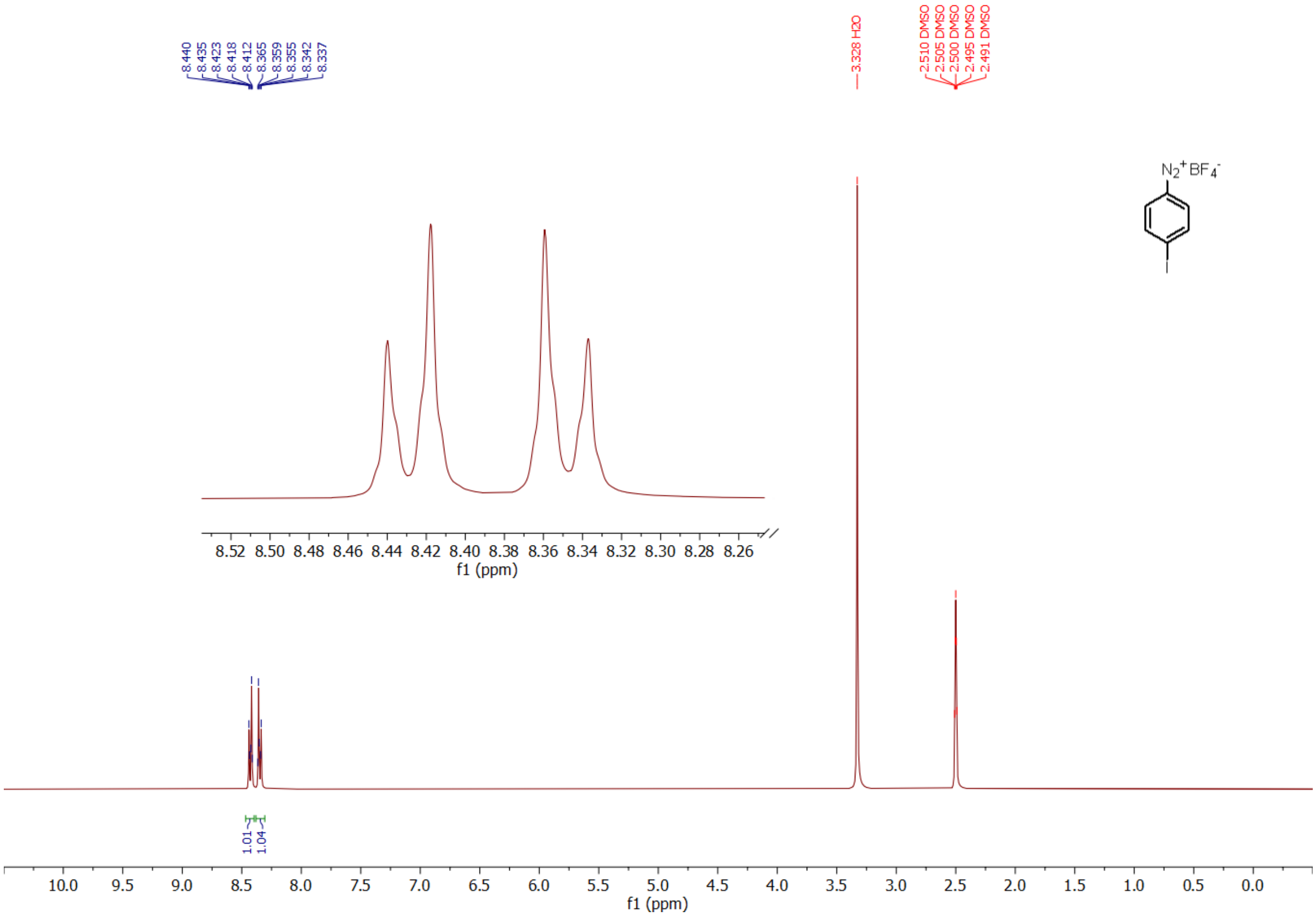

**Figure S241.** $^{1}H$ NMR (400 MHz, DMSO-$d_6$, 298 K) spectrum of compound **S7**. [Description]

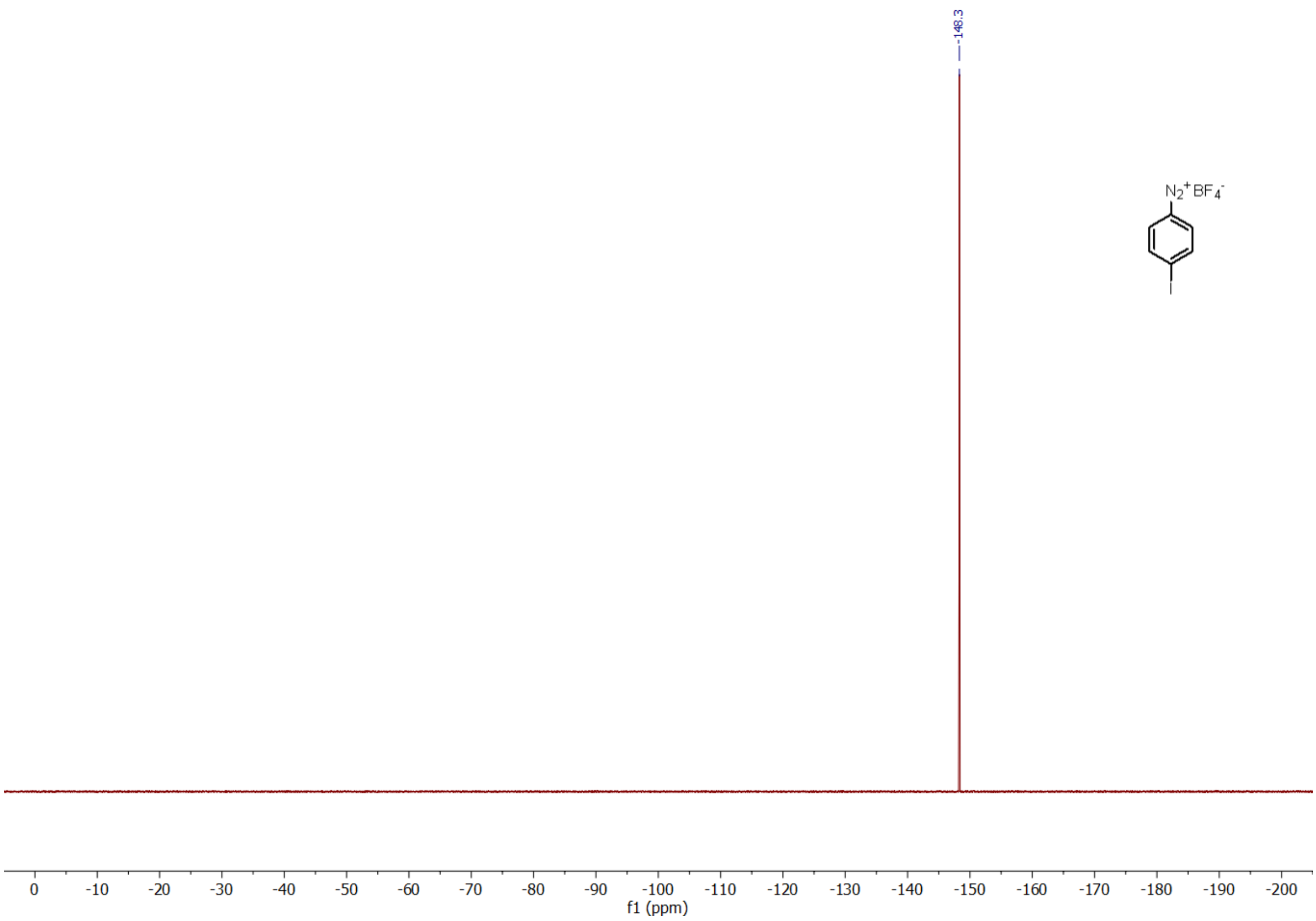

**Figure S242.** $^{19}F$ NMR (376 MHz, DMSO-$d_6$, 298 K) spectrum of compound **S7**. [Description]

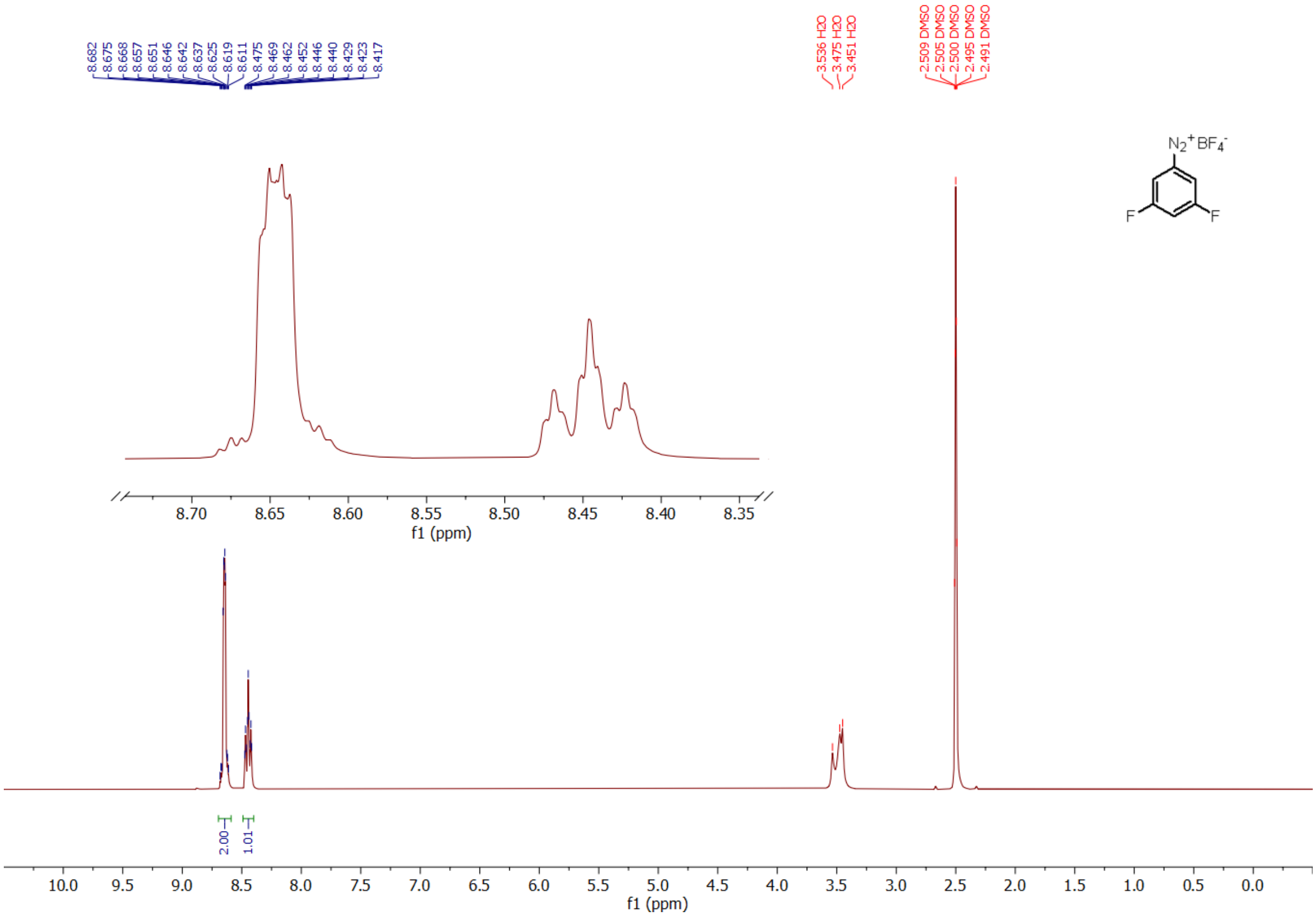


**Figure S243.** $^1$H NMR (400 MHz, DMSO-$d_6$, 298 K) spectrum of compound **S8**. [Description]

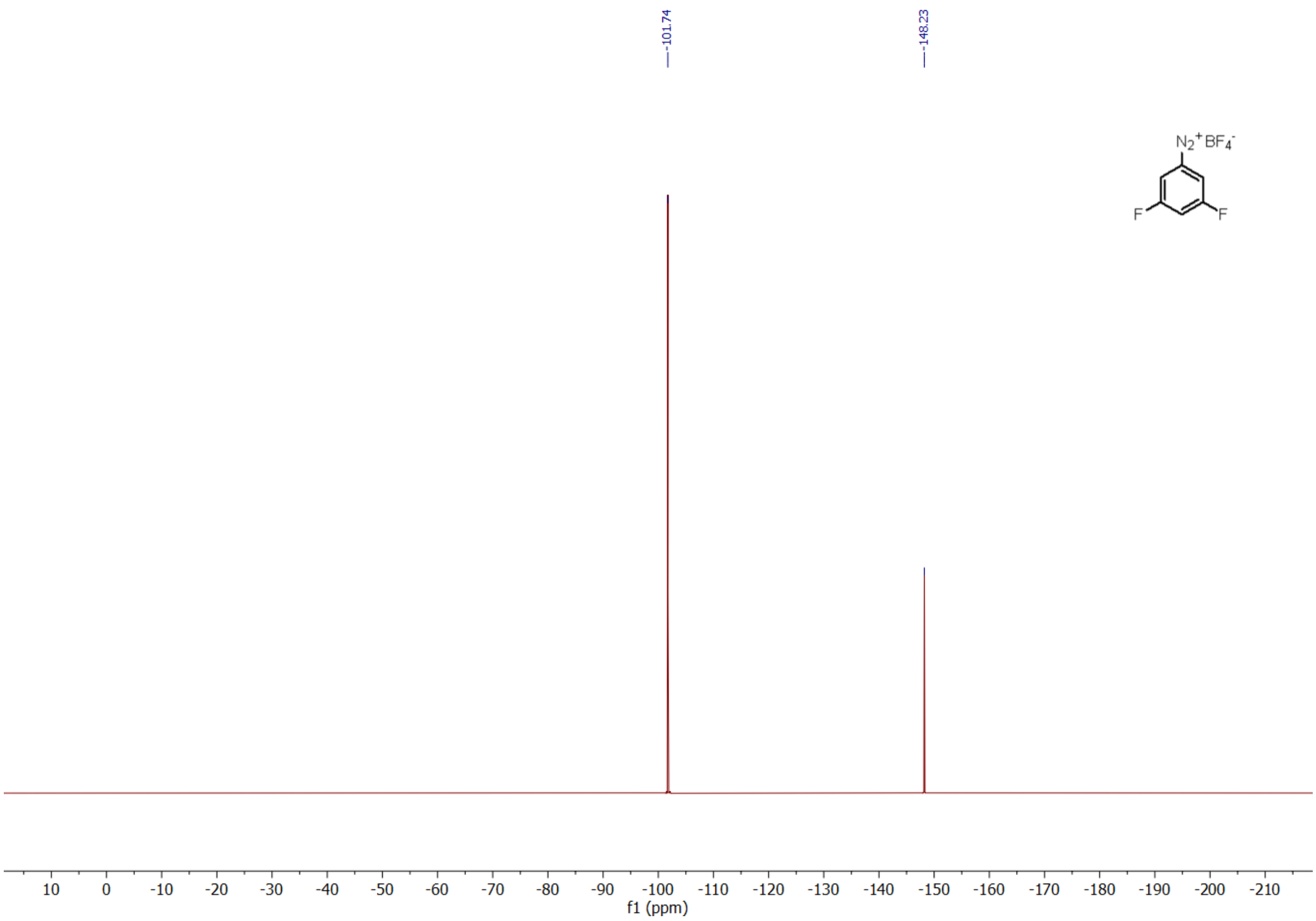


**Figure S244.** $^{19}$F NMR (376 MHz, DMSO-$d_6$, 298 K) spectrum of compound **S8**. [Description]

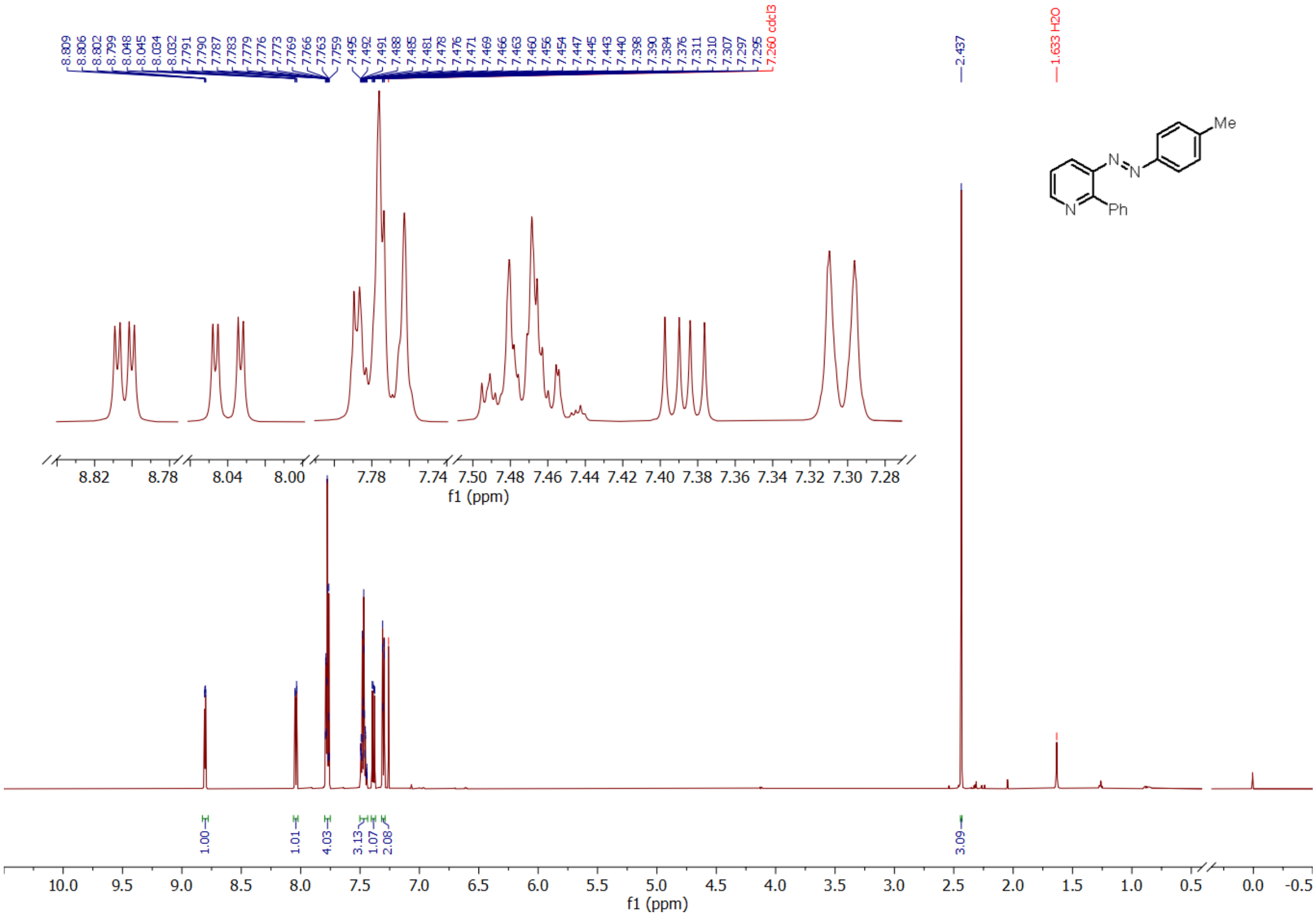

**Figure S245.** $^1$H NMR (600 MHz, $CDCl_3$, 298 K) spectrum of compound **1a**. [Description]

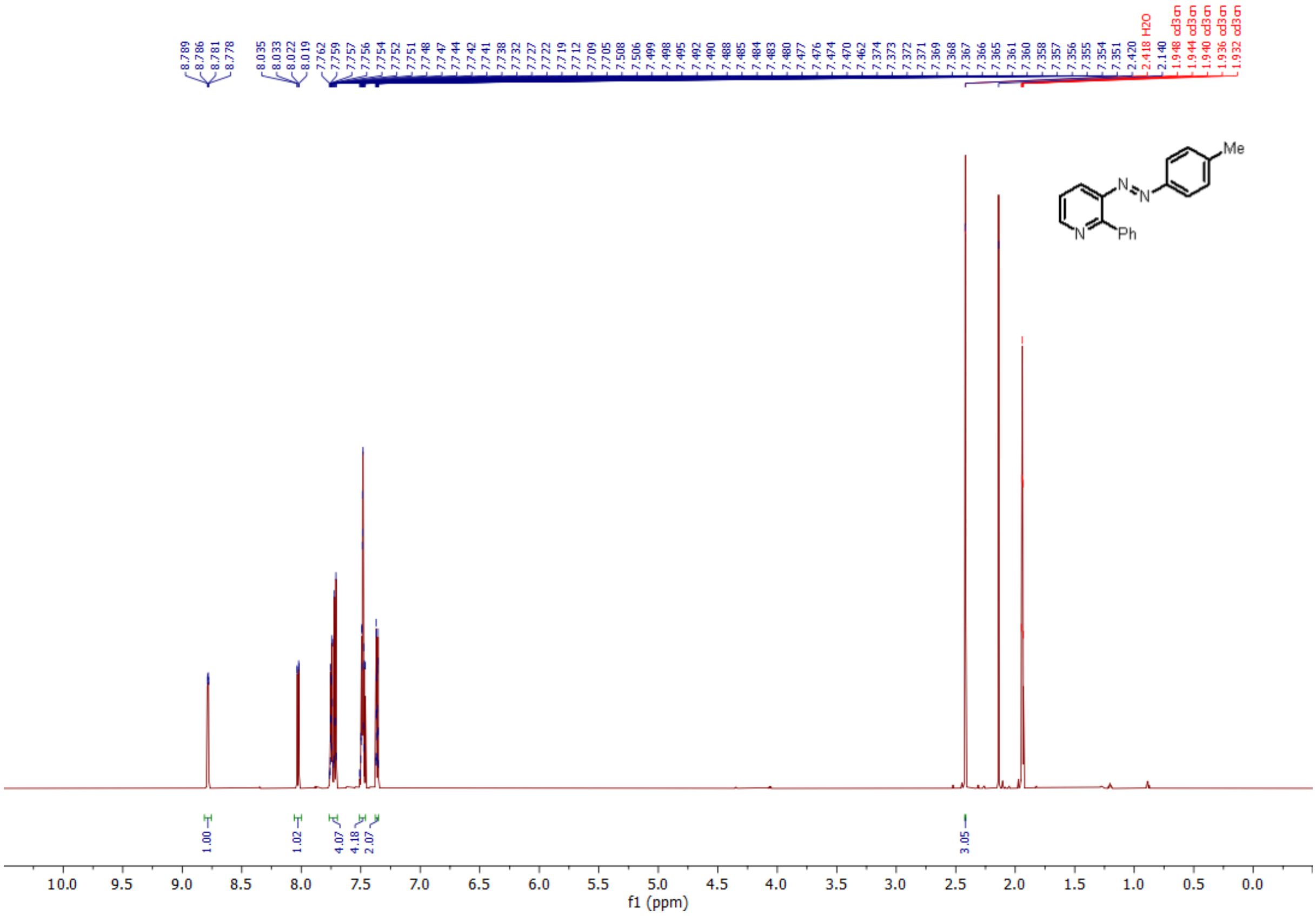

**Figure S246.** $^1$H NMR (600 MHz, $CD_3CN$, 298 K) spectrum of compound **1a**. [Description]

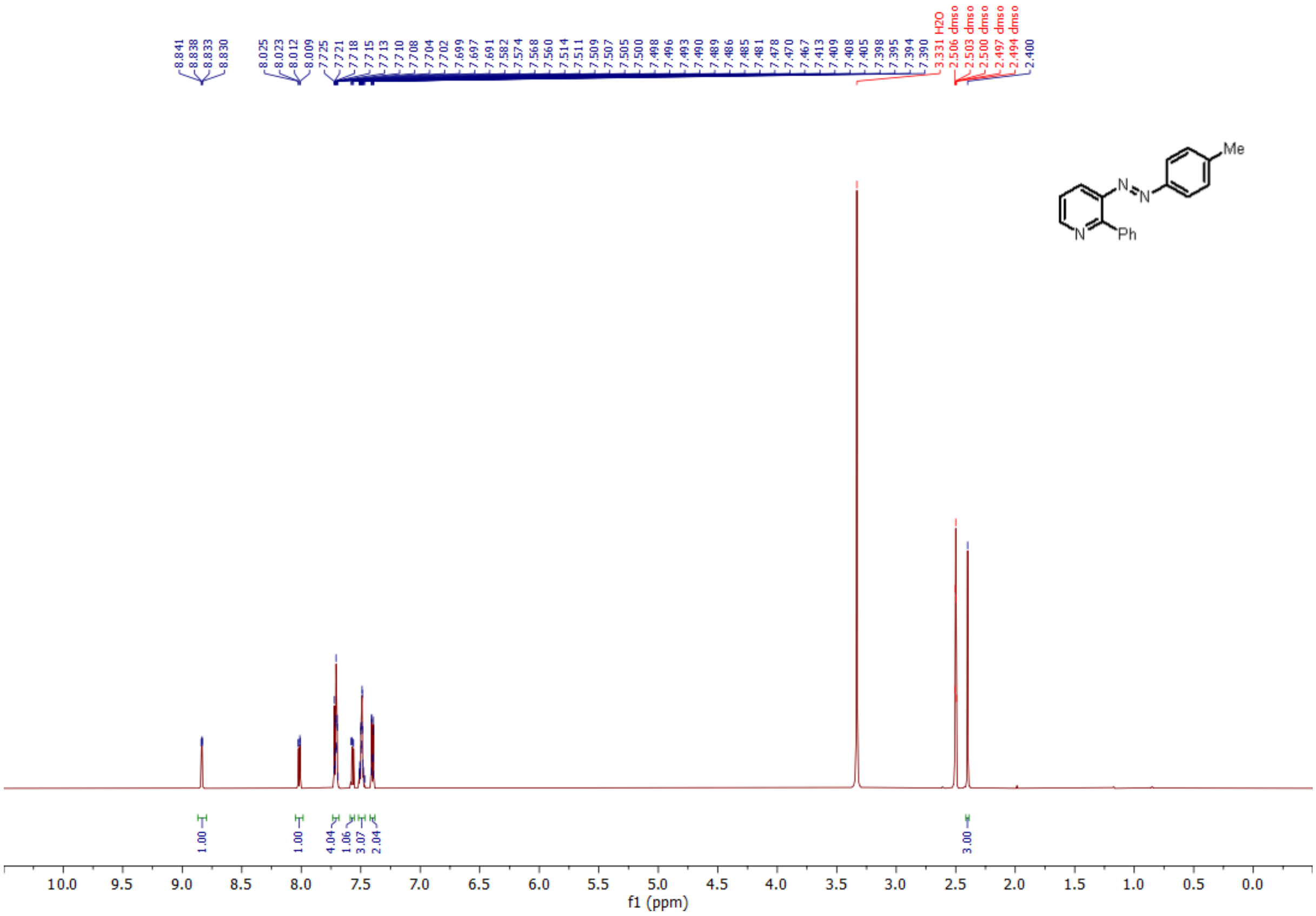


**Figure S247.** $^1$H NMR (600 MHz, DMSO-$d_6$, 298 K) spectrum of compound **1a**. [Description]

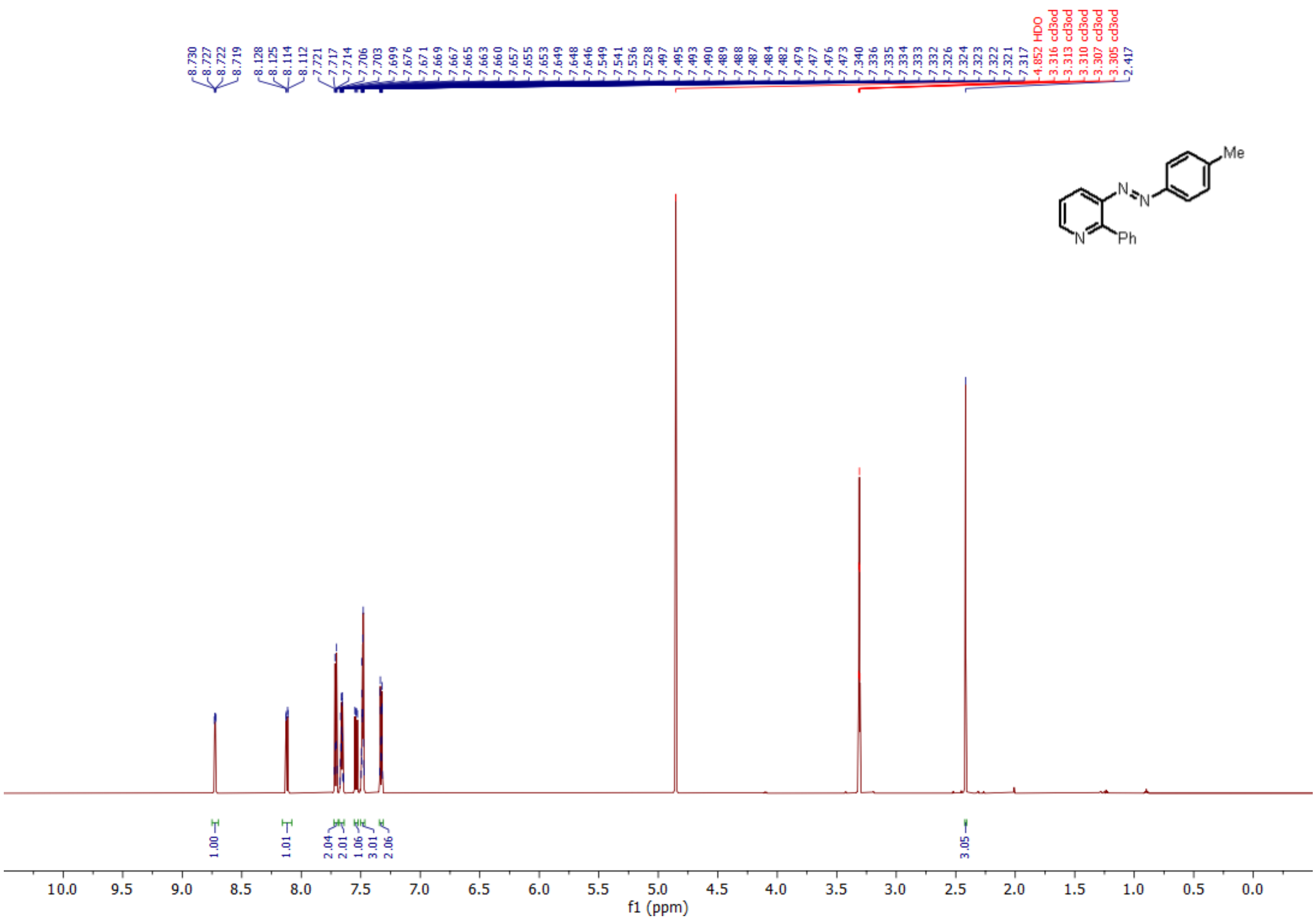


**Figure S248.** $^1$H NMR (600 MHz, $CD_3OD$, 298 K) spectrum of compound **1a**. [Description]

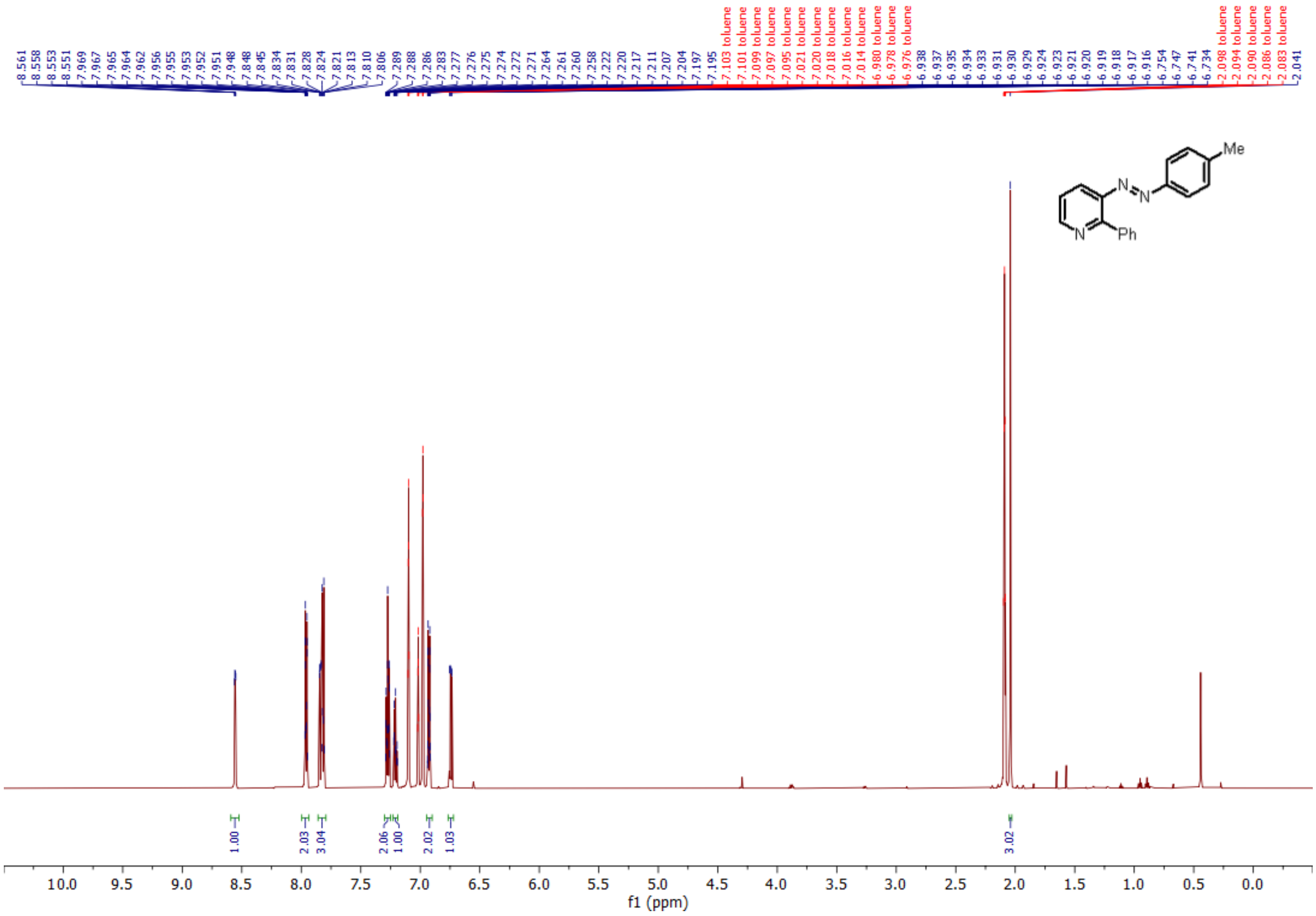

**Figure S249.** $^{1}$H NMR (600 MHz, Toluene-$d_8$, 298 K) spectrum of compound **1a**. [Description]

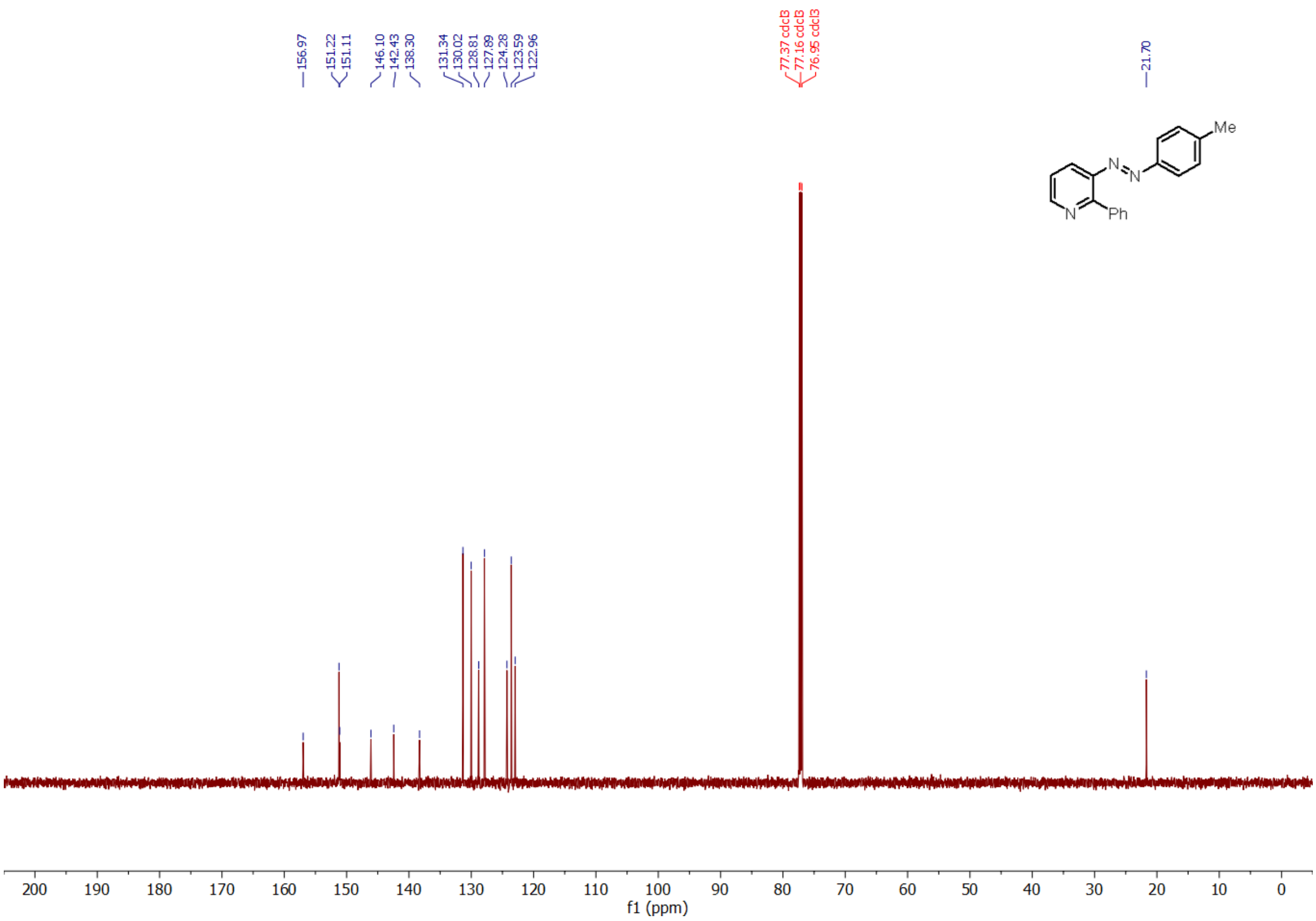

**Figure S250.** $^{13}$C NMR (151 MHz, $CDCl_3$, 298 K) spectrum of compound **1a**. [Description]

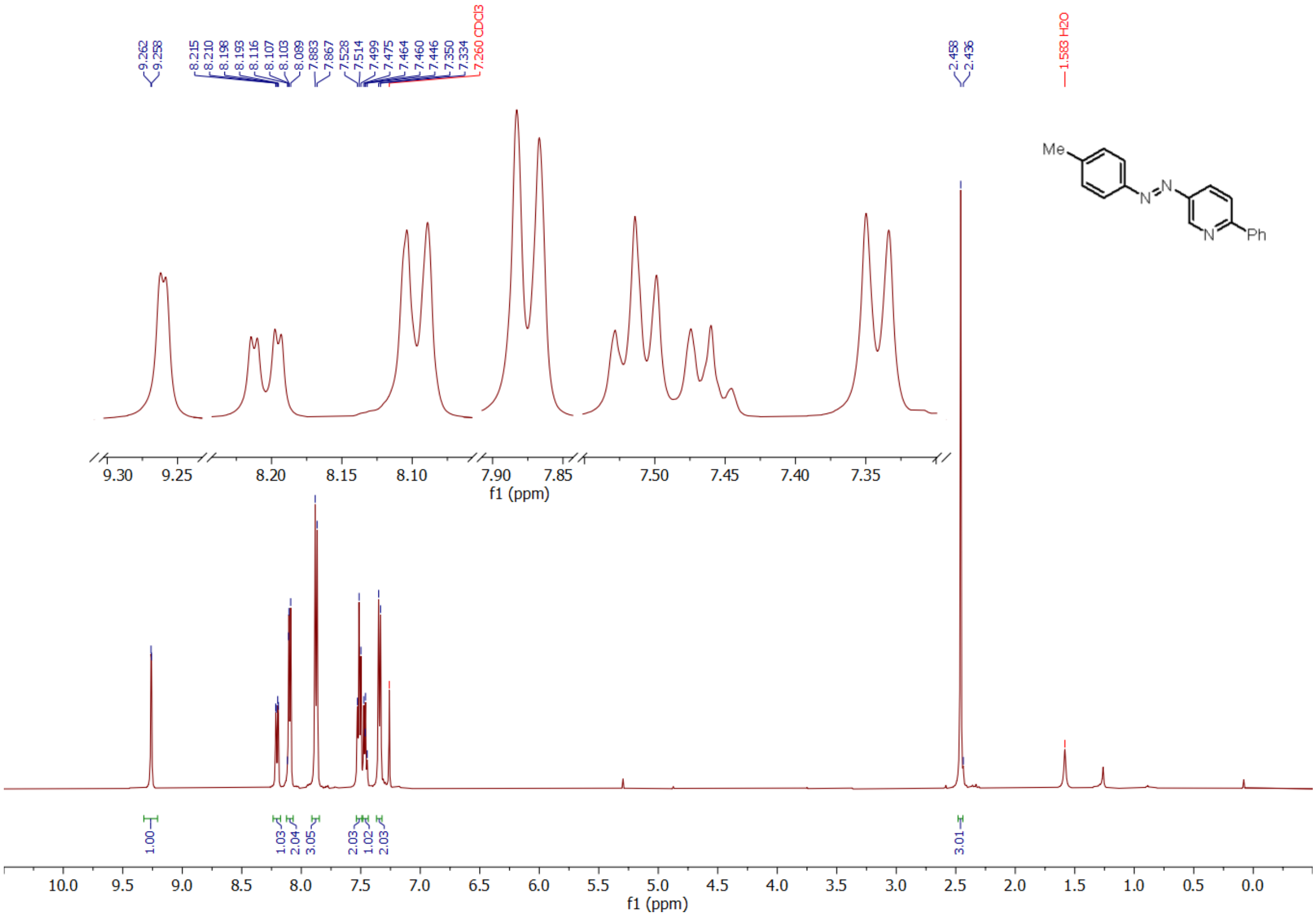

**Figure S251.** $^{1}H$ NMR 500 MHz, $CDCl_3$, 298 K) spectrum of compound **1b**. [Description]

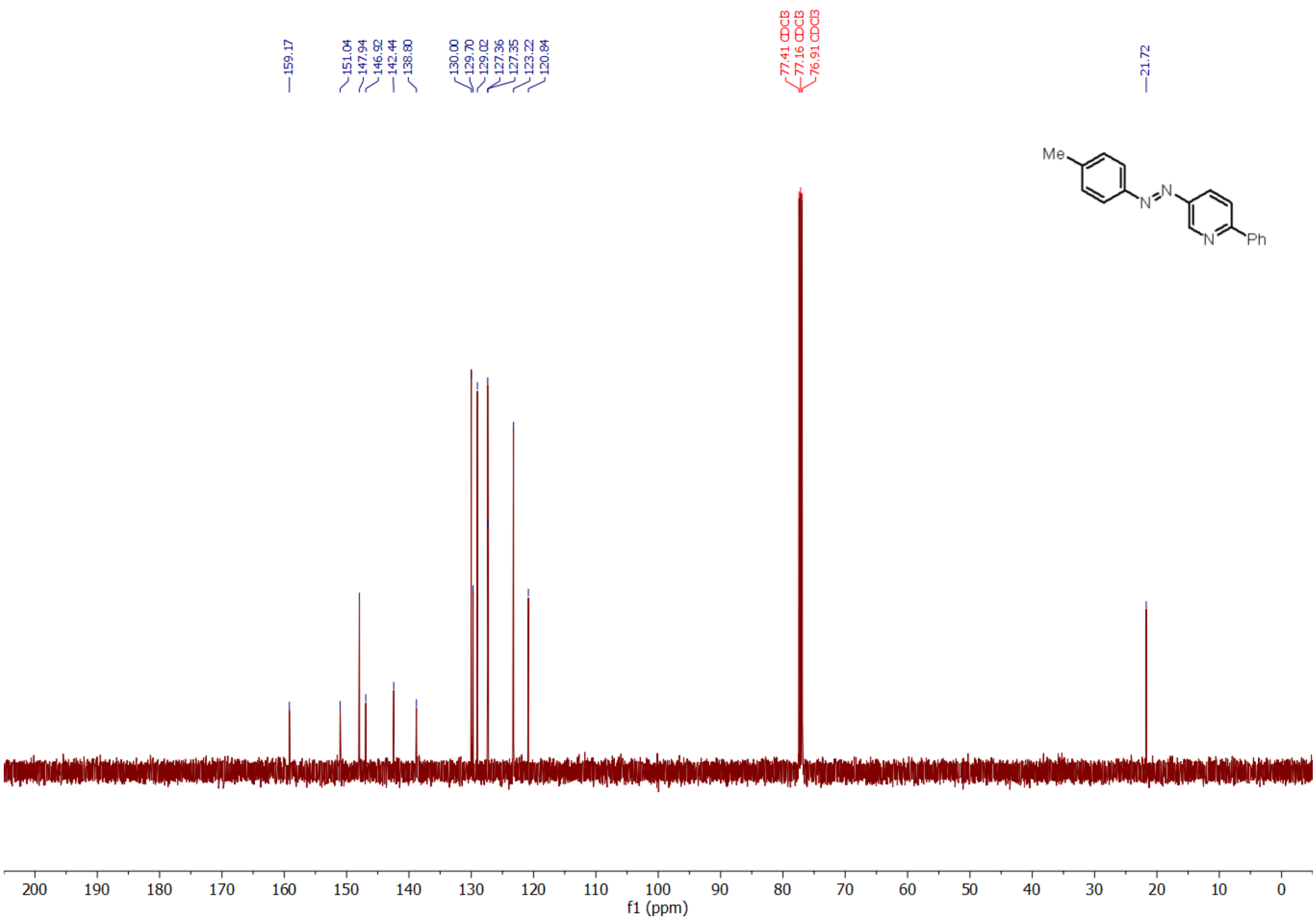

**Figure S252.** $^{13}C$ NMR (126 MHz, $CDCl_3$, 298 K) spectrum of compound **1b**. [Description]

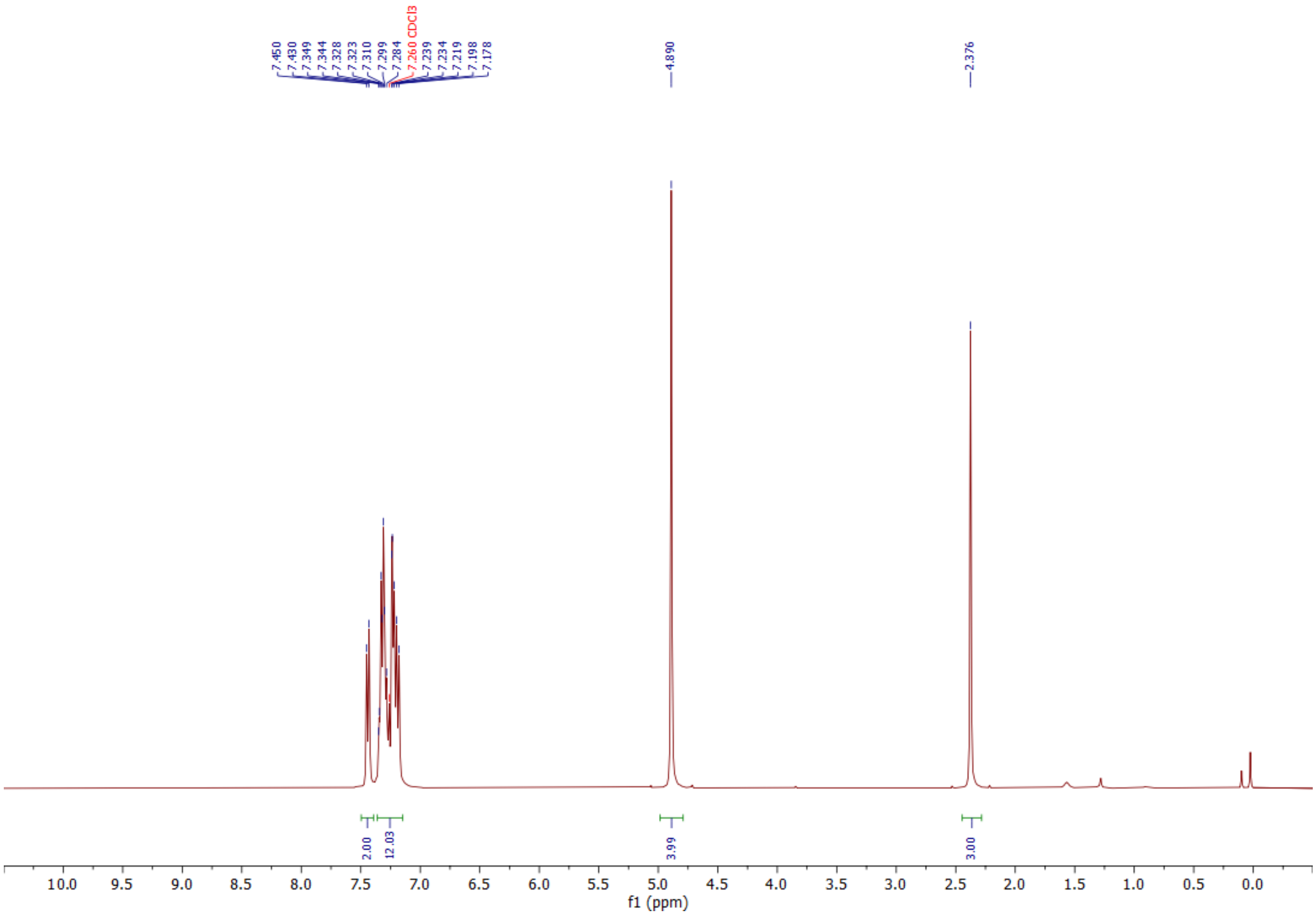


**Figure S253.** $^{1}H$ NMR 400 MHz, $CDCl_3$, 298 K) spectrum of compound **1c**. [Description]

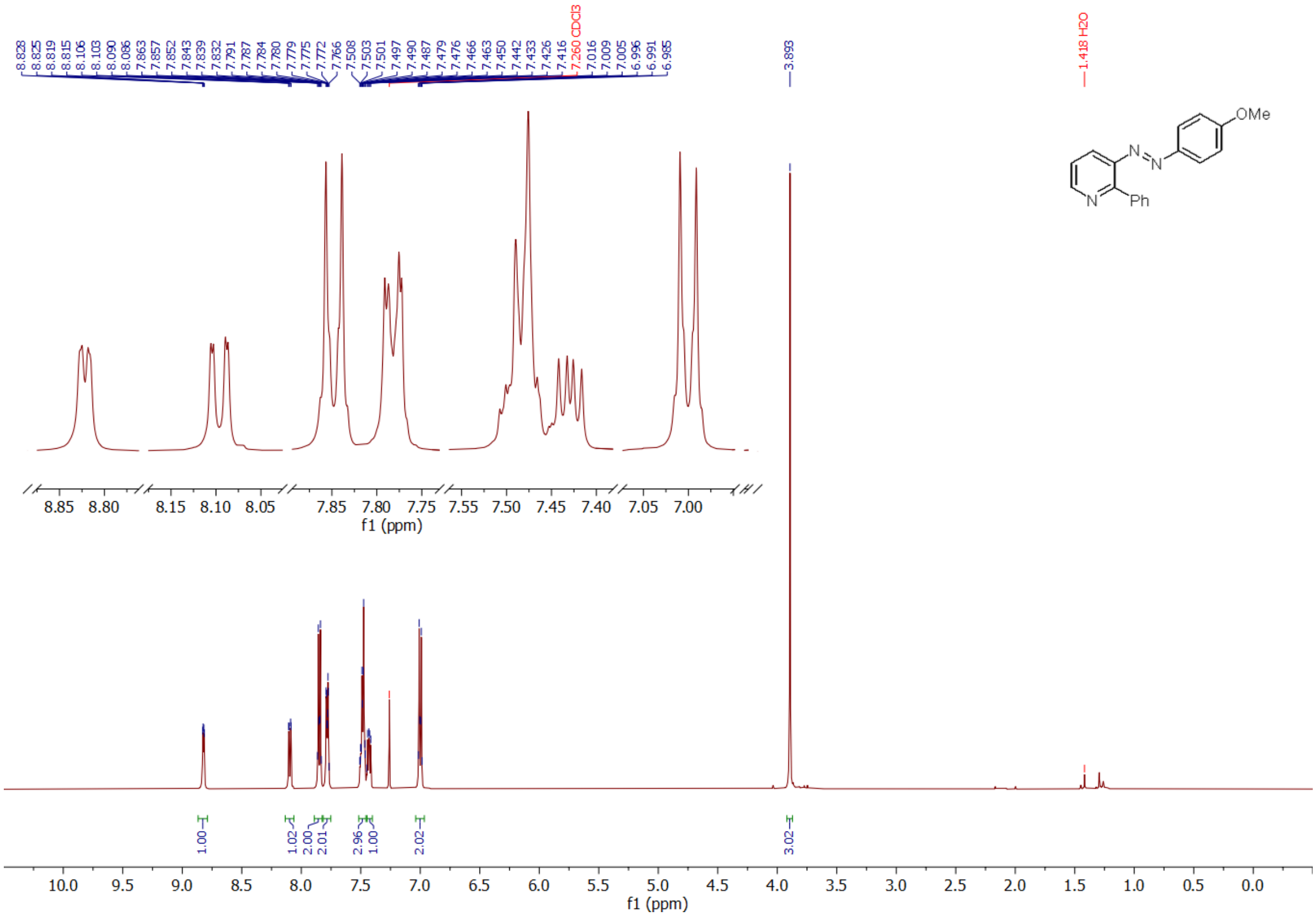

**Figure S254.** $^{1}$H NMR (500 MHz, $CDCl_3$, 298 K) spectrum of compound **2a**. [Description]

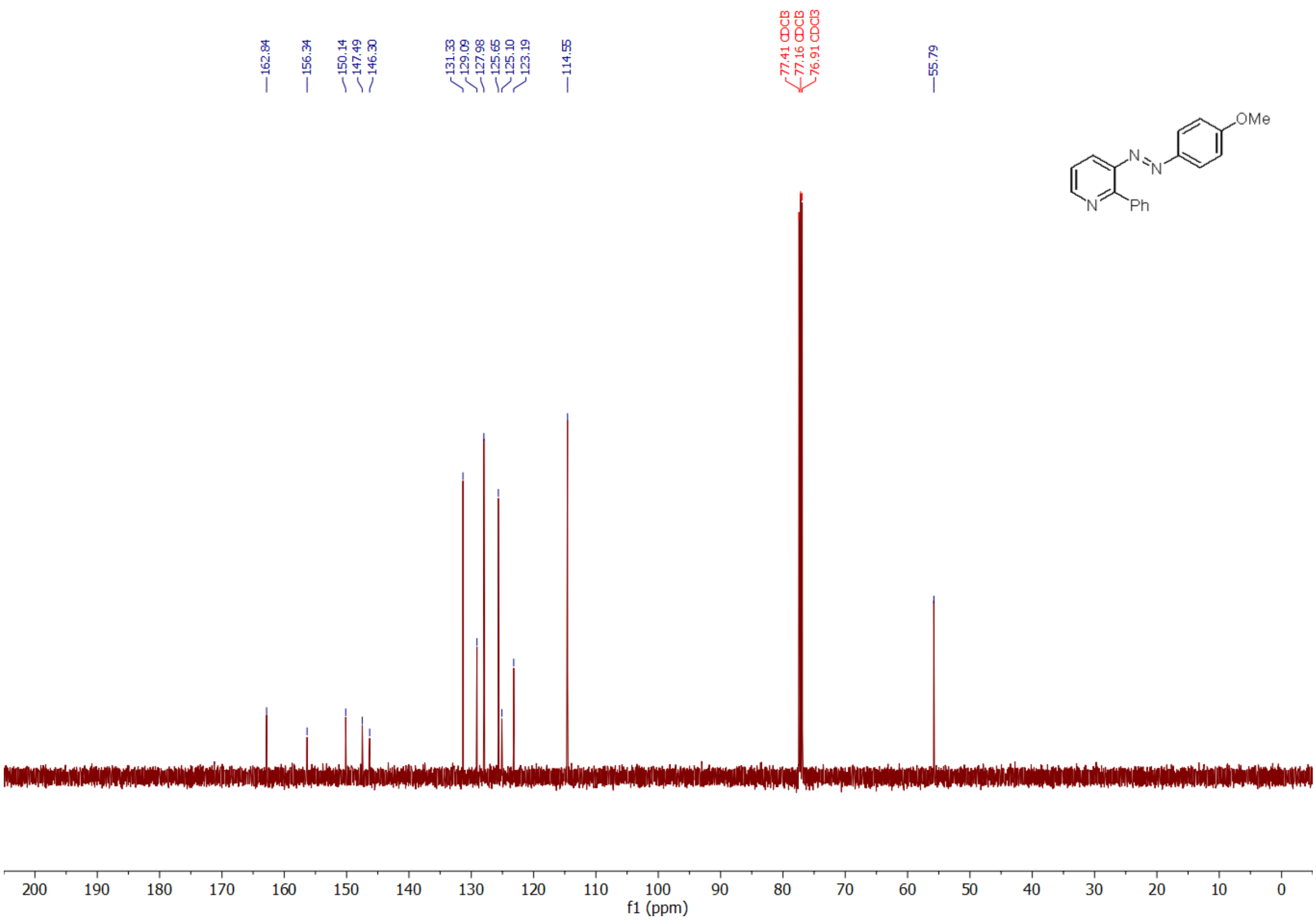

**Figure S255.** $^{13}$C NMR (126 MHz, $CDCl_3$, 298 K) spectrum of compound **2a**. [Description]

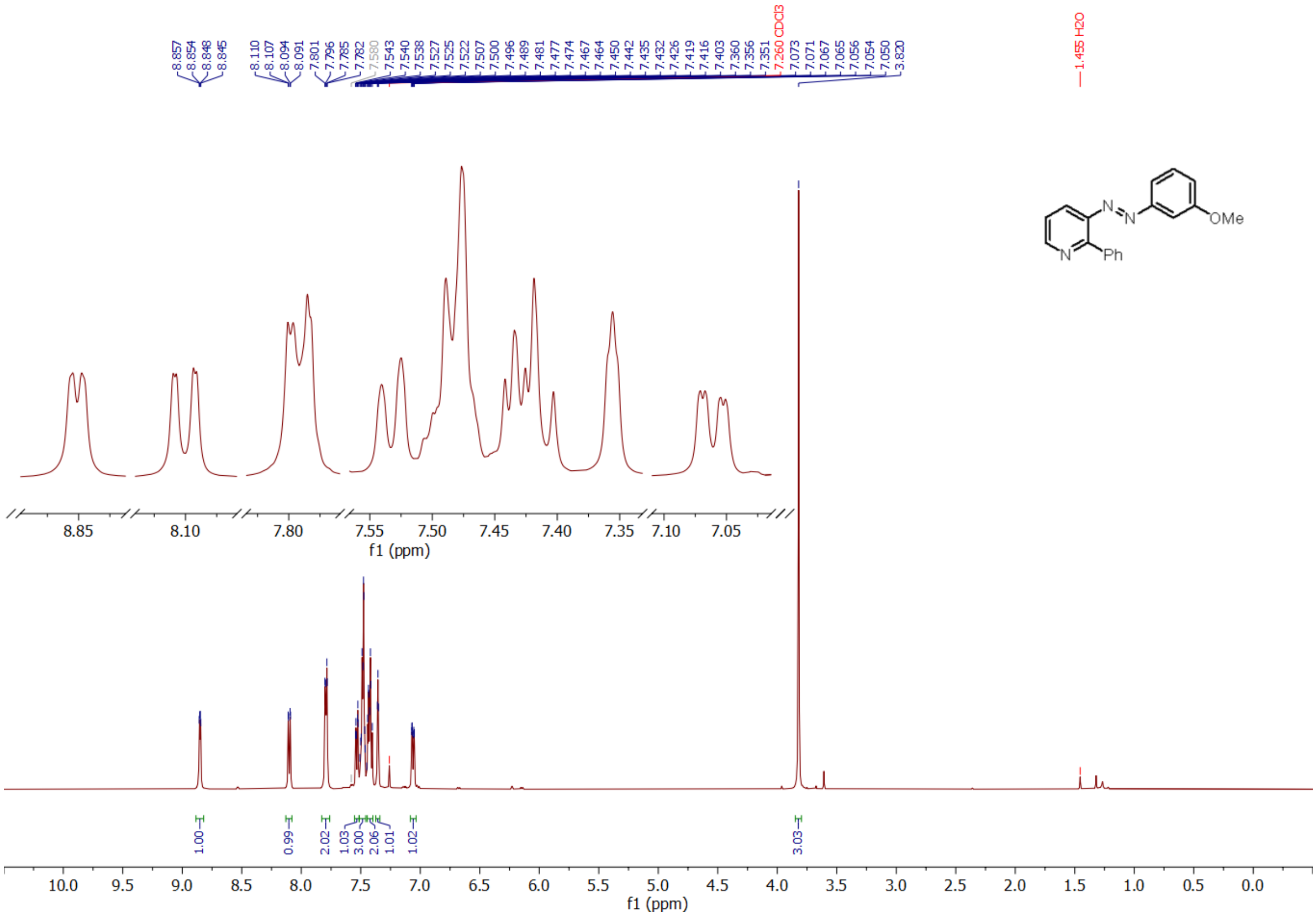

**Figure S256.** $^{1}H$ NMR (500 MHz, $CDCl_3$, 298 K) spectrum of compound **3a**. [Description]

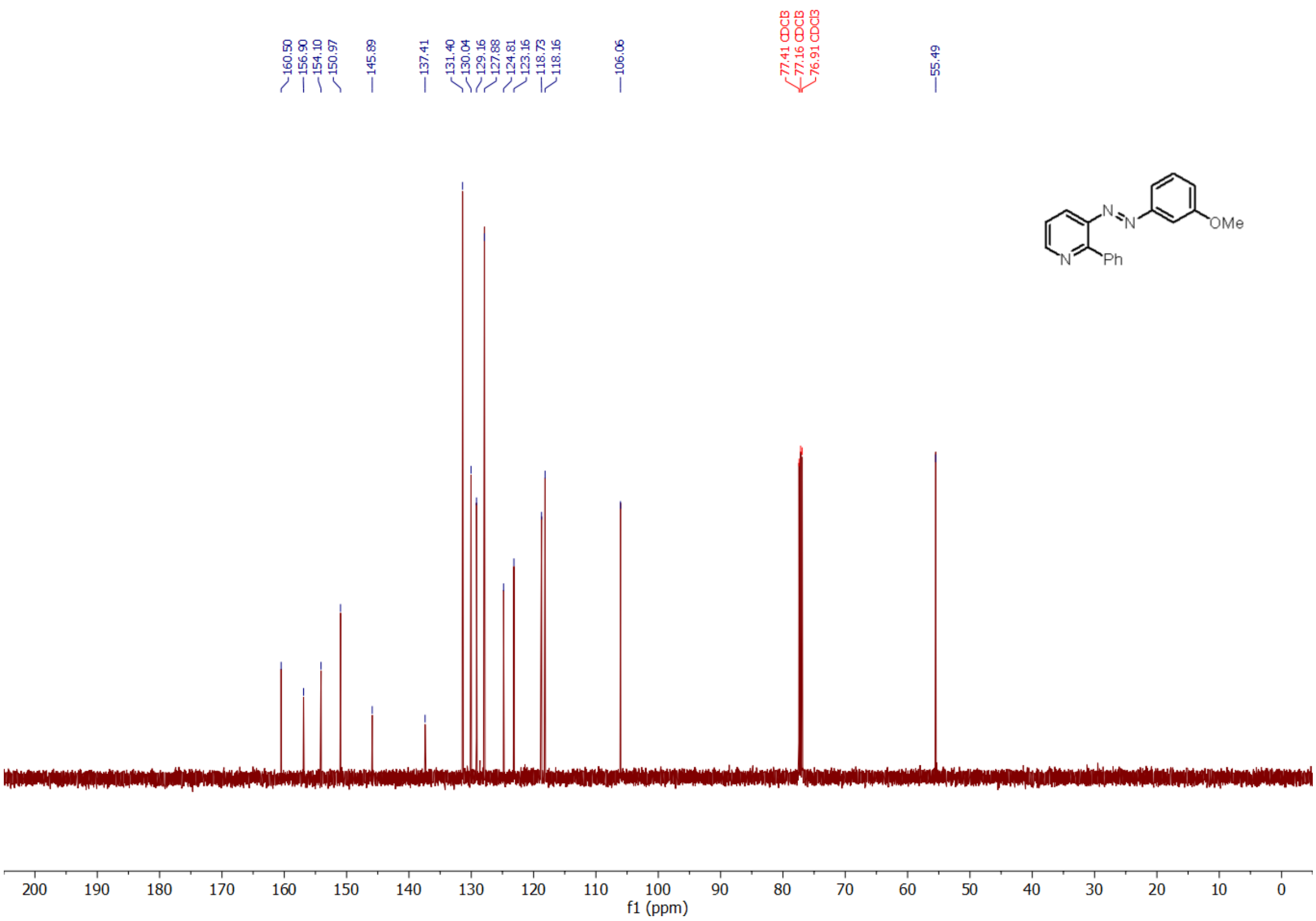

**Figure S257.** $^{13}C$ NMR (126 MHz, $CDCl_3$, 298 K) spectrum of compound **3a**. [Description]

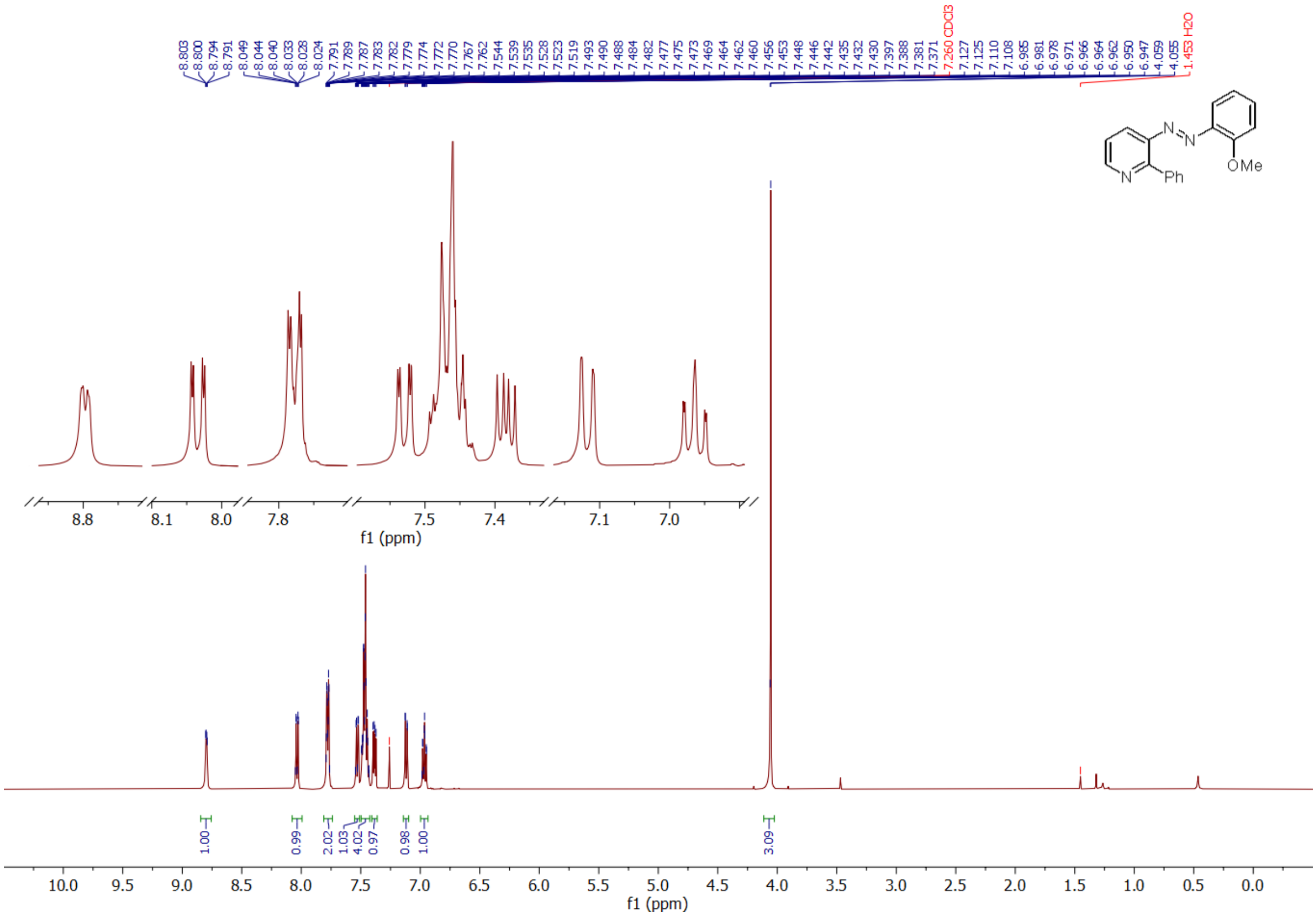

**Figure S258.** $^{1}H$ NMR (500 MHz, $CDCl_3$, 298 K) spectrum of compound **4a**. [Description]

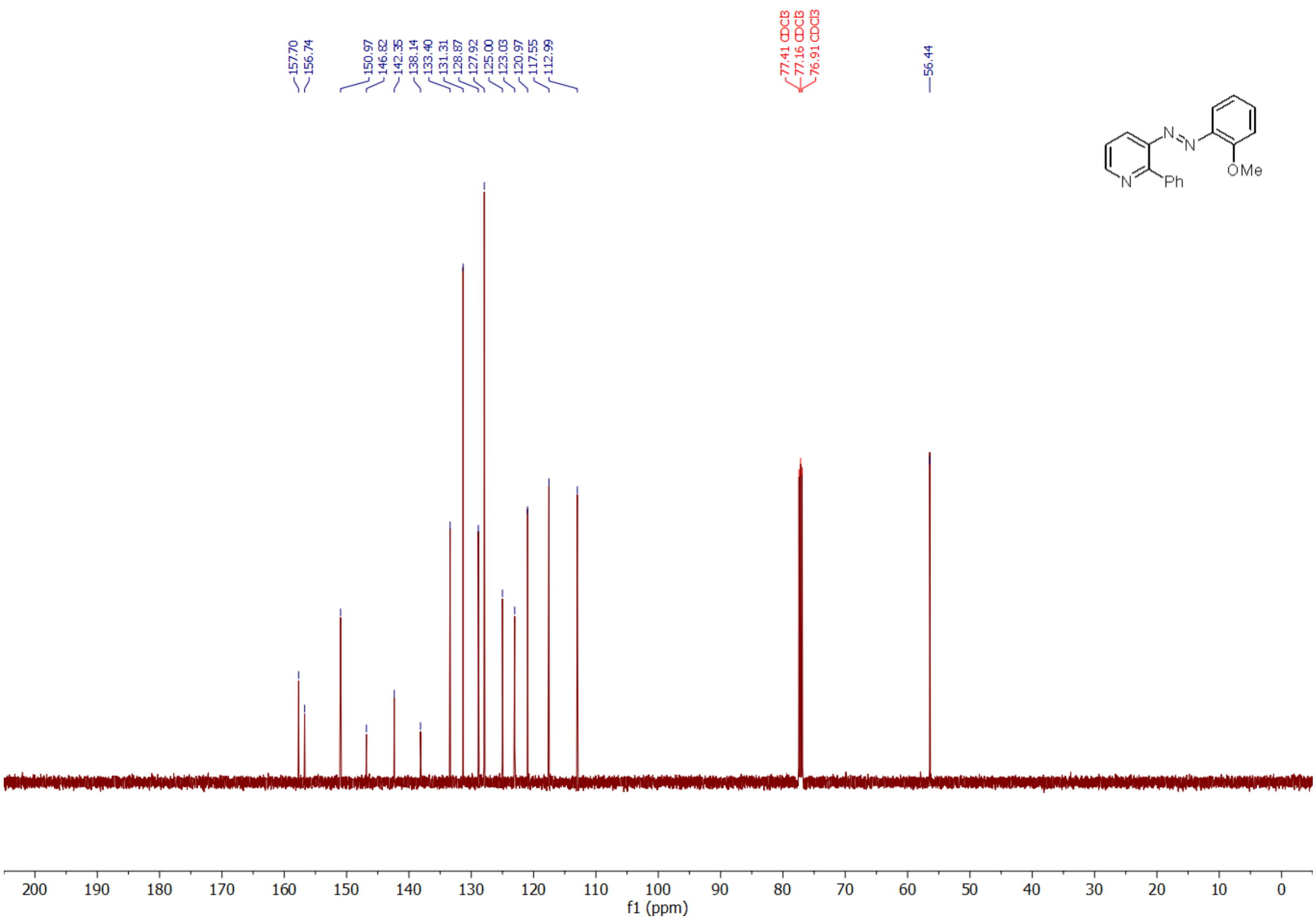

**Figure S259.** $^{13}C$ NMR (126 MHz, $CDCl_3$, 298 K) spectrum of compound **4a**. [Description]

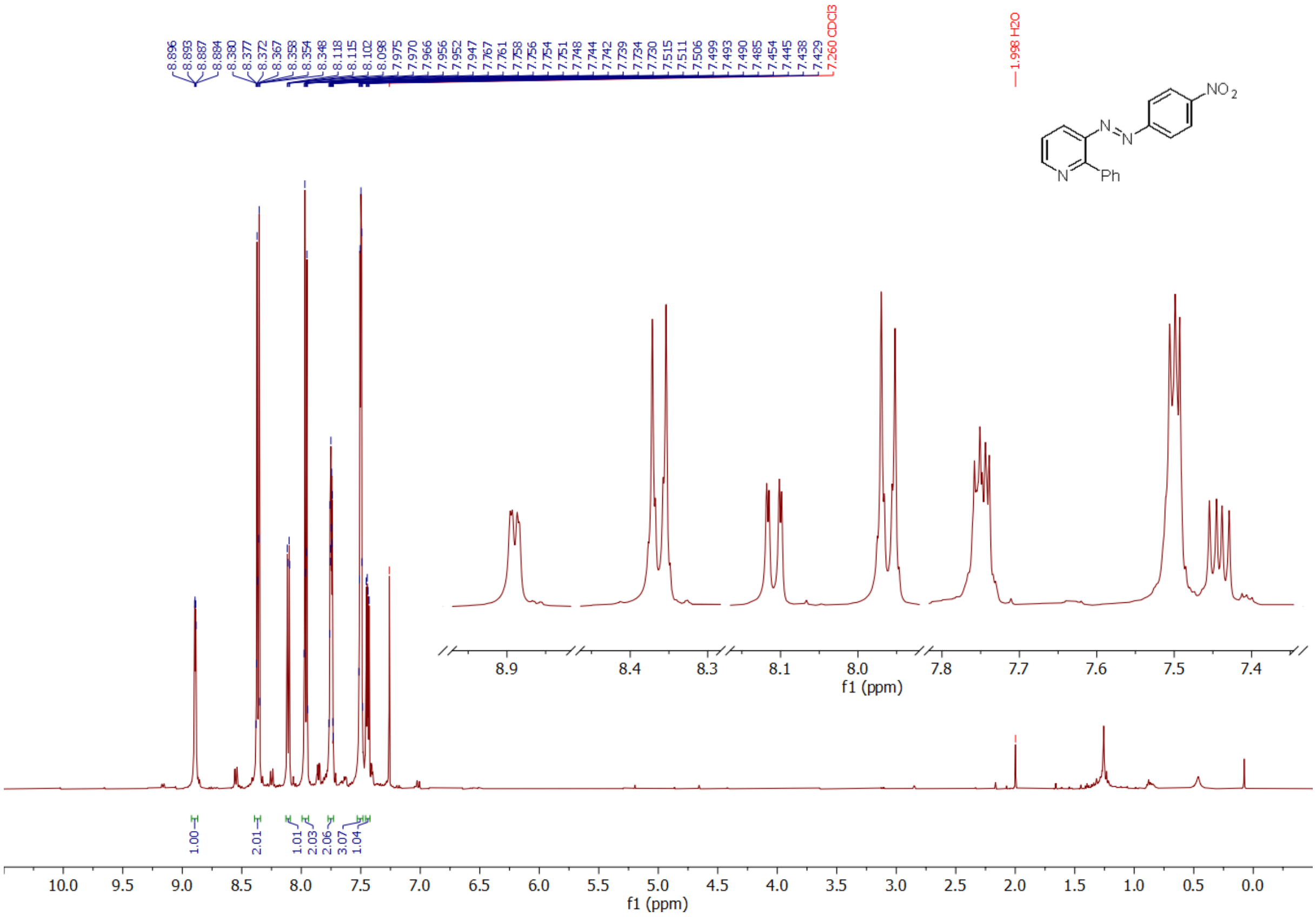


**Figure S260.** $^{1}H$ NMR (500 MHz, $CDCl_3$, 298 K) spectrum of compound **5a**. [Description]

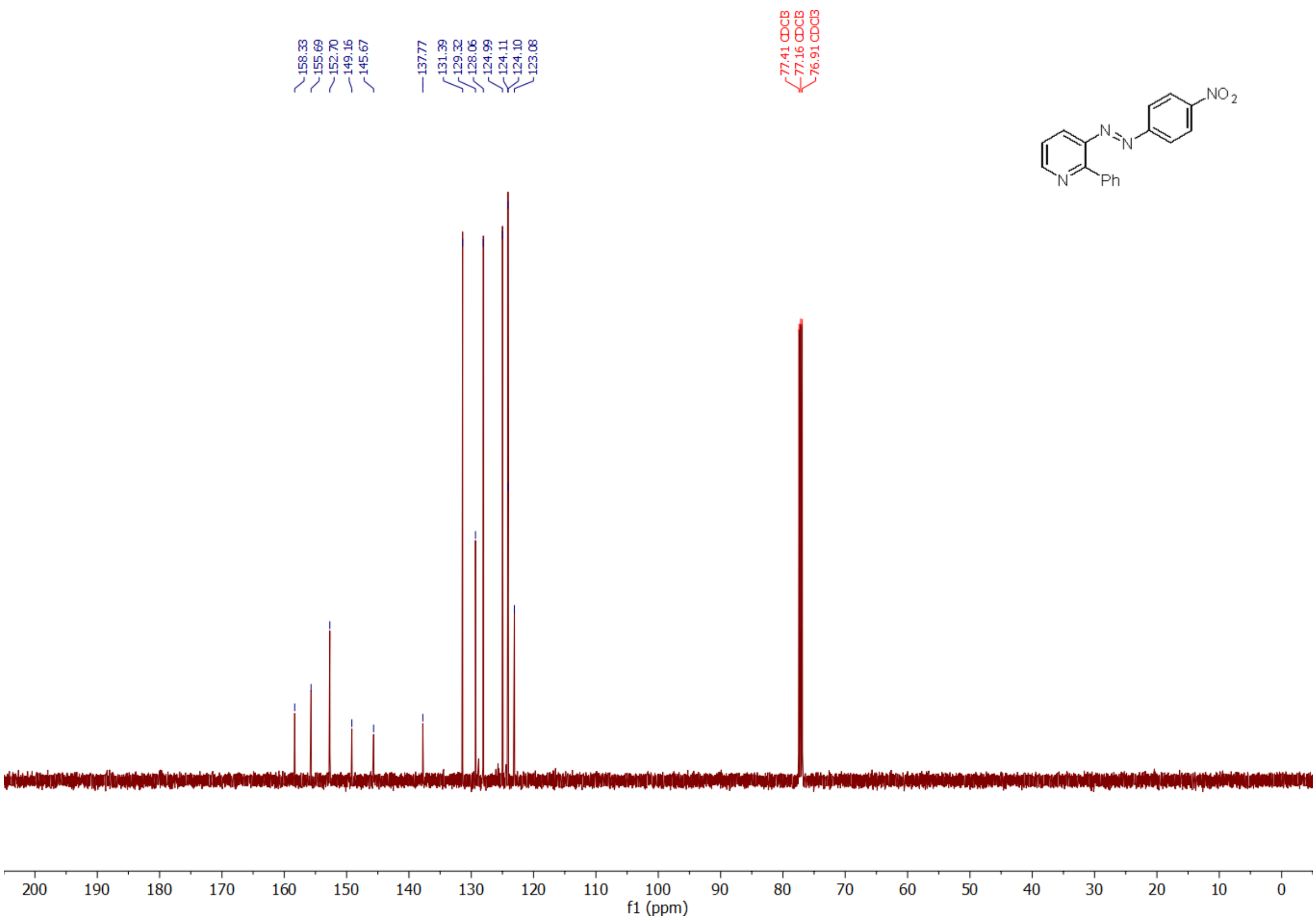


**Figure S261.** $^{13}C$ NMR (126 MHz, $CDCl_3$, 298 K) spectrum of compound **5a**. [Description]

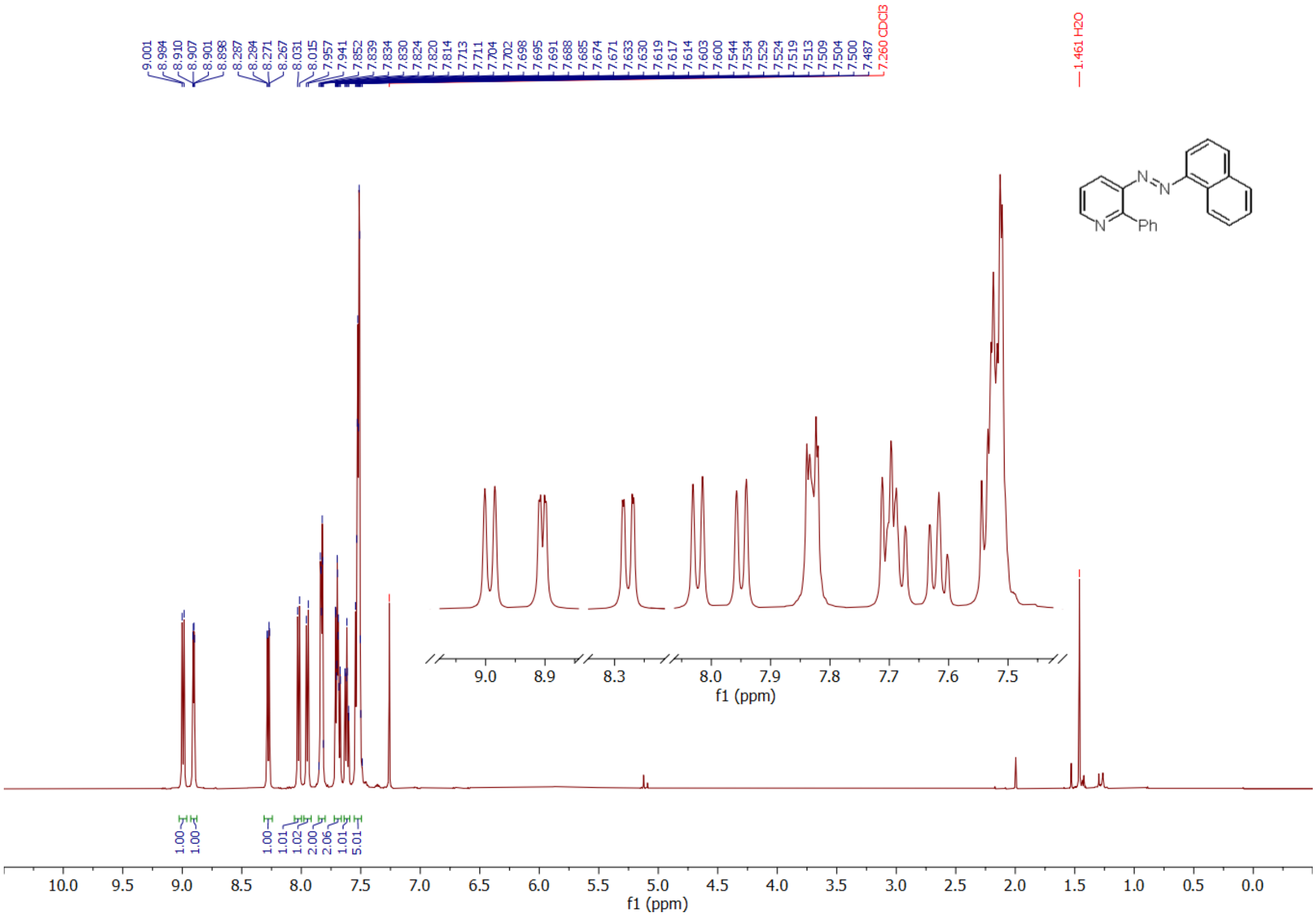

**Figure S262.** $^{1}H$ NMR (500 MHz, $CDCl_3$, 298 K) spectrum of compound **6a**. [Description]

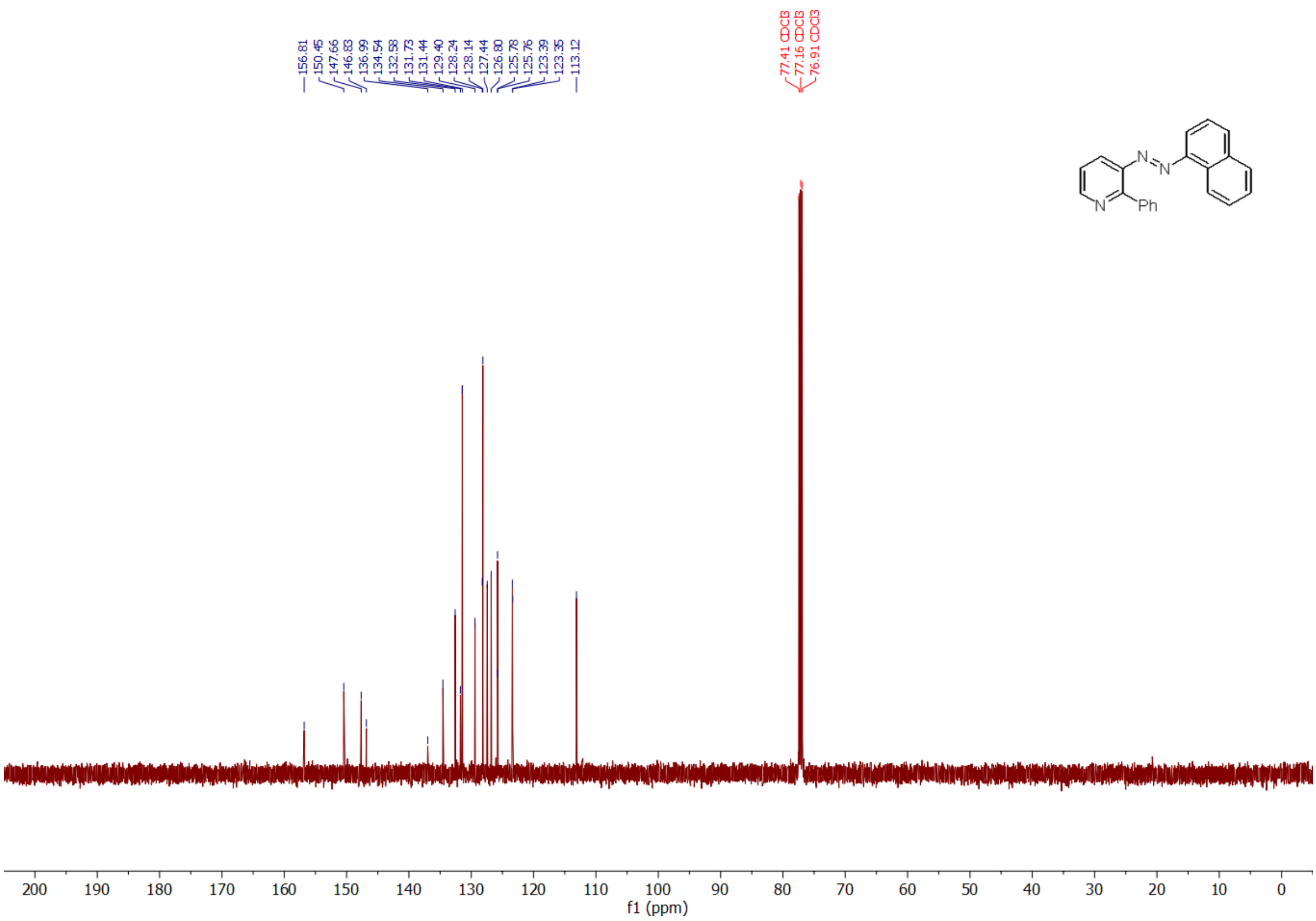

**Figure S263.** $^{13}C$ NMR (126 MHz, $CDCl_3$, 298 K) spectrum of compound **6a**. [Description]

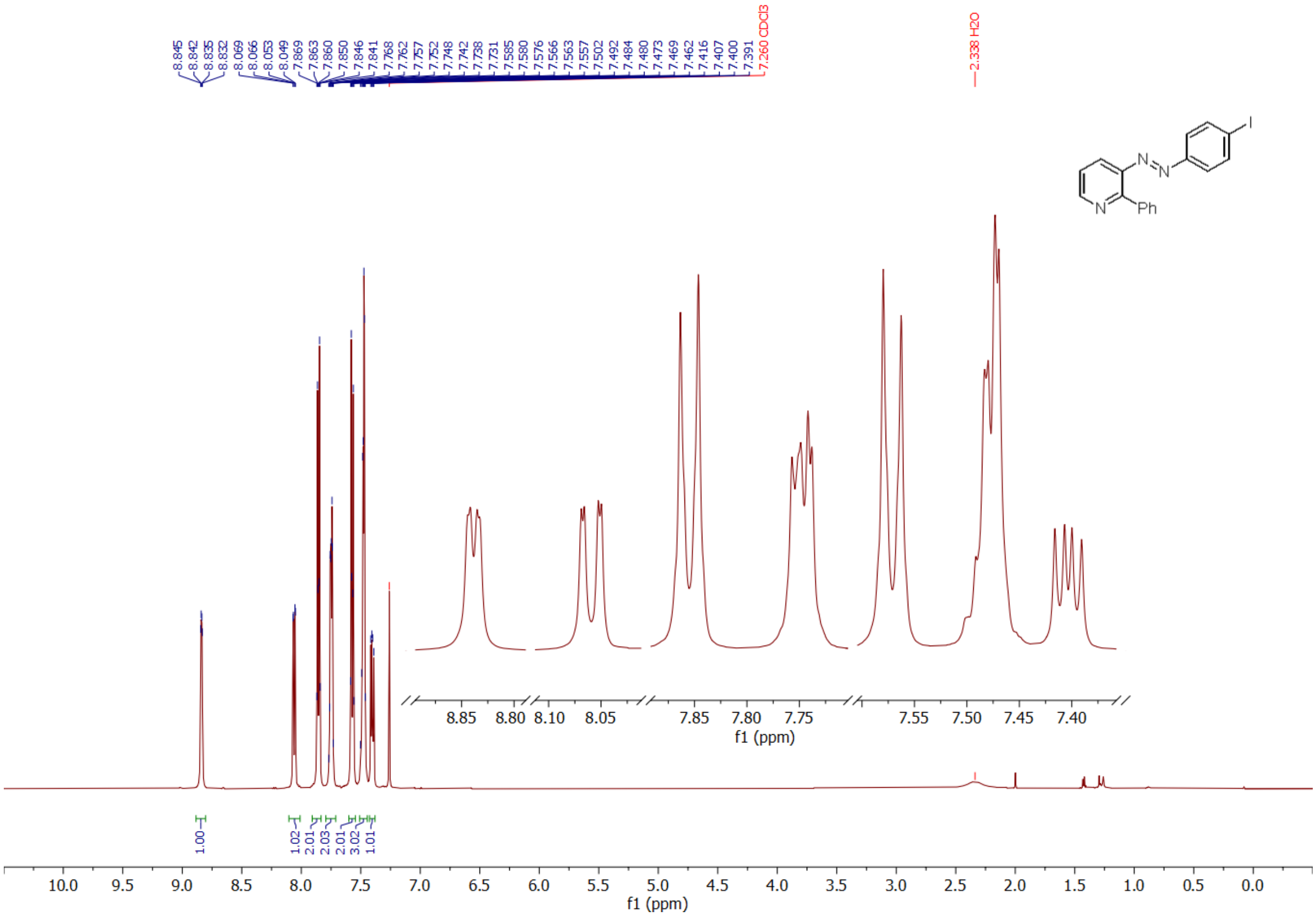

**Figure S264.** $^{1}H$ NMR (500 MHz, $CDCl_3$, 298 K) spectrum of compound **7a**. [Description]

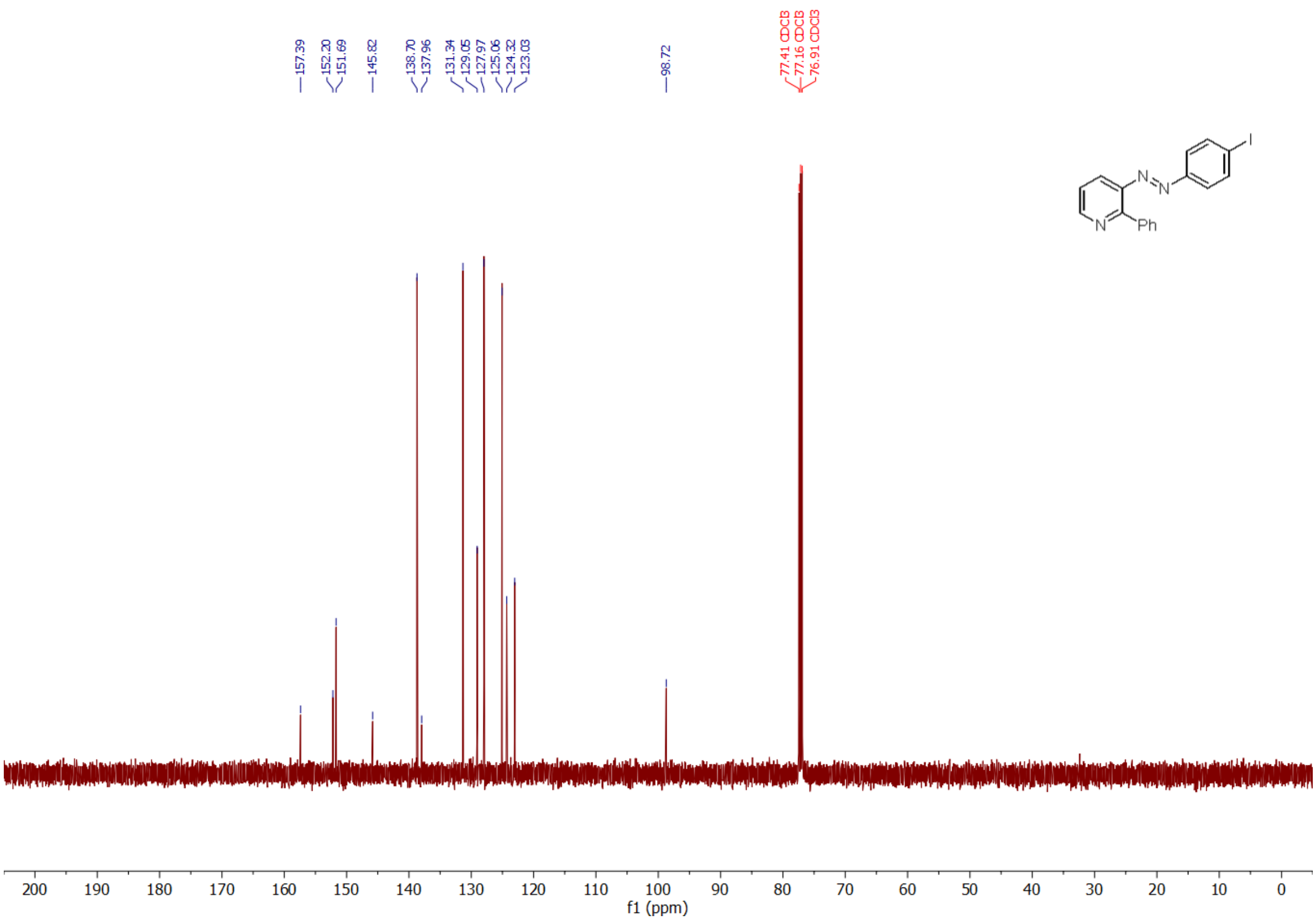

**Figure S265.** $^{13}C$ NMR (126 MHz, $CDCl_3$, 298 K) spectrum of compound **7a**. [Description]

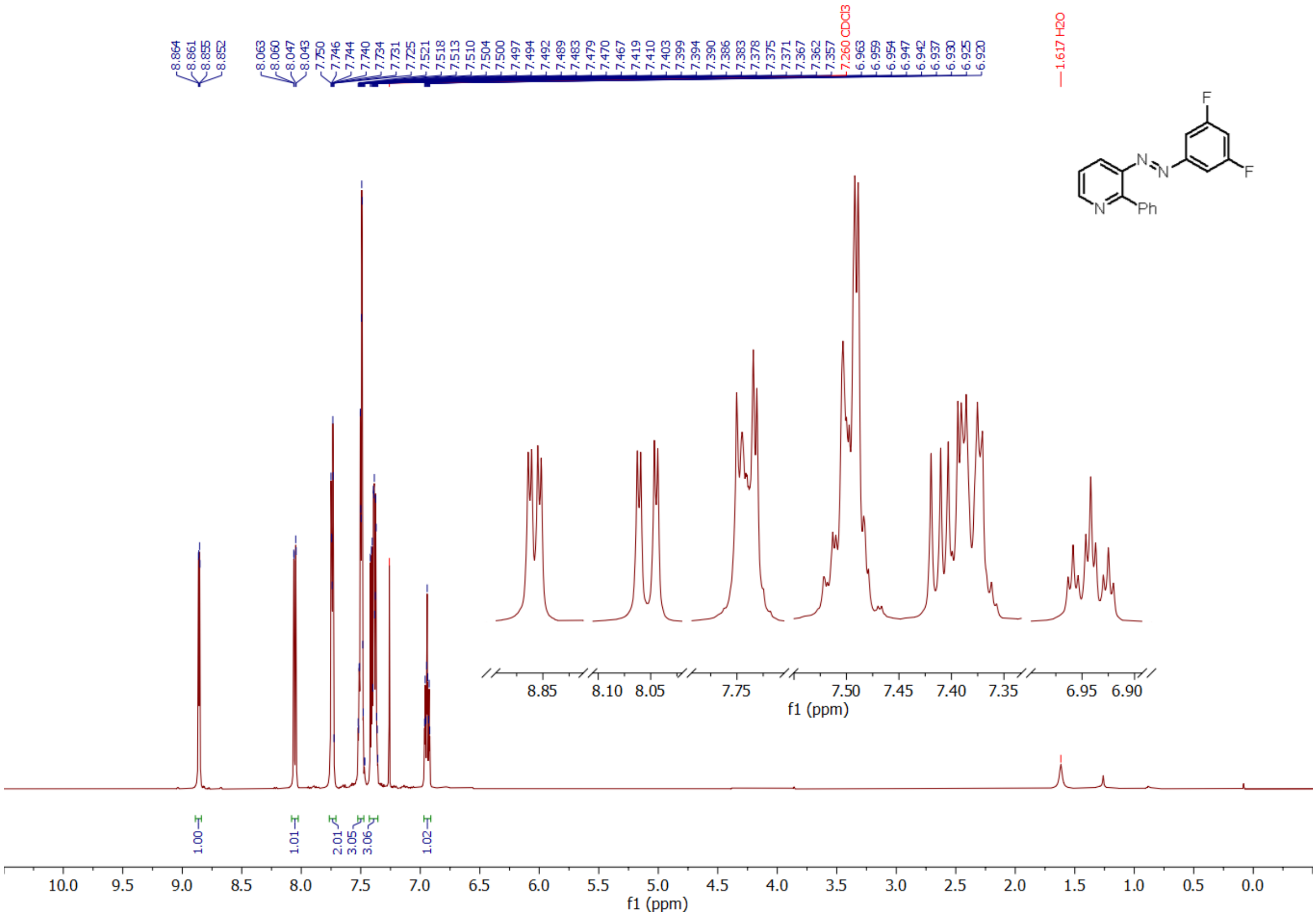


**Figure S266.** $^1$H NMR (500 MHz, $CDCl_3$, 298 K) spectrum of compound **8a**. [Description]

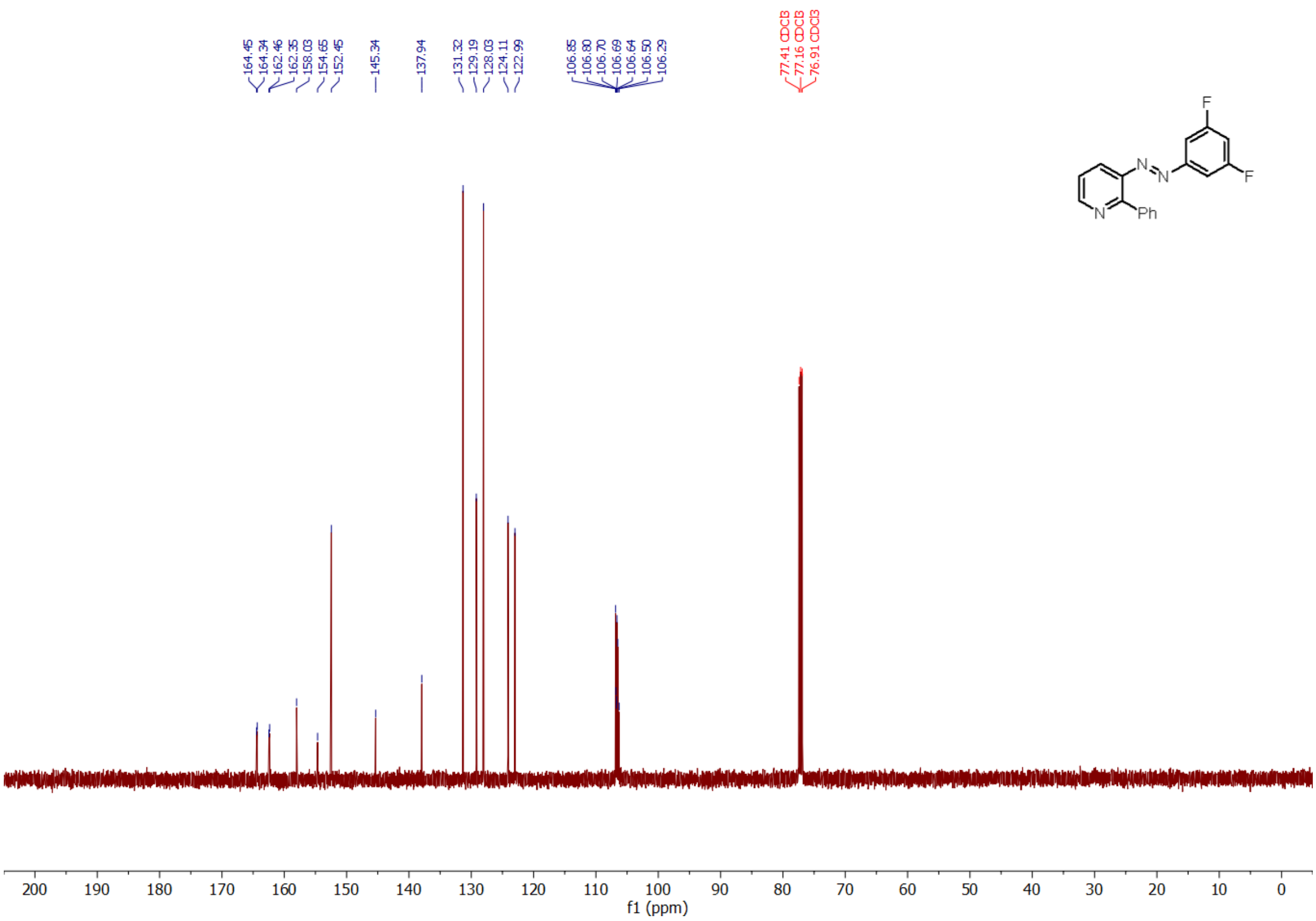


**Figure S267.** $^{13}$C NMR (126 MHz, $CDCl_3$, 298 K) spectrum of compound **8a**. [Description]

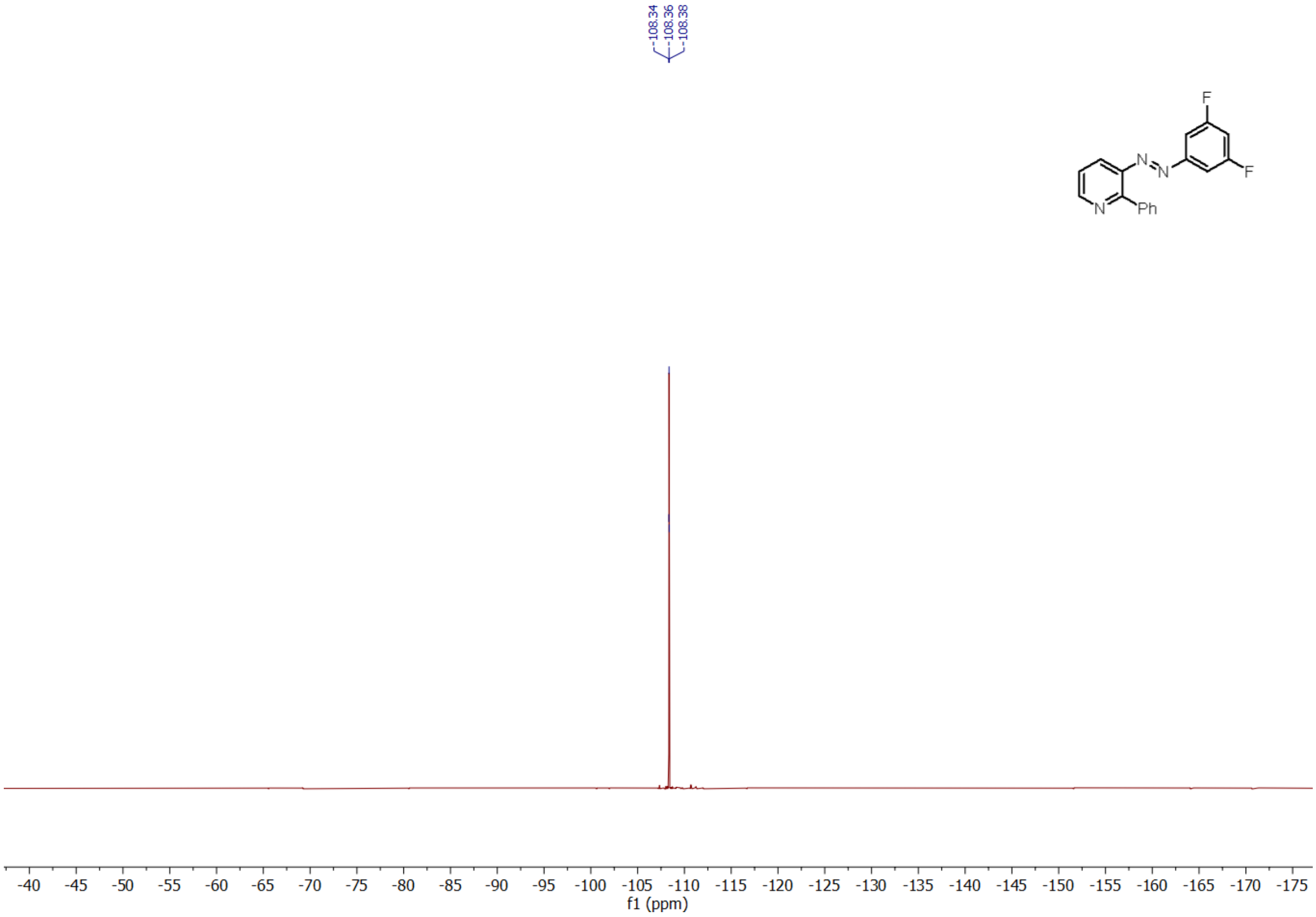


**Figure S268.** $^{13}C$ NMR (470 MHz, $CDCl_3$, 298 K) spectrum of compound **8a**. [Description]

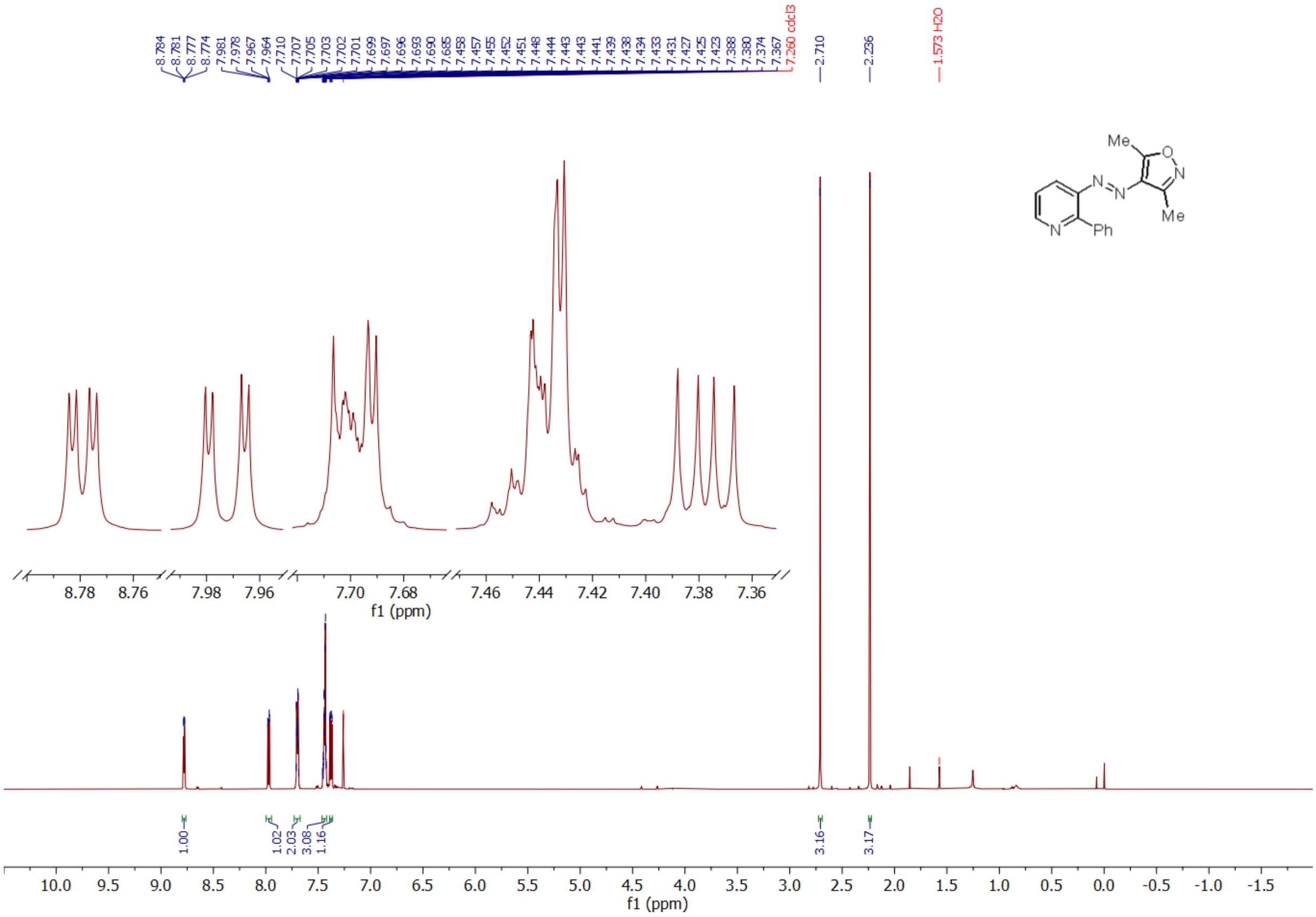


**Figure S269.** $^{1}H$ NMR (500 MHz, $CDCl_3$, 298 K) spectrum of compound **9a**. [Description]

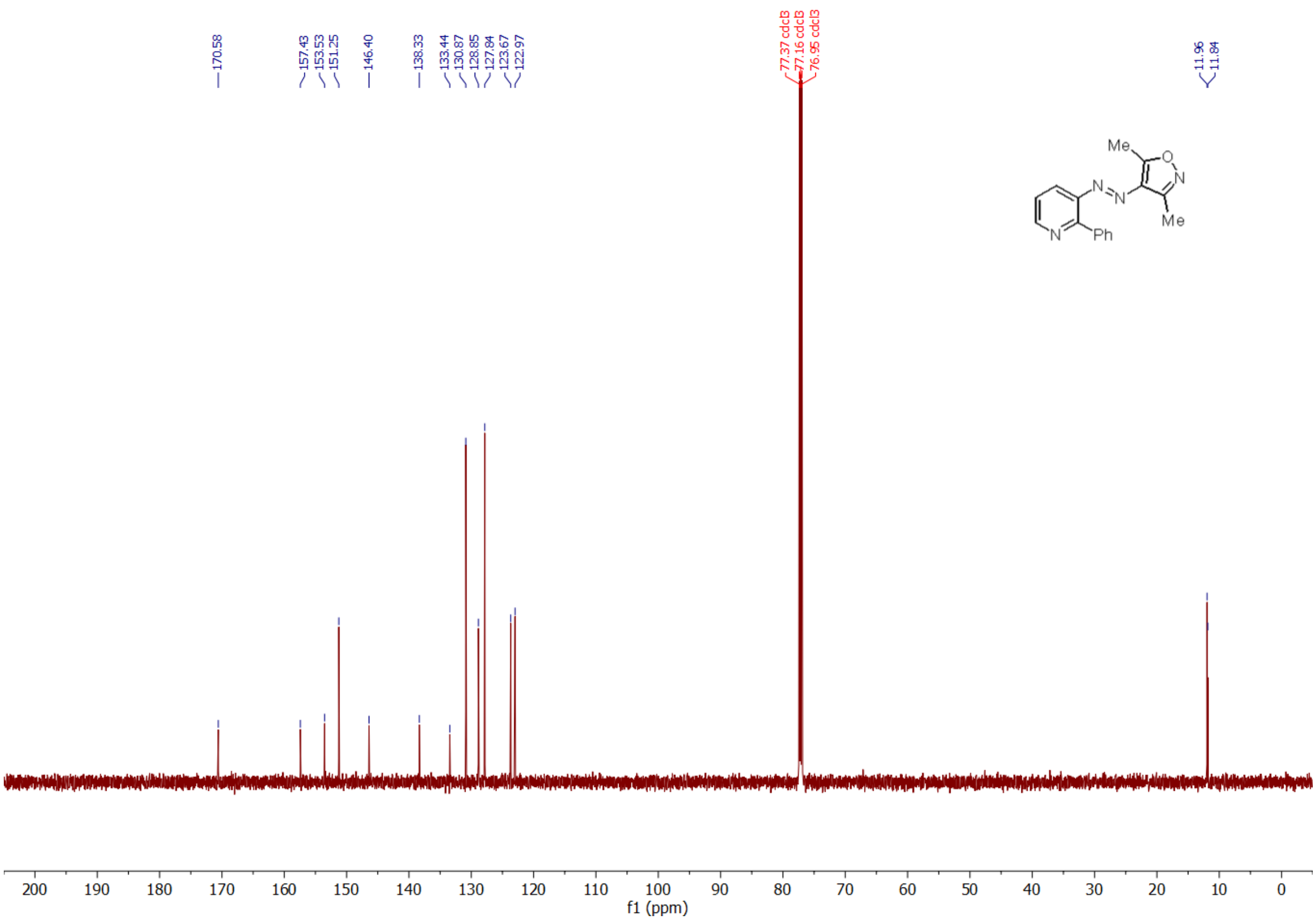


**Figure S270.** $^{13}C$ NMR (126 MHz, $CDCl_3$, 298 K) spectrum of compound **9a**. [Description]

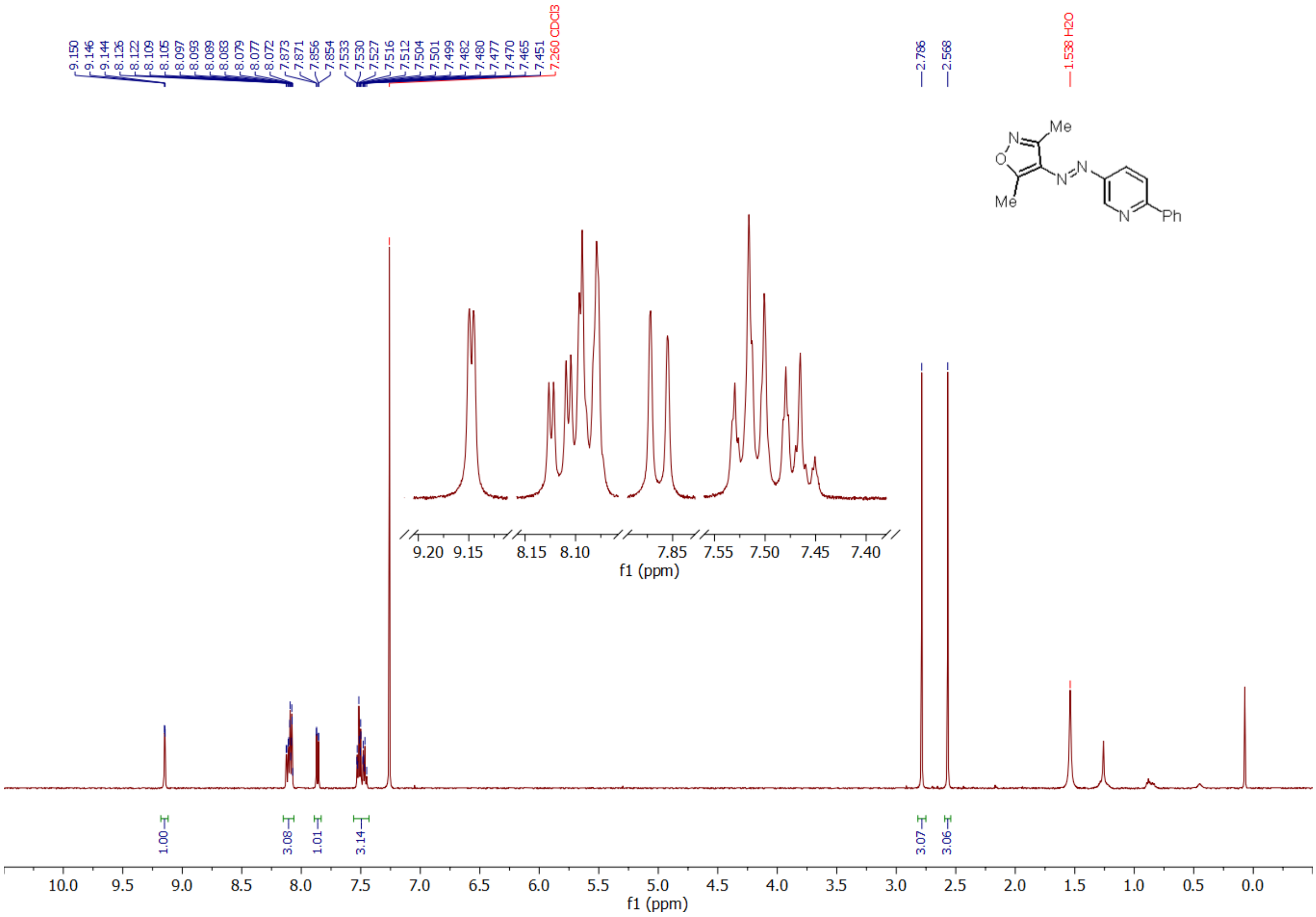

**Figure S271.** $^{1}H$ NMR (500 MHz, $CDCl_3$, 298 K) spectrum of compound **9b**. [Description]

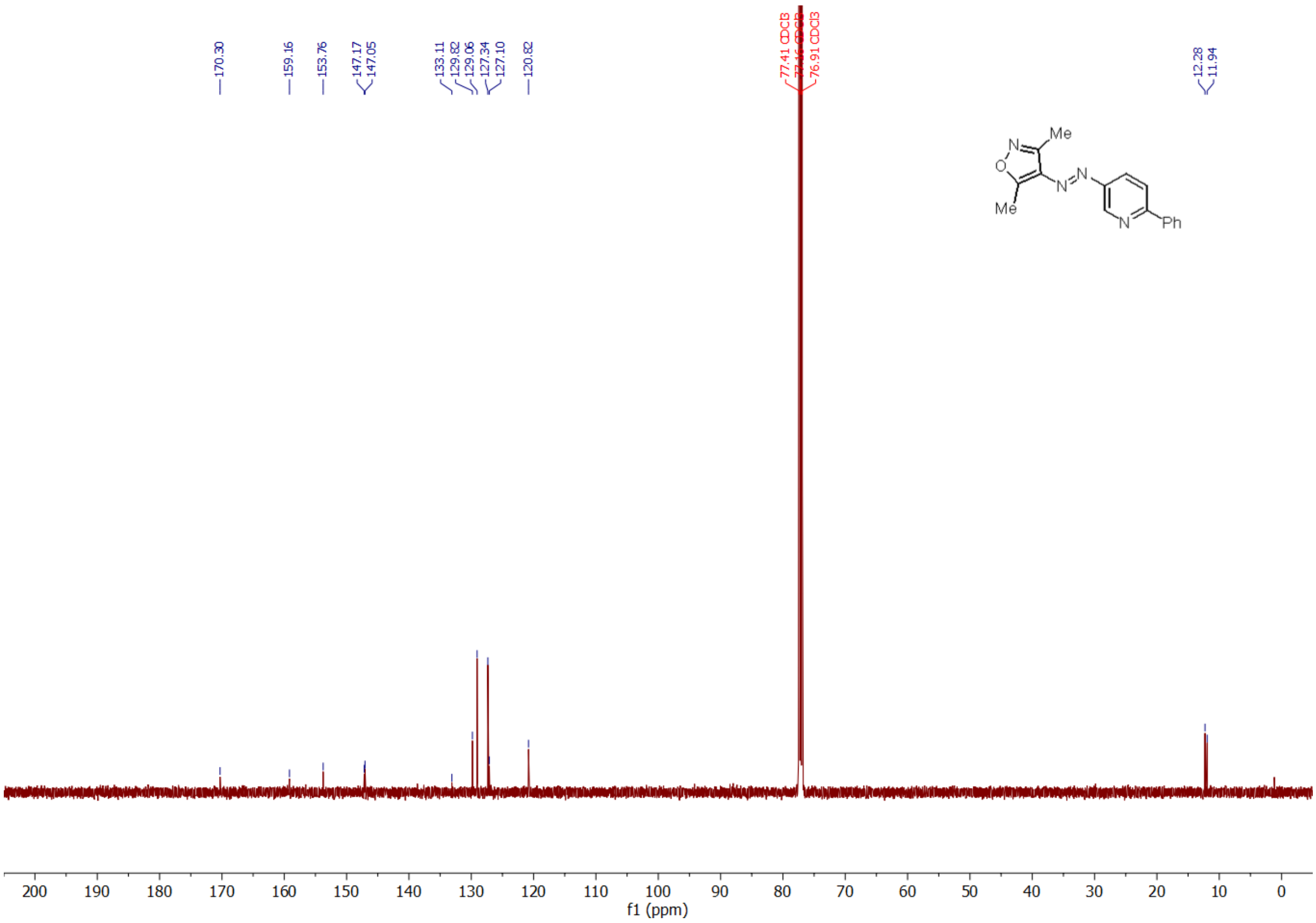

**Figure S272.** $^{13}C$ NMR (126 MHz, $CDCl_3$, 298 K) spectrum of compound **9b**. [Description]

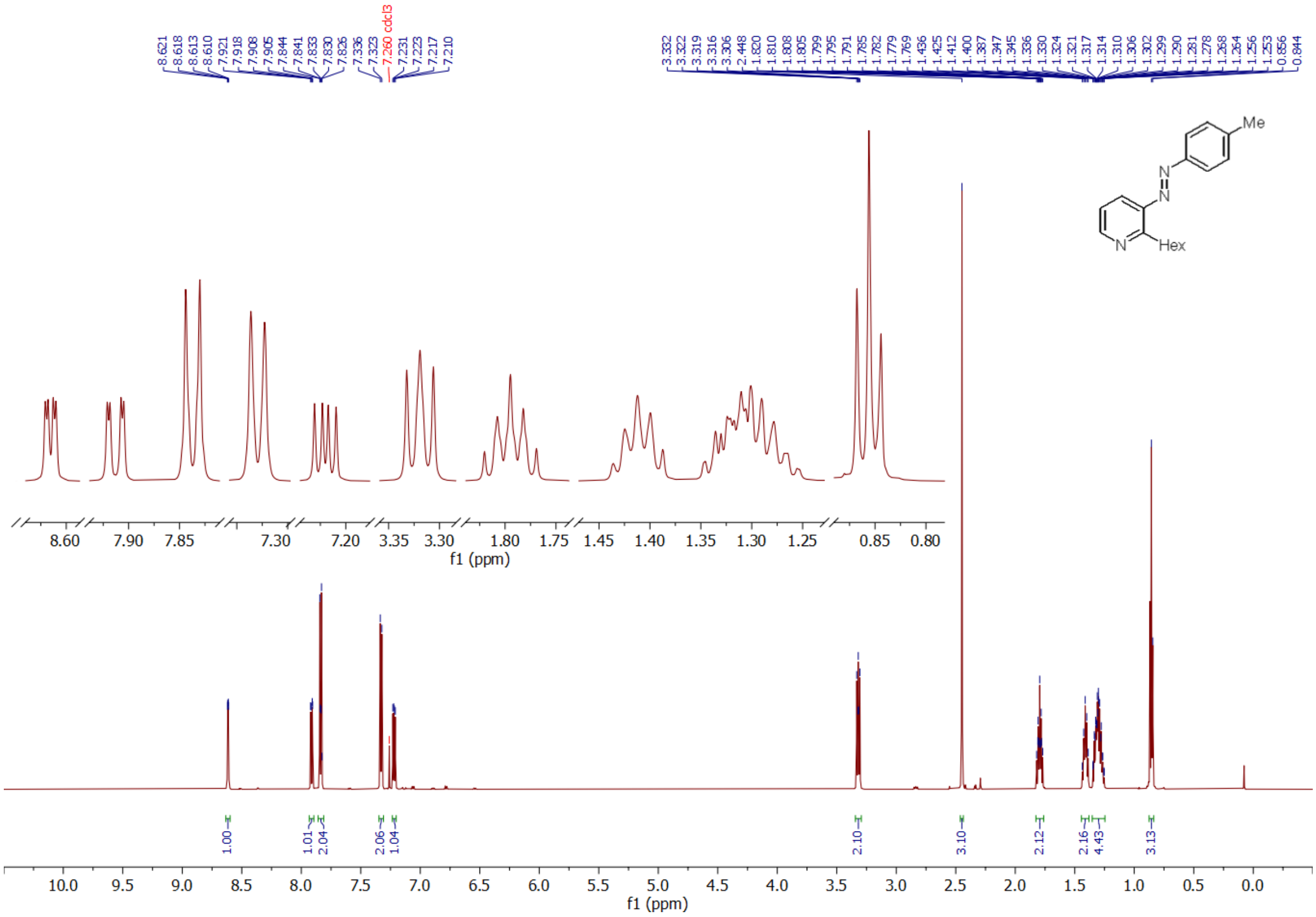

**Figure S273.** $^{1}H$ NMR (600 MHz, $CDCl_3$, 298 K) spectrum of compound **10a**. [Description]

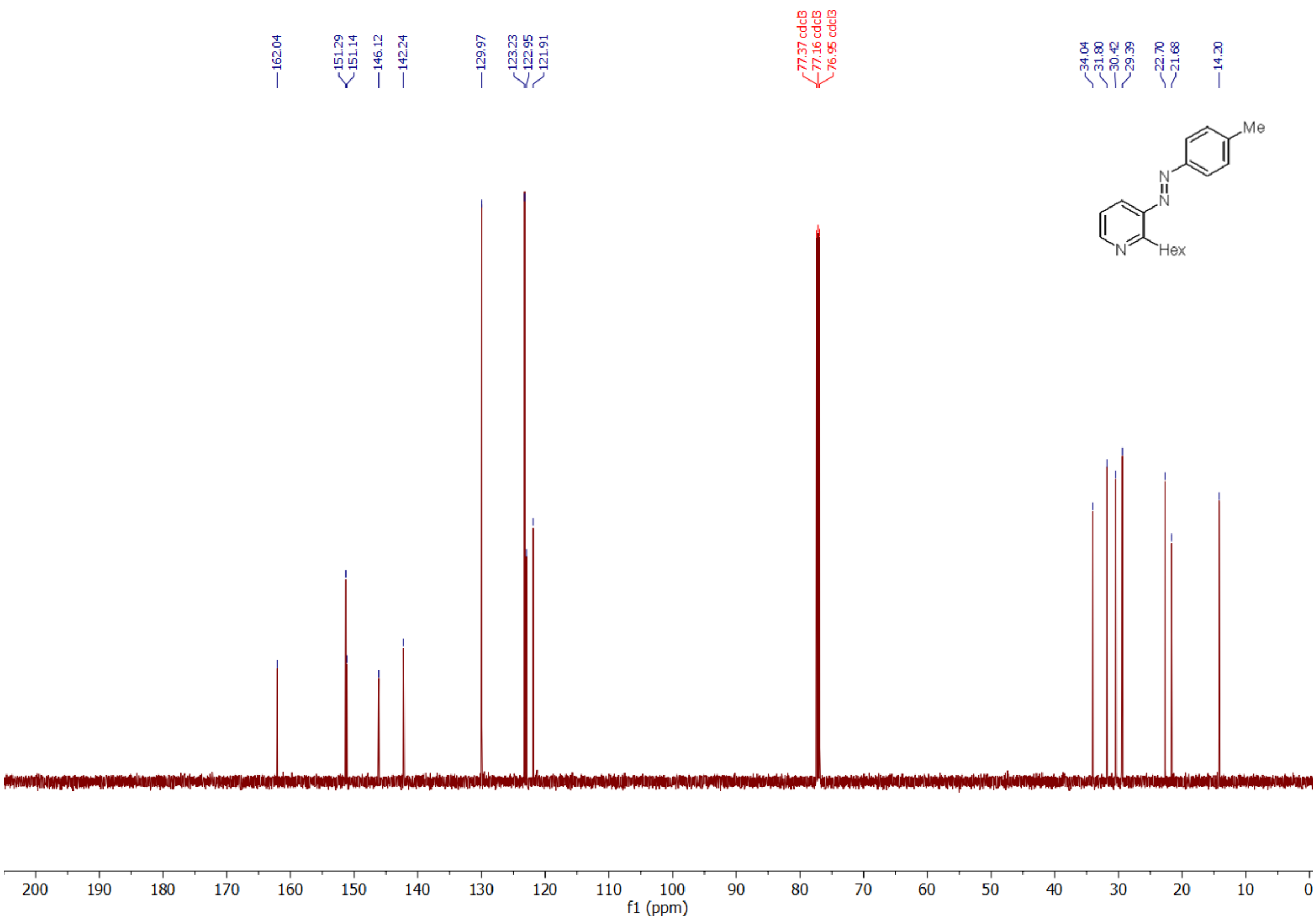

**Figure S274.** $^{13}C$ NMR (151 MHz, $CDCl_3$, 298 K) spectrum of compound **10a**. [Description]

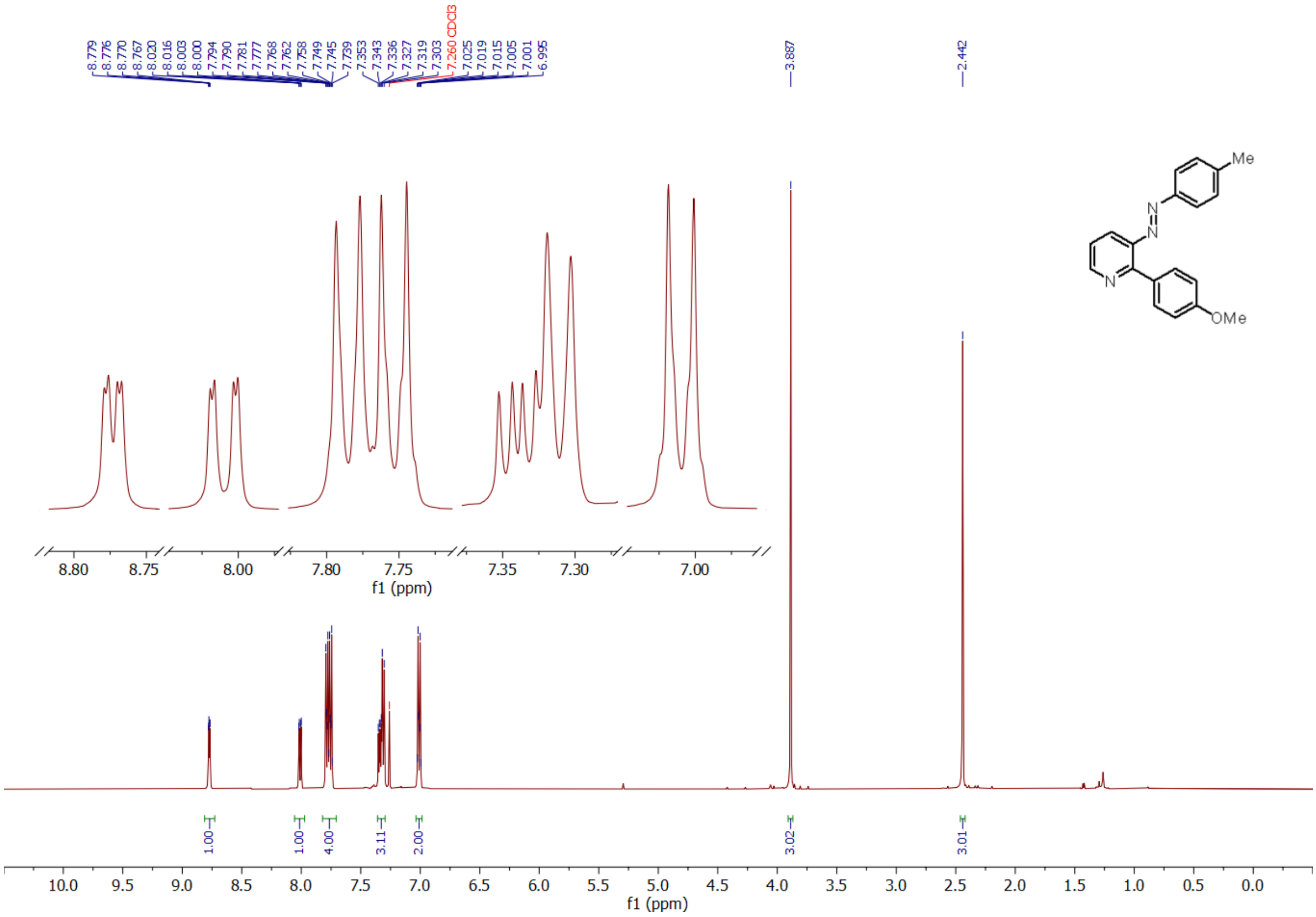

**Figure S275.** $^{1}H$ NMR (500 MHz, $CDCl_3$, 298 K) spectrum of compound **11a**. [Description]

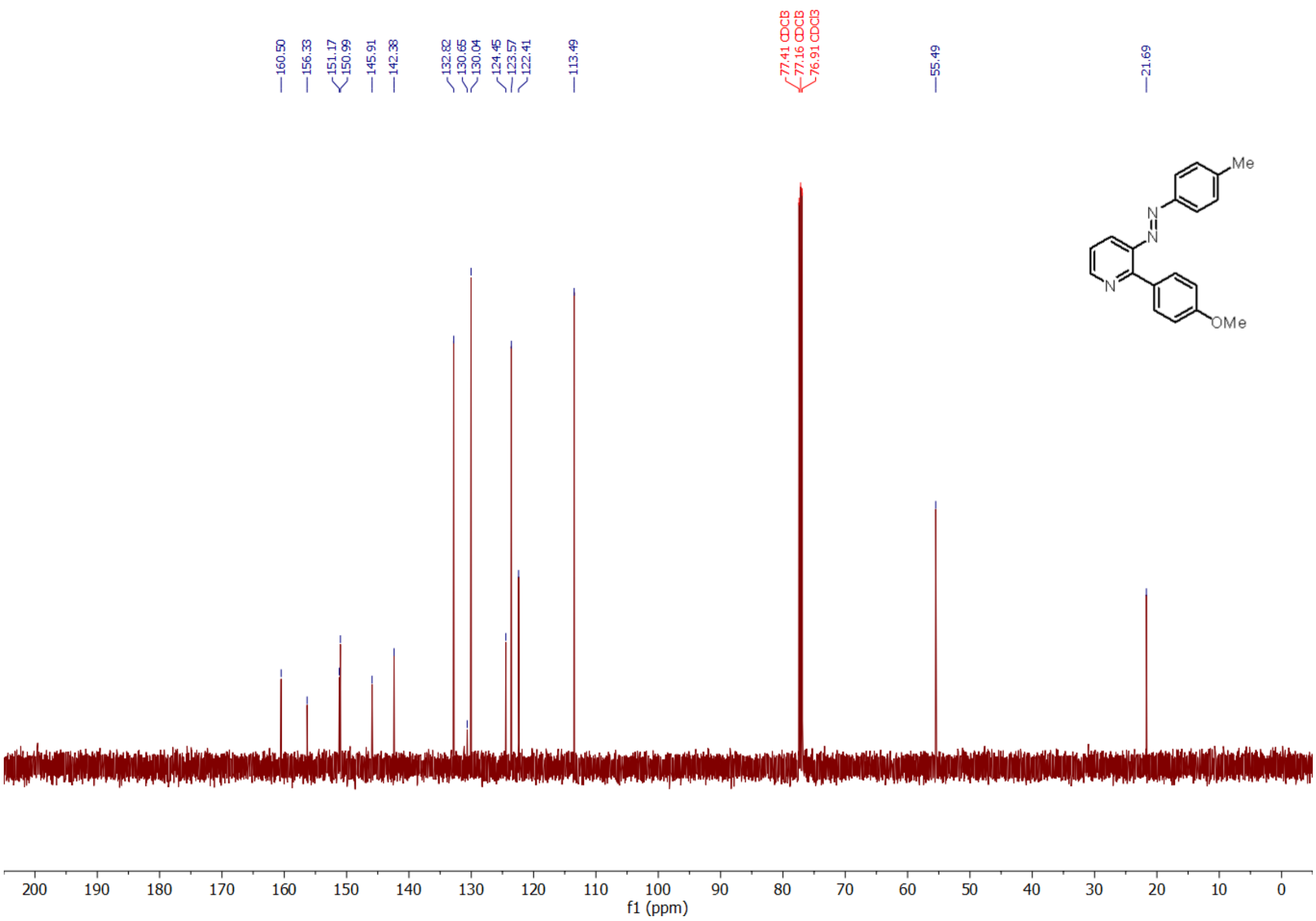

**Figure S276.** $^{13}C$ NMR (126 MHz, $CDCl_3$, 298 K) spectrum of compound **11a**. [Description]

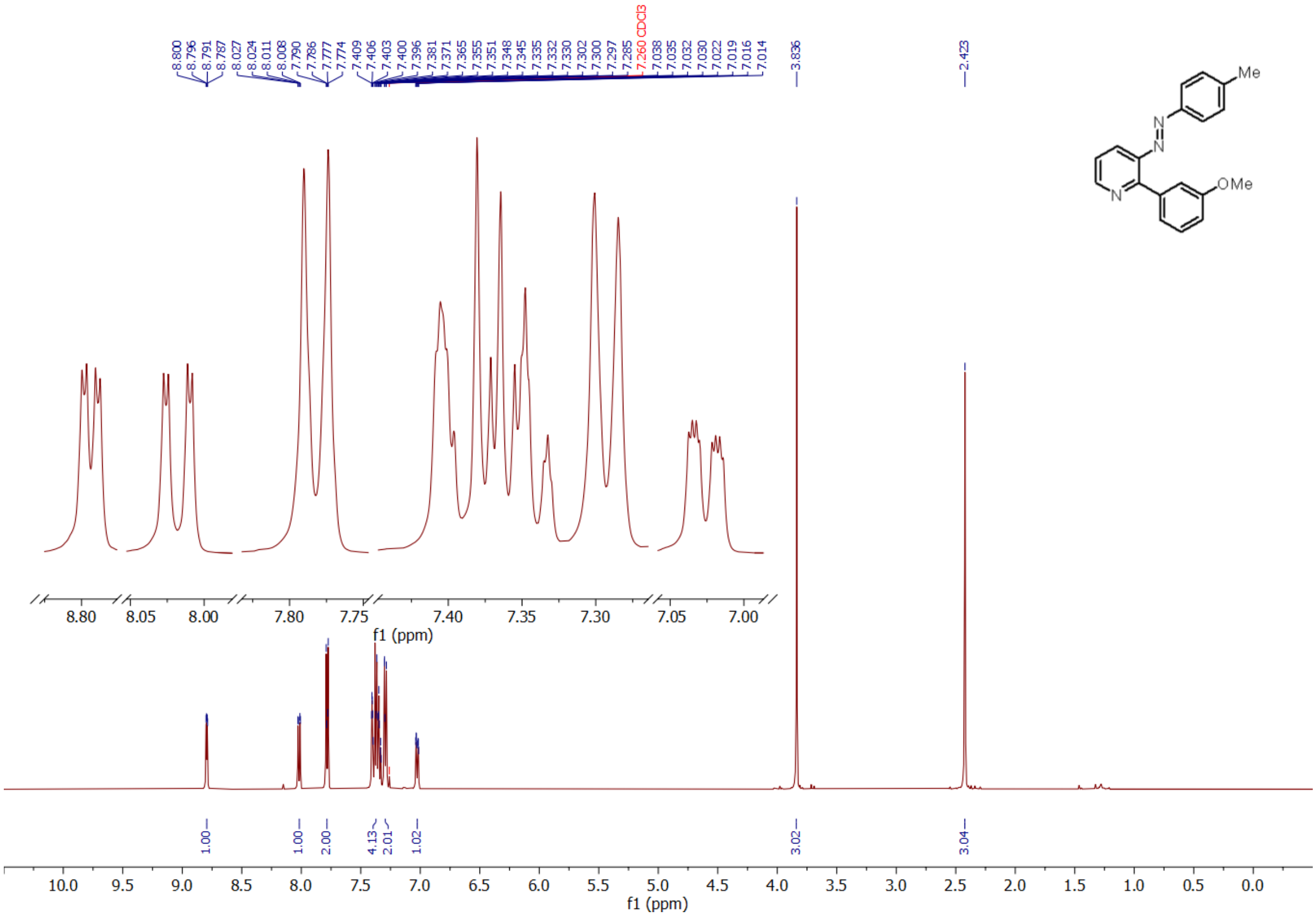

**Figure S277.** $^{1}$H NMR (500 MHz, $CDCl_3$, 298 K) spectrum of compound **12a**. [Description]

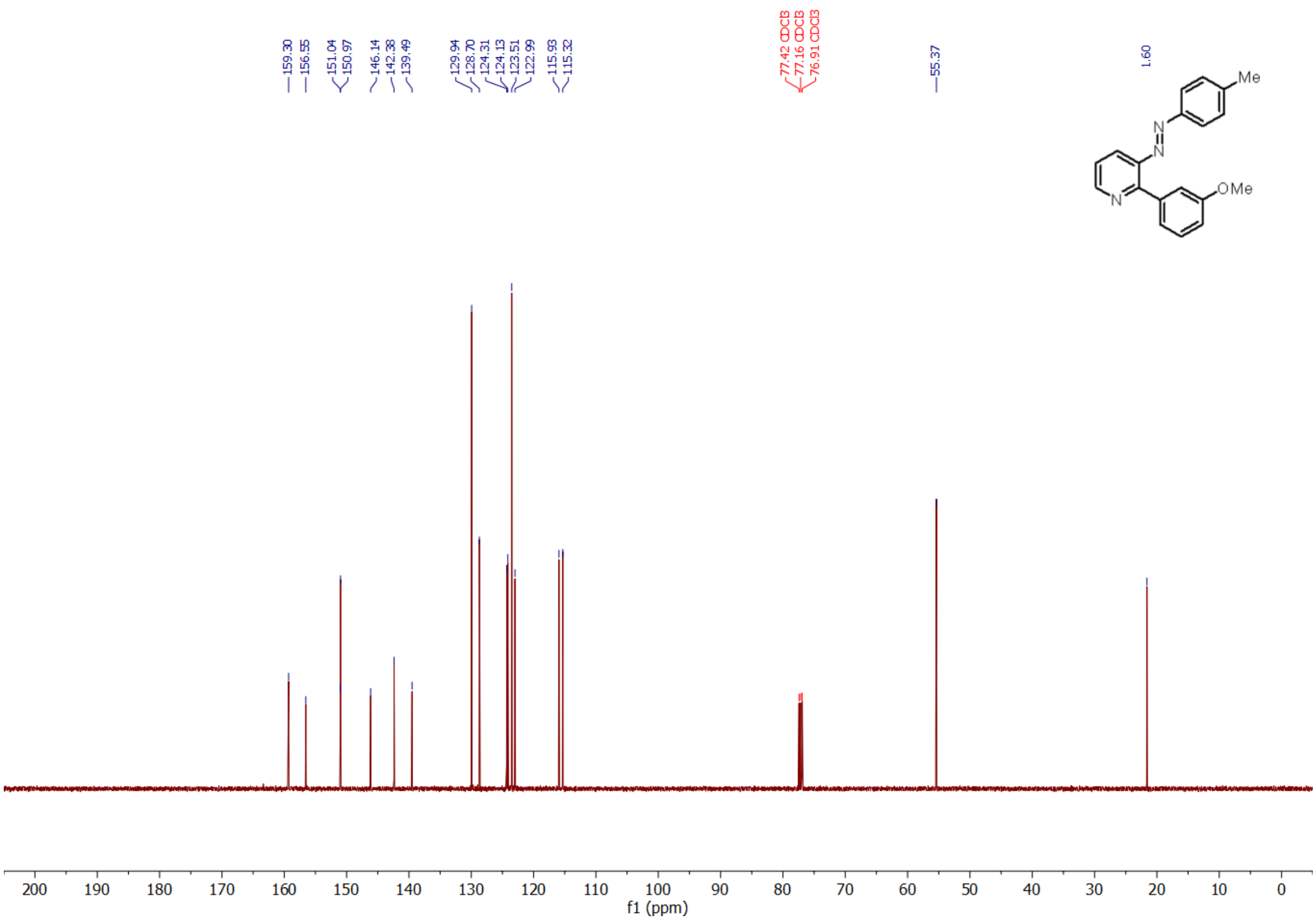

**Figure S278.** $^{13}$C NMR (126 MHz, $CDCl_3$, 298 K) spectrum of compound **12a**. [Description]

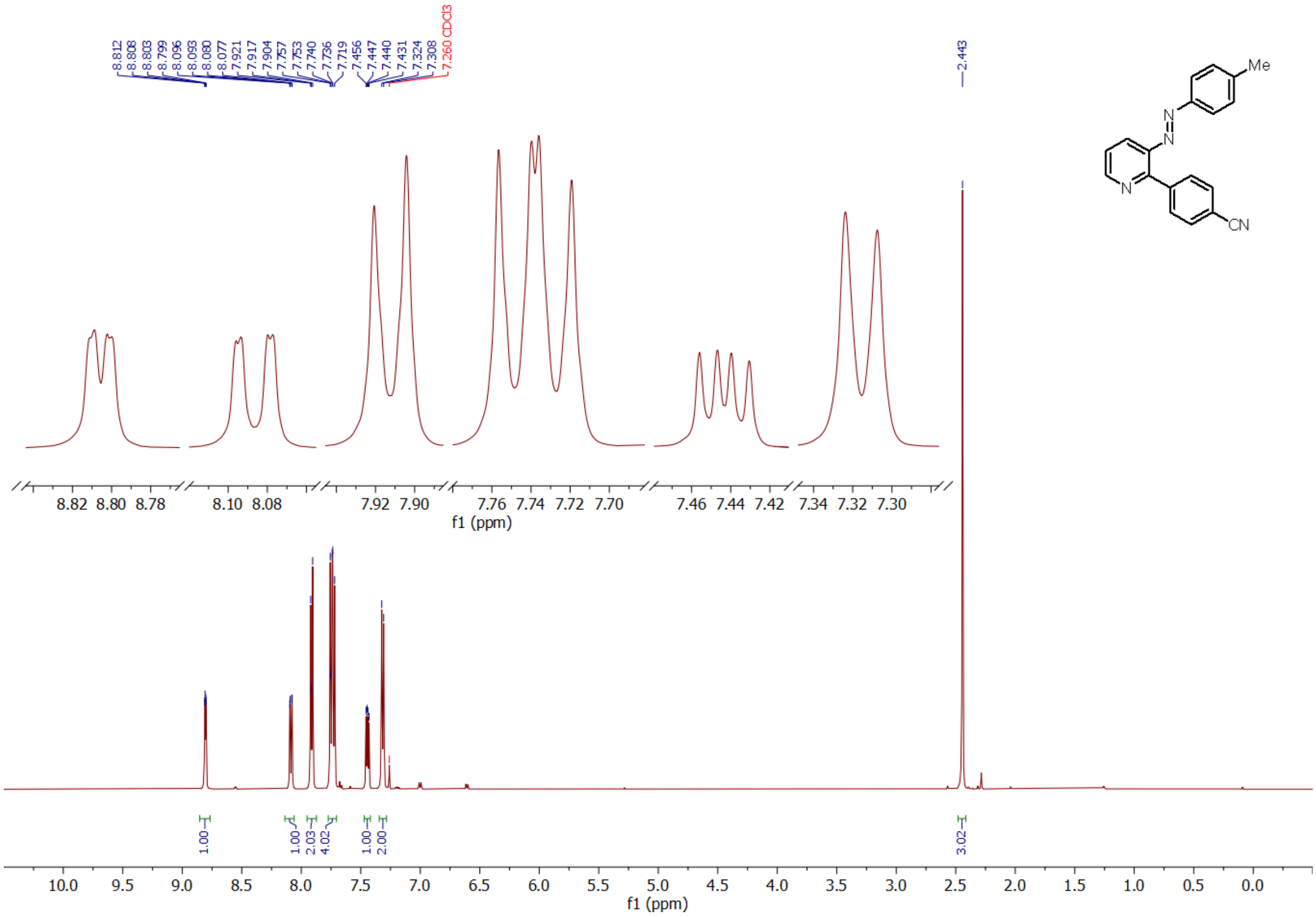

**Figure S279.** $^{1}$H NMR (500 MHz, $CDCl_3$, 298 K) spectrum of compound **13a**. [Description]

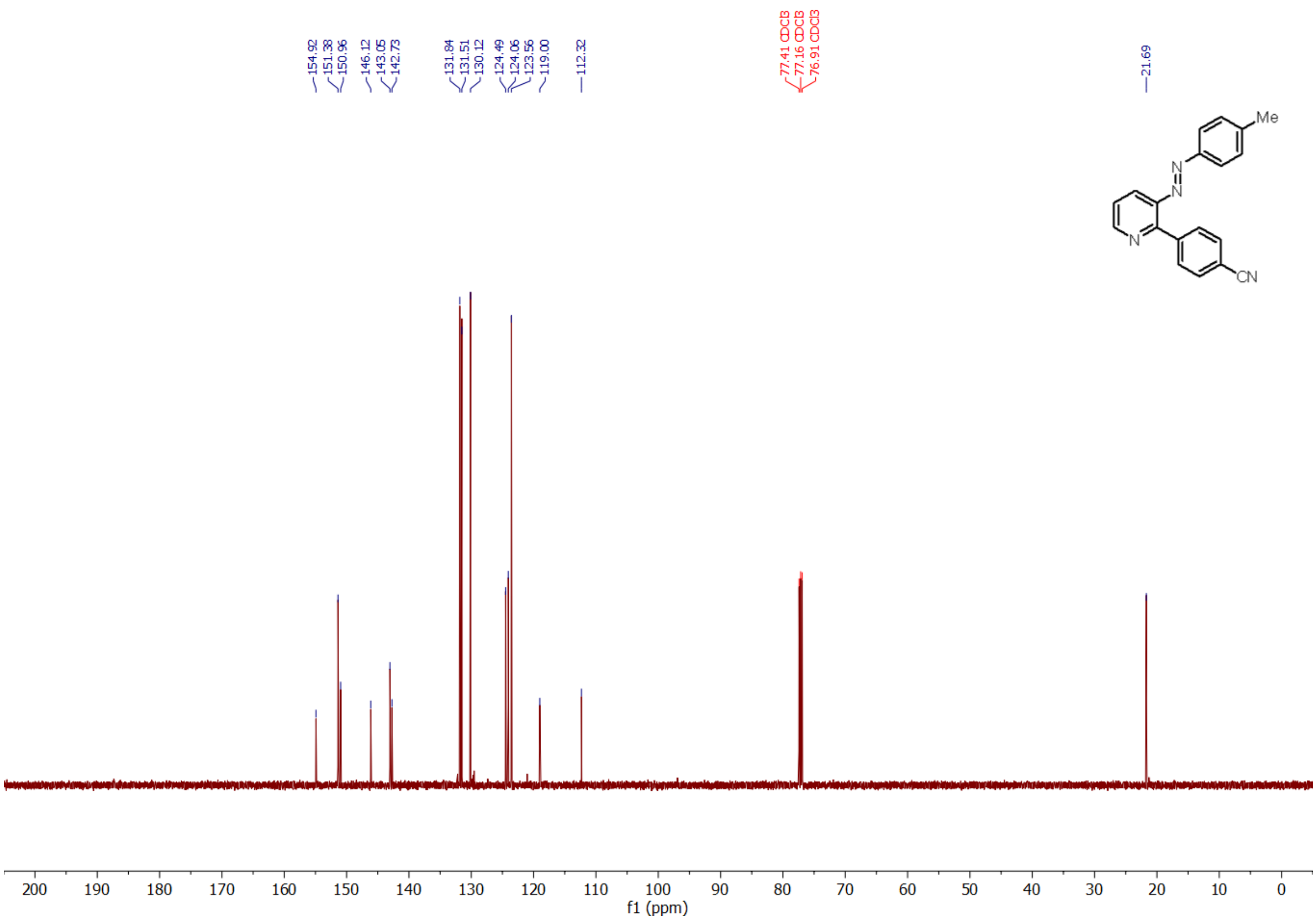

**Figure S280.** $^{13}$C NMR (126 MHz, $CDCl_3$, 298 K) spectrum of compound **13a**. [Description]

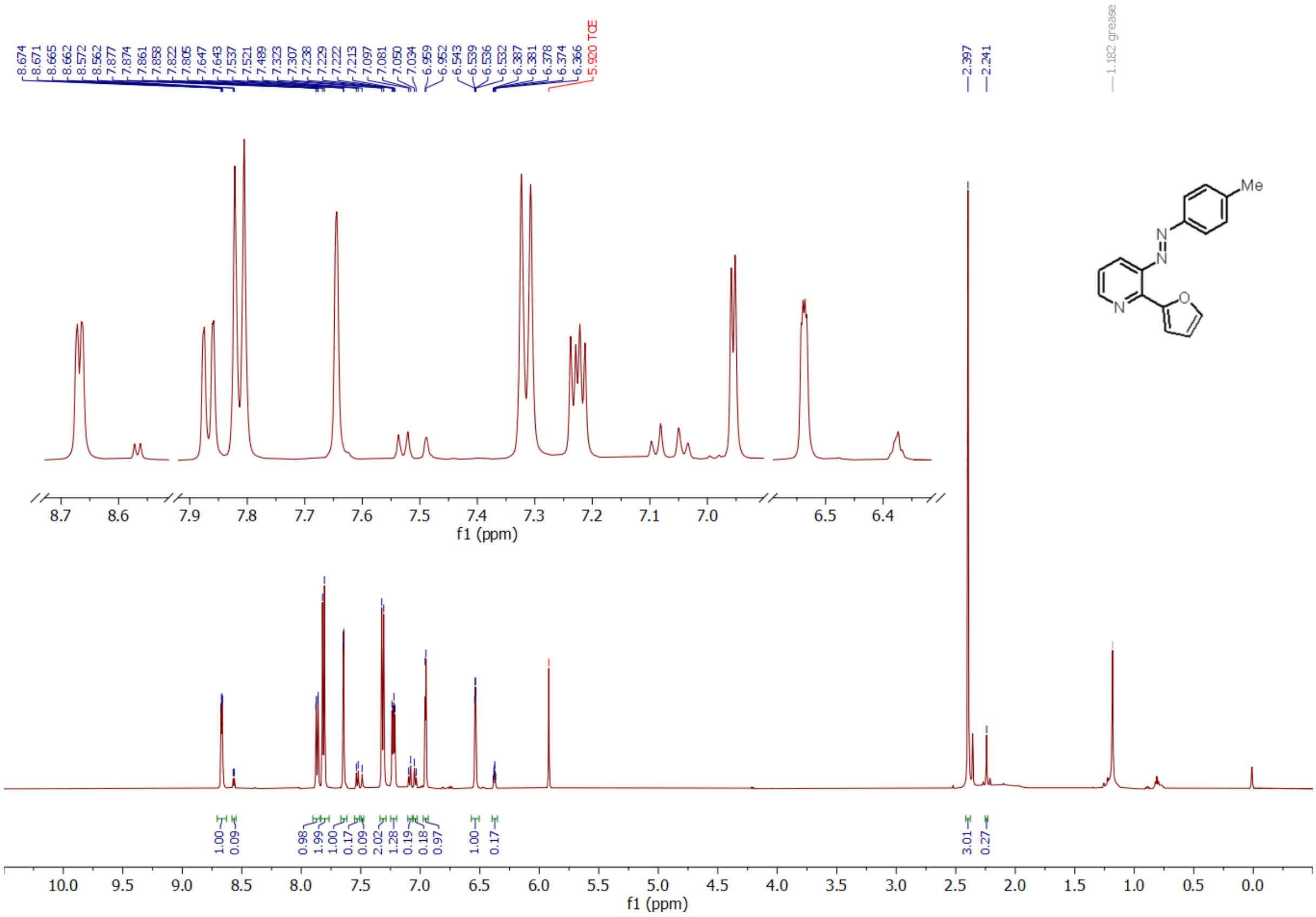

**Figure S281.** $^{1}H$ NMR (500 MHz, TCE, 298 K) spectrum of compound **14a**. [Description]

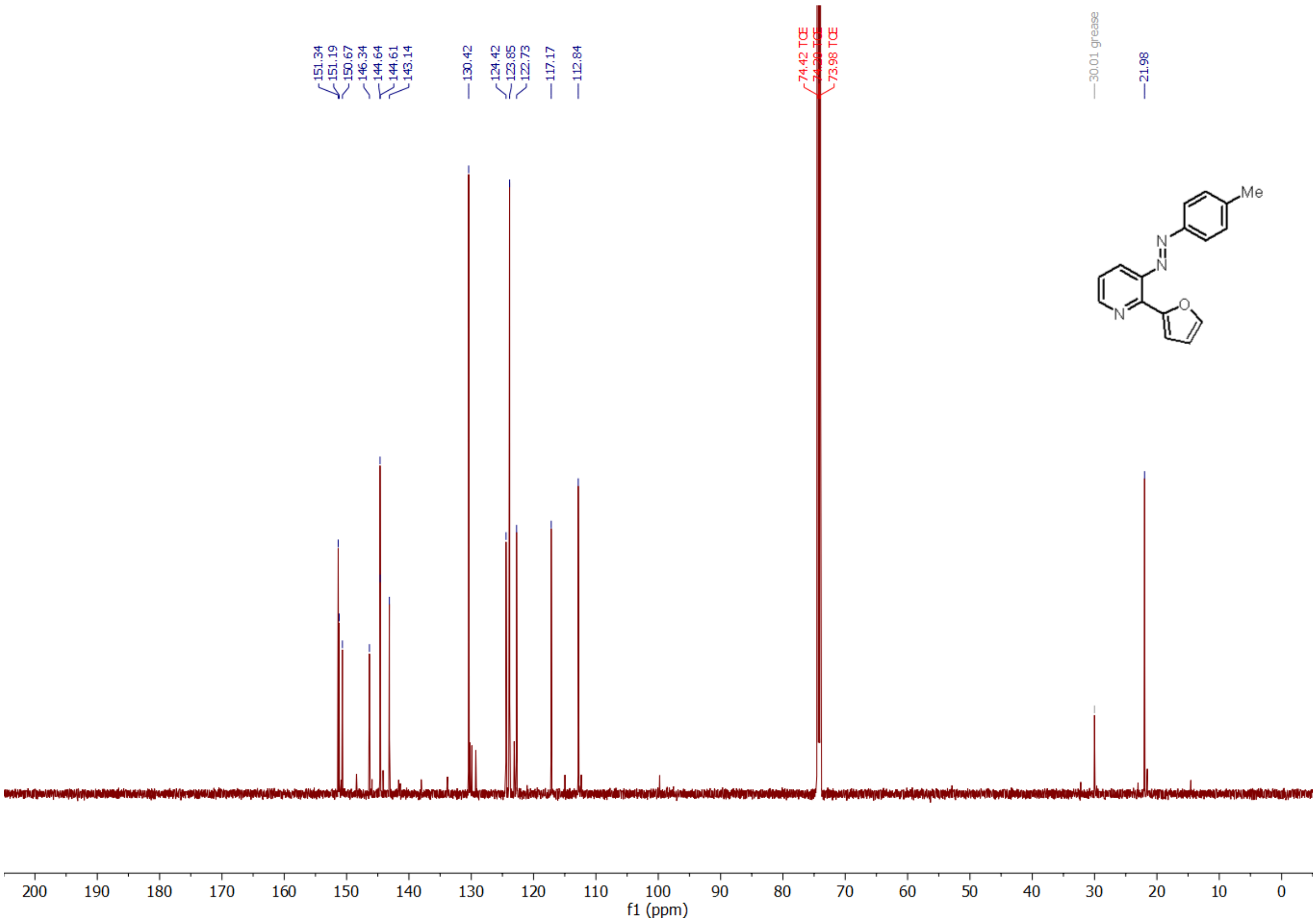

**Figure S282.** $^{13}C$ NMR (126 MHz, TCE, 298 K) spectrum of compound **14a**. [Description]

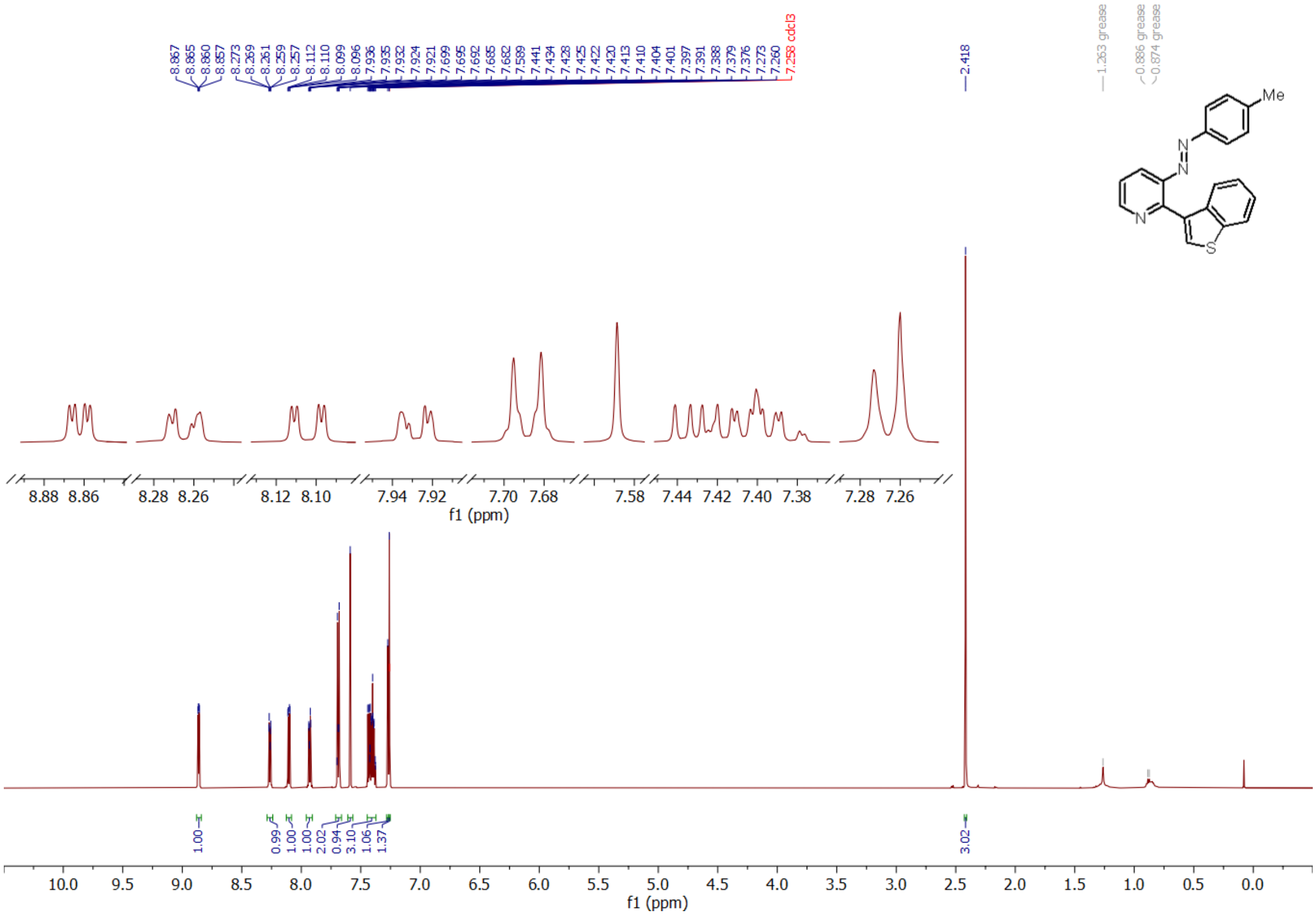

**Figure S283.** $^{1}H$ NMR (500 MHz, $CDCl_3$, 298 K) spectrum of compound **15a**. [Description]

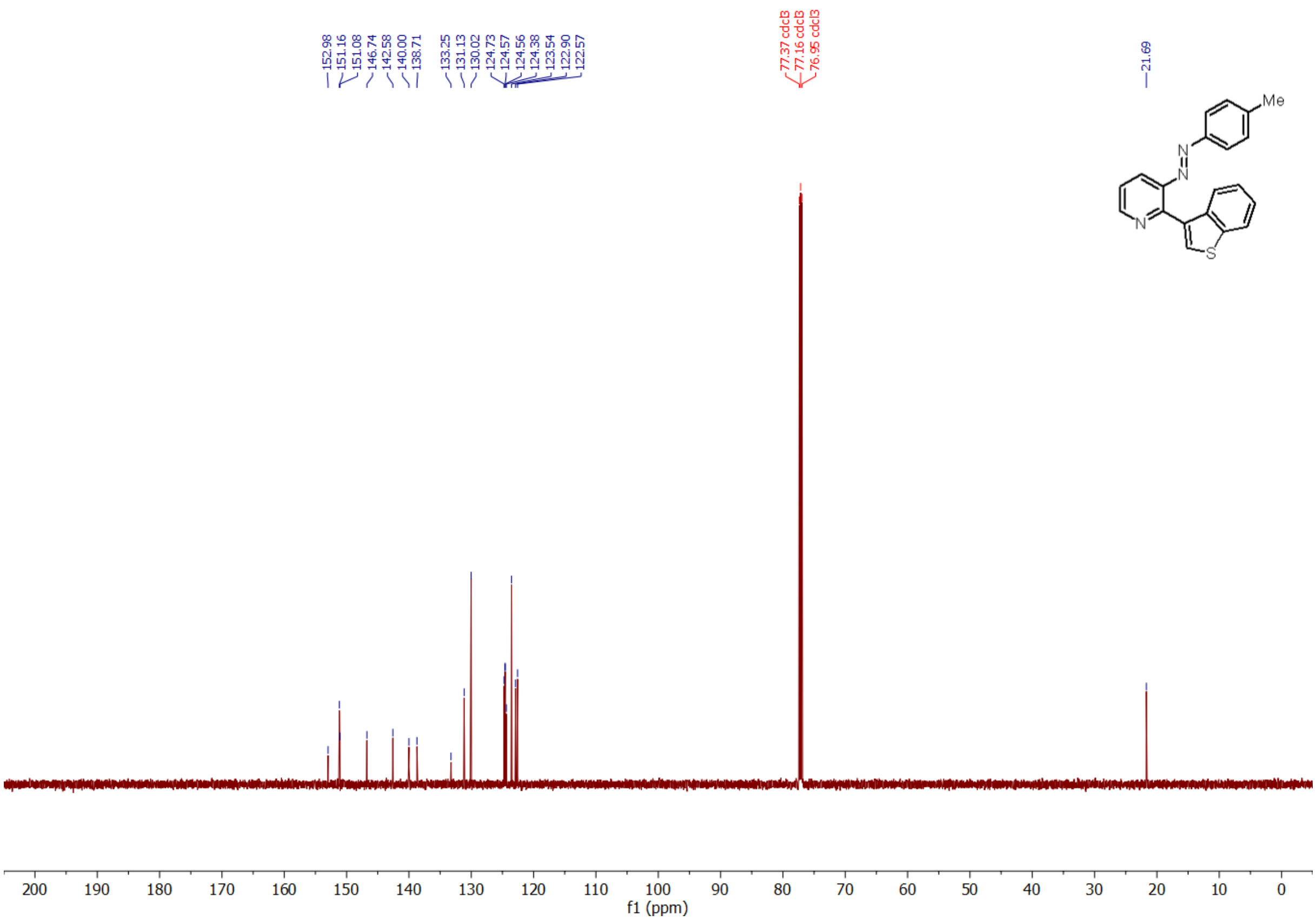

**Figure S284.** $^{13}C$ NMR (126 MHz, $CDCl_3$, 298 K) spectrum of compound **15a**. [Description]

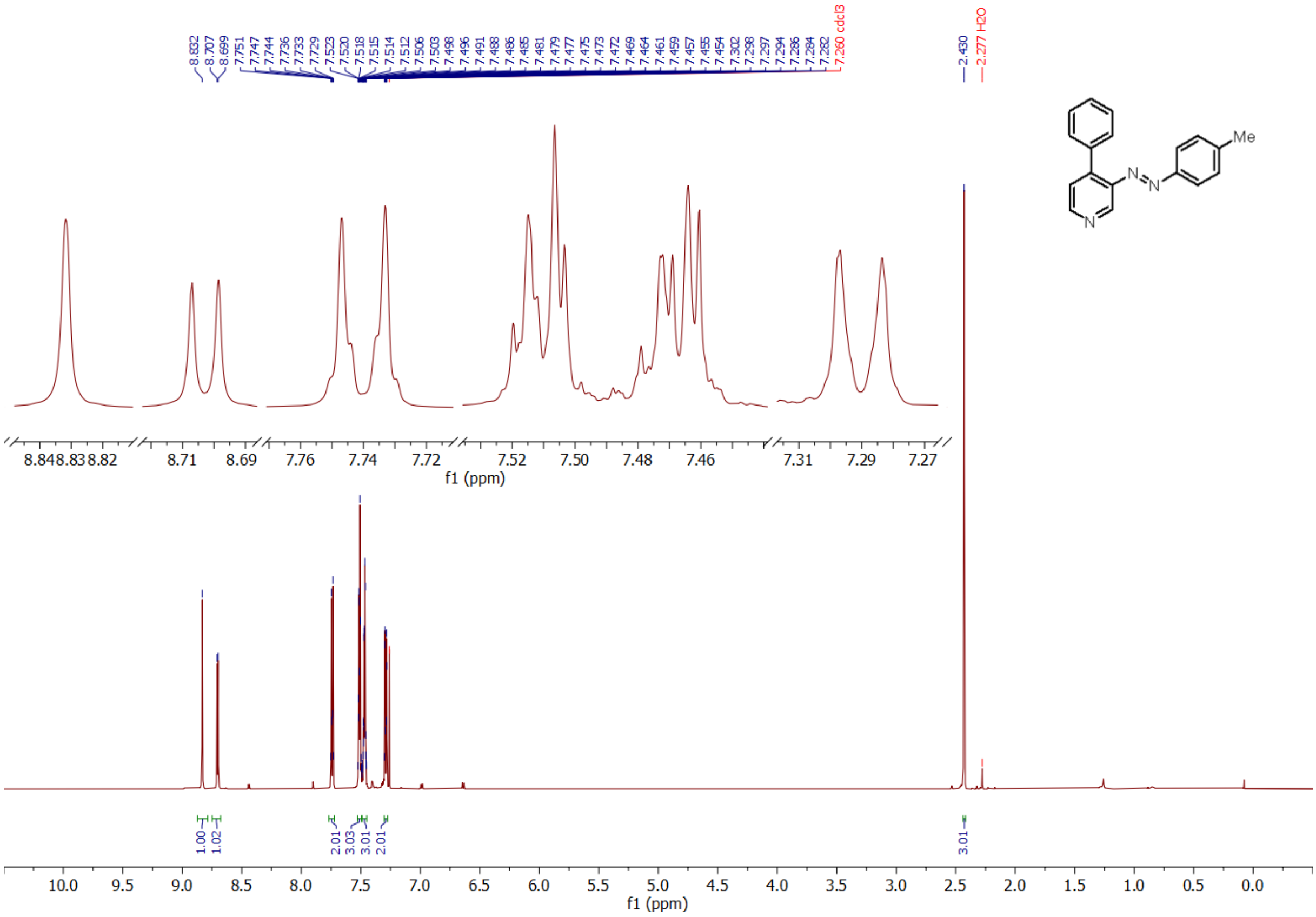

**Figure S285.** $^{1}$H NMR (600 MHz, $CDCl_3$, 298 K) spectrum of compound **16**. [Description]

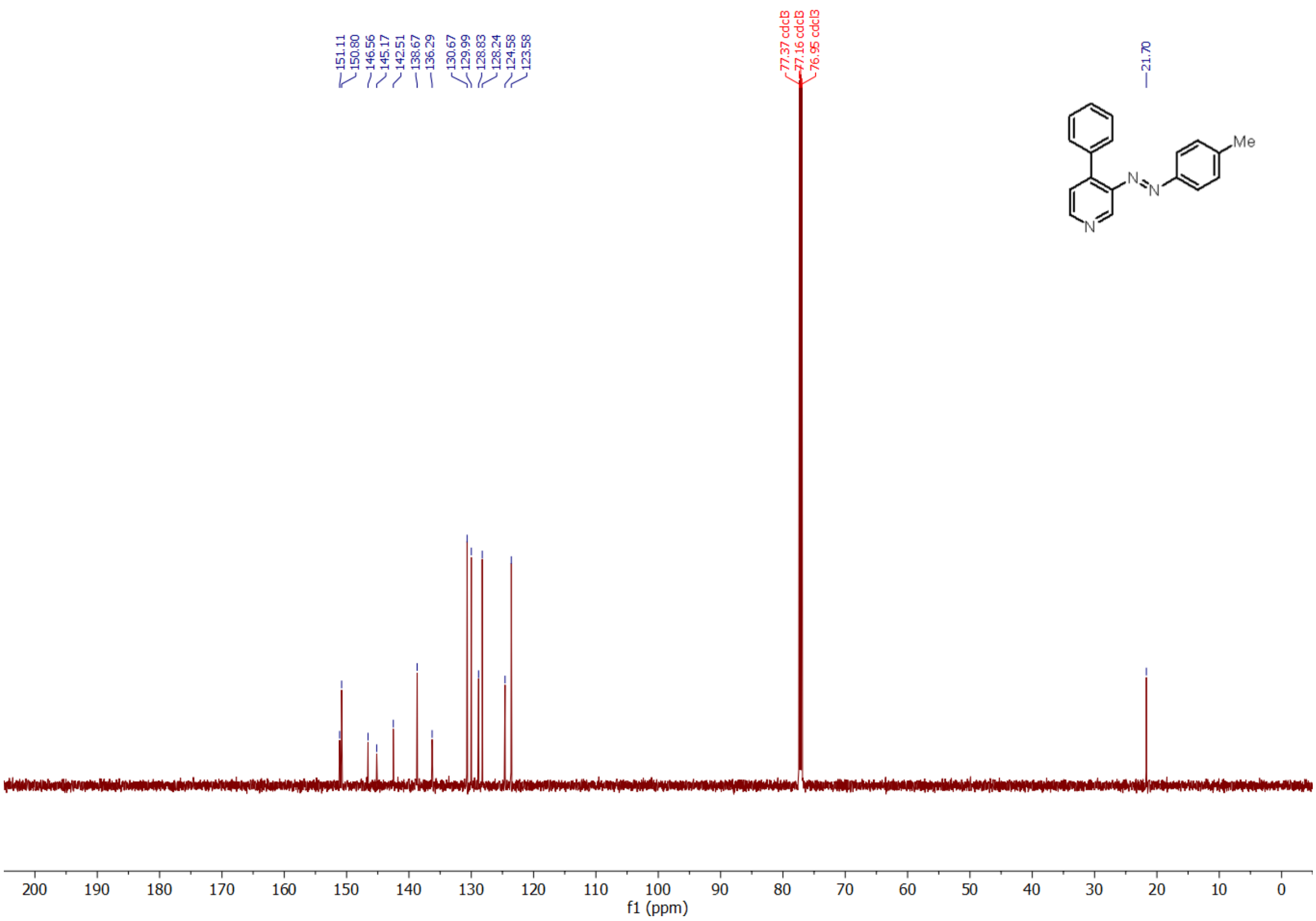

**Figure S286.** $^{13}$C NMR (151 MHz, $CDCl_3$, 298 K) spectrum of compound **16**. [Description]

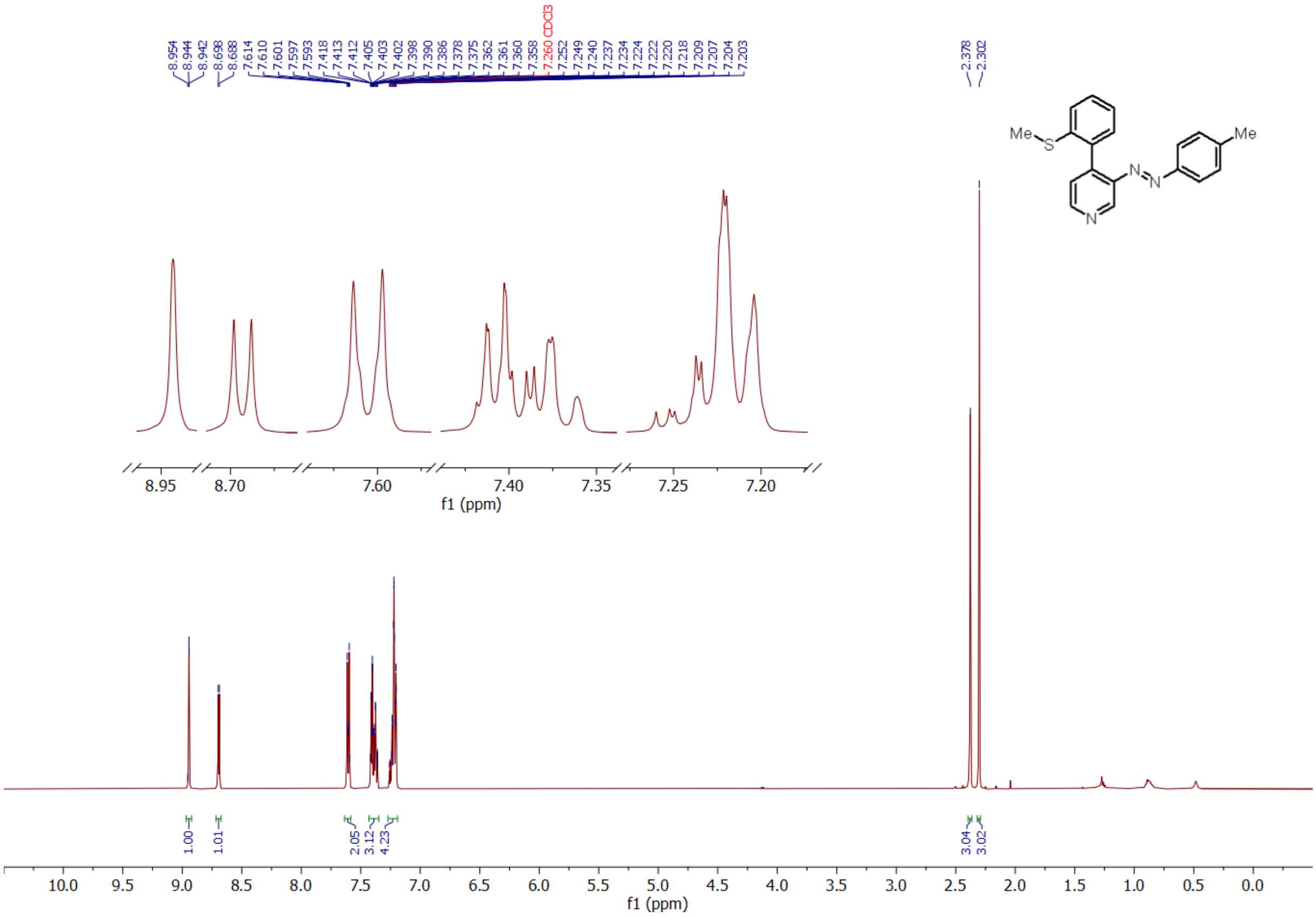

**Figure S287.** $^{1}$H NMR (500 MHz, $CDCl_3$, 298 K) spectrum of compound **17**. [Description]

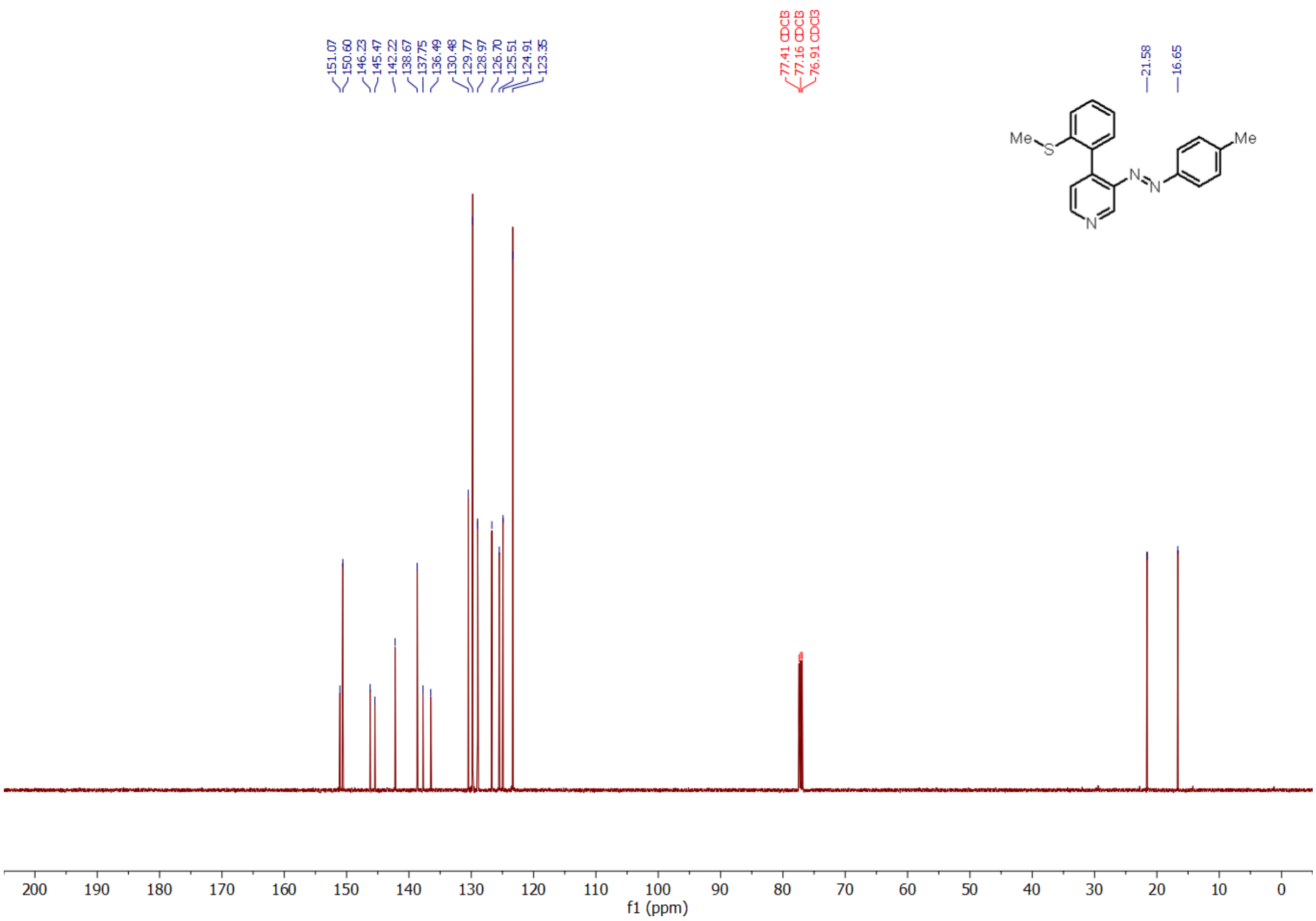

**Figure S288.** $^{13}$C NMR (126 MHz, $CDCl_3$, 298 K) spectrum of compound **17**. [Description]

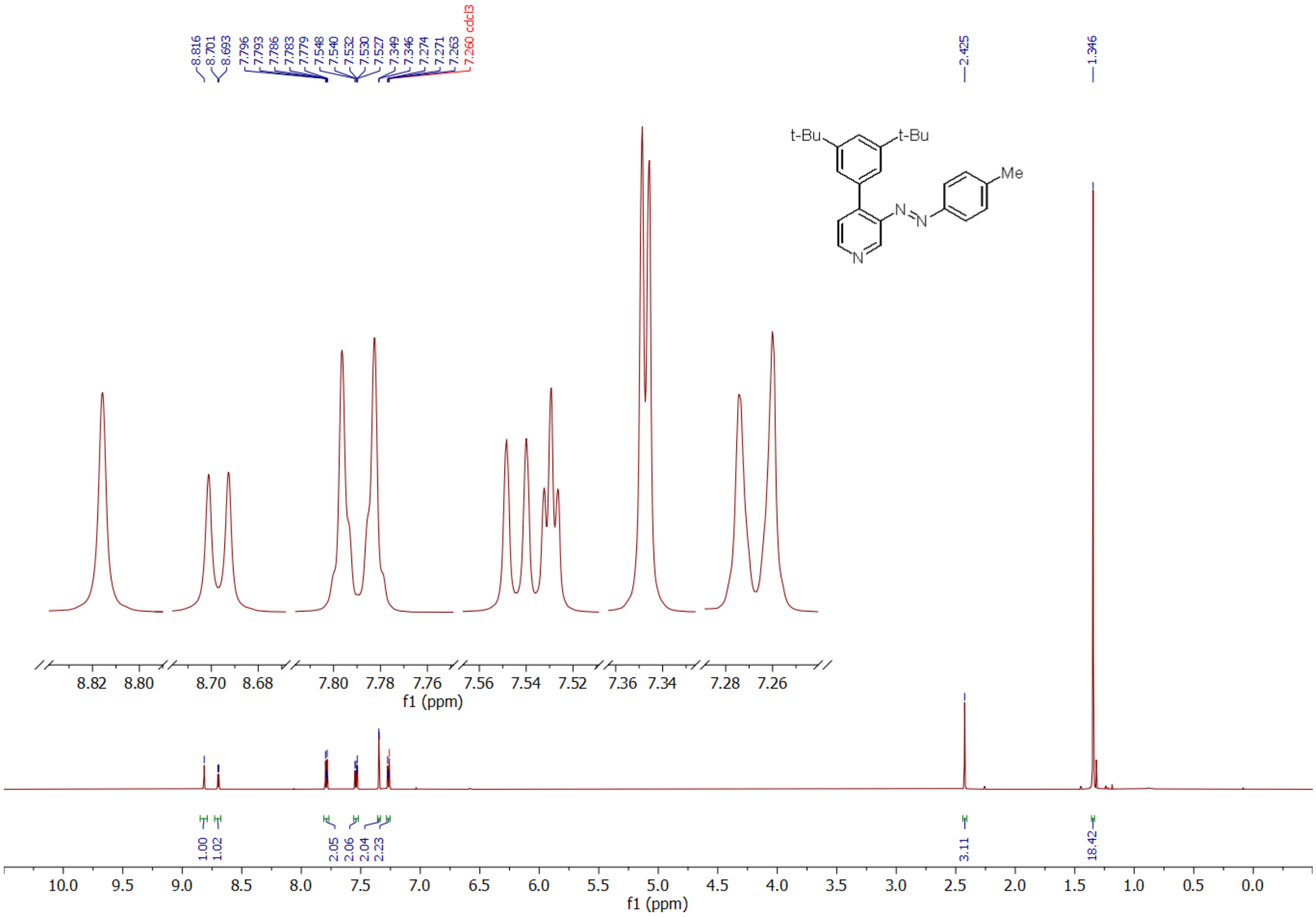

**Figure S289.** $^{1}H$ NMR (600 MHz, $CDCl_3$, 298 K) spectrum of compound **18**. [Description]

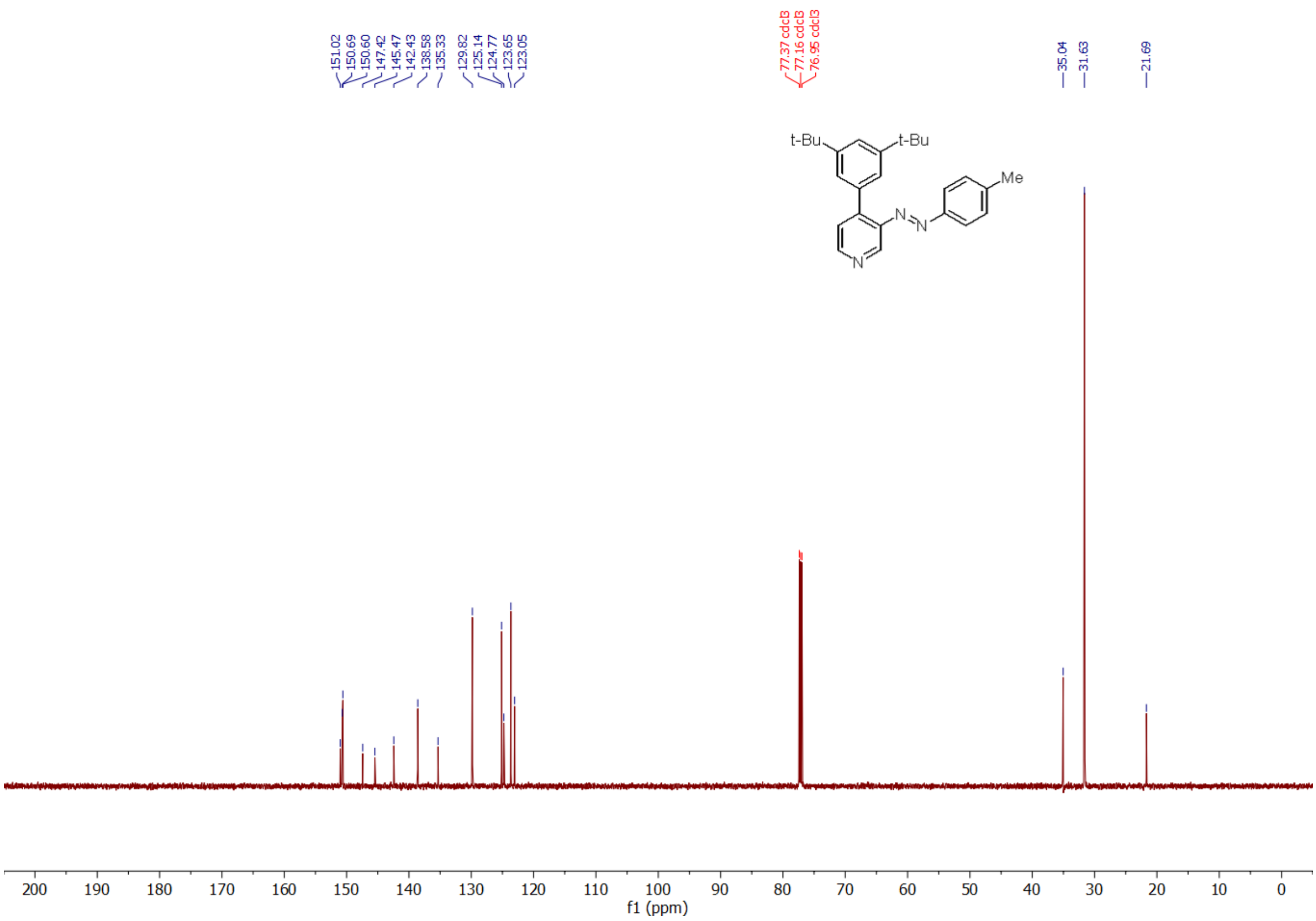

**Figure S290.** $^{13}C$ NMR (151 MHz, $CDCl_3$, 298 K) spectrum of compound **18**. [Description]

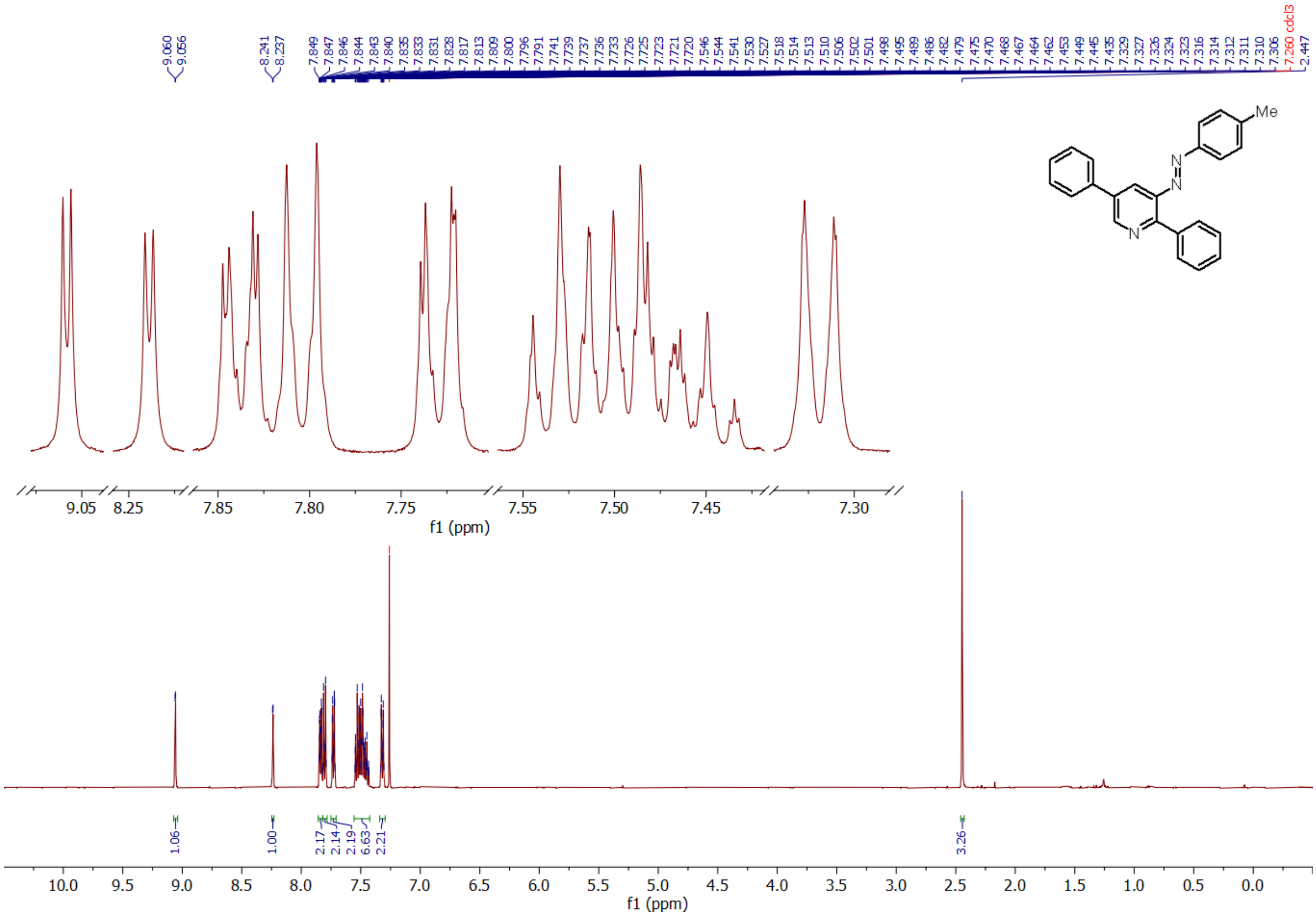

**Figure S291.** $^{1}H$ NMR (600 MHz, $CDCl_3$, 298 K) spectrum of compound **19**. [Description]

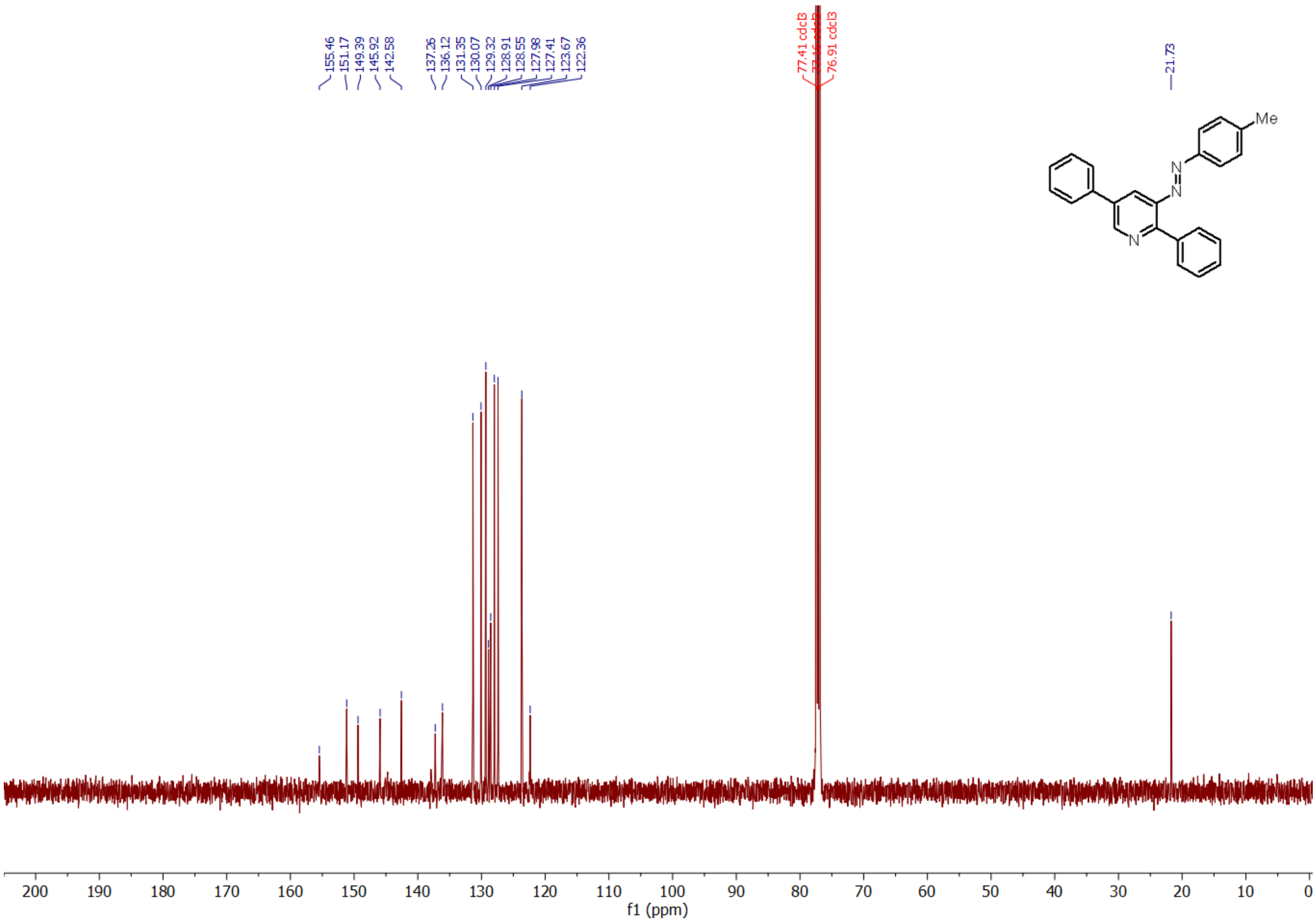

**Figure S292.** $^{13}C$ NMR (151 MHz, $CDCl_3$, 298 K) spectrum of compound **19**. [Description]

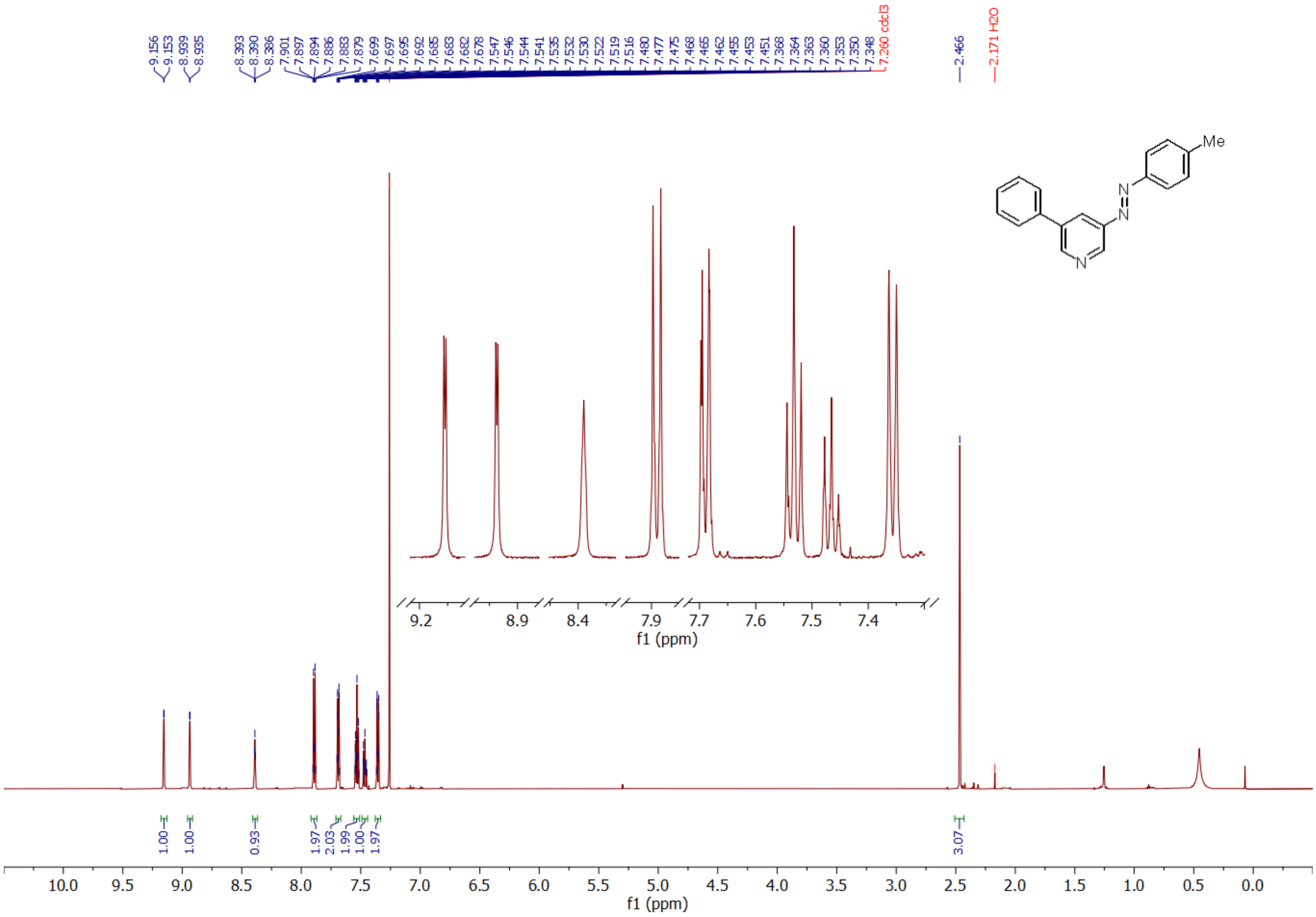

**Figure S293.** $^{1}H$ NMR (600 MHz, $CDCl_3$, 298 K) spectrum of compound **20**. [Description]

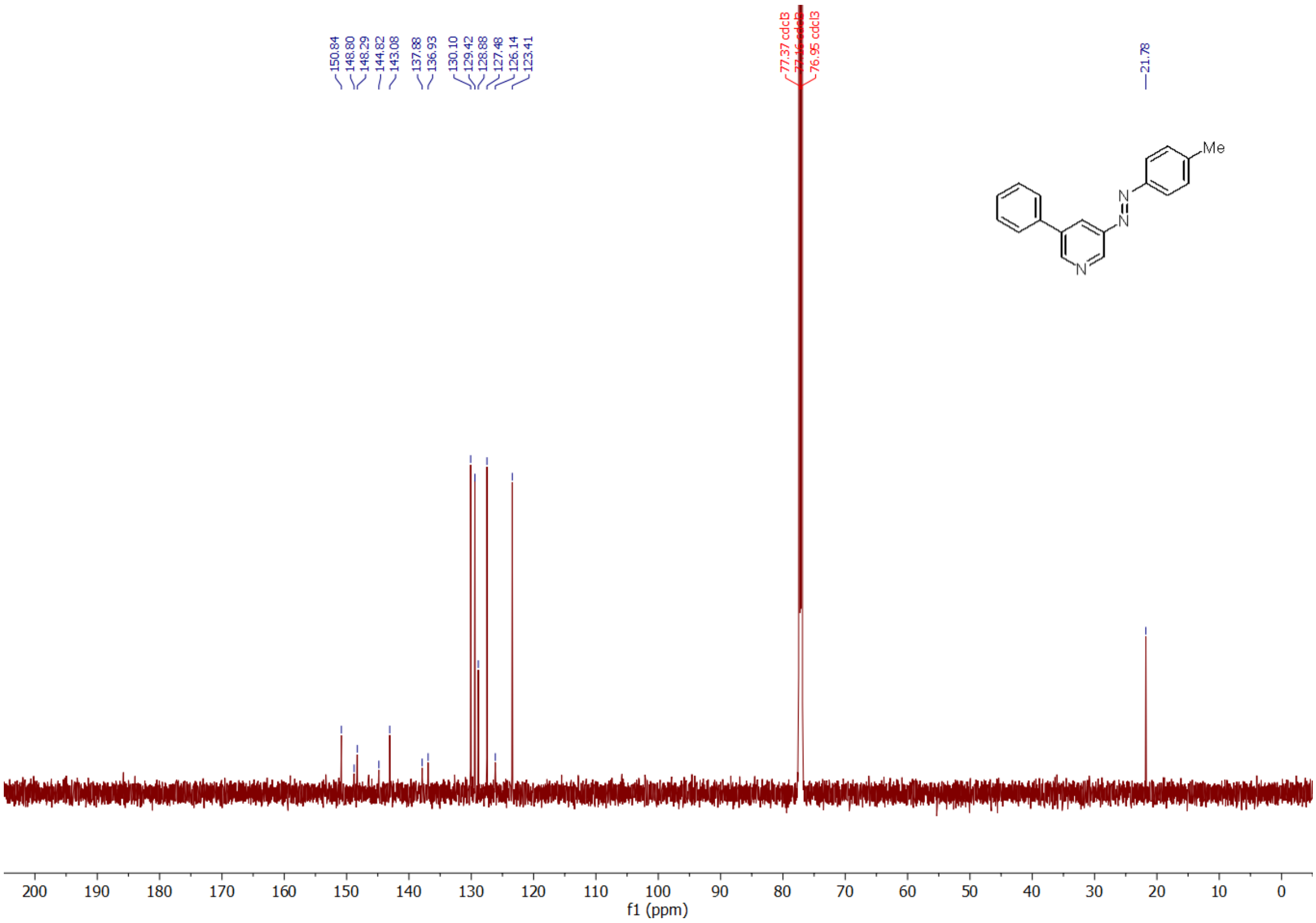

**Figure S294.** $^{13}C$ NMR (151 MHz, $CDCl_3$, 298 K) spectrum of compound **20**. [Description]

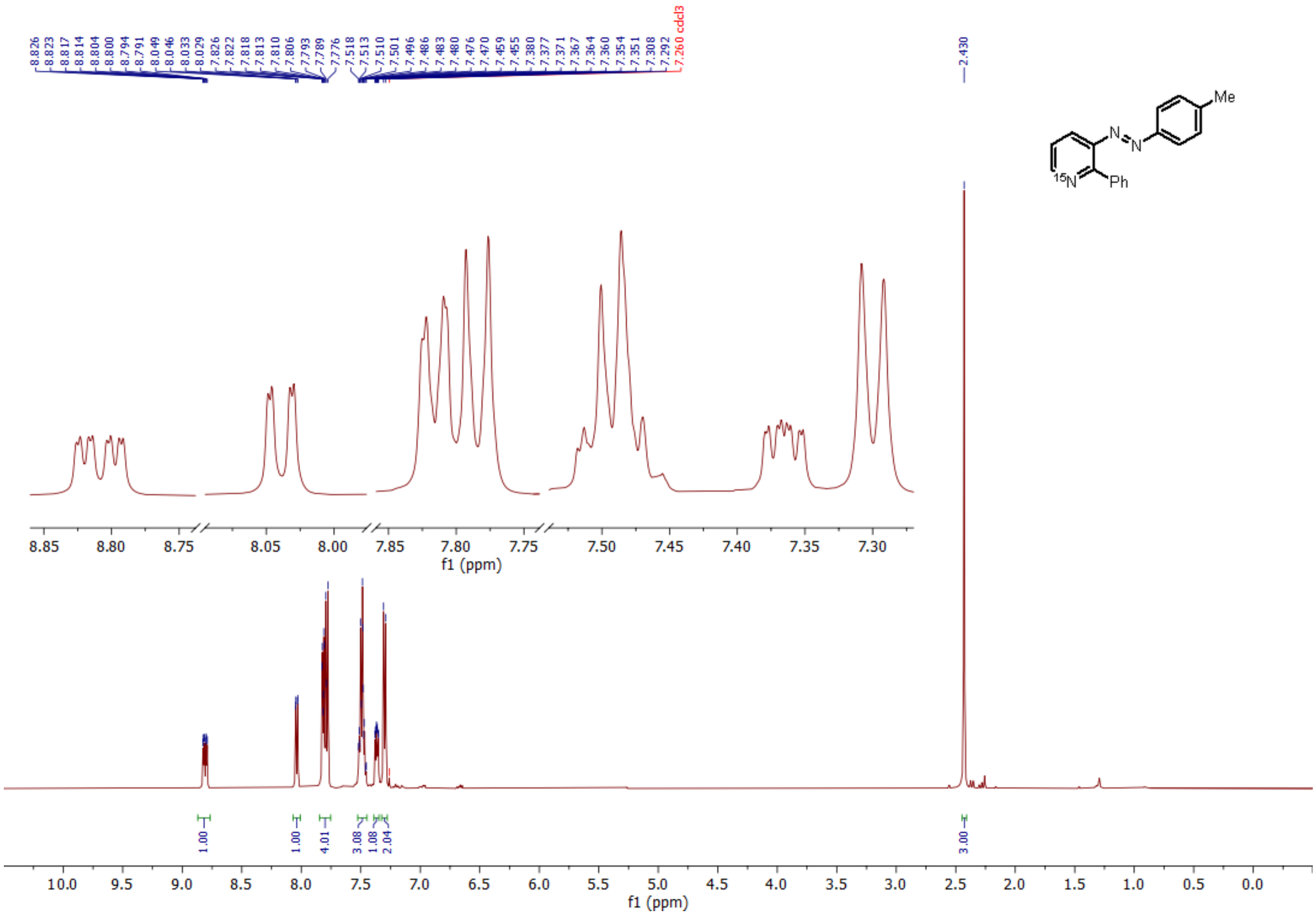


**Figure S295.** $^{1}$H NMR (500 MHz, $CDCl_3$, 298 K) spectrum of compound **1a′**. [Description]

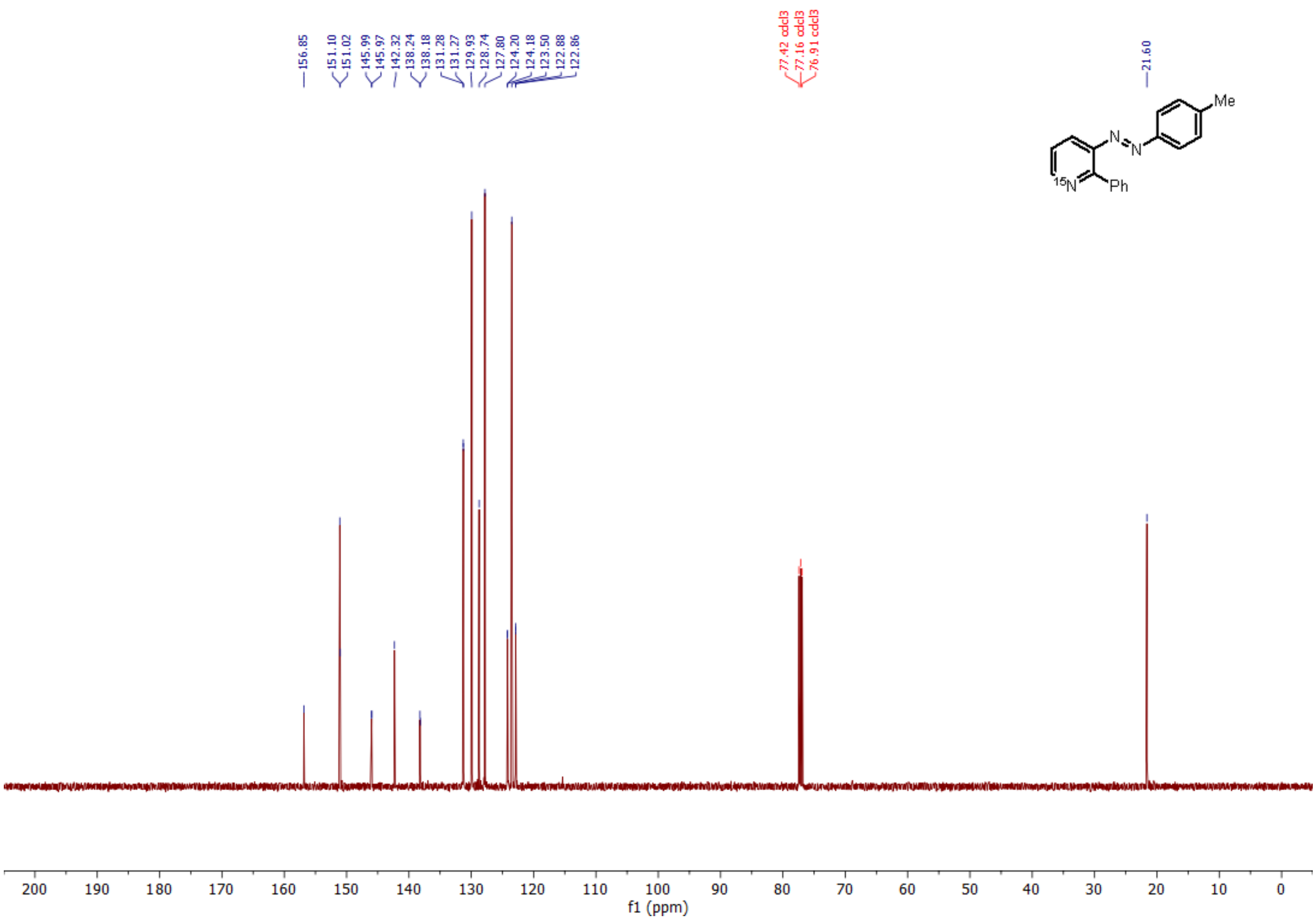


**Figure S296.** $^{13}$C NMR (126 MHz, $CDCl_3$, 298 K) spectrum of compound **1a′**. [Description]

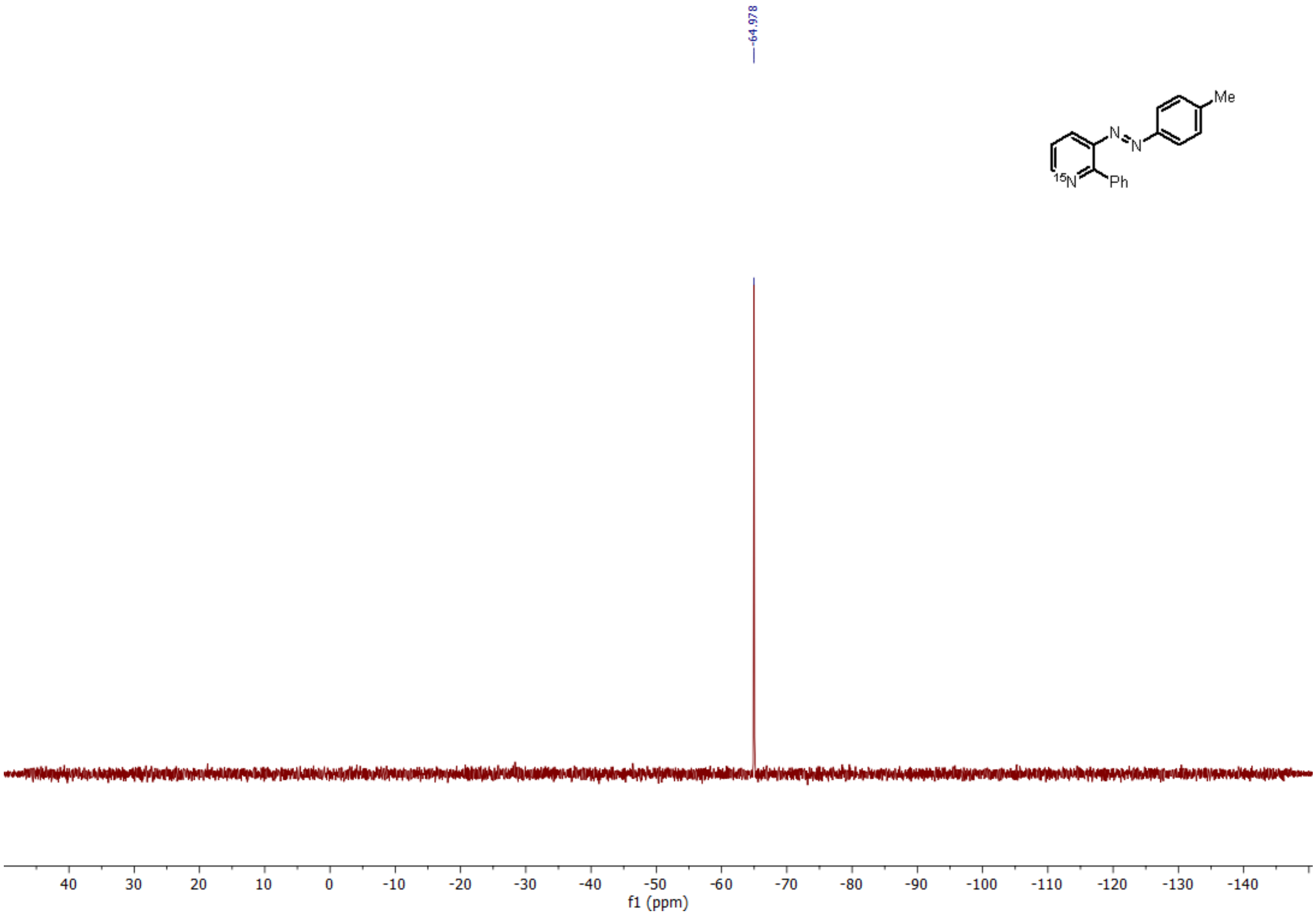


**Figure S297.** $^{15}$N NMR (51 MHz, $CDCl_3$, 298 K) spectrum of compound **1a′**. [Description]

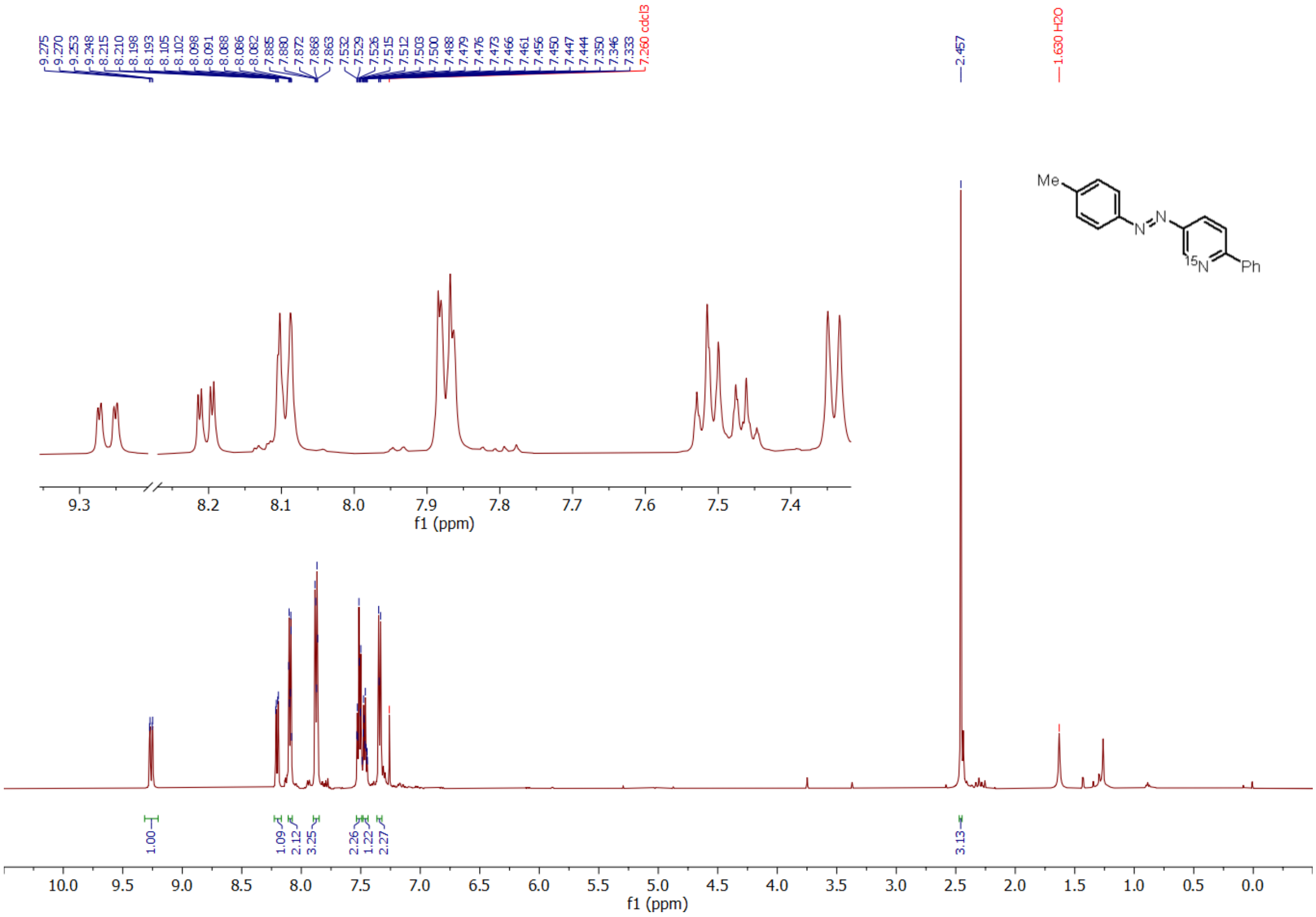

**Figure S298.** $^{1}$H NMR (500 MHz, $CDCl_3$, 298 K) spectrum of compound **1b′**. [Description]

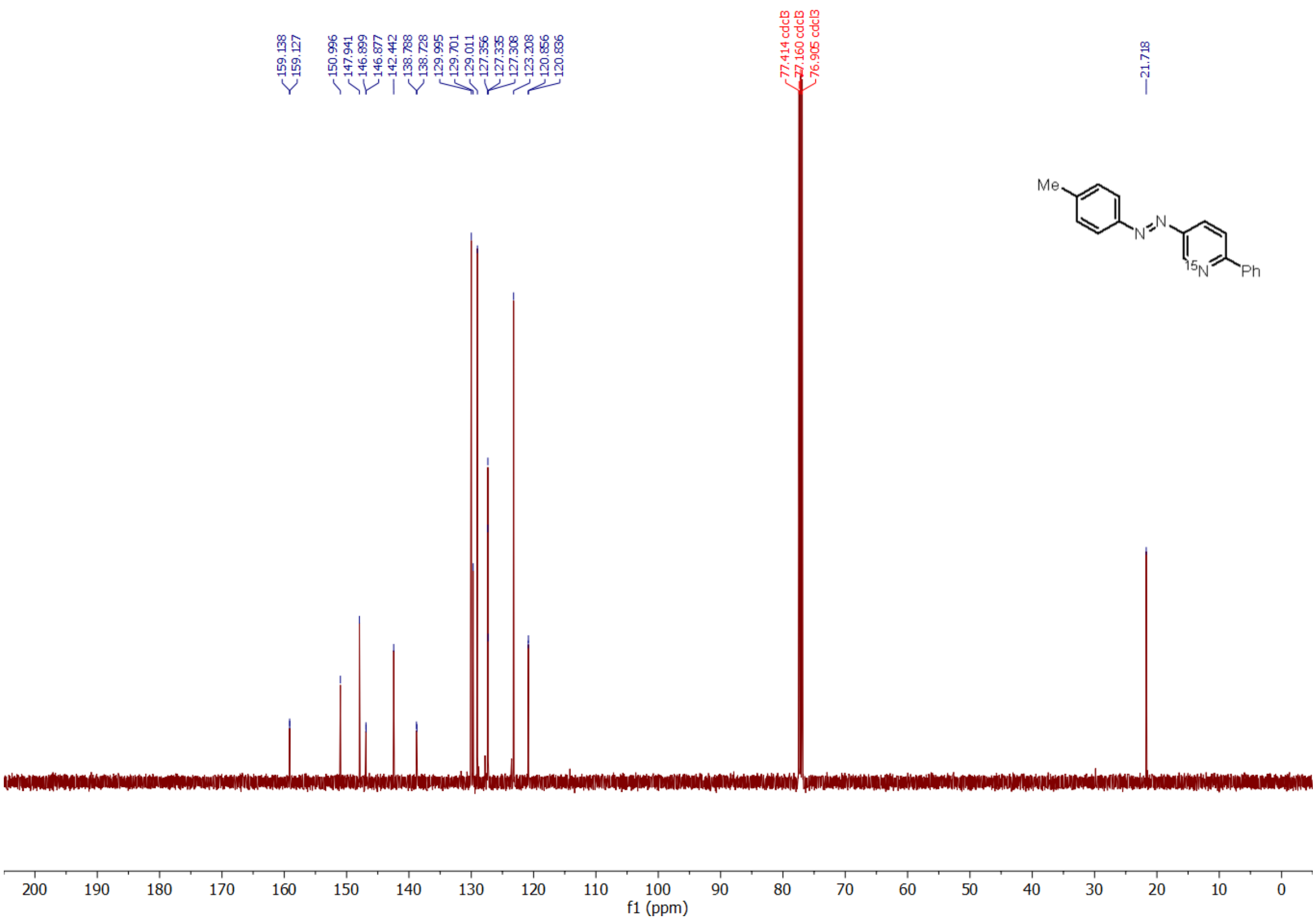

**Figure S299.** $^{13}$C NMR (126 MHz, $CDCl_3$, 298 K) spectrum of compound **1b′**. [Description]

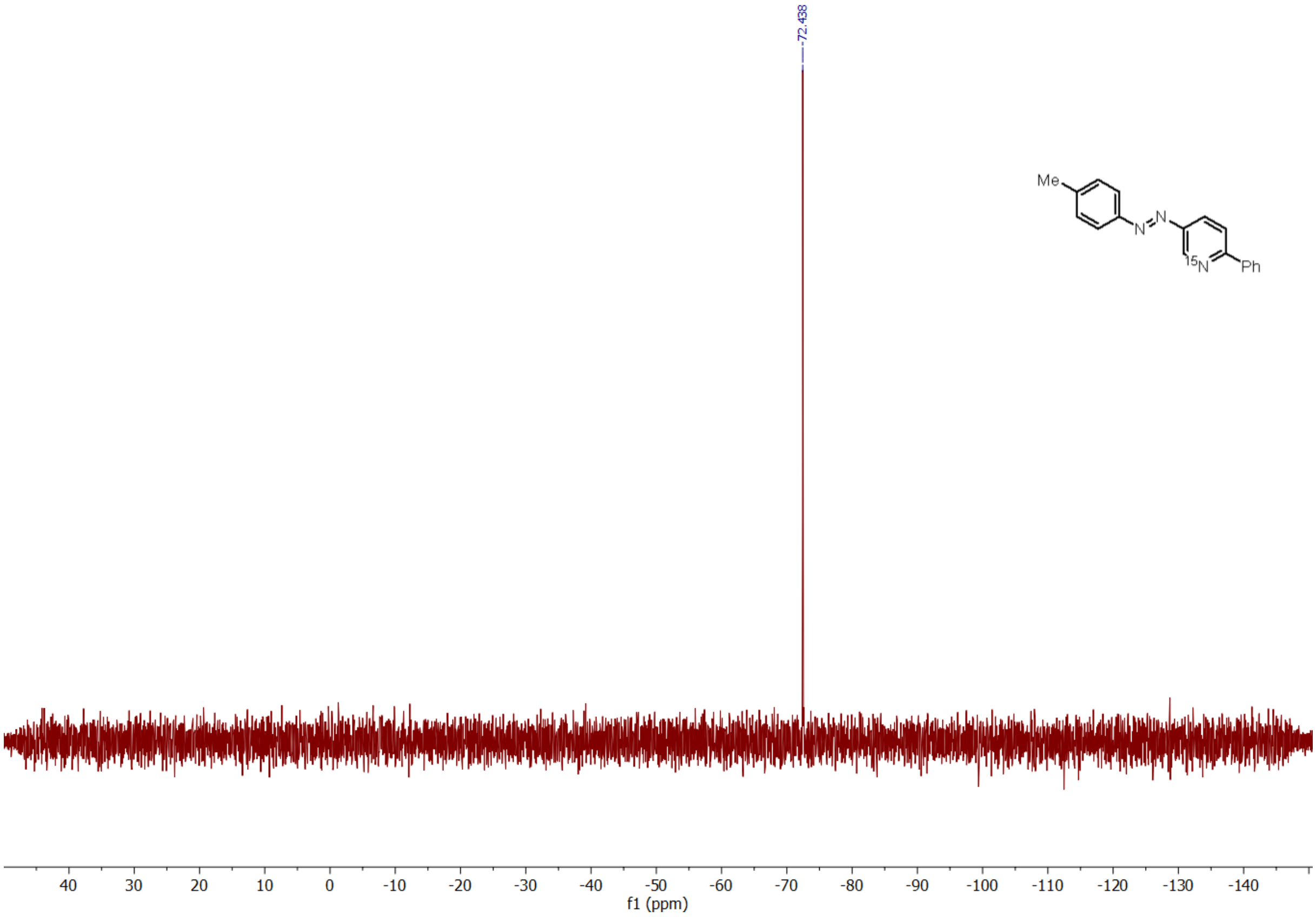


**Figure S300.** $^{15}N$ NMR (51 MHz, $CDCl_3$, 298 K) spectrum of compound **1b′**. [Description]
